# The Impacts of Surgery and Intracerebral Electrodes in C57BL/6J Mouse Kainate Model of Epileptogenesis: Seizure Threshold, Proteomics, and Cytokine Profiles

**DOI:** 10.3389/fneur.2021.625017

**Published:** 2021-07-12

**Authors:** Karen Tse, Edward Beamer, Deborah Simpson, Robert J. Beynon, Graeme J. Sills, Thimmasettappa Thippeswamy

**Affiliations:** ^1^Department of Musculoskeletal Biology, Institute of Ageing and Chronic Disease, University of Liverpool, Liverpool, United Kingdom; ^2^Department of Molecular and Clinical Pharmacology, Institute of Translational Medicine, University of Liverpool, Liverpool, United Kingdom; ^3^Centre for Proteome Research, Institute of Integrative Biology, University of Liverpool, Liverpool, United Kingdom

**Keywords:** intracerebral electrodes, traumatic brain injury, neuroinflammation, proinflammatory cytokines, epilepsy, blood-brain barrier, seizure threshold

## Abstract

Intracranial electroencephalography (EEG) is commonly used to study epileptogenesis and epilepsy in experimental models. Chronic gliosis and neurodegeneration at the injury site are known to be associated with surgically implanted electrodes in both humans and experimental models. Currently, however, there are no reports on the impact of intracerebral electrodes on proteins in the hippocampus and proinflammatory cytokines in the cerebral cortex and plasma in experimental models. We used an unbiased, label-free proteomics approach to identify the altered proteins in the hippocampus, and multiplex assay for cytokines in the cerebral cortex and plasma of C57BL/6J mice following bilateral surgical implantation of electrodes into the cerebral hemispheres. Seven days following surgery, a repeated low dose kainate (KA) regimen was followed to induce *status epilepticus (SE)*. Surgical implantation of electrodes reduced the amount of KA necessary to induce SE by 50%, compared with mice without surgery. Tissues were harvested 7 days post-SE (i.e., 14 days post-surgery) and compared with vehicle-treated mice. Proteomic profiling showed more proteins (103, 6.8% of all proteins identified) with significantly changed expression (*p* < 0.01) driven by surgery than by KA treatment itself without surgery (27, 1.8% of all proteins identified). Further, electrode implantation approximately doubled the number of KA-induced changes in protein expression (55, 3.6% of all identified proteins). Further analysis revealed that intracerebral electrodes and KA altered the expression of proteins associated with epileptogenesis such as inflammation (C1q system), neurodegeneration (cystatin-C, galectin-1, cathepsin B, heat-shock protein 25), blood–brain barrier dysfunction (fibrinogen-α, serum albumin, α2 macroglobulin), and gliosis (vimentin, GFAP, filamin-A). The multiplex assay revealed a significant increase in key cytokines such as TNFα, IL-1β, IL-4, IL-5, IL-6, IL-10, IL12p70, IFN-γ, and KC/GRO in the cerebral cortex and some in the plasma in the surgery group. Overall, these findings demonstrate that surgical implantation of depth electrodes alters some of the molecules that may have a role in epileptogenesis in experimental models.

## Introduction

In some animal models of epilepsy, cranial surgery (drilling burr holes through the skull) is required to implant electrodes for acquiring electroencephalography (EEG) recordings (1–4) and electrical kindling (5, 6) or to implant a cannula for intracerebral administration of drugs (7–9). The incidence of post-craniotomy seizures in humans has been estimated to be 15–20% (10). To our knowledge, no post-operative spontaneous seizures have been reported in rodent models. A study in the rat model, however, revealed abnormal EEG patterns (11), and several other studies in rodent models reported a reduced seizure threshold to chemoconvulsants following cranial surgery (12–15). Implantation of electrodes for kindling causes focal neuronal damage, resembling a penetrating brain injury in humans (12, 13, 16, 17), and it is unclear the extent to which the injury itself contributes to the kindling process (14). Recently, we demonstrated that even epidurally placed electrodes reduce seizure threshold induced by systemic administration of kainate (KA) (15).

KA is widely used as a chemoconvulsant in experimental rodent models for the study of the cellular and molecular mechanisms involved in epileptogenesis (18, 19). Continuous EEG is employed for the detection of the onset and progression of chronic epilepsy, via monitoring of the frequency, duration, and semiology of spontaneous recurrent seizures (SRS) and tracking of other epilepsy-associated electrographic signatures such as interictal spikes (2, 3, 20–26). Acquisition of EEG from experimental models requires the implantation of electrodes on either the *dura mater* or into the brain. It is presumed that the mechanism of epileptogenesis following chemoconvulsant-induced *status epilepticus* (SE) in experimental animals is independent of electrode implantation, but the mechanisms are not completely known. Understanding the differential expression of proteins due to surgical implantation of electrodes alone and subsequent exposure to chemoconvulsant may provide some insight into the mechanism of epileptogenesis. Three proteomics studies have been reported from rodent epilepsy models so far: a C57BL/6J mouse model with intrahippocampal KA approach but without electrodes (27), a rat pilocarpine model without electrodes (28), and an amygdala electrical kindling in female rats (29). There are currently no reports, however, on demonstrating the impact of surgically implanted intracerebral electrodes on protein expression in the hippocampus or cerebral cortical and plasma cytokines levels. Surgical procedure reduce seizure threshold, thereby requiring less KA to induce severe SE (15). This is likely due to associated inflammation in the brain; however, the mechanistic pathways are still largely unknown. To address this, we conducted a proteomics study on the hippocampus and proinflammatory cytokine assay of both cortical tissue and plasma in C57BL/6J mice with and without the implantation of intracerebral electrodes and with and without KA exposure. Proteins that alter due to surgical procedures and KA may facilitate the development of epilepsy and may reveal potential therapeutic targets for disease modification in epileptogenesis.

## Materials and Methods

### Experimental Animals

**Figure 1:**
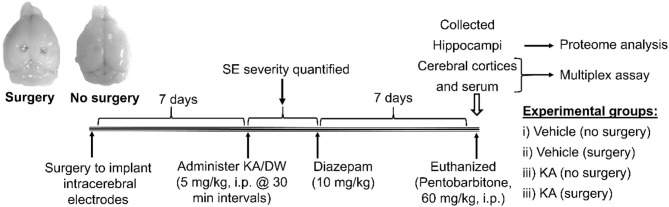
The experimental design. The brain image on the left illustrates the gross impact of the intracerebral electrodes (surgery), and the image on the right is from a mouse with no surgery.

All experimental procedures in animals were reviewed and approved by the University of Liverpool Ethics Committee as per the Animal (Scientific Procedures) Act, 1986 (UK). All experiments were conducted at the University of Liverpool. Adult male C57BL/6J mice (25–30 g; 8–9 weeks old) were purchased from Charles River, Margate, UK, and habituated for at least 4 days prior to the procedures. All animals had unlimited access to food and water. To determine the effects of intracerebral electrodes on seizure threshold for KA-induced SE, we used 15 mice that had undergone surgery and compared with a large cohort of mice (*n* = 187) without surgery. For the proteomics and Meso Scale Discovery (MSD) multiplex studies, we used a total of eight KA-treated mice (four mice per group; surgery and without surgery). These eight mice were chosen based on similar SE severity i.e., >45 min of convulsive seizures during the 2 h SE period from the first stage 5 seizure to diazepam treatment, and the KA dose of 20–25 mg/kg (4/5 doses of 5 mg/kg). All other mice were euthanized at various time points post-SE, and the tissues were archived for the other studies (not reported here). An additional eight mice (four per group) of the same age from each vehicle-treated (with and without surgery) served as controls for surgery and KA groups. All animals were euthanized at the end of the experiment with pentobarbitone (60 mg/kg, i.p.). The experimental design and experimental groups are illustrated in Figure 1. The experiments were designed and reported as per the principles of the ARRIVE guidelines (30).

### Surgical Procedure for Intracerebral Electrode Implantation

Eight mice were anesthetized with 3% isoflurane in 2 L/min oxygen until the loss of pinch reflex and maintained at 2% isoflurane during the surgery. The head was shaved and disinfected with Videne. Before surgery, animals were administered with a prophylactic antibiotic, Baytril® (Bayer Health group, Germany, 5 mg/kg, s.c.), and an analgesic, buprenorphine (Vetergesic®, Reckitt Benckiser Healthcare, UK, 0.3 mg/kg, i.m.). An incision was made through the skin, muscle, and connective tissue over the skull, as described previously (31). Bilateral burr holes of 1.8 mm diameter were made through the skull corresponding to the cerebral hemispheres and underlying hippocampi (5 mm cranial to lambda and 2 mm lateral to midline over each hemisphere). We used sterile DSI electrode wires (Data Scientific International, USA). The electrodes used were twisted (coiled bifilar) and bipolar, i.e., they measure the voltage between the two leads between the electrode coil in the (+) red insulated lead and the electrode coil in the negative (−) clear insulated lead. Each of the two electrode leads consists of a high-performance nickel cobalt alloy as used in medical implants. The electrode is a coil consisting of two wires (bifilar), but the two wires are electrically common to one another. The diameter of the coil was 0.356 mm. The coil insulation was a medical grade silicone with an outer diameter of 0.94 mm. Following removal of the electrodes' insulation, a V-shaped bend was made in the wire with an extended flat terminal end (~2 mm). The length/depth of the V-shaped electrode was 2.8 mm, which was enough to penetrate the cortex. The width of the V bend varied from bottom to the top (0.5–2 mm), which was sufficient to pass through the 1.8 mm burr hole. The V-shaped electrode was manually inserted through the burr hole. The flat free terminal end and an insulated wire at the other end of the hole were touching the skull. Electrodes were secured to the skull with dental cement constituted from Alphacryl Rapid Repair and methyl acrylate (National Dental Supplies, Southport, U.K). The incision was sutured using Polysorb 4.0 and reinforced with Vetbond™ Tissue adhesive (3M Animal Care Products, USA). All animals were given subcutaneous injections of saline for rehydration and soft food to facilitate recovery of their body weight, post-surgery.

### Repeated Low Doses of KA Administration to Induce SE

KA (Abcam Biochemicals®, Cambridge, UK) was dissolved in sterile distilled water (DW) and administered at 5 mg/kg per dose or an equal volume of DW as the vehicle. Sixteen mice were divided into four groups of four mice each, which were either surgically implanted with intracerebral electrodes or not, and either treated with KA or vehicle (DW), i.p., in a two-by-two trial design [the four groups are vehicle (no surgery), vehicle (surgery), KA (no surgery), and KA (surgery)]. KA-treated animals were scored for seizure severity using a modified Racine scale (32). The stages include stage 1, freezing; stage 2, head nodding; stage 3, rearing; stage 4, rearing and falling; and stage 5, generalized seizures. Repeated doses of KA were given at 30 min intervals until animals reach stage 5 seizures, after which the dosing was discontinued as described previously (32). Each cohort included 8–10 mice since using more animals in a cohort would be difficult to manually quantify initial SE severity, although two experimenters scored SE at the same time. KA-surgery and KA no surgery groups were treated with KA simultaneously. Vehicle-treated animals were matched to KA-treated mice by number of injections. Two hours after the onset of stage 5 seizures, all mice that received KA and their vehicle counterparts were given diazepam (10 mg/kg, i.m., Hameln Pharmaceuticals Ltd, Gloucester, UK) to standardize the duration of behavioral seizures in KA treated animals. The objective of the study was to determine the impact of surgically implanted intracerebral electrodes in the brain on protein and cytokine expression; therefore, EEG was not acquired (dummy telemetry devices were used) during the 7 day post-SE period. Videos, however, were acquired to confirm that the KA-treated mice had SRS during the following 7 days.

### Tissue Collection, Sample Preparation, and Protein Estimation

Seven days following the induction of SE with KA, all mice, including the vehicle-treated, were euthanized. Blood samples were collected by cardiac puncture into lithium heparin-coated tubes, centrifuged at 2,000 × *g* for 20 min at 4°C for the isolation of plasma, and stored at −80°C. Brains were removed, and the whole hippocampi and cerebral cortices including the tissue surrounding the electrodes were dissected, collected in cryovials, and snap-frozen in liquid nitrogen. Brain and plasma samples were stored at −80°C until required.

Hippocampi and cortical tissue samples were thawed on ice, dabbed with a sterile tissue paper to remove the water, and weighed before performing assays. Tris lysis buffer containing 1:50 protease inhibitor was added to each sample at a volume of 10 μl/mg of tissue. Samples were then homogenized using a TissueRuptor, sonicated, and centrifuged at 10,000 × *g* for 20 min at 4°C. The resulting supernatants were aliquoted, and protein concentrations were determined using a Bradford assay (Sigma Aldrich, UK). Bovine serum albumin (BSA) standards were prepared using a stock solution containing 4 g/ml BSA. BSA standards were diluted with distilled water to concentrations ranging from 100 to 1,400 μg/ml. A volume of 10 μl of each sample and BSA standards were added in duplicates to a 96-well plate. Bradford reagent (200 μl) was added to each well, and the 96-well plate was incubated at room temperature for 5 min. The maximum absorbance frequency for each sample was measured at 595 nm using a multimode plate reader (DTX 880; Beckman Coulter, USA), and the protein content of samples was determined by comparing to a standard curve generated using a serial dilution of BSA. Tissue supernatants were diluted to 1:50 with distilled water before protein assay. Plasma samples were thawed on ice, vortexed, and centrifuged at 13,000 × *g* for 10 min at 4°C. Hippocampi lysates were used for proteome analysis, and cortical lysates and plasma were used for MSD V-PLEX assay.

### Hippocampi Lysate Processing for Proteomics

The hippocampal lysates were processed for proteome analysis, as described previously (33). For digestion, 15 μl (~100 μg) of homogenate was diluted to 160 μl of ammonium bicarbonate in LoBind tubes. The proteins were denatured using 10 μl of 1% (w/v) RapiGest^TM^ SF surfactant (Waters, Manchester, UK) in 25 mM ammonium bicarbonate followed by incubation at 80°C for 10 min. Samples were reduced by adding 10 μl of 60 mM dithiothreitol and incubated at 60°C for 10 min and alkylated by adding 10 μl of 180 mM iodoacetamide and incubated at room temperature in the dark for 30 min. Trypsin (Sigma, UK) was reconstituted in 50 mM acetic acid to a concentration of 0.2 μg/μl. Digestion was performed by the addition of 10 μl of trypsin to the samples followed by incubation at 37°C overnight. Trifluoroacetic acid (TFA) was added to each sample for acidification, and the samples were incubated at 37°C for 45 min. Samples were centrifuged at 17,000 × *g* for 45 min, and the supernatant was transferred to a LoBind tube. The centrifugation step was repeated, and 10 μl of supernatant was transferred to a total recovery vial for LC-MS analysis. Pre- and post-acidification digest was analyzed by SDS-PAGE to confirm complete digestion.

### LC Separation

All peptide separations were carried out using a nanoAcquity UPLC™ system (Waters MS Technologies, Manchester, UK), as previously described (33). For each analysis, 1 μl of sample digest was loaded onto a trapping column (C_18_, 180 μm × 20 mm, Waters), using partial loop injections for 3 min at 5 μl/min with an aqueous solution containing 0.1% (v/v) TFA and 2% (v/v) acetonitrile. The sample was resolved on an analytical column (nanoAcquity UPLC™ HSS T3 column, C_18_ 150 mm × 75 μm inner diameter, 1.8 μm, Waters) using a mobile gradient phase composed of a cocktail of aqueous (A) and organic (B) solvents. Solvent A contained 0.1% (v/v) formic acid in HPLC grade water, and solvent B contained 0.1% (v/v) formic acid in HPLC grade acetonitrile. Separations were performed by applying a linear gradient of 3 to 40% solvent B over 90 min at 300 nl/min followed by a washing step (5 min at 99% solvent B) and an equilibration step (15 min at 3.8% solvent B). An equivalent to 500 ng of protein for each sample was injected.

### Mass Spectrometry

The LTQ-Orbitrap Velos instrument (Thermo Fisher) was operated in the data-dependent mode to switch between full-scan MS and MS/MS acquisition automatically. Survey full-scan MS spectra (*m/z* 3,350–2,000) were acquired in the Orbitrap with 30,000 resolution (*m/z* 400) after the accumulation of ions to 1 × 10^6^ target value based on predictive automatic gain control values from the previous full scan. The 20 most intense multiply charged ions (*z* ≥ 2) were sequentially isolated and fragmented in the linear ion trap by collision-induced dissociation with a fixed injection time of 100 ms. Dynamic exclusion was set to 20 s. Typical mass spectrometric conditions were as follows: spray voltage, 1.5 kV, no sheath and Auxillary gas flow; heated capillary temperature, 200°C; normalized CID collision energy 35%. The MS/MS ion selection threshold was set to 500 counts, and a 1.2 Da isolation width was set. A metal-coated picotip (New Objective, Presearch, UK) was used in the nanospray assembly and was maintained at a voltage of 1,500 V.

### Proteome Identification and Analysis

The hippocampi from all four groups in this study were processed simultaneously for proteomics using the same protocol and the instrument that was used in our previous study (33). We used the vehicle and KA (with no surgery) group's raw data from our recently published study (33) to compare with surgery groups. All four raw data sets were reanalyzed with Proteome Discoverer 2.2.0.388. The data were searched using Mascot 2.2.07 against Uniprot-Mus musculus with quantification using the Minora feature detector. Peptide validation was performed using the Percolator node within Proteome Discoverer. The searches were performed with static modifications of carbamidomethyl (Cys), dynamic modifications of oxidation (Met), and deamidation (Asn, Gun).

Further analysis was performed using the R package MethaboanalystR 2.0 (34), which contains R functions and libraries in the MetaboAnalyst webserver (35). Upon checking the data integrity as satisfactory (i.e., no peptide with more than 50% missing replicates, and positive values for the area), missing value estimation was imputed using the Singular Value Decomposition (SVD) method. Non-informative values that were near-constant throughout the experimental conditions were detected using the interquartile range (IQR) estimation method and deleted. Data were normalized using the Quantile normalization method. Data transformation was performed based on Generalized Logarithm Transformation (glog) to make individual features more comparable. The group samples were compared by *t*-test for paired groups with the adjusted *p*-value and False Discovery Rate (FDR) set at 0.01. Fold change analysis with a threshold of 2 was performed to compare the absolute value of change between group values (for paired groups). A volcano plot was created to combine the fold change and the two-sample *t*-test analysis. The PCA analysis was performed using the prompt package, and pairwise score plots were created to provide an overview of the various separation patterns among the most significant components. Partial least squared (PLS) regression was then performed using the plsr function provided by the R pls package to predict the continuous and discrete variables. A PLS-DA model was built to classify and cross-validated PLS using the caret package. The uniport protein ids that were altered with the *p* < 0.01 were used to retrieve the corresponding KEGG ids using the “Retrieve/ID mapping” tool of UniProt (accessible at http://www.uniprot.org/uploadlists/). KEGG ids were then used to retrieve the biological pathway association of the proteins. Enrichment analysis was performed using the Database for Annotation, Visualization, and Integrated Discovery (DAVID) 6.8 Tools (36, 37). The one-way analysis of variance (ANOVA) was used to determine whether there were any significant differences between the means ±SEM within four groups. *Post-hoc* analysis was performed with Fisher's Least Significant Difference method. Proteins with FDR values <0.01 were considered significant. Box plots were created for all significantly altered proteins determined by ANOVA (Supplementary Figure 1).

### Meso Scale Discovery Assay

#### Reagents Preparation

The standards, antibody detection solution, and read buffer were prepared in accordance with the manufacturer's instructions (MSD Kit # K15048D-1). All reagents were warmed to room temperature before preparation. The multi-analyte lyophilized calibrator, supplied by MSD, contains the highest concentration of all the cytokines and served as the standard stock for the assay. The calibrator was reconstituted in 1,000 μl of Diluent 41 (MSD, USA), mixed by vortexing, and left for 5 min before serial dilution. A series of standards were prepared by serial dilution of the calibrator solution by adding 100 μl of the calibrator to 300 μl of Diluent 41 and vortex-mixed, and the process was repeated five times to generate a total of seven standards. Diluent 41 alone was used as the blank. The kit provided 10 separate detection antibodies, 60 μl of each at 50X stock solution. All detection antibody solutions were combined together (600 μl) and added to 2,400 μl of Diluent 45 (MSD, USA) to achieve 1:50 dilution. Reader buffer T 4X stock solution (MSD, USA) was diluted to 1:2 with distilled water.

#### Linearity of Dilution for the MSD V-PLEX Kit

The linearity of dilution was first performed on 16 wells of the customized 96-well plate using two cerebral cortices supernatant samples. One sample was from the vehicle (no surgery) group, while the other sample was from KA-treated (surgery). These samples were predicted to have the least and the highest amount of inflammatory changes, respectively, due to the extent of insult to the brain. The cortical supernatants or plasma samples were serially diluted to 1:2, 1:4, 1:8, and 1:16 using Diluent 41 (MSD). The observed values were assessed relative to the standard curve for all 10 inflammatory cytokines. The results were calculated based on the standard curve, and the observed concentration was multiplied by the dilution factor. The criteria for acceptable dilutional linearity were for the corrected observed concentrations to vary no more than 80% to 120% of the theoretical concentration between each serial dilution for each analyte (38).

#### MSD V-PLEX Assay Protocol

The assay was performed in accordance with the manufacturer's instructions (MSD kit reference K15048D-1). Plasma samples were diluted 1:2 using Diluent 41. The standards, blank, and samples (cortical lysates or plasma) solution were measured in duplicates, with 50 μl of each solution added to an allocated well within the customized 96-well plate. The plate was sealed and incubated at room temperature on a shaker for 2 h. After incubation, the plate was washed three times with wash buffer, and 25 μl of the diluted detection antibody was added to each well, the plate resealed, and incubated at room temperature on a shaker for a further 2 h. After antibody incubation, the plate was washed three times, as previously described, and then 150 μl of 2X read buffer T was added to each well. The plate was then placed on the MSD instrument and read immediately. The reading of the V-Plex plate was performed using the MSD SECTOR Imager 2400, according to the manufacturer's manual (MSD, USA). The plate had an MSD barcode that allowed the SECTOR Imager to detect the type of plate being run. The data generated was automatically analyzed with a template using the Discovery Workbench version 4.0 software. The cytokines levels were expressed in pg/ml for plasma and pg/mg of protein detected in a 100 mg/ml tissue lysate, determined using the Bradford protein assay. One-way ANOVA with Tukey's *post hoc* analysis was performed using the SPSS software.

## Results

### Intracerebral Electrodes Reduced Seizure Threshold for KA-Induced SE at Day 7 Post-surgery

**Figure 2:**
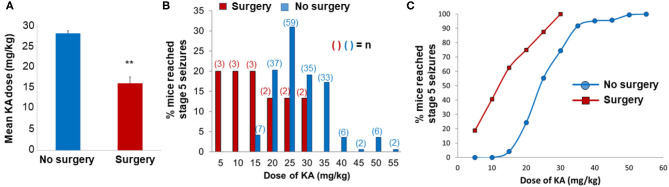
Impact of surgery on seizure threshold in response to kainate (KA). **(A,B)** The mean **(A)** and the total **(B)** KA dose required to induce generalized convulsive seizures (stage 5 on the Racine scale) in C57BL/6J mice that were either implanted with intracerebral electrodes (Surgery, *n* = 15) or not implanted (no surgery, *n* = 187). KA was administered 7 days post-surgery. In graph A, the data are expressed as the mean (±SEM) KA dose (mg/kg) and compared the groups using a two-sample *t*-test (**p < 0.01). The data in B are expressed as a percentage of the total number of animals in each group that reached stage 5 seizures at each dose increment. **(C)** The dose-response data for KA are expressed as a cumulative percentage of the total number of animals in each group.

The rationale for choosing the 7 day time point post-SE, in contrast to the early or later time points, was based on our previous work that demonstrated a significant increase in gliosis, neurodegeneration, and neurogenesis in C57BL/6J mouse KA model at 7 day post-SE (26). KA-induced seizure susceptibility of mice with surgically implanted intracerebral electrodes was compared with no surgery (naïve) animals. We assessed the total amount of KA required to induce stage 5 seizures in both surgery and non-surgery groups at day 7 post-surgery. Electrodes implanted mice required significantly less KA (15.78 ± 1.47 mg/kg; *n* = 15) than non-implanted mice (27.98 ± 0.6 mg/kg; *n* = 187) to induce generalized convulsive seizures (*p* < 0.01; Figures 2A,B). We had to use a large cohort of naïve mice to cover a wide range of KA doses to achieve stage 5 seizures. There was a left shift in the KA dose-response curve in the surgery group when compared with the group without surgery (Figure 2C). The range of total KA doses for post-surgery mice was 5–30 mg/kg, and for mice without surgery, the range was 15–55 mg/kg (Figure 2B). After a single dose of 5 mg/kg KA, 18.75% of mice in the post-surgery group experienced generalized seizures (stage 5), whereas no mice that had not previously undergone surgical implantation of electrodes had convulsive seizures (Figure 2C). At a total dose of 30 mg/kg KA, all mice that had undergone surgery experienced generalized seizures, compared with 74.4% of naïve mice (Figure 2C). Of these, four animals from each group that had similar SE severity (continuous generalized seizures lasting for >45 min) and KA doses (four to five doses of 5 mg/kg) were used for proteomics and cytokine assays. The remaining animals were euthanized at various time-points post-SE and used for other analyses, and not reported here. Video analysis of all eight KA-treated mice used in this proteomics study had a minimum of one SRS during the 7 days post-SE.

After the mice were euthanized, the extent of gross damage (similar in all animals) was confirmed, while the hippocampus was separated from the cerebral cortex under a dissection microscope. Also, clear electrode marks on the cerebral cortices were visible in all animals (an example is shown in Figure 1). The hippocampi were used for proteomics and the cortical tissue and serum for cytokine assays.

### Overall Changes in Proteins in the Hippocampus in Response to Surgery and KA at 14 Days Post-surgery (i.e., 7 Days Post-SE)

The number of proteins identified from the raw data in which expression was significantly altered (*p* < 0.01) between vehicle and KA-treated mice with or without surgery, as searched for against the UniProt mouse database, is listed in Table 1. Data presented as the absolute number of proteins with significant changes (downregulated, upregulated, and total significant) as a percentage of the total number of proteins identified in the study (Table 1). The surgery increased higher number of proteins regardless of whether they were subsequently treated with KA or vehicle (A, B in Table 1). The impact of surgery alone on the percentage of protein expression was greater than that of KA-induced seizures in mice that had no surgery (103 vs. 27 total significant; 6.8 vs. 1.8%). KA had a greater effect in the surgery group (C, 55 proteins altered; 3.8% of total significant) than in no surgery group (D, 27 proteins altered; 1.8% of total significant) (Table 1). We also investigated whether any of the endogenous resolving proteins were altered in response to surgery and/or KA induced brain trauma. We detected leukotriene A-4 hydrolase (FDR, 0.92; *p* = 0.89, but interestingly, not the leukotriene B4, a chemoattractant at an early phase of insult) and prostaglandin E synthase (FDR, 0.46, *p* = 0.26), but there were no significant differences. Also, our search did not yield any lipoxins or resolvins.

**Table 1:** Comparison of the changes in the expression of proteins in the hippocampus between treatment groups are reported as absolute numbers and percentage of total identified proteins.

**Treatment groups**	**Downregulated**	**Upregulated**	**Total significant**	**% significant of all proteins identified**
(A) Vehicle: Surgery vs. no surgery	59	44	103	6.80%
(B) KA: Surgery vs. no surgery	47	35	82	5.40%
(C) Surgery: KA vs. vehicle	32	23	55	3.60%
(D) No surgery: KA vs. vehicle	16	11	27	1.80%

*Treatment groups (n = 4) comprised animals that had undergone surgery for the implantation of intracerebral electrodes or no surgery and that subsequently received KA to induce seizures or vehicle as a control. Data reports proteins with significantly altered between groups (t-test, p < 0.01), either as absolute numbers or as percent significant of all identified proteins*.

#### Differential Expression of Proteins in the Hippocampus in Response to Surgery Alone or KA (With or Without Surgery) at 14 Days Post-surgery (i.e., 7 Days Post-KA)

One-way ANOVA with Fisher's *post-hoc* analysis of all four groups revealed a significant increase in the proteins that have role in neuroinflammation including gliosis [C1q system, glial fibrillary acidic protein (GFAP), and vimentin] and neurodegeneration (cystatin-C and galectin-1) in both surgery groups treated with KA or vehicle, and in KA-treated group without surgery. The Ras and Rab related proteins (Rab6a and Rras2) and calcium/calmodulin-dependent protein kinase IIa (CaMKIIα that have a role in neuronal plasticity were significantly reduced in all three groups (Table 2, Supplementary Figure 1). In KA groups, with or without surgery, glypican-1, heat shock protein beta-1 (HSP25), unconventional myosin-Va, neurosecretory protein VGF (VGF-derived peptide TLQP-62), and EF-hand domain-containing protein D2 (Swiprosin-1) were significantly upregulated, while the voltage dependent GABA A transporter (GAT-1) and glutamine synthase (GS) were downregulated (Table 2, Supplementary Figure 1). In addition to the above listed proteins, the proteins that were up- or downregulated in surgery groups (with or without KA) are listed in Table 2 and box plots for each protein are illustrated in Supplementary Figure 1.

**Table 2:** The impact of intracerebral electrode implants (surgery) and KA or vehicle on altered protein expression in the hippocampus.

**Uniprot ID**	**Protein names**	**Gene names**	***f*-value**	***p*-value**	**Neg log_**10**_(p)**	**FDR**	**Abundance on a log scale**			
							**Veh-NoSurg**	**Veh-Surg**	**KA-NoSurg**	**KA-Surgery**
**Proteins upregulated in surgery vehicle and both KA groups**										
P03995	Glial fibrillary acidic protein (GFAP)	Gfap	31.792	5.43E-06	5.265	0.00138	25.55244	27.025413	26.87475	27.968345
P20152	Vimentin	Vim	20.7	4.91E-05	4.3091	0.00361	23.70164	25.16986	24.87103	26.487395
P98086	Complement C1qa	C1qa	15.099	0.000224	3.6494	0.00901	17.52368	19.70244	18.78533	19.755233
P14106	Complement C1qb su	C1qb	25.31	1.78E-05	4.7495	0.00191	19.04926	20.753115	19.97015	21.130523
Q02105	Complement C1q subunit C	C1qc C1qg	16.908	0.000131	3.8812	0.00633	18.60421	20.583218	19.43826	20.605043
P16045	Galectin-1 (Gal-1/Galaptin)	Lgals1 Gbp	23.294	2.72E-05	4.566	0.00244	18.55418	19.299368	19.29811	20.263458
P21460	Cystatin-C (Cystatin-3)	Cst3	15.395	0.000205	3.6888	0.00869	21.29772	21.565828	21.74042	22.158848
P62301	40S ribosomal protein S13	Rps13	20.655	4.96E-05	4.3044	0.00361	21.70742	21.852868	21.85315	21.976885
**Proteins downregulated in surgery vehicle and both KA groups**										
P35279	Ras-related protein Rab-6A (Rab-6)	Rab6a	18.567	8.37E-05	4.077	0.00553	21.63157	21.371815	21.51557	21.370305
P62071	Ras-related protein R-Ras2	Rras2	17.853	0.000101	3.9946	0.00576	20.50515	20.053165	20.35101	19.987185
P11798	Calcium/calmodulin-dependent protein kinase IIa (CaMK-IIa)	Camk2a	24.743	0.00002	4.6992	0.00191	28.23778	28.094555	28.03671	27.681833
**Proteins upregulated in both KA groups (with or without surgery)**										
Q9QZF2	Glypican-1 [Cleaved into: Secreted glypican-1]	Gpc1	33.445	4.15E-06	5.3816	0.00138	19.79506	19.762235	20.0398	20.213773
P14602	Heat shock protein beta-1 (HspB1/HSP 25)	Hspb1 Hsp25/27	26.34	1.45E-05	4.8385	0.00191	18.34595	18.024863	20.10053	21.608808
Q99104	Unconventional myosin-Va	Myo5a Dilute	17.692	0.000106	3.9756	0.00576	23.74006	23.75106	23.87258	23.905998
Q0VGU4	Neurosecretory protein VGF (VGF-derived peptide TLQP-62)	Vgf	17.569	0.000109	3.961	0.00576	20.14906	19.824463	20.7232	21.123153
Q9D8Y0	EF-hand domain-containing protein D2 (Swiprosin-1)	Efhd2 Sws1	17.443	0.000113	3.946	0.00576	23.16364	23.261423	23.45649	23.668985
**Proteins downregulated in both KA groups (with or without surgery)**										
P31648	Sodium- and chloride-dependent GABA transporter 1 (GAT-1)	Slc6a1 Gabt1 Gat-1	17.805	0.000103	3.9889	0.00576	23.44462	23.413773	23.06983	22.927308
P15105	Glutamine synthetase (GS)	Glul Glns	14.75	0.00025	3.6022	0.00969	26.8151	26.797415	26.51625	26.618135
**Protein upregulated in both surgery groups (vehicle and KA)**										
Q8VHL1	Histone-lysine N-methyltransferase SETD7 (SET7/9)	Setd7 Set7 Set9	66.863	9.25E-08	7.0337	0.00014	21.23233	21.911073	21.15768	21.993103
Q9D0M5	Dynein light chain 2, cytoplasmic (DLC8)	Dynll2 Dlc2	58.2	2.02E-07	6.6954	0.00015	22.21056	21.12811	22.18282	21.00142
Q6A026	Sister chromatid cohesion protein PDS5 A	Pds5a Kiaa0648	36.452	2.62E-06	5.5812	0.00134	19.8724	20.885968	19.80909	20.84635
P03995	Glial fibrillary acidic protein (GFAP)	Gfap	31.792	5.43E-06	5.265	0.00138	25.55244	27.025413	26.87475	27.968345
Q8CIG9	F-box/LRR-repeat protein 8 (F-box protein FBL8)	Fbxl8 Fbl8	32.684	4.69E-06	5.3286	0.00138	19.18971	20.350538	18.93956	20.272563
Q6PB66	Leucine-rich PPR motif-containing protein, mitochondrial (LRP 130)	Lrpprc Lrp130	29.347	8.27E-06	5.0823	0.0018	21.12106	21.556105	21.02334	21.506653
Q3URK3	Methylcytosine dioxygenase TET1 (CXXC-type zinc finger protein 6)	Tet1 Cxxc6 Kiaa1676	27.562	1.15E-05	4.9404	0.00181	19.20219	20.01175	18.97767	19.870405
P55012	Solute carrier family 12 member 2 (Basolateral Na-K-Cl symporter)	Slc12a2 Nkcc1	27.452	1.17E-05	4.9313	0.00181	21.57142	22.651055	21.59296	22.838318
P10605	Cathepsin B/B1)	Ctsb	15.512	0.000198	3.7042	0.00868	21.24294	21.535165	21.29265	21.835295
P14602	Heat shock protein beta-1 (HspB1/ HSP 25)	Hspb1 Hsp25/27	26.34	1.45E-05	4.8385	0.00191	18.34595	18.024863	20.10053	21.608808
Q5SSL4	Active breakpoint cluster region-related protein	Abr	24.9	1.94E-05	4.7132	0.00191	21.39741	22.032938	21.47865	22.021938
P16045	Galectin-1 (Gal-1/Galaptin)	Lgals1 Gbp	23.294	2.72E-05	4.566	0.00244	18.55418	19.299368	19.29811	20.263458
Q9WVA3	Mitotic checkpoint protein BUB3	Bub3	22.904	2.96E-05	4.5289	0.00251	19.26223	19.585013	19.20765	19.39816
Q02105	Complement C1qc	C1qc C1qg	16.908	0.000131	3.8812	0.00633	18.60421	20.583218	19.43826	20.605043
P20152	Vimentin	Vim	20.7	4.91E-05	4.3091	0.00361	23.70164	25.16986	24.87103	26.487395
P62301	40S ribosomal protein S13	Rps13	20.655	4.96E-05	4.3044	0.00361	21.70742	21.852868	21.85315	21.976885
Q9CQJ6	Density-regulated protein (DRP)	Denr	19.443	6.69E-05	4.1749	0.00464	19.67067	20.136073	19.63894	20.130675
P02468	Laminin subunit gamma-1 (Laminin B2 chain)	Lamc1 Lamb-2	18.078	9.53E-05	4.0209	0.00576	18.75928	19.305835	18.70097	19.563578
P21460	Cystatin-C (Cystatin-3)	Cst3	15.395	0.000205	3.6888	0.00869	21.29772	21.565828	21.74042	22.158848
P98086	Complement C1qa	C1qa	15.099	0.000224	3.6494	0.00901	17.52368	19.70244	18.78533	19.755233
**Proteins downregulated in both surgery groups (vehicle and KA)**										
P32037	Solute carrier family 2-(Glucose transporter type 3, brain) (GLUT-3)	Slc2a3 Glut3	24.736	0.00002	4.6986	0.00191	22.52552	22.390868	22.63737	22.245415
Q62283	Tetraspanin-7 (Tspan-7/CD antigen CD231)	Tspan7 Mxs1	21.247	4.31E-05	4.3654	0.00347	19.89033	19.44267	19.63764	19.222073
Q8C0E2	Vacuolar protein sorting-associated protein 26B	Vps26b	27.386	1.19E-05	4.926	0.00181	21.77001	21.464778	21.70237	21.46987
Q8CBW3	Abl interactor 1 (Abelson interactor 1) (Abi-1)	Abi1 Ssh3bp1	18.428	8.69E-05	4.0612	0.00553	21.26101	20.738385	21.16048	20.689868
P35279	Ras-related protein Rab-6A (Rab-6)	Rab6a Rab6	18.567	8.37E-05	4.077	0.00553	21.63157	21.371815	21.51557	21.370305
P62071	Ras-related protein R-Ras2	Rras2	17.853	0.000101	3.9946	0.00576	20.50515	20.053165	20.35101	19.987185
P31648	Sodium- and chloride-dependent GABA transporter 1 (GAT-1)	Slc6a1 Gat-1	17.805	0.000103	3.9889	0.00576	23.44462	23.413773	23.06983	22.927308
Q9D8Y0	EF-hand domain-containing protein D2 (Swiprosin-1)	Efhd2 Sws1	17.443	0.000113	3.946	0.00576	23.16364	23.261423	23.45649	23.668985
P39053	Dynamin-1 (EC 3.6.5.5)	Dnm1 Dnm	16.874	0.000133	3.8771	0.00633	27.24975	27.13156	27.22933	26.941998
Q9QYB8	Beta-adducin (Add97)	Add2	15.586	0.000193	3.7139	0.00868	23.79847	23.675545	23.77589	23.580043
Q62188	Dihydropyrimidinase-related protein 3 (DRP-3)	Dpysl3 Drp3 Ulip	15.49	0.000199	3.7014	0.00868	24.70239	24.31222	24.70526	24.134963
P61161	Actin-related protein 2 (Actin-like protein 2)	Actr2 Arp2	15.215	0.000216	3.665	0.00893	24.06893	23.887608	24.00868	23.72985
P15105	Glutamine synthetase (GS)	Glul Glns	14.75	0.00025	3.6022	0.00969	26.8151	26.797415	26.51625	26.618135

*The numbers in the abundance column are the abundances normalized and averaged for four animals (two technical replicates/animal) in each group, and represented on a log scale. All four groups were compared using one-way ANOVA and Fisher's post-hoc test. The proteins that were significantly altered by p < 0.01 and FDR < 0.01, and relevant to epileptogenesis/epilepsy are only listed in the table. Box plots for these proteins are included in Supplementary Figure 1. All other proteins are included in the (Supplementary Table 1)*.

#### Differential Expression of Proteins in the Hippocampus in Response to Surgically Implanted Intracerebral Electrodes (Without KA)

**Figure 3:**
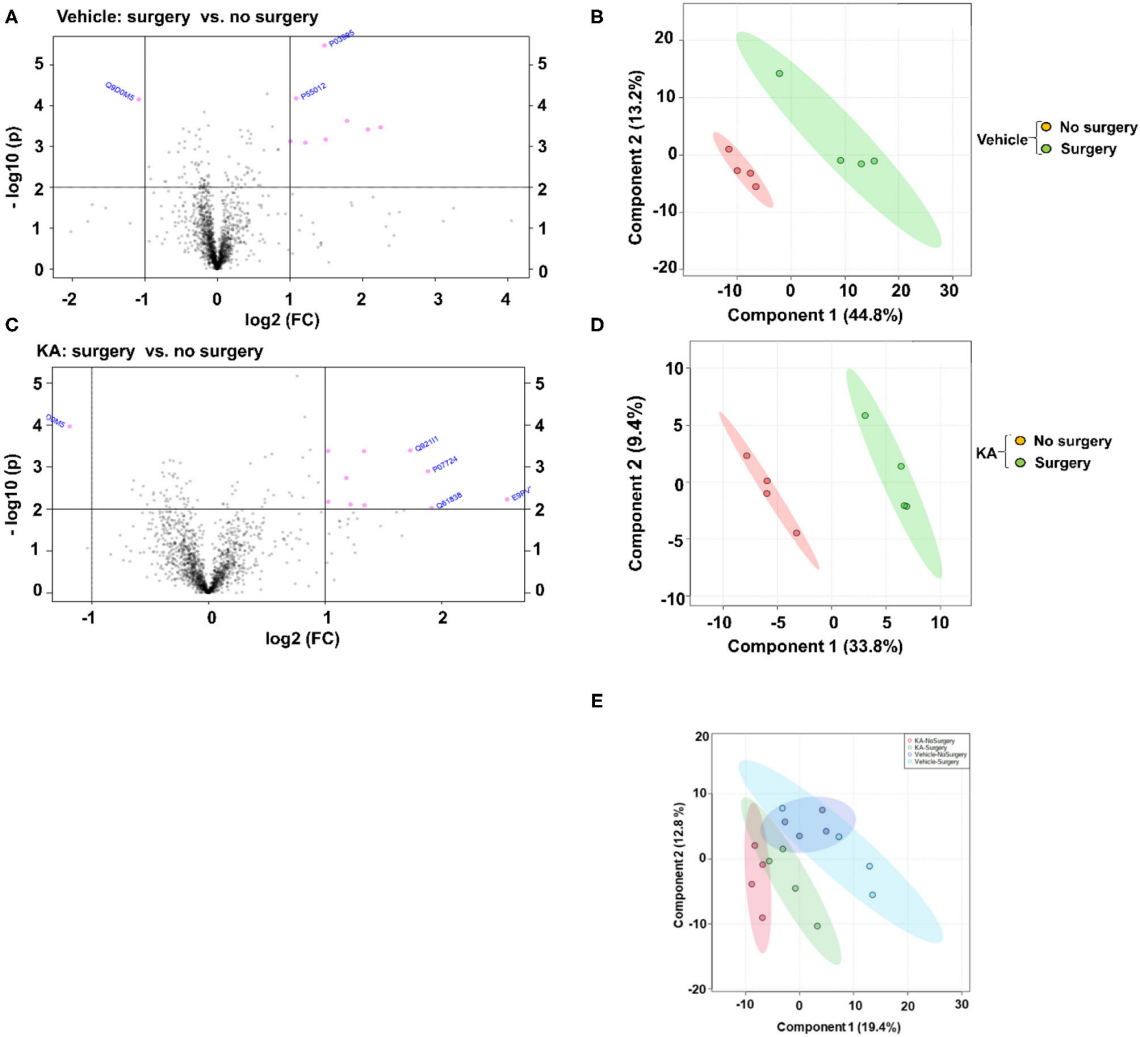
Fold change (FC) analysis of the vehicle or KA treated groups, with or without surgery, with a threshold of 2 was performed to compare the absolute change between group values. The Volcano plots **(A,C)** were created to show both the fold change and the two-sample *t*-test analysis. The PCA analysis was performed using the prccomp package, and pairwise score plots **(B,D)** and all four-way plot **(E)** represent an overview of the various separation patterns among the most significant components.

**Figure 4:**
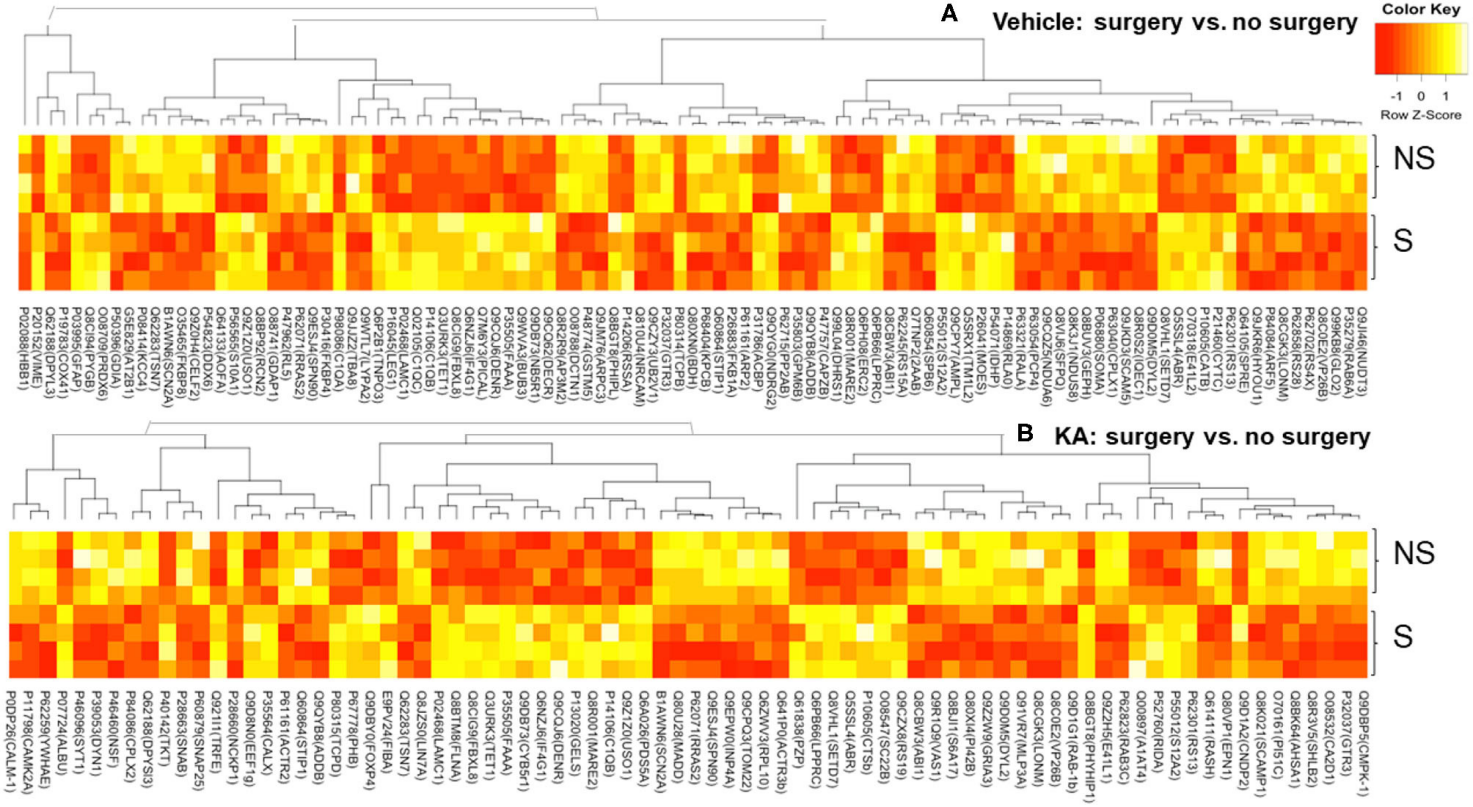
Heatmaps—an overview of the variation in proteins between surgery and no surgery groups treated with either vehicle **(A)** or KA **(B)**. NS, no surgery; S, surgery.

A large number of proteins were altered in mice with intracerebral electrodes compared with the mice without surgery and electrodes (103 proteins, *p* < 0.01; Tables 1, 3, Supplementary Table 2). An overview of the variation of all proteins between surgery and without surgery groups is illustrated in volcano plot, PCA, and heatmap (Figures 3A,B, 4A). The proteins associated with the innate immune system such as complement components C1q a, b, and c were increased by 3.34-fold (*p* = 0.0003) in the surgery group. Astroglia cytoskeletal proteins such as vimentin and GFAP were also upregulated in the surgery group (>2-fold, *p* < 0.0001). The other key proteins that were upregulated by >2-fold (*p* < 0.001) were F-box and leucine-rich repeat protein 8 (FBXL8), solute carrier family 12 member 2 (basolateral Na-K-Cl symporter), and dehydrogenase/reductase SDR family member 1 (DHRS1). The KEGG pathway enrichment analysis revealed a significant increase in proteins involved in the process of neurodegeneration, neuroinflammation, and oxidative stress (for example, prion disease pathway proteins—C1qs and Stip1; Chagas disease pathway proteins—serine/threonine-protein phosphatase 2A (PP2A) related proteins; glutathione pathway proteins such as glutathione S-transferase and peroxiredoxin-6) (Supplementary Table 4, Supplementary Figure 2). FDR for the proteins in prion disease and ribosome pathways were ≤1, while the other pathways were >9 (Supplementary Table 4).

**Table 3:** The impact of intracerebral electrode implants on altered protein expression in the hippocampus.

**Uniprot ID**	**Protein names**	**Gene names**	**KEGG-ID (mmu)**	**Fold change**	**log_**2**_(FC)**	***P*-value**	**neg log_**10**_(*p*)**
P98086	Complement C1q subcomponent subunit A	C1qa	12,259	4.7473	2.2471	0.000337	3.4723
Q02105	Complement C1q subcomponent subunit C	C1qc C1qg	12,262	4.2034	2.0715	0.000383	3.4169
P14106	Complement C1q subcomponent subunit B	C1qb	12,260	3.4434	1.7838	0.000235	3.6293
P20152	Vimentin	Vim	22,352	2.8094	1.4903	0.00067	3.174
P03995	Glial fibrillary acidic protein (GFAP)	Gfap	14,580	2.7743	1.4721	3.33E-06	5.4779
Q8CIG9	F-box and leucine-rich repeat protein 8	Fbxl8 Fbl8	50,788	2.3142	1.2105	0.000804	3.095
P55012	Solute carrier family 12 member 2 (Basolateral Na-K-Cl symporter)	Slc12a2 Nkcc1	20,496	2.1188	1.0833	6.57E-05	4.1824
Q99L04	Dehydrogenase/reductase SDR family member 1	Dhrs1 D14ertd484e	52,585	2.0032	1.0023	0.000742	3.1294

*The groups compared were between the surgery vs. no surgery treated with vehicle (distilled water). The proteins that were significantly altered by p < 0.01 and >2-fold are only listed in the table. All other proteins are included in Supplementary Table 2*.

#### Differential Expression of Proteins in the Hippocampus in Response to KA in Surgically Implanted Intracerebral Electrodes vs. KA Without Surgery at 14 Days Post-surgery (i.e., 7 Days Post-SE)

We analyzed the impact of KA-induced SE on protein levels in mice that had surgery and without surgery. The volcano plot, PCA, and heatmap represent an overview of the variation in protein expression between the treatment groups (Figures 3C–E, 4B). The proteins that were significantly increased by >2-fold (*p* < 0.01) in response to KA in surgery group when compared with no surgery group were BBB function-related proteins such as fibrinogen-α (aids as an adhesive substrate for platelets, endothelial cells, and leukocytes), pregnancy zone protein (α2 macroglobulin), serum albumin, serotransferrin (transferrin/β1 metal-binding globulin), and α1-antitrypsin 1–4 (α1 protease inhibitor 4, a serum glycoprotein) (Table 4). FBXL8 and C1qb were also increased. A cation-coupled chloride transporter (Na-K-Cl symporter/SCL12A), a core cycle regulator (sister chromatid cohesion protein PDS5 homolog A), and a cytoskeletal protein Filamin-A (FLN-A/actin-binding protein 280) were significantly increased in KA-treated mice in the surgery group (Table 4, Supplementary Table 3). The list of KEGG pathways and the proteins altered are listed in Supplementary Table 5. The most striking KEGG pathway proteins affected in the surgery group in response to KA, with <1% FDR, were the synaptic vesicle cycle pathway proteins. If we consider *p* ≤ 0.05, however, specific proteins involved in the regulation of actin cytoskeleton, prion diseases (neurodegeneration and neuroinflammation), endocytosis, phosphatidylinositol, and neurotrophin signaling pathways, Legionellosis (inflammation-related), glioma, and long-term potentiation were significantly affected in pathway enrichment analysis (Supplementary Table 5, Supplementary Figure 3).

**Table 4:** The impact of KA-induced SE in intracerebral electrode implanted animals on the expression of proteins in the hippocampus.

**Uniprot ID**	**Protein names**	**Gene names**	**KEGG-ID (mmu)**	**Fold change**	**log_**2**_(FC)**	***P*-value**	**neg log_**10**_(*p*)**
E9PV24	Fibrinogen alpha chain	Fga	14,161	5.9018	2.5612	0.005928	2.2271
Q61838	Pregnancy zone protein (Alpha-2-macroglobulin)	Pzp A2m		3.7671	1.9135	0.009382	2.0277
P07724	Serum albumin	Alb Alb-1 Alb1	11,657	3.6877	1.8827	0.001262	2.899
Q921I1	Serotransferrin (Transferrin) (Beta-1 metal-binding globulin)	Tf Trf	22,041	3.3186	1.7306	0.000404	3.3938
Q00897	Alpha-1-antitrypsin 1-4 (α-1 protease inhibitor 4)	Serpina1d Dom4 Spi1-4	20,703	2.5277	1.3378	0.008136	2.0896
Q8CIG9	F-box/LRR-repeat protein 8	Fbxl8 Fbl8	50,788	2.521	1.334	0.000421	3.3757
P14106	Complement C1q subcomponent subunit B	C1qb	12,260	2.3267	1.2183	0.007832	2.1061
P55012	Solute carrier family 12 member 2 (Na-K-Cl symporter)	Slc12a2 Nkcc1	20,496	2.2689	1.182	0.001829	2.7379
Q6A026	Sister chromatid cohesion protein PDS5 homolog A	Pds5a Kiaa0648		2.0361	1.0258	0.000415	3.3817
Q8BTM8	Filamin-A (FLN-A) (Actin-binding protein 280)	Flna Fln Fln1	192,176	2.0359	1.0257	0.006744	2.1711

*The groups compared were between surgery vs. no surgery treated with KA. The proteins that were significantly altered by p < 0.01 and >2-fold are only listed in the table. All other proteins are included in Supplementary Table 3*.

### Multiplex Assay to Determine Differential Expression of Cytokines in Cortical Tissue and Plasma

We used the MSD multiplex kit since it had cytokines/chemokines relevant to epilepsy/seizures and trauma (references are included in the summary **Table 6**). The other advantages of this kit over the other commercial multiplex kits are as follows: (i) it requires small volume of analyte (25 μl, in contrast to 50 μl), therefore suitable for mouse tissue; (ii) it is compatible for both plasma and brain lysate; and (iii) it measures both high and low abundance analytes with a 5-Log+ dynamic range.

#### Linearity of Dilution Assessment and Detection Ranges of Cytokines in 1:2 Diluted Test Samples

A serial dilution of mouse cortex supernatant was conducted to determine the optimum dilution required for the MSD assay for each cytokine. Two cortical supernatants were used: one from without surgery and KA (no surgery + vehicle) that was expected to have the lowest cytokine concentrations and one from an electrode implanted and KA-treated mouse (surgery + KA) that was anticipated to have the highest cytokine concentrations. Based on the linearity of dilution results, 1:2 was considered to be the optimum dilution for the quantification of inflammatory cytokines using the MSD kit. We also determined whether the cytokine levels in a 1:2 dilution of cortical supernatants lie within the detectable (dynamic) range of the MSD assay. Six out of 10 cytokines showed concentrations within the anticipated dynamic range for both samples (Table 5), suggesting the reliability of the assay kit.

**Table 5:** The linearity of dilution assessment and detection ranges of cytokines in 1:2 diluted test samples using the MSD assay kit.

**MSD cytokines tested**	**Expected dynamic range (pg/ml)**	**Detected conc. in 1:2 dilution (no surgery+ vehicle) (pg/ml)**	**Detected conc. In 1:2 dilution (surgery + KA) (pg/ml)**
IFN-γ	0.0471–815	321	346
IL-10	0.742–2,540	1,016	1,590
IL-12p70	7.98–22,900	429	422
IL-1	0.123–1,470	617	2,367
IL-2	0.259–2,110	441	581
IL-4	0.120–1,320	753	886
IL-5	0.0667–821	123	131
IL-6	0.830–3,490	846	3,838
KC/GRO	0.208–1,540	3,032	18,048
TNFα	0.127–507	866	1,124

*Two cortical supernatants were used: one from a non-implanted, non-KA mouse (no surgery + vehicle) that was expected to have the lowest cytokine concentrations and one from an electrode implanted and KA-treated mouse (surgery + KA) that was predicted to have the highest cytokine concentrations*.

#### Cytokine Levels in Cortical Samples

**Figure 5:**
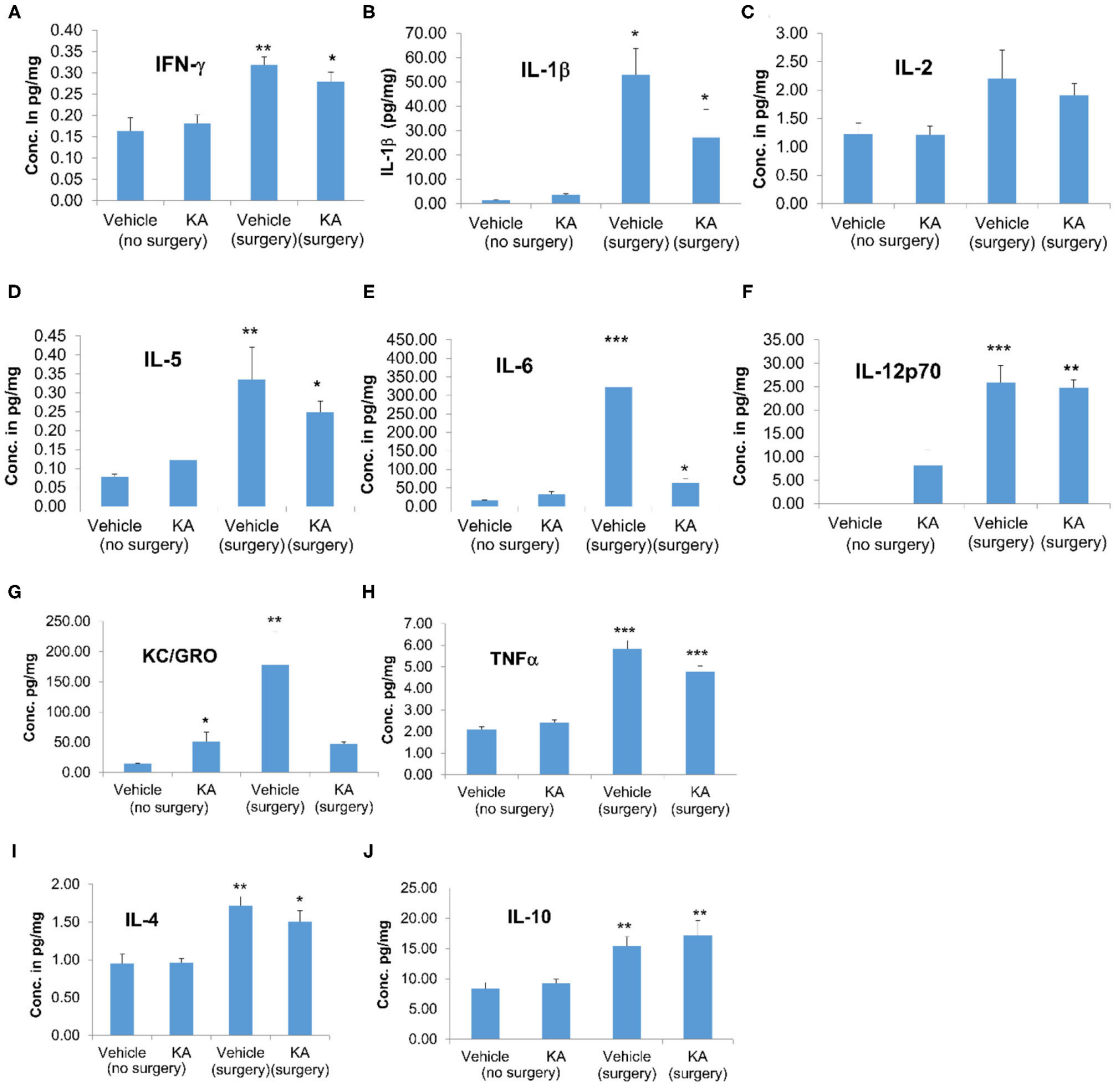
The effects of the vehicle or KA treatment in mice that had surgery for implantation of intracerebral electrodes or without surgery on inflammatory cytokines in the cerebral cortex. The following cytokines were measured and represented in the graphs: **(A)** interferon-γ (IFN-γ), **(B)** interleukin-1β (IL-1β), **(C)** interleukin-2 (IL-2), **(D)** interleukin-5 (IL-5), **(E)** interleukin-6 (IL-6), **(F)** interleukin-12p70 (IL-12p70), **(G)** keratinocyte-derived cytokine/growth-related oncogene (KC-GRO), **(H)** tumor necrosis factor-α (TNF-α), **(I)** interleukin-4 (IL-4), **(J)** interleukin-10 (IL-10). The data are expressed as the mean (± SEM) cytokine concentration in pg/mg protein. The values were compared across all four groups using one-way ANOVA with Tukey's *post-hoc* test (*n* = 4, *p < 0.05; **p < 0.01; ***p < 0.001).

We used cortical tissue to perform the cytokine assay. Surgical implantation of intracerebral electrodes caused a significant increase in the concentrations of proinflammatory cytokines IFN-γ, IL-1β, IL-5, IL-6, IL-12p70, and TNFα irrespective of the vehicle or KA treatment post-surgery (Figure 5). Likewise, interestingly, the pleiotropic anti-inflammatory cytokines IL-4 and IL-10 were also increased in the mice that had intracerebral electrodes irrespective of the vehicle or KA treatment post-surgery (Figures 5I,J). KC/GRO was the only proinflammatory cytokine that was increased in KA-treated group (naïve animals without surgery). KC/GRO, however, was also upregulated in surgery group treated with the vehicle but not with KA (Figure 5G). In summary, surgery caused a significant increase in all cytokine levels tested, except IL-2, irrespective of the vehicle or KA treatment post-surgery.

#### Plasma Cytokine Levels

**Figure 6:**
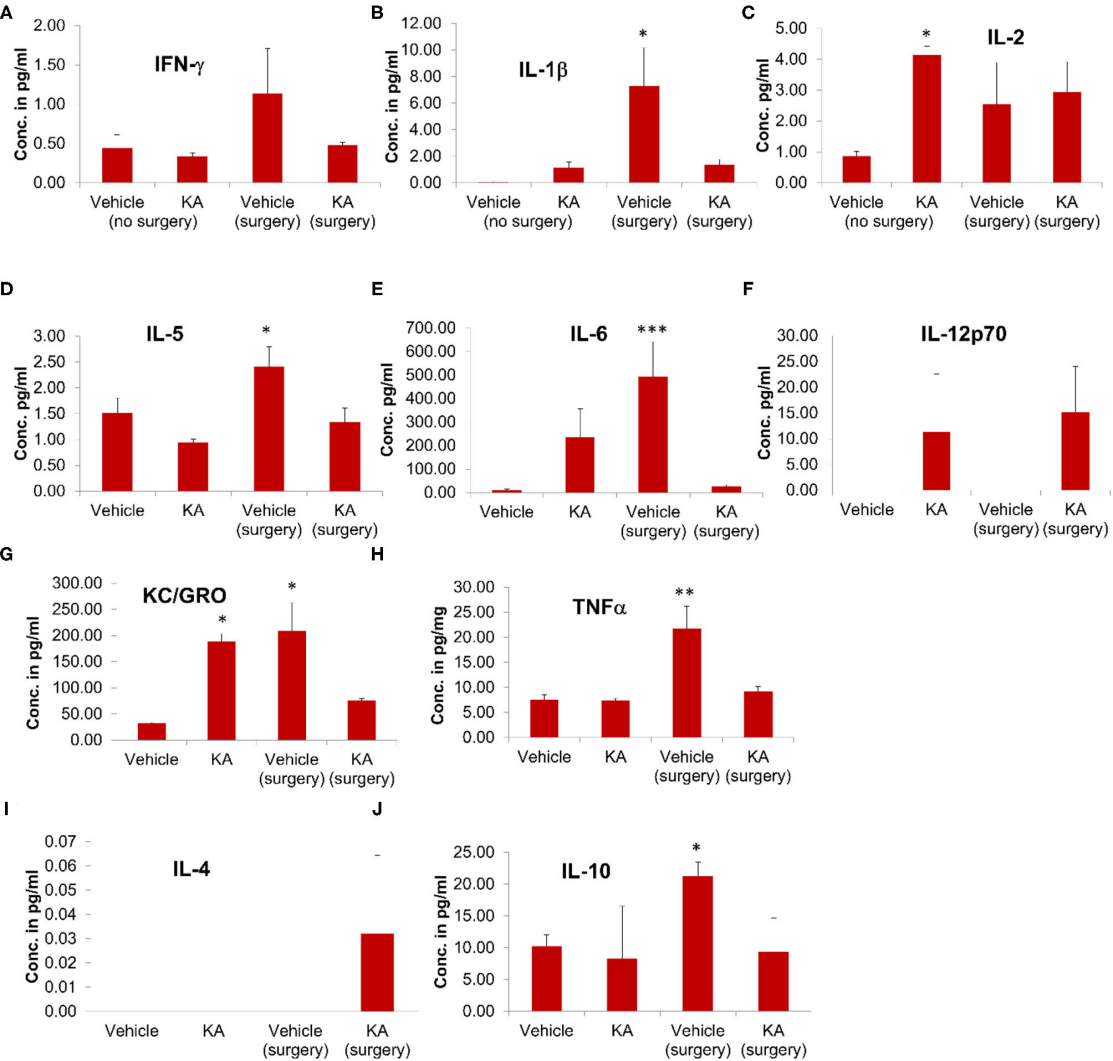
The effects of the vehicle or KA treatment in the mice that had surgical implantation of EEG electrodes or without surgery on inflammatory cytokines in the plasma from the same animals that were used for proteomics (hippocampi) and multiplex assay (cerebral cortices). The following inflammatory cytokines were measured and represented in the graphs: **(A)** interferon-γ (IFN-γ), **(B)** interleukin-1β (IL-1β), **(C)** interleukin-2 (IL-2), **(D)** interleukin-5 (IL-5), **(E)** interleukin-6 (IL-6), **(F)** interleukin-12p70 (IL-12p70), **(G)** keratinocyte-derived cytokine/growth-related oncogene (KC-GRO), **(H)** tumor necrosis factor-α (TNF-α), **(I)** interleukin-4 (IL-4), **(J)** interleukin-10 (IL-10). The data are expressed as the mean (± SEM) cytokine concentration in pg/ml of plasma. The values were compared statistically using one-way ANOVA with Tukey's *post-hoc* test (*n* = 4, *p < 0.05; **p < 0.01; ***p < 0.001).

The effect of surgery and KA-induced seizures on the cytokine profile was also investigated in plasma samples. Surgical implantation of intracerebral electrodes caused a significant increase in the plasma concentrations of proinflammatory cytokines IL-1β, IL-5, IL-6, KC/GRO, and TNFα in the vehicle-treated group (Figure 6). KA treatment, post-surgery, had no effect on any of the plasma cytokines tested. However, KA in naive animals (without surgery) increased IL-2 and KC/GRO plasma levels (Figures 6C,G). The pleiotropic anti-inflammatory cytokine IL-10 was increased in the mice that had intracerebral electrodes and treated with the vehicle, but not KA, post-surgery (Figure 6J). There were no significant effects of either surgery or KA on plasma IFN-γ, IL-12p70, and IL-4 levels (Figures 6A,F,I).

It is unknown why there was increased plasma IL-1β in the vehicle surgery group but not in the KA group (Fig. 6B). However, in both KA groups, compared with the vehicle (no surgery), there was an increase in IL-1β but the differences were not statistically significant. The levels of IL-6 increased in both surgery groups (with or without KA) in the hippocampus (Figure 5E). In the plasma, we did observe increase of IL-6 in surgery group without KA, and an increase in KA without surgery, but the differences in the latter were not significant (Figure 6E). However, it is also unclear why there was no increase of IL-6 and KC/GRO levels in KA+surgery group, though either KA or surgery on their own caused an increase.

A summary of proteins that are relevant to epileptogenesis/epilepsy/trauma, detected by proteomics and cytokines/chemokines by multiplex assay, and the references are tabulated in Table 6.

**Table 6:** Summary of altered hippocampal proteins and cortical and plasma proinflammatory cytokines and chemokines between groups.

**Protein/cytokine/chemokine**	**No surgery**		**Surgery**		**References related to epilepsy/seizure/trauma**
	**Vehicle**	**KA**	**Vehicle**	**KA**	
**Gliosis related proteins**					
GFAP	++	+ + + +	+ + +	+ + + ++	(25, 26, 39, 40)
Vimentin	++	+ + + +	+ + +	+ + + ++	(41, 42)
Filamin A	++	++	+ + +	+ + +	(43, 44)
Heat shock protein beta-1 (HSP 25)	++	+ + + +	++	+ + + ++	(45)
Glutamine synthase	+ + + +	++	+ + +	++	(46, 47)
GAT-1	+ + + +	++	+ + +	++	(48, 49)
Complement C1qa	+	+ + +	+ + + +	+ + + +	(50–55)
Complement C1qb	++	+ + +	+ + + +	+ + + +	
Complement C1qc	++	+ + +	+ + + +	+ + + +	
**Neurodegeneration related proteins**					
Galectin-1	++	+ + +	+ + +	+ + + ++	(56)
Cystatin-C (Cystatin-3)	++	+ + +	++	+ + +	(4)
Cathepsin B	++	+ + +	++	+ + + +	(57–59)
**BBB integrity related proteins**					
Fibrinogen alpha chain	++	+ + + +	+	+ + +	(60, 61)
Alpha 2 macroglobulin	++	+ + +	+ + + +	+ + + +	(62)
Transferrin	++	+ + +	+ + + +	+ + + +	(63)
α1 antitrypsin	++	++	+ + +	+ + + +	(64)
**Cytokines and chemokines [+** **for cortical lysate and** **+** **for plasma; – (the minus sign) indicates undetected]**					
Interferon-γ	++ ++	+ + + + ++	++ + + + +	+ + + + ++	(65, 66)
Interleukin-1β	+ +	++ ++	+ + + + + + + +	+ + + ++	(67–69)
Interleukin-2	++ ++	++ + + + +	+ + + + + + +	+ + + + + + +	(70)
Interleukin-4	++ –	++ –	+ + + + –	+ + + + +	(71, 72)
Interleukin-5	+ + + +	++ ++	+ + + + + + +	+ + + + + + +	(72)
Interleukin-6	+ +	++ + + +	+ + + ++ + + + ++	+ + + ++	(66, 69, 73)
Interleukin-10	++ ++	++ ++	+ + + + + + + +	+ + + + ++	(66, 68, 74)
Interleukin-12p70	– –	++ ++	+ + + ++ –	+ + + ++ + + +	(71, 75)
KC/GRO (CX_3_CL)	+ +	+ + + + + + +	+ + + ++ + + + ++	+ + + + + +	(76, 77)
Tumor necrosis-α	++ ++	++ ++	+ + + + + + + +	+ + + + + + +	(66, 68, 69)

*Relative abundance in each group is indicated by a plus sign*.

## Discussion

### Intracranial Surgical Procedure Can Compromise Seizure Threshold for Chemoconvulsants

In this study, we observed a significant difference in KA sensitivity for inducing generalized convulsive seizures in animals that had intracerebral electrodes and without electrodes, suggesting that surgical procedure and intracerebral electrodes can impact seizure threshold in experimental models. This finding is consistent with our previous observation in both rat and mouse telemetry models in which the electrodes were placed epidurally (2, 15, 25, 26). Both hippocampal proteomics and cortical MSD cytokine analyses indicate that cellular processes involved in neuroinflammation (including gliosis and proinflammatory cytokines release), neurodegeneration, synaptic plasticity, and reduced blood–brain barrier integrity were significantly altered as a consequence of the surgery and implanted intracerebral electrodes.

Several studies have reported results from RNA microarrays from both experimental models of epilepsy and human samples, highlighting the role of certain genes in the development of epilepsy (78–84). Sampling transcription, however, cannot account for the numerous levels of post-transcriptional control, which continue to emerge (85). Proteomics studies, therefore, may offer a better insight into the molecular mechanisms of the development of epilepsy. The first proteomics study was from a C57BL/6J mouse model of epileptogenesis induced by intrahippocampal KA injection (27). They compared the proteins at 1, 3, and 30 days post-SE (27). Although their model involves intracranial surgery for KA injection, and the same strain of mouse as ours, intracerebral electrodes were not implanted in their study. Furthermore, intrahippocampal KA does not necessarily affect the cortex at an early stage of epileptogenesis, as in the intraperitoneal KA approach (25, 26, 86–88). Our recent proteomics on interventional studies in epileptogenesis in the C57BL/6J mouse model, too, did not use intracerebral electrodes (33). Another study on proteomics used an electric kindling in the amygdala of female rats to induce epilepsy (29). It is important to note that in this study, the electrodes penetrated the cerebral hemispheres (Figure 1), and the KA was administered as repeated low doses at 30 min intervals *via* the intraperitoneal route, 7 days after surgery, and the proteins were analyzed a further 7 days later.

### The Hippocampal Proteins That Mediate Neurodegeneration

Comparing across all four groups, using one-way ANOVA and Fisher's *post-hoc* test, we found a significant increase in some of the common proteins that have a role in neuroinflammation and neurodegeneration, the two important hallmarks of epileptogenesis associated with brain trauma or exposure to chemoconvulsants (2, 3, 15, 25, 26, 89, 90). Galectin-1 (Gal-1) was significantly increased in response to surgery with or without KA, but surgery seems to potentiate the KA effects on Gal-1 expression (Supplementary Figure 1). It binds to β-galactoside moieties present in cell surface glycoproteins of neurons and astrocytes in the brain (91, 92). It is a member of lectin family and a downstream effector of low affinity nerve growth factor receptor, p75^NTR^, and regulates apoptosis and axonal growth (93). A selective proapoptotic role of Gal-1 in a subpopulation of GABAergic interneurons has been demonstrated in a pilocarpine model of epilepsy (56). Gal-1 inhibits CD45 protein phosphatase and dephosphorylates Lyn kinase, a member of the Src tyrosine kinase family (94, 95). Interestingly, we also observed a significant reduction of another member of Src kinase, Abl interactor 1 (Abi-1), in surgery group with or without KA (Table 3). Abi-1 interacts with CaMKIIα and regulates dendritic growth and spine maturation (96, 97). CD45, a common leukocyte antigen, expression increases in epileptic brain due to leaky BBB and infiltrated leukocytes (98). We also demonstrated a significant increase in phosphorylated Src kinases in a mouse model of epileptogenesis (2), suggesting a role for galectin–CD45–Src kinase signaling pathway in epilepsy. Ras and Rab related proteins, and CaMKIIα control neuronal plasticity by coordinating dendritic filopodial motility and AMPA receptor turnover (96, 97). In our proteomics study, Ras-related proteins Rab-6A and R-Ras2, and CaMKIIα were significantly decreased in surgery groups with or without KA, and with KA on its own (without surgery) (Table 2, Supplementary Figure 1), implying that the aberrant neurite growth and excitatory receptor activity could exacerbate during epileptogenesis.

Cystatin-C (CysC) is an endogenous inhibitor of cysteine protease such as cathepsin B (CatB) (99, 100). Interestingly, we observed a significant increase of both CysC and CatB in surgery groups with or without KA, but KA on its own did not increase CatB (Table 2, Supplementary Figure 1). CysC modulates both neurodegeneration and neurogenesis, during epileptogenesis and in the established epilepsy (4). The increased expression of CysC was observed in glial cells in the molecular layer of the dentate gyrus and associated with granule cell dispersion in both rat model and human patients with TLE (4). High levels of CatB were reported in serum of human patients with surgically resected temporal lobes (59) and in glial cells of progressive myoclonic epilepsies (57), suggesting that CatB can be a potential therapeutic target for certain epilepsies (58).

### The Hippocampal Proteins Involved in BBB Integrity

KA-induced SE in intracerebral electrodes mice significantly increased the number of proteins related to BBB integrity. BBB dysfunction occurs within hours of the insult in traumatic brain injury patients and persists for days to weeks (101–104). Leaky BBB has also been reported as a frequent event in post-traumatic patients with epilepsy (105–107). BBB integrity is compromised in response to KA-induced SE in experimental models (25, 26, 108). The fibrinogen alpha chain (FAC), a plasma glycoprotein, was increased by 5.9-fold (*p* = 0.005) in the hippocampus in the surgery+KA group when compared with no surgery+KA group (Table 4). FAC protein infiltrates into the brain through a leaky BBB and promotes neuroinflammation (60, 61). FAC aids as an adhesive substrate for platelets, endothelial cells, and leukocytes including brain infiltrated monocytes and further increases vascular permeability by activating the extracellular signal-regulated kinase 1/2 (ERK1/2) pathways in traumatic brain injury (61, 90, 109). The significant increase of the other serum glycoproteins in the hippocampus, such as serum albumin, α2 macroglobulin, serotransferrin, and α1 protease inhibitor 4 (α1 antitrypsin), further confirms the compromised BBB integrity due to the brain trauma caused by intracerebral electrodes and subsequent exposure to KA. The brain infiltrated serum albumin binds to astrocytic transforming growth factor-beta (TGF-β) receptors, phosphorylates SMAD-2/3, increases the cytoskeletal proteins, and induces inflammatory signaling, thus causing reactive gliosis (25, 26, 110–115). Indeed, we observed a significant increase in three key cytoskeletal proteins: GFAP (2.7-fold, *p* = 0.0003), vimentin (2.8-fold, *p* = 0.0007), and filamin A (actin-binding protein 280, >2-fold, *p* = 0.006) in surgery groups. Immunohistochemistry of mice brain sections from the 7 day post-KA of electrode implanted animals confirmed the increased reactive astrogliosis (25, 26). In a model of acquired epilepsy with BBB dysfunction, serum albumin has been shown to induce excitatory synaptogenesis through astrocytic TGF-β/ALK5 signaling pathway (116). There are reports of filamin A pathology, as astrocytic inclusions, in human patients with epilepsy (43, 44).

### Glia-Related Proteins and Synaptic Plasticity in the Hippocampus

We observed a significant increase in heat shock protein 25 (HSPβ1 or HSP25) in KA treated animals with or without surgery (Table 2). HSP25 functions as a molecular chaperone and regulates phosphorylation and the axonal transport of neurofilament proteins (117). HSP25 was upregulated in astrocytes and persisted for a long term in the hippocampus after the induction of SE in a rat pilocarpine model (45). The glutamine synthase (GS) and voltage-dependent GABA-A transporter (GAT-1, encoded by SLC6A1) proteins were significantly decreased in KA groups with or without surgery (both, FDR < 0.01). GS was predominantly expressed in astrocytes and required for the synthesis of glutamate and ammonia in the brain. GS deficiency causes epilepsy in both humans and animal models (46, 47). In a recent study, a selective deletion of GS in the mouse cerebral cortex induced glial dysfunction and vascular impairment (leaky BBB) that preceded the onset of epilepsy and neurodegeneration (118). Gat-1 is one of the major GABA transporters in the brain and is responsible for re-uptake of GABA by astrocytes at the synapses. The studies in human patients revealed GAT-1 mutation/polymorphism in febrile seizures, myoclonic-atonic seizures, and TLE (48, 49).

The activation of astrocytes and the induction of excitatory synaptogenesis exacerbates the hyperexcitability of neurons, which we had previously shown this phenomenon as an increased epileptiform spiking during the first week of epileptogenesis (2, 25, 26, 31). Activated astrocytes compromise extracellular buffering at synapses by downregulating Kir1.4 ion channels (25, 26). In this study, we also found a significant change in the synaptic vesicle cycle and glutathione metabolism pathways (Supplementary Table 5). We have recently shown that activated astrocytes after SE induction produce complement C and chemokines, which attract microglia at the synaptic terminals/neurons (3, 23). In this study, we found a significant increase in C1q related proteins in the surgery group with or without KA exposure, suggesting the role of these proteins in neuroinflammation and epileptogenesis. Several studies have demonstrated both astrogliosis and microgliosis during epileptogenesis and epilepsy (2, 3, 39, 40). A significant increase in GFAP, vimentin, filamin A, and C1q proteins in this study confirms that the proteomics method and the analysis are reliable and reiterate the plausible role of the identified proteins in the process of onset of spontaneous recurrent seizures/epileptogenesis.

### The Cortical and Peripheral Cytokines in Response to Surgery and/or KA

Reactive astrocytes and microglia produce proinflammatory cytokines (39, 40). The cytokine assay of the cerebral cortex and the plasma from the same animals in our study showed a significant increase in some of the key proinflammatory cytokines in the surgery group with or without KA when compared with the group without surgery (Figures 5, 6). Proinflammatory cytokines are well-known to lower the seizure threshold (39, 40). Both hippocampus and cortex were affected in both human and animal models of temporal lobe epilepsy (25, 26, 86, 119–121). The epileptogenic network that involves the initiation and propagation of seizures has been well-defined in both humans and experimental models of epilepsy (115, 122, 123). Our previous work in the C57BL/6J mouse KA model, focused on the hippocampus, the entorhinal cortex, and the amygdala, suggested that the hippocampal gliosis and neurodegenerative changes were maximally affected at 7 days post-KA but beginning to decrease thereafter in contrast to the rat KA model of progressive epilepsy (25, 26). Interestingly, cortical changes persisted. Continuous video-EEG monitoring from these mice revealed persistent non-convulsive seizures (26). Therefore, in this study, we used the hippocampus for protein profiling and the cerebral cortex, where the electrodes penetrated the brain, for cytokine assay at 7 days post-KA (and plasma from the same animals). It has been demonstrated that the localized electrode-induced trauma causes localized gliosis (124–126). We were also interested in determining whether the impact of surgery and KA-induced SE or KA, on its own, would change cytokine profiles in the plasma, which may serve as a biomarker for epileptogenesis in experimental models. Indeed, we observed a significant increase in pro-inflammatory cytokines IFN-γ, IL-1β, IL-5, 1L-6, IL-10, IL-12p70, KC/GRO (also known as CXCL1), and TNFα in the cerebral cortex in the surgery group, and some in KA, suggesting the occurrence of neuroinflammation due to implanted electrodes and/or KA. Appropriate references with respect to the roles of these cytokines in epilepsy/seizure/trauma are included in Table 6. In the plasma of KA-induced SE in animals without surgery, only IL-2 and KC/GRO were upregulated. Interestingly, IL-2 was unaffected in the cerebral cortex, implying the peripheral effects. Although there is no literature on either plasma/serum or cerebral tissue cytokines at 14 days post-trauma, there are reports on early time-points demonstrating upregulation of some of these cytokines (89, 127). Therefore, the plausible reason for the reduced seizure threshold in the mice that had surgery could be due to localized cerebral inflammation, which seems to have persisted for a further 7 days as a result of the implanted electrodes.

The occurrence of seizures immediately prior to euthanasia could affect some of the cytokine levels. In this study, we did not monitor the animals for seizures throughout. Once a spontaneous seizure was confirmed in a mouse, the recording was stopped. An important variable in this study and Ravizza et al. (128) is the model (pilocarpine rat model vs. KA mouse model). The rat pilocarpine model is a progressive epilepsy model, while C57BL/6J mouse KA model is a regressive model (convulsive seizures are infrequent) (129).

### The Limitations of This Study

The proteins discussed in this study, focused on the effects of surgery and KA on the development of epilepsy, are based on the literature. We have investigated the hippocampus only for proteomes and the cortex only for cytokines at 7 day post-KA (only one time-point), and the other epileptogenic areas and the time-points were not investigated. It has been suggested that the endogenous proresolving molecules such as lipoxins, resolvins, protectins, and maresins synthesis occur within hours to days of insult (130). In this study, none of the proresolving endogenous mediators of inflammation were significantly altered in any groups. Since we did proteomics at 14 days post-surgery or 7 days post-SE, it is likely that the resolution phase has passed. For example, the brain infiltrated monocytes and leucocytes persist only during the first 3 days of chemoconvulsant-induced SE (131). The altered levels of proteins or cytokines/chemokines alone do not necessarily affect the brain function *per se*. In this context, further studies are needed to determine the cell types and the receptor subtype expression in different regions of the brain at various time points post-SE to determine the functional outcome.

A summary of proteins identified in this study with a known role in seizures/epilepsy/trauma are listed in Table 6. In addition to the proteins discussed, there are many other proteins that may have a role in epileptogenesis that were not detected in this study due to single time point analysis. However, a few other proteins that were altered due to surgery and/or KA are briefly mentioned here. VGF, a BDNF-inducible peptide precursor, regulates fear-associated memory formation in hippocampus (132–134). Glypican-1, a member of the heparin sulfate proteoglycans, mediates phosphatidylinositol glycan signal propagation and receptor activation in the brain. Although glypican-4 has been demonstrated in the brains of epileptic patients and epileptic animals (135), the role of glypican-1 in epileptogenesis is yet unknown. Unconventional myosin-Va is required for neuronal plasticity, motor learning, oligodendrocyte morphogenesis, and myelination (136, 137), but its role in epileptogenesis is also unknown. Further mining of the proteomics data can yield other molecular mechanisms that may be affected by surgery and KA. The raw data and analyzed data are available at https://github.com/ISUgenomics/2020_Thippeswamy_Surgery-Proteomics/tree/ or *via* ProteomeXchange (Project doi: 10.6019/PXD021554). The results of this study did not show significant changes in the other proteins of certain pathways such as glutamate receptor activation, immunomodulating effects, or calcium homeostasis; however, the identified proteins having a significant role in inflammation, neurodegeneration, BBB integrity, and gliosis support the hypotheses of the potential impact of intracerebral electrodes on brain pathology and electrical activity. Surgery and intracerebral electrodes can alter chemoconvulsants' sensitivity in animals and appear to have a higher impact on altering the expression of proteins involved in epilepsy than KA itself. Thus, it can be misleading to assume that the development of epilepsy observed in EEG will reflect what occurs in brains without electrodes. These findings should give insights into the impact of intracranial surgery on epilepsy development and should be considered in the design of epilepsy studies and the interpretation of data arising from models in which intracranial procedures have been used. It should be noted that the sample size used in this study is small (*n* = 4) as in the rat pilocarpine model (28). However, a combination of proteomics and multiplex assay from two different brain regions, and the plasma assay, revealed the altered key proteins in trauma and/or KA-induced brain injury.

In conclusion, surgery and intracerebral electrodes reduce seizure threshold and increase the key proteins that regulate the BBB function, neuroinflammation, and neurodegeneration as revealed by the proteomics at 7 days post-KA. The cytokine assay from the cerebral cortex and plasma suggest that the intracerebral electrode-induced proinflammatory cytokine responses may partly contribute to the decreased seizure threshold but not necessarily promote epileptogenesis, which was not tested in this study. It should also be noted that some of the inflammatory molecules (IL-6, IL-1beta, KC/GRO) reported in this study are inconsistent and require further investigation. The transient inflammatory responses evoked by surgery and electrodes procedures (persistent responses at different time-points were not tested in this study) unlikely have an impact on the development of epilepsy; otherwise, it is difficult to extrapolate the refined experimental models of SE induction by electrical stimulation in which only a small percentage of animals develop epilepsy (138). Our past EEG studies in the C57BL/6J mice did not show epileptiform spikes in their baseline EEG during the first 10 days of continuous (24/7) EEG monitoring post-surgery before KA challenge, suggesting that mere electrode implantation *per se* unlikely initiates epileptogenesis (129). However, the surgical procedure decreases the seizure threshold; therefore, to reduce mortality in KA models, irrespective of rodent species, a repeated low-dose method of administering KA would be advantageous (32, 139).

## Data Availability Statement

The datasets presented in this study can be found in online repositories. The names of the repository/repositories and accession number(s) can be found at: ProteomeXchange, http://www.proteomexchange.org/, PXD021554.

## Ethics Statement

The animal study was reviewed and approved by The University of Liverpool Ethics Committee as per the Animal (Scientific Procedures) Act, 1986 (U.K).

## Author Contributions

TT conceived the idea and secured funding in collaboration with GS. TT and RB designed experiments. KT and EB conducted *in vivo* experiments. KT performed MSD assay. KT and GS analyzed the data. DS, KT, and RB conducted proteomics experiments. TT wrote the manuscript. KT wrote the Methods section and some of the Introduction. EB edited and proof-read the first manuscript. All authors contributed to the article and approved the submitted version.

## Conflict of Interest

The authors declare that the research was conducted in the absence of any commercial or financial relationships that could be construed as a potential conflict of interest.

## Supplementary Material

Supplementary Figure 1 Box plots for all 40 significant proteins identified by one-way ANOVA.

Supplementary Figure 2 The schematic diagrams of KEGG pathways (vehicle: surgery vs. no surgery).

Supplementary Figure 3 The schematic diagrams of KEGG pathways (KA: surgery vs. no surgery).

Supplementary Table 1 List of all proteins identified by comparing all four groups using one-way ANOVA.

Supplementary Table 2 The impact of intracerebral electrode implants on the expression of proteins in the hippocampus. The groups compared were between surgery vs. no surgery treated with vehicle (distilled water), and all the proteins that were significantly altered by *p* > 0.01 are listed and the proteins with >2-fold change are highlighted.

Supplementary Table 3 The impact of KA-induced SE in intracerebral electrode implanted animals on the expression of proteins in the hippocampus. The groups compared were between surgery vs. no surgery treated with KA, and all the proteins that were significantly altered by *p* > 0.01 are listed and the proteins with >2-fold change are highlighted.

Supplementary Table 4 The list of KEGG pathways and the key proteins of the pathways affected by intracerebral electrode implants. The groups compared were between surgery vs. no surgery and treated with the vehicle (distilled water).

Supplementary Table 5 The list of KEGG pathways and the key proteins of the pathways affected by intracerebral electrode implants and KA-induced SE. The groups compared were between surgery vs. no surgery and treated with KA.
