# Supplementary material for: The Impacts of Surgery and Intracerebral Electrodes in C57BL/6J Mouse Kainate Model of Epileptogenesis: Seizure Threshold, Proteomics, and Cytokine Profiles
Source: Front Neurol. 2021 Jul 12;12:625017. doi: 10.3389/fneur.2021.625017 (PMC8312573; doi:10.3389/fneur.2021.625017)
Supplement: Supplementary Figure 1 — Box plots for all 40 significant proteins identified by one-way ANOVA. [file Data_Sheet_1.PDF]

Uniprot ID	Protein names	Gene names	f.value	p.value	neg log10(p)	FDR	Abundance on log scale			
							Veh-NoSurg	Veh-Surg	KA-NoSurg	KA-Surgery
**Proteins upregulated in surgery vehicle and both KA groups**										
P03995	Glial fibrillary acidic protein (GFAP)	Gfap	31.792	5.43E-06	5.265	0.00138	25.55244	27.025413	26.87475	27.968345
P20152	Vimentin	Vim	20.7	4.91E-05	4.3091	0.00361	23.70164	25.16986	24.87103	26.487395
P98086	Complement C1qa	C1qa	15.099	0.000224	3.6494	0.00901	17.52368	19.70244	18.78533	19.755233
P14106	Complement C1qb	C1qb	25.31	1.78E-05	4.7495	0.00191	19.04926	20.753115	19.97015	21.130523
Q02105	Complement C1qc	C1qc	16.908	0.000131	3.8812	0.00633	18.60421	20.583218	19.43826	20.605043
P16045	Galectin-1 (Gal-1/Galaptin)	Lgals1 Gbp	23.294	2.72E-05	4.566	0.00244	18.55418	19.299368	19.29811	20.263458
P21460	Cystatin-C (Cystatin-3)	Cst3	15.395	0.000205	3.6888	0.00869	21.29772	21.565828	21.74042	22.158848
P62301	40S ribosomal protein S13	Rps13	20.655	4.96E-05	4.3044	0.00361	21.70742	21.852868	21.85315	21.976885
**Proteins downregulated in surgery vehicle and both KA groups**										
P35279	Ras-related protein Rab-6A (Rab-6)	Rab6a	18.567	8.37E-05	4.077	0.00553	21.63157	21.371815	21.51557	21.370305
P62071	Ras-related protein R-Ras2	Rras2	17.853	0.000101	3.9946	0.00576	20.50515	20.053165	20.35101	19.987185
P11798	Calcium/calmodulin-dependent protein kinase IIa (CaMK-IIa)	Camk2a	24.743	0.00002	4.6992	0.00191	28.23778	28.094555	28.03671	27.681833

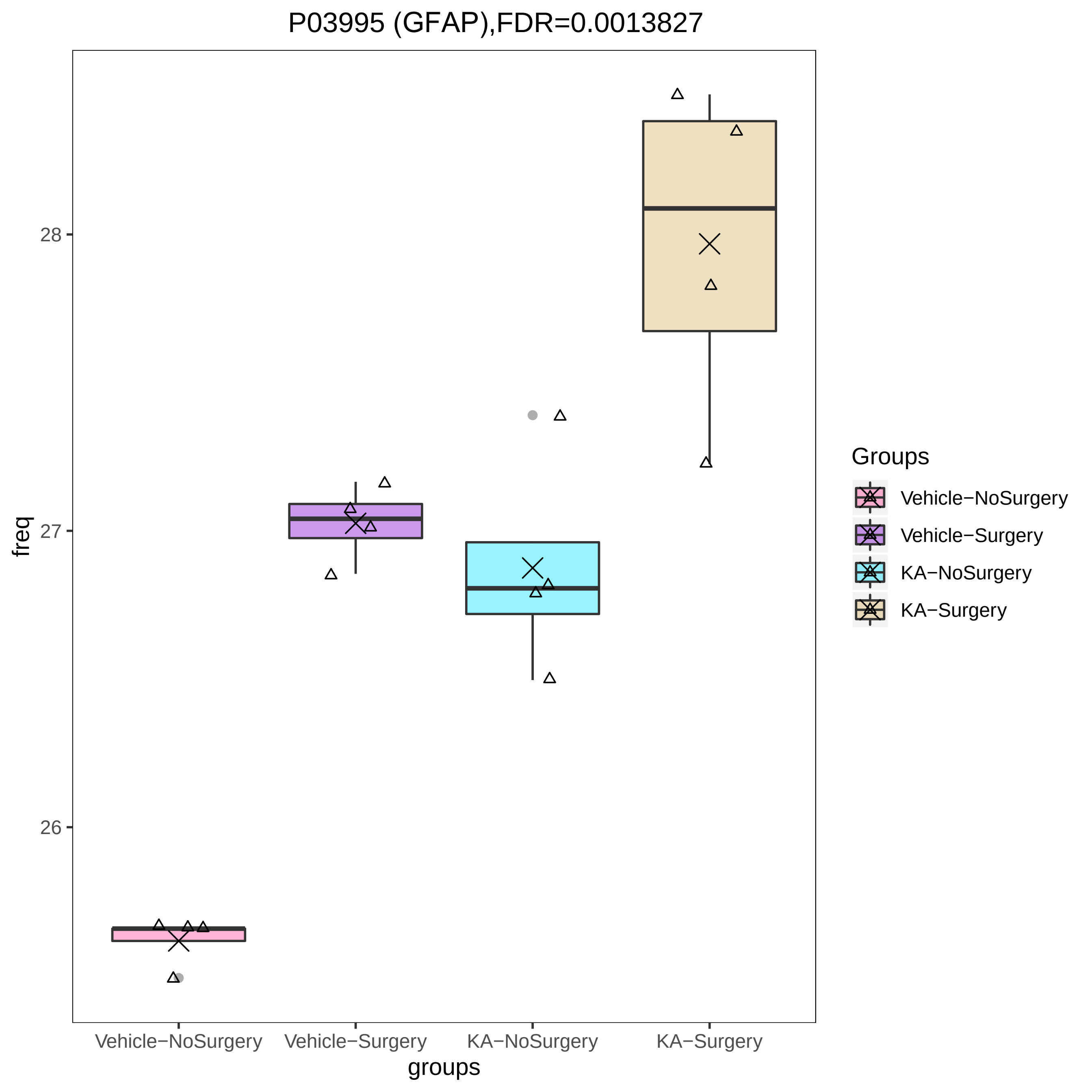
P03995 (GFAP),FDR=0.0013827
freq
28
27
26
groups
Vehicle−NoSurgery
Vehicle−Surgery
KA−NoSurgery
KA−Surgery
Groups
Vehicle−NoSurgery
Vehicle−Surgery
KA−NoSurgery
KA−Surgery

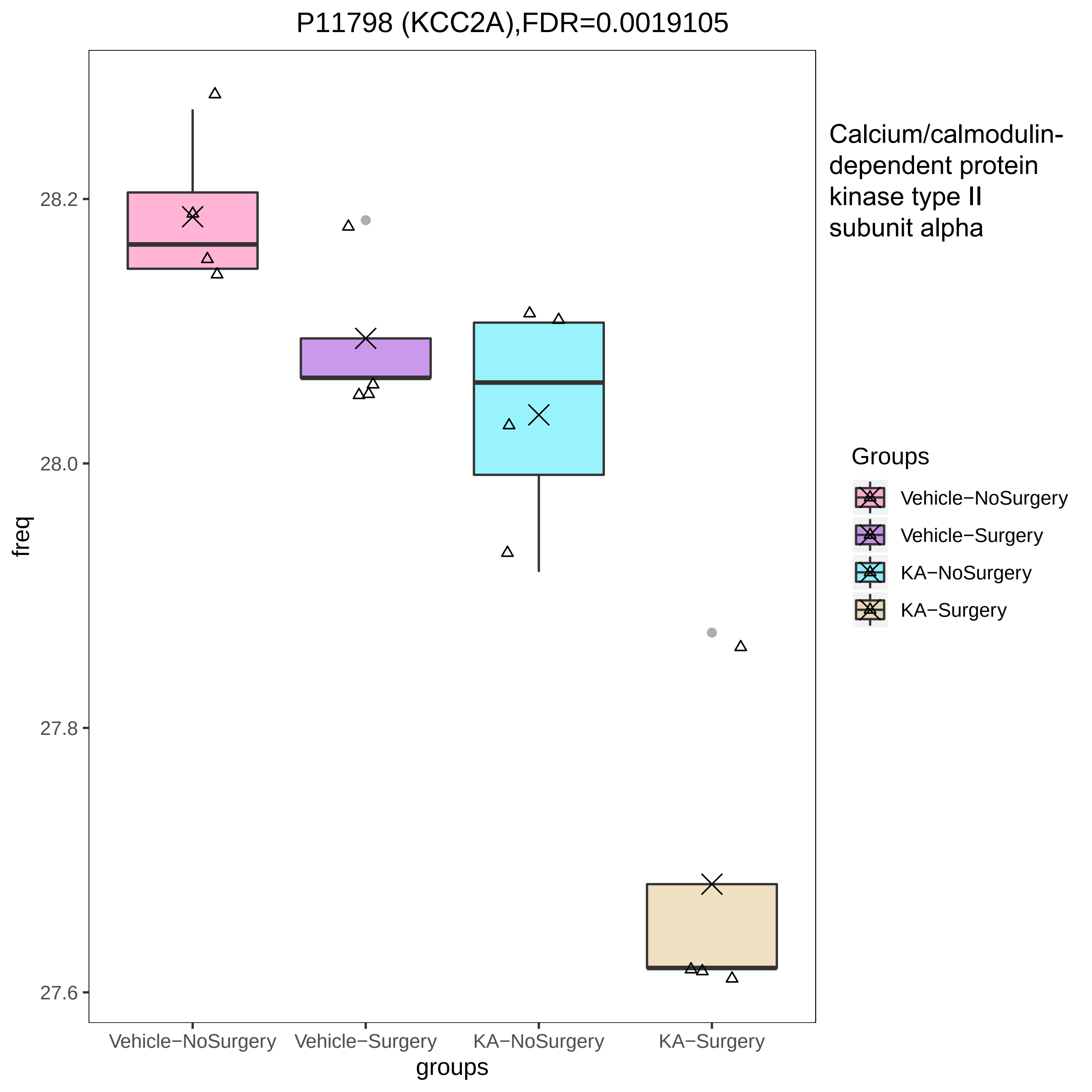

P11798 (KCC2A),FDR=0.0019105
Calcium/calmodulin-dependent protein kinase type II subunit alpha
freq
28.2
28.0
27.8
27.6
groups
Vehicle−NoSurgery
Vehicle−Surgery
KA−NoSurgery
KA−Surgery
Groups
Vehicle−NoSurgery
Vehicle−Surgery
KA−NoSurgery
KA−Surgery

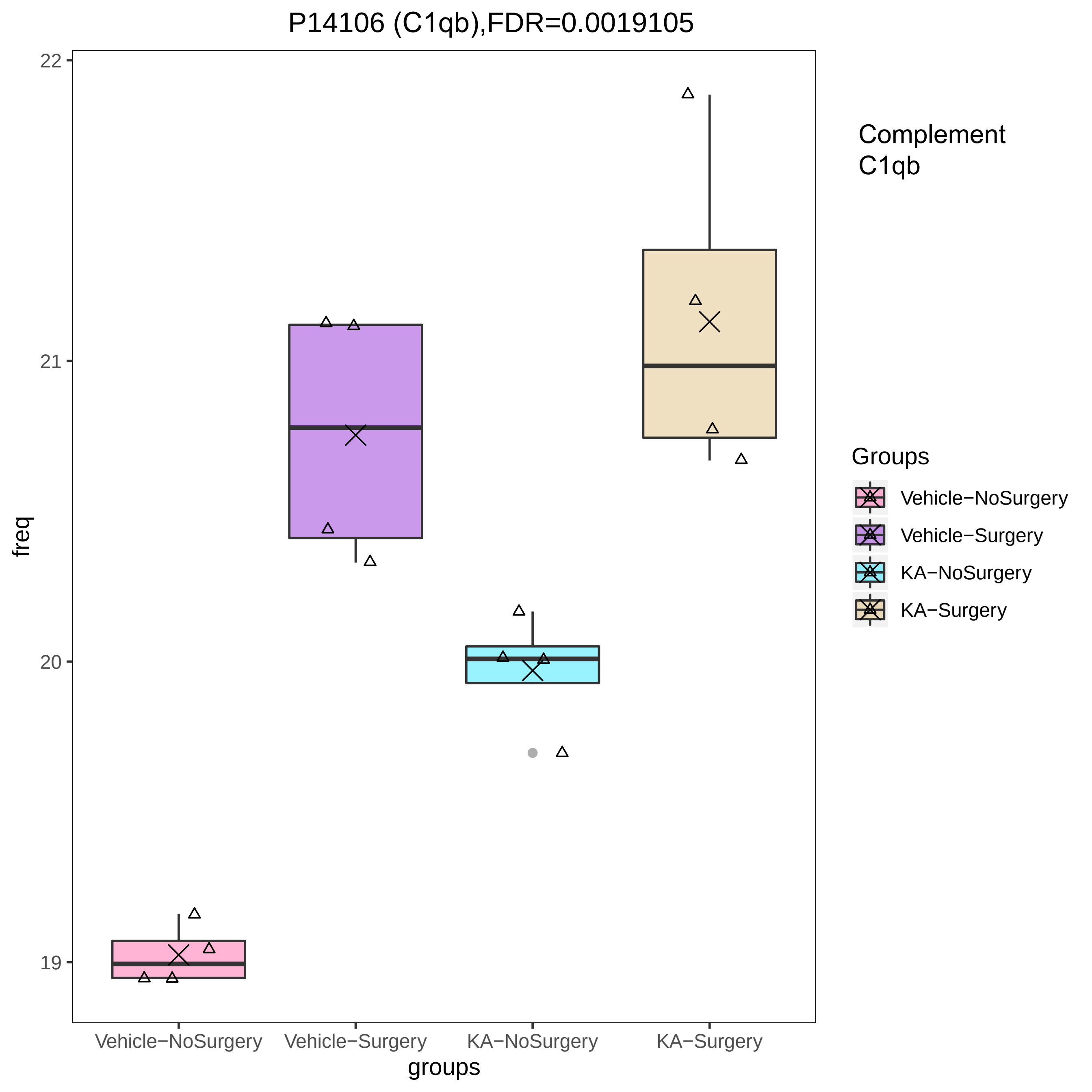
P14106 (C1qb),FDR=0.0019105
Complement
C1qb
Groups
Vehicle−NoSurgery
Vehicle−Surgery
KA−NoSurgery
KA−Surgery
freq
22
21
20
19
Vehicle−NoSurgery
Vehicle−Surgery
KA−NoSurgery
KA−Surgery
groups

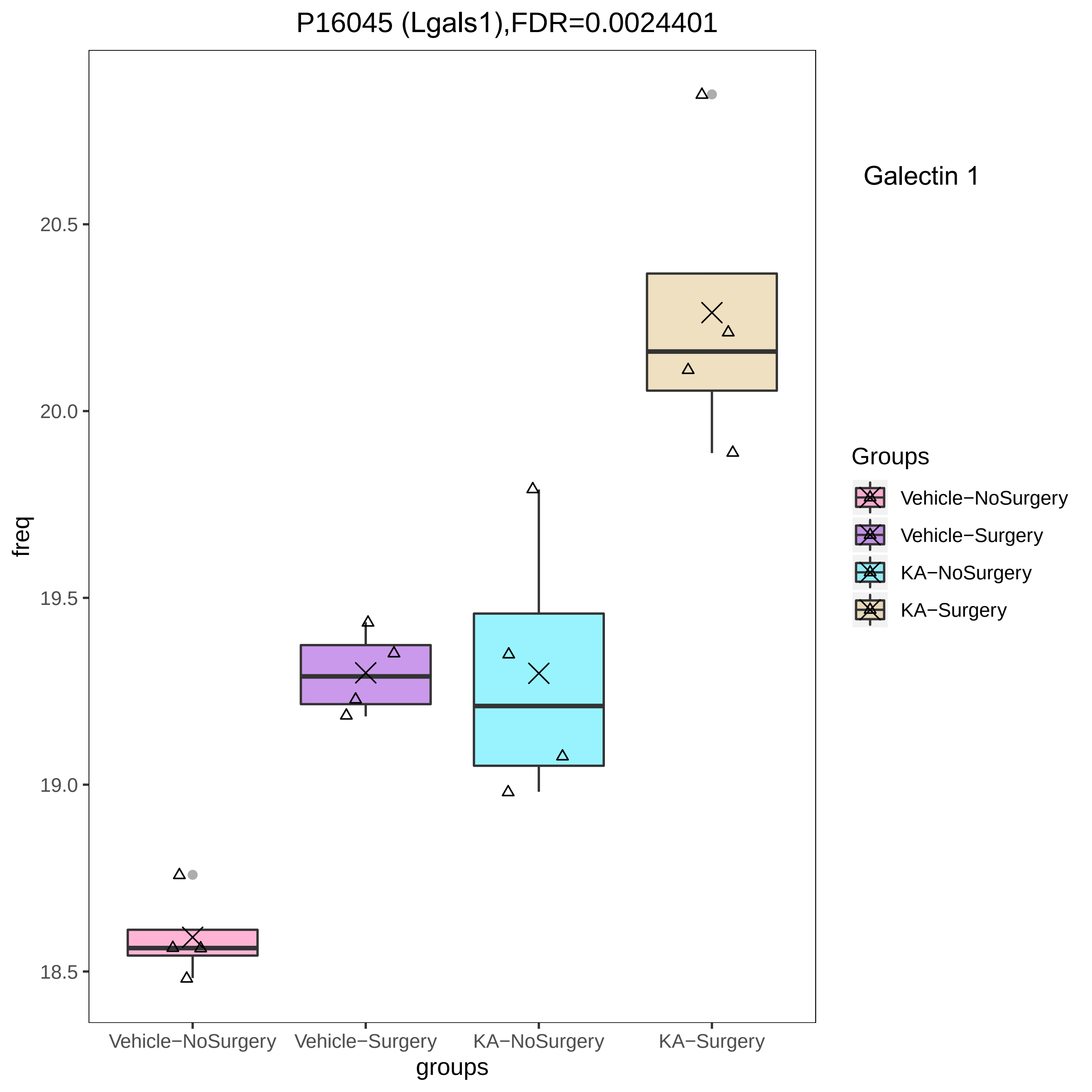
P16045 (Lgals1),FDR=0.0024401
Galectin 1
freq
20.5
20.0
19.5
19.0
18.5
Vehicle−NoSurgery
Vehicle−Surgery
KA−NoSurgery
KA−Surgery
groups
Groups
Vehicle−NoSurgery
Vehicle−Surgery
KA−NoSurgery
KA−Surgery

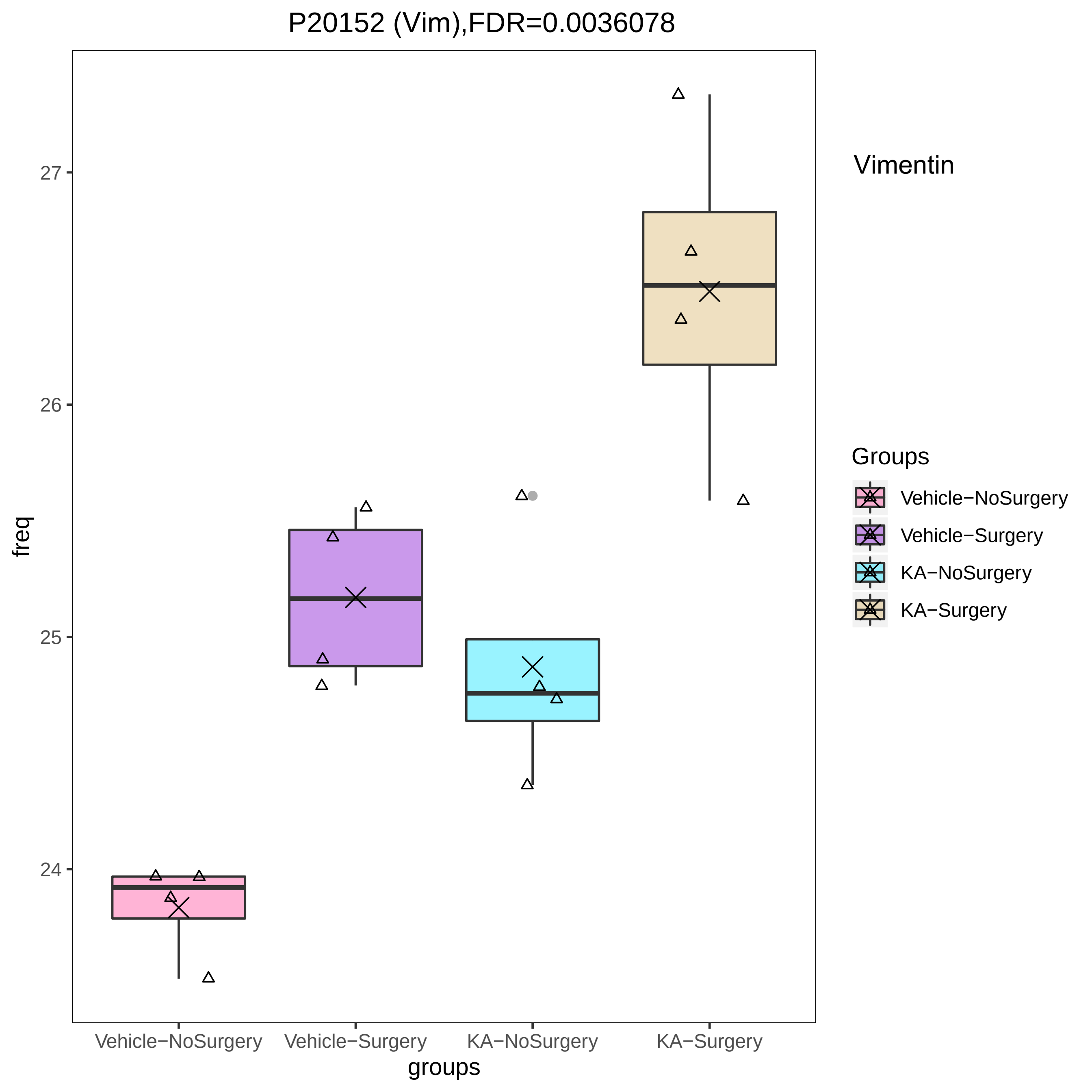

P20152 (Vim),FDR=0.0036078
Vimentin
freq
27
26
25
24
groups
Vehicle−NoSurgery
Vehicle−Surgery
KA−NoSurgery
KA−Surgery
Groups
Vehicle−NoSurgery
Vehicle−Surgery
KA−NoSurgery
KA−Surgery

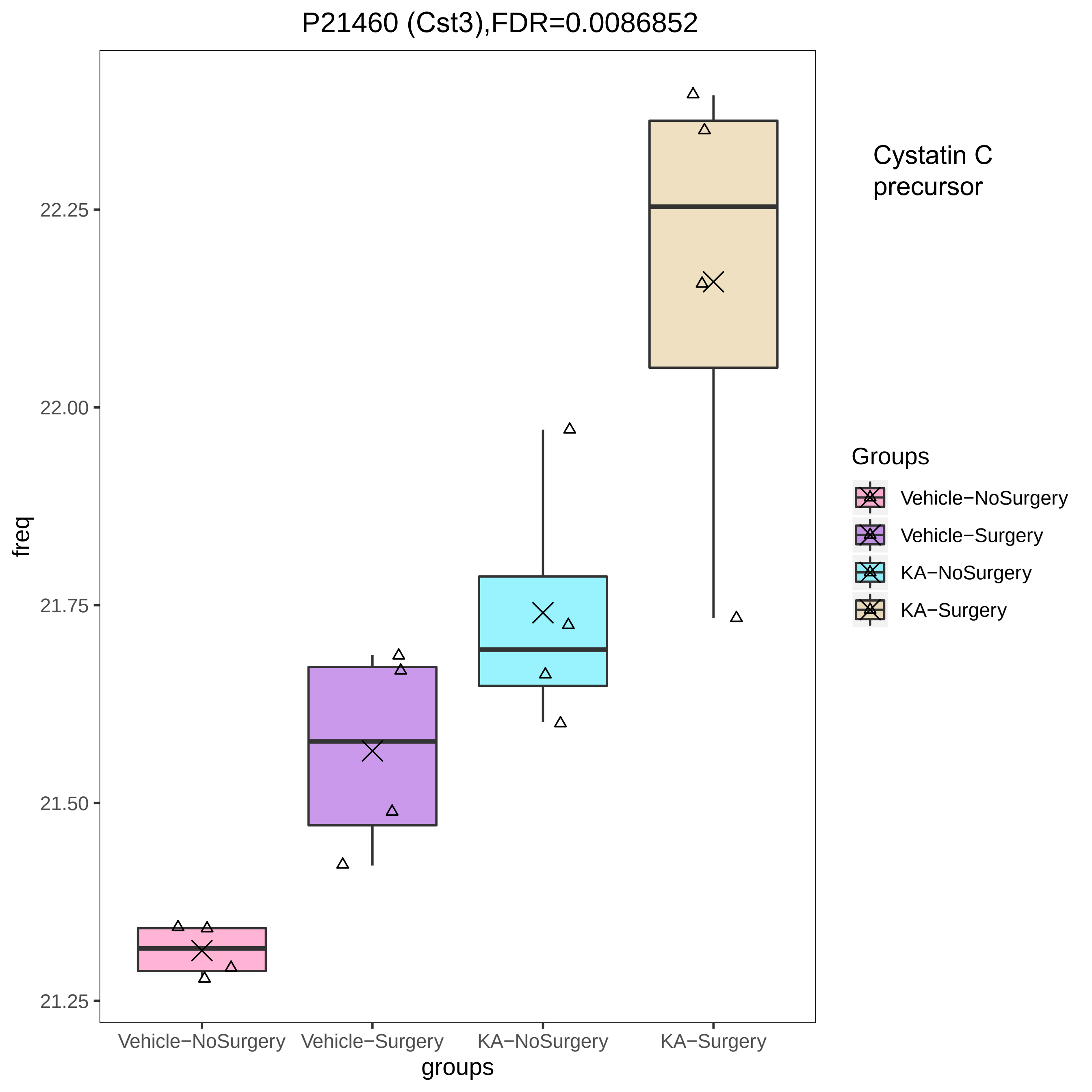

P21460 (Cst3),FDR=0.0086852
Cystatin C
precursor
freq
22.25
22.00
21.75
21.50
21.25
Vehicle−NoSurgery
Vehicle−Surgery
KA−NoSurgery
KA−Surgery
groups
Groups
Vehicle−NoSurgery
Vehicle−Surgery
KA−NoSurgery
KA−Surgery

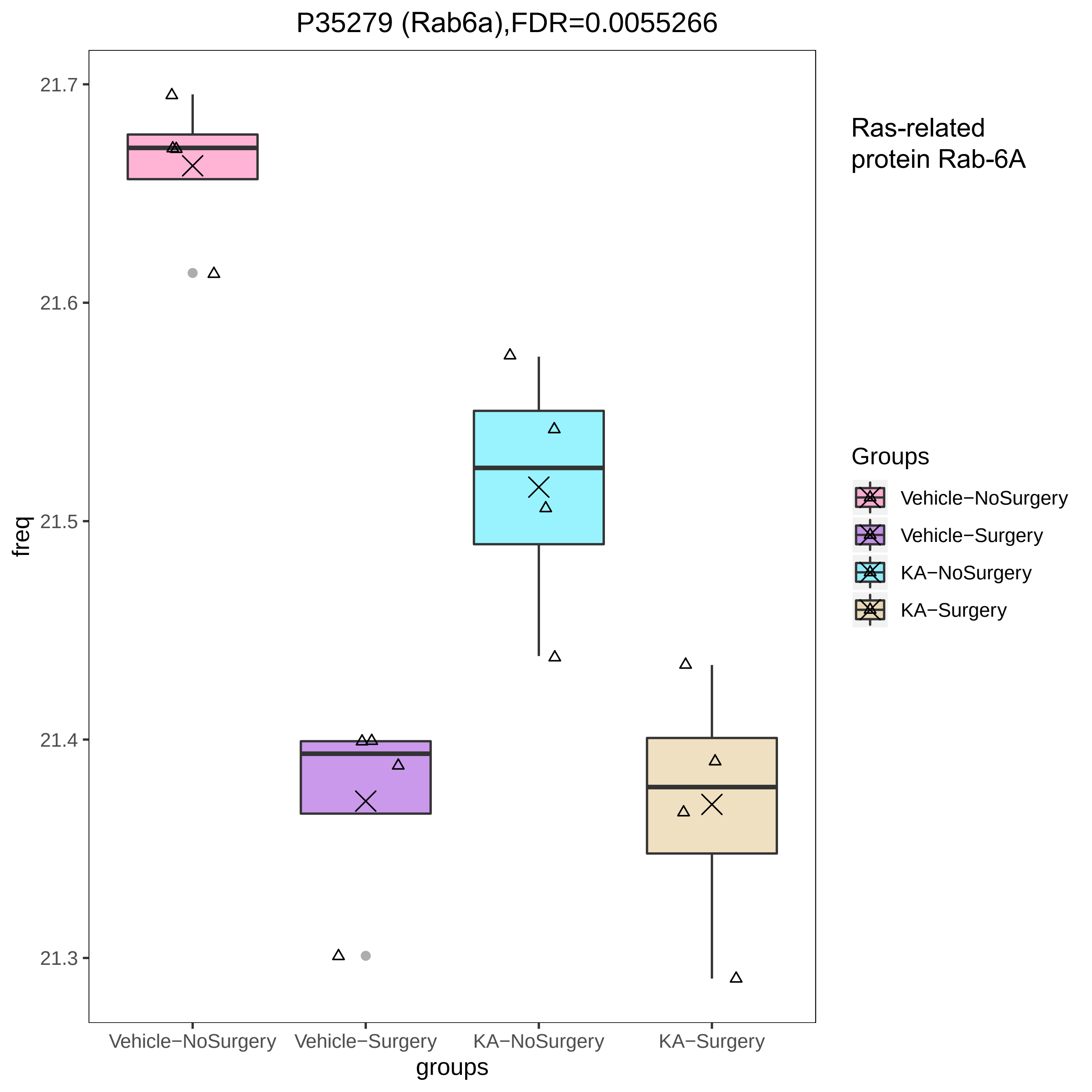
P35279 (Rab6a),FDR=0.0055266
Ras-related protein Rab-6A
freq
21.7
21.6
21.5
21.4
21.3
Vehicle−NoSurgery
Vehicle−Surgery
KA−NoSurgery
KA−Surgery
groups
Groups
Vehicle−NoSurgery
Vehicle−Surgery
KA−NoSurgery
KA−Surgery

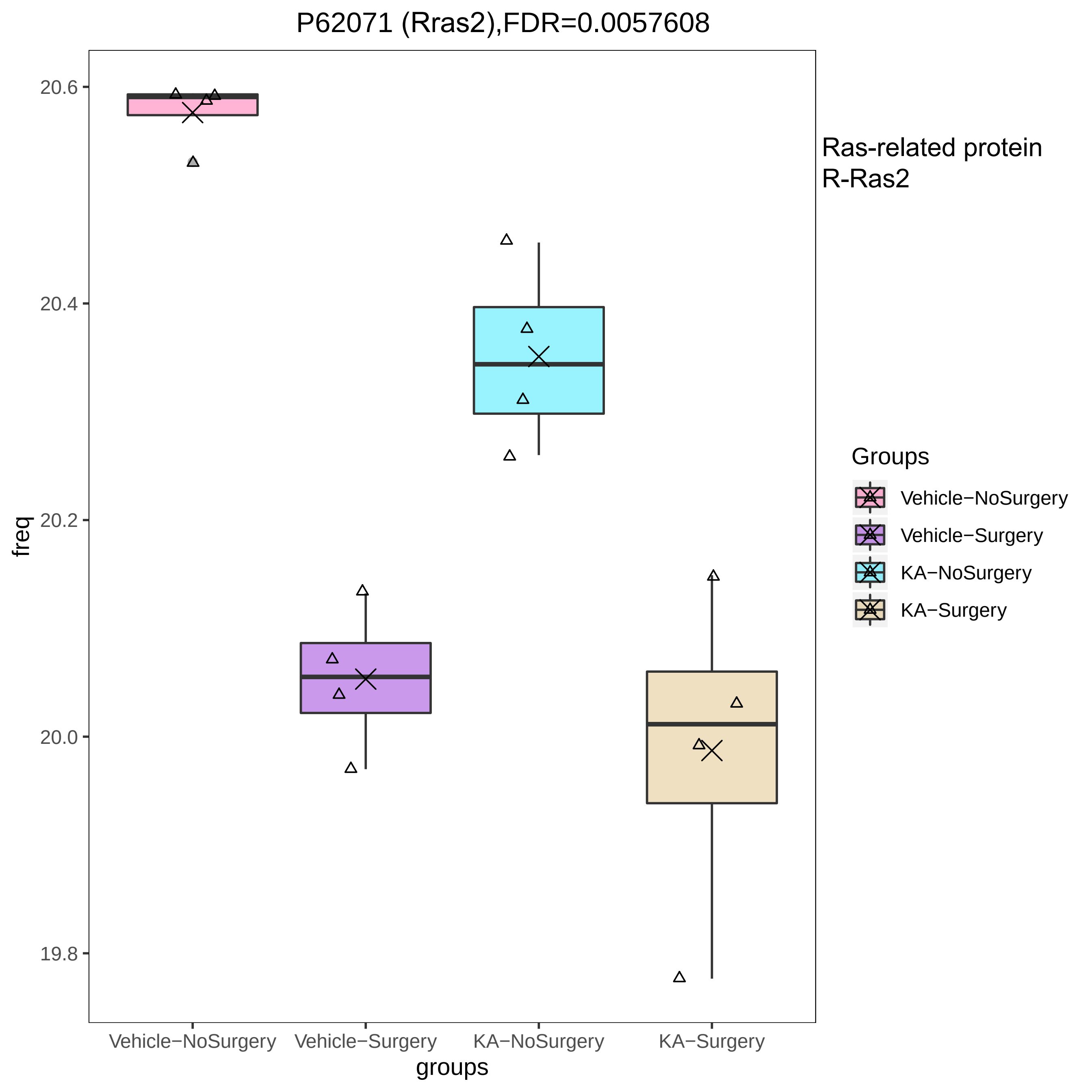
P62071 (Rras2),FDR=0.0057608
Ras-related protein
R-Ras2
freq
20.6
20.4
20.2
20.0
19.8
Groups
Vehicle−NoSurgery
Vehicle−Surgery
KA−NoSurgery
KA−Surgery
Vehicle−NoSurgery
Vehicle−Surgery
KA−NoSurgery
KA−Surgery
groups

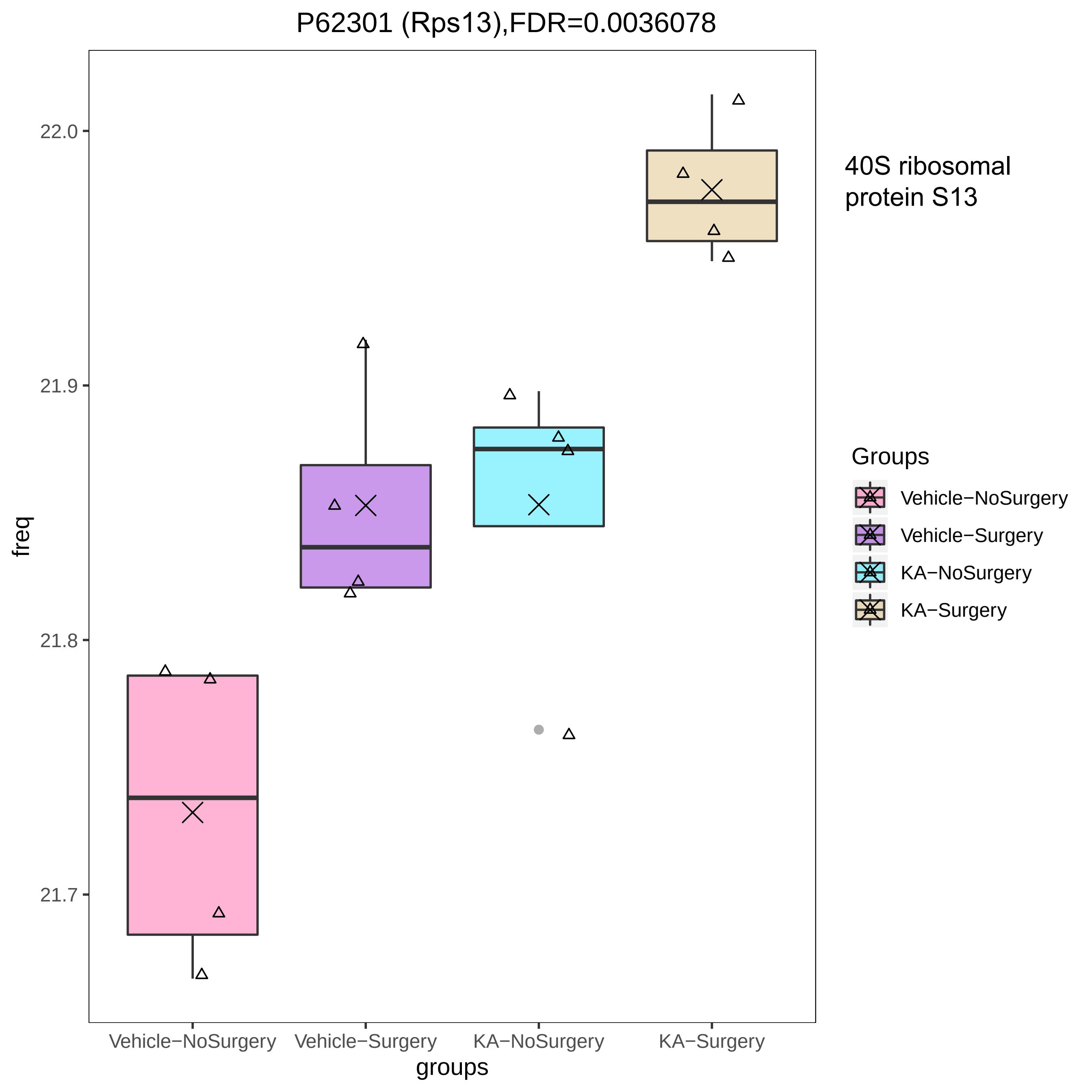

P62301 (Rps13),FDR=0.0036078
40S ribosomal
protein S13
freq
22.0
21.9
21.8
21.7
groups
Vehicle−NoSurgery
Vehicle−Surgery
KA−NoSurgery
KA−Surgery
Groups
Vehicle−NoSurgery
Vehicle−Surgery
KA−NoSurgery
KA−Surgery

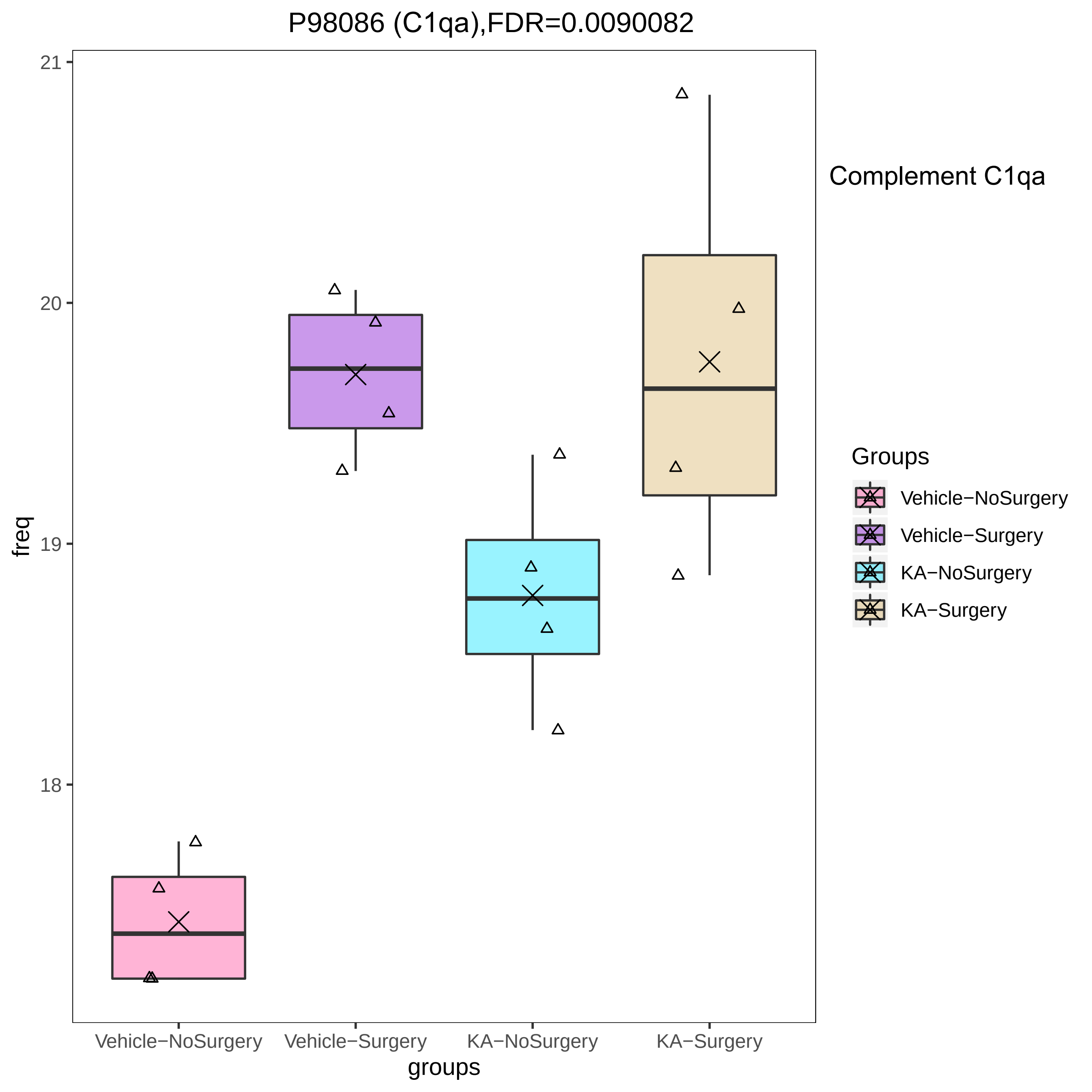
P98086 (C1qa),FDR=0.0090082
Complement C1qa
freq
21
20
19
18
groups
Vehicle−NoSurgery
Vehicle−Surgery
KA−NoSurgery
KA−Surgery
Groups
Vehicle−NoSurgery
Vehicle−Surgery
KA−NoSurgery
KA−Surgery

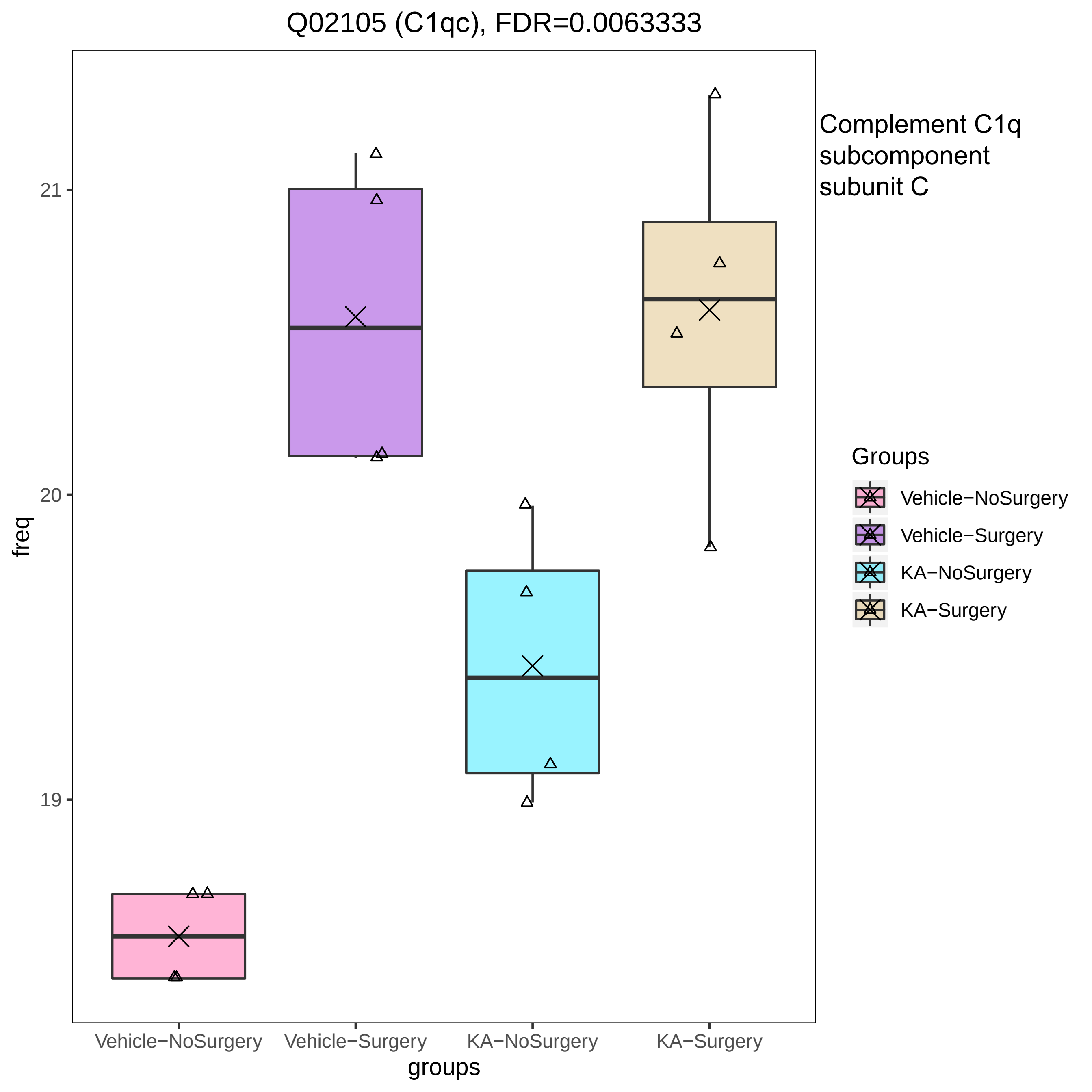

Q02105 (C1qc), FDR=0.0063333
Complement C1q
subcomponent
subunit C
freq
21
20
19
groups
Vehicle−NoSurgery
Vehicle−Surgery
KA−NoSurgery
KA−Surgery
Groups
Vehicle−NoSurgery
Vehicle−Surgery
KA−NoSurgery
KA−Surgery

Uniprot ID	Protein names	Gene names	f.value	p.value	neg log10(p)	FDR	Abundance on log scale			
							Veh-NoSurg	Veh-Surg	KA-NoSurg	KA-Surgery
**Proteins upregulated in both KA groups (with or without surgery)**										
Q9QZF2	Glypican-1 [Cleaved into: Secreted glypican-1]	Gpc1	33.445	4.15E-06	5.3816	0.00138	19.79506	19.762235	20.0398	20.213773
P14602	Heat shock protein beta-1 (HspB1/ HSP 25)	Hspb1 Hsp25/27	26.34	1.45E-05	4.8385	0.00191	18.34595	18.024863	20.10053	21.608808
Q99104	Unconventional myosin-Va	Myo5a Dilute	17.692	0.000106	3.9756	0.00576	23.74006	23.75106	23.87258	23.905998
Q0VGU4	Neurosecretory protein VGF (VGF-derived peptide TLQP-62)	Vgf	17.569	0.000109	3.961	0.00576	20.14906	19.824463	20.7232	21.123153
Q9D8Y0	EF-hand domain-containing protein D2 (Swiprosin-1)	Efhd2 Sws1	17.443	0.000113	3.946	0.00576	23.16364	23.261423	23.45649	23.668985
**Proteins downregulated in both KA groups (with or without surgery)**										
P31648	Sodium- and chloride-dependent GABA transporter 1 (GAT-1)	Slc6a1 Gabt1 Gat-1	17.805	0.000103	3.9889	0.00576	23.44462	23.413773	23.06983	22.927308
P15105	Glutamine synthetase (GS)	Glul Glns	14.75	0.00025	3.6022	0.00969	26.8151	26.797415	26.51625	26.618135

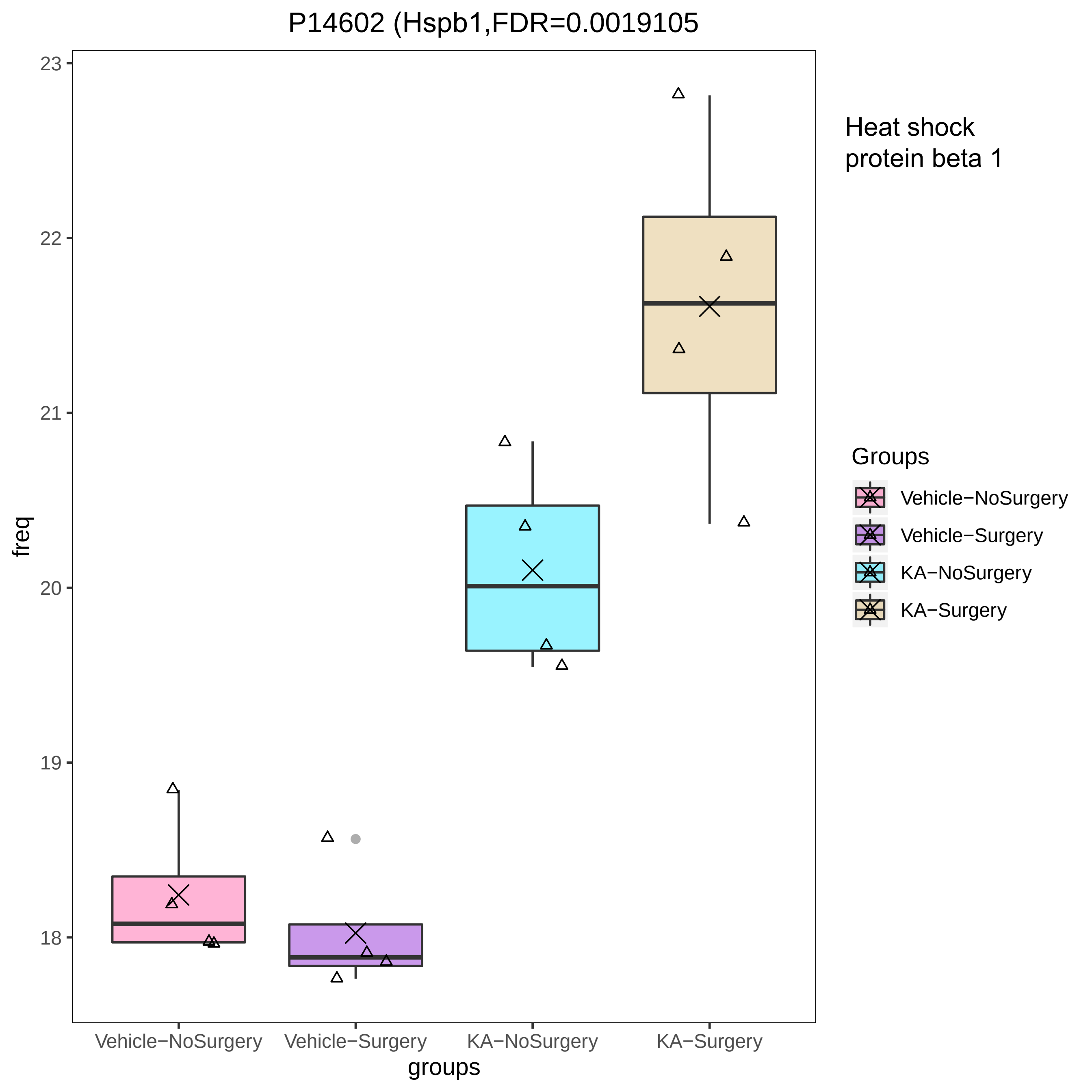
P14602 (Hspb1,FDR=0.0019105
Heat shock
protein beta 1
freq
23
22
21
20
19
18
Vehicle−NoSurgery
Vehicle−Surgery
KA−NoSurgery
KA−Surgery
groups
Groups
Vehicle−NoSurgery
Vehicle−Surgery
KA−NoSurgery
KA−Surgery

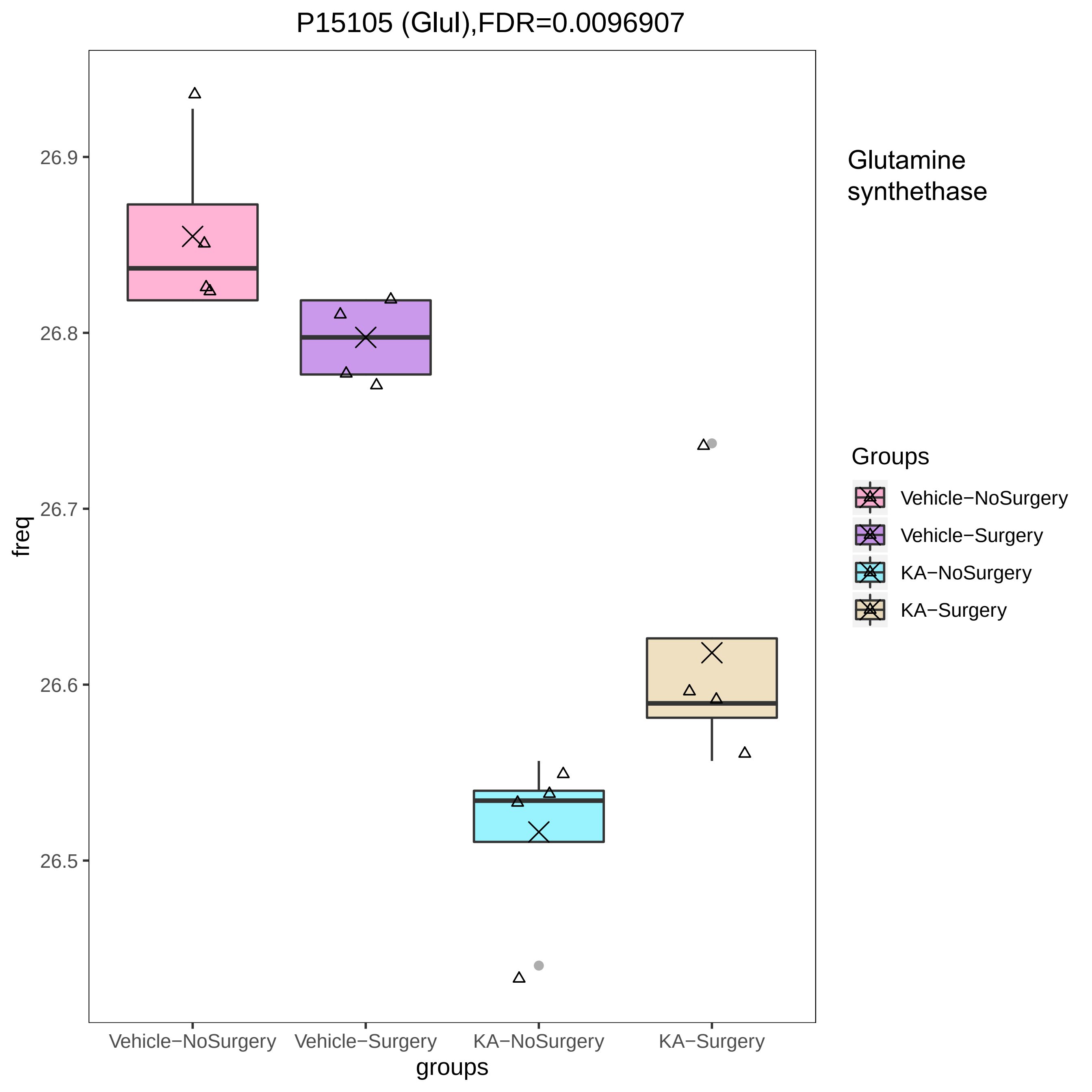

P15105 (Glul),FDR=0.0096907
Glutamine
synthethase
26.9
26.8
26.7
26.6
26.5
freq
Vehicle−NoSurgery
Vehicle−Surgery
KA−NoSurgery
KA−Surgery
groups
Groups
Vehicle−NoSurgery
Vehicle−Surgery
KA−NoSurgery
KA−Surgery

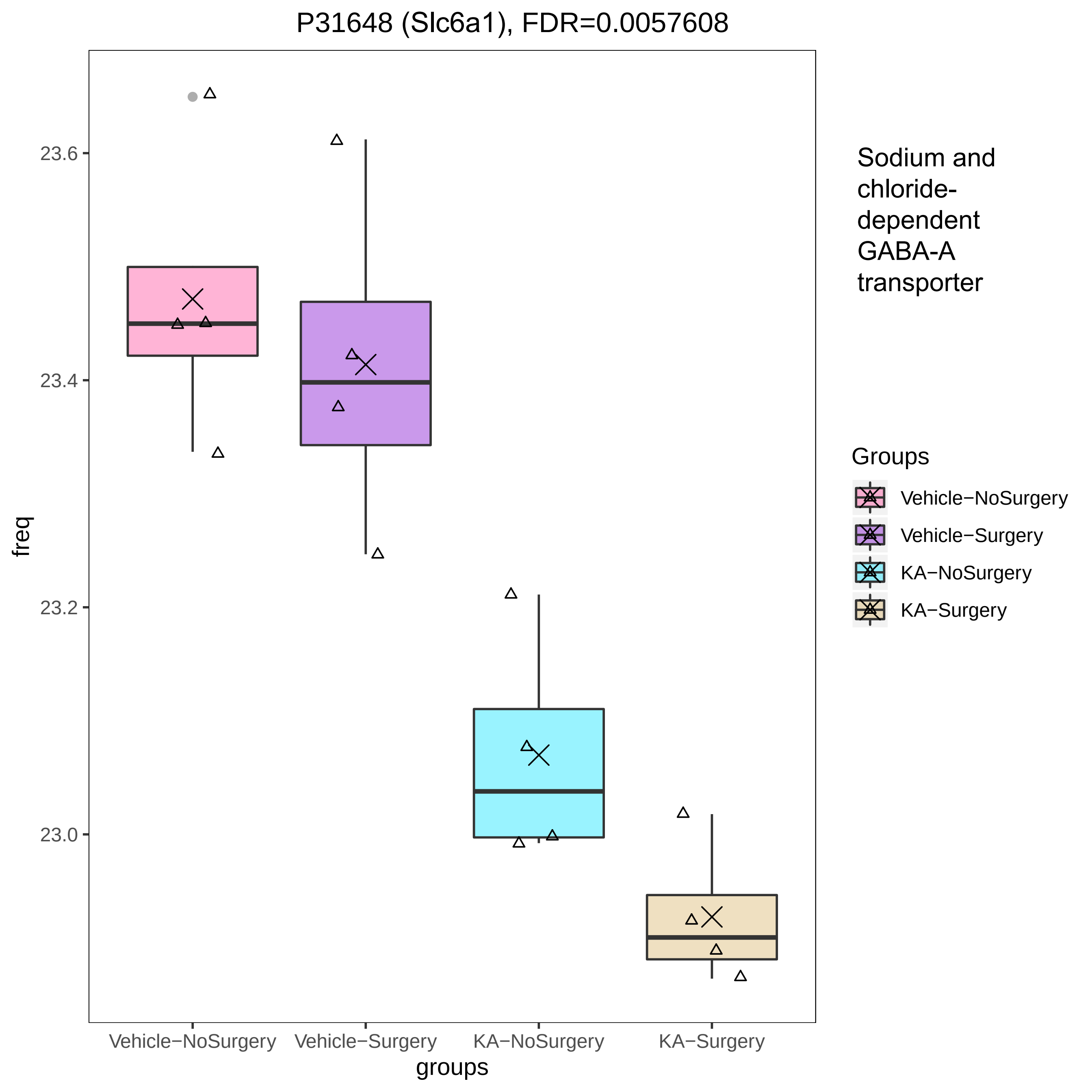
P31648 (Slc6a1), FDR=0.0057608
freq
23.6
23.4
23.2
23.0
Vehicle−NoSurgery
Vehicle−Surgery
KA−NoSurgery
KA−Surgery
groups
Sodium and chloride-dependent GABA-A transporter
Groups
Vehicle−NoSurgery
Vehicle−Surgery
KA−NoSurgery
KA−Surgery

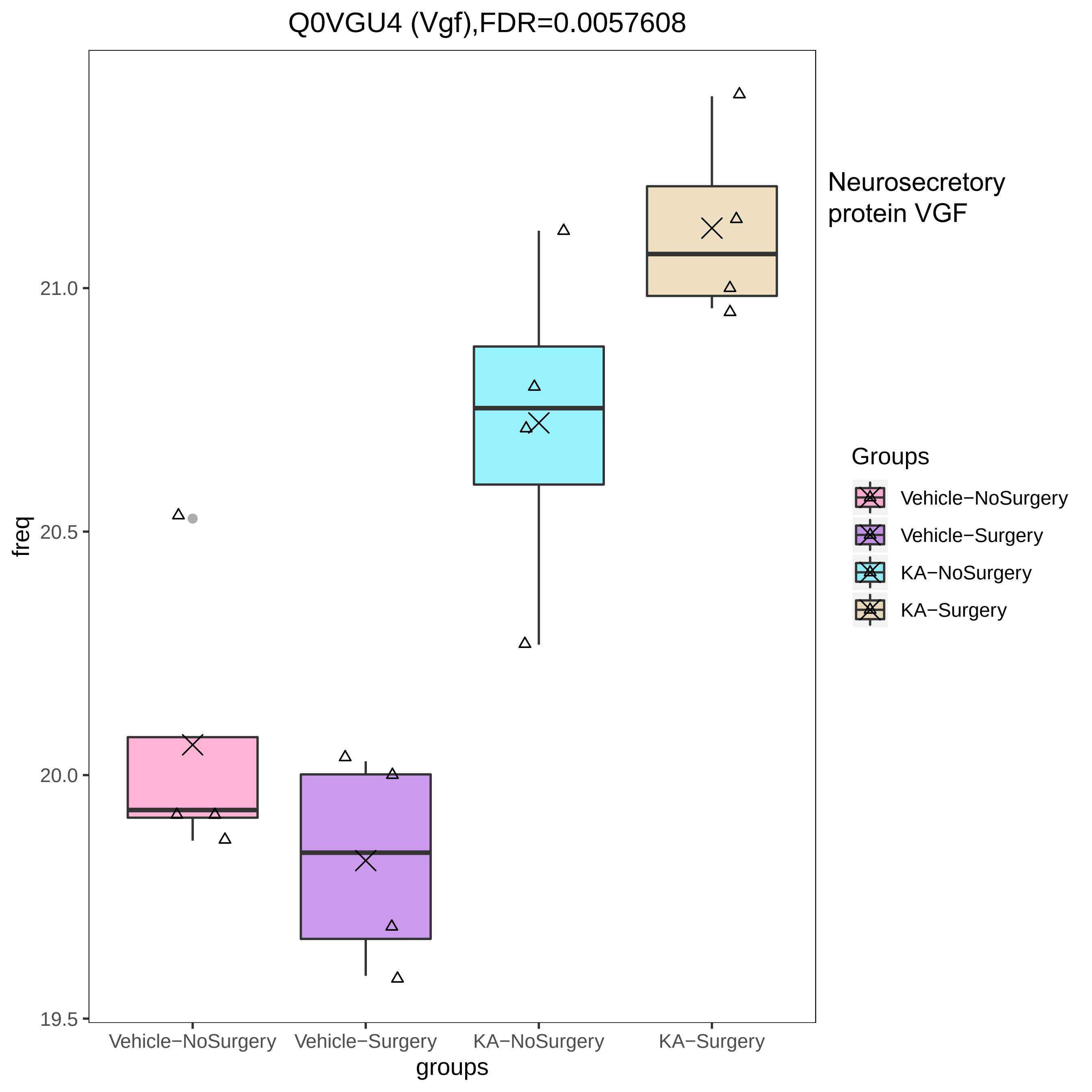
Q0VGU4 (Vgf),FDR=0.0057608
Neurosecretory protein VGF
freq
21.0
20.5
20.0
19.5
Vehicle−NoSurgery
Vehicle−Surgery
KA−NoSurgery
KA−Surgery
groups
Groups
Vehicle−NoSurgery
Vehicle−Surgery
KA−NoSurgery
KA−Surgery

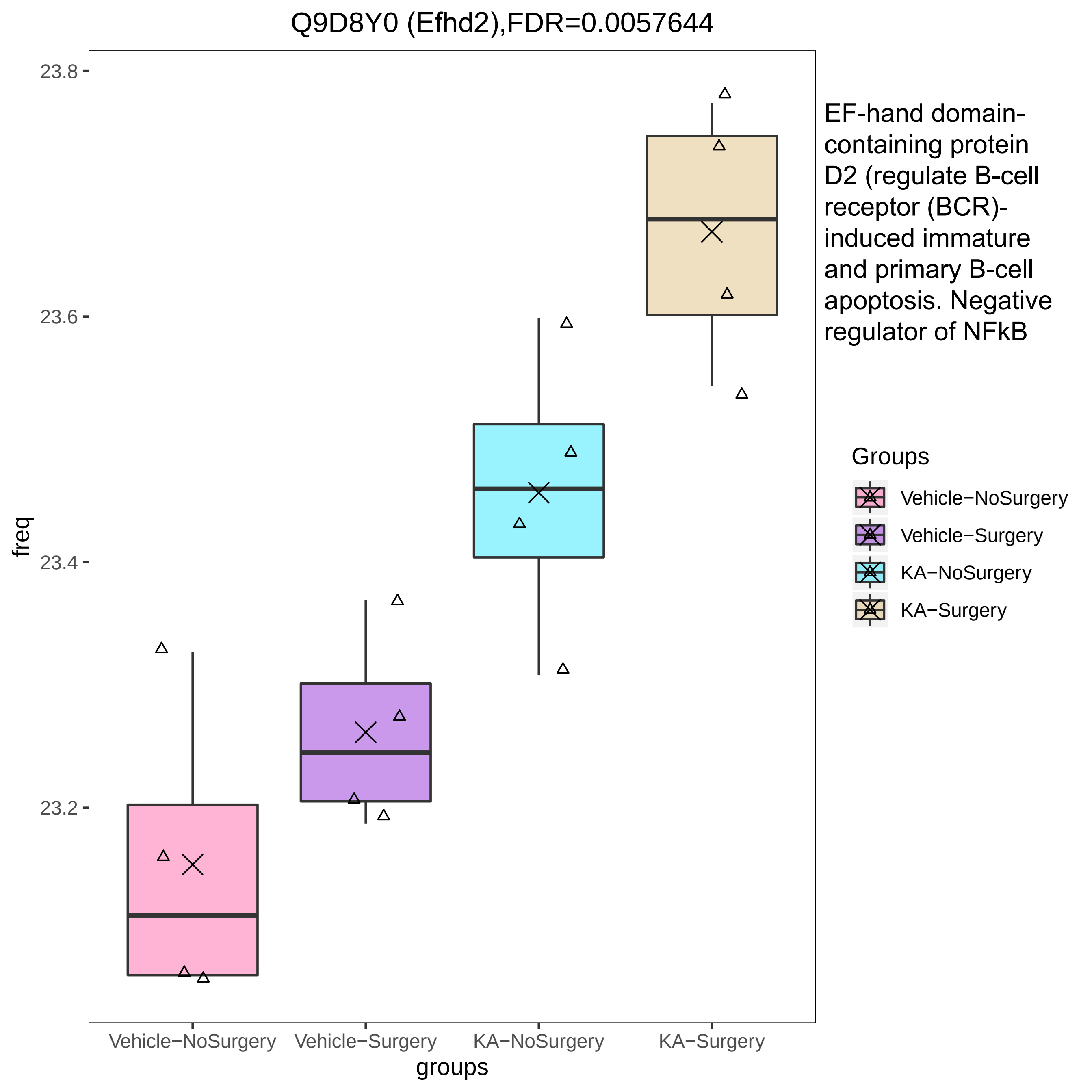
Q9D8Y0 (Efhd2),FDR=0.0057644
23.8
23.6
23.4
23.2
freq
Vehicle−NoSurgery
Vehicle−Surgery
KA−NoSurgery
KA−Surgery
groups
EF-hand domain-containing protein D2 (regulate B-cell receptor (BCR)-induced immature and primary B-cell apoptosis. Negative regulator of NFkB
Groups
Vehicle−NoSurgery
Vehicle−Surgery
KA−NoSurgery
KA−Surgery

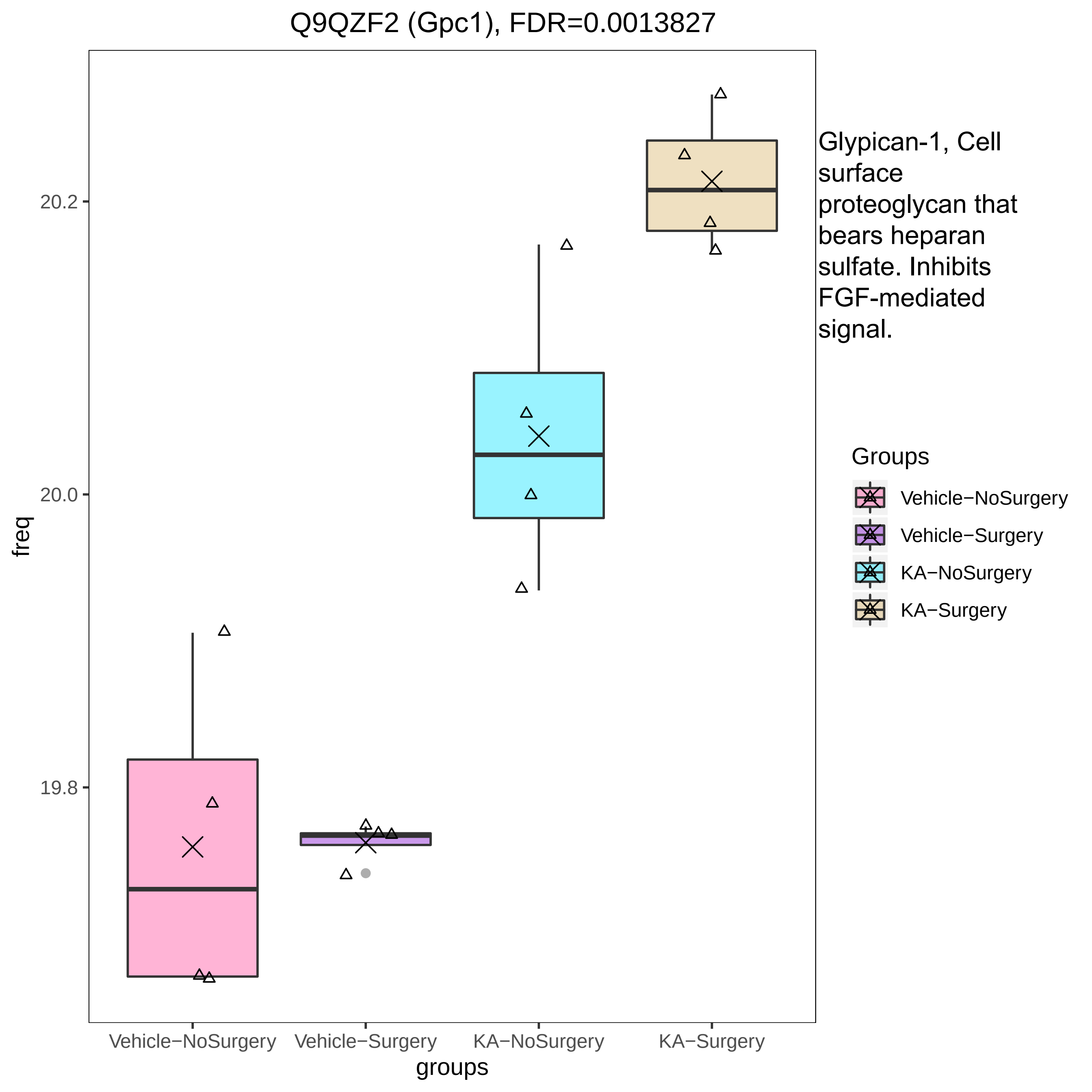
Q9QZF2 (Gpc1), FDR=0.0013827
Glypican-1, Cell surface proteoglycan that bears heparan sulfate. Inhibits FGF-mediated signal.
freq
20.2
20.0
19.8
groups
Vehicle−NoSurgery
Vehicle−Surgery
KA−NoSurgery
KA−Surgery
Groups
Vehicle−NoSurgery
Vehicle−Surgery
KA−NoSurgery
KA−Surgery

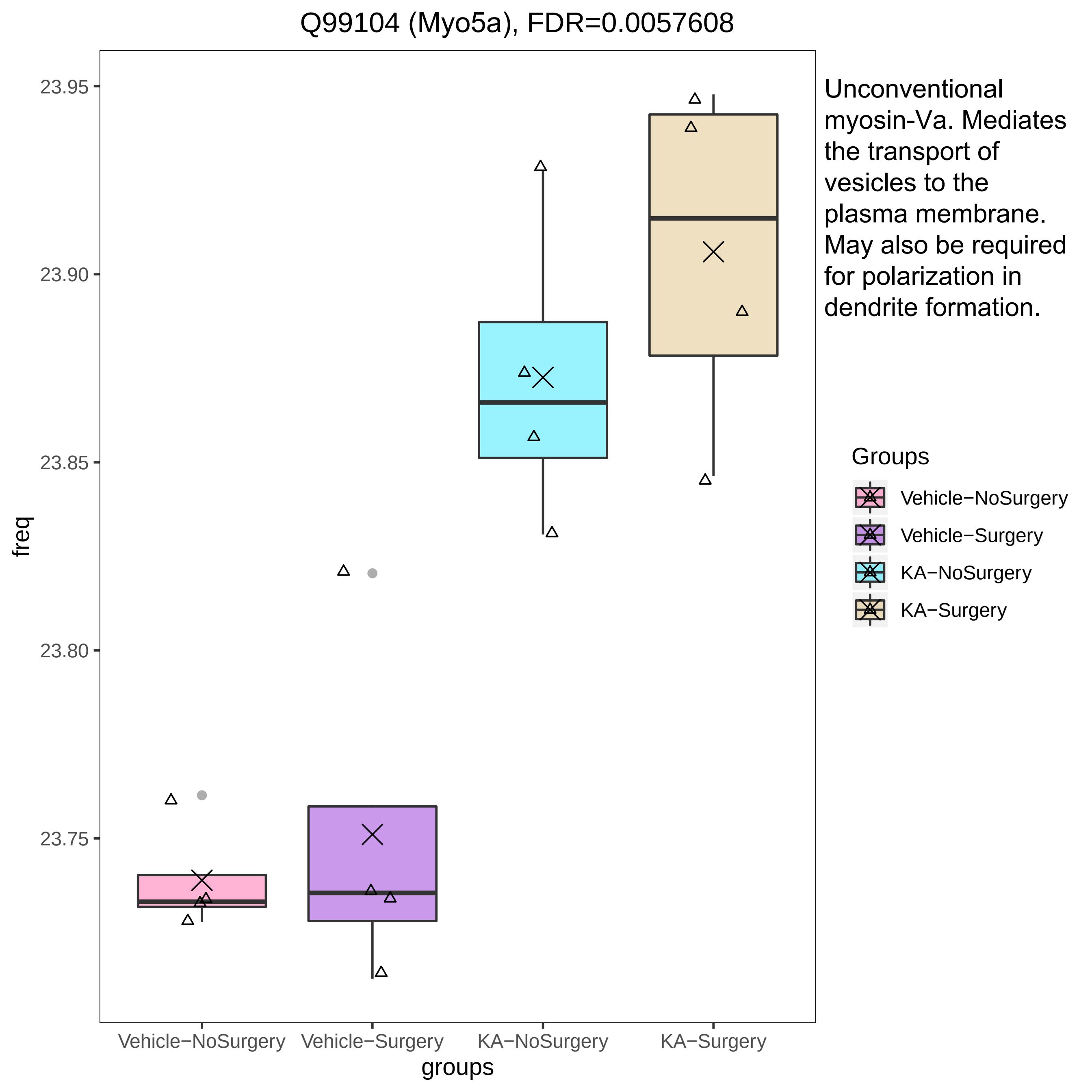
Q99104 (Myo5a), FDR=0.0057608
23.95
23.90
23.85
23.80
23.75
freq
Vehicle−NoSurgery
Vehicle−Surgery
KA−NoSurgery
KA−Surgery
groups
Unconventional myosin-Va. Mediates the transport of vesicles to the plasma membrane. May also be required for polarization in dendrite formation.
Groups
Vehicle−NoSurgery
Vehicle−Surgery
KA−NoSurgery
KA−Surgery

Uniprot ID	Protein names	Gene names	f.value	p.value	neg log10(p)	FDR	Abundance on log scale			
							Veh-NoSurg	Veh-Surg	KA-NoSurg	KA-Surgery
**Protein upregulated in both surgery groups (vehicle and KA)**										
Q8VHL1	Histone-lysine N-methyltransferase SETD7 (SET7/9)	Setd7 Set7 Set9	66.863	9.25E-08	7.0337	0.00014	21.23233	21.911073	21.15768	21.993103
Q9D0M5	Dynein light chain 2, cytoplasmic (DLC8)	Dynll2 Dlc2	58.2	2.02E-07	6.6954	0.00015	22.21056	21.12811	22.18282	21.00142
Q6A026	Sister chromatid cohesion protein PDS5 A	Pds5a Kiaa0648	36.452	2.62E-06	5.5812	0.00134	19.8724	20.885968	19.80909	20.84635
P03995	Glial fibrillary acidic protein (GFAP)	Gfap	31.792	5.43E-06	5.265	0.00138	25.55244	27.025413	26.87475	27.968345
Q8CIG9	F-box/LRR-repeat protein 8 (F-box protein FBL8)	Fbxl8 Fbl8	32.684	4.69E-06	5.3286	0.00138	19.18971	20.350538	18.93956	20.272563
Q6PB66	Leucine-rich PPR motif-containing protein, mitochondrial (LRP 130)	Lrpprc Lrp130	29.347	8.27E-06	5.0823	0.0018	21.12106	21.556105	21.02334	21.506653
Q3URK3	Methylcytosine dioxygenase TET1 (CXXC-type zinc finger protein 6)	Tet1 Cxxc6 Kiaa1676	27.562	1.15E-05	4.9404	0.00181	19.20219	20.01175	18.97767	19.870405
P55012	Solute carrier family 12 member 2 (Basolateral Na-K-Cl symporter)	Slc12a2 Nkcc1	27.452	1.17E-05	4.9313	0.00181	21.57142	22.651055	21.59296	22.838318
P10605	Cathepsin B/B1)	Ctsb	15.512	0.000198	3.7042	0.00868	21.24294	21.535165	21.29265	21.835295
P14602	Heat shock protein beta-1 (HspB1/ HSP 25)	Hspb1 Hsp25/27	26.34	1.45E-05	4.8385	0.00191	18.34595	18.024863	20.10053	21.608808
Q5SSL4	Active breakpoint cluster region-related protein	Abr	24.9	1.94E-05	4.7132	0.00191	21.39741	22.032938	21.47865	22.021938
P16045	Galectin-1 (Gal-1/Galaptin)	Lgals1 Gbp	23.294	2.72E-05	4.566	0.00244	18.55418	19.299368	19.29811	20.263458
Q9WVA3	Mitotic checkpoint protein BUB3	Bub3	22.904	2.96E-05	4.5289	0.00251	19.26223	19.585013	19.20765	19.39816
Q02105	Complement C1qc	C1qc C1qg	16.908	0.000131	3.8812	0.00633	18.60421	20.583218	19.43826	20.605043
P20152	Vimentin	Vim	20.7	4.91E-05	4.3091	0.00361	23.70164	25.16986	24.87103	26.487395
P62301	40S ribosomal protein S13	Rps13	20.655	4.96E-05	4.3044	0.00361	21.70742	21.852868	21.85315	21.976885
Q9CQJ6	Density-regulated protein (DRP)	Denr	19.443	6.69E-05	4.1749	0.00464	19.67067	20.136073	19.63894	20.130675
P02468	Laminin subunit gamma-1 (Laminin B2 chain)	Lamc1 Lamb-2	18.078	9.53E-05	4.0209	0.00576	18.75928	19.305835	18.70097	19.563578
P21460	Cystatin-C (Cystatin-3)	Cst3	15.395	0.000205	3.6888	0.00869	21.29772	21.565828	21.74042	22.158848
P98086	Complement C1qa	C1qa	15.099	0.000224	3.6494	0.00901	17.52368	19.70244	18.78533	19.755233
**Proteins downregulated in both surgery groups (vehicle and KA)**										
P32037	Solute carrier family 2-(Glucose transporter type 3, brain) (GLUT-3)	Slc2a3 Glut3	24.736	0.00002	4.6986	0.00191	22.52552	22.390868	22.63737	22.245415
Q62283	Tetraspanin-7 (Tspan-7/CD antigen CD231)	Tspan7 Mxs1	21.247	4.31E-05	4.3654	0.00347	19.89033	19.44267	19.63764	19.222073
Q8C0E2	Vacuolar protein sorting-associated protein 26B	Vps26b	27.386	1.19E-05	4.926	0.00181	21.77001	21.464778	21.70237	21.46987
Q8CBW3	Abl interactor 1 (Abelson interactor 1) (Abi-1)	Abi1 Ssh3bp1	18.428	8.69E-05	4.0612	0.00553	21.26101	20.738385	21.16048	20.689868
P35279	Ras-related protein Rab-6A (Rab-6)	Rab6a Rab6	18.567	8.37E-05	4.077	0.00553	21.63157	21.371815	21.51557	21.370305
P62071	Ras-related protein R-Ras2	Rras2	17.853	0.000101	3.9946	0.00576	20.50515	20.053165	20.35101	19.987185
P31648	Voltage-dependent GABA transporter 1 (GAT-1)	Slc6a1 Gat-1	17.805	0.000103	3.9889	0.00576	23.44462	23.413773	23.06983	22.927308
Q9D8Y0	EF-hand domain-containing protein D2	Efhd2 Sws1	17.443	0.000113	3.946	0.00576	23.16364	23.261423	23.45649	23.668985
P39053	Dynamin-1 (EC 3.6.5.5)	Dnm1	16.874	0.000133	3.8771	0.00633	27.24975	27.13156	27.22933	26.941998
Q9QYB8	Beta-adducin (Add97)	Add2	15.586	0.000193	3.7139	0.00868	23.79847	23.675545	23.77589	23.580043
Q62188	Dihydropyrimidinase-related protein 3 (DRP-3)	Dpysl3 Drp3 Ulip	15.49	0.000199	3.7014	0.00868	24.70239	24.31222	24.70526	24.134963
P61161	Actin-related protein 2 (Actin-like protein 2)	Actr2 Arp2	15.215	0.000216	3.665	0.00893	24.06893	23.887608	24.00868	23.72985
P15105	Glutamine synthetase	Glul Glns	14.75	0.00025	3.6022	0.00969	26.8151	26.797415	26.51625	26.618135

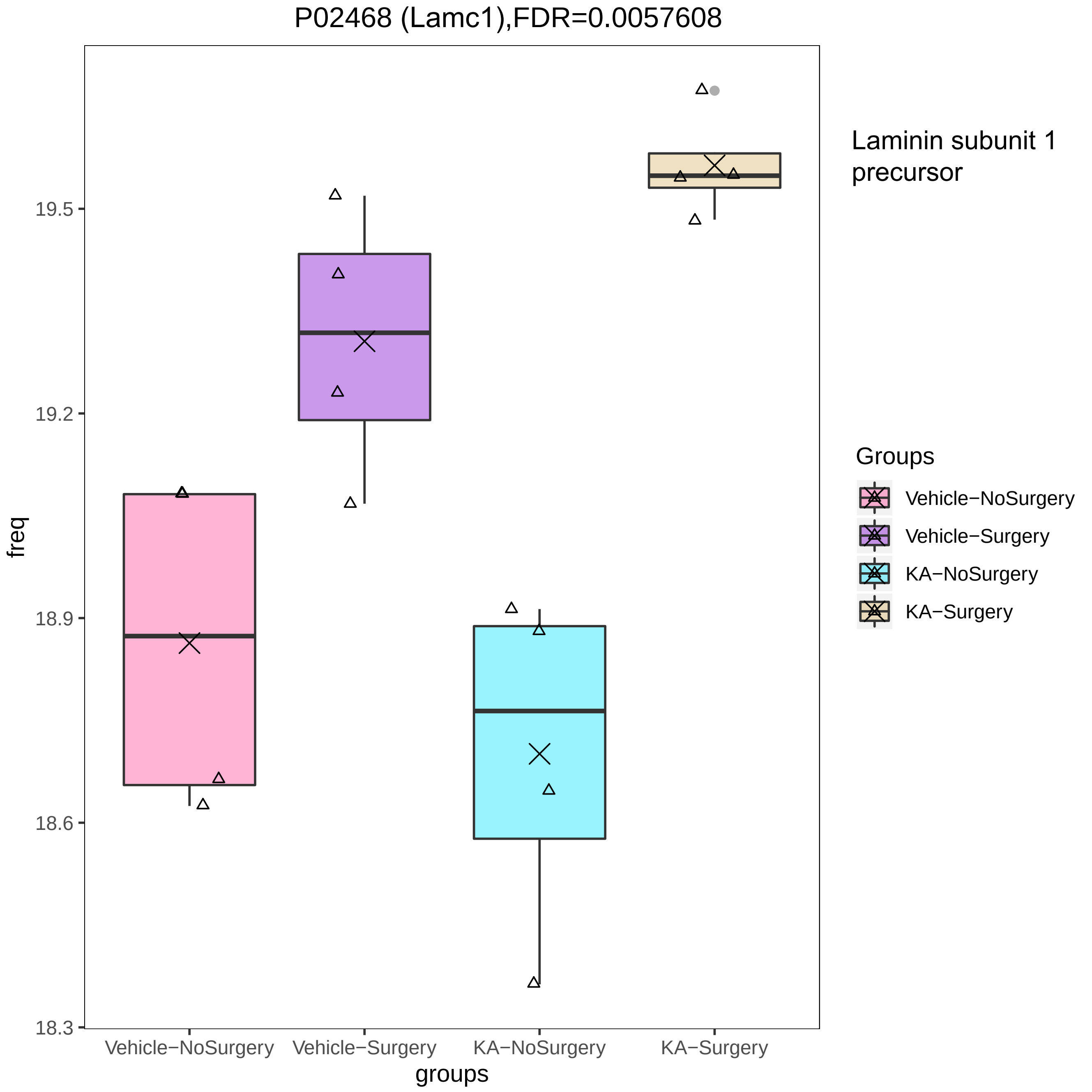

P02468 (Lamc1),FDR=0.0057608
Laminin subunit 1 precursor
Groups
Vehicle−NoSurgery
Vehicle−Surgery
KA−NoSurgery
KA−Surgery
freq
19.5
19.2
18.9
18.6
18.3
Vehicle−NoSurgery
Vehicle−Surgery
KA−NoSurgery
KA−Surgery
groups

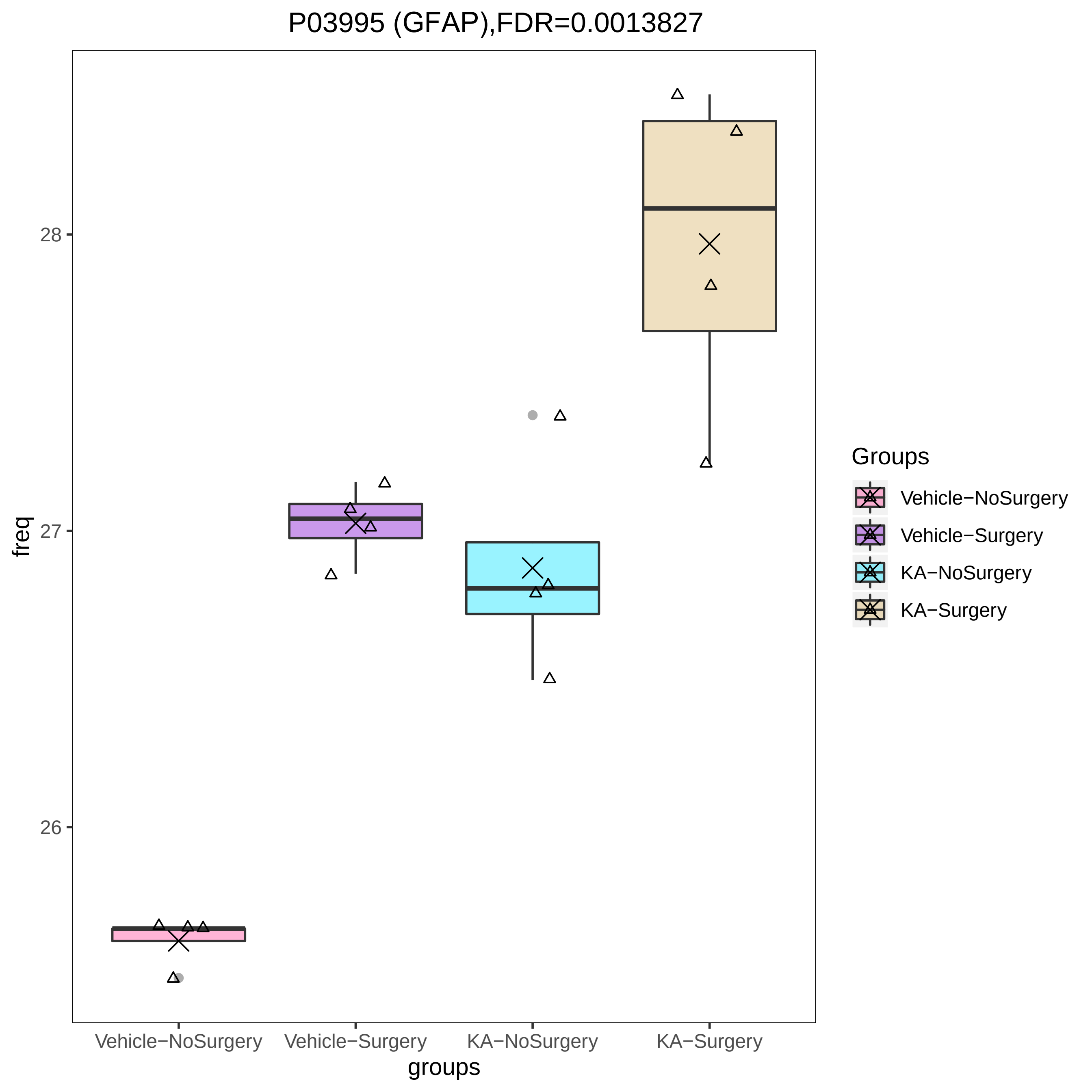
P03995 (GFAP),FDR=0.0013827
freq
28
27
26
groups
Vehicle−NoSurgery
Vehicle−Surgery
KA−NoSurgery
KA−Surgery
Groups
Vehicle−NoSurgery
Vehicle−Surgery
KA−NoSurgery
KA−Surgery

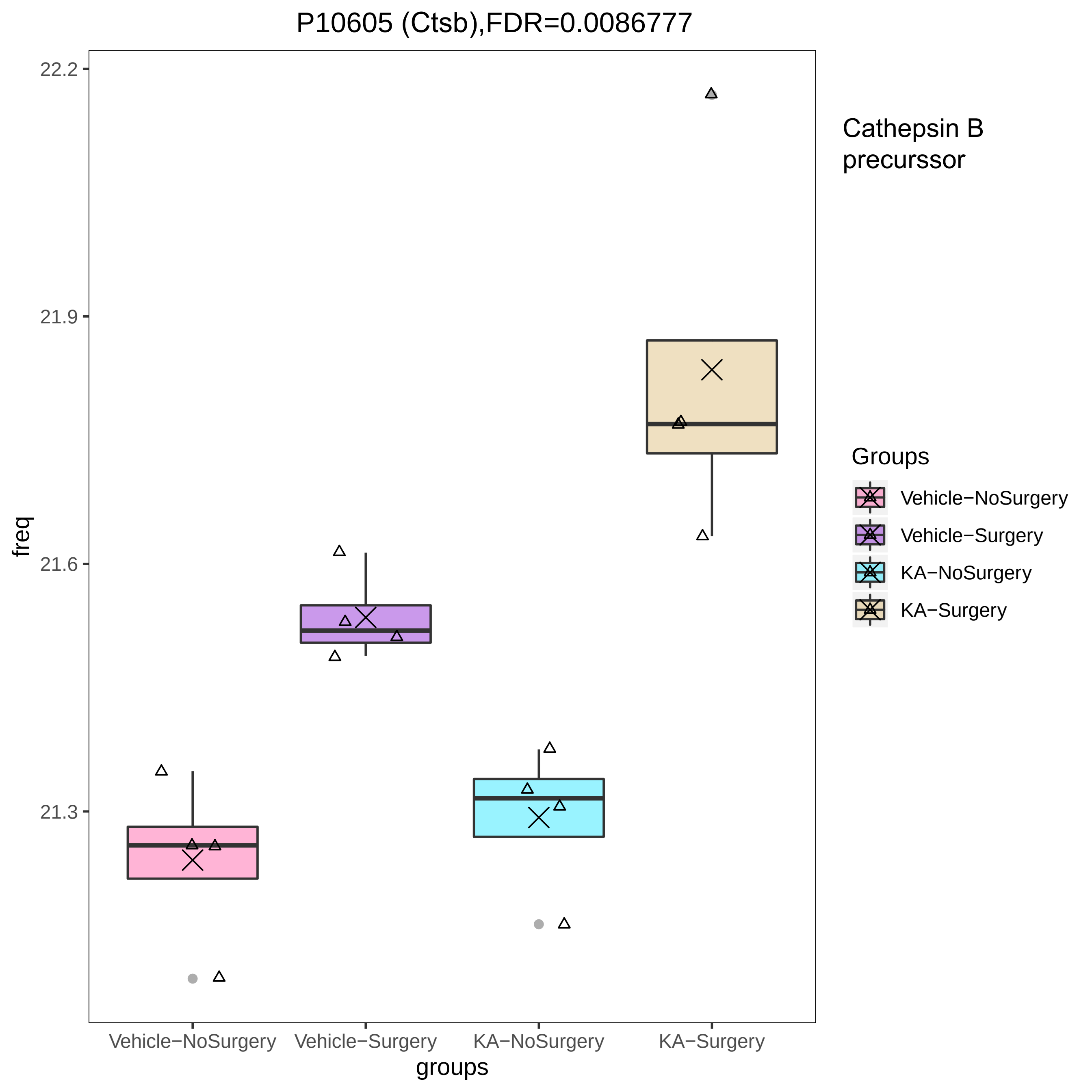

P10605 (Ctsb),FDR=0.0086777
Cathepsin B
precurssor
22.2
21.9
21.6
21.3
freq
Vehicle−NoSurgery
Vehicle−Surgery
KA−NoSurgery
KA−Surgery
groups
Groups
Vehicle−NoSurgery
Vehicle−Surgery
KA−NoSurgery
KA−Surgery

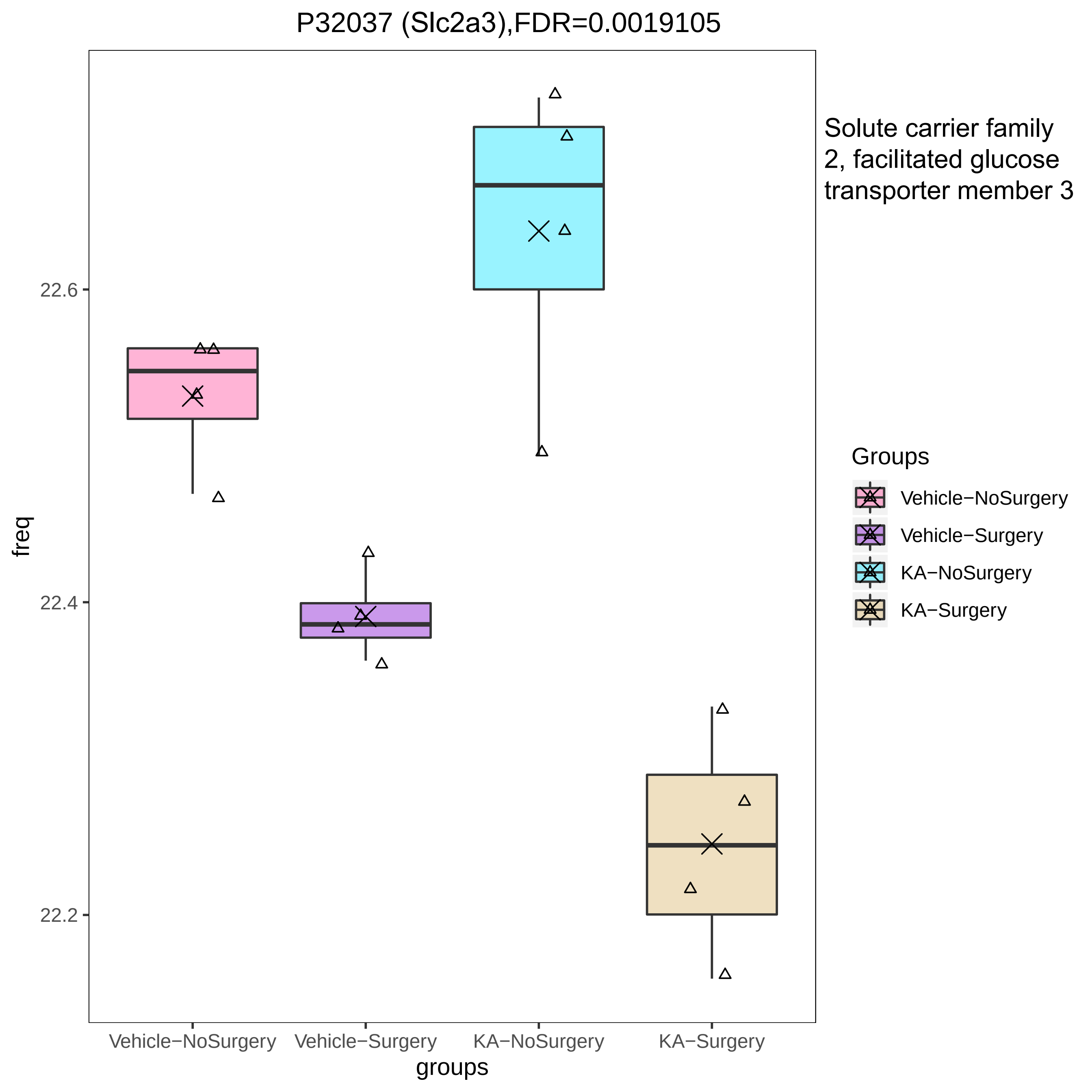

P32037 (Slc2a3),FDR=0.0019105
Solute carrier family 2, facilitated glucose transporter member 3
freq
22.6
22.4
22.2
Vehicle−NoSurgery
Vehicle−Surgery
KA−NoSurgery
KA−Surgery
groups
Groups
Vehicle−NoSurgery
Vehicle−Surgery
KA−NoSurgery
KA−Surgery

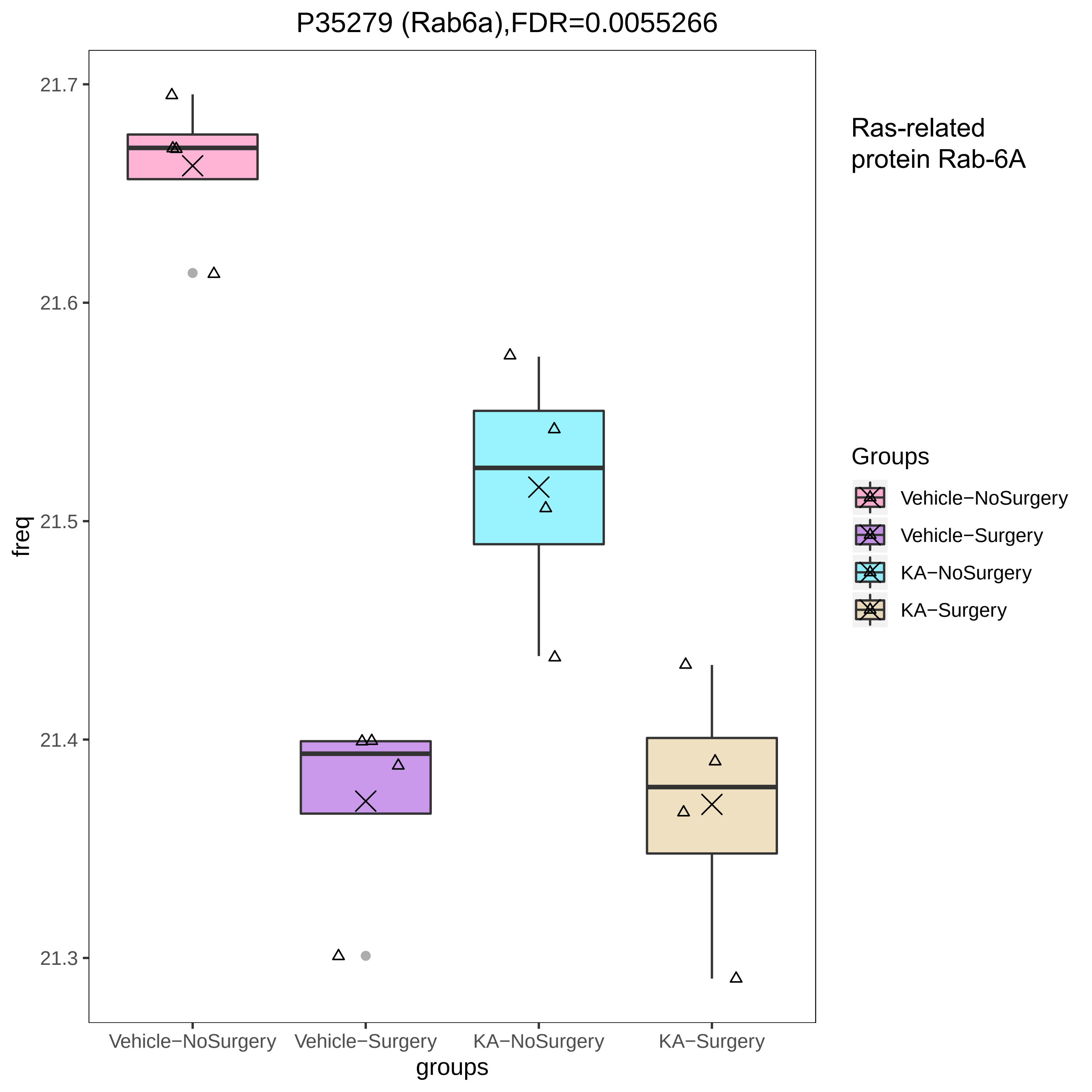

P35279 (Rab6a),FDR=0.0055266
Ras-related protein Rab-6A
freq
21.7
21.6
21.5
21.4
21.3
groups
Vehicle−NoSurgery
Vehicle−Surgery
KA−NoSurgery
KA−Surgery
Groups
Vehicle−NoSurgery
Vehicle−Surgery
KA−NoSurgery
KA−Surgery

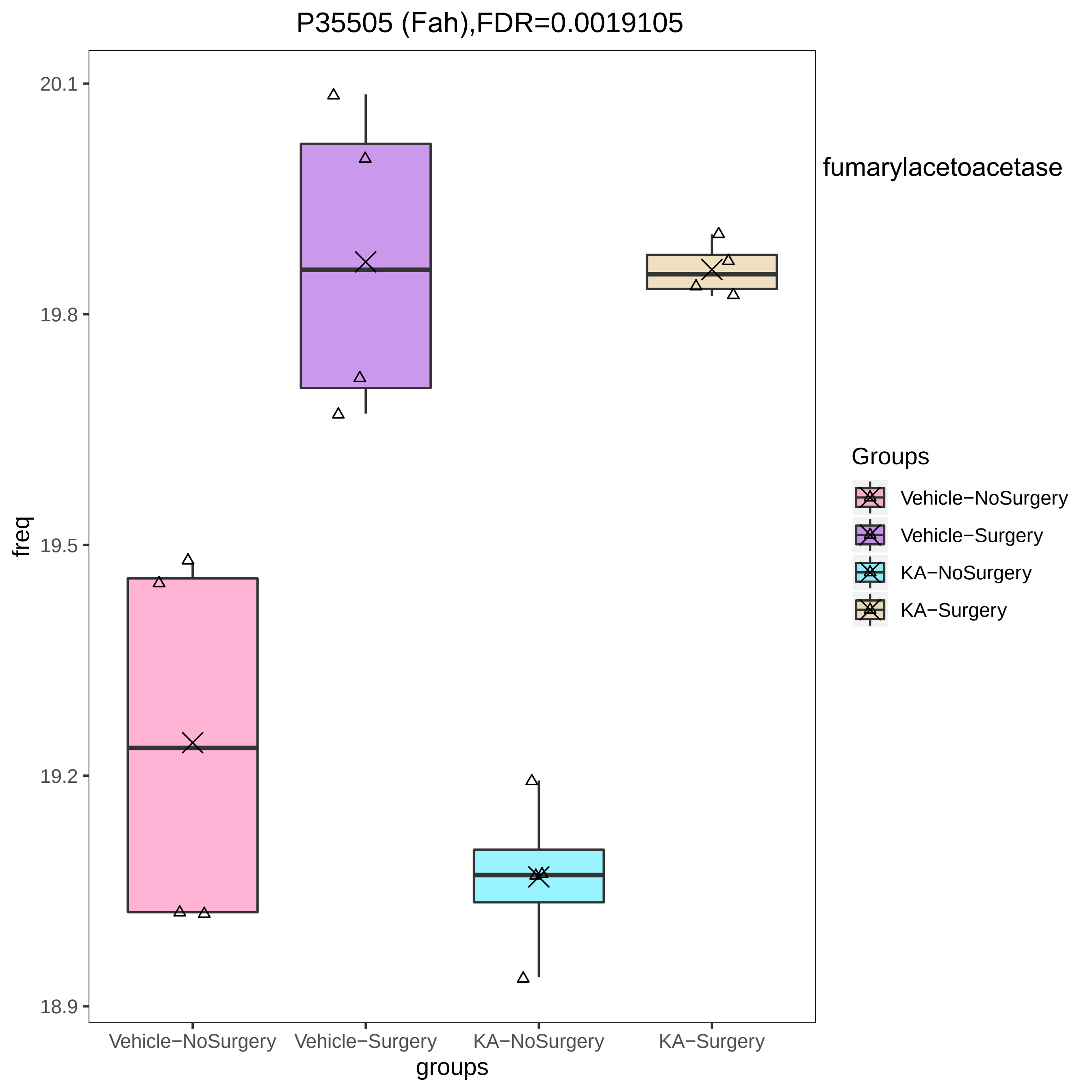

P35505 (Fah),FDR=0.0019105
fumarylacetoacetase
freq
20.1
19.8
19.5
19.2
18.9
Vehicle−NoSurgery
Vehicle−Surgery
KA−NoSurgery
KA−Surgery
groups
Groups
Vehicle−NoSurgery
Vehicle−Surgery
KA−NoSurgery
KA−Surgery

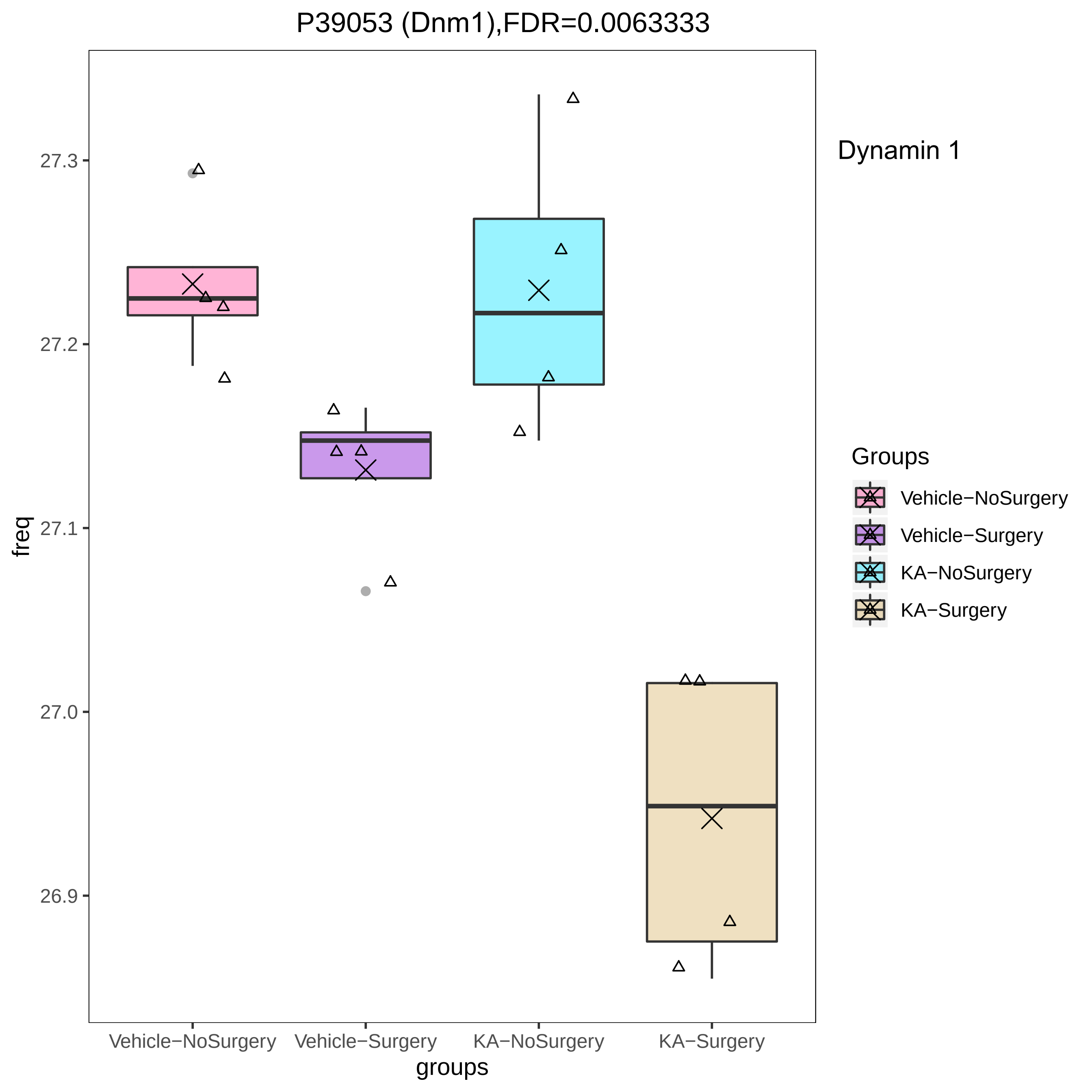

P39053 (Dnm1),FDR=0.0063333
Dynamin 1
freq
27.3
27.2
27.1
27.0
26.9
Vehicle−NoSurgery
Vehicle−Surgery
KA−NoSurgery
KA−Surgery
groups
Groups
Vehicle−NoSurgery
Vehicle−Surgery
KA−NoSurgery
KA−Surgery

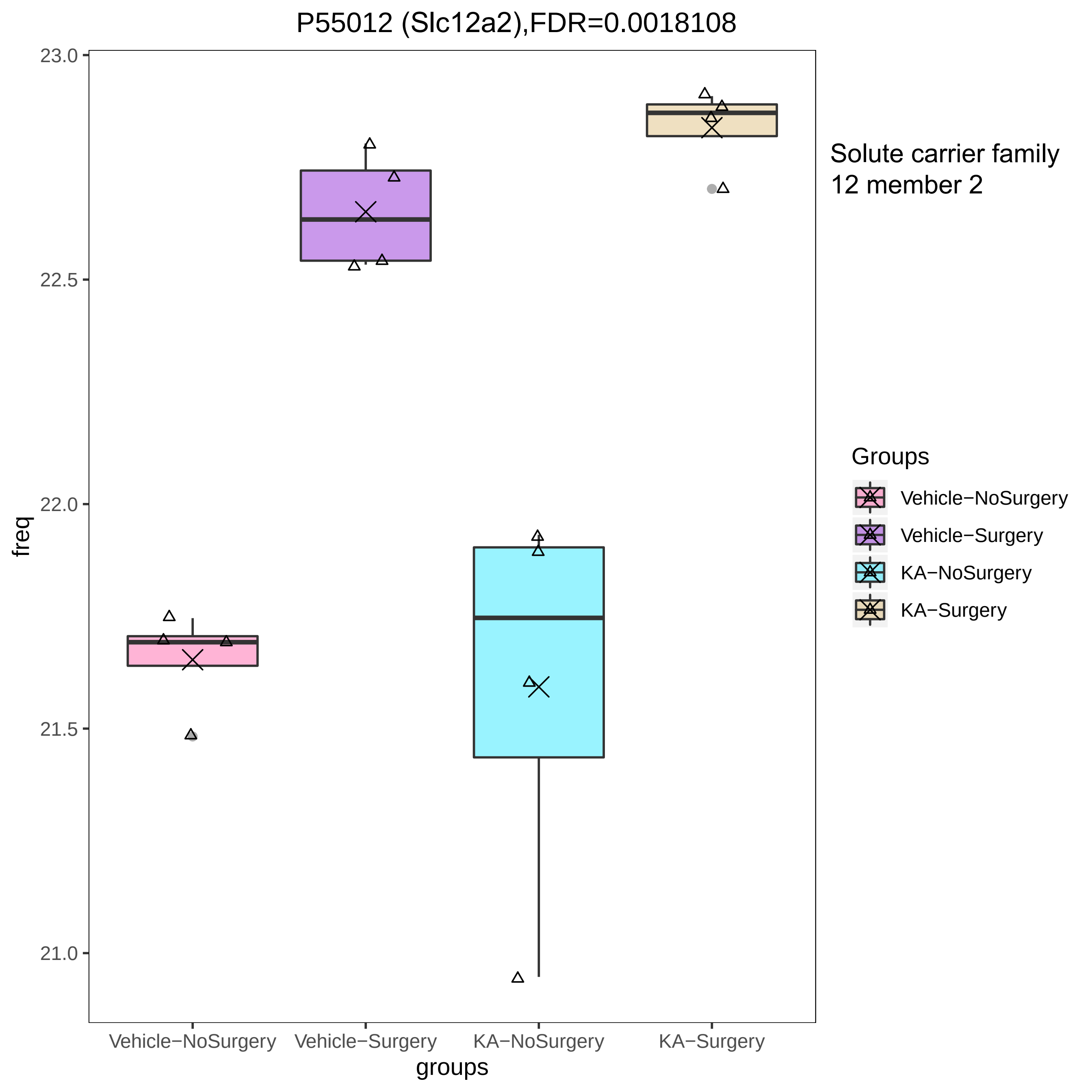
P55012 (Slc12a2),FDR=0.0018108
Solute carrier family
12 member 2
freq
groups
23.0
22.5
22.0
21.5
21.0
Vehicle−NoSurgery
Vehicle−Surgery
KA−NoSurgery
KA−Surgery
Groups
Vehicle−NoSurgery
Vehicle−Surgery
KA−NoSurgery
KA−Surgery

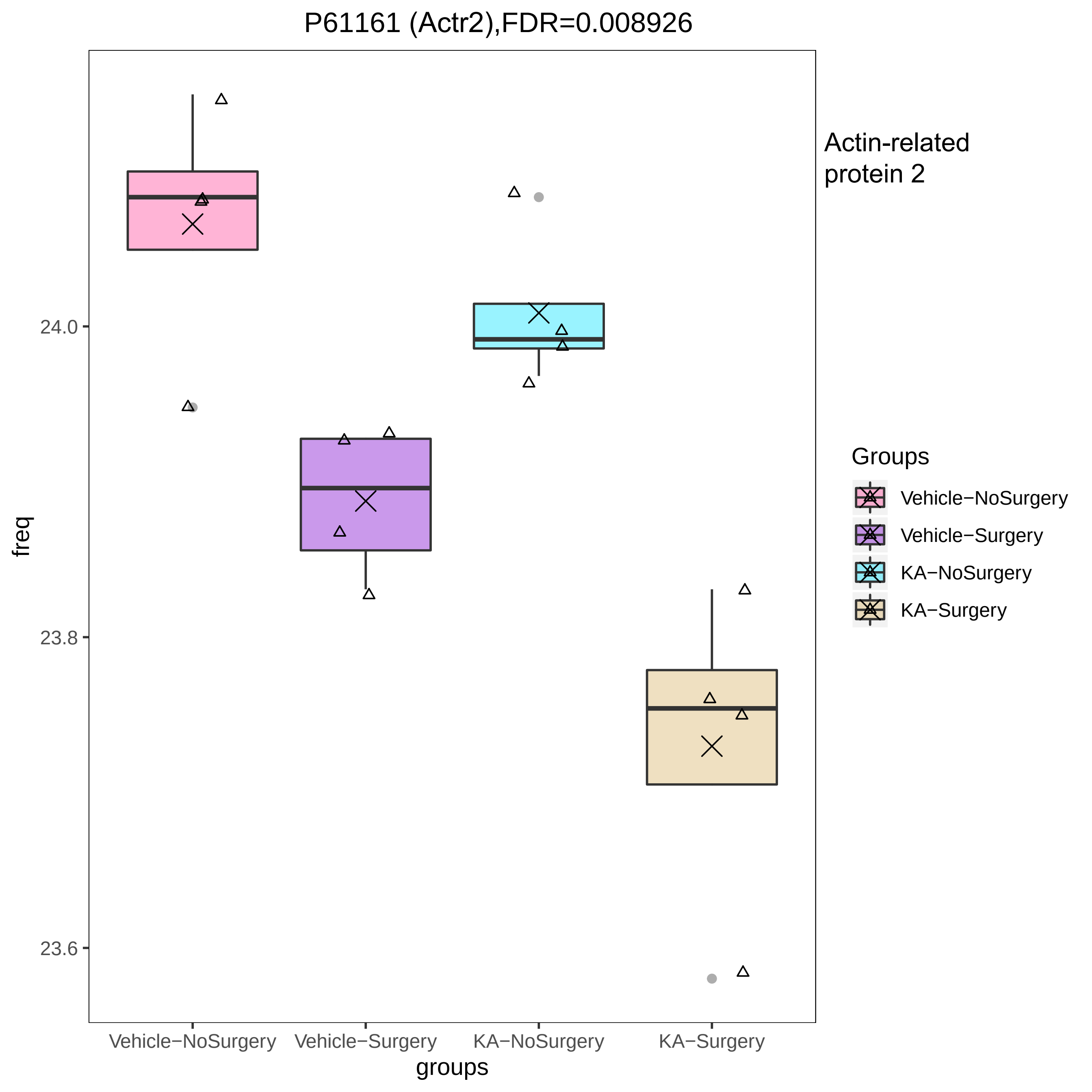

P61161 (Actr2),FDR=0.008926
Actin-related protein 2
freq
24.0
23.8
23.6
Vehicle−NoSurgery
Vehicle−Surgery
KA−NoSurgery
KA−Surgery
groups
Groups
Vehicle−NoSurgery
Vehicle−Surgery
KA−NoSurgery
KA−Surgery

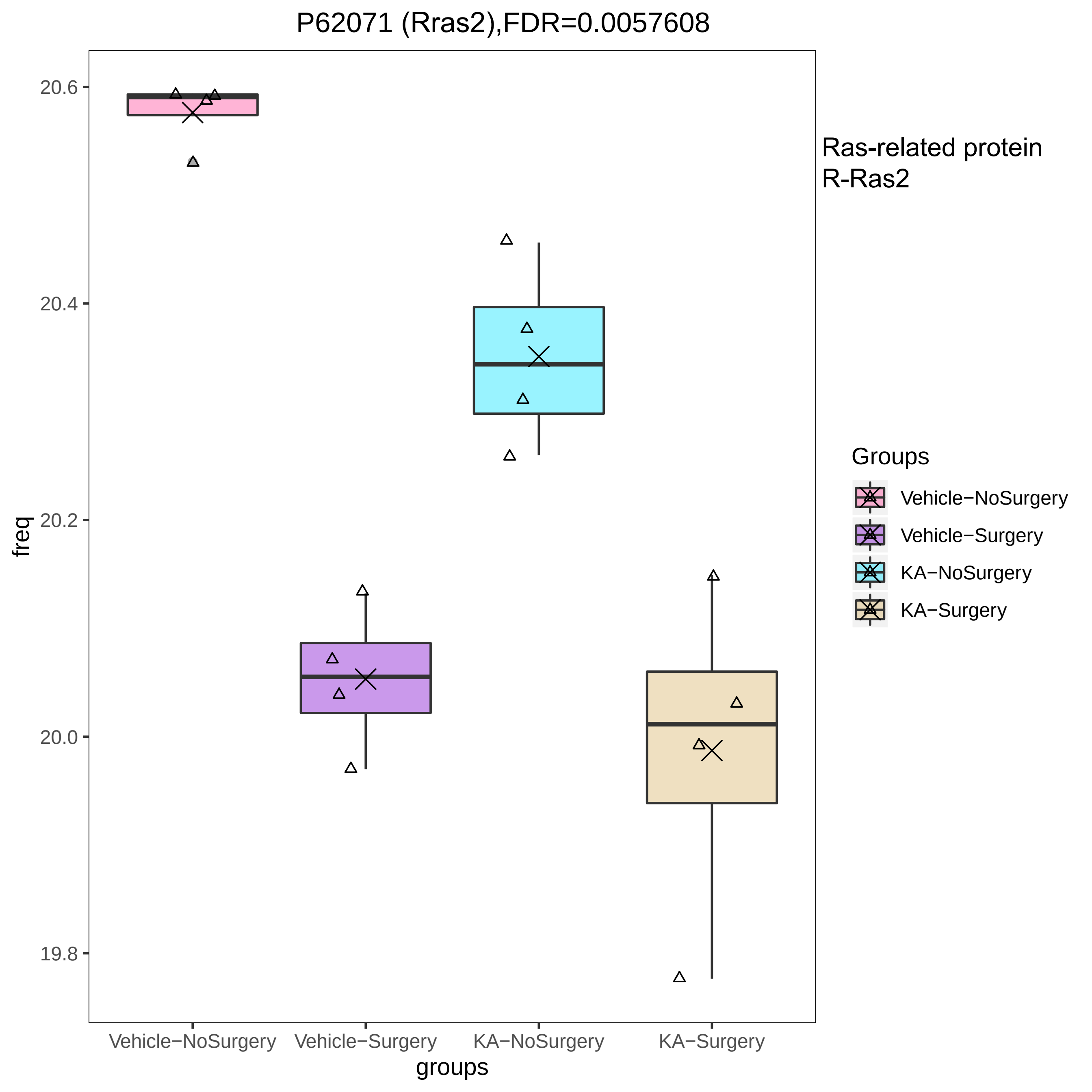

P62071 (Rras2),FDR=0.0057608
Ras-related protein
R-Ras2
freq
20.6
20.4
20.2
20.0
19.8
Vehicle−NoSurgery
Vehicle−Surgery
KA−NoSurgery
KA−Surgery
groups
Groups
Vehicle−NoSurgery
Vehicle−Surgery
KA−NoSurgery
KA−Surgery

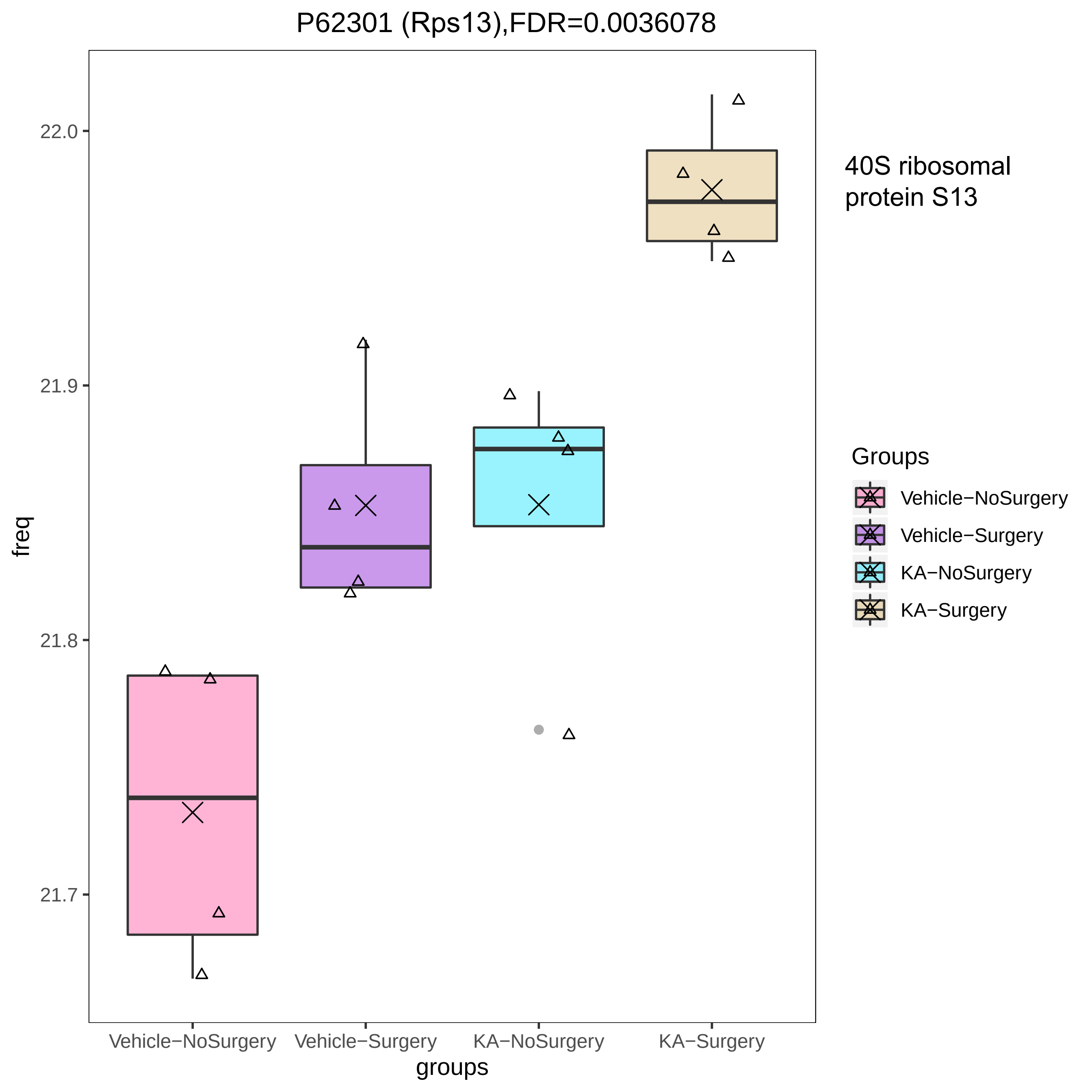
P62301 (Rps13),FDR=0.0036078
freq
22.0
21.9
21.8
21.7
groups
Vehicle−NoSurgery
Vehicle−Surgery
KA−NoSurgery
KA−Surgery
40S ribosomal protein S13
Groups
Vehicle−NoSurgery
Vehicle−Surgery
KA−NoSurgery
KA−Surgery

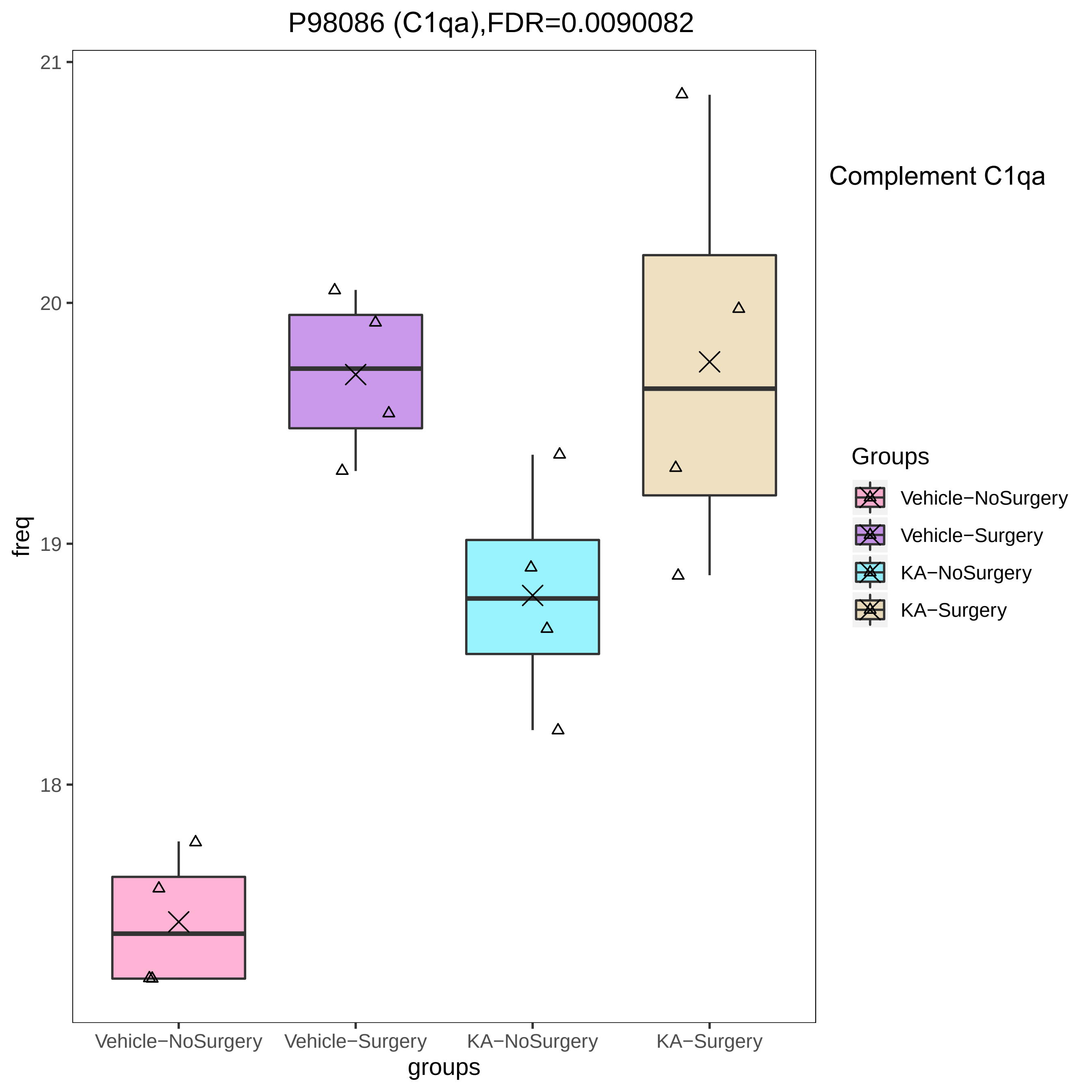
P98086 (C1qa),FDR=0.0090082
Complement C1qa
freq
21
20
19
18
Vehicle−NoSurgery
Vehicle−Surgery
KA−NoSurgery
KA−Surgery
groups
Groups
Vehicle−NoSurgery
Vehicle−Surgery
KA−NoSurgery
KA−Surgery

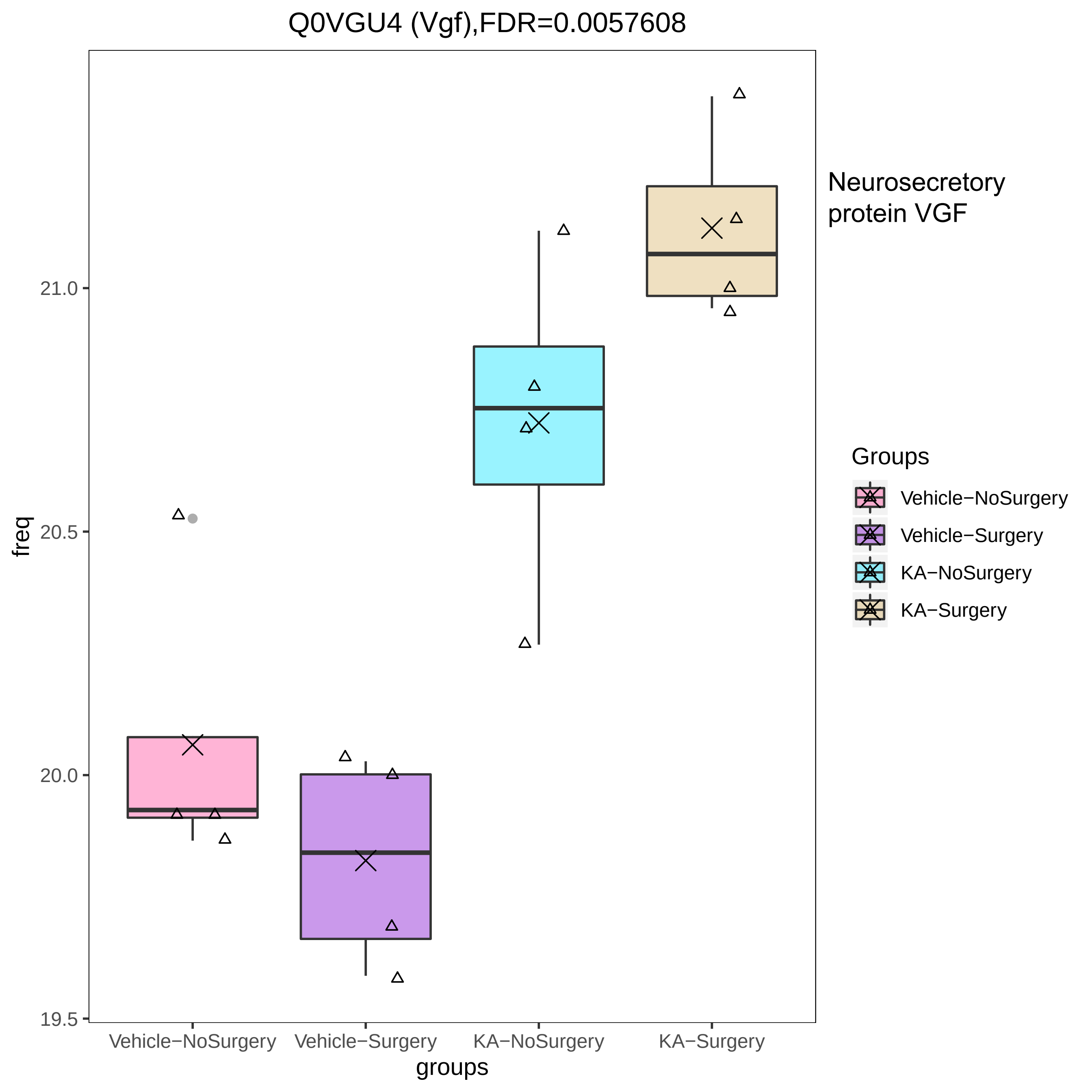

Q0VGU4 (Vgf),FDR=0.0057608
Neurosecretory protein VGF
freq
21.0
20.5
20.0
19.5
Vehicle−NoSurgery
Vehicle−Surgery
KA−NoSurgery
KA−Surgery
groups
Groups
Vehicle−NoSurgery
Vehicle−Surgery
KA−NoSurgery
KA−Surgery

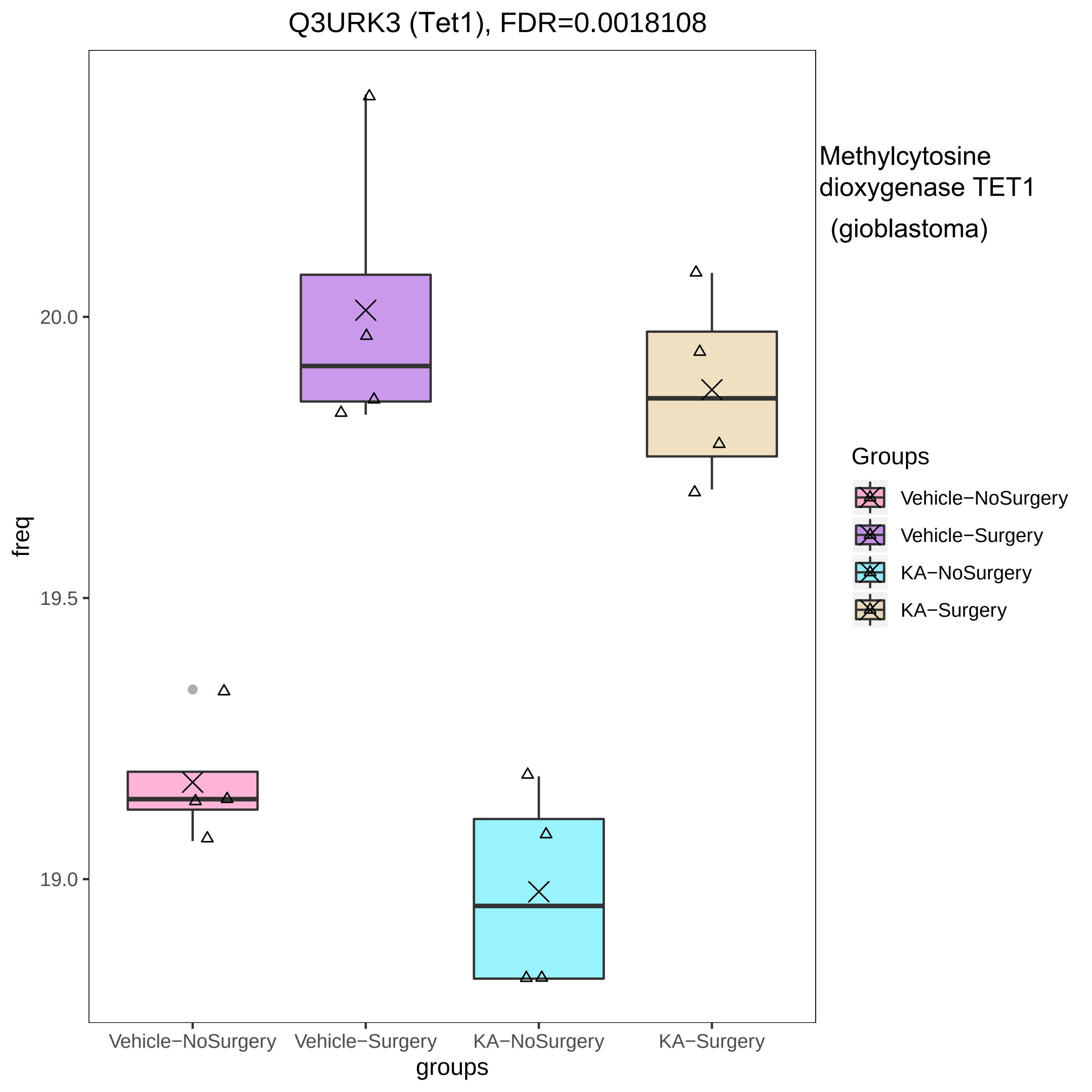
Q3URK3 (Tet1), FDR=0.0018108
Methylcytosine dioxygenase TET1 (gioblastoma)
freq
20.0
19.5
19.0
Vehicle−NoSurgery
Vehicle−Surgery
KA−NoSurgery
KA−Surgery
groups
Groups
Vehicle−NoSurgery
Vehicle−Surgery
KA−NoSurgery
KA−Surgery

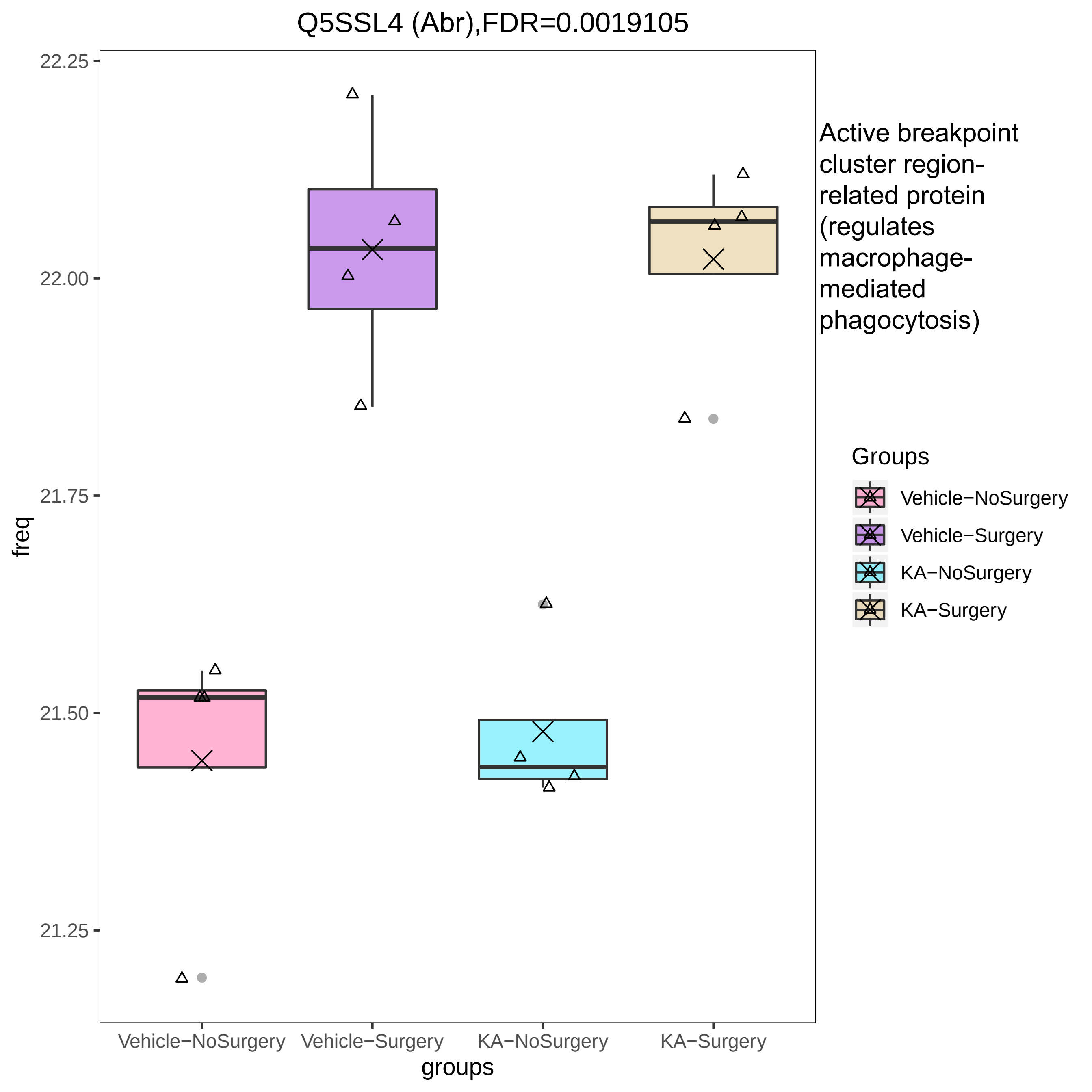

Q5SSL4 (Abr),FDR=0.0019105
Active breakpoint cluster region-related protein (regulates macrophage-mediated phagocytosis)
freq
22.25
22.00
21.75
21.50
21.25
Vehicle−NoSurgery
Vehicle−Surgery
KA−NoSurgery
KA−Surgery
groups
Groups
Vehicle−NoSurgery
Vehicle−Surgery
KA−NoSurgery
KA−Surgery

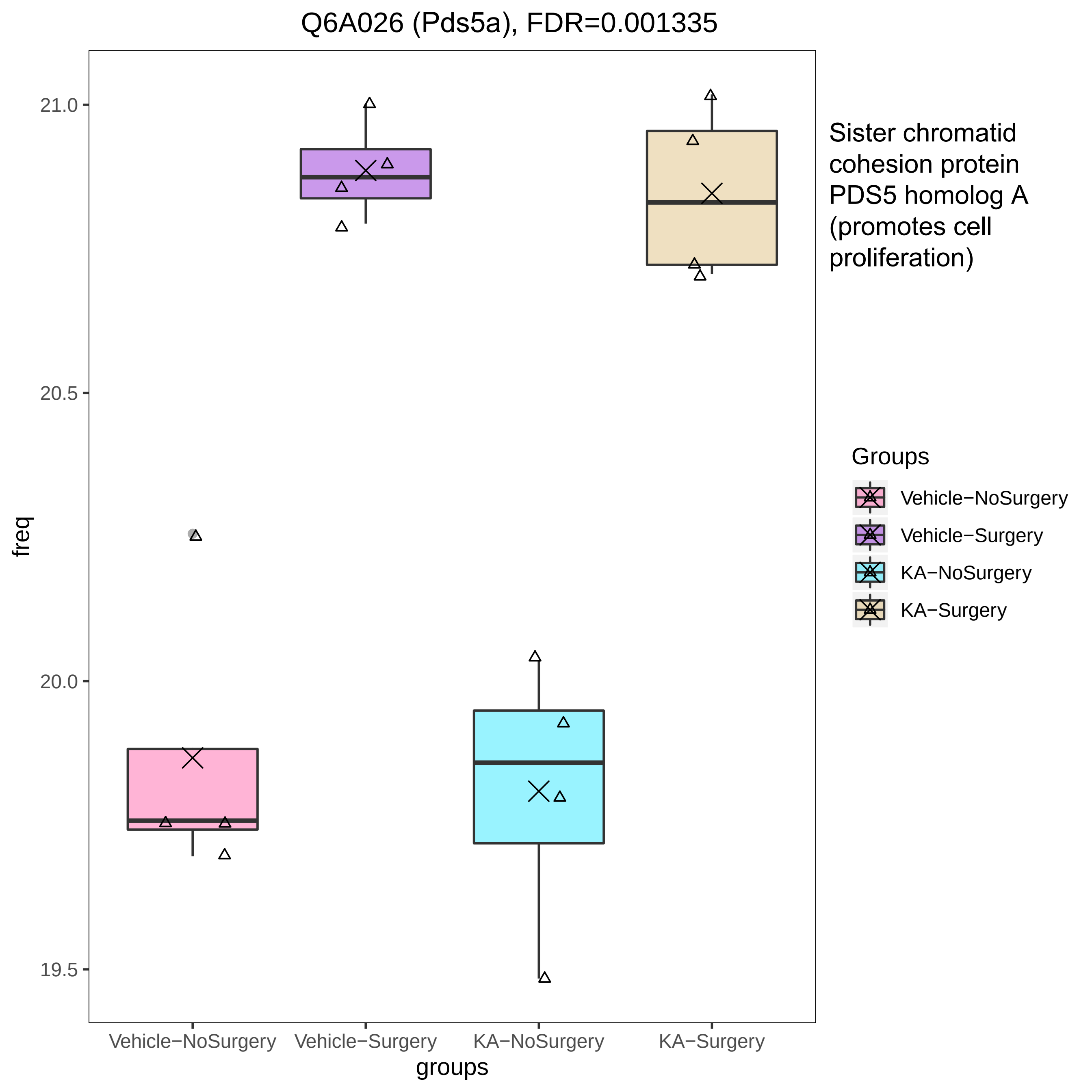
Q6A026 (Pds5a), FDR=0.001335
Sister chromatid cohesion protein PDS5 homolog A (promotes cell proliferation)
freq
21.0
20.5
20.0
19.5
groups
Vehicle−NoSurgery
Vehicle−Surgery
KA−NoSurgery
KA−Surgery
Groups
Vehicle−NoSurgery
Vehicle−Surgery
KA−NoSurgery
KA−Surgery

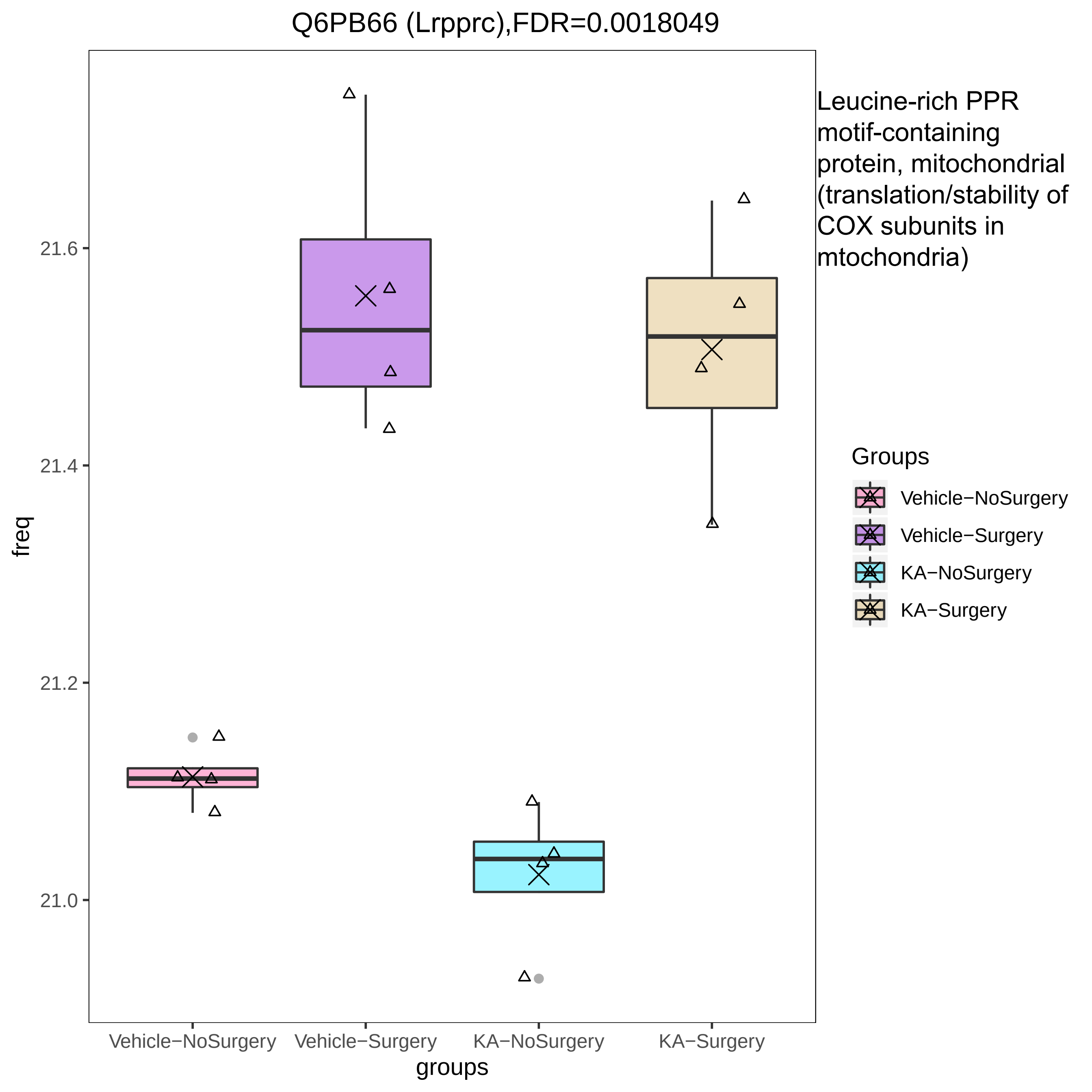

Q6PB66 (Lrpprc),FDR=0.0018049
Leucine-rich PPR motif-containing protein, mitochondrial (translation/stability of COX subunits in mtochondria)
freq
21.6
21.4
21.2
21.0
groups
Vehicle−NoSurgery
Vehicle−Surgery
KA−NoSurgery
KA−Surgery
Groups
Vehicle−NoSurgery
Vehicle−Surgery
KA−NoSurgery
KA−Surgery

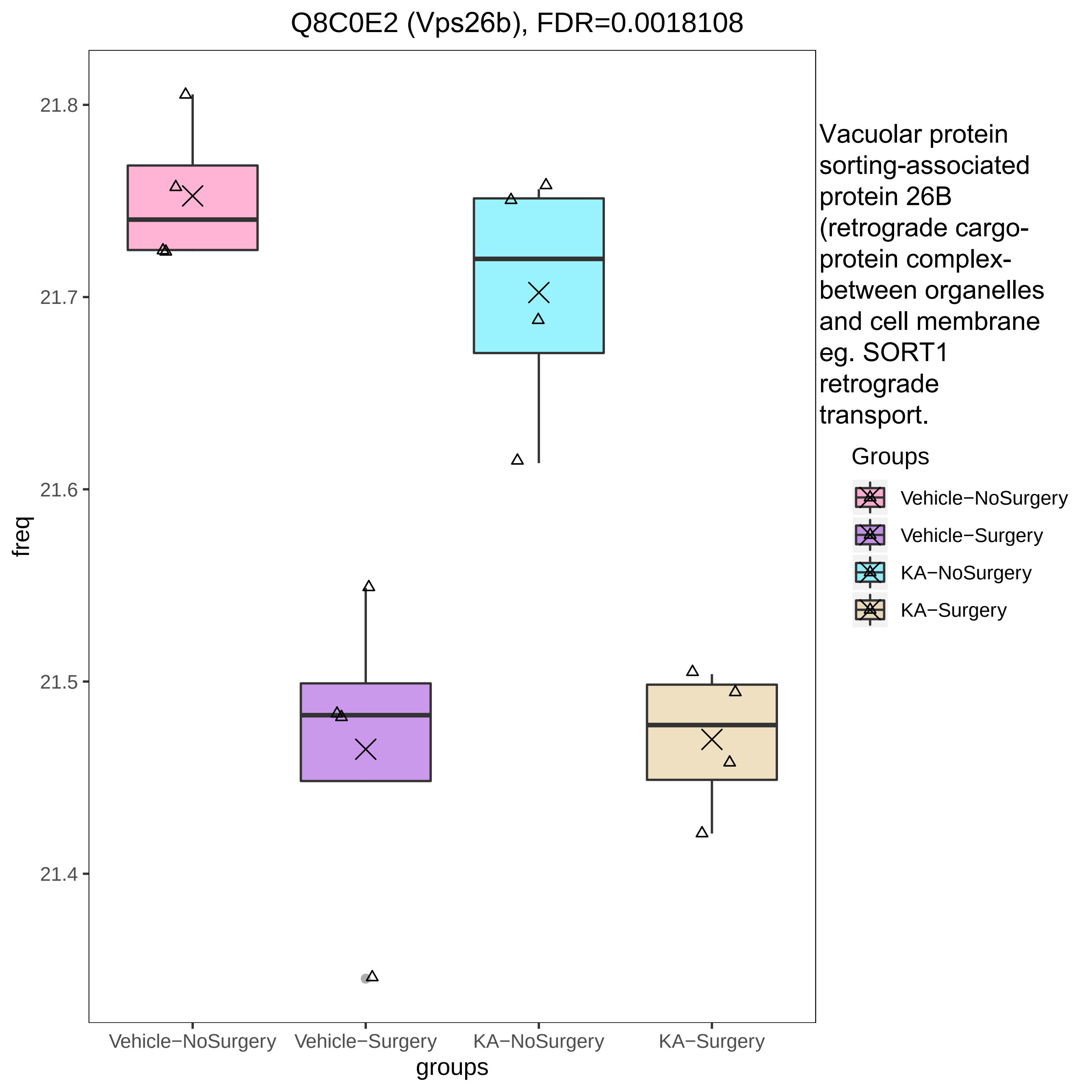
Q8C0E2 (Vps26b), FDR=0.0018108
freq
21.8
21.7
21.6
21.5
21.4
groups
Vehicle−NoSurgery
Vehicle−Surgery
KA−NoSurgery
KA−Surgery
Vacuolar protein sorting-associated protein 26B (retrograde cargo-protein complex-between organelles and cell membrane eg. SORT1 retrograde transport.
Groups
Vehicle−NoSurgery
Vehicle−Surgery
KA−NoSurgery
KA−Surgery

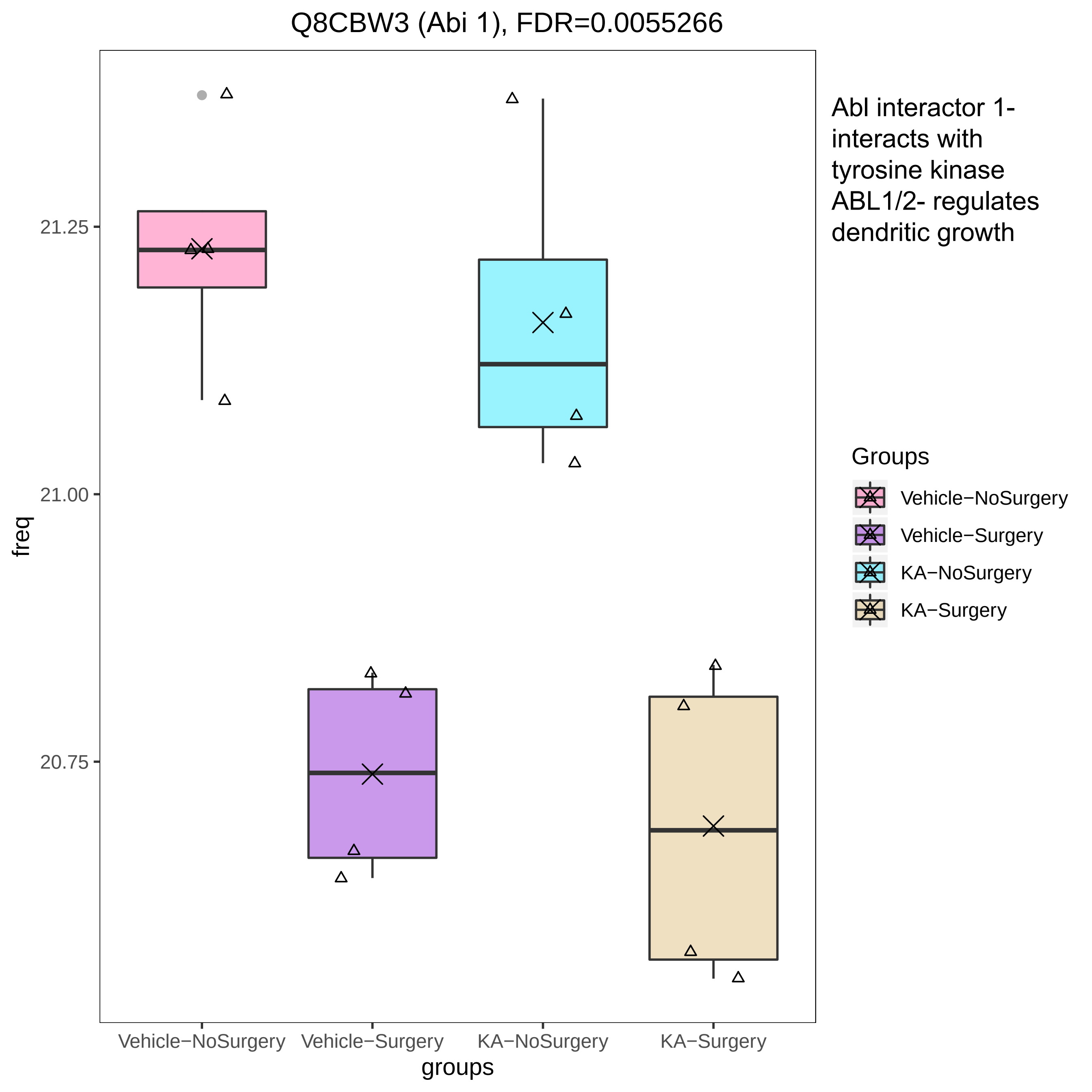
Q8CBW3 (Abi 1), FDR=0.0055266
Abl interactor 1- interacts with tyrosine kinase ABL1/2- regulates dendritic growth
freq
21.25
21.00
20.75
groups
Vehicle−NoSurgery
Vehicle−Surgery
KA−NoSurgery
KA−Surgery
Groups
Vehicle−NoSurgery
Vehicle−Surgery
KA−NoSurgery
KA−Surgery

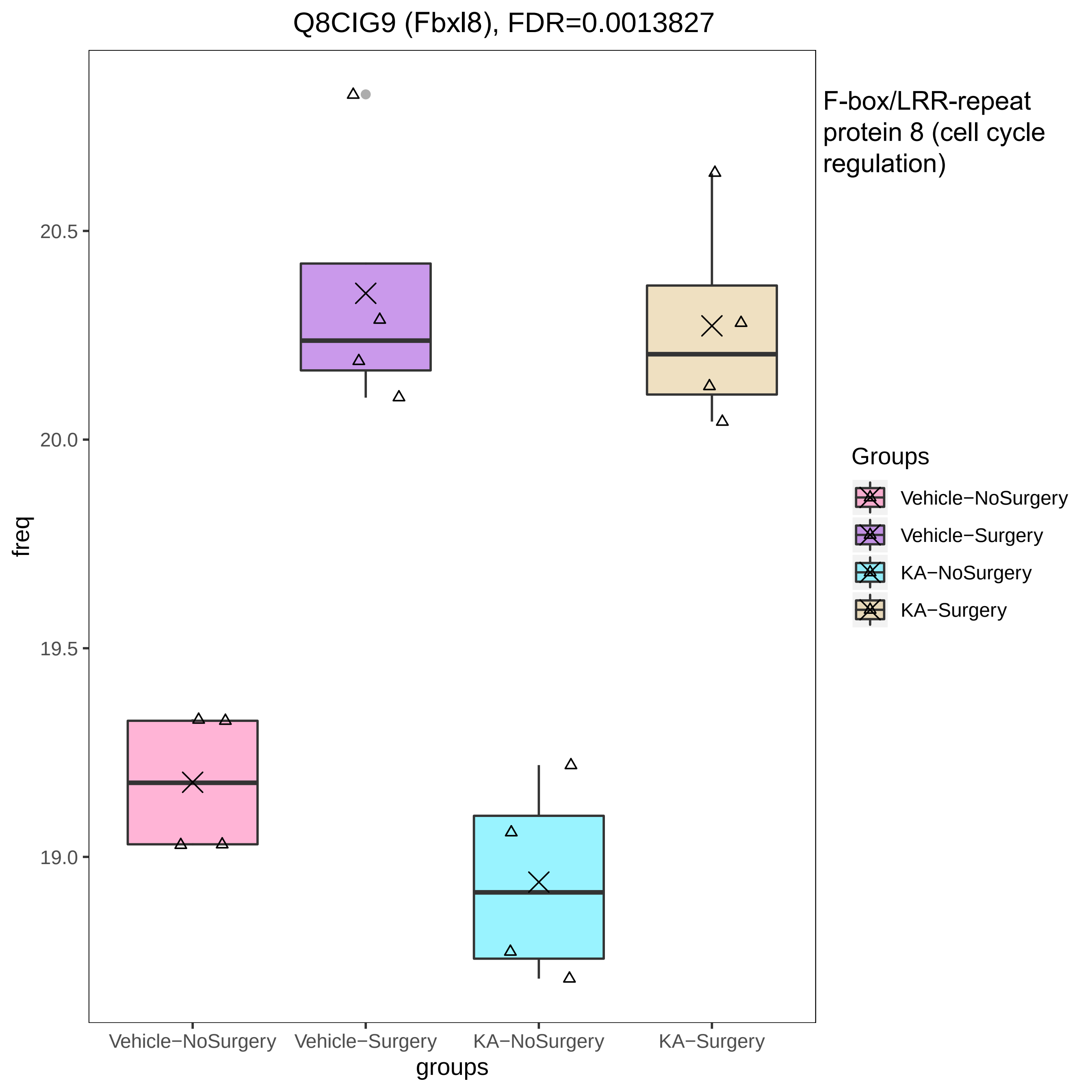

Q8CIG9 (Fbxl8), FDR=0.0013827
F-box/LRR-repeat protein 8 (cell cycle regulation)
freq
20.5
20.0
19.5
19.0
groups
Vehicle−NoSurgery
Vehicle−Surgery
KA−NoSurgery
KA−Surgery
Groups
Vehicle−NoSurgery
Vehicle−Surgery
KA−NoSurgery
KA−Surgery

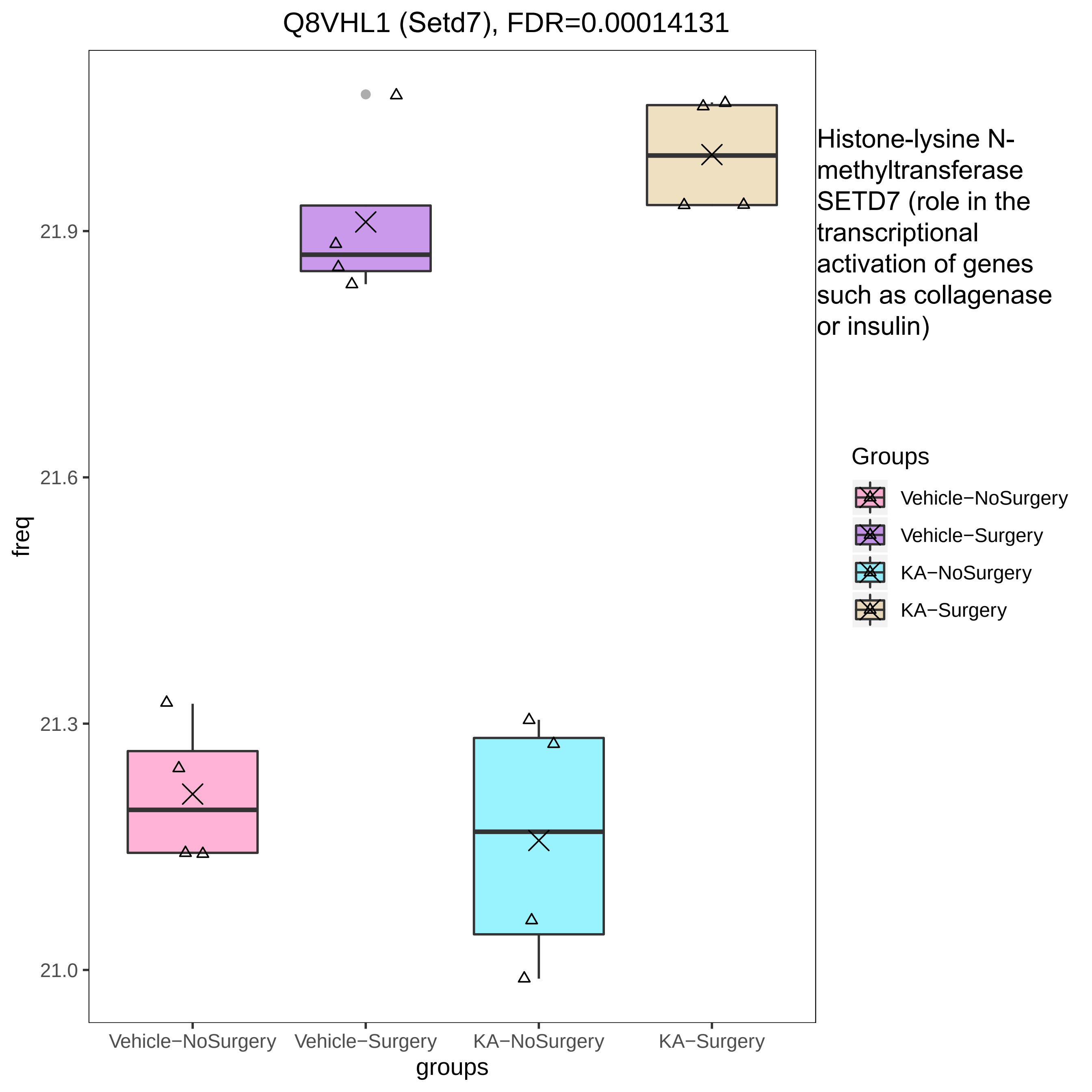
Q8VHL1 (Setd7), FDR=0.00014131
Histone-lysine N-methyltransferase SETD7 (role in the transcriptional activation of genes such as collagenase or insulin)
freq
21.9
21.6
21.3
21.0
groups
Vehicle−NoSurgery
Vehicle−Surgery
KA−NoSurgery
KA−Surgery
Groups
Vehicle−NoSurgery
Vehicle−Surgery
KA−NoSurgery
KA−Surgery

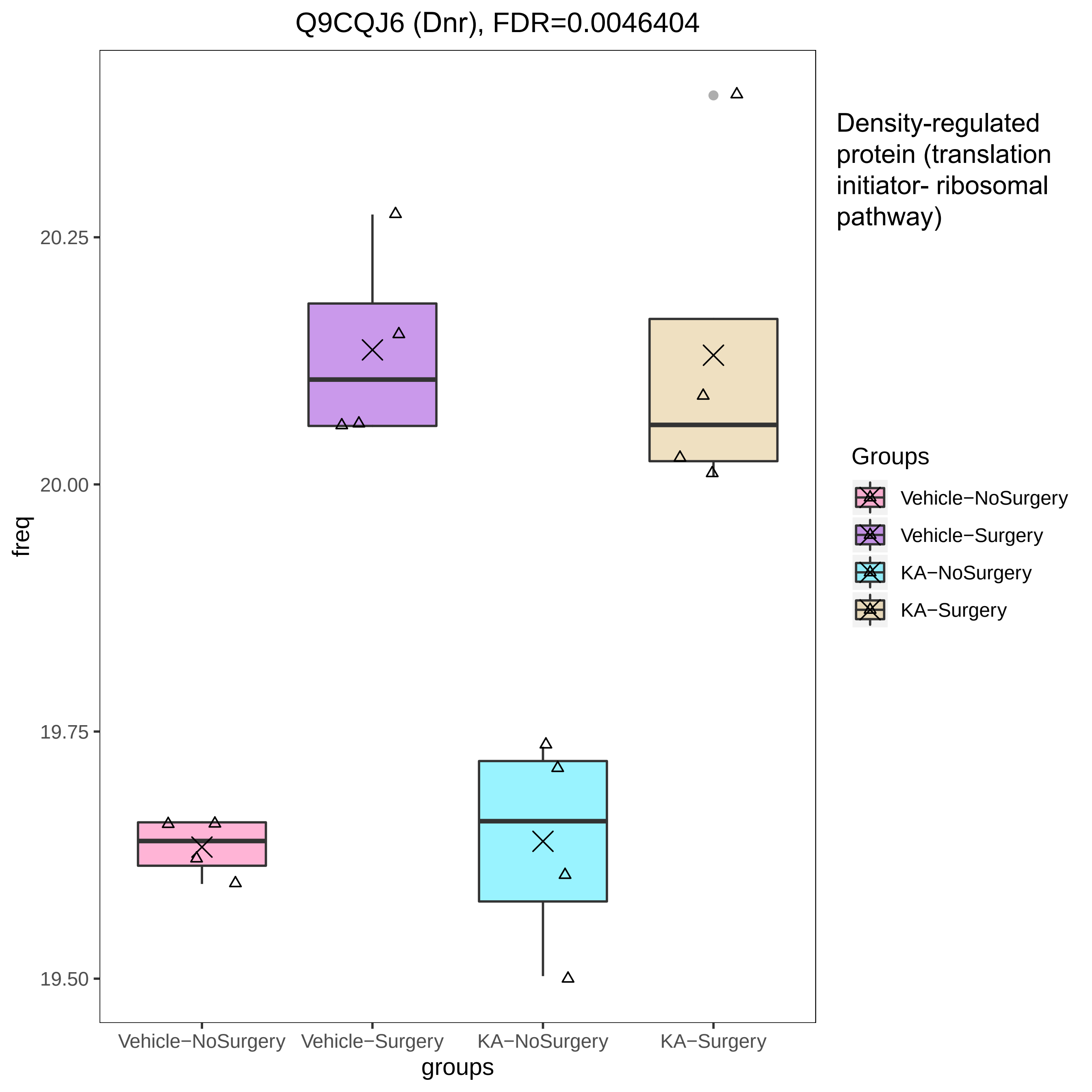

Q9CQJ6 (Dnr), FDR=0.0046404
Density-regulated protein (translation initiator- ribosomal pathway)
freq
20.25
20.00
19.75
19.50
groups
Vehicle−NoSurgery
Vehicle−Surgery
KA−NoSurgery
KA−Surgery
Groups
Vehicle−NoSurgery
Vehicle−Surgery
KA−NoSurgery
KA−Surgery

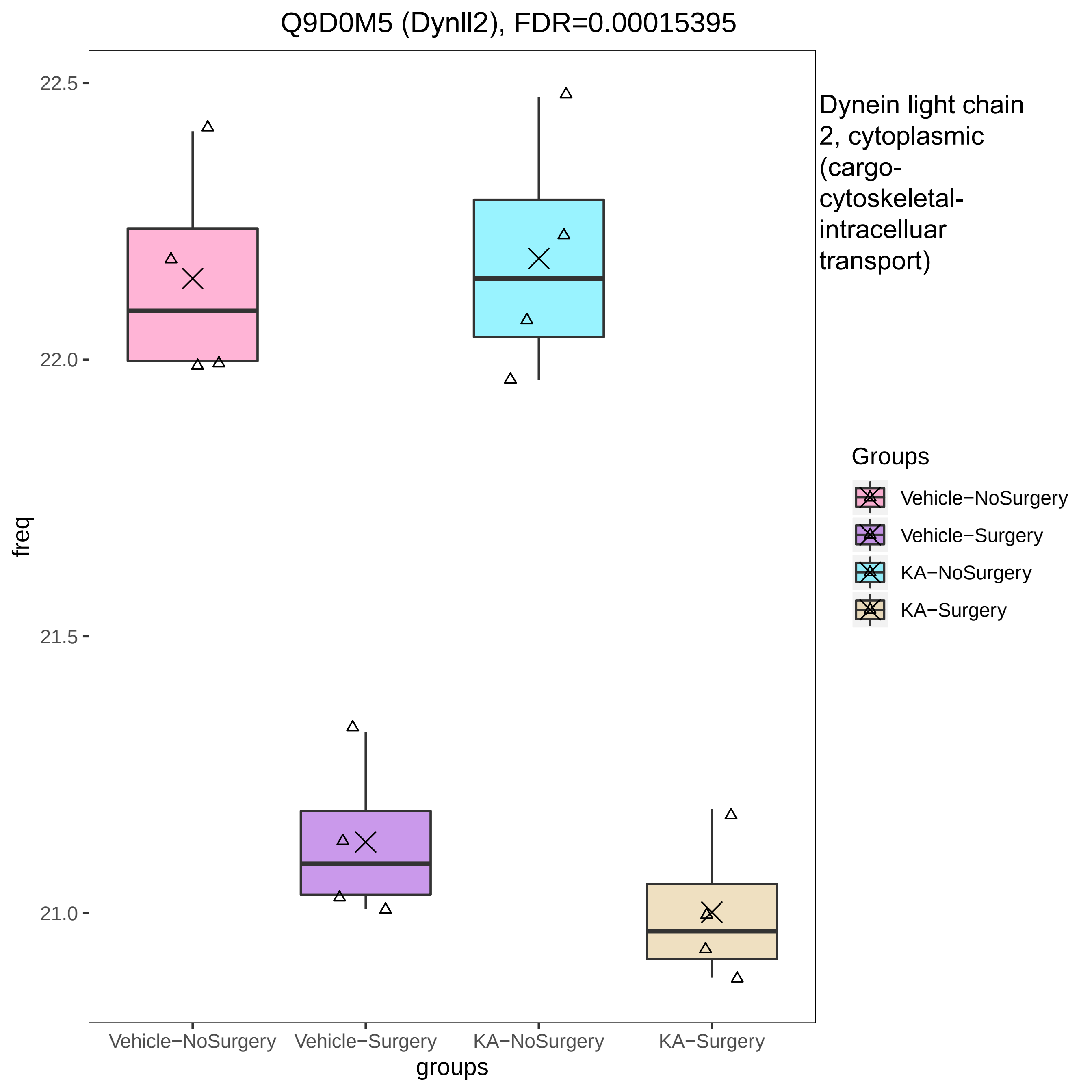
Q9D0M5 (Dynll2), FDR=0.00015395
Dynein light chain 2, cytoplasmic (cargo-cytoskeletal-intracelluar transport)
freq
22.5
22.0
21.5
21.0
groups
Vehicle−NoSurgery
Vehicle−Surgery
KA−NoSurgery
KA−Surgery
Groups
Vehicle−NoSurgery
Vehicle−Surgery
KA−NoSurgery
KA−Surgery

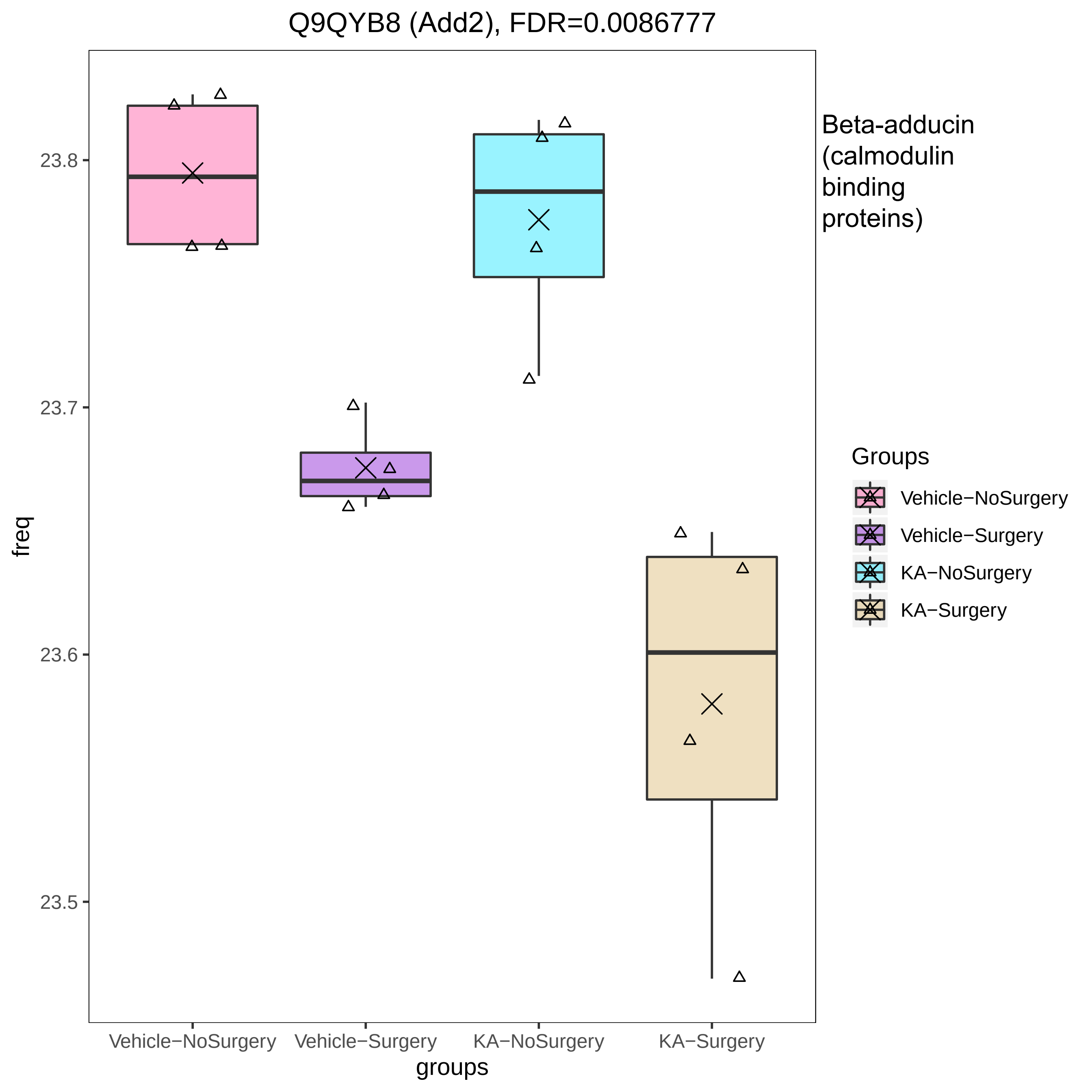

Q9QYB8 (Add2), FDR=0.0086777
Beta-adducin
(calmodulin
binding
proteins)
freq
23.8
23.7
23.6
23.5
Vehicle−NoSurgery
Vehicle−Surgery
KA−NoSurgery
KA−Surgery
groups
Groups
Vehicle−NoSurgery
Vehicle−Surgery
KA−NoSurgery
KA−Surgery

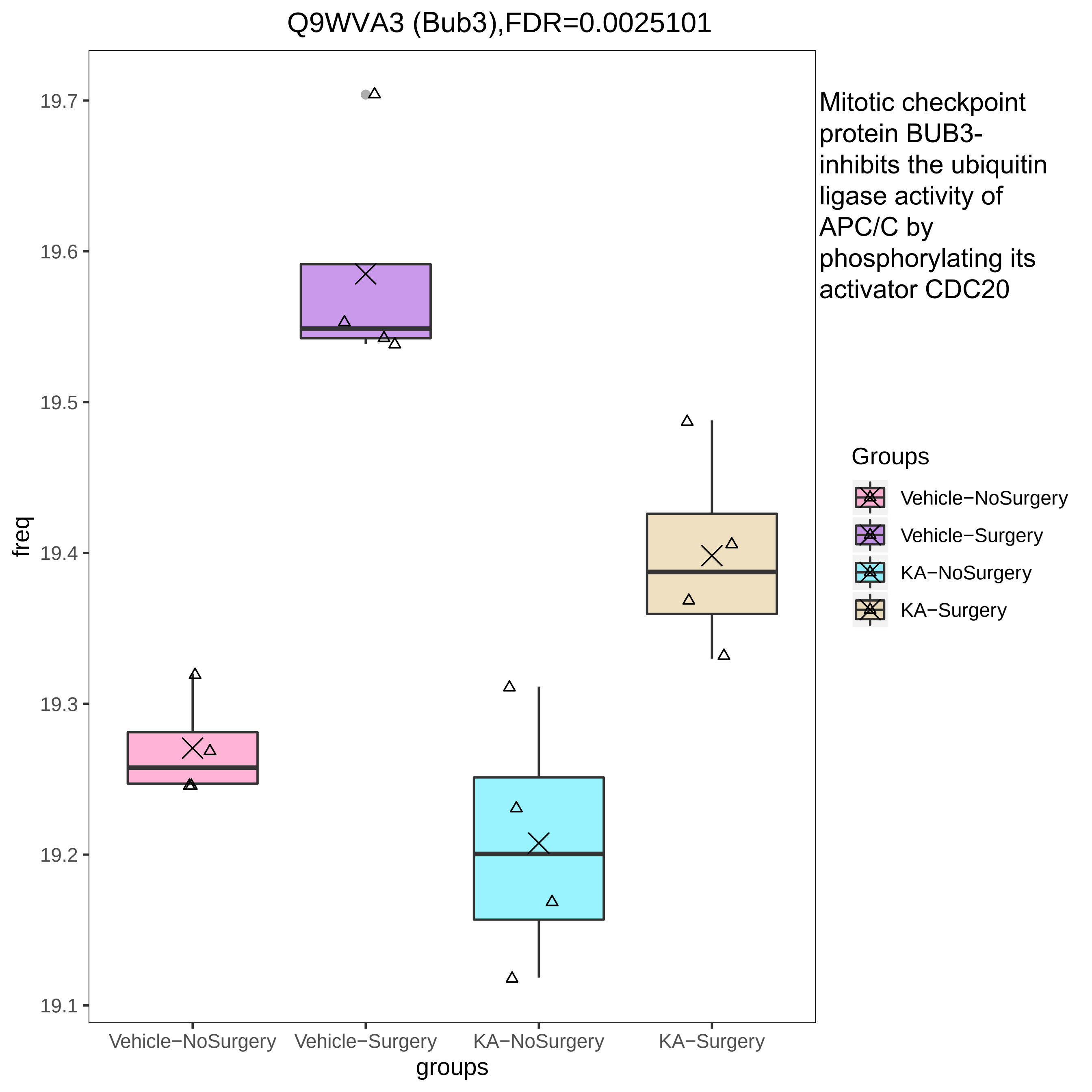
Q9WVA3 (Bub3),FDR=0.0025101
Mitotic checkpoint protein BUB3- inhibits the ubiquitin ligase activity of APC/C by phosphorylating its activator CDC20
freq
19.7
19.6
19.5
19.4
19.3
19.2
19.1
Vehicle−NoSurgery
Vehicle−Surgery
KA−NoSurgery
KA−Surgery
groups
Groups
Vehicle−NoSurgery
Vehicle−Surgery
KA−NoSurgery
KA−Surgery

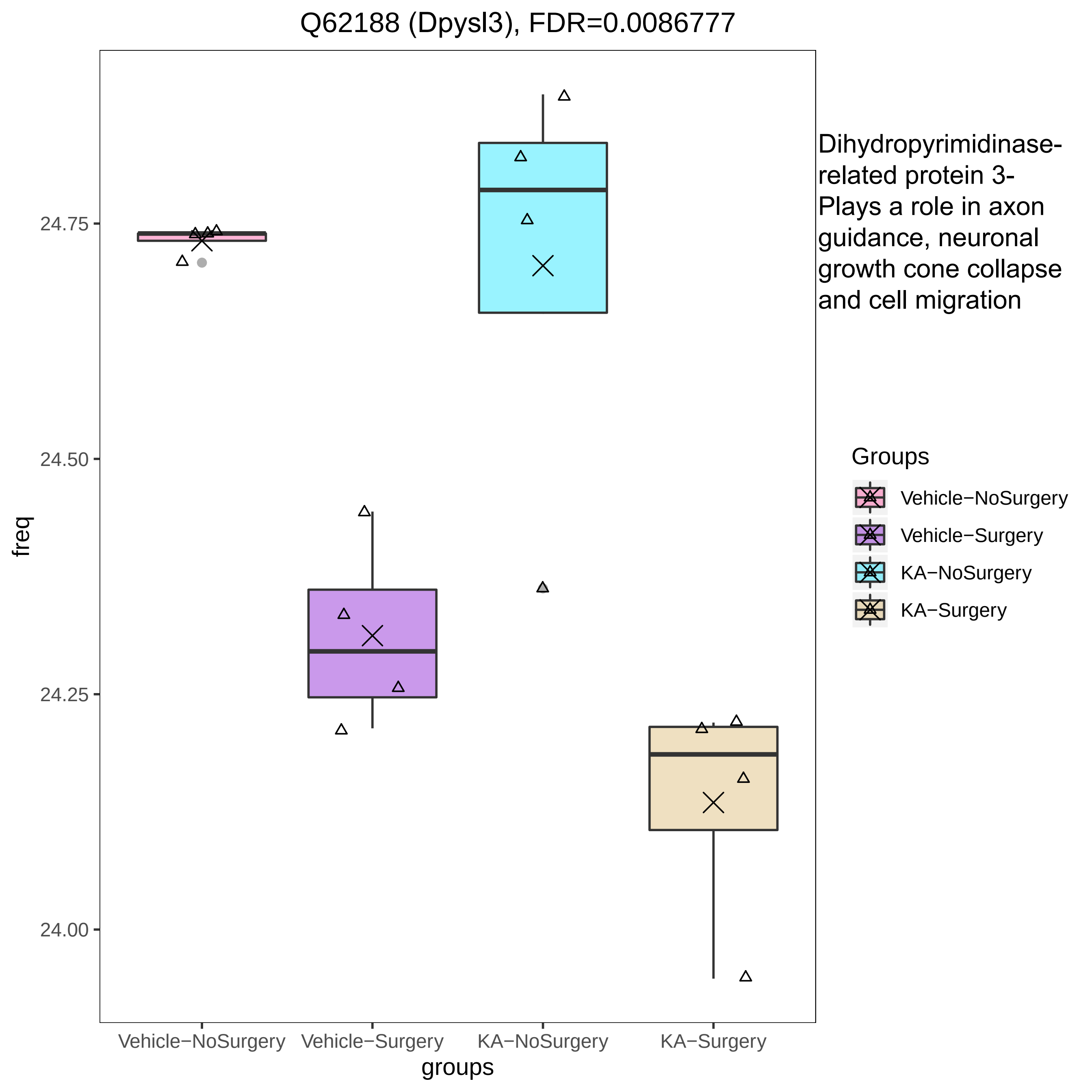

Q62188 (Dpysl3), FDR=0.0086777
Dihydropyrimidinase-related protein 3-Plays a role in axon guidance, neuronal growth cone collapse and cell migration
freq
24.75
24.50
24.25
24.00
groups
Vehicle−NoSurgery
Vehicle−Surgery
KA−NoSurgery
KA−Surgery
Groups
Vehicle−NoSurgery
Vehicle−Surgery
KA−NoSurgery
KA−Surgery

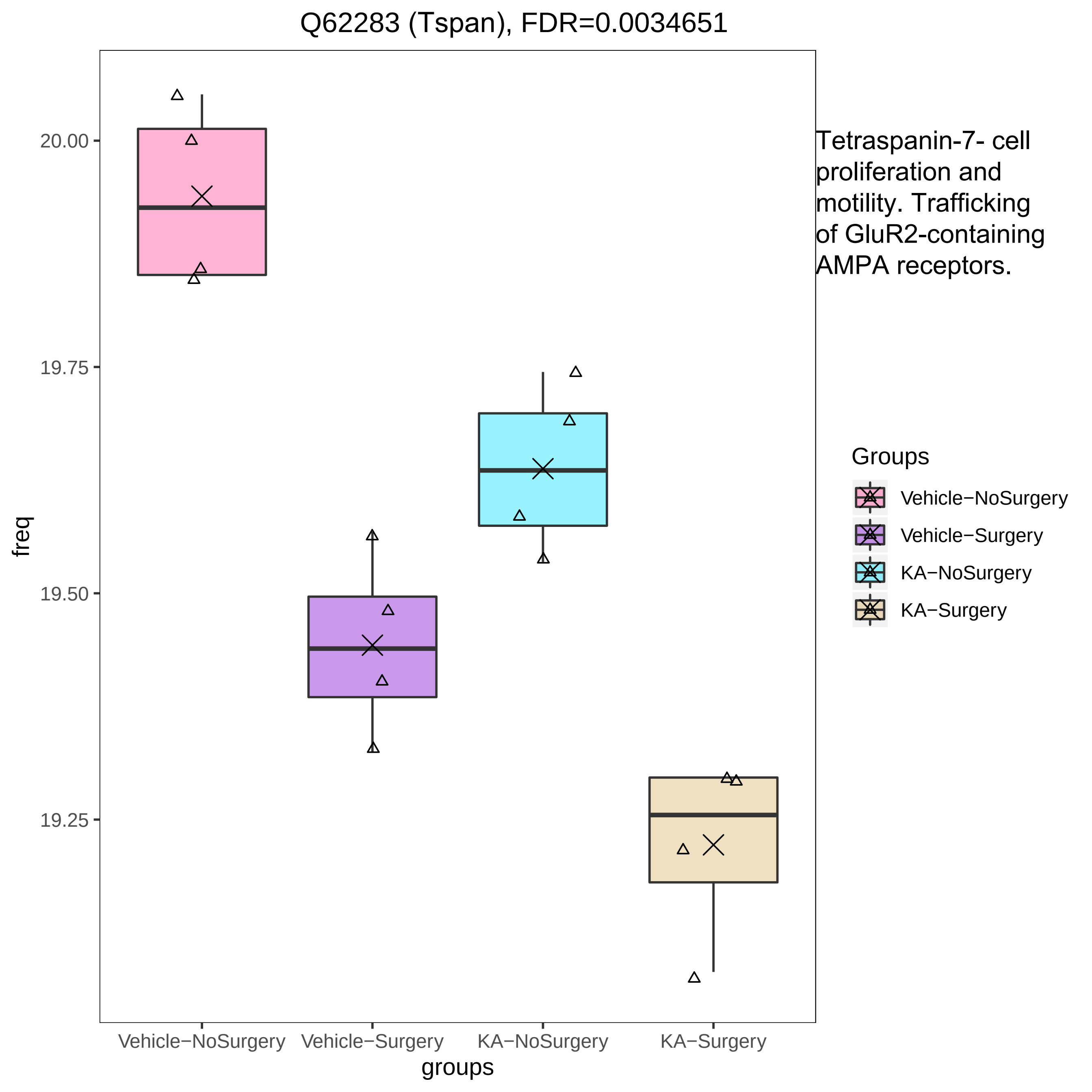
Q62283 (Tspan), FDR=0.0034651
Tetraspanin-7- cell proliferation and motility. Trafficking of GluR2-containing AMPA receptors.
freq
20.00
19.75
19.50
19.25
groups
Vehicle−NoSurgery
Vehicle−Surgery
KA−NoSurgery
KA−Surgery
Groups
Vehicle−NoSurgery
Vehicle−Surgery
KA−NoSurgery
KA−Surgery
