# Supplementary material for: The Impacts of Surgery and Intracerebral Electrodes in C57BL/6J Mouse Kainate Model of Epileptogenesis: Seizure Threshold, Proteomics, and Cytokine Profiles
Source: Front Neurol. 2021 Jul 12;12:625017. doi: 10.3389/fneur.2021.625017 (PMC8312573; doi:10.3389/fneur.2021.625017)
Supplement: Supplementary Figure 2 — The schematic diagrams of KEGG pathways (vehicle: surgery vs. no surgery). [file Data_Sheet_2.pdf]

SYNAPTIC VESICLE CYCLE

mmu04721

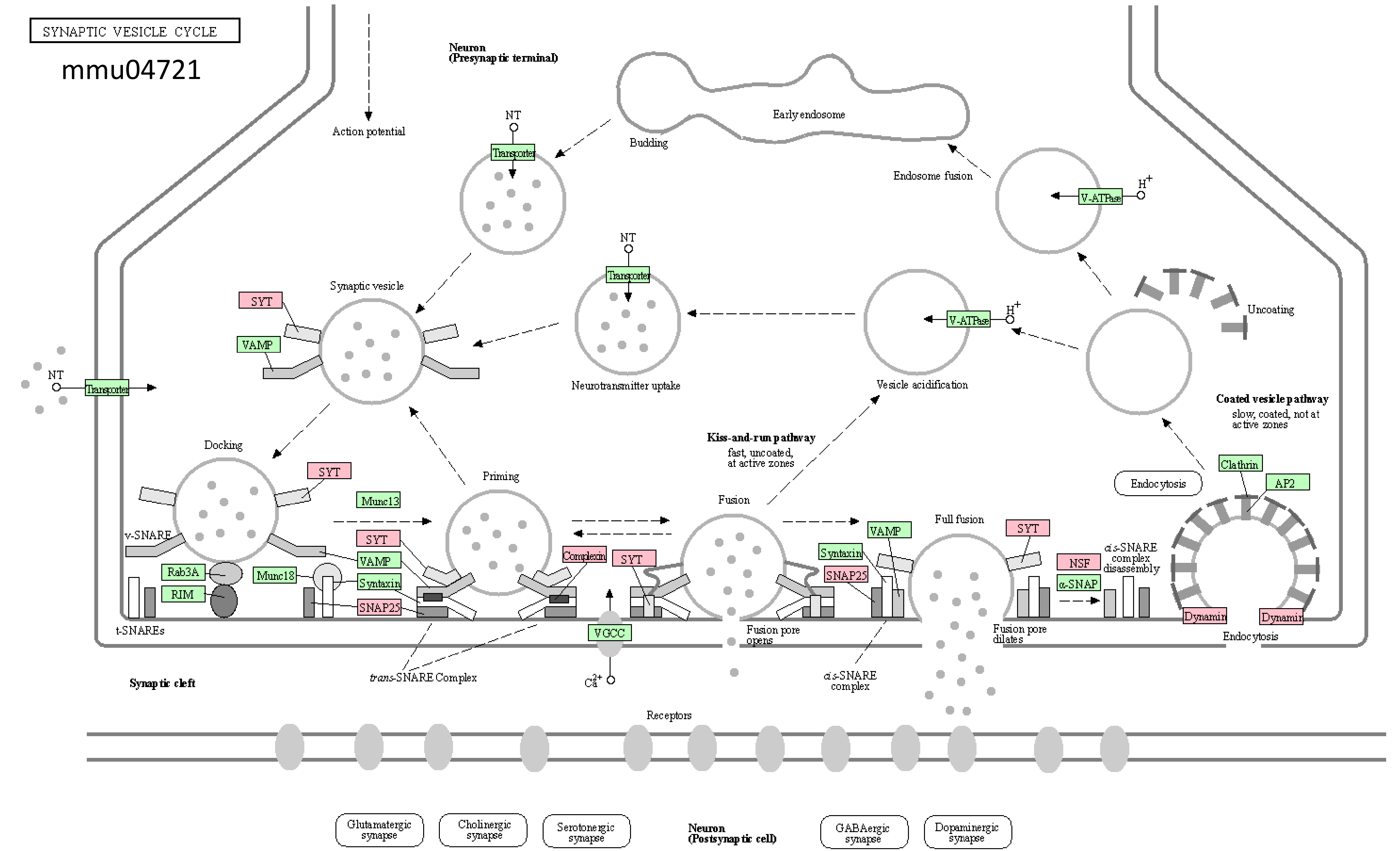

04721 6/12/19
(c) Kanehisa Laboratories

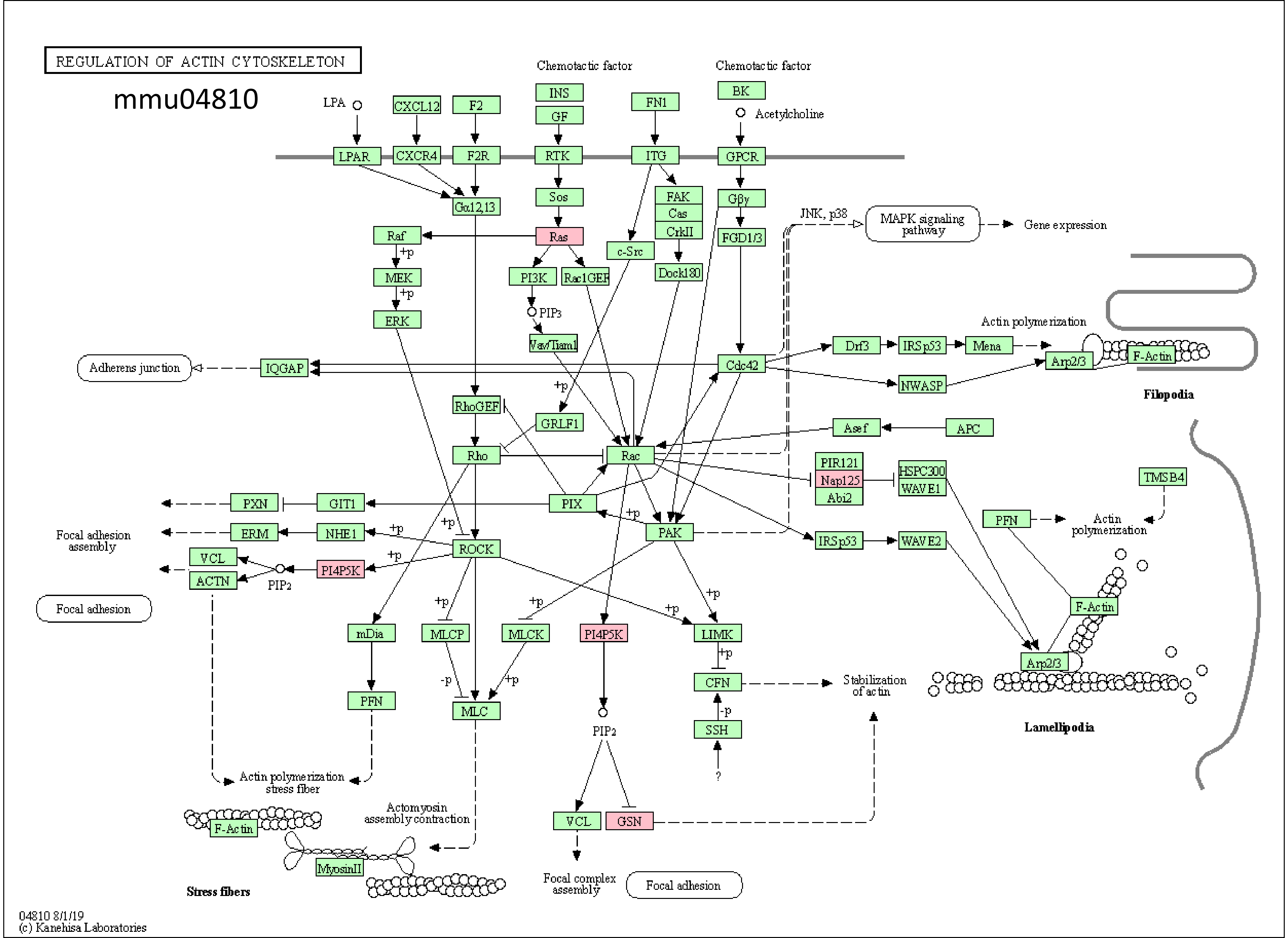
REGULATION OF ACTIN CYTOSKELETON
mmu04810
Chemotactic factor
Chemotactic factor
LPA
CXCL12
F2
INS
GF
FN1
BK
Acetylcholine
LPAR
CXCR4
F2R
RTK
ITG
GPCR
Gα12,13
Sos
FAK
Cas
CrkII
Gβγ
Raf
Ras
c-Src
FGD1/3
JNK, p38
MAPK signaling pathway
Gene expression
MEK
PI3K
Rac1GEF
Dock180
ERK
PIP3
Vav/Tiam1
Actin polymerization
Drf3
IRSp53
Mena
Arp2/3
F-Actin
Adherens junction
IQGAP
Cdc42
NWASP
Filopodia
RhoGEF
GRLF1
Asef
APC
Rho
Rac
PIR121
Nap125
Abi2
HSPC300
WAVE1
TMSB4
PXN
GIT1
PIX
PAK
IRSp53
WAVE2
PFN
Actin polymerization
Focal adhesion assembly
ERM
NHE1
ROCK
VCL
ACTN
PIP2
PI4P5K
Focal adhesion
F-Actin
mDia
MLCP
MLCK
PI4P5K
LIMK
Arp2/3
CFN
Stabilization of actin
PFN
MLC
SSH
Lamellipodia
PIP2
?
Actin polymerization stress fiber
Actomyosin assembly contraction
VCL
GSN
F-Actin
MyosinII
Focal complex assembly
Focal adhesion
Stress fibers
04810 8/1/19
(c) Kanehisa Laboratories

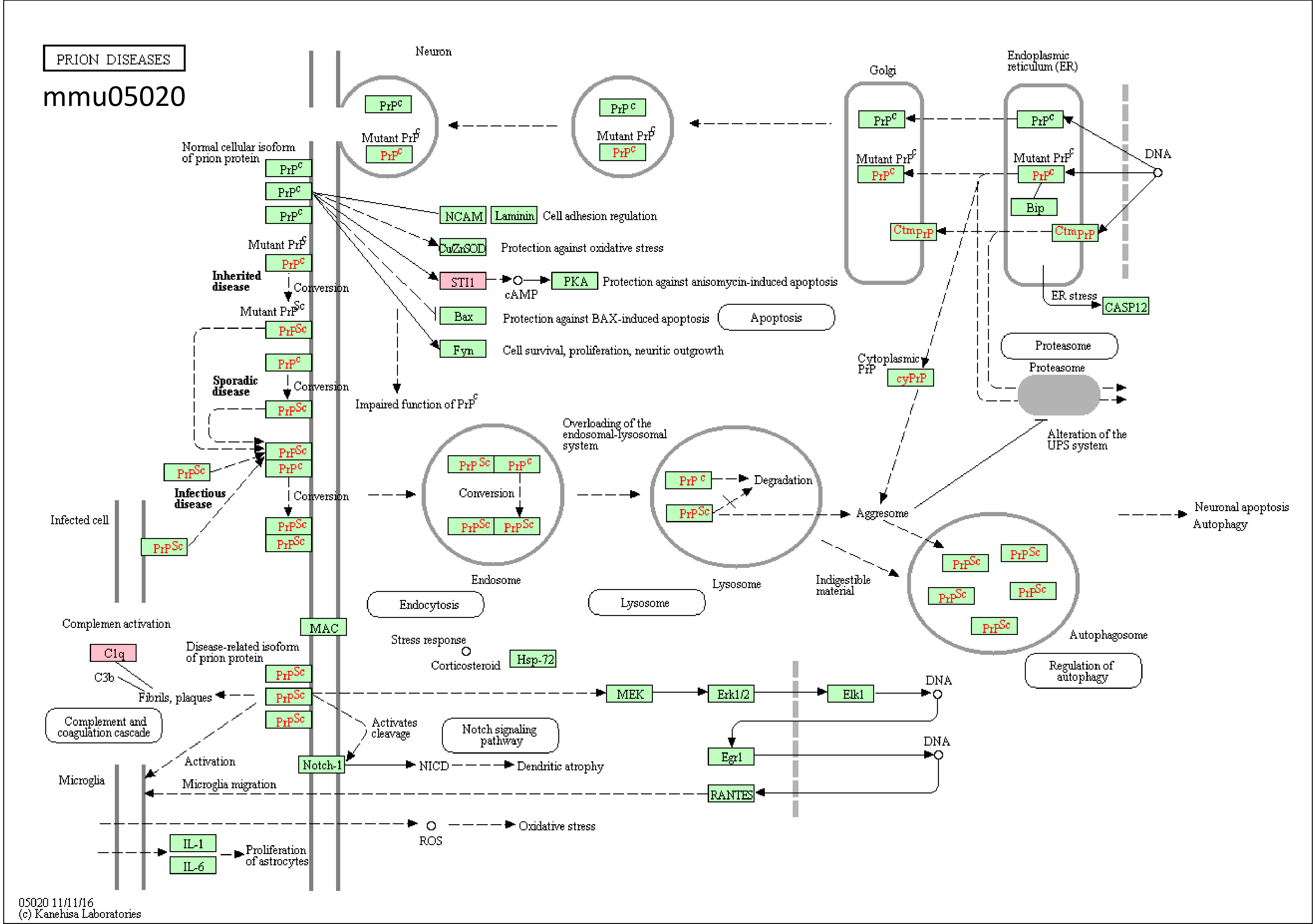
PRION DISEASES
mmu05020
Neuron
Golgi
Endoplasmic reticulum (ER)
DNA
Normal cellular isoform of prion protein
PrPc
Mutant PrPc
NCAM
Laminin
Cell adhesion regulation
CuZnSOD
Protection against oxidative stress
STI1
cAMP
PKA
Protection against anisomycin-induced apoptosis
Bax
Protection against BAX-induced apoptosis
Apoptosis
Fyn
Cell survival, proliferation, neuritic outgrowth
Bip
CtmPrP
ER stress
CASP12
Proteasome
Cytoplasmic PrP
cyPrP
Inherited disease
Conversion
Mutant PrPSc
PrPSc
Sporadic disease
Impaired function of PrPc
Alteration of the UPS system
Infectious disease
Infected cell
Overloading of the endosomal-lysosomal system
Degradation
Aggresome
Neuronal apoptosis
Autophagy
Endosome
Lysosome
Indigestible material
Autophagosome
Endocytosis
Regulation of autophagy
Complemen activation
MAC
C1q
C3b
Disease-related isoform of prion protein
Fibrils, plaques
Complement and coagulation cascade
Stress response
Corticosteroid
Hsp-72
MEK
Erk1/2
Elk1
Egr1
RANTES
Activates cleavage
Notch signaling pathway
Notch-1
NICD
Dendritic atrophy
Activation
Microglia
Microglia migration
ROS
Oxidative stress
IL-1
IL-6
Proliferation of astrocytes
05020 11/11/16
(c) Kanehisa Laboratories

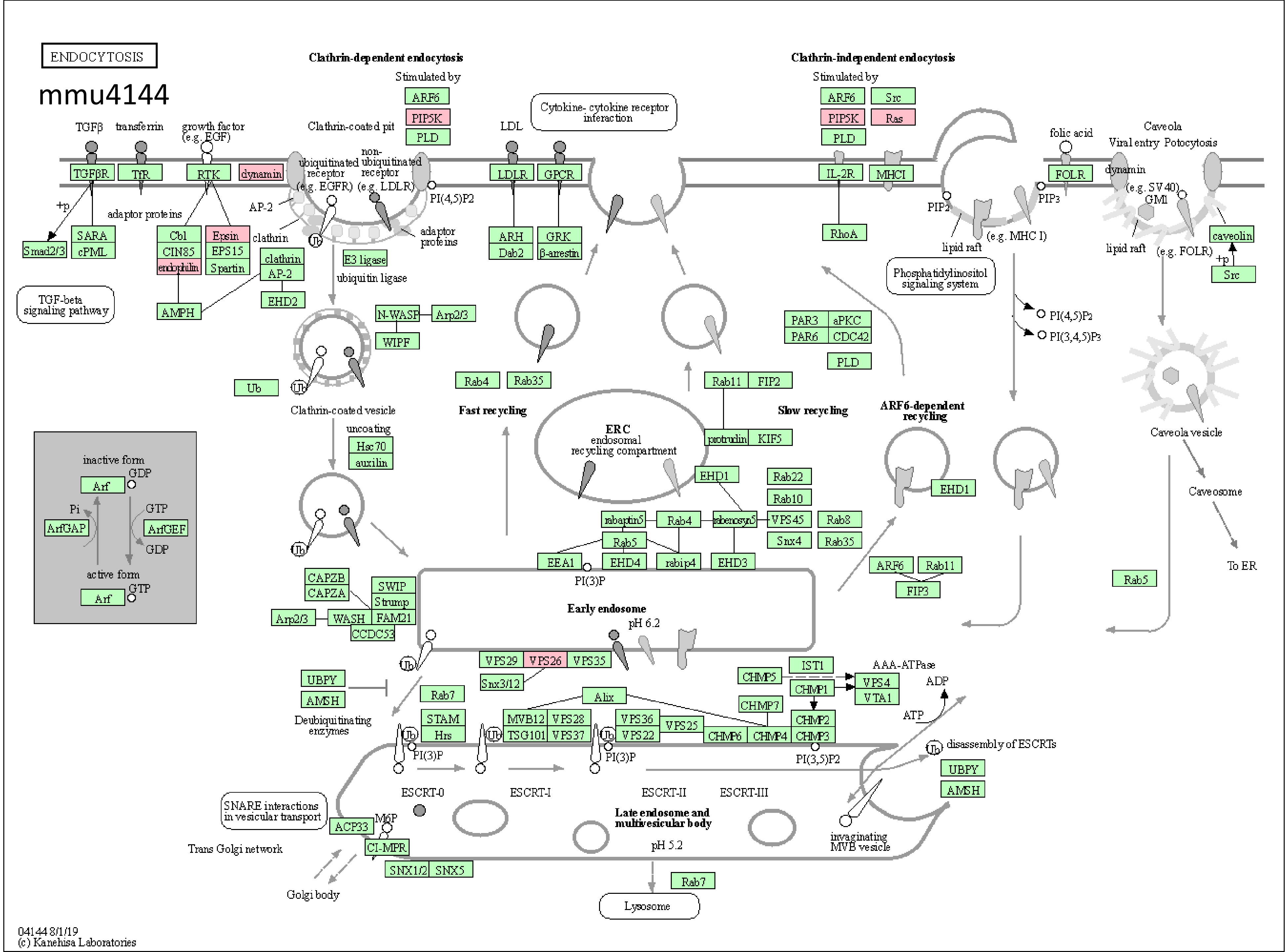
ENDOCYTOSIS
mmu4144
Clathrin-dependent endocytosis
Stimulated by
ARF6
PIP5K
PLD
Clathrin-independent endocytosis
Stimulated by
ARF6
Src
PIP5K
Ras
PLD
TGFβ
transferrin
growth factor (e.g. EGF)
Clathrin-coated pit
LDL
Cytokine- cytokine receptor interaction
folic acid
Caveola
Viral entry Potocytosis
TGFβR
TfR
RTK
dynamin
ubiquitinated receptor (e.g. EGFR)
non-ubiquitinated receptor (e.g. LDLR)
PI(4,5)P2
LDLR
GPCR
IL-2R
MHCI
FOLR
dynamin
(e.g. SV40)
GM1
+p
adaptor proteins
AP-2
SARA
cPML
Smad2/3
Cbl
CIN85
endophilin
Epsin
EPS15
Spartin
clathrin
adaptor proteins
ARH
Dab2
GRK
β-arrestin
RhoA
PIP2
PIP3
lipid raft
(e.g. MHC I)
lipid raft
(e.g. FOLR)
caveolin
Src
clathrin
AP-2
E3 ligase
ubiquitin ligase
EHD2
AMPH
TGF-beta signaling pathway
Phosphatidylinositol signaling system
PI(4,5)P2
PI(3,4,5)P3
N-WASP
Arp2/3
WIPF
PAR3
aPKC
PAR6
CDC42
PLD
Ub
Ub
Rab4
Rab35
Rab11
FIP2
Clathrin-coated vesicle
Fast recycling
Slow recycling
ARF6-dependent recycling
Caveola vesicle
uncoating
Hsc70
auxilin
ERC
endosomal recycling compartment
protrudin
KIF5
inactive form
GDP
Arf
Pi
GTP
ArfGAP
ArfGEF
GDP
active form
GTP
Arf
EHD1
Rab22
Rab10
EHD1
Caveosome
rabaptin5
Rab4
rabenosyn5
VPS45
Rab8
Rab5
Snx4
Rab35
Ub
EEA1
EHD4
rabip4
EHD3
ARF6
Rab11
FIP3
Rab5
To ER
PI(3)P
CAPZB
CAPZA
SWIP
Strump
Arp2/3
WASH
FAM21
CCDC53
Early endosome
pH 6.2
Ub
VPS29
VPS26
VPS35
Snx3/12
UBPY
AMSH
Deubiquitinating enzymes
Rab7
Alix
CHMP5
IST1
CHMP1
AAA-ATPase
VPS4
VTA1
ADP
CHMP7
ATP
STAM
Hrs
MVB12
VPS28
TSG101
VPS37
VPS36
VPS22
VPS25
CHMP6
CHMP4
CHMP2
CHMP3
Ub
Ub
Ub
disassembly of ESCRTs
PI(3)P
PI(3)P
PI(3,5)P2
UBPY
AMSH
ESCRT-0
ESCRT-I
ESCRT-II
ESCRT-III
SNARE interactions in vesicular transport
M6P
ACP33
Late endosome and multivesicular body
pH 5.2
invaginating MVB vesicle
Trans Golgi network
CI-MPR
SNX1/2
SNX5
Golgi body
Rab7
Lysosome
04144 8/1/19
(c) Kanehisa Laboratories

mmu04070

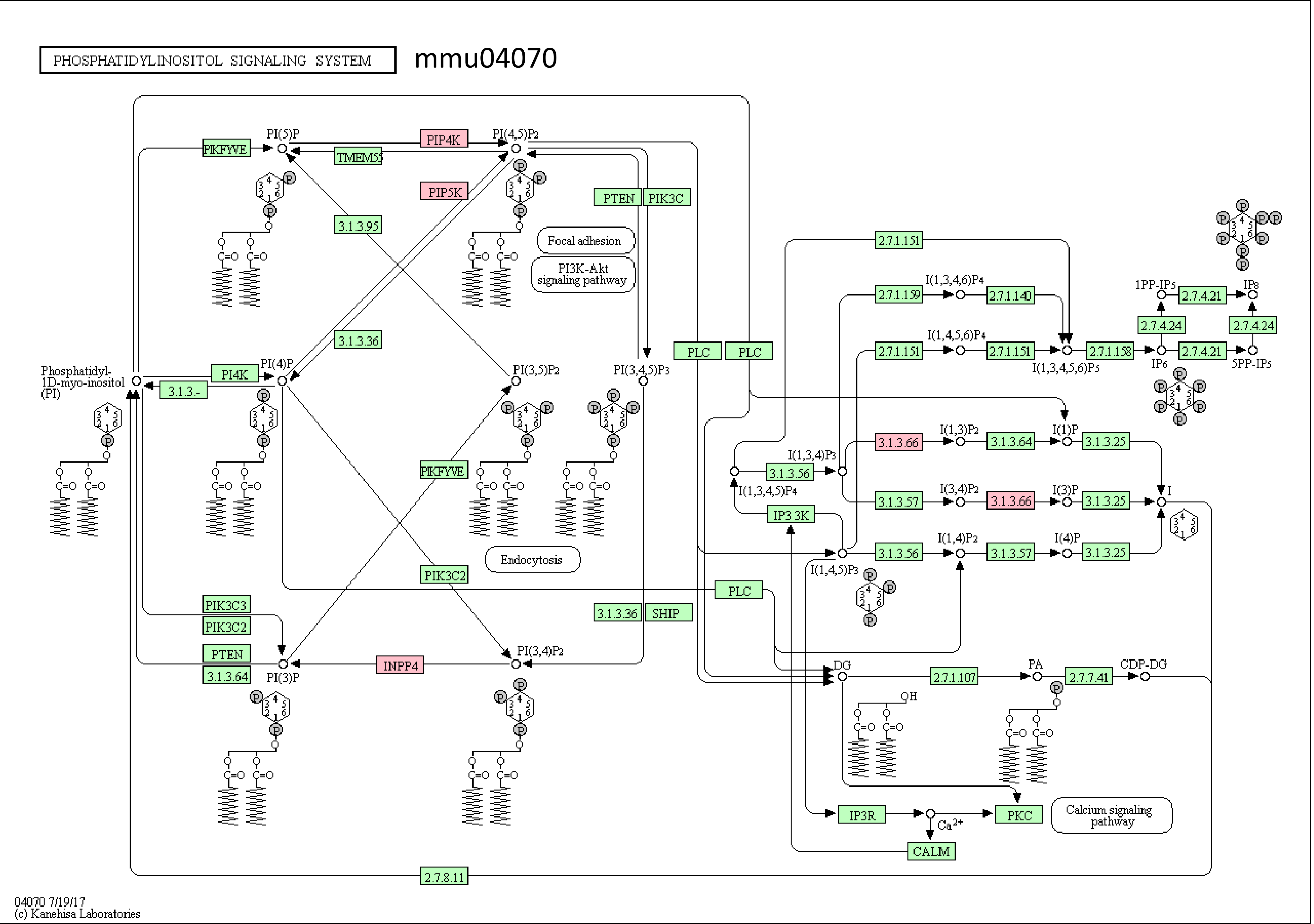

PHOSPHATIDYLINOSITOL SIGNALING SYSTEM
PI(5)P
PIKFYVE
PIP4K
TMEM55
PI(4,5)P2
PIP5K
3.1.3.95
PTEN
PIK3C
Focal adhesion
PI3K-Akt signaling pathway
3.1.3.36
Phosphatidyl-1D-myo-inositol (PI)
PI4K
PI(4)P
3.1.3.-
PI(3,5)P2
PI(3,4,5)P3
PLC
PIKFYVE
Endocytosis
PIK3C2
PLC
3.1.3.36
SHIP
PIK3C3
PIK3C2
PTEN
3.1.3.64
PI(3)P
INPP4
PI(3,4)P2
2.7.8.11
2.7.1.151
2.7.1.159
I(1,3,4,6)P4
2.7.1.140
2.7.1.151
I(1,4,5,6)P4
2.7.1.151
I(1,3,4,5,6)P5
2.7.1.158
IP6
1PP-IP5
2.7.4.21
IP8
2.7.4.24
2.7.4.24
2.7.4.21
5PP-IP5
3.1.3.66
I(1,3)P2
3.1.3.64
I(1)P
3.1.3.25
I(1,3,4)P3
3.1.3.56
I(1,3,4,5)P4
3.1.3.57
I(3,4)P2
3.1.3.66
I(3)P
3.1.3.25
I
IP3 3K
3.1.3.56
I(1,4)P2
3.1.3.57
I(4)P
3.1.3.25
I(1,4,5)P3
DG
2.7.1.107
PA
2.7.7.41
CDP-DG
IP3R
Ca2+
PKC
Calcium signaling pathway
CALM
04070 7/19/17
(c) Kanehisa Laboratories

mmu04722

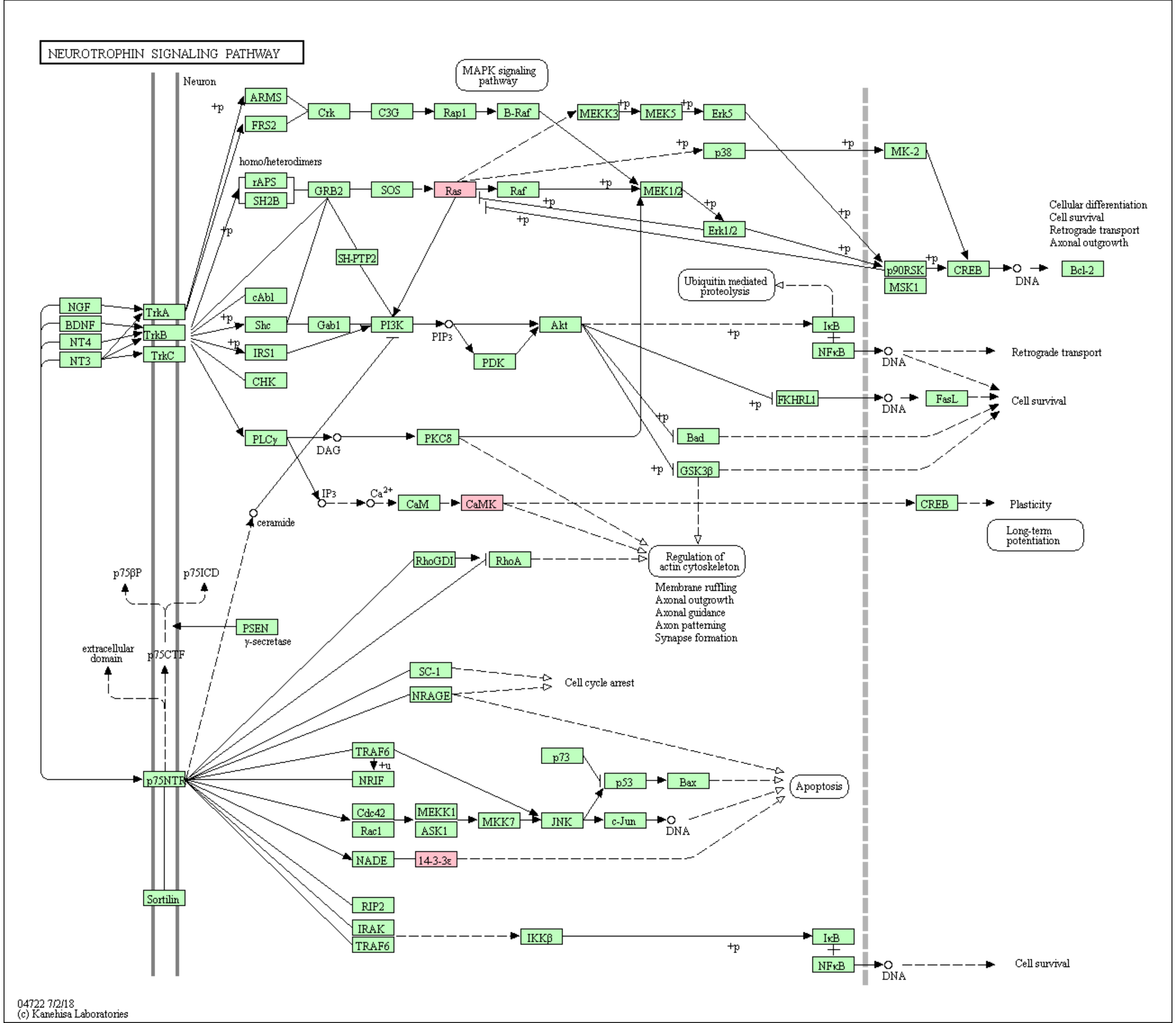

NEUROTROPHIN SIGNALING PATHWAY
Neuron
MAPK signaling pathway
ARMS
FRS2
Crk
C3G
Rap1
B-Raf
MEKK3
MEK5
Erk5
p38
MK-2
homo/heterodimers
rAPS
SH2B
GRB2
SOS
Ras
Raf
MEK1/2
Erk1/2
SH-PTP2
p90RSK
MSK1
CREB
DNA
Bcl-2
Cellular differentiation
Cell survival
Retrograde transport
Axonal outgrowth
Ubiquitin mediated proteolysis
cAbl
NGF
BDNF
NT4
NT3
TrkA
TrkB
TrkC
Shc
Gab1
PI3K
PIP3
PDK
Akt
IκB
NFκB
Retrograde transport
Cell survival
IRS1
CHK
FKHRL1
FasL
Bad
GSK3β
PLCγ
DAG
PKCδ
IP3
Ca2+
CaM
CaMK
CREB
Plasticity
Long-term potentiation
ceramide
RhoGDI
RhoA
Regulation of actin cytoskeleton
Membrane ruffling
Axonal outgrowth
Axonal guidance
Axon patterning
Synapse formation
p75βP
p75ICD
PSEN
γ-secretase
extracellular domain
p75CTF
SC-1
NRAGE
Cell cycle arrest
TRAF6
NRIF
p73
p53
Bax
Apoptosis
p75NTR
Cdc42
Rac1
MEKK1
ASK1
MKK7
JNK
c-Jun
NADE
14-3-3ε
Sortilin
RIP2
IRAK
TRAF6
IKKβ
IκB
NFκB
Cell survival
04722 7/2/18
(c) Kanehisa Laboratories

mmu05134

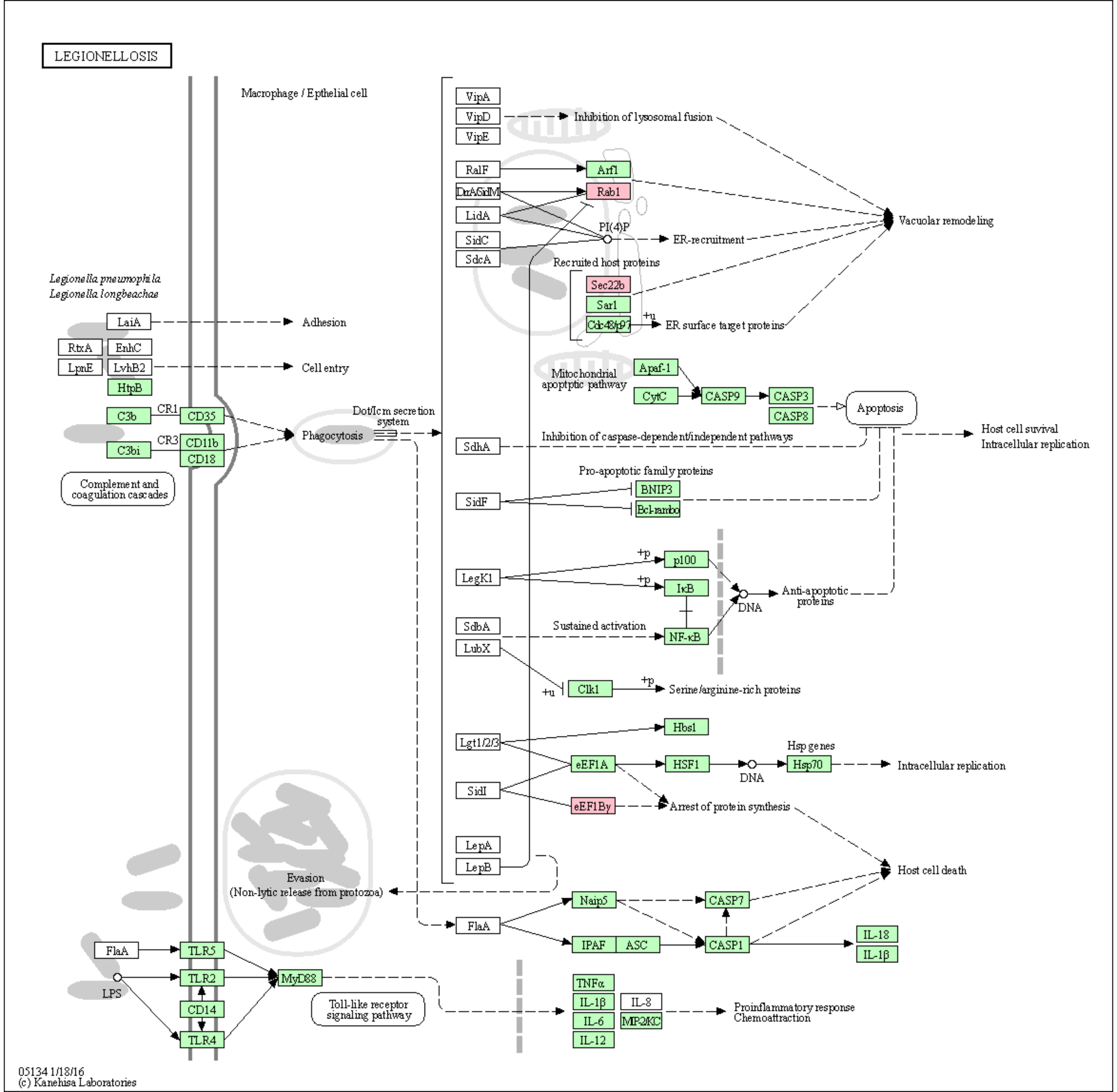

LEGIONELLOSIS
Macrophage / Epthelial cell
Legionella pneumophila
Legionella longbeachae
LaiA
Adhesion
RtxA
EnhC
LpnE
LvhB2
Cell entry
HtpB
C3b
CR1
CD35
CR3
CD11b
CD18
C3bi
Phagocytosis
Dot/Icm secretion system
Complement and coagulation cascades
VipA
VipD
VipE
Inhibition of lysosomal fusion
RalF
Arf1
DrrA/SidM
Rab1
LidA
PI(4)P
SidC
ER-recruitment
SdcA
Vacuolar remodeling
Recruited host proteins
Sec22b
Sar1
Cdc48/p97
+u
ER surface target proteins
Mitochondrial apoptptic pathway
Apaf-1
CytC
CASP9
CASP3
CASP8
Apoptosis
Host cell suvival
Intracellular replication
SdhA
Inhibition of caspase-dependent/independent pathways
Pro-apoptotic family proteins
SidF
BNIP3
Bcl-rambo
LegK1
+p
p100
IκB
DNA
Anti-apoptotic proteins
SdbA
Sustained activation
NF-κB
LubX
+u
Clk1
Serine/arginine-rich proteins
Lgt1/2/3
Hbs1
eEF1A
HSF1
Hsp genes
Hsp70
Intracellular replication
SidI
eEF1Bγ
Arrest of protein synthesis
LepA
LepB
Host cell death
Evasion
(Non-lytic release from protozoa)
FlaA
Naip5
CASP7
IPAF
ASC
CASP1
IL-18
IL-1β
TLR5
LPS
TLR2
MyD88
CD14
TLR4
Toll-like receptor signaling pathway
TNFα
IL-1β
IL-8
IL-6
MIP-2/KC
IL-12
Proinflammatory response
Chemoattraction
05134 1/18/16
(c) Kanehisa Laboratories

GLIOMA mmu05214

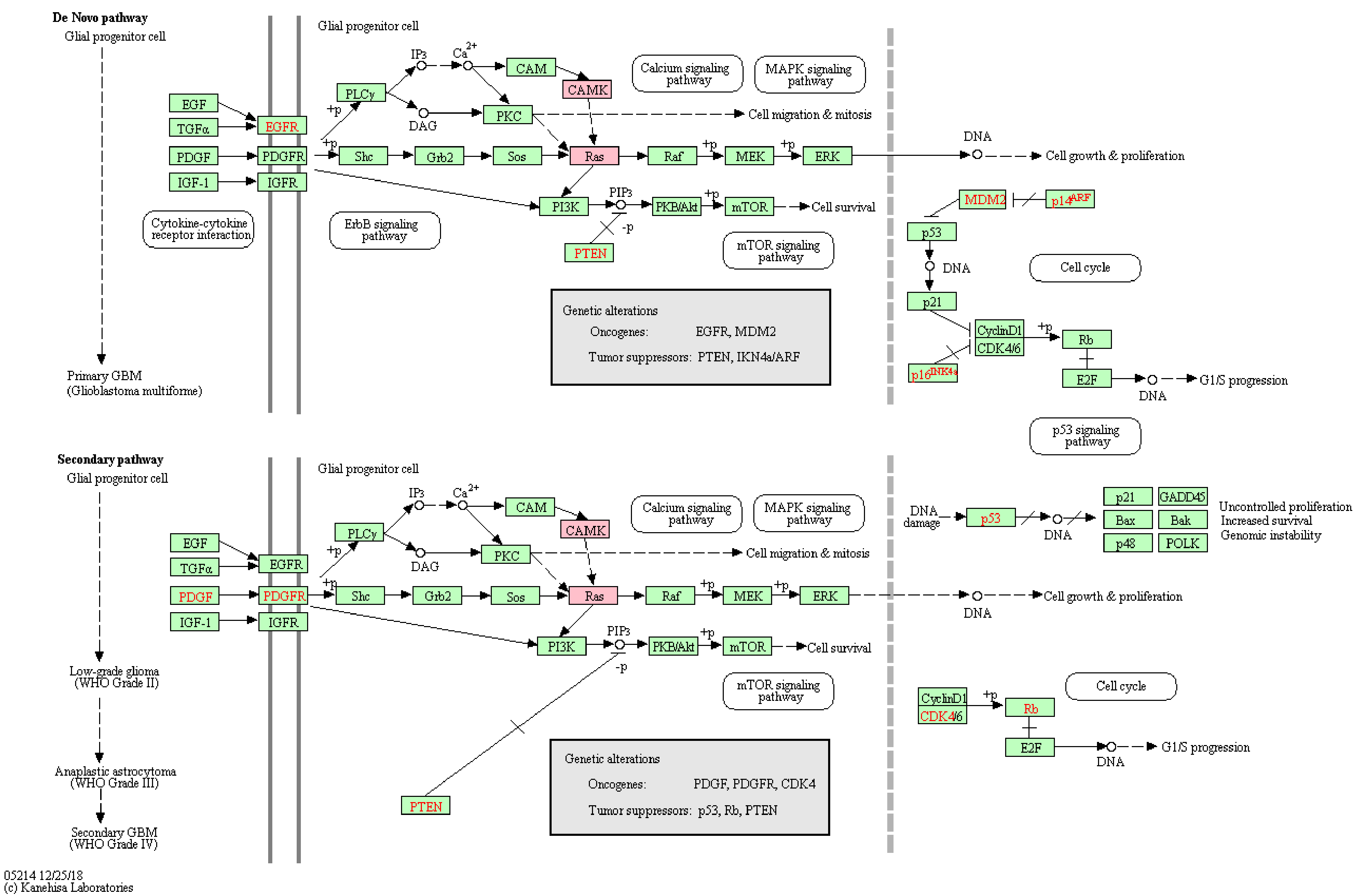

mmu04720

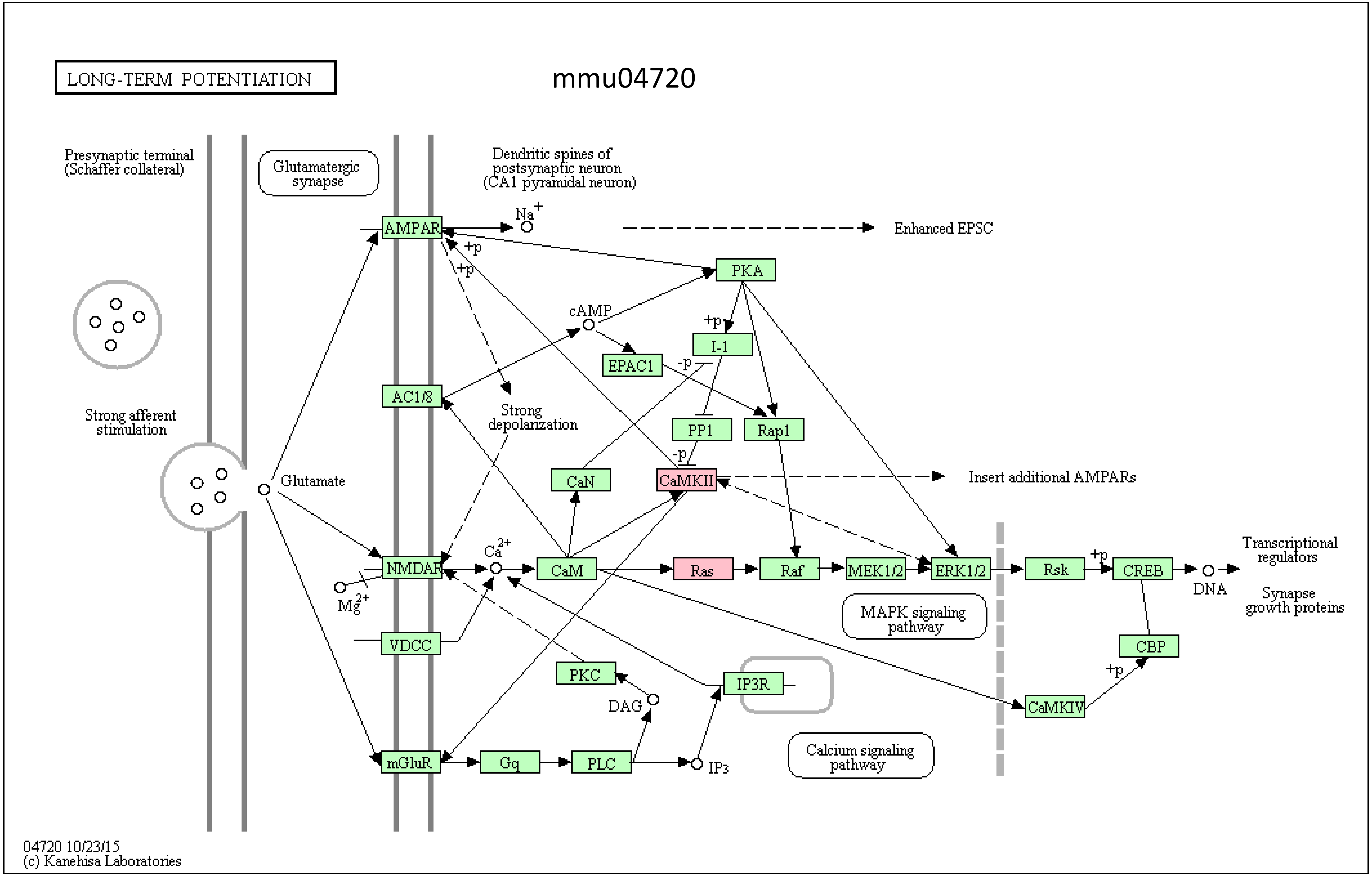

LONG-TERM POTENTIATION
Presynaptic terminal (Schaffer collateral)
Glutamatergic synapse
Dendritic spines of postsynaptic neuron (CA1 pyramidal neuron)
AMPAR
Na+
Enhanced EPSC
+p
+p
PKA
cAMP
+p
I-1
EPAC1
-p
AC1/8
Strong depolarization
PP1
Rap1
Strong afferent stimulation
-p
CaN
CaMKII
Insert additional AMPARs
Glutamate
NMDAR
Ca2+
CaM
Ras
Raf
MEK1/2
ERK1/2
Rsk
+p
CREB
DNA
Transcriptional regulators
Synapse growth proteins
Mg2+
MAPK signaling pathway
VDCC
CBP
PKC
IP3R
+p
DAG
CaMKIV
Calcium signaling pathway
mGluR
Gq
PLC
IP3
04720 10/23/15
(c) Kanehisa Laboratories

mmu04921

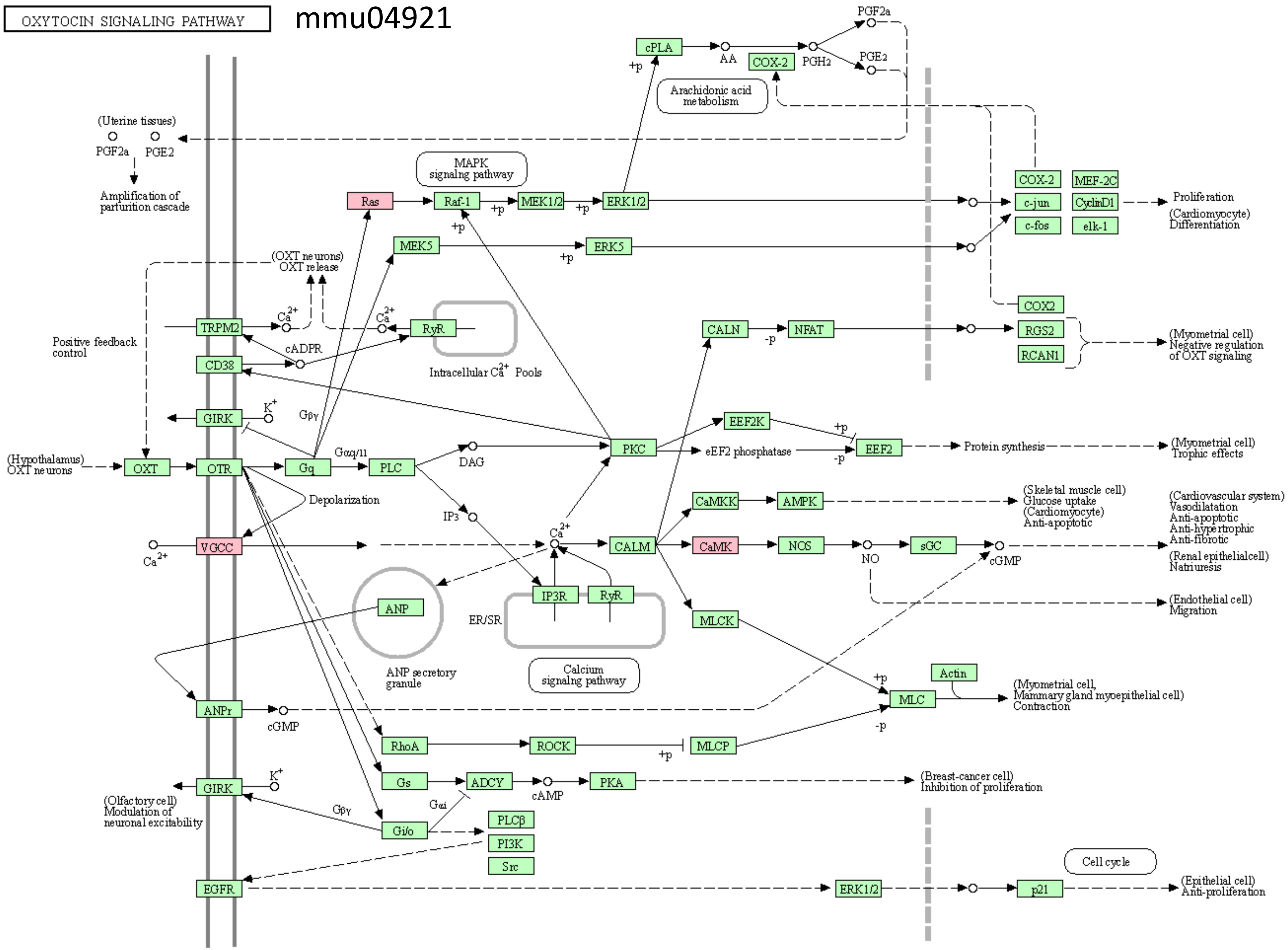

(c) Kanehisa Laboratories

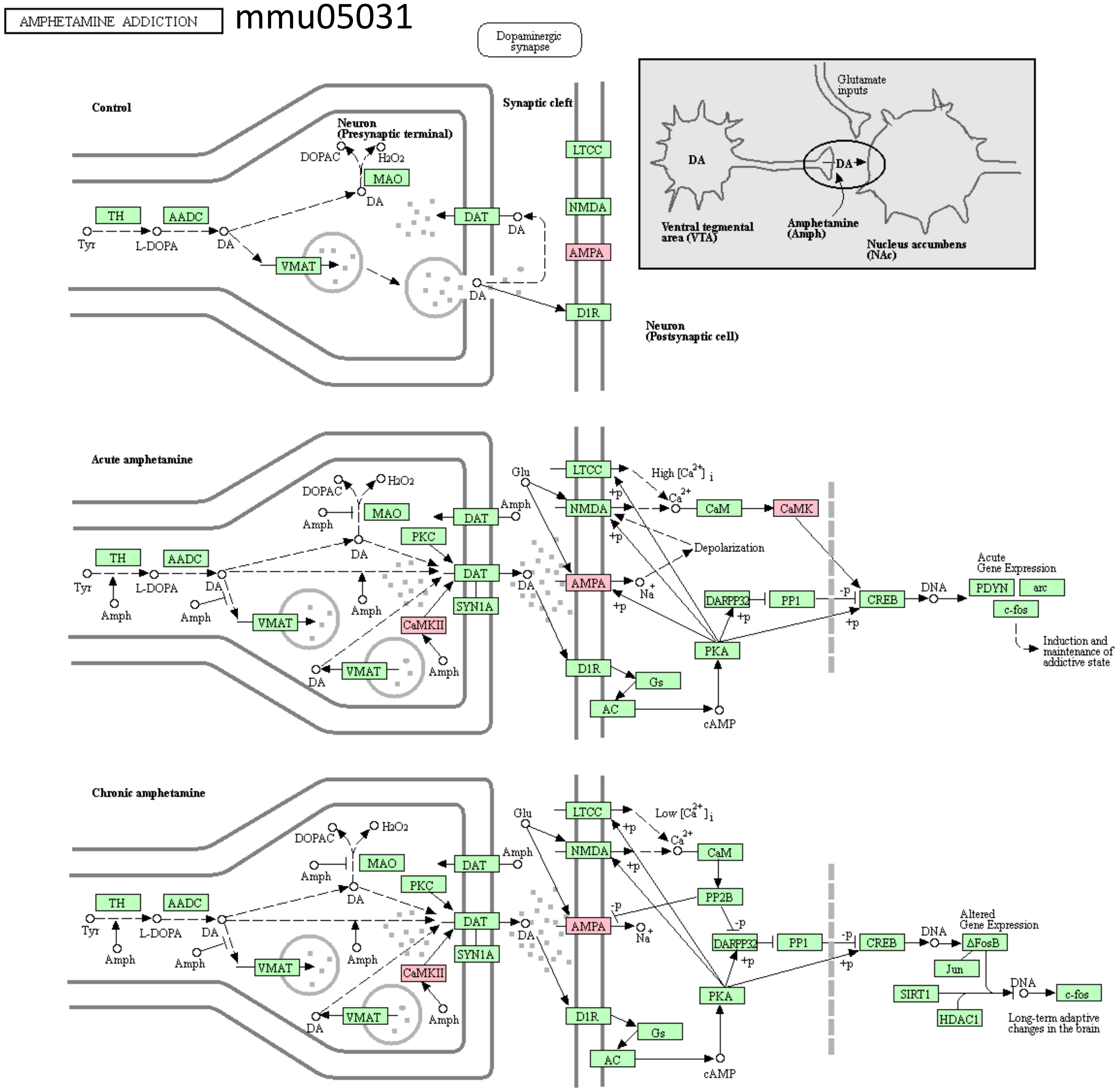

05031 10/28/19

(c) Kanehisa Laboratories

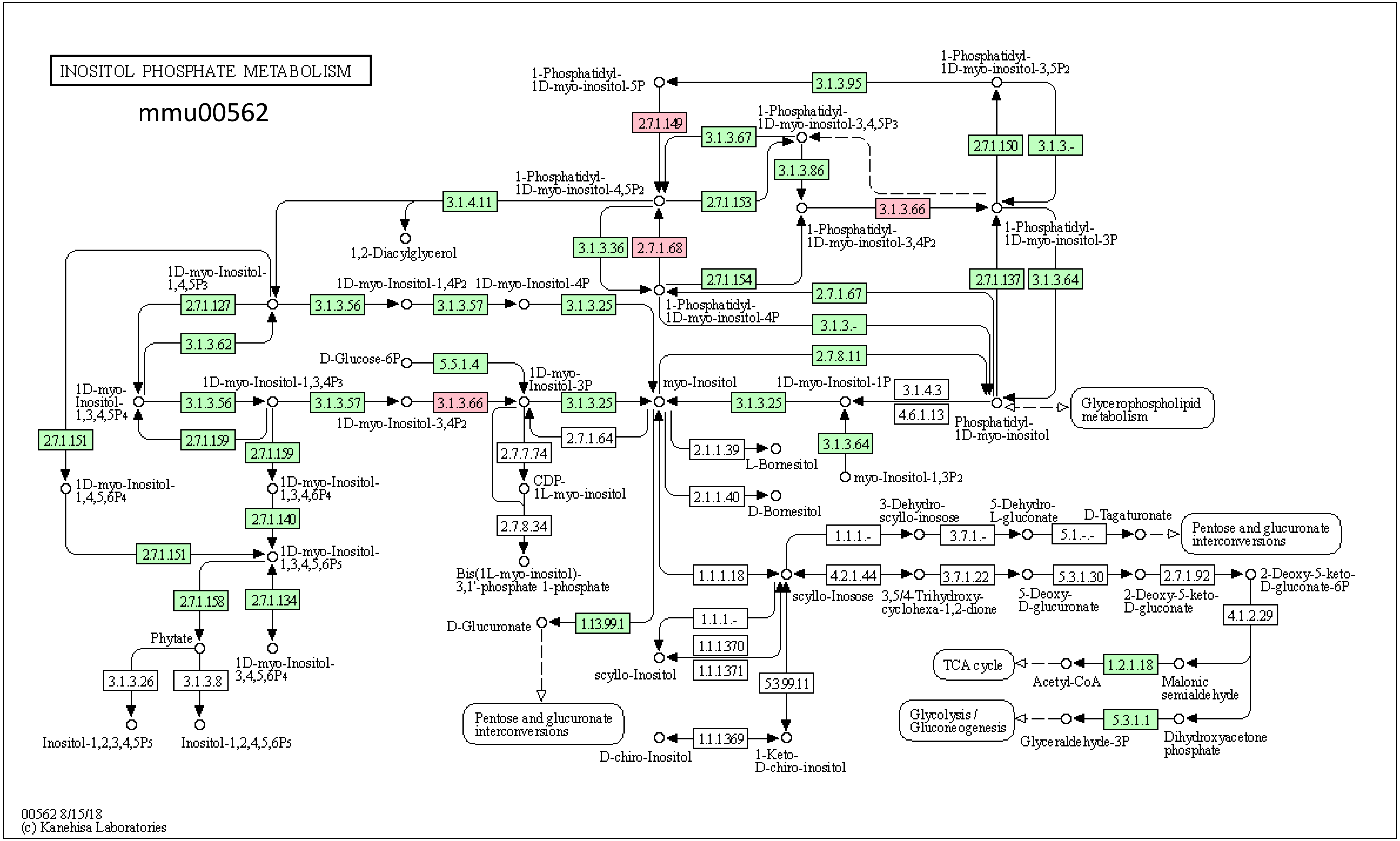
INOSITOL PHOSPHATE METABOLISM
mmu00562
1-Phosphatidyl-1D-myo-inositol-5P
1-Phosphatidyl-1D-myo-inositol-3,5P2
1-Phosphatidyl-1D-myo-inositol-3,4,5P3
1-Phosphatidyl-1D-myo-inositol-4,5P2
1-Phosphatidyl-1D-myo-inositol-3,4P2
1-Phosphatidyl-1D-myo-inositol-3P
1-Phosphatidyl-1D-myo-inositol-4P
1,2-Diacylglycerol
1D-myo-Inositol-1,4,5P3
1D-myo-Inositol-1,4P2
1D-myo-Inositol-4P
D-Glucose-6P
1D-myo-Inositol-3P
myo-Inositol
1D-myo-Inositol-1P
Phosphatidyl-1D-myo-inositol
Glycerophospholipid metabolism
1D-myo-Inositol-1,3,4,5P4
1D-myo-Inositol-1,3,4P3
1D-myo-Inositol-3,4P2
CDP-1L-myo-inositol
Bis(1L-myo-inositol)-3,1'-phosphate 1-phosphate
L-Bornesitol
D-Bornesitol
myo-Inositol-1,3P2
1D-myo-Inositol-1,4,5,6P4
1D-myo-Inositol-1,3,4,6P4
1D-myo-Inositol-1,3,4,5,6P5
Phytate
1D-myo-Inositol-3,4,5,6P4
Inositol-1,2,3,4,5P5
Inositol-1,2,4,5,6P5
D-Glucuronate
Pentose and glucuronate interconversions
3-Dehydro-scyllo-inosose
5-Dehydro-L-gluconate
D-Tagaturonate
scyllo-Inosose
3,5/4-Trihydroxy-cyclohexa-1,2-dione
5-Deoxy-D-glucuronate
2-Deoxy-5-keto-D-gluconate
2-Deoxy-5-keto-D-gluconate-6P
scyllo-Inositol
D-chiro-Inositol
1-Keto-D-chiro-inositol
TCA cycle
Acetyl-CoA
Malonic semialdehyde
Glycolysis / Gluconeogenesis
Glyceraldehyde-3P
Dihydroxyacetone phosphate
3.1.3.95
2.7.1.149
3.1.3.67
3.1.3.86
2.7.1.150
3.1.3.-
3.1.4.11
2.7.1.153
3.1.3.66
3.1.3.36
2.7.1.68
2.7.1.154
2.7.1.67
2.7.1.137
3.1.3.64
2.7.1.127
3.1.3.56
3.1.3.57
3.1.3.25
3.1.3.62
2.7.8.11
5.5.1.4
3.1.4.3
4.6.1.13
2.7.1.151
2.7.1.159
2.7.1.64
2.7.7.74
2.1.1.39
2.1.1.40
2.7.1.140
2.7.8.34
1.1.1.-
3.7.1.-
5.1.-.-
1.1.1.18
4.2.1.44
3.7.1.22
5.3.1.30
2.7.1.92
2.7.1.158
2.7.1.134
1.13.99.1
1.1.1.370
1.1.1.371
5.3.99.11
4.1.2.29
1.2.1.18
5.3.1.1
3.1.3.26
3.1.3.8
1.1.1.369
00562 8/15/18
(c) Kanehisa Laboratories

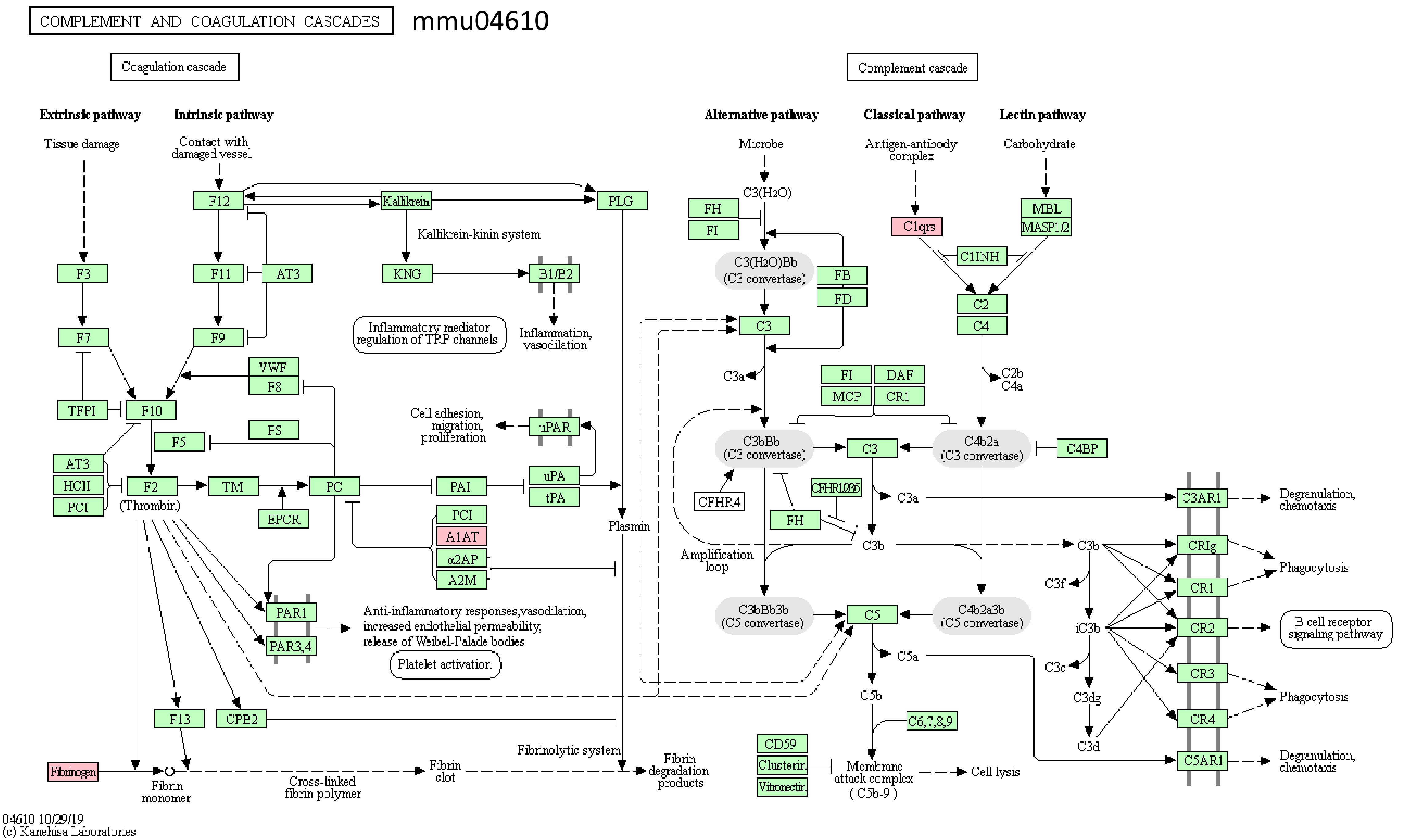
COMPLEMENT AND COAGULATION CASCADES
mmu04610
Coagulation cascade
Complement cascade
Extrinsic pathway
Intrinsic pathway
Alternative pathway
Classical pathway
Lectin pathway
Tissue damage
Contact with damaged vessel
Microbe
Antigen-antibody complex
Carbohydrate
F12
Kallikrein
PLG
Kallikrein-kinin system
F3
F11
AT3
KNG
B1/B2
F7
F9
Inflammatory mediator regulation of TRP channels
Inflammation, vasodilation
VWF
F8
TFPI
F10
PS
F5
Cell adhesion, migration, proliferation
uPAR
AT3
HCII
PCI
F2 (Thrombin)
TM
PC
PAI
uPA
tPA
EPCR
PCI
A1AT
α2AP
A2M
Plasmin
PAR1
PAR3,4
Anti-inflammatory responses,vasodilation, increased endothelial permeability, release of Weibel-Palade bodies
Platelet activation
F13
CPB2
Fibrinolytic system
Fibrinogen
Fibrin monomer
Cross-linked fibrin polymer
Fibrin clot
Fibrin degradation products
C3(H2O)
FH
FI
C3(H2O)Bb (C3 convertase)
FB
FD
C3
C1qrs
MBL
MASP1/2
C1INH
C2
C4
C3a
C2b
C4a
FI
DAF
MCP
CR1
C3bBb (C3 convertase)
C3
C4b2a (C3 convertase)
C4BP
CFHR4
CFHR1/2/5
FH
C3a
C3b
Amplification loop
C3b
C3f
iC3b
C3c
C3dg
C3d
C3bBb3b (C5 convertase)
C5
C4b2a3b (C5 convertase)
C5a
C5b
C6,7,8,9
CD59
Clusterin
Vitronectin
Membrane attack complex (C5b-9)
Cell lysis
C3AR1
CRIg
CR1
CR2
CR3
CR4
C5AR1
Degranulation, chemotaxis
Phagocytosis
B cell receptor signaling pathway
Phagocytosis
Degranulation, chemotaxis
04610 10/29/19
(c) Kanehisa Laboratories

mmu04912

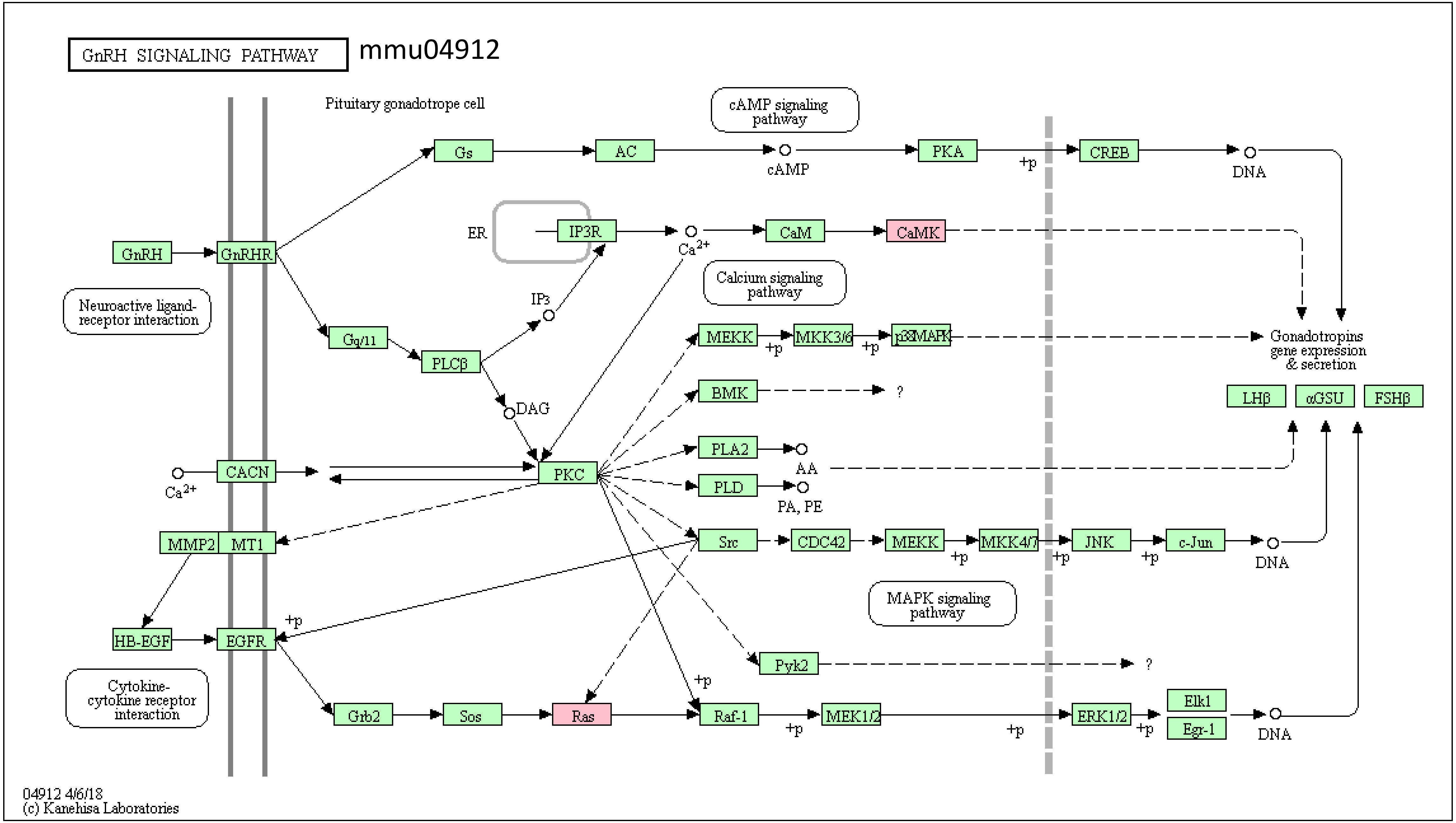
GnRH SIGNALING PATHWAY
Pituitary gonadotrope cell
cAMP signaling pathway
Gs
AC
cAMP
PKA
+p
CREB
DNA
ER
IP3R
Ca2+
CaM
CaMK
GnRH
GnRHR
Neuroactive ligand-receptor interaction
IP3
Calcium signaling pathway
Gq/11
PLCβ
MEKK
MKK3/6
p38MAPK
+p
Gonadotropins gene expression & secretion
DAG
BMK
?
LHβ
αGSU
FSHβ
PLA2
AA
CACN
Ca2+
PKC
PLD
PA, PE
MMP2
MT1
Src
CDC42
MEKK
MKK4/7
JNK
c-Jun
DNA
MAPK signaling pathway
HB-EGF
EGFR
+p
Pyk2
?
Cytokine-cytokine receptor interaction
Grb2
Sos
Ras
Raf-1
MEK1/2
ERK1/2
Elk1
Egr-1
DNA
04912 4/6/18
(c) Kanehisa Laboratories
