# Supplementary material for: The Impacts of Surgery and Intracerebral Electrodes in C57BL/6J Mouse Kainate Model of Epileptogenesis: Seizure Threshold, Proteomics, and Cytokine Profiles
Source: Front Neurol. 2021 Jul 12;12:625017. doi: 10.3389/fneur.2021.625017 (PMC8312573; doi:10.3389/fneur.2021.625017)
Supplement: Supplementary Figure 3 — The schematic diagrams of KEGG pathways (KA: surgery vs. no surgery). [file Data_Sheet_3.pdf]

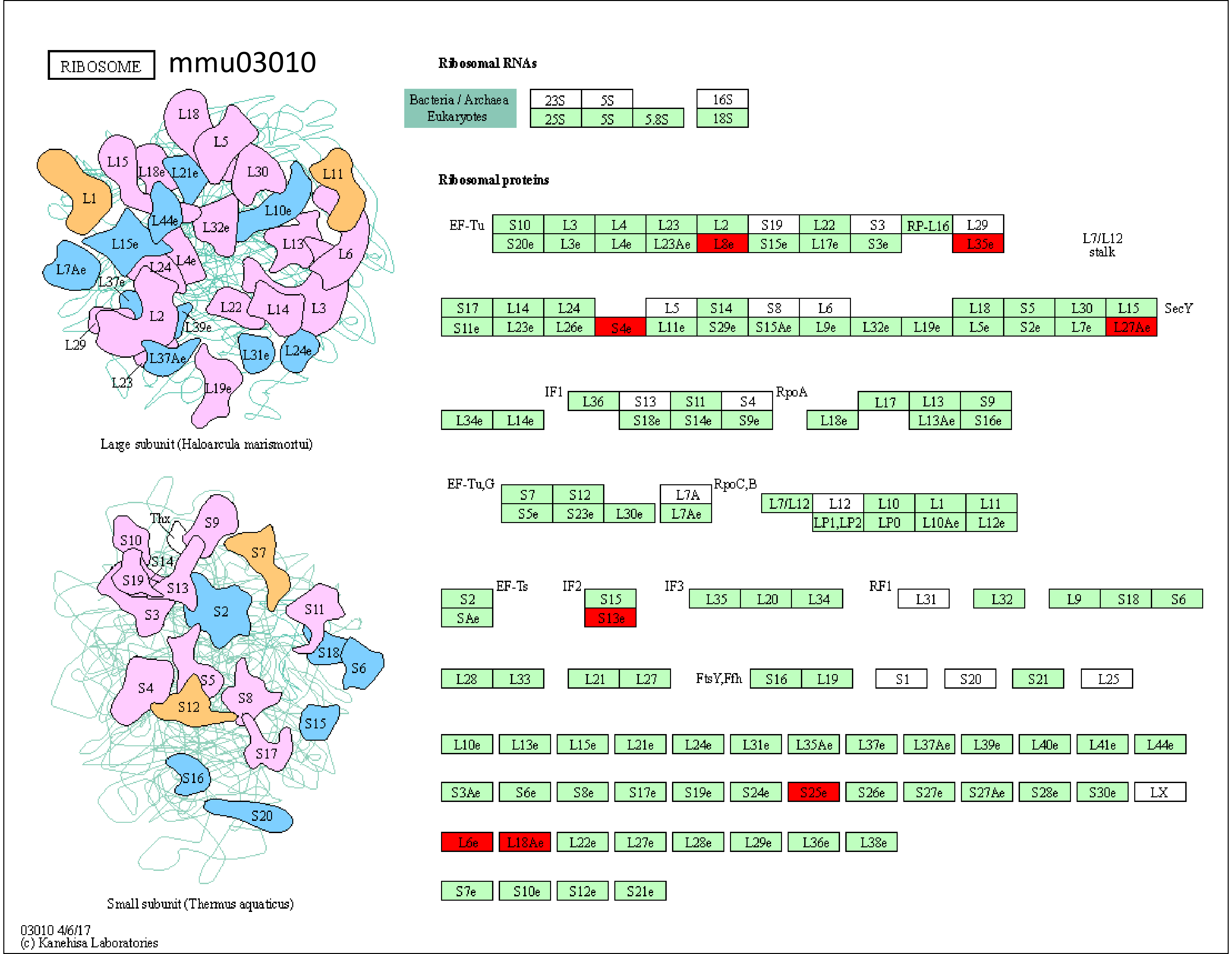
RIBOSOME
mmu03010
Ribosomal RNAs
Bacteria / Archaea
Eukaryotes
23S
5S
16S
25S
5S
5.8S
18S
Ribosomal proteins
EF-Tu
S10 L3 L4 L23 L2 S19 L22 S3 RP-L16 L29
S20e L3e L4e L23Ae L8e S15e L17e S3e L35e
L7/L12 stalk
S17 L14 L24 L5 S14 S8 L6 L18 S5 L30 L15
S11e L23e L26e S4e L11e S29e S15Ae L9e L32e L19e L5e S2e L7e L27Ae
SecY
IF1
L36 S13 S11 S4
RpoA
L17 L13 S9
L34e L14e S18e S14e S9e L18e L13Ae S16e
EF-Tu,G
S7 S12 L7A
S5e S23e L30e L7Ae
RpoC,B
L7/L12 L12 L10 L1 L11
LP1,LP2 LP0 L10Ae L12e
EF-Ts
S2
SAe
IF2
S15
S13e
IF3
L35 L20 L34
RF1
L31 L32 L9 S18 S6
L28 L33 L21 L27
FtsY,Ffh
S16 L19 S1 S20 S21 L25
L10e L13e L15e L21e L24e L31e L35Ae L37e L37Ae L39e L40e L41e L44e
S3Ae S6e S8e S17e S19e S24e S25e S26e S27e S27Ae S28e S30e LX
L6e L18Ae L22e L27e L28e L29e L36e L38e
S7e S10e S12e S21e
L18 L5 L15 L18e L21e L30 L11 L1 L44e L10e L32e L15e L13 L6 L7Ae L24 L4e L37e L22 L14 L3 L2 L39e L29 L37Ae L31e L24e L23 L19e
Large subunit (Haloarcula marismortui)
Thx S9 S10 S14 S7 S19 S13 S2 S3 S11 S18 S6 S4 S5 S12 S8 S15 S17 S16 S20
Small subunit (Thermus aquaticus)
03010 4/6/17
(c) Kanehisa Laboratories

# mmu05031

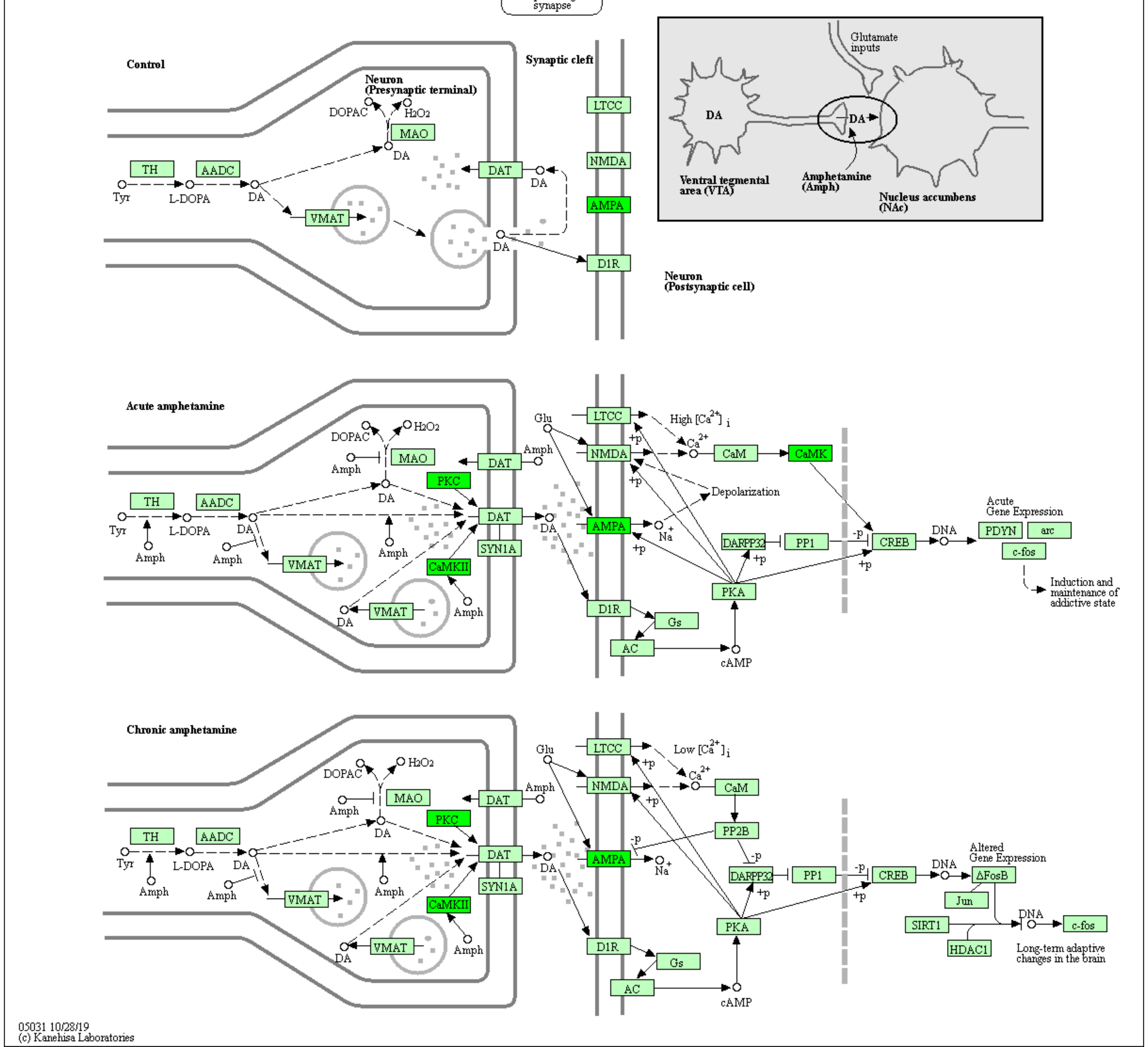
AMPHETAMINE ADDICTION
Dopaminergic synapse
Control
Neuron (Presynaptic terminal)
Synaptic cleft
DOPAC
H2O2
MAO
DA
TH
AADC
Tyr
L-DOPA
VMAT
DAT
LTCC
NMDA
AMPA
D1R
Glutamate inputs
Ventral tegmental area (VTA)
Amphetamine (Amph)
Nucleus accumbens (NAc)
Neuron (Postsynaptic cell)
Acute amphetamine
Amph
PKC
SYN1A
CaMKII
Glu
High [Ca2+]i
Ca2+
CaM
CaMK
Depolarization
Na+
DARPP32
PP1
CREB
DNA
Acute Gene Expression
PDYN
arc
c-fos
Induction and maintenance of addictive state
PKA
Gs
AC
cAMP
Chronic amphetamine
Low [Ca2+]i
PP2B
Altered Gene Expression
ΔFosB
Jun
SIRT1
HDAC1
Long-term adaptive changes in the brain
05031 10/28/19
(c) Kanehisa Laboratories

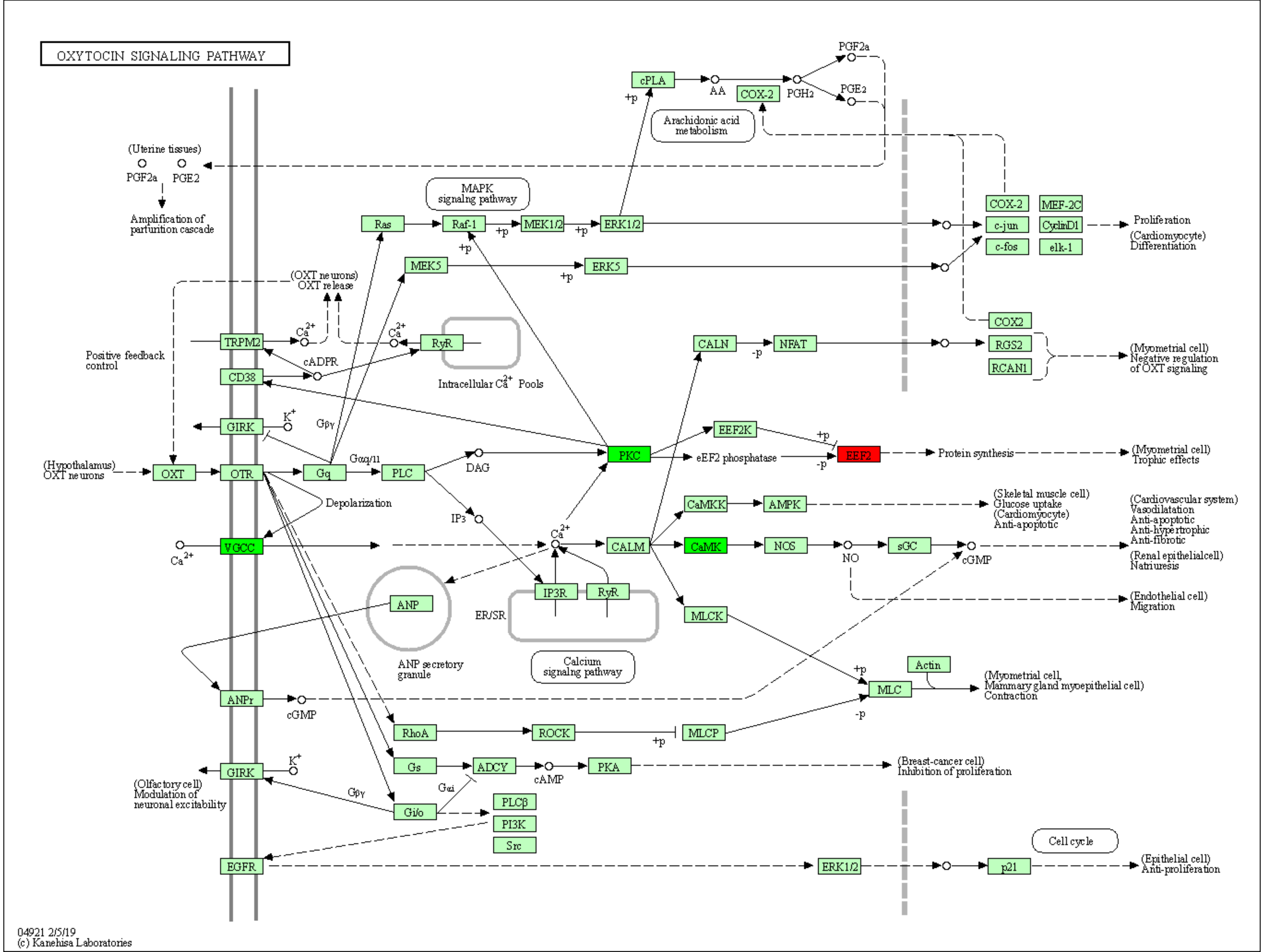
OXYTOCIN SIGNALING PATHWAY
(Uterine tissues)
PGF2a
PGE2
Amplification of parturition cascade
MAPK signaling pathway
Ras
Raf-1
MEK1/2
ERK1/2
cPLA
AA
COX-2
PGH2
PGF2a
PGE2
Arachidonic acid metabolism
MEK5
ERK5
(OXT neurons) OXT release
TRPM2
CD38
cADPR
RyR
Intracellular Ca2+ Pools
Positive feedback control
GIRK
Gβγ
(Hypothalamus) OXT neurons
OXT
OTR
Gq
Gαq/11
PLC
DAG
PKC
IP3
Depolarization
VGCC
CALM
ANP
ANP secretory granule
IP3R
RyR
ER/SR
Calcium signaling pathway
ANPr
cGMP
RhoA
ROCK
MLCP
Gs
ADCY
cAMP
PKA
Gαi
GIRK
Gβγ
Gi/o
PLCβ
PI3K
Src
(Olfactory cell) Modulation of neuronal excitability
EGFR
ERK1/2
p21
Cell cycle
(Epithelial cell) Anti-proliferation
COX-2
c-jun
c-fos
MEF-2C
CyclinD1
elk-1
Proliferation
(Cardiomyocyte) Differentiation
CALN
NFAT
COX2
RGS2
RCAN1
(Myometrial cell) Negative regulation of OXT signaling
EEF2K
eEF2 phosphatase
EEF2
Protein synthesis
(Myometrial cell) Trophic effects
CaMKK
AMPK
(Skeletal muscle cell) Glucose uptake (Cardiomyocyte) Anti-apoptotic
CaMK
NOS
NO
sGC
cGMP
(Cardiovascular system) Vasodilatation Anti-apoptotic Anti-hypertrophic Anti-fibrotic
(Renal epithelial cell) Natriuresis
(Endothelial cell) Migration
MLCK
Actin
MLC
(Myometrial cell, Mammary gland myoepithelial cell) Contraction
(Breast-cancer cell) Inhibition of proliferation
04921 2/5/19
(c) Kanehisa Laboratories

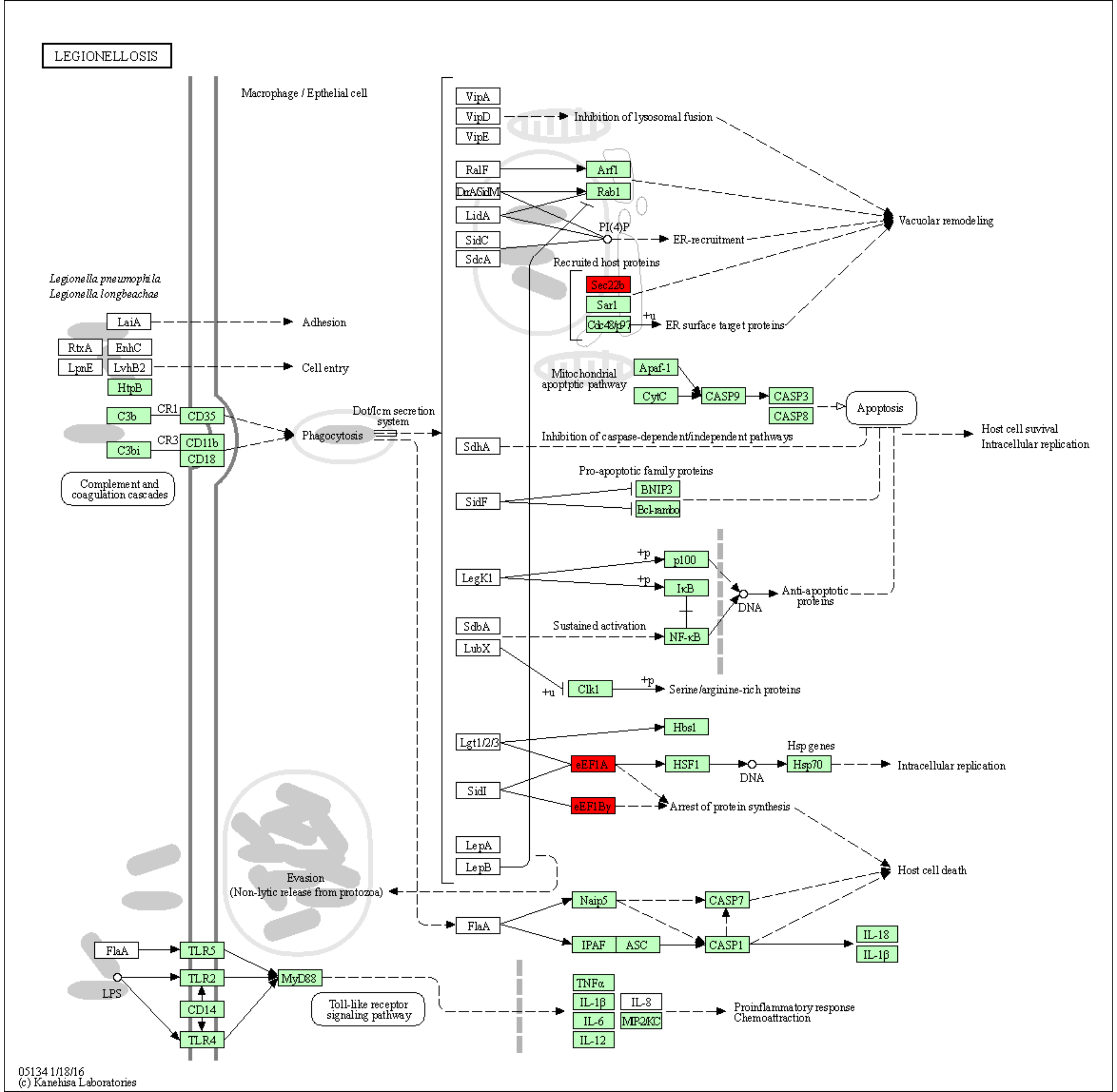
LEGIONELLOSIS
Macrophage / Epthelial cell
Legionella pneumophila
Legionella longbeachae
LaiA
Adhesion
RtxA
EnhC
LpnE
LvhB2
Cell entry
HtpB
C3b
CR1
CD35
C3bi
CR3
CD11b
CD18
Phagocytosis
Dot/Icm secretion system
Complement and coagulation cascades
VipA
VipD
VipE
Inhibition of lysosomal fusion
RalF
Arf1
DrrA/SidM
Rab1
LidA
PI(4)P
SidC
ER-recruitment
SdcA
Recruited host proteins
Sec22b
Sar1
Cdc48/p97
+u
ER surface target proteins
Vacuolar remodeling
Mitochondrial apoptptic pathway
Apaf-1
CytC
CASP9
CASP3
CASP8
Apoptosis
Host cell survival
Intracellular replication
SdhA
Inhibition of caspase-dependent/independent pathways
Pro-apoptotic family proteins
SidF
BNIP3
Bcl-rambo
LegK1
+p
p100
IκB
DNA
Anti-apoptotic proteins
SdbA
Sustained activation
NF-κB
LubX
+u
Clk1
Serine/arginine-rich proteins
Lgt1/2/3
Hbs1
eEF1A
HSF1
Hsp genes
Hsp70
Intracellular replication
SidI
eEF1Bγ
Arrest of protein synthesis
Host cell death
LepA
LepB
Evasion
(Non-lytic release from protozoa)
FlaA
Naip5
CASP7
IPAF
ASC
CASP1
IL-18
IL-1β
TLR5
TLR2
MyD88
LPS
CD14
TLR4
Toll-like receptor signaling pathway
TNFα
IL-1β
IL-8
IL-6
MIP-2/KC
IL-12
Proinflammatory response
Chemoattraction
05134 1/18/16
(c) Kanehisa Laboratories

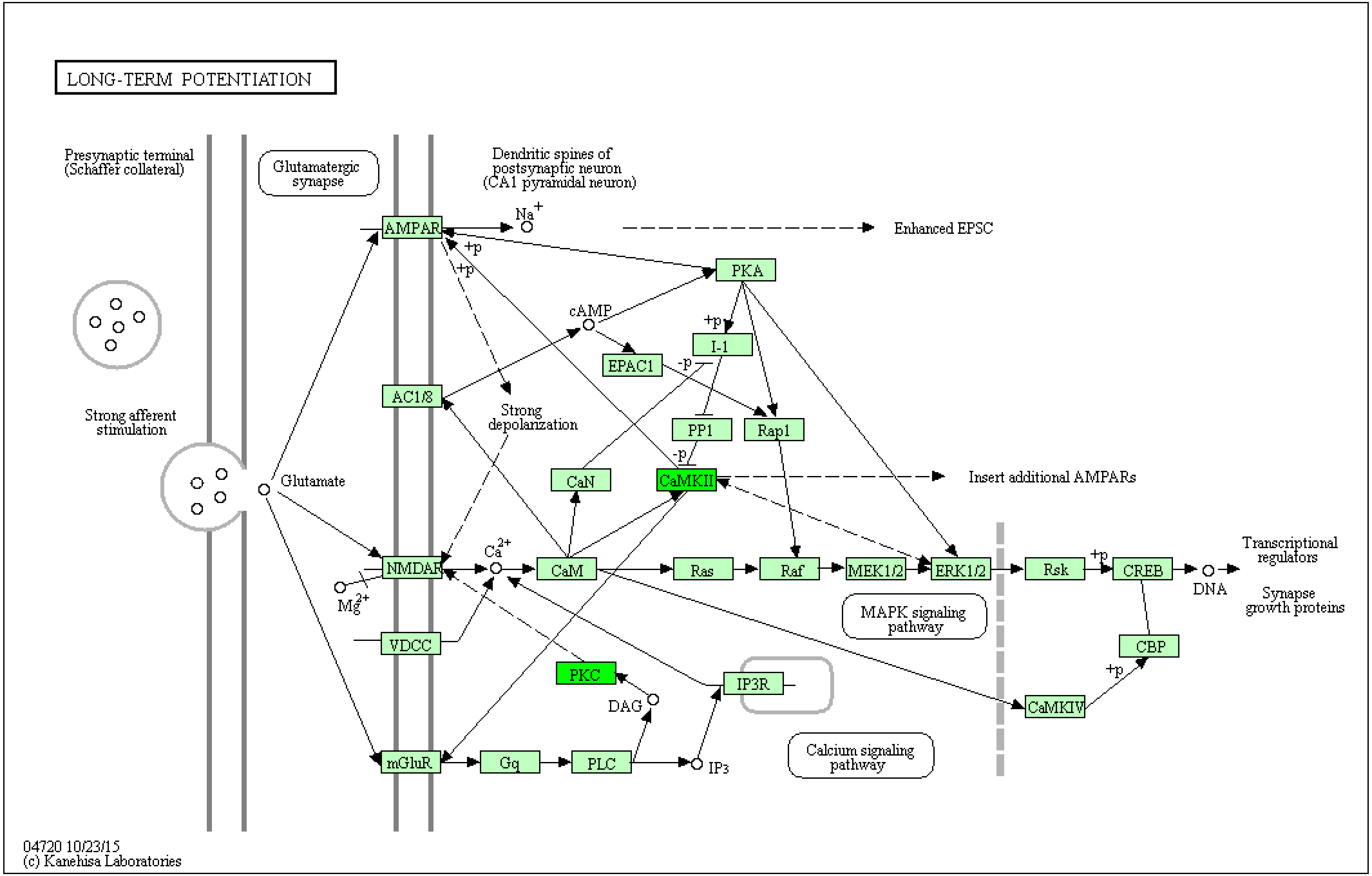
LONG-TERM POTENTIATION
Presynaptic terminal (Schaffer collateral)
Glutamatergic synapse
Dendritic spines of postsynaptic neuron (CA1 pyramidal neuron)
Strong afferent stimulation
Glutamate
AMPAR
Na+
Enhanced EPSC
PKA
+p
cAMP
I-1
EPAC1
-p
AC1/8
Strong depolarization
PP1
Rap1
CaN
CaMKII
Insert additional AMPARs
NMDAR
Ca2+
CaM
Ras
Raf
MEK1/2
ERK1/2
Rsk
CREB
DNA
Transcriptional regulators
Synapse growth proteins
Mg2+
VDCC
MAPK signaling pathway
CBP
PKC
DAG
IP3R
CaMKIV
Calcium signaling pathway
mGluR
Gq
PLC
IP3
04720 10/23/15
(c) Kanehisa Laboratories

Biosynthesis of amino acids
mmu01230.

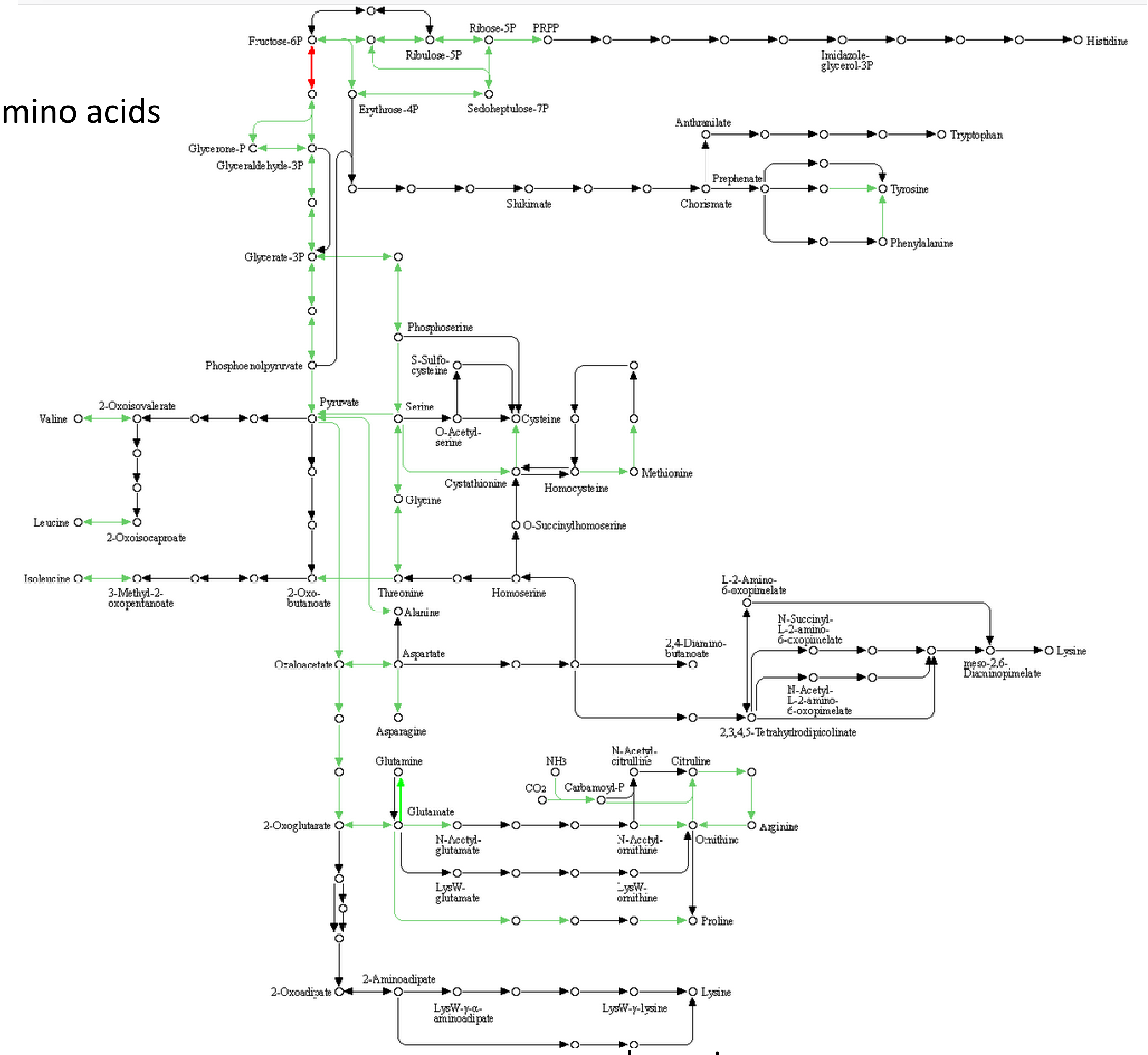


saccharopine

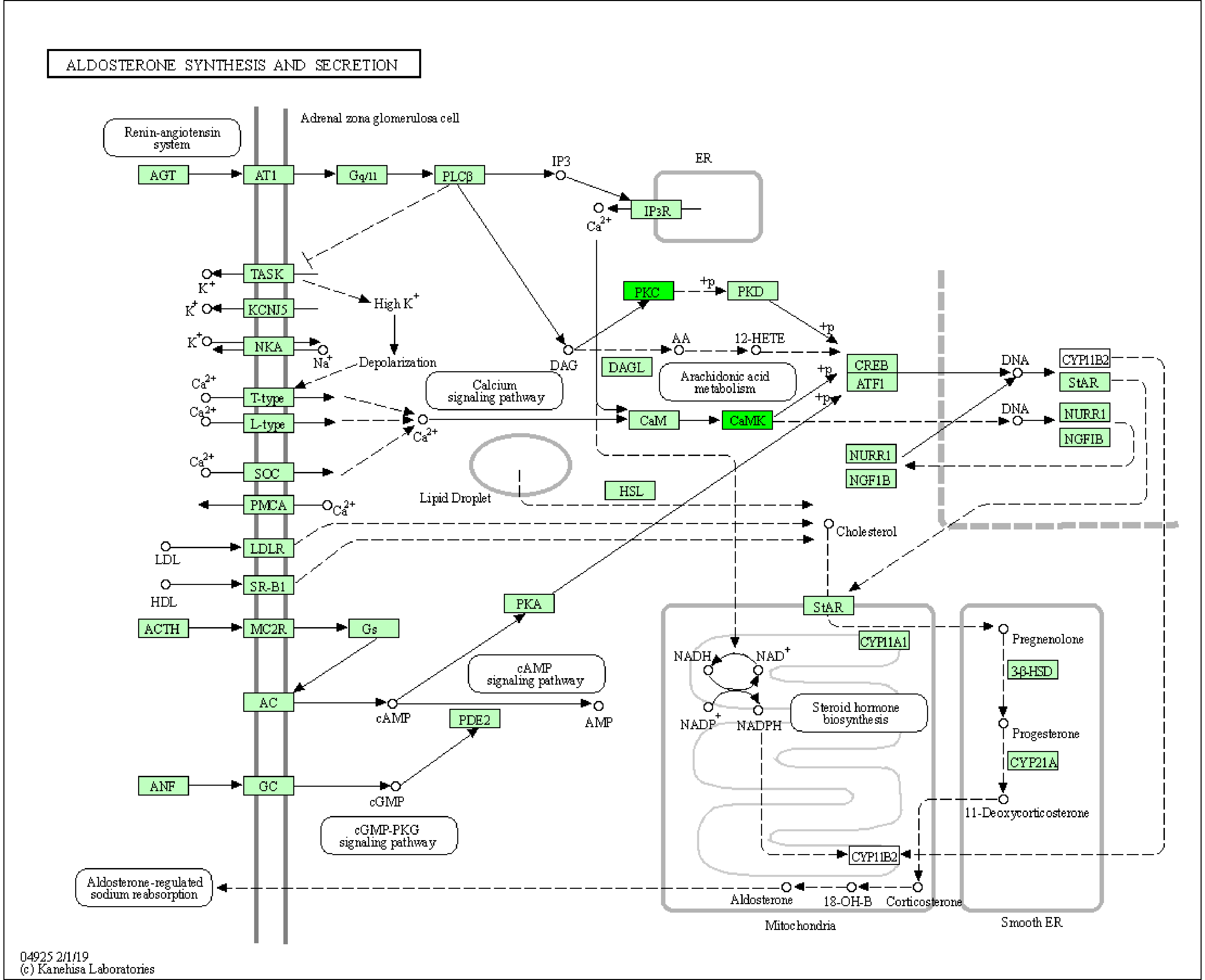

ALDOSTERONE SYNTHESIS AND SECRETION
Adrenal zona glomerulosa cell
Renin-angiotensin system
AGT
AT1
Gq/11
PLCβ
IP3
ER
IP3R
Ca2+
TASK
K+
KCNJ5
High K+
NKA
Na+
Depolarization
DAG
PKC
PKD
+p
AA
12-HETE
DAGL
Arachidonic acid metabolism
CREB
ATF1
DNA
CYP11B2
StAR
NURR1
NGFIB
NGF1B
T-type
L-type
SOC
PMCA
Calcium signaling pathway
CaM
CaMK
Lipid Droplet
HSL
Cholesterol
LDLR
LDL
SR-B1
HDL
ACTH
MC2R
Gs
PKA
AC
cAMP
cAMP signaling pathway
AMP
PDE2
ANF
GC
cGMP
cGMP-PKG signaling pathway
Aldosterone-regulated sodium reabsorption
CYP11A1
NADH
NAD+
NADP+
NADPH
Steroid hormone biosynthesis
Pregnenolone
3β-HSD
Progesterone
CYP21A
11-Deoxycorticosterone
Aldosterone
18-OH-B
Corticosterone
Mitochondria
Smooth ER
04925 2/1/19
(c) Kanehisa Laboratories

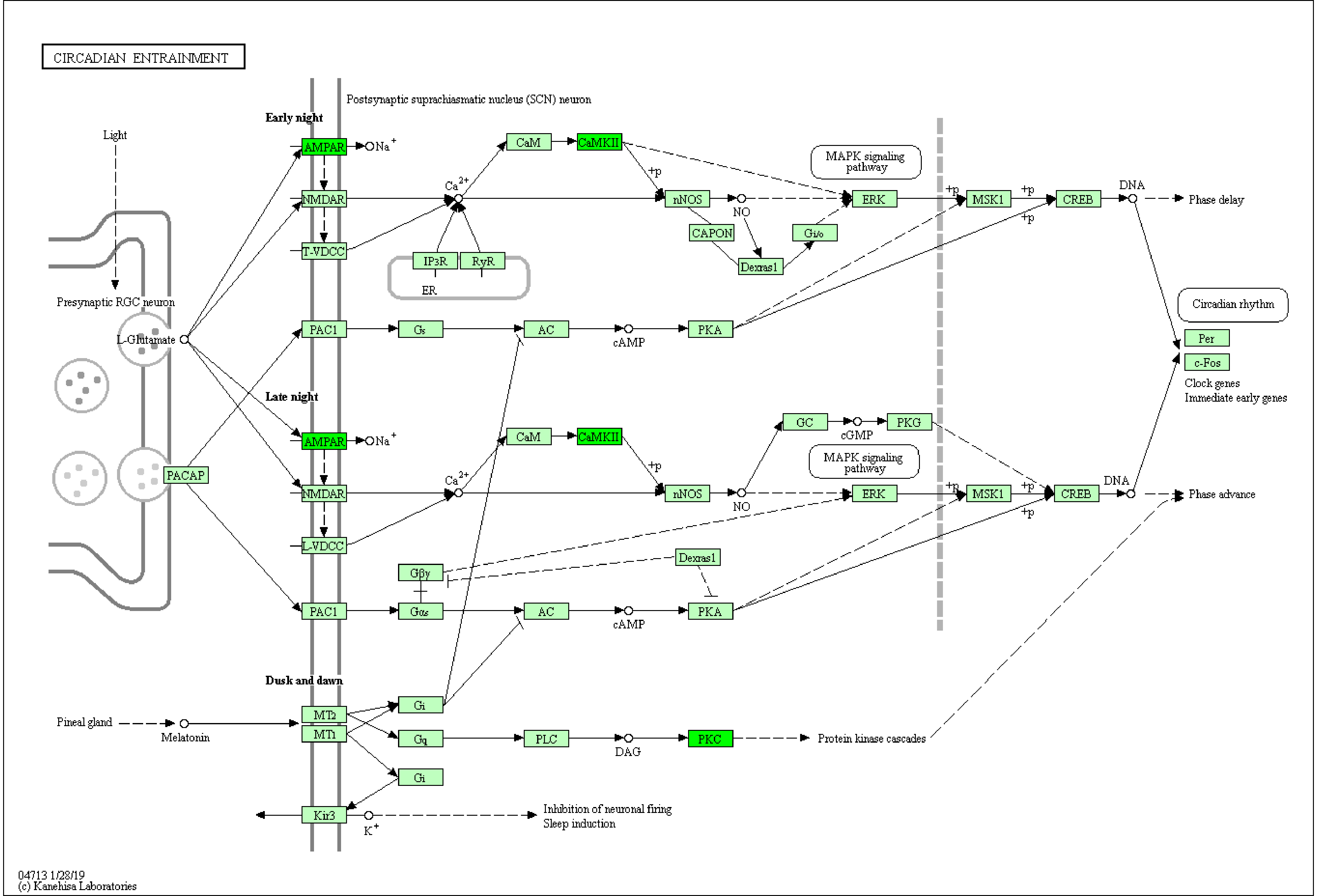
CIRCADIAN ENTRAINMENT
Postsynaptic suprachiasmatic nucleus (SCN) neuron
Early night
Light
Presynaptic RGC neuron
L-Glutamate
AMPAR
Na+
NMDAR
T-VDCC
Ca2+
IP3R
RyR
ER
CaM
CaMKII
+p
nNOS
NO
CAPON
Dexras1
Gi/o
ERK
MAPK signaling pathway
MSK1
CREB
DNA
Phase delay
PAC1
Gs
AC
cAMP
PKA
Circadian rhythm
Per
c-Fos
Clock genes
Immediate early genes
Late night
PACAP
L-VDCC
GC
cGMP
PKG
Phase advance
Gβγ
Gαs
Dusk and dawn
Pineal gland
Melatonin
MT2
MT1
Gi
Gq
PLC
DAG
PKC
Protein kinase cascades
Kir3
K+
Inhibition of neuronal firing
Sleep induction
04713 1/28/19
(c) Kanehisa Laboratories

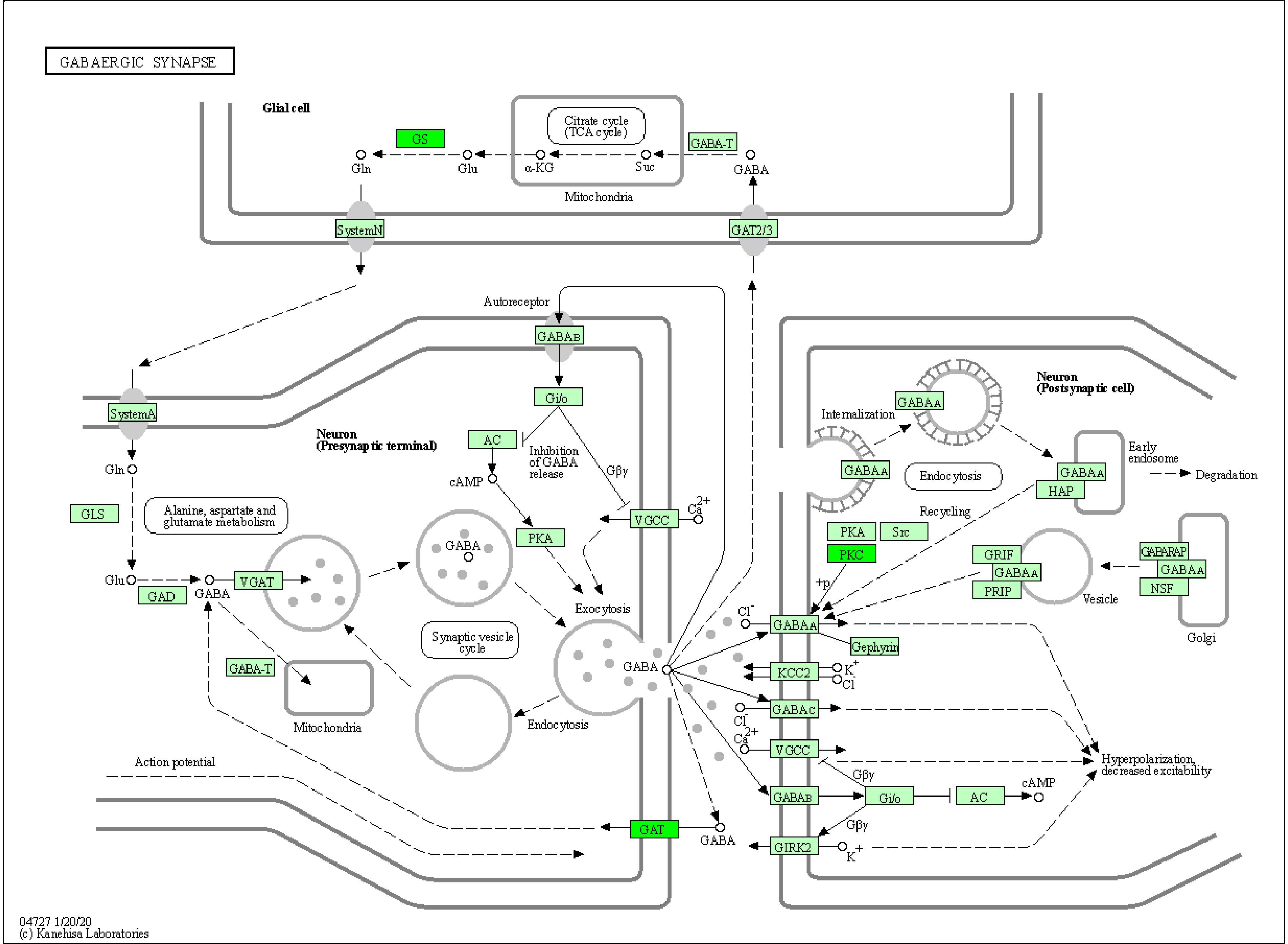
GABAERGIC SYNAPSE
Glial cell
GS
Citrate cycle (TCA cycle)
GABA-T
Gln
Glu
α-KG
Suc
GABA
Mitochondria
SystemN
GAT2/3
Autoreceptor
GABAB
Gi/o
AC
Inhibition of GABA release
Gβγ
cAMP
SystemA
Neuron (Presynaptic terminal)
Gln
GLS
Alanine, aspartate and glutamate metabolism
PKA
VGCC
Ca2+
Glu
GAD
GABA
VGAT
GABA
Exocytosis
Synaptic vesicle cycle
GABA-T
Mitochondria
Endocytosis
GABA
Action potential
GAT
GABA
Neuron (Postsynaptic cell)
Internalization
GABAA
Endocytosis
Early endosome
Degradation
HAP
Recycling
PKA
Src
PKC
+p
GRIF
GABAA
PRIP
Vesicle
GABARAP
NSF
Golgi
Cl-
GABAA
Gephyrin
KCC2
K+
Cl-
GABAC
VGCC
Hyperpolarization, decreased excitability
GABAB
Gi/o
AC
cAMP
GIRK2
04727 1/20/20
(c) Kanehisa Laboratories

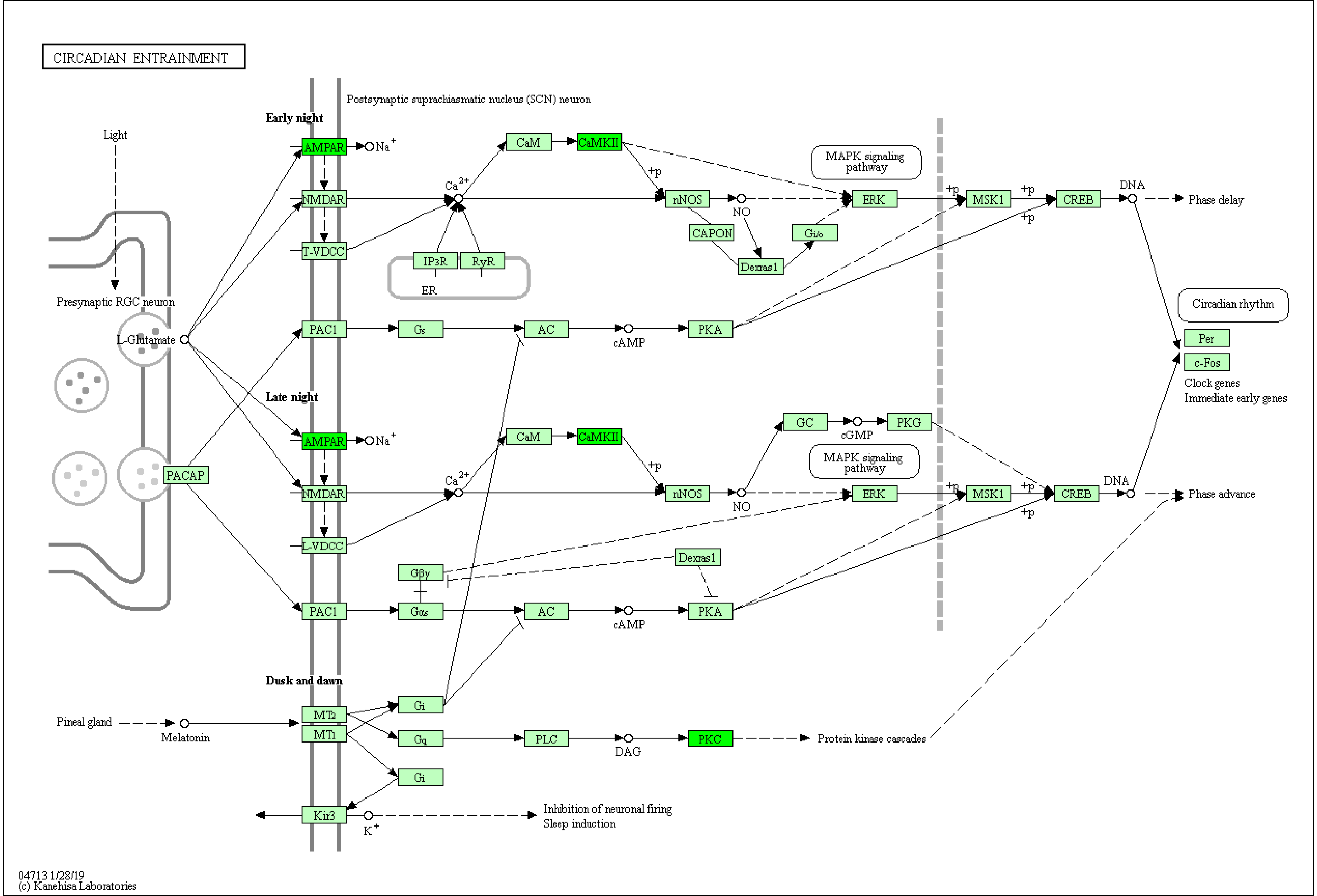
CIRCADIAN ENTRAINMENT
Postsynaptic suprachiasmatic nucleus (SCN) neuron
Early night
Light
Presynaptic RGC neuron
L-Glutamate
AMPAR
Na+
NMDAR
T-VDCC
Ca2+
CaM
CaMKII
+p
nNOS
NO
CAPON
Dexras1
Gi/o
ERK
MAPK signaling pathway
IP3R
RyR
ER
MSK1
CREB
DNA
Phase delay
PAC1
Gs
AC
cAMP
PKA
Circadian rhythm
Per
c-Fos
Clock genes
Immediate early genes
Late night
PACAP
GC
cGMP
PKG
L-VDCC
Gβγ
Gαs
Phase advance
Dusk and dawn
Pineal gland
Melatonin
MT2
MT1
Gi
Gq
PLC
DAG
PKC
Protein kinase cascades
Kir3
K+
Inhibition of neuronal firing
Sleep induction
04713 1/28/19
(c) Kanehisa Laboratories

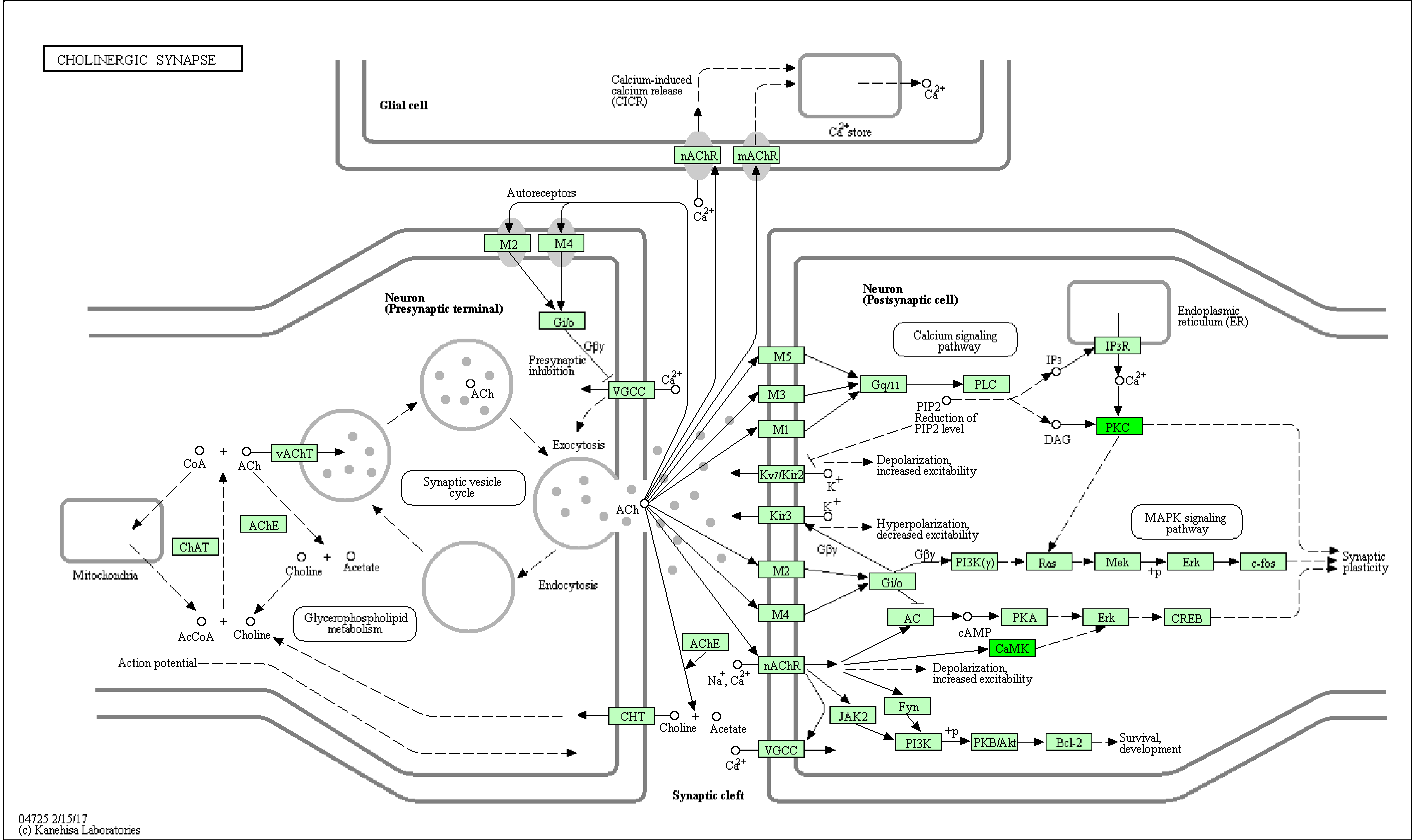
CHOLINERGIC SYNAPSE
Glial cell
Calcium-induced calcium release (CICR)
Ca2+ store
nAChR
mAChR
Autoreceptors
M2
M4
Gi/o
Gβγ
Presynaptic inhibition
VGCC
Neuron (Presynaptic terminal)
ACh
Exocytosis
CoA
vAChT
Synaptic vesicle cycle
AChE
ChAT
Mitochondria
Choline
Acetate
AcCoA
Endocytosis
Glycerophospholipid metabolism
Action potential
CHT
Synaptic cleft
Neuron (Postsynaptic cell)
Calcium signaling pathway
Endoplasmic reticulum (ER)
IP3R
IP3
M5
M3
M1
Gq/11
PLC
PIP2
Reduction of PIP2 level
DAG
PKC
Kv7/Kir2
K+
Depolarization, increased excitability
Kir3
Hyperpolarization, decreased excitability
MAPK signaling pathway
PI3K(γ)
Ras
Mek
Erk
c-fos
+p
Synaptic plasticity
AC
cAMP
PKA
CREB
CaMK
Na+, Ca2+
Fyn
JAK2
PI3K
PKB/Akt
Bcl-2
Survival, development
04725 2/15/17
(c) Kanehisa Laboratories

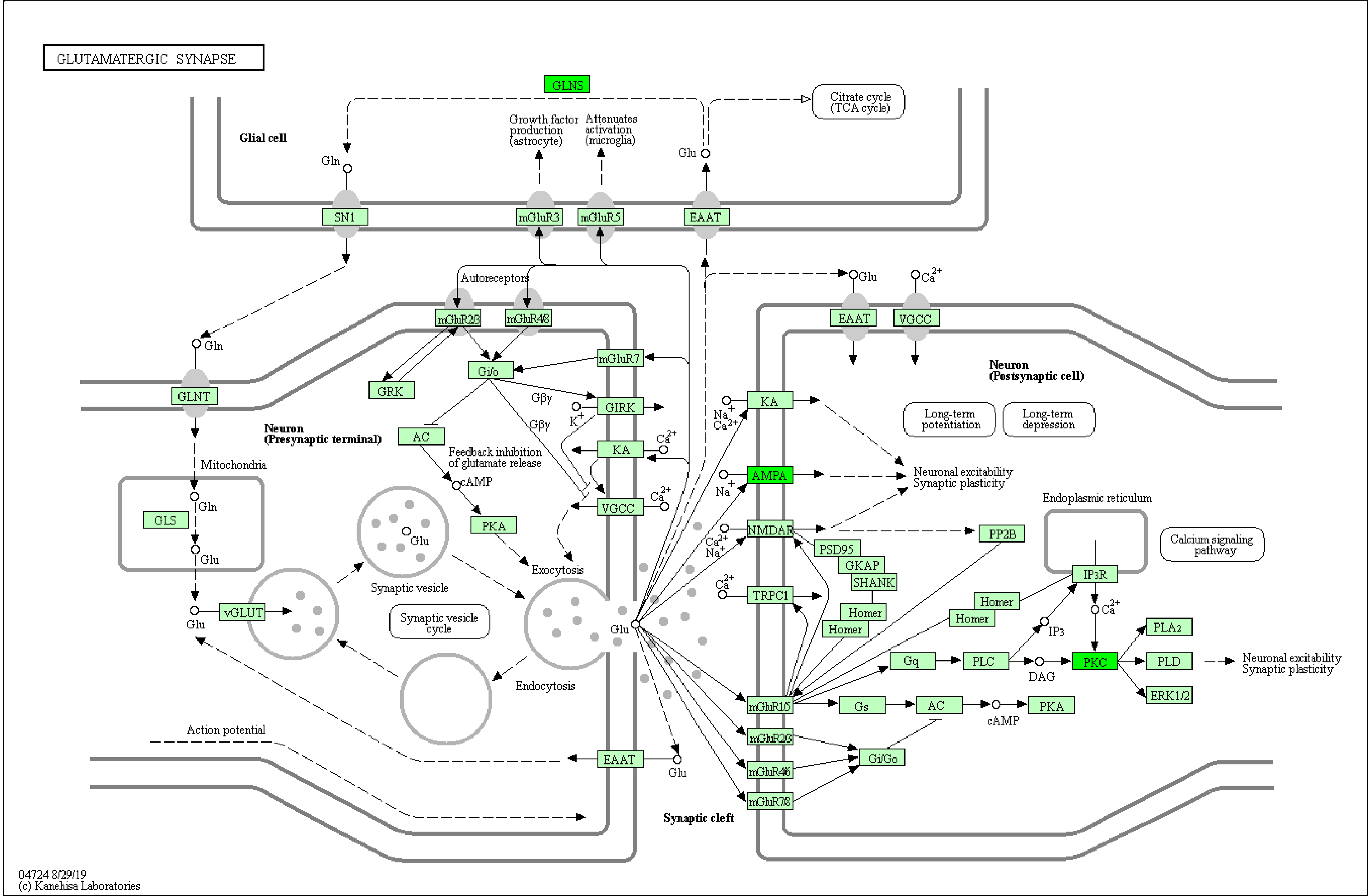
GLUTAMATERGIC SYNAPSE
GLNS
Citrate cycle (TCA cycle)
Glial cell
Growth factor production (astrocyte)
Attenuates activation (microglia)
Gln
Glu
SN1
mGluR3
mGluR5
EAAT
Autoreceptors
mGluR2/3
mGluR4/8
Gln
GLNT
GRK
Gi/o
mGluR7
Gβγ
GIRK
K+
Gβγ
AC
KA
Ca2+
Neuron (Presynaptic terminal)
Feedback inhibition of glutamate release
cAMP
VGCC
Ca2+
Mitochondria
Gln
GLS
Glu
PKA
Glu
Synaptic vesicle
Exocytosis
vGLUT
Glu
Synaptic vesicle cycle
Glu
Endocytosis
Action potential
EAAT
Glu
Synaptic cleft
Glu
Ca2+
EAAT
VGCC
Neuron (Postsynaptic cell)
Na+
Ca2+
KA
Long-term potentiation
Long-term depression
Na+
AMPA
Neuronal excitability Synaptic plasticity
Endoplasmic reticulum
Ca2+
Na+
NMDAR
PP2B
PSD95
GKAP
SHANK
Calcium signaling pathway
Ca2+
TRPC1
IP3R
Homer
Homer
Homer
Homer
Ca2+
IP3
PLA2
Gq
PLC
DAG
PKC
PLD
Neuronal excitability Synaptic plasticity
ERK1/2
mGluR1/5
Gs
AC
cAMP
PKA
mGluR2/3
Gi/Go
mGluR4/6
mGluR7/8
04724 8/29/19
(c) Kanehisa Laboratories

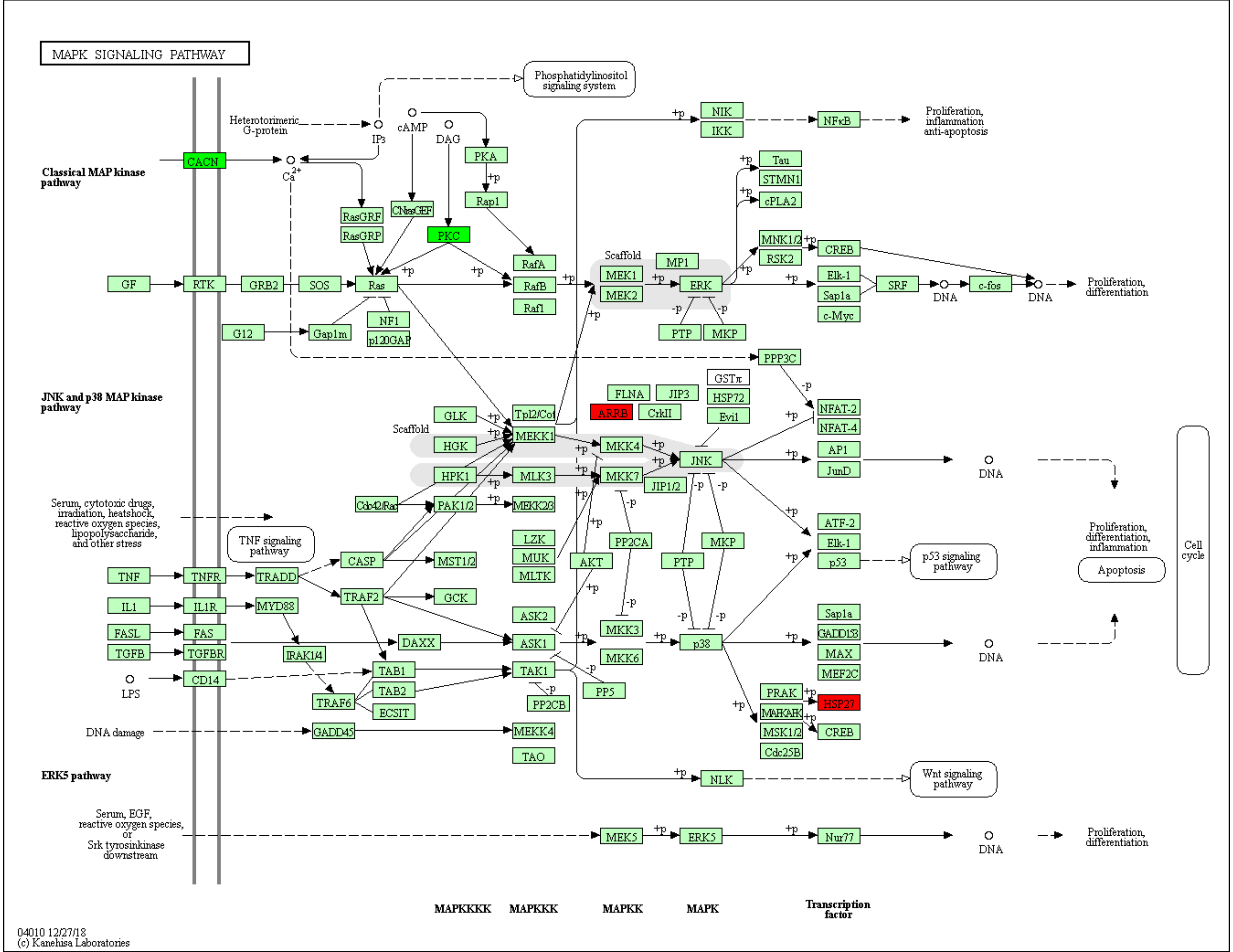
MAPK SIGNALING PATHWAY
Phosphatidylinositol signaling system
Heterotorimeric G-protein
IP3
cAMP
DAG
Classical MAP kinase pathway
CACN
Ca2+
PKA
Rap1
RasGRF
RasGRP
CNrasGEF
PKC
Scaffold
NIK
IKK
NFκB
Proliferation, inflammation anti-apoptosis
Tau
STMN1
cPLA2
MNK1/2
RSK2
CREB
GF
RTK
GRB2
SOS
Ras
RafA
RafB
Raf1
MEK1
MEK2
MP1
ERK
Elk-1
Sap1a
c-Myc
SRF
DNA
c-fos
DNA
Proliferation, differentiation
G12
Gap1m
NF1
p120GAP
PTP
MKP
PPP3C
JNK and p38 MAP kinase pathway
GSTπ
HSP72
Evi1
FLNA
JIP3
ARRB
CrkII
NFAT-2
NFAT-4
GLK
Tpl2/Cot
Scaffold
HGK
MEKK1
MKK4
JNK
AP1
JunD
DNA
HPK1
MLK3
MKK7
JIP1/2
Cdc42/Rac
PAK1/2
MEKK2/3
Serum, cytotoxic drugs, irradiation, heatshock, reactive oxygen species, lipopolysaccharide, and other stress
TNF signaling pathway
LZK
MUK
MLTK
PP2CA
MKP
AKT
PTP
ATF-2
Elk-1
p53
p53 signaling pathway
Proliferation, differentiation, inflammation
Apoptosis
Cell cycle
TNF
TNFR
TRADD
CASP
MST1/2
IL1
IL1R
MYD88
TRAF2
GCK
ASK2
FASL
FAS
DAXX
ASK1
MKK3
p38
Sap1a
GADD153
MAX
MEF2C
DNA
TGFB
TGFBR
IRAK1/4
MKK6
LPS
CD14
TAB1
TAK1
TAB2
PP2CB
PP5
TRAF6
ECSIT
PRAK
HSP27
MAPKAPK
MSK1/2
CREB
Cdc25B
DNA damage
GADD45
MEKK4
TAO
ERK5 pathway
NLK
Wnt signaling pathway
Serum, EGF, reactive oxygen species, or Srk tyrosinkinase downstream
MEK5
ERK5
Nur77
DNA
Proliferation, differentiation
+p
-p
MAPKKKK
MAPKKK
MAPKK
MAPK
Transcription factor
04010 12/27/18
(c) Kanehisa Laboratories
