# Supplementary material for: The Impacts of Surgery and Intracerebral Electrodes in C57BL/6J Mouse Kainate Model of Epileptogenesis: Seizure Threshold, Proteomics, and Cytokine Profiles
Source: Front Neurol. 2021 Jul 12;12:625017. doi: 10.3389/fneur.2021.625017 (PMC8312573; doi:10.3389/fneur.2021.625017)
Supplement: Supplementary Table 1 — List of all proteins identified by comparing all four groups using one-way ANOVA. [file Table_1.pdf]

Uniprot ID	Protein names	Gene names	KEGG-ID (mmu)	f.value	p.value	neg LOG10(p)	FDR	abundance	abundance	abundance	abundance
group								Vehicle-NoSurgery	Vehicle-Surgery	KA-NoSurgery	KA-Surgery
Q8VHL1	Histone-lysine N-methyltransferase SETD7 (EC 2.1.1.-) (Histone H3-K4 methyltransferase SETD7) (H3-K4-HMTase SETD7) (SET domain-containing protein 7) (SET7/9)	Setd7 Kiaa1717 Set7 Set9	73251	66.863	9.25E-08	7.0337	0.00014131	21.2323325	21.9110725	21.157675	21.9931025
Q9D0M5	Dynein light chain 2, cytoplasmic (8 kDa dynein light chain b) (DLC8) (DLC8b) (Dynein light chain LC8-type 2)	Dynll2 Dlc2	68097	58.2	0.000000202	6.6954	0.00015395	22.2105575	21.12811	22.1828225	21.00142
Q6A026	Sister chromatid cohesion protein PDS5 homolog A	Pds5a Kiaa0648		36.452	0.00000262	5.5812	0.001335	19.8724025	20.8859675	19.80909	20.84635
P03995	Glial fibrillary acidic protein (GFAP)	Gfap	14580	31.792	0.00000543	5.265	0.0013827	25.552435	27.0254125	26.8747475	27.968345
Q9QZF2	Glypican-1 [Cleaved into: Secreted glypican-1]	Gpc1	14733	33.445	0.00000415	5.3816	0.0013827	19.795055	19.762235	20.0398	20.2137725
Q8CIG9	F-box/LRR-repeat protein 8 (F-box and leucine-rich repeat protein 8) (F-box protein FBL8)	Fbxl8 Fbl8	50788	32.684	0.00000469	5.3286	0.0013827	19.1897075	20.3505375	18.93956	20.2725625
Q6PB66	Leucine-rich PPR motif-containing protein, mitochondrial (130 kDa leucine-rich protein) (LRP 130) (mLRP130)	Lrpprc Lrp130	72416	29.347	0.00000827	5.0823	0.0018049	21.1210575	21.556105	21.0233375	21.5066525
Q3URK3	Methylcytosine dioxygenase TET1 (EC 1.14.11.n2) (CXXC-type zinc finger protein 6) (Ten-eleven translocation 1 gene protein homolog)	Tet1 Cxxc6 Kiaa1676		27.562	0.0000115	4.9404	0.0018108	19.20219	20.01175	18.9776675	19.870405
P55012	Solute carrier family 12 member 2 (Basolateral Na-K-Cl symporter) (Bumetanide-sensitive sodium-(potassium)-chloride cotransporter 2)	Slc12a2 Nkcc1	20496	27.452	0.0000117	4.9313	0.0018108	21.57142	22.651055	21.59296	22.8383175
Q8C0E2	Vacuolar protein sorting-associated protein 26B (Vesicle protein sorting 26B)	Vps26b	69091	27.386	0.0000119	4.926	0.0018108	21.7700075	21.4647775	21.7023675	21.46987
P14106	Complement C1q subcomponent subunit B	C1qb	12260	25.31	0.0000178	4.7495	0.0019105	19.04926	20.753115	19.97015	21.1305225
P11798	Calcium/calmodulin-dependent protein kinase type II subunit alpha (CaM kinase II subunit alpha) (CaMK-II subunit alpha) (EC 2.7.11.17)	Camk2a	12322	24.743	0.00002	4.6992	0.0019105	28.23778	28.094555	28.0367125	27.6818325
P35505	Fumarylacetoacetase (FAA) (EC 3.7.1.2) (Beta-diketonase) (Fumarylacetoacetate hydrolase)	Fah	14085	26.129	0.0000151	4.8205	0.0019105	19.29231	19.8681325	19.0682925	19.8580125
P14602	Heat shock protein beta-1 (HspB1) (Growth-related 25 kDa protein) (Heat shock 25 kDa protein) (HSP 25) (Heat shock 27 kDa protein) (HSP 27) (p25)	Hspb1 Hsp25 Hsp27	15507	26.34	0.0000145	4.8385	0.0019105	18.345945	18.0248625	20.100525	21.6088075
P32037	Solute carrier family 2, facilitated glucose transporter member 3 (Glucose transporter type 3, brain) (GLUT-3)	Slc2a3 Glut3	20527	24.736	0.00002	4.6986	0.0019105	22.525515	22.3908675	22.637365	22.245415
Q5SSL4	Active breakpoint cluster region-related protein	Abr	109934	24.9	0.0000194	4.7132	0.0019105	21.39741	22.0329375	21.478645	22.0219375
P16045	Galectin-1 (Gal-1) (14 kDa lectin) (Beta-galactoside-binding lectin L-14-I) (Galaptin) (Lactose-binding lectin 1) (Lectin galactoside-binding soluble 1) (S-Lac lectin 1)	Lgals1 Gbp	16852	23.294	0.0000272	4.566	0.0024401	18.5541825	19.2993675	19.298105	20.2634575
Q9WVA3	Mitotic checkpoint protein BUB3 (WD repeat type I transmembrane protein A72.5)	Bub3	12237	22.904	0.0000296	4.5289	0.0025101	19.262225	19.5850125	19.2076475	19.39816
Q62283	Tetraspanin-7 (Tspan-7) (Cell surface glycoprotein A15) (PE31) (TALLA homolog) (Transmembrane 4 superfamily member 2) (CD antigen CD231)	Tspan7 Mxs1 Tm4sf2	21912	21.247	0.0000431	4.3654	0.0034651	19.890325	19.44267	19.6376425	19.2220725
P20152	Vimentin	Vim	22352	20.7	0.0000491	4.3091	0.0036078	23.70164	25.16986	24.871025	26.487395
P62301	40S ribosomal protein S13	Rps13	68052	20.655	0.0000496	4.3044	0.0036078	21.7074225	21.8528675	21.85315	21.976885

Q9CQJ6	Density-regulated protein (DRP)	Denr	68184	19.443	0.0000669	4.1749	0.0046404	19.6706675	20.1360725	19.6389375	20.130675
Q8CBW3	Abl interactor 1 (Abelson interactor 1) (Abi-1) (Ablphilin-1) (Eps8 SH3 domain-binding protein) (Eps8-binding protein) (Spectrin SH3 domain-binding protein 1) (e3B1)	Abi1 Ssh3bp1	11308	18.428	0.0000869	4.0612	0.0055266	21.2610075	20.738385	21.1604775	20.6898675
P35279	Ras-related protein Rab-6A (Rab-6)	Rab6a Rab6 MNCb-1660	19346	18.567	0.0000837	4.077	0.0055266	21.631565	21.371815	21.51557	21.370305
P02468	Laminin subunit gamma-1 (Laminin B2 chain) (Laminin-1 subunit gamma) (Laminin-10 subunit gamma) (Laminin-11 subunit gamma) (Laminin-2 subunit gamma) (Laminin-3 subunit gamma) (Laminin-4 subunit gamma) (Laminin-6 subunit gamma) (Laminin-7 subunit gamma) (Laminin-8 subunit gamma) (Laminin-9 subunit gamma) (S-laminin subunit gamma) (S-LAM gamma)	Lamc1 Lamb-2 Lamc-1		18.078	0.0000953	4.0209	0.0057608	18.7592775	19.305835	18.700965	19.5635775
Q99104	Unconventional myosin-Va (Dilute myosin heavy chain, non-muscle)	Myo5a Dilute	17918	17.692	0.00010578	3.9756	0.0057608	23.74006	23.75106	23.8725825	23.9059975
P62071	Ras-related protein R-Ras2	Rras2	66922	17.853	0.00010126	3.9946	0.0057608	20.505145	20.053165	20.351005	19.987185
P31648	Sodium- and chloride-dependent GABA transporter 1 (GAT-1) (Solute carrier family 6 member 1)	Slc6a1 Gabt1 Gat-1 Gat1	232333	17.805	0.0001026	3.9889	0.0057608	23.4446175	23.4137725	23.0698275	22.9273075
Q0VGU4	Neurosecretory protein VGF [Cleaved into: Neuroendocrine regulatory peptide-1 (NERP-1); Neuroendocrine regulatory peptide-2 (NERP-2); VGF-derived peptide TLQP-21; VGF-derived peptide TLQP-62]	Vgf	381677	17.569	0.00010941	3.961	0.0057608	20.149055	19.8244625	20.7232	21.1231525
Q9D8Y0	EF-hand domain-containing protein D2 (Swiprosin-1)	Efhd2 Sws1		17.443	0.00011325	3.946	0.0057644	23.1636375	23.2614225	23.4564875	23.668985
Q02105	Complement C1q subcomponent subunit C	C1qc C1qg	12262	16.908	0.00013145	3.8812	0.0063333	18.6042125	20.5832175	19.4382575	20.6050425
P39053	Dynamin-1 (EC 3.6.5.5)	Dnm1 Dnm Kiaa4093	13429	16.874	0.00013272	3.8771	0.0063333	27.24975	27.13156	27.2293275	26.9419975
Q9QYB8	Beta-adducin (Add97) (Erythrocyte adducin subunit beta)	Add2	11519	15.586	0.00019323	3.7139	0.0086777	23.798465	23.675545	23.7758925	23.5800425
P10605	Cathepsin B (EC 3.4.22.1) (Cathepsin B1) [Cleaved into: Cathepsin B light chain; Cathepsin B heavy chain]	Ctsb	13030	15.512	0.00019758	3.7042	0.0086777	21.2429425	21.535165	21.29265	21.835295
Q62188	Dihydropyrimidinase-related protein 3 (DRP-3) (Unc-33-like phosphoprotein 1) (ULIP-1)	Dpysl3 Drp3 Ulip	22240	15.49	0.0001989	3.7014	0.0086777	24.70239	24.31222	24.7052575	24.1349625
P21460	Cystatin-C (Cystatin-3)	Cst3	13010	15.395	0.00020476	3.6888	0.0086852	21.29772	21.5658275	21.740415	22.1588475
P61161	Actin-related protein 2 (Actin-like protein 2)	Actr2 Arp2	66713	15.215	0.00021628	3.665	0.008926	24.06893	23.8876075	24.0086775	23.72985
P98086	Complement C1q subcomponent subunit A	C1qa	12259	15.099	0.00022417	3.6494	0.0090082	17.523675	19.70244	18.785325	19.7552325
Q9Z2H5	Band 4.1-like protein 1 (Neuronal protein 4.1) (4.1N)	Epb41l1 Epb4 Epb4.1l1 Kiaa0338	13821	14.7	0.00025385	3.5954	0.0096907	22.8270275	22.77557	22.8274725	22.5536575
P15105	Glutamine synthetase (GS) (EC 6.3.1.2) (Glutamate--ammonia ligase) (Palmitoyltransferase GLUL) (EC 2.3.1.225)	Glul Glns	14645	14.75	0.00024989	3.6022	0.0096907	26.8151025	26.797415	26.5162525	26.618135
Q8K0S0	Phytanoyl-CoA hydroxylase-interacting protein (Phytanoyl-CoA hydroxylase-associated protein 1) (PAHX-AP1) (PAHXAP1)	Phyhip	105653	14.378	0.00028117	3.551	0.010223	22.730525	22.6948975	22.624895	22.378555
P13020	Gelsolin (Actin-depolymerizing factor) (ADF) (Brevin)	Gsn Gsb	227753	14.443	0.00027534	3.5601	0.010223	20.636415	20.872325	20.3412725	21.2156125
P80315	T-complex protein 1 subunit delta (TCP-1-delta) (A45) (CCT-delta)	Cct4 Cctd	12464	13.904	0.00032783	3.4844	0.011642	23.604635	23.6332625	23.5951	23.8069725
P48774	Glutathione S-transferase Mu 5 (EC 2.5.1.18) (Fibrous sheath component 2) (Fsc2) (GST class-mu 5)	Gstm5 Fsc2 Gstm3	14866	13.338	0.00039602	3.4023	0.011937	23.1223575	22.9011175	22.875265	22.7292525

Q61699	Heat shock protein 105 kDa (42 degrees C-HSP) (Heat shock 110 kDa protein) (Heat shock-related 100 kDa protein E7I) (HSP-E7I)	Hsph1 Hsp105 Hsp110 Kiaa0201	15505	13.611	0.00036127	3.4422	0.011937	24.1238425	23.9117625	24.4177	24.3203725
Q60864	Stress-induced-phosphoprotein 1 (STI1) (mSTI1) (Hsc70/Hsp90-organizing protein) (Hop)	Stip1	20867	13.549	0.00036879	3.4332	0.011937	23.9713425	23.853425	24.05359	23.91518
Q9Z1Z0	General vesicular transport factor p115 (Protein USO1 homolog) (Transcytosis-associated protein) (TAP) (Vesicle-docking protein)	Uso1 Vdp	56041	13.382	0.0003901	3.4088	0.011937	20.1995325	20.56909	19.904485	20.6245675
P62823	Ras-related protein Rab-3C	Rab3c	67295	13.318	0.00039868	3.3994	0.011937	23.00598	22.846675	23.0221	22.6693425
Q9DAW9	Calponin-3 (Calponin, acidic isoform)	Cnn3	71994	13.343	0.00039535	3.403	0.011937	19.146545	19.96104	20.157205	21.2932175
Q9DB73	NADH-cytochrome b5 reductase 1 (b5R.1) (EC 1.6.2.2) (NAD(P)H:quinone oxidoreductase type 3 polypeptide A2)	Cyb5r1 Nqo3a2	72017	13.439	0.00038275	3.4171	0.011937	19.3392025	19.8236025	19.31185	20.037075
B1AWN6	Sodium channel protein type 2 subunit alpha (Voltage-gated sodium channel subunit alpha Nav1.2)	Scn2a Scn2a1	110876	13.719	0.00034849	3.4578	0.011937	20.40293	19.85405	20.378525	19.717235
Q9WVA4	Transgelin-2 (SM22-beta)	Tagln2 Kiaa0120	21346	13.121	0.00042637	3.3702	0.012521	18.514165	18.7312275	19.2615	19.81564
Q8BIZ1	Ankyrin repeat and sterile alpha motif domain-containing protein 1B (Amyloid-beta protein intracellular domain-associated protein 1) (AIDA-1) (E2A-PBX1-associated protein) (EB-1)	Anks1b	77531	13.006	0.00044369	3.3529	0.012783	21.7332075	21.5619925	21.538305	21.1258475
Q9WUA3	ATP-dependent 6-phosphofructokinase, platelet type (ATP-PFK) (PFK-P) (EC 2.7.1.11) (6-phosphofructokinase type C) (Phosphofructo-1-kinase isozyme C) (PFK-C) (Phosphohexokinase)	Pfkp Pfkc	56421	12.831	0.00047143	3.3266	0.013089	24.222935	24.2833675	24.438795	24.4339925
Q6NZJ6	Eukaryotic translation initiation factor 4 gamma 1 (eIF-4-gamma 1) (eIF-4G 1) (eIF-4G1)	Eif4g1	208643	12.869	0.00046531	3.3323	0.013089	19.5309775	19.9471425	19.55449	20.01697
Q99L04	Dehydrogenase/reductase SDR family member 1 (EC 1.1.-.-)	Dhrs1 D14ertd484e	52585	12.764	0.00048254	3.3165	0.013158	20.2825625	21.2544475	20.767305	22.02584
O88741	Ganglioside-induced differentiation-associated protein 1 (GDAP1)	Gdap1	14545	12.493	0.00053115	3.2748	0.013803	20.902805	19.9807425	20.360985	19.87696
Q01097	Glutamate receptor ionotropic, NMDA 2B (GluN2B) (Glutamate [NMDA] receptor subunit epsilon-2) (N-methyl D-aspartate receptor subtype 2B) (NMDAR2B) (NR2B)	Grin2b	14812	12.481	0.00053332	3.273	0.013803	21.3902225	21.11142	21.1335775	20.6426975
Q91VI7	Ribonuclease inhibitor (Ribonuclease/angiogenin inhibitor 1)	Rnh1 Rnh	107702	12.485	0.00053262	3.2736	0.013803	20.95285	21.066705	21.24231	21.5124425
Q80VP1	Epsin-1 (EPS-15-interacting protein 1) (Intersectin-EH-binding protein 1) (Ibp1)	Epn1	13854	12.313	0.00056631	3.2469	0.013855	22.0676675	21.988785	22.1249275	21.8529225
Q8BGT8	Phytanoyl-CoA hydroxylase-interacting protein-like	Phyhipl	70911	12.244	0.00058069	3.2361	0.013855	22.55278	22.944585	22.5245525	23.03507
Q80XI4	Phosphatidylinositol 5-phosphate 4-kinase type-2 beta (EC 2.7.1.149) (1-phosphatidylinositol 5-phosphate 4-kinase 2-beta) (Diphosphoinositide kinase 2-beta) (Phosphatidylinositol 5-phosphate 4-kinase type II beta) (PI(5)P 4-kinase type II beta) (PIP4KII-beta) (PtdIns(5)P-4-kinase isoform 2-beta)	Pip4k2b Pip5k2b	108083	12.303	0.00056848	3.2453	0.013855	21.2027225	21.0485575	21.1886075	21.0013375
Q80U28	MAP kinase-activating death domain protein (Rab3 GDP/GTP exchange factor)	Madd Kiaa0358	228355	12.351	0.00055872	3.2528	0.013855	20.45633	20.187835	20.483835	19.9161975
Q7M6Y3	Phosphatidylinositol-binding clathrin assembly protein (Clathrin assembly lymphoid myeloid leukemia) (CALM)	Picalm Calm Fit1	233489	12.259	0.00057756	3.2384	0.013855	19.7694925	19.9714375	20.0624075	19.91016
P63318	Protein kinase C gamma type (PKC-gamma) (EC 2.7.11.13)	Prkcg Pkcc Pkcg Prkcc	18752	12.19	0.00059198	3.2277	0.013907	25.2724275	25.175815	25.0801775	24.8800575
Q03517	Secretogranin-2 (Chromogranin-C) (Secretogranin II) (SgII) [Cleaved into: Secretoneurin (SN); Manserin]	Scg2 Chgc Scg-2	20254	12.104	0.00061077	3.2141	0.01392	19.9008975	19.9947025	20.671545	20.654945

P46096	Synaptotagmin-1 (Synaptotagmin I) (SytI) (p65)	Syt1	20979	12.14	0.00060302	3.2197	0.01392	26.72158	26.6448325	26.7172075	26.4754325
Q8R001	Microtubule-associated protein RP/EB family member 2 (APC-binding protein EB2) (End-binding protein 2) (EB2)	Mapre2	212307	12.039	0.00062554	3.2037	0.014047	20.5243875	21.1106125	20.5616275	21.0607775
Q9D1A2	Cytosolic non-specific dipeptidase (EC 3.4.13.18) (CNDP dipeptidase 2) (Glutamate carboxypeptidase-like protein 1)	Cndp2 Cn2	66054	11.925	0.00065233	3.1855	0.014436	22.1219475	22.14448	22.0955525	22.34039
Q9JM76	Actin-related protein 2/3 complex subunit 3 (Arp2/3 complex 21 kDa subunit) (p21-ARC)	Arpc3	56378	11.851	0.00067038	3.1737	0.014624	23.274375	22.924555	23.17411	22.81725
P26041	Moesin (Membrane-organizing extension spike protein)	Msn	17698	11.735	0.0006998	3.155	0.015051	22.0154375	22.43039	22.4288575	23.1862025
P47757	F-actin-capping protein subunit beta (CapZ beta)	Capzb Cappb1	12345	11.65	0.00072224	3.1413	0.015318	23.863535	23.7331175	23.800495	23.688735
P0DP26	Calmodulin-1	Calm1 Calm Cam Cam1	1.23131E+14	11.538	0.00075341	3.123	0.01576	27.704655	27.704655	27.7334	27.501775
Q6P2B1	Transportin-3	Tnpo3	320938	11.495	0.00076569	3.1159	0.0158	18.0729525	18.9378925	18.5120925	18.9698475
Q921I1	Serotransferrin (Transferrin) (Beta-1 metal-binding globulin) (Siderophilin)	Tf Trf	22041	11.433	0.00078405	3.1057	0.015963	22.7249675	24.592435	22.4667425	24.1860475
P46460	Vesicle-fusing ATPase (EC 3.6.4.6) (N-ethylmaleimide-sensitive fusion protein) (NEM-sensitive fusion protein) (Suppressor of K(+) transport growth defect 2) (Protein SKD2) (Vesicular-fusion protein NSF)	Nsf Skd2	18195	11.325	0.00081674	3.0879	0.01641	27.364545	27.1957075	27.2949125	27.11948
P48036	Annexin A5 (Anchorin CII) (Annexin V) (Annexin-5) (Calphobindin I) (CBP-I) (Endonexin II) (Lipocortin V) (Placental anticoagulant protein 4) (PP4) (Placental anticoagulant protein I) (PAP-I) (Thromboplastin inhibitor) (Vascular anticoagulant-alpha) (VAC-alpha)	Anxa5 Anx5	11747	11.29	0.00082793	3.082	0.016414	22.9750325	23.2200475	23.06671	23.83366
Q8BK64	Activator of 90 kDa heat shock protein ATPase homolog 1 (AHA1)	Ahsa1	217737	11.257	0.00083846	3.0765	0.016414	22.24132	22.1257375	22.355265	22.056115
O70318	Band 4.1-like protein 2 (Generally expressed protein 4.1) (4.1G)	Epb41l2 Epb4.1l2	13822	11.052	0.00090743	3.0422	0.01754	21.4002975	21.737905	21.53289	21.90647
O08547	Vesicle-trafficking protein SEC22b (ER-Golgi SNARE of 24 kDa) (ERS-24) (ERS24) (SEC22 vesicle-trafficking protein homolog B) (SEC22 vesicle-trafficking protein-like 1) (mSec22b)	Sec22b Sec22l1	20333	10.923	0.00095447	3.0202	0.018218	21.486875	21.521715	21.374145	21.72766
P07724	Serum albumin	Alb Alb-1 Alb1	11657	10.705	0.00104	2.983	0.019235	26.73342	27.7777125	26.13416	27.9946525
P00405	Cytochrome c oxidase subunit 2 (EC 1.9.3.1) (Cytochrome c oxidase polypeptide II)	Mtco2 COII COX2 mt-Co2	17709	10.692	0.0010455	2.9807	0.019235	25.4208075	25.2559175	25.6176	25.27178
Q9QXS1	Plectin (PCN) (PLTN) (Plectin-1) (Plectin-6)	Plec Plec1	18810	10.689	0.0010467	2.9802	0.019235	23.93367	24.139575	24.052955	24.276045
Q9WTL7	Acyl-protein thioesterase 2 (APT-2) (EC 3.1.2.-) (Lysophospholipase 2) (Lysophospholipase II) (LPL-II) (LysoPLA II) (mLyso II)	Lypla2	26394	10.638	0.0010681	2.9714	0.019235	19.45755	18.96704	19.533185	19.01797
Q9DBY0	Forkhead box protein P4 (Fork head-related protein-like A) (mFKHLA)	Foxp4	74123	10.603	0.0010833	2.9653	0.019235	18.3423475	19.32305	18.3148825	19.24893
Q8BUV3	Gephyrin [Includes: Molybdopterin adenylyltransferase (MPT adenylyltransferase) (EC 2.7.7.75) (Domain G); Molybdopterin molybdenumtransferase (MPT Mo-transferase) (EC 2.10.1.1) (Domain E)]	Gphn	268566	10.621	0.0010755	2.9684	0.019235	22.04537	21.8644875	21.9781025	21.80613
Q9CPQ3	Mitochondrial import receptor subunit TOM22 homolog (Translocase of outer membrane 22 kDa subunit homolog)	Tomm22 Tom22	223696	10.467	0.0011444	2.9414	0.020087	20.7585925	20.5205125	20.8351	20.478105
P58252	Elongation factor 2 (EF-2)	Eef2	13629	10.417	0.0011675	2.9327	0.020259	24.614985	24.6250425	24.64119	24.9027875

P17879	Heat shock 70 kDa protein 1B (Heat shock 70 kDa protein 1) (HSP70.1)	Hspa1b Hcp70.1 Hsp70-1 Hsp70a1 Hspa1	15511	10.285	0.0012322	2.9093	0.021141	20.5826075	20.50446	21.03027	21.0309875
P63040	Complexin-1 (921-S) (Complexin I) (CPX I) (Synaphin-2)	Cplx1	12889	10.233	0.0012584	2.9002	0.02135	22.1957175	21.8365125	22.04076	21.69162
P14206	40S ribosomal protein SA (37 kDa laminin receptor precursor) (37LRP) (37 kDa oncofetal antigen) (37/67 kDa laminin receptor) (LRP/LR) (67 kDa laminin receptor) (67LR) (Laminin receptor 1) (LamR) (Laminin-binding protein precursor p40) (LBP/p40) (OFA/iLRP)	Rpsa Lamr1 P40-8	16785	10.137	0.0013093	2.883	0.021713	22.5084625	22.6329875	22.499215	22.78697
P63054	Calmodulin regulator protein PCP4 (Brain-specific antigen PCP-4) (Brain-specific polypeptide PEP-19) (Purkinje cell protein 4)	Pcp4 Pep19	18546	10.113	0.0013224	2.8786	0.021713	22.3945725	22.0801025	22.2440075	21.8735825
P63328	Serine/threonine-protein phosphatase 2B catalytic subunit alpha isoform (EC 3.1.3.16) (CAM-PRP catalytic subunit) (Calmodulin-dependent calcineurin A subunit alpha isoform) (CNA alpha)	Ppp3ca Calna	19055	10.14	0.0013077	2.8835	0.021713	26.5175825	26.429785	26.3758725	26.1648525
Q9QVP9	Protein-tyrosine kinase 2-beta (EC 2.7.10.2) (Calcium-dependent tyrosine kinase) (CADTK) (Calcium-regulated non-receptor proline-rich tyrosine kinase) (Cell adhesion kinase beta) (CAK-beta) (CAKB) (Focal adhesion kinase 2) (FADK 2) (Proline-rich tyrosine kinase 2) (Related adhesion focal tyrosine kinase) (RAFTK)	Ptk2b Fak2 Pyk2 Raftk	19229	10.069	0.0013468	2.8707	0.021878	21.8614	21.6260225	21.4940425	21.1459475
Q8CGK3	Lon protease homolog, mitochondrial (EC 3.4.21.53) (Lon protease-like protein) (LONP) (Mitochondrial ATP-dependent protease Lon) (Serine protease 15)	Lonp1 Prss15	74142	9.9251	0.00143	2.8447	0.022985	21.553535	21.3629725	21.602595	21.280165
Q8JZS0	Protein lin-7 homolog A (Lin-7A) (mLin-7) (Mammalian lin-seven protein 1) (MALS-1) (Vertebrate lin-7 homolog 1) (Veli-1)	Lin7a Mals1 Veli1	108030	9.8574	0.0014713	2.8323	0.023402	20.0467975	19.53813	20.0015325	19.369805
O70161	Phosphatidylinositol 4-phosphate 5-kinase type-1 gamma (PIP5K1-gamma) (PtdIns(4)P-5-kinase 1 gamma) (EC 2.7.1.68) (Phosphatidylinositol 4-phosphate 5-kinase type I gamma) (PIP5KIgamma)	Pip5k1c Kiaa0589	18717	9.7885	0.0015146	2.8197	0.023765	22.314815	22.0694525	22.337615	22.03283
Q8CI94	Glycogen phosphorylase, brain form (EC 2.4.1.1)	Pygb	110078	9.7721	0.0015252	2.8167	0.023765	25.248885	25.406675	25.249005	25.4990475
P28660	Nck-associated protein 1 (NAP 1) (Brain protein H19) (MH19) (Membrane-associated protein HEM-2) (p125Nap1)	Nckap1 Hem2 Kiaa0587 Nap1	50884	9.64	0.0016134	2.7923	0.024885	23.2878625	23.14601	23.3989675	23.0737375
P61979	Heterogeneous nuclear ribonucleoprotein K (hnRNP K)	Hnrnpk Hnrpk	15387	9.5609	0.001669	2.7775	0.025486	24.36472	24.4799525	24.441845	24.5781
Q9WUR9	Adenylate kinase 4, mitochondrial (AK 4) (EC 2.7.4.10) (EC 2.7.4.6) (Adenylate kinase 3-like) (Adenylate kinase isoenzyme 4) (GTP:AMP phosphotransferase AK4)	Ak4 Ak-4 Ak3b Ak3l1	11639	9.5128	0.001704	2.7685	0.025762	21.1321	20.9578275	20.81185	20.5620275
P50396	Rab GDP dissociation inhibitor alpha (Rab GDI alpha) (Guanosine diphosphate dissociation inhibitor 1) (GDI-1)	Gdi1 Rabgdia	14567	9.4159	0.001777	2.7503	0.026603	25.911435	25.7975875	26.00431	25.72274
Q60598	Src substrate cortactin	Cttn Ems1	13043	9.3011	0.0018682	2.7286	0.027697	22.75785	22.612865	22.5884425	22.421425
P28652	Calcium/calmodulin-dependent protein kinase type II subunit beta (CaM kinase II subunit beta) (CaMK-II subunit beta) (EC 2.7.11.17)	Camk2b Camk2d	12323	9.2176	0.001938	2.7127	0.028455	25.6829925	25.540075	25.61686	25.3386925
P52760	2-iminobutanoate/2-iminopropanoate deaminase (EC 3.5.99.10) (Heat-responsive protein 12) (Reactive intermediate imine deaminase A homolog) (Translation inhibitor L-PSP ribonuclease)	Rida Hrp12	15473	9.0399	0.0020969	2.6784	0.030495	21.72352	22.2627025	21.5593175	22.3609075
Q8VHW2	Voltage-dependent calcium channel gamma-8 subunit (Neuronal voltage-gated calcium	Cacng8		8.9144	0.0022183	2.654	0.031655	21.9685025	21.6512325	21.7361525	21.188255

	channel gamma-8 subunit) (Transmembrane AMPAR regulatory protein gamma-8) (TARP gamma-8)										
P80318	T-complex protein 1 subunit gamma (TCP-1-gamma) (CCT-gamma) (Matricin) (mTRiC-P5)	Cct3 Cctg	12462	8.9096	0.0022231	2.653	0.031655	23.18799	23.1676025	23.3074725	23.3648975
Q9JKK7	Tropomodulin-2 (Neuronal tropomodulin) (N-Tmod)	Tmod2	50876	8.894	0.0022389	2.65	0.031655	23.1220475	23.1132075	23.2491475	23.01985
Q06890	Clusterin (Apolipoprotein J) (Apo-J) (Clustrin) (Sulfated glycoprotein 2) (SGP-2) [Cleaved into: Clusterin beta chain; Clusterin alpha chain]	Clu Apoj Msgp-2	12759	8.759	0.0023802	2.6234	0.033345	21.24293	21.963535	21.68343	22.690925
Q3UHL1	CaM kinase-like vesicle-associated protein	Camkv	235604	8.5973	0.0025634	2.5912	0.035584	25.0949925	25.08133	24.88231	24.7179125
P19096	Fatty acid synthase (EC 2.3.1.85) [Includes: [Acyl-carrier-protein] S-acetyltransferase (EC 2.3.1.38); [Acyl-carrier-protein] S-malonyltransferase (EC 2.3.1.39); 3-oxoacyl-[acyl-carrier-protein] synthase (EC 2.3.1.41); 3-oxoacyl-[acyl-carrier-protein] reductase (EC 1.1.1.100); 3-hydroxyacyl-[acyl-carrier-protein] dehydratase (EC 4.2.1.59); Enoyl-[acyl-carrier-protein] reductase (EC 1.3.1.39); Oleoyl-[acyl-carrier-protein] hydrolase (EC 3.1.2.14)]	Fasn	14104	8.5734	0.0025918	2.5864	0.035655	23.675375	23.7595675	23.8300275	23.98368
Q8VDP6	CDP-diacylglycerol--inositol 3-phosphatidyltransferase (EC 2.7.8.11) (Phosphatidylinositol synthase) (PI synthase) (PtdIns synthase)	Cdipt Pis1	52858	8.3286	0.0029054	2.5368	0.039261	20.093925	19.78151	20.0950425	19.769485
Q9R1Q8	Transgelin-3 (Neuronal protein NP25)	Tagln3 Np25	56370	8.3441	0.0028843	2.54	0.039261	24.1663525	23.95592	24.29161	24.1734
Q9D8N0	Elongation factor 1-gamma (EF-1-gamma) (eEF-1B gamma)	Eef1g	67160	8.2859	0.0029645	2.528	0.039364	23.353545	23.3873425	23.3423025	23.5657975
Q8R071	Inositol-trisphosphate 3-kinase A (EC 2.7.1.127) (Inositol 1,4,5-trisphosphate 3-kinase A) (IP3 3-kinase A) (IP3K A) (InsP 3-kinase A)	Itpka	228550	8.3009	0.0029436	2.5311	0.039364	22.34186	22.403655	22.1485075	21.9210275
P08414	Calcium/calmodulin-dependent protein kinase type IV (CaMK IV) (EC 2.7.11.17) (CaM kinase-GR)	Camk4		8.2479	0.0030183	2.5202	0.039732	20.11602	19.1794325	19.974685	19.666665
Q9CPY7	Cytosol aminopeptidase (EC 3.4.11.1) (Leucine aminopeptidase 3) (LAP-3) (Leucyl aminopeptidase) (Proline aminopeptidase) (EC 3.4.11.5) (Prolyl aminopeptidase)	Lap3 Lapep	66988	8.1703	0.0031317	2.5042	0.040873	21.8562875	22.2342975	21.99734	22.442995
Q9R1Q9	V-type proton ATPase subunit S1 (V-ATPase subunit S1) (Protein C7-1) (V-ATPase Ac45 subunit) (V-ATPase S1 accessory protein) (Vacuolar proton pump subunit S1)	Atp6ap1 Atp6ip1 Atp6s1	54411	8.1463	0.0031679	2.4992	0.040995	21.079565	20.79305	21.2067125	20.6912175
Q6ZWV3	60S ribosomal protein L10 (Protein QM homolog) (Ribosomal protein L10)	Rpl10 Qm	110954	8.0334	0.0033442	2.4757	0.042912	20.588475	20.3341025	20.7087425	20.32727
P40142	Transketolase (TK) (EC 2.2.1.1) (P68)	Tkt	21881	8.0147	0.0033745	2.4718	0.04294	24.74178	24.775385	24.70898	24.9143425
Q9DBP5	UMP-CMP kinase (EC 2.7.4.14) (Deoxycytidylate kinase) (CK) (dCMP kinase) (Nucleoside-diphosphate kinase) (EC 2.7.4.6) (Uridine monophosphate/cytidine monophosphate kinase) (UMP/CMP kinase) (UMP/CMPK)	Cmpk1 Cmk Cmpk Uck Umk Umpk	66588	7.9429	0.0034938	2.4567	0.044091	22.506215	22.3926025	22.611045	22.374645
P80314	T-complex protein 1 subunit beta (TCP-1-beta) (CCT-beta)	Cct2 Cctb	12461	7.8961	0.0035743	2.4468	0.044737	23.2403325	23.49765	23.41207	23.53325
P63216	Guanine nucleotide-binding protein G(I)/G(S)/G(O) subunit gamma-3	Gng3 Gngt3	14704	7.8535	0.0036494	2.4378	0.045306	22.689165	22.4210325	22.74996	22.3192625
P23818	Glutamate receptor 1 (GluR-1) (AMPA-selective glutamate receptor 1) (GluR-A) (GluR-K1) (Glutamate receptor ionotropic, AMPA 1) (GluA1)	Gria1 Glur1	14799	7.825	0.0037006	2.4317	0.045572	23.1339325	22.8269025	22.9084475	22.4863525

P27773	Protein disulfide-isomerase A3 (EC 5.3.4.1) (58 kDa glucose-regulated protein) (58 kDa microsomal protein) (p58) (Disulfide isomerase ER-60) (Endoplasmic reticulum resident protein 57) (ER protein 57) (ERp57) (Endoplasmic reticulum resident protein 60) (ER protein 60) (ERp60)	Pdia3 Erp Erp60 Grp58	14827	7.7902	0.0037644	2.4243	0.045986	24.25358	24.2352075	24.28669	24.54165
Q9JHU4	Cytoplasmic dynein 1 heavy chain 1 (Cytoplasmic dynein heavy chain 1) (Dynein heavy chain, cytosolic)	Dync1h1 Dhc1 Dnch1 Dnchc1 Dyhc	13424	7.7699	0.0038022	2.42	0.046079	25.83596	25.8827675	25.89578	25.9345675
Q9Z2W9	Glutamate receptor 3 (GluR-3) (AMPA-selective glutamate receptor 3) (GluR-C) (GluR-K3) (Glutamate receptor ionotropic, AMPA 3) (GluA3)	Gria3 Glur3 Kiaa4184	53623	7.7277	0.0038821	2.4109	0.046676	21.2032225	21.286915	21.322195	20.9337525
O70443	Guanine nucleotide-binding protein G(z) subunit alpha (G(x) alpha chain) (Gz-alpha)	Gnaz	14687	7.6931	0.0039491	2.4035	0.046746	22.64333	22.4577975	22.742785	22.3393175
Q6ZQ38	Cullin-associated NEDD8-dissociated protein 1 (Cullin-associated and neddylation-dissociated protein 1) (p120 CAND1)	Cand1 D10Ertd516e Kiaa0829	71902	7.695	0.0039453	2.4039	0.046746	23.7345075	23.8408025	23.96651	23.8912125
P97427	Dihydropyrimidinase-related protein 1 (DRP-1) (Collapsin response mediator protein 1) (CRMP-1) (Inactive dihydropyrimidinase) (Unc-33-like phosphoprotein 3) (ULIP-3)	Crmp1 Dpysl1 Ulip3	12933	7.6044	0.0041271	2.3844	0.048143	25.71165	25.6238575	25.7647225	25.4620375
P68404	Protein kinase C beta type (PKC-B) (PKC-beta) (EC 2.7.11.13)	Prkcb Pkcb Prkcb1	18751	7.6029	0.0041301	2.384	0.048143	23.6869375	23.43405	23.58409	23.37421
Q64521	Glycerol-3-phosphate dehydrogenase, mitochondrial (GPD-M) (GPDH-M) (EC 1.1.5.3) (Protein TISP38)	Gpd2 Gdm1	14571	7.5799	0.0041779	2.379	0.048331	24.8258375	24.764455	24.8052425	24.662745
O08709	Peroxiredoxin-6 (EC 1.11.1.15) (1-Cys peroxiredoxin) (1-Cys PRX) (Acidic calcium-independent phospholipase A2) (aiPLA2) (EC 3.1.1.4) (Antioxidant protein 2) (Non-selenium glutathione peroxidase) (NSGPx)	Prdx6 Aop2 Ltw4 Prdx5	11758	7.5051	0.0043375	2.3628	0.049062	25.42138	25.89032	25.6392175	26.22826
P63044	Vesicle-associated membrane protein 2 (VAMP-2) (Synaptobrevin-2)	Vamp2 Syb2	22318	7.5091	0.0043288	2.3636	0.049062	24.33924	24.103575	24.236665	23.9826625
Q8CHX7	Raftlin-2 (Raft-linking protein 2)	Rftn2	74013	7.5242	0.0042961	2.3669	0.049062	20.3478675	20.3866925	20.084725	20.219705
P50516	V-type proton ATPase catalytic subunit A (V-ATPase subunit A) (EC 7.1.2.2) (V-ATPase 69 kDa subunit) (Vacuolar proton pump subunit alpha)	Atp6v1a Atp6a1 Atp6a2 Atp6v1a1	11964	7.4889	0.0043731	2.3592	0.049101	26.6924075	26.62327	26.8269425	26.6163225
P17183	Gamma-enolase (EC 4.2.1.11) (2-phospho-D-glycerate hydro-lyase) (Enolase 2) (Neural enolase) (Neuron-specific enolase) (NSE)	Eno2 Eno-2	13807	7.4491	0.0044617	2.3505	0.049731	26.44807	26.47131	26.5758175	26.35803
P60469	Liprin-alpha-3 (Protein tyrosine phosphatase receptor type f polypeptide-interacting protein alpha-3) (PTPRF-interacting protein alpha-3)	Ppfia3	76787	7.3978	0.004579	2.3392	0.050668	20.748585	20.44135	20.65075	20.273025
P55066	Neurocan core protein (Chondroitin sulfate proteoglycan 3)	Ncan Cspg3	13004	7.3077	0.0047942	2.3193	0.05254	22.83813	23.038075	22.938665	23.2555925
Q8BLR2	Copine-4 (Copine IV)	Cpne4	74020	7.2984	0.004817	2.3172	0.05254	20.3785675	20.084765	20.0244	19.638225
O35874	Neutral amino acid transporter A (Alanine/serine/cysteine/threonine transporter 1) (ASCT-1) (SATT) (Solute carrier family 1 member 4)	Slc1a4 Asct1	55963	7.2213	0.0050116	2.3	0.054275	21.56002	21.9378675	22.000805	22.2471825
Q99P72	Reticulon-4 (Neurite outgrowth inhibitor) (Nogo protein)	Rtn4 Kiaa0886 Nogo	68585	7.196	0.0050772	2.2944	0.054597	24.0270175	24.07918	24.1879675	24.22461
Q8BTM8	Filamin-A (FLN-A) (Actin-binding protein 280) (ABP-280) (Alpha-filamin) (Endothelial actin-binding protein) (Filamin-1) (Non-muscle filamin)	Flna Fln Fln1	192176	7.182	0.0051142	2.2912	0.054611	18.7494675	19.1191175	18.9042275	19.97562
P14069	Protein S100-A6 (5B10) (Calcyclin) (Prolactin receptor-associated protein) (S100 calcium-binding protein A6)	S100a6 Cacy	20200	7.1452	0.0052123	2.283	0.054891	19.7351	19.5286375	19.591755	20.707745

Q80XN0	D-beta-hydroxybutyrate dehydrogenase, mitochondrial (EC 1.1.1.30) (3-hydroxybutyrate dehydrogenase) (BDH)	Bdh1 Bdh	71911	7.1505	0.0051981	2.2842	0.054891	23.6411	23.406245	23.6515925	23.3803175
P35803	Neuronal membrane glycoprotein M6-b (M6b)	Gpm6b M6b	14758	7.1155	0.0052935	2.2763	0.055364	23.8291	23.61138	23.639615	23.4751425
P84086	Complexin-2 (921-L) (Complexin II) (CPX II) (Synaphin-1)	Cplx2	12890	7.0659	0.005432	2.265	0.056426	24.4703425	24.4142875	24.5243475	24.17291
Q8K021	Secretory carrier-associated membrane protein 1 (Secretory carrier membrane protein 1)	Scamp1	107767	7.0282	0.00554	2.2565	0.05716	22.3761725	22.2963375	22.3869325	22.164275
P62270	40S ribosomal protein S18 (Ke-3) (Ke3)	Rps18	20084	6.9614	0.0057377	2.2413	0.058802	22.0260025	22.20016	22.13329	22.352765
Q9JME5	AP-3 complex subunit beta-2 (Adaptor protein complex AP-3 subunit beta-2) (Adaptor-related protein complex 3 subunit beta-2) (Beta-3B-adaptin) (Clathrin assembly protein complex 3 beta-2 large chain)	Ap3b2	11775	6.8803	0.0059889	2.2227	0.060238	21.83769	21.607915	21.676595	21.46443
Q62420	Endophilin-A1 (Endophilin-1) (SH3 domain protein 2A) (SH3 domain-containing GRB2-like protein 2) (SH3p4)	Sh3gl2 Een1 Sh3d2a	20404	6.8657	0.0060356	2.2193	0.060238	25.1224675	24.922645	25.118285	24.8753425
F6SEU4	Ras/Rap GTPase-activating protein SynGAP (Neuronal RasGAP) (Synaptic Ras GTPase-activating protein 1) (Synaptic Ras-GAP 1)	Syngap1	240057	6.8686	0.0060262	2.22	0.060238	23.9857225	23.9828475	23.9224925	23.5798975
Q9Z0H4	CUGBP Elav-like family member 2 (CELF-2) (Bruno-like protein 3) (CUG triplet repeat RNA-binding protein 2) (CUG-BP2) (CUG-BP- and ETR-3-like factor 2) (ELAV-type RNA-binding protein 3) (ETR-3) (mETR-3) (Neuroblastoma apoptosis-related RNA-binding protein) (mNapor) (RNA-binding protein BRUNOL-3)	Celf2 Cugbp2 Napor	14007	6.902	0.0059206	2.2276	0.060238	20.2252875	19.7627075	20.02439	19.8194875
Q8R0S2	IQ motif and SEC7 domain-containing protein 1	Iqsec1 Kiaa0763	232227	6.7536	0.0064075	2.1933	0.063534	22.2781825	21.9659175	22.1168925	21.9884325
Q9CQZ5	NADH dehydrogenase [ubiquinone] 1 alpha subcomplex subunit 6 (Complex I-B14) (CI-B14) (NADH-ubiquinone oxidoreductase B14 subunit)	Ndufa6	67130	6.7152	0.006541	2.1844	0.064439	22.2871225	22.1605375	22.106295	22.0772625
P23819	Glutamate receptor 2 (GluR-2) (AMPA-selective glutamate receptor 2) (GluR-B) (GluR-K2) (Glutamate receptor ionotropic, AMPA 2) (GluA2)	Gria2 Glur2		6.6704	0.0067006	2.1739	0.06446	23.869825	23.6963775	23.6413475	23.323225
Q9D0F9	Phosphoglucomutase-1 (PGM 1) (EC 5.4.2.2) (Glucose phosphomutase 1) (Phosphoglucomutase-2)	Pgm1 Pgm2	72157	6.7021	0.0065871	2.1813	0.06446	22.65725	22.7475275	22.5834425	22.638995
Q7TQI3	Ubiquitin thioesterase OTUB1 (EC 3.4.19.12) (Deubiquitinating enzyme OTUB1) (OTU domain-containing ubiquitin aldehyde-binding protein 1) (Otubain-1) (Ubiquitin-specific-processing protease OTUB1)	Otub1	107260	6.6586	0.0067437	2.1711	0.06446	23.4384	23.449595	23.3391825	23.394585
Q6NS52	Diacylglycerol kinase beta (DAG kinase beta) (EC 2.7.1.107) (Diglyceride kinase beta) (DGK-beta)	Dgkb Kiaa0718	217480	6.6678	0.00671	2.1733	0.06446	19.825355	19.8389725	19.45579	19.10711
Q7TPM6	Fibronectin type III and SPRY domain-containing protein 1	Fsd1	240121	6.6557	0.0067542	2.1704	0.06446	19.307345	18.9525175	19.53649	18.72285
Q61838	Pregnancy zone protein (Alpha-2-macroglobulin) (Alpha-2-M) [Cleaved into: Alpha-2-macroglobulin 165 kDa subunit; Alpha-2-macroglobulin 35 kDa subunit]	Pzp A2m		6.6336	0.0068352	2.1652	0.064671	20.9669125	23.0714225	20.3725775	22.1607625
Q9QUR7	Peptidyl-prolyl cis-trans isomerase NIMA-interacting 1 (EC 5.2.1.8) (Peptidyl-prolyl cis-trans isomerase Pin1) (PPIase Pin1)	Pin1	23988	6.6267	0.006861	2.1636	0.064671	20.744435	20.239785	20.560445	20.25966
P28740	Kinesin-like protein KIF2A (Kinesin-2)	Kif2a Kif2 Kns2	16563	6.6075	0.006933	2.1591	0.064949	21.293155	21.11292	21.1679675	20.88349
P70333	Heterogeneous nuclear ribonucleoprotein H2 (hnRNP H2) (Heterogeneous nuclear ribonucleoprotein H') (hnRNP H') [Cleaved into:	Hnrnph2 Hnrph2	56258	6.5816	0.0070312	2.153	0.065467	20.0786925	19.8530825	20.1713675	19.8623525

	Heterogeneous nuclear ribonucleoprotein H2, N-terminally processed]										
Q62418	Drebrin-like protein (Actin-binding protein 1) (SH3 domain-containing protein 7)	Dbnl Abp1 Sh3p7	13169	6.5472	0.0071644	2.1448	0.066303	22.2294325	22.122095	22.1696425	21.9346875
P61164	Alpha-centractin (Centractin) (ARP1) (Actin-RPV) (Centrosome-associated actin homolog)	Actr1a Ctrn1	54130	6.5255	0.0072497	2.1397	0.066689	22.83178	22.741945	22.8675225	22.91856
P62908	40S ribosomal protein S3 (EC 4.2.99.18)	Rps3	27050	6.4959	0.0073683	2.1326	0.067374	23.3860375	23.4449375	23.3741075	23.61716
P35438	Glutamate receptor ionotropic, NMDA 1 (GluN1) (Glutamate [NMDA] receptor subunit zeta-1) (N-methyl-D-aspartate receptor subunit NR1) (NMD-R1)	Grin1 Glurz1	14810	6.4191	0.0076863	2.1143	0.069863	22.373405	22.15976	22.088475	21.9530425
P68040	Receptor of activated protein C kinase 1 (12-3) (Guanine nucleotide-binding protein subunit beta-2-like 1) (Receptor for activated C kinase) (Receptor of activated protein kinase C 1) (p205) [Cleaved into: Receptor of activated protein C kinase 1, N-terminally processed (Guanine nucleotide-binding protein subunit beta-2-like 1, N-terminally processed)]	Rack1 Gnb2-rs1 Gnb2l1	14694	6.4009	0.007764	2.1099	0.070152	22.610215	22.748295	22.745795	22.916495
Q8CAY6	Acetyl-CoA acetyltransferase, cytosolic (EC 2.3.1.9) (Cytosolic acetoacetyl-CoA thiolase)	Acat2	110460	6.3702	0.007897	2.1025	0.070934	21.71279	21.533425	21.9052975	21.65077
P48453	Serine/threonine-protein phosphatase 2B catalytic subunit beta isoform (EC 3.1.3.16) (CAM-PRP catalytic subunit) (Calmodulin-dependent calcineurin A subunit beta isoform) (CNA beta)	Ppp3cb Calnb	19056	6.3543	0.0079669	2.0987	0.071143	22.255945	21.769415	22.0675525	21.6793425
Q921F2	TAR DNA-binding protein 43 (TDP-43)	Tardbp Tdp43	230908	6.3368	0.0080449	2.0945	0.071422	20.64877	20.7918975	20.9727075	20.9074675
Q9DBG6	Dolichyl-diphosphooligosaccharide--protein glycosyltransferase subunit 2 (Dolichyl-diphosphooligosaccharide--protein glycosyltransferase 63 kDa subunit) (Ribophorin II) (RPN-II) (Ribophorin-2)	Rpn2	20014	6.3252	0.0080971	2.0917	0.07147	20.28205	20.60156	20.62976	20.7954125
P20108	Thioredoxin-dependent peroxide reductase, mitochondrial (EC 1.11.1.15) (Antioxidant protein 1) (AOP-1) (PRX III) (Perioredoxin-3) (Protein MER5)	Prdx3 Aop1 Mer5	11757	6.3001	0.0082109	2.0856	0.072058	23.29468	23.4898075	23.3688175	23.48708
P0C7L0	WAS/WASL-interacting protein family member 3 (Corticosteroids and regional expression protein 16 homolog)	Wipf3 Cr16		6.1941	0.0087142	2.0598	0.075261	20.571555	20.32685	20.3079	20.2682525
P18242	Cathepsin D (EC 3.4.23.5)	Ctsd	13033	6.1827	0.0087704	2.057	0.075261	21.89864	22.1988725	22.0804325	22.7861275
Q9CZX8	40S ribosomal protein S19	Rps19	20085	6.1996	0.008687	2.0611	0.075261	21.5271925	21.6218575	21.5247175	21.6859
Q80TJ1	Calcium-dependent secretion activator 1 (Calcium-dependent activator protein for secretion 1) (CAPS-1)	Cadps Caps Caps1 Kiaa1121	27062	6.1821	0.008773	2.0568	0.075261	24.4017375	24.282395	24.3364575	24.1684175
E9PV24	Fibrinogen alpha chain [Cleaved into: Fibrinopeptide A; Fibrinogen alpha chain]	Fga	14161	6.1194	0.0090901	2.0414	0.077545	18.8942875	21.15421	17.6640825	20.102215
Q61316	Heat shock 70 kDa protein 4 (Heat shock 70-related protein APG-2)	Hspa4 Apg2 Hsp110		6.0965	0.0092088	2.0358	0.07769	24.9810225	24.8962625	25.0535675	24.97207
P14148	60S ribosomal protein L7	Rpl7	19989	6.1015	0.009183	2.037	0.07769	22.0609075	22.0871575	22.0935025	22.2815975
Q61081	Hsp90 co-chaperone Cdc37 (Hsp90 chaperone protein kinase-targeting subunit) (p50Cdc37) [Cleaved into: Hsp90 co-chaperone Cdc37, N-terminally processed]	Cdc37	12539	6.0172	0.0096353	2.0161	0.080841	20.4972325	20.44924	20.7173775	20.5132575
Q9QYJ0	DnaJ homolog subfamily A member 2 (mDj3)	Dnaja2	56445	5.9617	0.0099476	2.0023	0.083005	21.9455175	21.720155	21.924825	21.7284
Q91X72	Hemopexin	Hpx Hpxn	15458	5.9517	0.010005	1.9998	0.08303	20.30757	22.88745	20.4314275	21.5803425

Q91WG7	Diacylglycerol kinase gamma (DAG kinase gamma) (EC 2.7.1.107) (88 kDa diacylglycerol kinase) (Diglyceride kinase gamma) (DGK-gamma)	Dgkg Dagk3	110197	5.9418	0.010062	1.9973	0.083054	19.794405	19.6627525	19.6784625	19.0019625
Q8BKZ9	Pyruvate dehydrogenase protein X component, mitochondrial (Dihydrolipoamide dehydrogenase-binding protein of pyruvate dehydrogenase complex) (Lipoyl-containing pyruvate dehydrogenase complex component X)	Pdhx	27402	5.9323	0.010118	1.9949	0.083063	22.6232075	22.431385	22.4712975	22.307935
P63082	V-type proton ATPase 16 kDa proteolipid subunit (V-ATPase 16 kDa proteolipid subunit) (PL16) (Vacuolar proton pump 16 kDa proteolipid subunit)	Atp6v0c Atp6c Atp6l Atpl Mvp	11984	5.9014	0.0103	1.9872	0.084107	23.5905625	22.7642975	23.6384625	22.865885
P20029	Endoplasmic reticulum chaperone BiP (EC 3.6.4.10) (78 kDa glucose-regulated protein) (GRP-78) (Binding-immunoglobulin protein) (BiP) (Heat shock protein 70 family protein 5) (HSP70 family protein 5) (Heat shock protein family A member 5) (Immunoglobulin heavy chain-binding protein)	Hspa5 Grp78	14828	5.8667	0.010509	1.9784	0.08536	24.34168	24.291245	24.3653025	24.5272375
Q61411	GTPase HRas (H-Ras-1) (Transforming protein p21) (c-H-ras) (p21ras) [Cleaved into: GTPase HRas, N-terminally processed]	Hras Hras1	15461	5.8054	0.010891	1.9629	0.087996	21.8675125	21.8433175	21.9883775	21.772875
Q9D3A9	Protein tweety homolog 1 (mTTY1)	Ttyh1	57776	5.7809	0.011049	1.9567	0.088416	20.89435	20.642965	20.45929	20.27998
Q9CWF2	Tubulin beta-2B chain	Tubb2b	73710	5.7792	0.011059	1.9563	0.088416	22.99725	22.678395	22.850735	22.587855
Q60780	Growth arrest-specific protein 7 (GAS-7)	Gas7		5.7498	0.011252	1.9488	0.089488	20.0832925	19.976205	20.0311925	19.7189175
P54071	Isocitrate dehydrogenase [NADP], mitochondrial (IDH) (EC 1.1.1.42) (ICD-M) (IDP) (NADP(+)-specific ICDH) (Oxalosuccinate decarboxylase)	Idh2	269951	5.7389	0.011324	1.946	0.089597	22.0956975	22.56157	22.192485	22.57145
Q9CWZ7	Gamma-soluble NSF attachment protein (SNAP-gamma) (N-ethylmaleimide-sensitive factor attachment protein gamma)	Napg Snapg	108123	5.682	0.01171	1.9314	0.092175	23.897085	23.7640075	23.80743	23.71939
Q8K212	Phosphofurin acidic cluster sorting protein 1 (PACS-1)	Pacs1	107975	5.6549	0.0119	1.9245	0.093185	19.7191	19.545045	19.7881875	19.471575
P61021	Ras-related protein Rab-5B	Rab5b	19344	5.6225	0.012131	1.9161	0.094509	20.8419625	20.733285	20.7684175	20.5458575
Q8VDD5	Myosin-9 (Cellular myosin heavy chain, type A) (Myosin heavy chain 9) (Myosin heavy chain, non-muscle IIa) (Non-muscle myosin heavy chain A) (NMMHC-A) (Non-muscle myosin heavy chain IIa) (NMMHC II-a) (NMMHC-IIA)	Myh9	17886	5.5236	0.012869	1.8904	0.098544	22.4430925	22.5494875	22.6690275	22.836965
Q00915	Retinol-binding protein 1 (Cellular retinol-binding protein) (CRBP) (Cellular retinol-binding protein I) (CRBP-I) (mCRBPI)	Rbp1 Crbpi Rbp-1	19659	5.5157	0.012931	1.8884	0.098544	18.49154	18.7762925	19.02276	19.6864525
P0C0S6	Histone H2A.Z (H2A/z)	H2az1 H2afz H2az	51788	5.5022	0.013036	1.8849	0.098544	20.951595	21.2052175	21.023955	21.27349
Q8BWT1	3-ketoacyl-CoA thiolase, mitochondrial (EC 2.3.1.16) (Acetyl-CoA acetyltransferase) (EC 2.3.1.9) (Acetyl-CoA acyltransferase) (Acyl-CoA hydrolase, mitochondrial) (EC 3.1.2.-) (EC 3.1.2.1) (EC 3.1.2.2) (Beta-ketothiolase) (Mitochondrial 3-oxoacyl-CoA thiolase)	Acaa2	52538	5.5368	0.012767	1.8939	0.098544	21.528675	21.2647875	21.3597925	20.8706075
Q9D6M3	Mitochondrial glutamate carrier 1 (GC-1) (Glutamate/H(+) symporter 1) (Solute carrier family 25 member 22)	Slc25a22 Gc1	68267	5.5048	0.013016	1.8855	0.098544	24.8398525	24.746705	24.8474625	24.6917275
Q91VW3	SH3 domain-binding glutamic acid-rich-like protein 3	Sh3bgrl3	73723	5.5052	0.013012	1.8857	0.098544	22.376265	22.187665	22.4745675	22.2201075
Q60854	Serpin B6 (Placental thrombin inhibitor) (Proteinase inhibitor 6) (PI-6)	Serpinb6 Serpinb6a Spi3	20719	5.4796	0.013215	1.8789	0.099038	21.43811	21.19337	21.211965	21.2635575

P12970	60S ribosomal protein L7a (Surfeit locus protein 3)	Rpl7a Surf-3 Surf3	27176	5.4775	0.013231	1.8784	0.099038	22.0779325	22.126045	21.9669325	22.2910475
Q9CQI6	Coactosin-like protein	Cotl1 Clp	72042	5.4691	0.013299	1.8762	0.099058	22.4871725	22.6161125	22.5032375	22.803365
P07356	Annexin A2 (Annexin II) (Annexin-2) (Calpactin I heavy chain) (Calpactin-1 heavy chain) (Chromobindin-8) (Lipocortin II) (Placental anticoagulant protein IV) (PAP-IV) (Protein I) (p36)	Anxa2 Anx2 Cal1h	12306	5.4455	0.013489	1.87	0.099332	18.15158	18.4752975	18.91306	19.579185
P63011	Ras-related protein Rab-3A	Rab3a	19339	5.4416	0.013521	1.869	0.099332	26.398095	26.28494	26.3180675	26.1208975
P62259	14-3-3 protein epsilon (14-3-3E)	Ywhae	22627	5.4405	0.01353	1.8687	0.099332	28.12331	28.0288	28.155435	27.9791475
Q9EQF6	Dihydropyrimidinase-related protein 5 (DRP-5) (Collapsin response mediator protein 5) (CRMP-5)	Dpysl5 Crmp5	65254	5.4226	0.013678	1.864	0.099933	23.7360775	23.5063925	23.7570075	23.2860075
P24270	Catalase (EC 1.11.1.6)	Cat Cas-1 Cas1	12359	5.4077	0.013802	1.8601	0.10036	19.18145	19.04133	19.319695	19.5534675
O08532	Voltage-dependent calcium channel subunit alpha-2/delta-1 (Voltage-gated calcium channel subunit alpha-2/delta-1) [Cleaved into: Voltage-dependent calcium channel subunit alpha-2-1; Voltage-dependent calcium channel subunit delta-1]	Cacna2d1 Cacna2	12293	5.3883	0.013965	1.855	0.10106	22.4158375	22.3775675	22.530655	22.2252975
P31786	Acyl-CoA-binding protein (ACBP) (Diazepam-binding inhibitor) (DBI) (Endozepine) (EP)	Dbi	13167	5.3441	0.014347	1.8432	0.10301	23.5923675	23.790225	23.5175	23.9351425
P62855	40S ribosomal protein S26	Rps26	27370	5.3415	0.014369	1.8426	0.10301	20.69866	20.754135	20.79754	20.9714675
Q9CYT6	Adenylyl cyclase-associated protein 2 (CAP 2)	Cap2	67252	5.3299	0.014472	1.8395	0.10327	22.9266775	22.76914	22.92898	22.68561
Q6R0H7	Guanine nucleotide-binding protein G(s) subunit alpha isoforms XLas (Adenylate cyclase-stimulating G alpha protein) (Extra large alphas protein) (XLalphas)	Gnas Gnas1	14683	5.3116	0.014635	1.8346	0.10377	21.1394325	21.357435	21.2484325	21.30768
P67778	Prohibitin (B-cell receptor-associated protein 32) (BAP 32)	Phb	18673	5.3029	0.014713	1.8323	0.10377	23.76142	23.8959975	23.738985	23.866535
Q9QYX7	Protein piccolo (Aczonin) (Brain-derived HLMN protein) (Multidomain presynaptic cytomatrix protein)	Pclo Acz	26875	5.2991	0.014747	1.8313	0.10377	22.628785	22.49775	22.62817	22.2733025
P35700	Peroxiredoxin-1 (EC 1.11.1.15) (Macrophage 23 kDa stress protein) (Osteoblast-specific factor 3) (OSF-3) (Thioredoxin peroxidase 2) (Thioredoxin-dependent peroxide reductase 2)	Prdx1 Msp23 Paga Tdpx2	18477	5.2841	0.014884	1.8273	0.10425	24.572325	24.7003225	24.43803	24.89176
Q63912	Oligodendrocyte-myelin glycoprotein	Omg Omgp	18377	5.2394	0.0153	1.8153	0.10668	20.596335	20.6552775	20.647475	20.8117575
P22599	Alpha-1-antitrypsin 1-2 (AAT) (Alpha-1 protease inhibitor 2) (Alpha-1-antiproteinase) (Serine protease inhibitor 1-2) (Serine protease inhibitor A1b) (Serpin A1b)	Serpina1b Aat2 Dom2 Spi1-2	20701	5.1814	0.015859	1.7997	0.10911	19.4689625	21.1108375	19.6850275	20.7400225
Q00897	Alpha-1-antitrypsin 1-4 (Alpha-1 protease inhibitor 4) (Serine protease inhibitor 1-4) (Serine protease inhibitor A1d) (Serpin A1d)	Serpina1d Dom4 Spi1-4	20703	5.1684	0.015987	1.7962	0.10911	21.8422475	22.98512	21.588345	22.8601775
P56695	Wolframin	Wfs1	22393	5.1775	0.015897	1.7987	0.10911	20.032935	19.9452775	20.3576075	20.3938875
Q9CZM2	60S ribosomal protein L15	Rpl15	66480	5.1636	0.016035	1.7949	0.10911	21.12435	21.145965	21.17344	21.36444
Q91V92	ATP-citrate synthase (EC 2.3.3.8) (ATP-citrate (pro-S-)-lyase) (Citrate cleavage enzyme)	Acly	104112	5.1522	0.016149	1.7919	0.10911	22.6008775	22.5285525	22.79023	22.6163925
Q8R127	Saccharopine dehydrogenase-like oxidoreductase (EC 1.-.-.-)	Sccpdh	109232	5.175	0.015922	1.798	0.10911	19.585905	19.513705	19.937985	19.6114675
Q8BWF0	Succinate-semialdehyde dehydrogenase, mitochondrial (EC 1.2.1.24) (Aldehyde	Aldh5a1	214579	5.1586	0.016085	1.7936	0.10911	24.0662825	24.147575	24.0076425	24.1122625

	dehydrogenase family 5 member A1) (NAD(+)-dependent succinic semialdehyde dehydrogenase)										
P29699	Alpha-2-HS-glycoprotein (Countertrypin) (Fetuin-A)	Ahsg Fetua	11625	5.1339	0.016335	1.7869	0.1094	18.878725	20.8020275	18.9249625	20.465835
O35633	Vesicular inhibitory amino acid transporter (GABA and glycine transporter) (Solute carrier family 32 member 1) (Vesicular GABA transporter) (mVGAT) (mVIAAT)	Slc32a1 Vgat Viaat	22348	5.1391	0.016281	1.7883	0.1094	21.85051	21.69924	21.6162925	21.536195
O08529	Calpain-2 catalytic subunit (EC 3.4.22.53) (80 kDa M-calpain subunit) (CALP80) (Calcium-activated neutral proteinase 2) (CANP 2) (Calpain M-type) (Calpain-2 large subunit) (Millimolar-calpain) (M-calpain)	Capn2	12334	5.0957	0.016729	1.7765	0.11155	20.9928275	21.0884675	21.1473975	21.454575
Q9CPU4	Microsomal glutathione S-transferase 3 (Microsomal GST-3) (Glutathione peroxidase MGST3) (EC 1.11.1.-) (Microsomal glutathione S-transferase III) (Microsomal GST-III)	Mgst3	66447	5.0834	0.016858	1.7732	0.11192	20.7899	20.3282775	20.9891825	20.3395025
Q05816	Fatty acid-binding protein 5 (Epidermal-type fatty acid-binding protein) (E-FABP) (Fatty acid-binding protein, epidermal) (Keratinocyte lipid-binding protein) (Psoriasis-associated fatty acid-binding protein homolog) (PA-FABP)	Fabp5 Fabpe Klbp Mal1	16592	5.0646	0.017059	1.7681	0.11197	23.593135	23.3580075	23.6313625	23.67675
Q9Z2U1	Proteasome subunit alpha type-5 (EC 3.4.25.1) (Macropain zeta chain) (Multicatalytic endopeptidase complex zeta chain) (Proteasome zeta chain)	Psma5	26442	5.061	0.017097	1.7671	0.11197	21.828235	21.666245	21.750805	21.8207725
P57746	V-type proton ATPase subunit D (V-ATPase subunit D) (V-ATPase 28 kDa accessory protein) (Vacuolar proton pump subunit D)	Atp6v1d Atp6m Vatd	73834	5.0579	0.017131	1.7662	0.11197	23.4663325	23.61701	23.5663925	23.80509
Q61753	D-3-phosphoglycerate dehydrogenase (3-PGDH) (EC 1.1.1.95) (A10)	Phgdh	236539	5.0553	0.017158	1.7655	0.11197	23.27424	23.3939375	23.196055	23.5837025
Q60625	Intercellular adhesion molecule 5 (ICAM-5) (Telencephalin)	Icam5 Icam3 Tlcn	15898	5.0335	0.017395	1.7596	0.11266	23.2224475	23.090175	23.1767225	22.78432
Q8BLQ9	Cell adhesion molecule 2 (Immunoglobulin superfamily member 4D) (IgSF4D) (Nectin-like protein 3) (NECL-3) (Synaptic cell adhesion molecule 2) (SynCAM 2)	Cadm2 Igsf4d Necl3	239857	5.032	0.017412	1.7592	0.11266	23.454855	23.294	23.519685	23.13701
Q64105	Sepiapterin reductase (SPR) (EC 1.1.1.153)	Spr		4.9923	0.017854	1.7483	0.11287	21.95332	21.663005	21.8374275	21.7376375
P84084	ADP-ribosylation factor 5	Arf5	11844	4.9711	0.018095	1.7424	0.11287	21.8894825	21.701765	21.820265	21.5921125
P26883	Peptidyl-prolyl cis-trans isomerase FKBP1A (PPIase FKBP1A) (EC 5.2.1.8) (12 kDa FK506-binding protein) (12 kDa FKBP) (FKBP-12) (Calstabin-1) (FK506-binding protein 1A) (FKBP-1A) (Immunophilin FKBP12) (Rotamase)	Fkbp1a Fkbp1	14225	5.0184	0.017562	1.7554	0.11287	24.070705	23.8529725	23.8732825	23.7424725
P09103	Protein disulfide-isomerase (PDI) (EC 5.3.4.1) (Cellular thyroid hormone-binding protein) (Endoplasmic reticulum resident protein 59) (ER protein 59) (ERp59) (Prolyl 4-hydroxylase subunit beta) (p55)	P4hb Pdia1	18453	4.9671	0.018141	1.7413	0.11287	21.0979375	21.22766	21.2534325	21.4852225
P35293	Ras-related protein Rab-18	Rab18	19330	4.9678	0.018133	1.7415	0.11287	21.715335	21.81428	21.5383525	21.7513025
P62702	40S ribosomal protein S4, X isoform	Rps4x Rps4	20102	4.9634	0.018183	1.7403	0.11287	21.5735475	21.3925575	21.4896175	21.6516875
P08113	Endoplasmin (94 kDa glucose-regulated protein) (GRP-94) (Endoplasmic reticulum resident protein 99) (ERp99) (Heat shock protein 90 kDa beta member 1) (Polymorphic tumor rejection antigen 1) (Tumor rejection antigen gp96)	Hsp90b1 Grp94 Tra-1 Tra1	22027	5.0042	0.01772	1.7515	0.11287	23.19503	23.12479	23.2415225	23.3973625
Q9QYG0	Protein NDRG2 (N-myc downstream-regulated gene 2 protein) (Protein Ndr2)	Ndrg2 Kiaa1248 Ndr2	29811	4.9696	0.018112	1.742	0.11287	23.5586875	23.865035	23.75811	23.8634425

G5E829	Plasma membrane calcium-transporting ATPase 1 (EC 7.2.2.10) (Plasma membrane calcium ATPase isoform 1) (PMCA1) (Plasma membrane calcium pump isoform 1)	Atp2b1	67972	4.9691	0.018119	1.7419	0.11287	26.0039875	25.8739475	25.90278	25.77942
Q3ULJ0	Glycerol-3-phosphate dehydrogenase 1-like protein (EC 1.1.1.8)	Gpd1l Kiaa0089	333433	4.9712	0.018093	1.7425	0.11287	22.3588075	22.3205825	22.2518125	22.0853175
P61226	Ras-related protein Rap-2b	Rap2b	74012	4.9568	0.01826	1.7385	0.11289	22.6656175	22.4555275	22.6172175	22.3986125
P40124	Adenylyl cyclase-associated protein 1 (CAP 1)	Cap1 Cap	12331	4.9341	0.018526	1.7322	0.11407	24.298555	24.3078775	24.408485	24.4854875
Q5SRX1	TOM1-like protein 2 (Target of Myb-like protein 2)	Tom1l2	216810	4.915	0.018752	1.7269	0.115	21.9167375	22.201385	21.89811	22.13771
Q9JIG8	PRA1 family protein 2	Praf2 DXImx39e	54637	4.9041	0.018882	1.7239	0.11533	20.4244	20.172835	20.336645	20.05997
P63321	Ras-related protein Ral-A	Rala Ral Ral-a	56044	4.8972	0.018966	1.722	0.11538	22.4527375	22.240145	22.32121	22.248525
Q9D6U8	Protein FAM162A (E2-induced gene 5 protein homolog) (Growth and transformation-dependent protein) (HGTD-P)	Fam162a E2ig5	70186	4.835	0.019737	1.7047	0.1196	20.0379675	19.82956	19.9759575	19.638855
P59999	Actin-related protein 2/3 complex subunit 4 (Arp2/3 complex 20 kDa subunit) (p20-ARC)	Arpc4 Arc20	68089	4.8203	0.019925	1.7006	0.11979	23.94581	23.8375275	23.760935	23.703265
Q6PHN9	Ras-related protein Rab-35	Rab35	77407	4.8207	0.019921	1.7007	0.11979	21.04917	20.858105	21.081815	20.874
P60879	Synaptosomal-associated protein 25 (SNAP-25) (Super protein) (SUP) (Synaptosomal-associated 25 kDa protein)	Snap25 Snap	20614	4.7809	0.020438	1.6896	0.12191	25.440285	25.1915725	25.3940825	25.00534
Q6ZWN5	40S ribosomal protein S9	Rps9	76846	4.7862	0.020368	1.6911	0.12191	22.358525	22.385335	22.2929775	22.558845
P62242	40S ribosomal protein S8	Rps8	20116	4.7707	0.020573	1.6867	0.12224	21.7119325	21.8448825	21.8224675	22.123695
Q9Z140	Copine-6 (Copine VI) (Neuronal-copine) (N-copine)	Cpne6	12891	4.7331	0.021081	1.6761	0.12381	24.1304475	24.0500775	23.93543	23.8049975
Q6PER3	Microtubule-associated protein RP/EB family member 3 (EB1 protein family member 3) (EBF3) (End-binding protein 3) (EB3) (RP3)	Mapre3	100732	4.7429	0.020948	1.6789	0.12381	22.1657175	22.161015	22.1751425	22.0484125
Q8R3V5	Endophilin-B2 (SH3 domain-containing GRB2-like protein B2)	Sh3glb2 Kiaa1848	227700	4.7356	0.021047	1.6768	0.12381	22.35963	22.3901725	22.434155	22.2705175
Q62426	Cystatin-B (Stefin-B)	Cstb Cst6 Stfb	13014	4.7267	0.021169	1.6743	0.12385	20.4525	20.89147	20.7658625	21.18012
P02301	Histone H3.3C (Embryonic)	H3f3c Gm14384		4.7159	0.021319	1.6712	0.12425	25.1599025	25.30904	25.0927275	25.43784
P05480	Neuronal proto-oncogene tyrosine-protein kinase Src (EC 2.7.10.2) (Proto-oncogene c-Src) (pp60c-src) (p60-Src)	Src	20779	4.7041	0.021482	1.6679	0.12473	21.68244	21.4589875	21.5792925	21.34649
Q60605	Myosin light polypeptide 6 (17 kDa myosin light chain) (LC17) (Myosin light chain 3) (MLC-3) (Myosin light chain alkali 3) (Myosin light chain A3) (Smooth muscle and nonmuscle myosin light chain alkali 6)	Myl6 Myln	17904	4.6906	0.021672	1.6641	0.12498	22.4862425	22.5478575	22.59829	22.788455
Q8BPN8	DmX-like protein 2 (Rabconnectin-3)	Dmxl2 Kiaa0856	235380	4.6894	0.02169	1.6637	0.12498	23.593955	23.509045	23.563615	23.3480075
P53810	Phosphatidylinositol transfer protein alpha isoform (PI-TP-alpha) (PtdIns transfer protein alpha) (PtdInsTP alpha)	Pitpna Pitpn	18738	4.6572	0.02215	1.6546	0.12716	22.2865	22.1244125	22.3000125	22.1132325
P63005	Platelet-activating factor acetylhydrolase IB subunit alpha (Lissencephaly-1 protein) (LIS-1) (PAF acetylhydrolase 45 kDa subunit) (PAF-AH 45 kDa subunit) (PAF-AH alpha) (PAFAH alpha)	Pafah1b1 Lis-1 Lis1 Pafaha	18472	4.5914	0.02313	1.6358	0.13228	22.8326175	22.669995	22.694605	22.784755
P47962	60S ribosomal protein L5	Rpl5	100503670	4.5711	0.023442	1.63	0.13356	20.6605825	20.2151625	20.5735775	20.472785

Q9D8E6	60S ribosomal protein L4	Rpl4	67891	4.5309	0.024074	1.6184	0.13565	22.4456925	22.4633675	22.486515	22.6557075
Q91ZJ5	UTP--glucose-1-phosphate uridylyltransferase (EC 2.7.7.9) (UDP-glucose pyrophosphorylase) (UDPGP) (UGPase)	Ugp2	216558	4.5318	0.02406	1.6187	0.13565	21.3563675	21.5069775	21.522505	21.65388
P85094	Isochorismatase domain-containing protein 2A	Isoc2a Isoc2	664994	4.5342	0.024021	1.6194	0.13565	19.737965	20.3838525	20.145515	20.2585725
O89112	Glutathione S-transferase LANCL1 (EC 2.5.1.18) (40 kDa erythrocyte membrane protein) (p40) (LanC-like protein 1)	Lancl1 Gpr69a	14768	4.5121	0.024377	1.613	0.13685	21.76858	21.71156	21.6274	21.4480625
P62627	Dynein light chain roadblock-type 1 (Dynein light chain 2A, cytoplasmic)	Dynlrb1 Dncl2a Dnlc2a	67068	4.4767	0.024958	1.6028	0.1396	21.357645	21.1637575	21.509855	21.1899275
P02088	Hemoglobin subunit beta-1 (Beta-1-globin) (Hemoglobin beta-1 chain) (Hemoglobin beta-major chain)	Hbb-b1	100503605 101488143 15129	4.4606	0.025229	1.5981	0.14009	28.0426	27.3383725	27.757535	27.873235
Q9D415	Disks large-associated protein 1 (DAP-1) (Guanylate kinase-associated protein) (PSD-95/SAP90-binding protein 1) (SAP90/PSD-95-associated protein 1) (SAPAP1)	Dlgap1 Gkap Kiaa4162	224997	4.4618	0.025208	1.5985	0.14009	19.30364	18.6879525	18.987495	18.3513975
Q8QZT1	Acetyl-CoA acetyltransferase, mitochondrial (EC 2.3.1.9) (Acetoacetyl-CoA thiolase)	Acat1	110446	4.4507	0.025396	1.5952	0.14051	24.7344775	24.6296825	24.6758125	24.6053875
Q8R5M8	Cell adhesion molecule 1 (Immunoglobulin superfamily member 4) (IgSF4) (Nectin-like protein 2) (NECL-2) (Spermatogenic immunoglobulin superfamily) (SgIgSF) (Synaptic cell adhesion molecule) (SynCAM) (Tumor suppressor in lung cancer 1) (TSLC-1)	Cadm1 Igsf4 Necl2 Ra175 Syncam SynCam1 Tslc1	54725	4.4323	0.02571	1.5899	0.14173	22.3542825	22.255015	22.4242475	22.1056375
Q8R3Z5	Voltage-dependent L-type calcium channel subunit beta-1 (CAB1) (Calcium channel voltage-dependent subunit beta 1)	Cacnb1 Cacnlb1	12295	4.4105	0.026089	1.5835	0.14279	19.4441325	19.4798975	19.42252	18.8385875
Q9QXS6	Drebrin (Developmentally-regulated brain protein)	Dbn1 Drba	56320	4.4132	0.026042	1.5843	0.14279	24.0005925	23.9278975	23.96286	23.8182875
Q8K3J1	NADH dehydrogenase [ubiquinone] iron-sulfur protein 8, mitochondrial (EC 1.6.99.3) (EC 7.1.1.2) (Complex I-23kD) (CI-23kD) (NADH-ubiquinone oxidoreductase 23 kDa subunit)	Ndufs8	225887	4.3997	0.026279	1.5804	0.14331	22.09563	21.8576325	21.9285625	21.7252325
Q9D8W7	OCIA domain-containing protein 2	Ociad2	433904	4.3938	0.026383	1.5787	0.14337	20.8713425	20.794375	20.9777125	21.04034
P62852	40S ribosomal protein S25	Rps25	75617	4.3762	0.026698	1.5735	0.14457	20.863775	20.818035	20.6527975	21.0362925
Q6PH08	ERC protein 2 (CAZ-associated structural protein 1) (CAST1)	Erc2 Cast1 D14Ertd171e Kiaa0378	238988	4.3604	0.026985	1.5689	0.14561	20.924385	21.42662	20.7500125	21.24796
Q9JKR6	Hypoxia up-regulated protein 1 (GRP-170) (140 kDa Ca(2+)-binding protein) (CBP-140)	Hyou1 Grp170	12282	4.3207	0.02772	1.5572	0.14748	21.8124675	21.62012	21.830715	21.6797675
P28663	Beta-soluble NSF attachment protein (SNAP-beta) (Brain protein I47) (N-ethylmaleimide-sensitive factor attachment protein beta)	Napb Snapb	17957	4.3245	0.027649	1.5583	0.14748	25.03089	24.988275	25.14937	25.064265
P70175	Disks large homolog 3 (Synapse-associated protein 102) (SAP-102) (SAP102)	Dlg3 Dlgh3	53310	4.3225	0.027686	1.5577	0.14748	21.200495	21.1651575	21.1161875	20.825025
Q9CQI3	Glia maturation factor beta (GMF-beta)	Gmfb	63985	4.3264	0.027614	1.5589	0.14748	22.30503	22.0632375	22.3235725	21.8953575
O08917	Flotillin-1	Flot1	14251	4.2972	0.028166	1.5503	0.14921	21.79993	21.60186	21.54332	21.3680575
P10649	Glutathione S-transferase Mu 1 (EC 2.5.1.18) (GST 1-1) (GST class-mu 1) (Glutathione S-transferase GT8.7) (pmGT10)	Gstm1	14862	4.2912	0.028281	1.5485	0.14921	25.0651075	25.221155	24.950385	25.02841
Q9CPW4	Actin-related protein 2/3 complex subunit 5 (Arp2/3 complex 16 kDa subunit) (p16-ARC)	Arpc5	67771	4.2883	0.028337	1.5476	0.14921	22.4003475	22.261645	22.2540625	22.06224
Q9JI46	Diphosphoinositol polyphosphate phosphohydrolase 1 (DIPP-1) (muDIPP1) (EC 3.6.1.52) (Diadenosine 5',5'''-P1,P6-hexaphosphate hydrolase 1) (EC 3.6.1.-)	Nudt3 Dipp Dipp1	56409	4.2813	0.028473	1.5456	0.14941	21.6416825	21.374705	21.3852	21.2871225

	(Nucleoside diphosphate-linked moiety X motif 3) (Nudix motif 3)										
P45376	Aldo-keto reductase family 1 member B1 (EC 1.1.1.300) (EC 1.1.1.372) (EC 1.1.1.54) (Aldehyde reductase) (Aldo-keto reductase family 1 member B3) (Aldose reductase) (AR) (EC 1.1.1.21)	Akr1b1 Akr1b3 Aldor1 Aldr1 Alr2	11677	4.272	0.028654	1.5428	0.14985	22.9385275	23.01835	22.9162325	23.12233
Q9D0K2	Succinyl-CoA:3-ketoacid coenzyme A transferase 1, mitochondrial (EC 2.8.3.5) (3-oxoacid CoA-transferase 1) (Somatic-type succinyl-CoA:3-oxoacid CoA-transferase) (SCOT-s)	Oxct1 Oxct Scot	67041	4.2659	0.028773	1.541	0.14995	24.585	24.47894	24.5899325	24.3836725
P43277	Histone H1.3 (H1 VAR.4) (H1d)	H1-3 H1f3 Hist1h1d	14957	4.2592	0.028905	1.539	0.15013	24.1079125	24.248135	24.013935	24.4970675
P17751	Triosephosphate isomerase (TIM) (EC 5.3.1.1) (Methylglyoxal synthase) (EC 4.2.3.3) (Triose-phosphate isomerase)	Tpi1 Tpi	21991	4.2504	0.029079	1.5364	0.15026	27.00417	27.012755	27.01859	26.8939225
Q9DBF1	Alpha-aminoadipic semialdehyde dehydrogenase (Alpha-AASA dehydrogenase) (EC 1.2.1.31) (Aldehyde dehydrogenase family 7 member A1) (EC 1.2.1.3) (Antiquitin-1) (Betaine aldehyde dehydrogenase) (EC 1.2.1.8) (Delta1-piperideine-6-carboxylate dehydrogenase) (P6c dehydrogenase)	Aldh7a1 Ald7a1	110695	4.248	0.029128	1.5357	0.15026	21.7311675	21.9442	21.7732975	21.8722225
Q00623	Apolipoprotein A-I (Apo-AI) (ApoA-I) (Apolipoprotein A1) [Cleaved into: Proapolipoprotein A-I (ProapoA-I); Truncated apolipoprotein A-I]	Apoa1	11806	4.231	0.029468	1.5306	0.151	21.656485	22.5775475	21.0880375	22.4620975
P56379	ATP synthase subunit ATP5MPL, mitochondrial (6.8 kDa mitochondrial proteolipid protein) (MLQ)	Atp5mpl Mp68	70257	4.2324	0.02944	1.5311	0.151	21.29232	21.2348325	20.9525025	21.1120575
Q9Z1G4	V-type proton ATPase 116 kDa subunit a isoform 1 (V-ATPase 116 kDa isoform a1) (Clathrin-coated vesicle/synaptic vesicle proton pump 116 kDa subunit) (Vacuolar adenosine triphosphatase subunit Ac116) (Vacuolar proton pump subunit 1) (Vacuolar proton translocating ATPase 116 kDa subunit a isoform 1)	Atp6v0a1 Atp6n1	11975	4.2107	0.029882	1.5246	0.15207	25.51352	25.462215	25.5555425	25.34701
P56565	Protein S100-A1 (S-100 protein alpha chain) (S-100 protein subunit alpha) (S100 calcium-binding protein A1)	S100a1	20193	4.2013	0.030076	1.5218	0.15207	19.9778325	20.41802	20.31891	20.454835
O70400	PDZ and LIM domain protein 1 (C-terminal LIM domain protein 1) (Elfin) (LIM domain protein CLP-36)	Pdlim1 Clim1	54132	4.2019	0.030062	1.522	0.15207	17.329145	18.273045	18.1148025	18.4734175
E9PUL5	Proline-rich transmembrane protein 2 (Dispanin subfamily B member 3) (DSPB3)	Prrt2	69017	4.2113	0.029869	1.5248	0.15207	22.9753275	22.9201025	23.067805	22.722335
P21279	Guanine nucleotide-binding protein G(q) subunit alpha (Guanine nucleotide-binding protein alpha-q)	Gnaq	14682	4.1778	0.030566	1.5148	0.15362	23.21186	23.1873125	23.1207825	23.072645
Q9CZY3	Ubiquitin-conjugating enzyme E2 variant 1 (UEV-1) (CROC-1)	Ube2v1 Croc1	66589	4.177	0.030583	1.5145	0.15362	22.5050725	22.3583325	22.4242525	22.30689
Q8BXR1	Probable cationic amino acid transporter (Solute carrier family 7 member 14)	Slc7a14 Kiaa1613	241919	4.1669	0.030796	1.5115	0.15418	20.262785	20.1770925	20.50461	19.9999725
Q61490	CD166 antigen (Activated leukocyte cell adhesion molecule) (BEN) (Protein DM-GRASP) (CD antigen CD166)	Alcam	11658	4.1562	0.031024	1.5083	0.15437	21.962425	21.944905	22.1133575	21.7700125
P61205	ADP-ribosylation factor 3	Arf3	11842	4.1424	0.031323	1.5041	0.15437	26.06632	26.0547	26.076005	25.89578
P51410	60S ribosomal protein L9	Rpl9	20005	4.1369	0.031441	1.5025	0.15437	20.1947175	20.6071475	20.4581175	20.7743025
Q9Z239	Phospholemman (FXYD domain-containing ion transport regulator 1) (Sodium/potassium-transporting ATPase subunit FXYD1)	Fxyd1 Plm	56188	4.1486	0.031187	1.506	0.15437	19.326555	19.59019	19.348645	19.9567175

Q8K2B3	Succinate dehydrogenase [ubiquinone] flavoprotein subunit, mitochondrial (EC 1.3.5.1) (Flavoprotein subunit of complex II) (Fp)	Sdha	66945	4.1413	0.031346	1.5038	0.15437	24.104415	24.1934725	24.1865625	24.22722
Q64522	Histone H2A type 2-B (H2a-613A)	Hist2h2ab	621893	4.1463	0.031237	1.5053	0.15437	21.4207025	21.738595	21.7081225	21.7323975
P00920	Carbonic anhydrase 2 (EC 4.2.1.1) (Carbonate dehydratase II) (Carbonic anhydrase II) (CA-II)	Ca2 Car2	12349	4.1241	0.031721	1.4987	0.15525	24.66847	24.7045775	24.5362275	25.000225
Q8BVE3	V-type proton ATPase subunit H (V-ATPase subunit H) (Vacuolar proton pump subunit H)	Atp6v1h	108664	4.1139	0.031946	1.4956	0.15585	24.4371675	24.42384	24.54675	24.3744875
Q8VHH5	Arf-GAP with GTPase, ANK repeat and PH domain-containing protein 3 (AGAP-3) (CRAM-associated GTPase) (CRAG) (Centaurin-gamma-3) (Cnt-g3) (MR1-interacting protein) (MRIP-1)	Agap3 Centg3		4.1041	0.032164	1.4926	0.15592	20.4081275	20.305595	20.33144	19.986425
Q91VR7	Microtubule-associated proteins 1A/1B light chain 3A (Autophagy-related protein LC3 A) (Autophagy-related ubiquitin-like modifier LC3 A) (MAP1 light chain 3-like protein 1) (MAP1A/MAP1B light chain 3 A) (MAP1A/MAP1B LC3 A) (Microtubule-associated protein 1 light chain 3 alpha)	Map1lc3a	66734	4.1072	0.032096	1.4935	0.15592	21.3499625	21.07012	21.5351775	21.0764625
P63213	Guanine nucleotide-binding protein G(I)/G(S)/G(O) subunit gamma-2 (G gamma-I)	Gng2	14702	4.0896	0.032489	1.4883	0.15675	23.5824275	23.36512	23.477685	23.3188325
Q9CQW1	Synaptobrevin homolog YKT6 (EC 2.3.1.-)	Ykt6	56418	4.0873	0.032541	1.4876	0.15675	21.1970575	21.62955	21.2786875	21.60704
P68372	Tubulin beta-4B chain (Tubulin beta-2C chain)	Tubb4b Tubb2c	227613	4.0568	0.033241	1.4783	0.15962	27.4741725	27.4272775	27.6323925	27.3575375
Q8BJI1	Sodium-dependent neutral amino acid transporter SLC6A17 (Sodium-dependent neurotransmitter transporter NTT4) (Solute carrier family 6 member 17)	Slc6a17 Ntt4	229706	4.0444	0.033531	1.4746	0.16051	20.81729	20.7477825	21.0452475	20.8504675
O55234	Proteasome subunit beta type-5 (EC 3.4.25.1) (Macropain epsilon chain) (Multicatalytic endopeptidase complex epsilon chain) (Proteasome chain 6) (Proteasome epsilon chain) (Proteasome subunit X)	Psmb5	19173	4.0336	0.033784	1.4713	0.16071	21.99336	21.85695	21.7779375	21.9580625
Q9D1T0	Leucine-rich repeat and immunoglobulin-like domain-containing nogo receptor-interacting protein 1 (Leucine-rich repeat neuronal protein 1) (Leucine-rich repeat neuronal protein 6A)	Lingo1 Lern1 Lrrn6a	235402	4.0338	0.033781	1.4713	0.16071	19.6665575	19.1845225	19.8311825	19.6794575
O55126	Protein NipSnap homolog 2 (NipSnap2) (Glioblastoma-amplified sequence)	Nipsnap2 Gbas		4.0234	0.034028	1.4682	0.16104	19.818475	20.00578	18.947365	19.9962225
Q8K0E8	Fibrinogen beta chain [Cleaved into: Fibrinopeptide B; Fibrinogen beta chain]	Fgb	110135	4.0174	0.034169	1.4664	0.16104	19.4264675	21.1172675	18.8847925	20.169645
Q641P0	Actin-related protein 3B (ARP3-beta) (Actin-like protein 3B)	Actr3b	242894	4.0194	0.034123	1.467	0.16104	20.5029725	20.62635	20.803885	20.322425
Q61301	Catenin alpha-2 (Alpha N-catenin)	Ctnna2 Catna2	12386	3.9956	0.034697	1.4597	0.16302	22.76489	22.7446075	22.92369	22.6170775
P47964	60S ribosomal protein L36	Rpl36		3.9813	0.035048	1.4553	0.1632	19.4947075	19.169765	19.5041725	19.362525
P49615	Cyclin-dependent-like kinase 5 (EC 2.7.11.1) (CR6 protein kinase) (CRK6) (Cell division protein kinase 5) (Serine/threonine-protein kinase PSSALRE) (Tau protein kinase II catalytic subunit) (TPKII catalytic subunit)	Cdk5 Cdkn5 Crk6	12568	3.981	0.035055	1.4553	0.1632	20.3483575	20.398785	20.51884	20.1342725
Q99JI6	Ras-related protein Rap-1b (GTP-binding protein smg p21B)	Rap1b	215449	3.9853	0.034951	1.4565	0.1632	20.6673475	20.3543125	20.5801475	20.3325575
O88487	Cytoplasmic dynein 1 intermediate chain 2 (Cytoplasmic dynein intermediate chain 2) (Dynein intermediate chain 2, cytosolic) (DH IC-2)	Dync1i2 Dnci2 Dncic2	13427	3.9697	0.035335	1.4518	0.164	19.5286475	19.407	19.7675225	19.424095

P99027	60S acidic ribosomal protein P2	Rplp2	67186	3.9646	0.035462	1.4502	0.16409	21.69745	21.873985	21.8231375	21.9883425
Q3TES0	IQ motif and SEC7 domain-containing protein 3	Iqsec3 Kiaa1110	243621	3.9578	0.035635	1.4481	0.16439	20.0818575	19.8901425	20.35084	19.8232125
P12960	Contactin-1 (Neural cell surface protein F3)	Cntn1	12805	3.9289	0.036368	1.4393	0.16528	25.5376775	25.4788075	25.539065	25.43512
P35486	Pyruvate dehydrogenase E1 component subunit alpha, somatic form, mitochondrial (EC 1.2.4.1) (PDHE1-A type I)	Pdha1 Pdha-1	18597	3.9392	0.036105	1.4424	0.16528	25.421295	25.3024875	25.3356625	25.28483
P47857	ATP-dependent 6-phosphofructokinase, muscle type (ATP-PFK) (PFK-M) (EC 2.7.1.11) (6-phosphofructokinase type A) (Phosphofructo-1-kinase isozyme A) (PFK-A) (Phosphohexokinase)	Pfkm Pfk-m Pfka	18642	3.9301	0.036339	1.4396	0.16528	24.5935775	24.59713	24.6141875	24.6712175
Q91XM9	Disks large homolog 2 (Channel-associated protein of synapse-110) (Chapsyn-110) (Postsynaptic density protein PSD-93)	Dlg2 Dlgh2	23859	3.9369	0.036163	1.4417	0.16528	23.238465	23.286715	23.33615	23.0706075
Q8BWG8	Beta-arrestin-1 (Arrestin beta-1)	Arrb1	109689	3.9424	0.036023	1.4434	0.16528	19.8824425	20.0001675	20.1540225	20.2812025
P84075	Neuron-specific calcium-binding protein hippocalcin	Hpca	15444	3.9208	0.03658	1.4368	0.16575	24.4470725	24.3502325	24.18469	24.1375
P63037	DnaJ homolog subfamily A member 1 (DnaJ protein homolog 2) (Heat shock 40 kDa protein 4) (Heat shock protein J2) (HSJ-2)	Dnaja1 Dnaj2 Hsj2 Hspf4	15502	3.9152	0.036724	1.435	0.16576	21.4000075	21.308515	21.428765	21.3488
P62082	40S ribosomal protein S7	Rps7	20115	3.9083	0.036904	1.4329	0.16576	21.6844225	21.9427575	22.07107	22.1506875
P60487	Pyridoxal phosphate phosphatase (PLP phosphatase) (EC 3.1.3.3) (EC 3.1.3.74) (Chronophin)	Pdxp Cin Plp Plpp	57028	3.9082	0.036908	1.4329	0.16576	22.90741	22.908135	22.8262625	22.69401
Q925N0	Sideroflexin-5	Sfxn5	94282	3.8693	0.037943	1.4209	0.16991	22.2207225	22.565985	22.463155	22.67241
P51174	Long-chain specific acyl-CoA dehydrogenase, mitochondrial (LCAD) (EC 1.3.8.8)	Acadl	11363	3.8426	0.038673	1.4126	0.17002	21.88628	21.93903	21.6986	22.02244
P10126	Elongation factor 1-alpha 1 (EF-1-alpha-1) (Elongation factor Tu) (EF-Tu) (Eukaryotic elongation factor 1 A-1) (eEF1A-1)	Eef1a1 Eef1a	13627	3.845	0.038606	1.4133	0.17002	24.9636725	25.10958	25.28692	25.4111575
O35465	Peptidyl-prolyl cis-trans isomerase FKBP8 (PPIase FKBP8) (EC 5.2.1.8) (38 kDa FK506-binding protein) (38 kDa FKBP) (FKBP-38) (mFKBP38) (FK506-binding protein 8) (FKBP-8) (FKBPR38) (Rotamase)	Fkbp8 Fkbp38 Sam11	14232	3.8596	0.038205	1.4179	0.17002	20.01131	19.8165525	20.1045175	19.92662
Q8C854	Myelin expression factor 2 (MEF-2) (MyEF-2)	Myef2 Kiaa1341 Mef2	17876	3.8492	0.038491	1.4146	0.17002	20.292365	20.0835975	20.2531175	20.0565425
O35864	COP9 signalosome complex subunit 5 (SGN5) (Signalosome subunit 5) (EC 3.4.-.-) (Jun activation domain-binding protein 1) (Kip1 C-terminus-interacting protein 2)	Cops5 Csn5 Jab1 Kic2	26754	3.8524	0.038402	1.4156	0.17002	20.493245	20.46406	20.2939175	20.548685
P63046	Sulfotransferase 4A1 (ST4A1) (EC 2.8.2.-) (Brain sulfotransferase-like protein) (mBR-STL) (Nervous system sulfotransferase) (NST)	Sult4a1 Sultx3	29859	3.8399	0.038746	1.4118	0.17002	19.855485	19.27434	19.7530275	19.710975
Q4KMM3	Oxidation resistance protein 1 (Protein C7)	Oxr1 C7 Gm1238	170719	3.8423	0.038679	1.4125	0.17002	24.0313275	23.890905	23.956675	23.8637025
P51880	Fatty acid-binding protein, brain (Brain lipid-binding protein) (BLBP) (Brain-type fatty acid-binding protein) (B-FABP) (Fatty acid-binding protein 7)	Fabp7 Blbp	12140	3.8243	0.039181	1.4069	0.17094	20.3816525	20.431025	20.1979975	20.92649
Q8VDM4	26S proteasome non-ATPase regulatory subunit 2 (26S proteasome regulatory subunit RPN1) (26S proteasome regulatory subunit S2) (26S proteasome subunit p97)	Psmd2	21762	3.826	0.039135	1.4074	0.17094	21.704395	21.77119	21.9021925	21.8305225
Q9CQE8	RNA transcription, translation and transport factor protein	RTRAF	68045	3.8114	0.039546	1.4029	0.17155	19.3797625	19.6772125	19.7453125	19.80543

P62751	60S ribosomal protein L23a	Rpl23a	268449	3.8138	0.039477	1.4037	0.17155	20.968465	20.8982	20.82619	21.1327775
P54823	Probable ATP-dependent RNA helicase DDX6 (EC 3.6.4.13) (ATP-dependent RNA helicase p54) (DEAD box protein 6) (Oncogene RCK homolog)	Ddx6 Hlr2 Rck	13209	3.8037	0.039764	1.4005	0.17201	20.2390475	19.9362	20.1443975	20.259925
P35980	60S ribosomal protein L18	Rpl18	19899	3.799	0.0399	1.399	0.17211	21.811645	21.8599375	21.8449325	22.02319
P11983	T-complex protein 1 subunit alpha (TCP-1-alpha) (CCT-alpha) (Tailless complex polypeptide 1A) (TCP-1-A) (Tailless complex polypeptide 1B) (TCP-1-B)	Tcp1 Cct1 Ccta	21454	3.7891	0.040186	1.3959	0.17283	23.46845	23.3151275	23.5253925	23.3562125
Q9CX86	Heterogeneous nuclear ribonucleoprotein A0 (hnRNP A0)	Hnrnpa0 Hnrpa0	77134	3.7854	0.040292	1.3948	0.17283	20.3095925	20.623265	20.302525	20.391625
O35381	Acidic leucine-rich nuclear phosphoprotein 32 family member A (Acidic nuclear phosphoprotein pp32) (Leucine-rich acidic nuclear protein) (LANP) (Potent heat-stable protein phosphatase 2A inhibitor I1PP2A)	Anp32a Anp32 Lanp	11737	3.7737	0.040631	1.3911	0.17305	22.3834975	22.3557425	22.38358	22.48872
P08226	Apolipoprotein E (Apo-E)	Apoe	11816	3.7706	0.040723	1.3902	0.17305	22.941915	23.2766725	22.80558	23.4734775
Q9Z218	Dipeptidyl aminopeptidase-like protein 6 (DPPX) (Dipeptidyl aminopeptidase-related protein) (Dipeptidyl peptidase 6) (Dipeptidyl peptidase IV-like protein) (Dipeptidyl peptidase VI) (DPP VI)	Dpp6 Dpp-6	13483	3.7648	0.040893	1.3884	0.17305	23.8657725	23.8406375	23.748195	23.5661425
P63028	Translationally-controlled tumor protein (TCTP) (21 kDa polypeptide) (p21) (p23)	Tpt1 Trt	22070	3.7604	0.041025	1.387	0.17305	22.11244	22.2426225	22.35217	22.4868475
Q8BYM8	Probable cysteine--tRNA ligase, mitochondrial (EC 6.1.1.16) (Cysteinyl-tRNA synthetase) (CysRS)	Cars2	71941	3.7631	0.040943	1.3878	0.17305	19.635255	18.98585	19.1966575	18.4534625
Q8VCW8	Medium-chain acyl-CoA ligase ACSF2, mitochondrial (EC 6.2.1.2)	Acsf2	264895	3.7636	0.040929	1.388	0.17305	19.81982	20.067985	19.8483175	20.1243275
P80316	T-complex protein 1 subunit epsilon (TCP-1-epsilon) (CCT-epsilon)	Cct5 Ccte Kiaa0098	12465	3.7478	0.041399	1.383	0.17415	22.52372	22.5131075	22.68484	22.605565
O88935	Synapsin-1 (Synapsin I)	Syn1 Syn-1	20964	3.7374	0.041711	1.3798	0.17498	27.2228675	27.189305	27.2275975	27.0246575
Q08331	Calretinin (CR)	Calb2	12308	3.7278	0.042002	1.3767	0.17543	22.9156375	22.90711	22.76546	22.408515
P97351	40S ribosomal protein S3a (Protein TU-11)	Rps3a Rps3a1	20091	3.7263	0.042047	1.3763	0.17543	22.7653075	22.6677425	22.73999	22.8599725
P30416	Peptidyl-prolyl cis-trans isomerase FKBP4 (PPIase FKBP4) (EC 5.2.1.8) (52 kDa FK506-binding protein) (52 kDa FKBP) (FKBP-52) (59 kDa immunophilin) (p59) (FK506-binding protein 4) (FKBP-4) (FKBP59) (HSP-binding immunophilin) (HBI) (Immunophilin FKBP52) (Rotamase) [Cleaved into: Peptidyl-prolyl cis-trans isomerase FKBP4, N-terminally processed]	Fkbp4 Fkpb52	14228	3.7166	0.042345	1.3732	0.17619	20.5980725	20.12478	20.38351	20.3528525
P35564	Calnexin	Canx	12330	3.6943	0.043034	1.3662	0.17857	23.4236925	23.4726425	23.4522975	23.61585
P56375	Acylphosphatase-2 (EC 3.6.1.7) (Acylphosphatase, muscle type isozyme) (Acylphosphate phosphohydrolase 2)	Acyp2 Acyp	75572	3.6818	0.043428	1.3622	0.17971	20.16361	19.9362675	19.84672	19.90754
P14685	26S proteasome non-ATPase regulatory subunit 3 (26S proteasome regulatory subunit RPN3) (26S proteasome regulatory subunit S3) (Proteasome subunit p58) (Transplantation antigen P91A) (Tum-P91A antigen)	Psmd3 P91a Tstap91a	22123	3.6661	0.043928	1.3573	0.18129	20.48252	20.47367	20.6995925	20.6607175
P62334	26S proteasome regulatory subunit 10B (26S proteasome AAA-ATPase subunit RPT4)	Psmc6 Sug2	67089	3.6572	0.044215	1.3544	0.18198	19.19784	18.956145	19.4135075	19.0817375

	(Proteasome 26S subunit ATPase 6) (Proteasome subunit p42)										
Q99LC5	Electron transfer flavoprotein subunit alpha, mitochondrial (Alpha-ETF)	Etfa	110842	3.6479	0.044515	1.3515	0.18273	22.7062125	22.8862625	22.664585	22.90653
Q61166	Microtubule-associated protein RP/EB family member 1 (APC-binding protein EB1) (End-binding protein 1) (EB1)	Mapre1	13589	3.6337	0.04498	1.347	0.1838	20.87944	21.159015	20.966465	21.1749475
P04925	Major prion protein (PrP) (PrP27-30) (PrP33-35C) (CD antigen CD230)	Prnp Prn-p Prp	19122	3.6325	0.045018	1.3466	0.1838	21.817945	21.808145	21.481925	21.3655325
Q60631	Growth factor receptor-bound protein 2 (Adapter protein GRB2) (SH2/SH3 adapter GRB2)	Grb2	14784	3.625	0.045269	1.3442	0.18433	21.4911925	21.41314	21.54497	21.5254025
P83741	Serine/threonine-protein kinase WNK1 (EC 2.7.11.1) (Protein kinase lysine-deficient 1) (Protein kinase with no lysine 1)	Wnk1 Hsn2 Prkwnk1	232341	3.6075	0.045852	1.3386	0.18621	19.0608625	19.3991875	18.706135	19.6261475
P23242	Gap junction alpha-1 protein (Connexin-43) (Cx43) (Gap junction 43 kDa heart protein)	Gja1 Cxn-43	14609	3.6017	0.046049	1.3368	0.18652	21.5193975	21.979095	21.45263	22.0656425
Q8K400	Syntaxin-binding protein 5 (Lethal(2) giant larvae protein homolog 3) (Tomosyn-1)	Stxbp5 Kiaa4253 Llgl3	78808	3.5931	0.046341	1.334	0.1872	20.6157275	20.8173075	20.521065	20.719795
Q3TXX4	Vesicular glutamate transporter 1 (VGluT1) (Brain-specific Na(+)-dependent inorganic phosphate cotransporter) (Solute carrier family 17 member 7)	Slc17a7 Bnpi Vglut1	72961	3.5861	0.046578	1.3318	0.18767	22.7442075	22.6171225	22.77191	22.48421
P62264	40S ribosomal protein S14	Rps14	20044	3.5818	0.046727	1.3304	0.18777	21.8524325	21.7418525	21.7305325	21.959755
P70195	Proteasome subunit beta type-7 (EC 3.4.25.1) (Macropain chain Z) (Multicatalytic endopeptidase complex chain Z) (Proteasome subunit Z)	Psmb7 Mmc14	19177	3.5687	0.047179	1.3263	0.18812	20.5640975	20.2822225	20.451395	20.3401625
P01831	Thy-1 membrane glycoprotein (Thy-1 antigen) (CD antigen CD90)	Thy1 Thy-1	21838	3.5612	0.047441	1.3238	0.18812	25.8747275	25.921735	25.7875625	25.840695
Q9Z2Y3	Homer protein homolog 1 (Homer-1) (VASP/Ena-related gene up-regulated during seizure and LTP 1) (Vesl-1)	Homer1 Vesl1	26556	3.5545	0.047676	1.3217	0.18812	22.6644825	22.527695	22.5761575	22.3929475
P56135	ATP synthase subunit f, mitochondrial (ATP synthase membrane subunit f)	Atp5mf Atp5j2	57423	3.5631	0.047375	1.3245	0.18812	23.541715	23.30788	23.64429	23.30038
Q9DB41	Mitochondrial glutamate carrier 2 (GC-2) (Glutamate/H(+) symporter 2) (Solute carrier family 25 member 18)	Slc25a18 Gc2	71803	3.5661	0.047269	1.3254	0.18812	19.7301125	20.1022975	19.7049125	20.1381025
Q8R5H6	Wiskott-Aldrich syndrome protein family member 1 (WASP family protein member 1) (Protein WAVE-1)	Wasf1 Wave1	83767	3.5632	0.047372	1.3245	0.18812	22.1660825	21.95277	22.0464825	21.5613175
P61358	60S ribosomal protein L27	Rpl27	108167922 19942	3.5578	0.04756	1.3228	0.18812	21.0474075	21.0021725	20.92366	21.1817575
Q9R1P0	Proteasome subunit alpha type-4 (EC 3.4.25.1) (Macropain subunit C9) (Multicatalytic endopeptidase complex subunit C9) (Proteasome component C9) (Proteasome subunit L)	Psma4	26441	3.5341	0.048402	1.3151	0.19049	21.4532375	21.8364525	21.8746225	22.024135
P26638	Serine--tRNA ligase, cytoplasmic (EC 6.1.1.11) (Seryl-tRNA synthetase) (SerRS) (Seryl-tRNA(Ser/Sec) synthetase)	Sars1 Sars Sers	20226	3.5246	0.048742	1.3121	0.19133	22.7159025	22.768615	22.887375	22.9085675
Q9CQ91	NADH dehydrogenase [ubiquinone] 1 alpha subcomplex subunit 3 (Complex I-B9) (CI-B9) (NADH-ubiquinone oxidoreductase B9 subunit)	Ndufa3	66091	3.4965	0.04977	1.303	0.19192	20.8993175	20.7337275	21.11516	20.6668425
Q6PGN3	Serine/threonine-protein kinase DCLK2 (EC 2.7.11.1) (CaMK-like CREB regulatory kinase 2) (CL2) (CLICK-II) (CLICK2) (Doublecortin-like and CAM kinase-like 2) (Doublecortin-like kinase 2)	Dclk2 Dcamkl2	70762	3.5028	0.049538	1.3051	0.19192	19.7687425	19.57787	19.852185	19.4707125
Q5SQX6	Cytoplasmic FMR1-interacting protein 2 (p53-inducible protein 121)	Cyfip2 Kiaa1168 Pir121	76884	3.5144	0.049114	1.3088	0.19192	23.8758075	23.74044	23.840915	23.66745
Q920I9	WD repeat-containing protein 7 (TGF-beta resistance-associated protein TRAG)	Wdr7 Kiaa0541 Trag	104082	3.5041	0.049488	1.3055	0.19192	23.03171	22.963135	23.1602925	22.946915

Q7TPR4	Alpha-actinin-1 (Alpha-actinin cytoskeletal isoform) (F-actin cross-linking protein) (Non-muscle alpha-actinin-1)	Actn1	109711	3.4969	0.049754	1.3032	0.19192	24.629155	24.8006575	24.88595	24.8183975
Q922B2	Aspartate--tRNA ligase, cytoplasmic (EC 6.1.1.12) (Aspartyl-tRNA synthetase) (AspRS)	Dars1 Dars	226414	3.4987	0.049687	1.3038	0.19192	22.201035	22.170285	22.1374675	22.3098775
Q8C419	Probable G-protein coupled receptor 158	Gpr158 Kiaa1136	241263	3.5004	0.049624	1.3043	0.19192	20.626025	20.615835	20.5248225	20.18206
P29758	Ornithine aminotransferase, mitochondrial (EC 2.6.1.13) (Ornithine--oxo-acid aminotransferase)	Oat	18242	3.4865	0.05014	1.2998	0.19286	21.3202425	21.594395	21.45038	21.547295
Q62318	Transcription intermediary factor 1-beta (TIF1-beta) (E3 SUMO-protein ligase TRIM28) (EC 2.3.2.27) (KRAB-A-interacting protein) (KRIP-1) (RING-type E3 ubiquitin transferase TIF1-beta) (Tripartite motif-containing protein 28)	Trim28 Kap1 Krip1 Tif1b	21849	3.4812	0.05034	1.2981	0.19314	20.227855	20.0176075	20.2224	20.0325575
P63017	Heat shock cognate 71 kDa protein (Heat shock 70 kDa protein 8)	Hspa8 Hsc70 Hsc73	15481	3.4739	0.050613	1.2957	0.1937	27.8835525	27.8862075	27.984185	27.96048
P62761	Visinin-like protein 1 (VILIP) (Neural visinin-like protein 1) (NVL-1) (NVP-1)	Vsnl1 Visl1	26950	3.4694	0.050784	1.2943	0.19387	25.3018025	25.1937275	25.402355	25.221295
P17710	Hexokinase-1 (EC 2.7.1.1) (Hexokinase type I) (HK I) (Hexokinase, tumor isozyme)	Hk1	15275	3.4577	0.051229	1.2905	0.19508	26.8157775	26.7858025	26.8601	26.7471575
Q80UW2	F-box only protein 2	Fbxo2 Fbs1 Fbx2	230904	3.4509	0.051492	1.2883	0.19559	21.3467275	21.5165675	21.37996	21.59901
Q9D2P8	Myelin-associated oligodendrocyte basic protein	Mobp	17433	3.4294	0.052326	1.2813	0.19632	20.7283275	20.6451325	20.12946	20.834645
P62849	40S ribosomal protein S24	Rps24	20088	3.4381	0.051985	1.2841	0.19632	20.2494025	20.2207175	20.5380175	20.388025
Q62442	Vesicle-associated membrane protein 1 (VAMP-1) (Synaptobrevin-1)	Vamp1 Syb1	22317	3.436	0.052066	1.2834	0.19632	19.914535	19.47318	19.789425	19.40254
P26040	Ezrin (Cytovillin) (Villin-2) (p81)	Ezr Vil2	22350	3.4349	0.052111	1.2831	0.19632	21.4915575	21.5961	21.5815175	22.010685
Q922R8	Protein disulfide-isomerase A6 (EC 5.3.4.1) (Thioredoxin domain-containing protein 7)	Pdia6 Txndc7	71853	3.4306	0.052278	1.2817	0.19632	21.6891525	21.653405	21.7410975	21.84885
P31324	cAMP-dependent protein kinase type II-beta regulatory subunit	Prkar2b	19088	3.4252	0.052489	1.2799	0.19645	22.8736	22.92157	23.159545	22.76681
P62631	Elongation factor 1-alpha 2 (EF-1-alpha-2) (Eukaryotic elongation factor 1 A-2) (eEF1A-2) (Statin-S1)	Eef1a2 Eef1al Stn	13628	3.4145	0.052913	1.2764	0.1973	26.25261	26.2663375	26.28864	26.4771625
P43274	Histone H1.4 (H1 VAR.2) (H1e)	H1-4 H1f4 Hist1h1e	50709	3.4129	0.052975	1.2759	0.1973	22.69354	22.7324925	22.423975	22.9016625
P62806	Histone H4	H4c1 Hist1h4a; H4c2 H4-53 Hist1h4b; H4c3 H4-12 Hist1h4c; H4c4 Hist1h4d; H4c6 Hist1h4f; H4c8 Hist1h4h; H4c9 Hist1h4i; H4c11 Hist1h4j; H4c12 Hist1h4k; Hist1h4m; H4c14 Hist2h4 Hist2h4a; H4f16 Hist4h4	100041230<br>319155<br>319156<br>319157<br>319158<br>319159<br>319160<br>319161<br>320332<br>326619<br>326620<br>69386 97122	3.4076	0.053188	1.2742	0.19761	27.2961575	27.451455	27.2519275	27.4032025
Q9Z2E3	Serine/threonine-protein kinase/endoribonuclease IRE2 (Endoplasmic reticulum-to-nucleus signaling 2) (Inositol-requiring protein 2) (Ire1-beta) (IRE1b) (mIre1) [Includes: Serine/threonine-protein kinase (EC 2.7.11.1); Endoribonuclease (EC 3.1.26.-)]	Ern2 Ire2	26918	3.3934	0.05376	1.2695	0.19925	21.5288675	20.9462275	21.1634525	20.201965
Q8BRT1	CLIP-associating protein 2 (Cytoplasmic linker-associated protein 2)	Clasp2 Kiaa0627	76499	3.3873	0.054007	1.2676	0.19968	20.793025	20.7859525	20.8735725	20.64909
Q06185	ATP synthase subunit e, mitochondrial (ATPase subunit e) (ATP synthase membrane subunit e)	Atp5me Atp5i Atp5k Lfm-1 Lfm1	11958	3.3801	0.054299	1.2652	0.20005	23.70784	23.6290375	23.4794625	23.6508575

Q3UM45	Protein phosphatase 1 regulatory subunit 7 (Protein phosphatase 1 regulatory subunit 22)	Ppp1r7 Sds22	66385	3.3755	0.054487	1.2637	0.20005	22.4492525	22.35725	22.4906825	22.202785
Q91YQ5	Dolichyl-diphosphooligosaccharide--protein glycosyltransferase subunit 1 (Dolichyl-diphosphooligosaccharide--protein glycosyltransferase 67 kDa subunit) (Ribophorin I) (RPN-I) (Ribophorin-1)	Rpn1	103963	3.3752	0.054498	1.2636	0.20005	20.7946975	20.8415	20.632625	21.08909
Q8K596	Sodium/calcium exchanger 2 (Na(+)/Ca(2+)-exchange protein 2) (Solute carrier family 8 member 2)	Slc8a2 Ncx2	110891	3.3672	0.054832	1.261	0.20079	22.016905	21.84551	22.0181875	21.6099175
Q02248	Catenin beta-1 (Beta-catenin)	Ctnnb1 Catnb	12387	3.3396	0.055985	1.2519	0.20236	22.6513325	22.5744025	22.698275	22.5040675
P70168	Importin subunit beta-1 (Karyopherin subunit beta-1) (Nuclear factor p97) (Pore targeting complex 97 kDa subunit) (PTAC97) (SCG)	Kpnb1 Impnb	16211	3.3416	0.055899	1.2526	0.20236	22.505245	22.5638775	22.587485	22.698425
Q61644	Protein kinase C and casein kinase substrate in neurons protein 1 (Syndapin-1)	Pacsin1 Pacsin	23969	3.3509	0.05551	1.2556	0.20236	25.07617	24.92785	24.98797	24.8453225
Q91Z53	Glyoxylate reductase/hydroxypyruvate reductase (EC 1.1.1.79) (EC 1.1.1.81)	Grhpr Glxr	76238	3.3384	0.056038	1.2515	0.20236	20.9215375	20.9672225	20.7263375	20.8444125
Q6PDM2	Serine/arginine-rich splicing factor 1 (ASF/SF2) (Pre-mRNA-splicing factor SRp30a) (Splicing factor, arginine/serine-rich 1)	Srsf1 Sfrs1	110809	3.338	0.056053	1.2514	0.20236	20.9102825	20.3389575	20.825645	20.50157
P35282	Ras-related protein Rab-21 (Rab-12)	Rab21	216344	3.3379	0.056056	1.2514	0.20236	20.811285	20.6130575	20.693175	20.768055
Q9QZM0	Ubiquilin-2 (Chap1) (DSK2 homolog) (Protein linking IAP with cytoskeleton 2) (PLIC-2) (Ubiquitin-like product Chap1/Dsk2)	Ubqln2 Plic2	54609	3.3197	0.056834	1.2454	0.20468	21.8182025	21.62921	21.881075	21.63612
Q9QYC0	Alpha-adducin (Erythrocyte adducin subunit alpha)	Add1	11518	3.3148	0.057048	1.2438	0.20497	24.51586	24.4478275	24.5015675	24.4488725
P19783	Cytochrome c oxidase subunit 4 isoform 1, mitochondrial (Cytochrome c oxidase polypeptide IV) (Cytochrome c oxidase subunit IV isoform 1) (COX IV-1)	Cox4i1 Cox4 Cox4a	12857	3.3065	0.057409	1.241	0.20559	25.094515	24.9118	24.9971325	24.968355
Q9Z1B3	1-phosphatidylinositol 4,5-bisphosphate phosphodiesterase beta-1 (EC 3.1.4.11) (PLC-154) (Phosphoinositide phospholipase C-beta-1) (Phospholipase C-beta-1) (PLC-beta-1)	Plcb1 Plcb	18795	3.3047	0.057489	1.2404	0.20559	23.82736	23.7975775	23.9660675	23.792985
Q9WVA2	Mitochondrial import inner membrane translocase subunit Tim8 A (Deafness dystonia protein 1 homolog)	Timm8a1 Ddp1 Tim8a Timm8a	30058	3.2933	0.057988	1.2367	0.20689	20.1727975	19.83949	19.940565	19.8692625
P62814	V-type proton ATPase subunit B, brain isoform (V-ATPase subunit B 2) (Endomembrane proton pump 58 kDa subunit) (Vacuolar proton pump subunit B 2)	Atp6v1b2 Atp6b2 Vat2	11966	3.2846	0.058372	1.2338	0.207	26.3355675	26.233275	26.377055	26.24789
P60867	40S ribosomal protein S20	Rps20	67427	3.2875	0.058245	1.2347	0.207	21.62635	21.473695	21.4852575	21.74917
Q3UHD9	Arf-GAP with GTPase, ANK repeat and PH domain-containing protein 2 (AGAP-2) (Centaurin-gamma-1) (Cnt-g1) (Phosphatidylinositol 3-kinase enhancer) (PIKE)	Agap2 Centg1 Kiaa0167	216439	3.2834	0.058427	1.2334	0.207	20.60813	20.5986725	20.3805775	20.364175
P15508	Spectrin beta chain, erythrocytic (Beta-I spectrin)	Sptb Spnb-1 Spnb1 Sptb1		3.2521	0.059837	1.223	0.2095	19.6394625	19.507445	19.13824	19.08845
P62862	40S ribosomal protein S30	Fau		3.2404	0.060375	1.2191	0.2095	19.732045	19.920255	19.7483475	19.9420925
Q03137	Ephrin type-A receptor 4 (EC 2.7.10.1) (Tyrosine-protein kinase receptor MPK-3) (Tyrosine-protein kinase receptor SEK-1)	Epha4 Sek Sek1	13838	3.2471	0.060066	1.2214	0.2095	20.4647425	20.131535	20.3539	19.896325
P49312	Heterogeneous nuclear ribonucleoprotein A1 (hnRNP A1) (HDP-1) (Helix-destabilizing protein) (Single-strand-binding protein) (Topoisomerase-inhibitor suppressed) (hnRNP core protein A1) [Cleaved into: Heterogeneous	Hnrnpa1 Fli-2 Hnrpa1 Tis	15382	3.2375	0.06051	1.2182	0.2095	21.504785	21.717615	21.7761275	21.8365225

	nuclear ribonucleoprotein A1, N-terminally processed]										
Q9JJV2	Profilin-2 (Profilin II)	Pfn2	18645	3.2389	0.060446	1.2186	0.2095	23.8513	23.73724	23.8145625	23.68605
Q8BP92	Reticulocalbin-2 (Taipoxin-associated calcium-binding protein 49) (TCBP-49)	Rcn2	26611	3.2618	0.059397	1.2262	0.2095	20.198035	20.582305	20.57846	20.599875
Q9CZS1	Aldehyde dehydrogenase X, mitochondrial (EC 1.2.1.3) (Aldehyde dehydrogenase family 1 member B1)	Aldh1b1 Aldhx	72535	3.2546	0.059724	1.2238	0.2095	20.146925	20.1044575	20.076405	19.83778
Q922B1	ADP-ribose glycohydrolase MACROD1 (MACRO domain-containing protein 1) (O-acetyl-ADP-ribose deacetylase MACROD1) (EC 3.1.1.106) (Protein LRP16) ([Protein ADP-ribosylaspartate] hydrolase MACROD1) (EC 3.2.2.-) ([Protein ADP-ribosylglutamate] hydrolase MACROD1) (EC 3.2.2.-)	Macrod1 Lrp16	107227	3.2408	0.060357	1.2193	0.2095	19.1825325	18.2966125	18.694515	19.12961
A2RT62	F-box/LRR-repeat protein 16 (F-box and leucine-rich repeat protein 16)	Fbxl16	214931	3.2533	0.059784	1.2234	0.2095	21.2505675	21.047235	21.2198775	20.8113425
P35979	60S ribosomal protein L12	Rpl12	269261	3.2347	0.060642	1.2172	0.2095	24.1942	24.2546875	24.0007825	24.29938
P61294	Ras-related protein Rab-6B	Rab6b D9Bwg0185e	270192	3.2539	0.059755	1.2236	0.2095	21.0137475	20.799995	20.898695	20.76015
Q8VEK3	Heterogeneous nuclear ribonucleoprotein U (hnRNP U) (Scaffold-attachment factor A) (SAF-A)	Hnrnpu Hnrpu	51810	3.2269	0.061003	1.2146	0.21	23.5336975	23.5352475	23.640205	23.68345
Q5DTL9	Sodium-driven chloride bicarbonate exchanger (Solute carrier family 4 member 10)	Slc4a10 Kiaa4136 Ncbe	94229	3.2257	0.06106	1.2142	0.21	21.4222125	21.2498975	21.68194	21.359865
Q99K85	Phosphoserine aminotransferase (PSAT) (EC 2.6.1.52) (Endometrial progesterone-induced protein) (EPIP) (Phosphohydroxythreonine aminotransferase)	Psat1 Psa Psat	107272	3.2044	0.062069	1.2071	0.21299	22.7337125	22.8047675	22.7190475	23.0942725
P27612	Phospholipase A-2-activating protein (PLA2P) (PLAP)	Plaa Plap	18786	3.2004	0.06226	1.2058	0.21316	19.6443725	19.0970475	19.4027725	19.0422475
Q9CZT8	Ras-related protein Rab-3B	Rab3b	69908	3.1853	0.062986	1.2008	0.21517	21.8883325	21.7521075	21.869635	21.5246375
Q9JMA1	Ubiquitin carboxyl-terminal hydrolase 14 (EC 3.4.19.12) (Deubiquitinating enzyme 14) (Ubiquitin thioesterase 14) (Ubiquitin-specific-processing protease 14)	Usp14	59025	3.1774	0.063371	1.1981	0.216	20.420515	20.265235	20.26226	20.2605475
P62996	Transformer-2 protein homolog beta (TRA-2 beta) (TRA2-beta) (Silica-induced gene 41 protein) (SIG-41) (Splicing factor, arginine/serine-rich 10) (Transformer-2 protein homolog B)	Tra2b Sfrs10 Silg41	20462	3.172	0.063635	1.1963	0.21636	18.9206725	18.982065	19.133185	19.19968
Q9QYA2	Mitochondrial import receptor subunit TOM40 homolog (Mitochondrial outer membrane protein of 35 kDa) (MOM35) (Protein Haymaker) (Translocase of outer membrane 40 kDa subunit homolog)	Tomm40 Mom35 Tom40	53333	3.1666	0.063901	1.1945	0.21636	19.9947825	19.6514825	19.94313	19.750265
Q91WQ3	Tyrosine--tRNA ligase, cytoplasmic (EC 6.1.1.1) (Tyrosyl-tRNA synthetase) (TyrRS) [Cleaved into: Tyrosine--tRNA ligase, cytoplasmic, N-terminally processed]	Yars1 Yars	107271	3.1686	0.063802	1.1952	0.21636	20.7526075	20.87094	20.8162625	20.95065
P56395	Cytochrome b5	Cyb5a Cyb5	109672	3.1533	0.064564	1.19	0.21812	19.9414125	20.2091675	20.1120225	20.46141
P97450	ATP synthase-coupling factor 6, mitochondrial (ATPase subunit F6) (ATP synthase peripheral stalk subunit F6)	Atp5pf Atp5j	11957	3.1374	0.065363	1.1847	0.22027	23.3532575	23.1697575	23.213835	23.098345
Q9DAS9	Guanine nucleotide-binding protein G(I)/G(S)/G(O) subunit gamma-12	Gng12	14701	3.1349	0.065489	1.1838	0.22027	22.4969225	22.2685025	22.2934675	22.1707125
P23506	Protein-L-isoaspartate(D-aspartate) O-methyltransferase (PIMT) (EC 2.1.1.77) (L-isoaspartyl protein carboxyl methyltransferase)	Pcmt1		3.1212	0.066187	1.1792	0.22164	21.922545	21.83935	22.230375	21.72613

	(Protein L-isoaspartyl/D-aspartyl methyltransferase) (Protein-beta-aspartate methyltransferase)										
Q922U2	Keratin, type II cytoskeletal 5 (Cytokeratin-5) (CK-5) (Keratin-5) (K5) (Type-II keratin Kb5)	Krt5 Krt2-5	110308	3.1227	0.066111	1.1797	0.22164	19.11854	17.1535125	18.8547375	18.47551
P47911	60S ribosomal protein L6 (TAX-responsive enhancer element-binding protein 107) (TAXREB107)	Rpl6	19988	3.1153	0.066491	1.1772	0.22168	22.0814575	22.10839	22.0175225	22.3422075
Q9Z0P5	Twinfilin-2 (A6-related protein) (mA6RP) (Twinfilin-1-like protein)	Twf2 Ptk9l	23999	3.1157	0.066468	1.1774	0.22168	20.7447625	20.736975	20.75585	20.4696125
P62317	Small nuclear ribonucleoprotein Sm D2 (Sm-D2) (snRNP core protein D2)	Snrpd2	107686	3.098	0.067394	1.1714	0.22421	19.8802775	19.350135	19.75165	19.660315
Q6PHZ2	Calcium/calmodulin-dependent protein kinase type II subunit delta (CaM kinase II subunit delta) (CaMK-II subunit delta) (EC 2.7.11.17)	Camk2d Kiaa4163	108058	3.0902	0.067805	1.1687	0.22508	21.84585	22.2276175	22.4534975	22.0154575
Q9CZW5	Mitochondrial import receptor subunit TOM70 (Mitochondrial precursor proteins import receptor) (Translocase of outer membrane 70 kDa subunit) (Translocase of outer mitochondrial membrane protein 70)	Tomm70 D16Wsu109e Tomm70a	28185	3.0855	0.068051	1.1672	0.22532	23.259545	23.21372	23.3039675	23.21556
Q922S4	cGMP-dependent 3',5'-cyclic phosphodiesterase (EC 3.1.4.17) (Cyclic GMP-stimulated phosphodiesterase) (CGS-PDE) (cGSPDE)	Pde2a	207728	3.0805	0.068316	1.1655	0.22532	22.0234575	22.0408675	22.2397775	22.0387275
Q6NS60	F-box only protein 41	Fbxo41 D6Ertd538e Kiaa1940	330369	3.0805	0.068319	1.1655	0.22532	19.6895075	19.5050175	19.7571675	19.3480025
O54983	Ketimine reductase mu-crystallin (EC 1.5.1.25) (NADP-regulated thyroid-hormone-binding protein)	Crym	12971	3.0727	0.068736	1.1628	0.22621	25.3512775	25.0758025	25.0187975	24.9489925
P28271	Cytoplasmic aconitate hydratase (Aconitase) (EC 4.2.1.3) (Citrate hydro-lyase) (Iron regulatory protein 1) (IRP1) (Iron-responsive element-binding protein 1) (IRE-BP 1)	Aco1 Ireb1 Irebp	11428	3.0607	0.069382	1.1588	0.22784	19.5588575	19.3185075	19.4271575	19.3990275
O35737	Heterogeneous nuclear ribonucleoprotein H (hnRNP H) [Cleaved into: Heterogeneous nuclear ribonucleoprotein H, N-terminally processed]	Hnrnph1 Hnrph Hnrph1	59013	3.0503	0.06995	1.1552	0.22922	22.7266625	22.6934175	22.71679	22.841215
P11499	Heat shock protein HSP 90-beta (Heat shock 84 kDa) (HSP 84) (HSP84) (Tumor-specific transplantation 84 kDa antigen) (TSTA)	Hsp90ab1 Hsp84 Hsp84-1 Hspcb	15516	3.0459	0.070189	1.1537	0.2295	27.0714825	27.05025	27.19282	27.1669275
P56480	ATP synthase subunit beta, mitochondrial (EC 7.1.2.2) (ATP synthase F1 subunit beta)	Atp5f1b Atp5b	11947	3.0431	0.070342	1.1528	0.22951	28.46113	28.47266	28.5354625	28.4120125
Q61656	Probable ATP-dependent RNA helicase DDX5 (EC 3.6.4.13) (DEAD box RNA helicase DEAD1) (mDEAD1) (DEAD box protein 5) (RNA helicase p68)	Ddx5 Tnz2		3.0392	0.070556	1.1515	0.22972	22.8119	22.6462775	22.80686	22.8378025
Q64010	Adapter molecule crk (Proto-oncogene c-Crk) (p38)	Crk Crko	12928	3.0163	0.07184	1.1436	0.2334	20.9454425	20.8011875	20.937595	20.7073175
P20065	Thymosin beta-4 (T beta 4) [Cleaved into: Hematopoietic system regulatory peptide (Seraspenide)]	Tmsb4x Ptmb4 Tmsb4	19241	2.9962	0.072983	1.1368	0.23661	21.321155	20.976045	20.748745	20.340495
P09411	Phosphoglycerate kinase 1 (EC 2.7.2.3)	Pgk1 Pgk-1	18655	2.9927	0.073186	1.1356	0.23677	27.0117675	26.9091975	27.0809975	26.9612275
Q80SW1	S-adenosylhomocysteine hydrolase-like protein 1 (IP3R-binding protein released with inositol 1,4,5-trisphosphate) (Putative adenosylhomocysteinase 2) (S-adenosyl-L-homocysteine hydrolase 2) (AdoHcyase 2)	Ahcyl1 Irbit	229709	2.9861	0.073569	1.1333	0.23751	24.2092825	24.1092175	24.01089	24.15847
P80313	T-complex protein 1 subunit eta (TCP-1-eta) (CCT-eta)	Cct7 Ccth	12468	2.9788	0.073993	1.1308	0.23783	23.01322	22.9298875	23.083075	23.0802225
Q9CVB6	Actin-related protein 2/3 complex subunit 2 (Arp2/3 complex 34 kDa subunit) (p34-ARC)	Arpc2	76709	2.9737	0.074292	1.1291	0.23783	24.114525	24.0775575	24.01755	23.94274
P61089	Ubiquitin-conjugating enzyme E2 N (EC 2.3.2.23) (Bendless-like ubiquitin-conjugating	Ube2n Blu	93765	2.9769	0.074101	1.1302	0.23783	22.9638375	23.2371475	23.15518	23.2386975

	enzyme) (E2 ubiquitin-conjugating enzyme N) (Ubc13) (Ubiquitin carrier protein N) (Ubiquitin-protein ligase N)										
P31650	Sodium- and chloride-dependent GABA transporter 3 (GAT-3) (Sodium- and chloride-dependent GABA transporter 4) (GAT-4) (Solute carrier family 6 member 11)	Slc6a11 Gabt3 Gabt4 Gat-4 Gat3 Gat4	243616	2.9746	0.074237	1.1294	0.23783	23.04519	22.989765	22.8037225	22.73623
P60041	Somatostatin [Cleaved into: Antrin; Somatostatin-28; Somatostatin-14; Neuronostatin (NST)]	Sst Smst	20604	2.9671	0.074679	1.1268	0.23857	19.55083	19.28852	19.5232025	18.8711975
Q8BYM5	Neuroligin-3 (Gliotactin homolog)	Nlgn3	245537	2.9616	0.075007	1.1249	0.23911	20.6121925	20.3650225	20.4241525	20.0792525
Q00898	Alpha-1-antitrypsin 1-5 (Alpha-1 protease inhibitor 5) (Serine protease inhibitor 1-5) (Serine protease inhibitor A1e) (Serpin A1e)	Serpina1e Dom5 Spi1-5	20704	2.9522	0.075563	1.1217	0.23939	19.0319275	20.6437025	18.9577825	19.4145375
P27659	60S ribosomal protein L3 (J1 protein)	Rpl3	27367	2.9533	0.075499	1.1221	0.23939	22.07862	22.12941	22.1064175	22.3472625
Q922Q1	Mitochondrial amidoxime reducing component 2 (mARC2) (EC 1.7.-.-) (Molybdenum cofactor sulfurase C-terminal domain-containing protein 2) (MOSC domain-containing protein 2) (Moco sulfurase C-terminal domain-containing protein 2)	Mtarc2 Marc2 Mg87 Mosc2	67247	2.9523	0.07556	1.1217	0.23939	21.19425	21.2750225	21.1024775	21.33434
Q9JI91	Alpha-actinin-2 (Alpha-actinin skeletal muscle isoform 2) (F-actin cross-linking protein)	Actn2	11472	2.9474	0.075852	1.12	0.2398	19.910305	19.8503975	19.63253	19.7888625
P48962	ADP/ATP translocase 1 (ADP,ATP carrier protein 1) (ADP,ATP carrier protein, heart/skeletal muscle isoform T1) (Adenine nucleotide translocator 1) (ANT 1) (Solute carrier family 25 member 4) (mANC1)	Slc25a4 Anc1 Ant1	11739	2.9406	0.076264	1.1177	0.2398	27.1909775	27.286055	27.132685	27.243475
Q9QUR6	Prolyl endopeptidase (PE) (EC 3.4.21.26) (Post-proline cleaving enzyme)	Prep Pep	19072	2.9356	0.076567	1.116	0.2398	20.042355	19.8541375	20.176805	19.7549225
P46471	26S proteasome regulatory subunit 7 (26S proteasome AAA-ATPase subunit RPT1) (Proteasome 26S subunit ATPase 2) (Protein MSS1)	Psmc2 Mss1	19181	2.9361	0.076532	1.1162	0.2398	21.0364675	21.0764775	21.2874925	21.167815
P14115	60S ribosomal protein L27a (L29)	Rpl27a	26451	2.9438	0.076071	1.1188	0.2398	19.5919975	19.6332975	19.5606975	19.8404625
Q91W90	Thioredoxin domain-containing protein 5 (Endoplasmic reticulum resident protein 46) (ER protein 46) (ERp46) (Plasma cell-specific thioredoxin-related protein) (PC-TRP) (Thioredoxin-like protein p46)	Txndc5 Tlp46	105245	2.9344	0.076635	1.1156	0.2398	18.7339525	18.919685	19.253335	19.0997925
P63276	40S ribosomal protein S17	Rps17	20068	2.9317	0.0768	1.1146	0.23982	20.6396775	20.6835425	20.81731	20.865275
P97315	Cysteine and glycine-rich protein 1 (Cysteine-rich protein 1) (CRP) (CRP1)	Csrp1 Crp1 Csrp	13007	2.9214	0.077434	1.1111	0.24131	23.2805325	23.3889675	23.2634475	23.616105
Q99JY0	Trifunctional enzyme subunit beta, mitochondrial (TP-beta) [Includes: 3-ketoacyl-CoA thiolase (EC 2.3.1.155) (EC 2.3.1.16) (Acetyl-CoA acyltransferase) (Beta-ketothiolase)]	Hadhb	231086	2.9184	0.077618	1.11	0.24139	22.1383925	22.16429	22.02758	22.38348
Q9CWJ9	Bifunctional purine biosynthesis protein PURH [Includes: Phosphoribosylaminoimidazolecarboxamide formyltransferase (EC 2.1.2.3) (5-aminoimidazole-4-carboxamide ribonucleotide formyltransferase) (AICAR transformylase); IMP cyclohydrolase (EC 3.5.4.10) (ATIC) (IMP synthase) (Inosinicase)]	Atic Purh	108147	2.9126	0.077975	1.108	0.24201	20.07337	20.3607425	20.4491025	20.51156
Q9JLJ2	4-trimethylaminobutyraldehyde dehydrogenase (TMABA-DH) (TMABADH) (EC 1.2.1.47) (Aldehyde dehydrogenase family 9 member A1) (EC 1.2.1.3)	Aldh9a1	56752	2.9077	0.07828	1.1063	0.24246	19.46713	18.59263	18.7754825	18.1138125

P62774	Myotrophin (Granule cell differentiation protein) (Protein V-1)	Mtpn Gcdp	14489	2.9045	0.07848	1.1052	0.24259	22.37948	22.28751	22.2619125	22.16054
P50580	Proliferation-associated protein 2G4 (IRES-specific cellular trans-acting factor 45 kDa) (ITAF45) (Mpp1) (Proliferation-associated protein 1) (Protein p38-2G4)	Pa2g4 Ebp1 Plfap	18813	2.8772	0.080211	1.0958	0.24644	21.0592125	21.1332725	21.00816	21.25133
P54728	UV excision repair protein RAD23 homolog B (HR23B) (mHR23B) (XP-C repair-complementing complex 58 kDa protein) (p58)	Rad23b Mhr23b	19359	2.8812	0.079954	1.0972	0.24644	20.3780125	20.143555	20.3461275	20.13644
Q5PR73	GTP-binding protein Di-Ras2 (Distinct subgroup of the Ras family member 2)	Diras2	68203	2.8784	0.080132	1.0962	0.24644	22.5488225	22.378235	22.4832175	22.324555
Q9Z268	RasGAP-activating-like protein 1 (RAS protein activator like 1) (Ras GTPase-activating-like protein)	Rasal1 Rasal	19415	2.8716	0.080569	1.0938	0.24655	21.22547	21.314255	21.2715025	20.964415
Q8BGN8	Synaptoporin	Synpr	72003	2.872	0.080541	1.094	0.24655	20.89616	20.72657	20.56315	20.4783325
P11404	Fatty acid-binding protein, heart (Fatty acid-binding protein 3) (Heart-type fatty acid-binding protein) (H-FABP) (Mammary-derived growth inhibitor) (MDGI)	Fabp3 Fabph1	14077	2.8664	0.080903	1.092	0.24678	22.50638	22.3246025	22.2819875	22.13612
Q80TZ3	Putative tyrosine-protein phosphatase auxilin (EC 3.1.3.48) (DnaJ homolog subfamily C member 6)	Dnajc6 Kiaa0473	72685	2.8654	0.080967	1.0917	0.24678	23.1247625	23.0656425	23.17001	23.05213
Q9R1V7	Disintegrin and metalloproteinase domain-containing protein 23 (ADAM 23) (Metalloproteinase-like, disintegrin-like, and cysteine-rich protein 3) (MDC-3)	Adam23 Mdc3	23792	2.8594	0.081355	1.0896	0.24747	20.91007	20.88309	20.894895	21.2327225
Q9DB77	Cytochrome b-c1 complex subunit 2, mitochondrial (Complex III subunit 2) (Core protein II) (Ubiquinol-cytochrome-c reductase complex core protein 2)	Uqcrc2	67003	2.8553	0.081624	1.0882	0.24779	25.8086175	25.77417	25.7073325	25.78721
Q07076	Annexin A7 (Annexin VII) (Annexin-7) (Synexin)	Anxa7 Anx7	11750	2.85	0.081973	1.0863	0.24787	20.2557825	20.1053825	20.052185	19.8105125
Q6P9K8	Caskin-1 (CASK-interacting protein 1)	Caskin1 Kiaa1306	268932	2.8512	0.081895	1.0867	0.24787	21.042025	21.1072	21.103555	20.72561
Q9DB05	Alpha-soluble NSF attachment protein (SNAP-alpha) (N-ethylmaleimide-sensitive factor attachment protein alpha)	Napa Snapa	108124	2.8443	0.082348	1.0843	0.24851	22.8455175	22.7499125	22.779885	22.669305
P14131	40S ribosomal protein S16	Rps16	20055	2.8412	0.082554	1.0833	0.24864	22.3681825	22.387425	22.39052	22.5597625
Q60714				2.8356	0.082928	1.0813	0.24927	17.481395	18.07458	17.40785	18.336305
P39688	Tyrosine-protein kinase Fyn (EC 2.7.10.2) (Proto-oncogene c-Fyn) (p59-Fyn)	Fyn	14360	2.8137	0.084397	1.0737	0.25024	20.2769925	20.181505	20.026855	20.1703575
Q9Z0X1	Apoptosis-inducing factor 1, mitochondrial (EC 1.6.99.-) (Programmed cell death protein 8)	Aifm1 Aif Pdcd8	26926	2.815	0.08431	1.0741	0.25024	21.100935	20.878795	20.960805	20.95017
P70441	Na(+)/H(+) exchange regulatory cofactor NHE-RF1 (NHERF-1) (Ezrin-radixin-moesin-binding phosphoprotein 50) (EBP50) (Regulatory cofactor of Na(+)/H(+) exchanger) (Sodium-hydrogen exchanger regulatory factor 1) (Solute carrier family 9 isoform A3 regulatory factor 1)	Slc9a3r1 Nherf Nherf1	26941	2.8259	0.083574	1.0779	0.25024	22.1116025	22.1100875	21.8597625	22.1764875
Q9CWS0	N(G),N(G)-dimethylarginine dimethylaminohydrolase 1 (DDAH-1) (Dimethylarginine dimethylaminohydrolase 1) (EC 3.5.3.18) (DDAHI) (Dimethylargininase-1)	Ddah1	69219	2.8144	0.084349	1.0739	0.25024	22.8925725	22.9787975	22.993535	23.1820425
Q80VP0	Tectonin beta-propeller repeat-containing protein 1	Tecpr1 Kiaa1358	70381	2.8163	0.084223	1.0746	0.25024	19.463855	19.54068	19.337865	19.59611
Q8BMS1	Trifunctional enzyme subunit alpha, mitochondrial (Monolysocardiolipin acyltransferase) (EC 2.3.1.-) (TP-alpha) [Includes: Long-chain enoyl-CoA hydratase (EC	Hadha	97212	2.8198	0.083984	1.0758	0.25024	23.22733	23.3615225	23.24398	23.5131425

	4.2.1.17); Long chain 3-hydroxyacyl-CoA dehydrogenase (EC 1.1.1.211)]										
P10853	Histone H2B type 1-F/J/L (H2B 291A)	H2bc7 H2b-f Hist1h2bf; H2bc11 H2b-j Hist1h2bj; H2bc13 H2b-l Hist1h2bl; H2bc15 H2b-n Hist1h2bn	319180 319183 319185 319187 665596 665622	2.816	0.084245	1.0745	0.25024	24.0500075	24.21729	24.0728575	24.370145
P61079	Ubiquitin-conjugating enzyme E2 D3 (EC 2.3.2.23) ((E3-independent) E2 ubiquitin-conjugating enzyme D3) (EC 2.3.2.24) (E2 ubiquitin-conjugating enzyme D3) (Ubiquitin carrier protein D3) (Ubiquitin-conjugating enzyme E2(17)KB 3) (Ubiquitin-conjugating enzyme E2-17 kDa 3) (Ubiquitin-protein ligase D3)	Ube2d3	66105	2.8003	0.085314	1.069	0.25247	21.1046325	21.1791275	21.063345	21.18405
Q9JJU8	SH3 domain-binding glutamic acid-rich-like protein	Sh3bgrl	56726	2.7947	0.085703	1.067	0.25313	20.3881325	20.29911	20.090335	20.2303025
Q02053	Ubiquitin-like modifier-activating enzyme 1 (EC 6.2.1.45) (Ubiquitin-activating enzyme E1) (Ubiquitin-activating enzyme E1 X) (Ubiquitin-like modifier-activating enzyme 1 X)	Uba1 Sbx Ube1 Ube1ax Ube1x	22201	2.7867	0.086255	1.0642	0.25354	25.492885	25.5180125	25.4904025	25.5767675
Q9Z0E0	Neurochondrin (M-Sema F-associating protein of 75 kDa) (Norbin)	Ncdn Kiaa0607 Sfap75	26562	2.789	0.086097	1.065	0.25354	25.4381725	25.5320975	25.5012575	25.330535
Q9DCT8	Cysteine-rich protein 2 (CRP-2) (Heart LIM protein)	Crip2 Crp2 Hlp	68337	2.7849	0.086384	1.0636	0.25354	20.3037075	20.5206625	20.687165	20.68752
Q8VCM7	Fibrinogen gamma chain	Fgg	99571	2.7831	0.086506	1.063	0.25354	23.5466275	23.96044	23.3788125	23.4399225
Q5FWK3	Rho GTPase-activating protein 1 (Rho-type GTPase-activating protein 1)	Arhgap1	228359	2.779	0.08679	1.0615	0.25389	21.198475	21.316425	21.6087425	21.55496
P16546	Spectrin alpha chain, non-erythrocytic 1 (Alpha-II spectrin) (Fodrin alpha chain)	Sptan1 Spna2 Spta2		2.7758	0.087016	1.0604	0.254	28.3716425	28.34653	28.37566	28.2578275
Q62108	Disks large homolog 4 (Postsynaptic density protein 95) (PSD-95) (Synapse-associated protein 90) (SAP-90) (SAP90)	Dlg4 Dlgh4 Psd95	13385	2.7721	0.087278	1.0591	0.254	23.795675	23.84517	23.686035	23.5282525
P22723	Gamma-aminobutyric acid receptor subunit gamma-2 (GABA(A) receptor subunit gamma-2)	Gabrg2	14406	2.7691	0.087493	1.058	0.254	19.9225875	19.958555	20.148755	19.7881875
Q9QZX7	Serine racemase (EC 5.1.1.18) (D-serine ammonia-lyase) (D-serine dehydratase) (EC 4.3.1.18) (L-serine ammonia-lyase) (L-serine dehydratase) (EC 4.3.1.17)	Srr	27364	2.7704	0.087399	1.0585	0.254	21.7476525	21.89799	21.8426625	21.675425
P14869	60S acidic ribosomal protein P0 (60S ribosomal protein L10E)	Rplp0 Arbp	11837	2.7657	0.087734	1.0568	0.25421	22.2006275	22.30164	22.308695	22.39508
P56376	Acylphosphatase-1 (EC 3.6.1.7) (Acylphosphatase, organ-common type isozyme) (Acylphosphate phosphohydrolase 1)	Acyp1 Acype	66204	2.7598	0.08815	1.0548	0.25445	21.1609975	20.9972925	21.060525	21.028905
Q6Q477	Plasma membrane calcium-transporting ATPase 4 (PMCA4) (EC 7.2.2.10)	Atp2b4	381290	2.7609	0.088073	1.0552	0.25445	21.7767925	22.37397	22.423705	22.1974
O08599	Syntaxin-binding protein 1 (Protein unc-18 homolog 1) (Unc18-1) (Protein unc-18 homolog A) (Unc-18A)	Stxbp1	20910	2.7484	0.088969	1.0508	0.25633	27.3239075	27.3799075	27.561885	27.3374925
P01942	Hemoglobin subunit alpha (Alpha-globin) (Hemoglobin alpha chain)	Hba Hba-a1		2.7317	0.090188	1.0449	0.25664	26.9849575	26.4090325	26.728875	26.9796375
P16014	Secretogranin-1 (Chromogranin-B) (CgB) (Secretogranin I) (SgI) [Cleaved into: CCB peptide; PE-11]	Chgb Scg-1 Scg1	12653	2.7315	0.090202	1.0448	0.25664	19.86831	19.7689075	20.086	20.0052975
P34884	Macrophage migration inhibitory factor (MIF) (EC 5.3.2.1) (Delayed early response protein 6) (DER6) (Glycosylation-inhibiting factor) (GIF) (L-dopachrome isomerase) (L-dopachrome tautomerase) (EC 5.3.3.12) (Phenylpyruvate tautomerase)	Mif	17319	2.7334	0.090059	1.0455	0.25664	24.3967175	24.22785	24.4059625	24.31862

Q9QYR6	Microtubule-associated protein 1A (MAP-1A) [Cleaved into: MAP1A heavy chain; MAP1 light chain LC2]	Map1a Mtap1 Mtap1a	17754	2.7348	0.089958	1.046	0.25664	26.001965	25.998505	25.930255	25.84976
Q60771	Claudin-11 (Oligodendrocyte transmembrane protein) (Oligodendrocyte-specific protein)	Cldn11 Osp Otm	18417	2.7424	0.089403	1.0486	0.25664	23.9269025	23.85525	23.51281	24.119235
P47708	Rabphilin-3A (Exophilin-1)	Rph3a	19894	2.7308	0.090252	1.0445	0.25664	22.3418675	22.25182	22.39447	21.9778975
Q91V12	Cytosolic acyl coenzyme A thioester hydrolase (EC 3.1.2.2) (Acyl-CoA thioesterase 7) (Brain acyl-CoA hydrolase) (BACH) (CTE-IIa) (CTE-II) (Long chain acyl-CoA thioester hydrolase)	Acot7 Bach	70025	2.7414	0.08948	1.0483	0.25664	25.16185	25.0944875	25.2099275	25.1873875
P62715	Serine/threonine-protein phosphatase 2A catalytic subunit beta isoform (PP2A-beta) (EC 3.1.3.16)	Ppp2cb	19053	2.7255	0.090641	1.0427	0.25727	23.7261075	23.57846	23.7906875	23.595475
Q9CYZ2	Tumor protein D54 (Tumor protein D52-like 2)	Tpd52l2	66314	2.7078	0.091953	1.0364	0.26045	20.20917	19.82658	20.2250175	19.99712
Q9CR16	Peptidyl-prolyl cis-trans isomerase D (PPIase D) (EC 5.2.1.8) (40 kDa peptidyl-prolyl cis-trans isomerase) (Cyclophilin-40) (CYP-40) (Rotamase D)	Ppid	67738	2.7058	0.092103	1.0357	0.26045	22.3809925	22.3128775	22.4365175	22.2965425
Q9R0N7	Synaptotagmin-7 (Synaptotagmin VII) (SytVII)	Syt7	54525	2.7027	0.092341	1.0346	0.26064	19.8690975	19.7061875	19.697015	19.41235
Q9D1G1	Ras-related protein Rab-1B	Rab1b	76308	2.6983	0.09267	1.0331	0.26108	21.65644	21.545275	21.8201125	21.49254
Q64332	Synapsin-2 (Synapsin II)	Syn2	20965	2.691	0.093227	1.0305	0.26217	26.261625	26.265705	26.29939	26.0664925
Q9R0Q6	Actin-related protein 2/3 complex subunit 1A (SOP2-like protein) (Sid 329)	Arpc1a Sid329	56443	2.6802	0.094048	1.0267	0.26399	24.2869175	24.20099	24.1997375	24.0572025
Q8BLK3	Limbic system-associated membrane protein (LSAMP)	Lsamp		2.6736	0.094563	1.0243	0.26416	24.7577075	24.66237	24.537565	24.5437325
Q99KB8	Hydroxyacylglutathione hydrolase, mitochondrial (EC 3.1.2.6) (Glyoxalase II) (Glx II)	Hagh Glo2	14651	2.6728	0.094625	1.024	0.26416	21.678445	21.4061225	21.543645	21.51535
P97441	Zinc transporter 3 (ZnT-3) (Solute carrier family 30 member 3)	Slc30a3 Znt3	22784	2.675	0.094453	1.0248	0.26416	19.5640675	19.68675	19.75219	19.3099025
O70310	Glycylpeptide N-tetradecanoyltransferase 1 (EC 2.3.1.97) (Myristoyl-CoA:protein N-myristoyltransferase 1) (NMT 1) (Type I N-myristoyltransferase) (Peptide N-myristoyltransferase 1)	Nmt1	18107	2.6668	0.095092	1.0219	0.26452	19.6061825	20.0368575	20.031165	20.029695
Q80ZJ1	Ras-related protein Rap-2a	Rap2a	76108	2.6666	0.095101	1.0218	0.26452	18.6266625	17.0856075	18.4515675	17.833025
P67871	Casein kinase II subunit beta (CK II beta) (Phosvitin)	Csnk2b Ck2n	13001	2.6609	0.095551	1.0198	0.26528	20.5951425	20.7324225	20.8089225	20.7896475
P48320	Glutamate decarboxylase 2 (EC 4.1.1.15) (65 kDa glutamic acid decarboxylase) (GAD-65) (Glutamate decarboxylase 65 kDa isoform)	Gad2 Gad65	14417	2.6462	0.096711	1.0145	0.26802	21.5833375	21.335825	21.6132775	21.252225
Q8BNW9	Kelch repeat and BTB domain-containing protein 11	Kbtbd11 Kiaa0711	74901	2.6379	0.097368	1.0116	0.26906	20.2310175	20.013825	19.9270375	19.88145
Q9DCN2	NADH-cytochrome b5 reductase 3 (B5R) (Cytochrome b5 reductase) (EC 1.6.2.2) (Diaphorase-1) [Cleaved into: NADH-cytochrome b5 reductase 3 membrane-bound form; NADH-cytochrome b5 reductase 3 soluble form]	Cyb5r3 Dia1	109754	2.637	0.097441	1.0113	0.26906	22.1941675	22.146665	22.2818875	22.524015
Q99LX0	Protein/nucleic acid deglycase DJ-1 (EC 3.1.2.-) (EC 3.5.1.-) (EC 3.5.1.124) (Maillard deglycase) (Parkinson disease protein 7 homolog) (Parkinsonism-associated deglycase) (Protein DJ-1) (DJ-1)	Park7	57320	2.631	0.097925	1.0091	0.26991	23.6112625	23.4079675	23.5924725	23.45832
P50518	V-type proton ATPase subunit E 1 (V-ATPase subunit E 1) (V-ATPase 31 kDa subunit) (p31) (Vacuolar proton pump subunit E 1)	Atp6v1e1 Atp6e Atp6e2	11973	2.6243	0.098462	1.0067	0.2709	24.7513025	24.6788125	24.815795	24.463865

Q9DCW4	Electron transfer flavoprotein subunit beta (Beta-ETF)	Etfb	110826	2.6195	0.098854	1.005	0.27149	22.48766	22.4588525	22.296635	22.505805
P51863	V-type proton ATPase subunit d 1 (V-ATPase subunit d 1) (P39) (Physophilin) (V-ATPase 40 kDa accessory protein) (V-ATPase AC39 subunit) (Vacuolar proton pump subunit d 1)	Atp6v0d1 Atp6d	11972	2.6094	0.099683	1.0014	0.27189	24.8605025	24.943805	24.9798875	24.8470025
P18760	Cofilin-1 (Cofilin, non-muscle isoform)	Cfl1	12631	2.593	0.10104	0.9955	0.27189	26.1293375	26.1696775	26.194215	26.2376025
P06880	Somatotropin (Growth hormone)	Gh1 Gh	14599	2.6035	0.10017	0.99926	0.27189	22.0636575	21.8499975	22.02444	21.81627
P08752	Guanine nucleotide-binding protein G(i) subunit alpha-2 (Adenylate cyclase-inhibiting G alpha protein)	Gnai2 Gnai-2	14678	2.5977	0.10065	0.99721	0.27189	23.586825	23.64845	23.547765	23.7130475
Q61548	Clathrin coat assembly protein AP180 (91 kDa synaptosomal-associated protein) (Clathrin coat-associated protein AP180) (Phosphoprotein F1-20)	Snap91	20616	2.5897	0.10131	0.99433	0.27189	25.253145	25.2579625	25.4304175	25.26402
Q63844	Mitogen-activated protein kinase 3 (MAP kinase 3) (MAPK 3) (EC 2.7.11.24) (ERT2) (Extracellular signal-regulated kinase 1) (ERK-1) (Insulin-stimulated MAP2 kinase) (MAP kinase isoform p44) (p44-MAPK) (MNK1) (Microtubule-associated protein 2 kinase) (p44-ERK1)	Mapk3 Erk1 Prkm3	26417	2.591	0.1012	0.99481	0.27189	19.499605	19.7639075	19.71235	19.50134
Q61151	Serine/threonine-protein phosphatase 2A 56 kDa regulatory subunit epsilon isoform (PP2A B subunit isoform B'-epsilon) (PP2A B subunit isoform B56-epsilon) (PP2A B subunit isoform PR61-epsilon) (PP2A B subunit isoform R5-epsilon)	Ppp2r5e Kiaa4006	26932	2.6057	0.099989	1	0.27189	20.3289725	20.517265	20.5158675	19.923175
Q9CX56	26S proteasome non-ATPase regulatory subunit 8 (26S proteasome regulatory subunit RPN12) (26S proteasome regulatory subunit S14)	Psmd8	57296	2.6034	0.10017	0.99924	0.27189	19.0540575	19.3895275	19.1947775	19.2376225
Q9D1P4	Cysteine and histidine-rich domain-containing protein 1 (CHORD domain-containing protein 1) (CHORD-containing protein 1) (Chp-1) (Protein morgana)	Chordc1 Chp1 Morgana	66917	2.6066	0.09991	1.0004	0.27189	19.707985	19.61735	19.9189425	19.7097325
Q8K0U4	Heat shock 70 kDa protein 12A	Hspa12a Kiaa0417	73442	2.5904	0.10126	0.99457	0.27189	24.89296	24.8792975	24.837095	24.785195
Q01853	Transitional endoplasmic reticulum ATPase (TER ATPase) (EC 3.6.4.6) (15S Mg(2+)-ATPase p97 subunit) (Valosin-containing protein) (VCP)	Vcp	269523	2.6002	0.10044	0.9981	0.27189	25.324255	25.35806	25.40538	25.466705
Q60902	Epidermal growth factor receptor substrate 15-like 1 (Epidermal growth factor receptor pathway substrate 15-related sequence) (Eps15-rs) (Eps15-related protein) (Eps15R)	Eps15l1 Eps15-rs Eps15R	13859	2.6041	0.10012	0.99948	0.27189	19.8535425	19.74855	19.85531	19.529
Q8VD04	GRIP1-associated protein 1 (GRASP-1) (HCMV-interacting protein) [Cleaved into: GRASP-1 C-terminal chain (30kDa C-terminus form)]	Gripap1 DXImx47e Kiaa1167	54645	2.5988	0.10056	0.99758	0.27189	18.801905	18.63368	18.8068825	18.172865
Q925I1	ATPase family AAA domain-containing protein 3 (AAA-ATPase TOB3)	Atad3 Atad3a Kiaa1273	108888	2.5648	0.10343	0.98536	0.27708	20.8626475	20.836275	20.881015	20.6435625
P23953	Carboxylesterase 1C (EC 3.1.1.1) (Liver carboxylesterase N) (Lung surfactant convertase) (PES-N)	Ces1c Es1	13884	2.5483	0.10485	0.97943	0.2804	17.724455	18.7030575	17.496805	18.4465075
P56564	Excitatory amino acid transporter 1 (Glial high affinity glutamate transporter) (High-affinity neuronal glutamate transporter) (GluT-1) (Sodium-dependent glutamate/aspartate transporter 1) (GLAST-1) (Solute carrier family 1 member 3)	Slc1a3 Eaat1 Gmt1	20512	2.5333	0.10617	0.974	0.28159	24.8751925	25.1461575	24.8792125	25.095035
P62281	40S ribosomal protein S11	Rps11	27207	2.5387	0.10569	0.97598	0.28159	21.779895	21.7283	21.6683275	21.8842575

Q9CZB0	Succinate dehydrogenase cytochrome b560 subunit, mitochondrial (Integral membrane protein CII-3) (QPs-1) (QPs1)	Sdhc	66052	2.5327	0.10622	0.9738	0.28159	20.5111125	20.7822975	20.64532	20.7455175
Q9CZ30	Obg-like ATPase 1 (GTP-binding protein 9)	Ola1 Gtpbp9	67059	2.5363	0.10591	0.97508	0.28159	22.032975	21.80158	21.984265	21.80589
Q99LG2	Transportin-2 (Karyopherin beta-2b)	Tnpo2	212999	2.5339	0.10611	0.97423	0.28159	19.49604	19.4223325	19.665665	19.6122825
P15116	Cadherin-2 (Neural cadherin) (N-cadherin) (CD antigen CD325)	Cdh2	12558	2.5268	0.10674	0.97166	0.28249	21.4047925	21.4526075	21.611865	21.4020175
Q4ACU6	SH3 and multiple ankyrin repeat domains protein 3 (Shank3) (Proline-rich synapse-associated protein 2) (ProSAP2) (SPANK-2)	Shank3 Kiaa1650 Prosap2	58234	2.5238	0.10701	0.9706	0.28269	19.972425	19.8473675	19.67464	19.4587575
Q9CQS8	Protein transport protein Sec61 subunit beta	Sec61b	66212	2.5142	0.10787	0.96712	0.28447	22.1925425	21.864355	21.9117425	21.4277125
P12658	Calbindin (Calbindin D28) (D-28K) (PCD-29) (Spot 35 protein) (Vitamin D-dependent calcium-binding protein, avian-type)	Calb1	12307	2.5025	0.10893	0.96287	0.28628	22.0755875	21.98288	21.8177725	21.55763
D3YVF0	A-kinase anchor protein 5 (AKAP-5) (A-kinase anchor protein 150 kDa) (AKAP 150) (P150) (cAMP-dependent protein kinase regulatory subunit II high affinity-binding protein)	Akap5 Akap150	238276	2.5031	0.10888	0.96307	0.28628	22.088085	22.0547925	22.100305	21.8199625
Q61207	Prosaposin (Sulfated glycoprotein 1) (SGP-1) [Cleaved into: Saposin-A; Saposin-B-Val; Saposin-B; Saposin-C; Saposin-D]	Psap Sgp1	19156	2.4987	0.10928	0.96147	0.28671	22.02225	22.67099	22.2164125	22.601985
O70133	ATP-dependent RNA helicase A (EC 3.6.4.13) (DEAH box protein 9) (mHEL-5) (Nuclear DNA helicase II) (NDH II) (RNA helicase A) (RHA)	Dhx9 Ddx9	13211	2.4915	0.10993	0.95888	0.28793	21.42117	21.35091	21.55604	21.440435
Q812A2	SLIT-ROBO Rho GTPase-activating protein 3 (srGAP3) (Rho GTPase-activating protein 14) (WAVE-associated Rac GTPase-activating protein) (WRP)	Srgap3 Arhgap14 Kiaa0411 Srgap2	259302	2.4847	0.11056	0.95639	0.28909	20.88799	20.685265	20.701445	20.6751975
Q8CDN6	Thioredoxin-like protein 1 (32 kDa thioredoxin-related protein)	Txnl1 Trp32 Txnl	53382	2.4783	0.11115	0.95409	0.29013	21.630335	21.495145	21.5365925	21.561755
P35585	AP-1 complex subunit mu-1 (AP-mu chain family member mu1A) (Adaptor protein complex AP-1 subunit mu-1) (Adaptor-related protein complex 1 subunit mu-1) (Clathrin assembly protein complex 1 mu-1 medium chain 1) (Clathrin coat assembly protein AP47) (Clathrin coat-associated protein AP47) (Golgi adaptor HA1/AP1 adaptin mu-1 subunit) (Mu-adaptin 1) (Mu1A-adaptin)	Ap1m1 Cltnm	11767	2.4737	0.11159	0.95239	0.29028	19.6647325	19.299035	19.72362	18.8358525
Q7TME0	Phospholipid phosphatase-related protein type 4 (EC 3.1.3.4) (Brain-specific phosphatidic acid phosphatase-like protein 1) (Lipid phosphate phosphatase-related protein type 4) (Plasticity-related gene 1 protein) (PRG-1)	Plppr4 D3Bwg0562e Kiaa0455 Lppr4 Php1 Prg1	229791	2.4741	0.11155	0.95254	0.29028	20.338405	20.198605	20.069515	19.8944975
Q62277	Synaptophysin (BM89 antigen) (Major synaptic vesicle protein p38)	Syp	20977	2.467	0.11222	0.94995	0.29142	25.593415	25.3657875	25.3367125	25.2928175
O35098	Dihydropyrimidinase-related protein 4 (DRP-4) (Collapsin response mediator protein 3) (CRMP-3) (UNC33-like phosphoprotein 4) (ULIP-4)	Dpysl4 Crmp3 Ulip4		2.4583	0.11303	0.94679	0.29305	23.318325	23.3031225	23.4219125	23.1401325
O88569	Heterogeneous nuclear ribonucleoproteins A2/B1 (hnRNP A2/B1)	Hnrnpa2b1 Hnrpa2b1	53379	2.4444	0.11436	0.94173	0.29598	25.01149	25.0885075	25.0522225	25.1601925
Q9D051	Pyruvate dehydrogenase E1 component subunit beta, mitochondrial (PDHE1-B) (EC 1.2.4.1)	Pdhb	68263	2.4396	0.11483	0.93996	0.29668	25.2553525	25.1412075	25.2661425	25.1503775
P37040	NADPH--cytochrome P450 reductase (CPR) (P450R) (EC 1.6.2.4)	Por	18984	2.4362	0.11515	0.93873	0.2968	19.655165	19.576305	19.802405	19.77361
Q8BL65	Actin-binding LIM protein 2 (abLIM-2) (Actin-binding LIM protein family member 2)	Ablim2	231148	2.4351	0.11526	0.93832	0.2968	19.77062	19.652325	19.7123025	19.5203525
Q3UNH4	G protein-regulated inducer of neurite outgrowth 1 (GRIN1)	Gprin1	26913	2.4264	0.1161	0.93515	0.29844	22.740335	22.6526625	22.5987175	22.3905475

Q9QXY6	EH domain-containing protein 3	Ehd3 Ehd2	57440	2.4236	0.11638	0.93412	0.29844	22.3415975	22.164895	22.6190175	22.31251
Q8R0Y6	Cytosolic 10-formyltetrahydrofolate dehydrogenase (10-FTHFDH) (FDH) (EC 1.5.1.6) (Aldehyde dehydrogenase family 1 member L1)	Aldh1l1 Fthfd	107747	2.4226	0.11648	0.93374	0.29844	25.0010625	24.8953575	24.9848125	25.1718275
Q7TQF7	Amphiphysin	Amph Amph1	218038	2.4167	0.11706	0.93158	0.29942	24.4432	24.5132475	24.5179	24.3965625
P42932	T-complex protein 1 subunit theta (TCP-1-theta) (CCT-theta)	Cct8 Cctq	12469	2.4073	0.11799	0.92815	0.30111	23.5849125	23.526645	23.51599	23.6705275
P62141	Serine/threonine-protein phosphatase PP1-beta catalytic subunit (PP-1B) (EC 3.1.3.16) (EC 3.1.3.53)	Ppp1cb	19046	2.4061	0.11812	0.92769	0.30111	23.38267	23.69027	23.627825	23.7219425
P62245	40S ribosomal protein S15a	Rps15a	267019	2.3994	0.11878	0.92524	0.30231	21.1360775	20.9395425	21.0712575	21.0469825
Q9D819	Inorganic pyrophosphatase (EC 3.6.1.1) (Pyrophosphate phospho-hydrolase) (PPase)	Ppa1 Pp Pyp	67895	2.3943	0.1193	0.92336	0.30311	21.4941275	21.6500575	21.720225	21.6194125
Q9WU78	Programmed cell death 6-interacting protein (ALG-2-interacting protein 1) (ALG-2-interacting protein X) (E2F1-inducible protein) (Eig2)	Pdcd6ip Aip1 Alix	18571	2.3866	0.12008	0.92054	0.30458	19.096945	19.47218	19.4338	19.2313825
Q56A07	Sodium channel subunit beta-2	Scn2b Gm183	72821	2.3813	0.12061	0.9186	0.30544	20.93961	20.7865175	20.78743	20.6990925
Q00612	Glucose-6-phosphate 1-dehydrogenase X (G6PD) (EC 1.1.1.49)	G6pdx G6pd G6pd-1	14381	2.3736	0.12141	0.91576	0.30693	19.2350825	18.394835	19.102335	19.31215
Q99PT1	Rho GDP-dissociation inhibitor 1 (Rho GDI 1) (GDI-1) (Rho-GDI alpha)	Arhgdia C87222 Gdi1	192662	2.3602	0.1228	0.91081	0.30994	24.3947225	24.4123275	24.46341	24.4594875
Q9CR57	60S ribosomal protein L14	Rpl14	67115	2.3512	0.12374	0.90748	0.31181	21.3395975	21.3553725	21.27319	21.49153
O55023	Inositol monophosphatase 1 (IMP 1) (IMPase 1) (EC 3.1.3.25) (D-galactose 1-phosphate phosphatase) (EC 3.1.3.94) (Inositol-1(or 4)-monophosphatase 1) (Lithium-sensitive myo-inositol monophosphatase A1)	Impa1		2.3421	0.1247	0.90412	0.31371	22.5081675	22.316255	22.29202	22.241995
P60904	DnaJ homolog subfamily C member 5 (Cysteine string protein) (CSP)	Dnajc5	13002	2.3392	0.12501	0.90305	0.31397	23.023615	23.161655	23.131935	23.04478
P62911	60S ribosomal protein L32	Rpl32	19951	2.3323	0.12574	0.90053	0.31528	20.1927	20.42766	20.2670375	20.6557925
Q9CR95	Adaptin ear-binding coat-associated protein 1 (NECAP endocytosis-associated protein 1) (NECAP-1)	Necap1	67602	2.3291	0.12609	0.89932	0.31564	22.4988725	22.5173125	22.518335	22.39385
Q501J6	Probable ATP-dependent RNA helicase DDX17 (EC 3.6.4.13) (DEAD box protein 17)	Ddx17	67040	2.3269	0.12633	0.89851	0.31571	19.2131675	18.6960125	19.41424	18.9161925
P17427	AP-2 complex subunit alpha-2 (100 kDa coated vesicle protein C) (Adaptor protein complex AP-2 subunit alpha-2) (Adaptor-related protein complex 2 subunit alpha-2) (Alpha-adaptin C) (Alpha2-adaptin) (Clathrin assembly protein complex 2 alpha-C large chain) (Plasma membrane adaptor HA2/AP2 adaptin alpha C subunit)	Ap2a2 Adtab	11772	2.3163	0.12747	0.89458	0.31613	25.8065725	25.768395	25.792375	25.6989325
Q9QUP5	Hyaluronan and proteoglycan link protein 1 (Cartilage-linking protein 1) (Cartilage-link protein) (Proteoglycan link protein)	Hapln1 Crtl1	12950	2.3167	0.12743	0.89472	0.31613	22.4792	22.58922	22.3618625	22.5617375
P68037	Ubiquitin-conjugating enzyme E2 L3 (EC 2.3.2.23) (E2 ubiquitin-conjugating enzyme L3) (UbcM4) (Ubiquitin carrier protein L3) (Ubiquitin-protein ligase L3)	Ube2l3 Ubce7	22195	2.3185	0.12723	0.8954	0.31613	22.09091	22.2623125	22.3255575	22.392105
P99028	Cytochrome b-c1 complex subunit 6, mitochondrial (Complex III subunit 6) (Complex III subunit VIII) (Cytochrome c1 non-heme 11 kDa protein) (Mitochondrial hinge protein) (Ubiquinol-cytochrome c reductase complex 11 kDa protein)	Uqcrh	66576	2.3193	0.12715	0.89569	0.31613	23.302815	23.41494	23.56353	23.348245

P35802	Neuronal membrane glycoprotein M6-a (M6a)	Gpm6a M6a	234267	2.3158	0.12753	0.89439	0.31613	25.6408975	25.6618475	25.4774225	25.4139575
Q9QXL2	Kinesin-like protein KIF21A	Kif21a Kiaa1708	16564	2.312	0.12794	0.89299	0.31664	20.8484325	20.739815	20.932985	20.7690175
Q64674	Spermidine synthase (SPDSY) (EC 2.5.1.16) (Putrescine aminopropyltransferase)	Srm	20810	2.3033	0.1289	0.88976	0.31849	20.069475	19.945485	20.2944125	20.4626
Q61361	Brevican core protein	Bcan	12032	2.2995	0.12932	0.88834	0.3185	24.5733275	24.5709525	24.44915	24.4645575
Q61879	Myosin-10 (Cellular myosin heavy chain, type B) (Myosin heavy chain 10) (Myosin heavy chain, non-muscle IIb) (Non-muscle myosin heavy chain B) (NMMHC-B) (Non-muscle myosin heavy chain IIb) (NMMHC II-b) (NMMHC-IIB)	Myh10	77579	2.3003	0.12923	0.88865	0.3185	24.2948875	24.30214	24.4369075	24.32807
Q5SSM3	Rho GTPase-activating protein 44 (Rho-type GTPase-activating protein RICH2) (RhoGAP interacting with CIP4 homologs protein 2) (RICH-2)	Arhgap44 Rich2	216831	2.295	0.12981	0.8867	0.31919	19.632745	19.35102	19.499515	19.231875
Q91ZA3	Propionyl-CoA carboxylase alpha chain, mitochondrial (PCCase subunit alpha) (EC 6.4.1.3) (Propanoyl-CoA:carbon dioxide ligase subunit alpha)	Pcca	110821	2.2922	0.13012	0.88565	0.31945	20.120005	19.97959	19.50102	19.8112925
Q60972	Histone-binding protein RBBP4 (Chromatin assembly factor 1 subunit C) (CAF-1 subunit C) (Chromatin assembly factor I p48 subunit) (CAF-I 48 kDa subunit) (CAF-I p48) (Nucleosome-remodeling factor subunit RBAP48) (Retinoblastoma-binding protein 4) (RBBP-4) (Retinoblastoma-binding protein p48)	Rbbp4 Rbap48	19646	2.279	0.13161	0.88072	0.32225	20.3760925	20.2647625	20.418395	20.3967125
P97352	Protein S100-A13 (S100 calcium-binding protein A13)	S100a13	20196	2.2783	0.13169	0.88046	0.32225	18.131695	18.2953575	19.7270625	18.5166075
Q8BFR5	Elongation factor Tu, mitochondrial	Tufm	233870	2.2735	0.13223	0.87867	0.32306	24.21152	24.147785	24.2608175	24.13836
Q8R570	Synaptosomal-associated protein 47 (SNAP-47) (Synaptosomal-associated 47 kDa protein)	Snap47	67826	2.2699	0.13264	0.87734	0.32354	20.9415075	20.91768	21.238905	21.11578
P11627	Neural cell adhesion molecule L1 (N-CAM-L1) (NCAM-L1) (CD antigen CD171)	L1cam Caml1		2.2672	0.13295	0.87632	0.32378	20.611105	20.6420775	20.8362275	20.61055
Q8K310	Matrin-3	Matr3	17184	2.2622	0.13351	0.87448	0.32464	23.0989525	23.1511825	23.1894125	23.225775
Q9D0M3	Cytochrome c1, heme protein, mitochondrial (EC 7.1.1.8) (Complex III subunit 4) (Complex III subunit IV) (Cytochrome b-c1 complex subunit 4) (Ubiquinol-cytochrome-c reductase complex cytochrome c1 subunit) (Cytochrome c-1)	Cyc1	66445	2.2474	0.13522	0.86895	0.32828	23.39771	23.471655	23.6619675	23.5233275
Q62465	Synaptic vesicle membrane protein VAT-1 homolog (EC 1.-.-.-)	Vat1 Vat-1	26949	2.2391	0.13619	0.86584	0.33011	19.831905	19.5543725	19.9018825	19.6672875
P62717	60S ribosomal protein L18a	Rpl18a	76808	2.2273	0.13759	0.86143	0.33295	21.0991775	20.95523	20.9013	21.2051825
Q99KI0	Aconitate hydratase, mitochondrial (Aconitase) (EC 4.2.1.3) (Citrate hydro-lyase)	Aco2	11429	2.2201	0.13845	0.85872	0.33398	27.23721	27.3280275	27.25462	27.2800375
Q9R1T4	Septin-6	Septin6 Kiaa0128 Sept6	56526	2.2211	0.13833	0.85908	0.33398	22.759955	22.6238	22.8248125	22.598395
O55091	Protein IMPACT (Imprinted and ancient gene protein)	Impact	16210	2.199	0.141	0.85078	0.3396	20.226055	20.2005975	20.0024775	20.0814325
Q99JG2	G-protein coupled receptor 37-like 1 (Endothelin B receptor-like protein 2) (ETBR-LP-2)	Gpr37l1 Etbrlp2	171469	2.1967	0.14128	0.84993	0.33973	20.1397725	20.0319575	19.77211	20.1641675
Q93092	Transaldolase (EC 2.2.1.2)	Taldo1 Tal Taldo	21351	2.1894	0.14217	0.84719	0.34135	22.2682725	22.2503075	22.200705	22.42503
Q8R464	Cell adhesion molecule 4 (Immunoglobulin superfamily member 4C) (IgSF4C) (Nectin-like protein 4) (NECL-4) (TSLC1-like protein 2)	Cadm4 Igsf4c Necl4 Tsll2	260299	2.1815	0.14314	0.84423	0.34314	21.0728675	21.1710425	21.021175	21.3208475

Q6PIE5	Sodium/potassium-transporting ATPase subunit alpha-2 (Na(+)/K(+) ATPase alpha-2 subunit) (EC 7.2.2.13) (Na(+)/K(+) ATPase alpha(+) subunit) (Sodium pump subunit alpha-2)	Atp1a2	98660	2.1723	0.1443	0.84074	0.34537	26.42665	26.4593975	26.310885	26.489565
Q9JMH9	Unconventional myosin-XVIIIa (Molecule associated with JAK3 N-terminus) (MAJN) (Myosin containing a PDZ domain) (Surfactant protein receptor SP-R210) (SP-R210)	Myo18a Myspdz	360013	2.1704	0.14453	0.84004	0.34538	20.4775475	20.52459	20.6484625	20.4686225
B0V2N1	Receptor-type tyrosine-protein phosphatase S (R-PTP-S) (EC 3.1.3.48) (PTPNU-3) (Receptor-type tyrosine-protein phosphatase sigma) (R-PTP-sigma)	Ptprs	19280	2.1684	0.14479	0.83927	0.34546	22.2077875	22.067095	22.1101375	22.0833075
P62918	60S ribosomal protein L8	Rpl8	26961	2.1554	0.14643	0.83436	0.34788	21.8135425	21.6611125	21.73446	21.907955
Q8CBE3	WD repeat-containing protein 37	Wdr37 Kiaa0982	207615	2.155	0.14649	0.8342	0.34788	20.11823	20.1354525	20.1098025	19.9721275
Q9D1R9	60S ribosomal protein L34	Rpl34	619547 68436	2.1554	0.14643	0.83437	0.34788	21.2608325	21.1926375	20.9514275	21.1789575
Q923T9	Calcium/calmodulin-dependent protein kinase type II subunit gamma (CaM kinase II subunit gamma) (CaMK-II subunit gamma) (EC 2.7.11.17)	Camk2g	12325	2.1447	0.1478	0.83032	0.34829	21.981025	21.9373	22.1389525	21.8266875
P60843	Eukaryotic initiation factor 4A-I (eIF-4A-I) (eIF4A-I) (EC 3.6.4.13) (ATP-dependent RNA helicase eIF4A-1)	Eif4a1 Ddx2a Eif4a	13681	2.145	0.14777	0.83043	0.34829	23.0285775	22.94746	23.0857825	23.1283475
Q63810	Calcineurin subunit B type 1 (Protein phosphatase 2B regulatory subunit 1) (Protein phosphatase 3 regulatory subunit B alpha isoform 1)	Ppp3r1 Cnb	19058	2.1459	0.14764	0.83078	0.34829	23.474175	23.4508375	23.5292425	23.191525
Q9JMH6	Thioredoxin reductase 1, cytoplasmic (TR) (EC 1.8.1.9) (Thioredoxin reductase TR1)	Txnrd1 Trxr1	50493	2.1486	0.1473	0.83181	0.34829	20.0589025	20.1691175	20.1365925	20.2203275
Q8R164	Valacyclovir hydrolase (VACVase) (Valacyclovirase) (EC 3.1.-.-) (Biphenyl hydrolase-like protein)	Bphl	68021	2.1481	0.14737	0.83161	0.34829	19.8775825	19.520525	19.664065	19.856285
Q9ERK4	Exportin-2 (Exp2) (Chromosome segregation 1-like protein) (Importin-alpha re-exporter)	Cse1l Xpo2	110750	2.1412	0.14826	0.82899	0.34882	19.6853725	19.668915	19.825155	19.81426
O55143	Sarcoplasmic/endoplasmic reticulum calcium ATPase 2 (SERCA2) (SR Ca(2+)-ATPase 2) (EC 7.2.2.10) (Calcium pump 2) (Calcium-transporting ATPase sarcoplasmic reticulum type, slow twitch skeletal muscle isoform) (Endoplasmic reticulum class 1/2 Ca(2+) ATPase)	Atp2a2	11938	2.1325	0.14938	0.82572	0.35092	24.88301	24.81383	24.900355	24.8494625
Q8C0M9	Isoaspartyl peptidase/L-asparaginase (EC 3.4.19.5) (EC 3.5.1.1) (Asparaginase-like protein 1) (Beta-aspartyl-peptidase) (Isoaspartyl dipeptidase) (L-asparagine amidohydrolase) [Cleaved into: Isoaspartyl peptidase/L-asparaginase alpha chain; Isoaspartyl peptidase/L-asparaginase beta chain]	Asrgl1	66514	2.1283	0.14993	0.82411	0.35168	22.8599625	22.791705	22.562015	22.7752125
P63137	Gamma-aminobutyric acid receptor subunit beta-2 (GABA(A) receptor subunit beta-2)	Gabrb2 Gabrb-2	14401	2.097	0.15409	0.81223	0.35585	20.5663075	20.55233	20.6029625	20.38152
Q8R050	Eukaryotic peptide chain release factor GTP-binding subunit ERF3A (Eukaryotic peptide chain release factor subunit 3a) (eRF3a) (G1 to S phase transition protein 1 homolog)	Gspt1 Erf3a	14852	2.0976	0.15401	0.81244	0.35585	19.3742675	19.765525	19.59572	19.865395
P03911	NADH-ubiquinone oxidoreductase chain 4 (EC 7.1.1.2) (NADH dehydrogenase subunit 4)	Mtnd4 mt-Nd4 Nd4	17719	2.0968	0.15412	0.81214	0.35585	21.15215	21.15125	21.608955	21.3260725
P70336	Rho-associated protein kinase 2 (EC 2.7.11.1) (Rho-associated, coiled-coil-containing protein kinase 2) (Rho-associated, coiled-coil-containing protein kinase II) (ROCK-II) (p164 ROCK-2)	Rock2	19878	2.1018	0.15344	0.81406	0.35585	21.488015	21.2073675	21.381725	21.42013

Q62261	Spectrin beta chain, non-erythrocytic 1 (Beta-II spectrin) (Embryonic liver fodrin) (Fodrin beta chain)	Sptbn1 Elf Spnb-2 Spnb2 Sptb2	20742	2.1012	0.15353	0.81382	0.35585	27.3577225	27.3883375	27.36938	27.320435
Q9WUM3	Coronin-1B (Coronin-2)	Coro1b	23789	2.1	0.15368	0.81337	0.35585	20.8036575	20.848315	20.8670175	20.946675
Q9ERT9	Protein phosphatase 1 regulatory subunit 1A (Protein phosphatase inhibitor 1) (I-1) (IPP-1)	Ppp1r1a	58200	2.1099	0.15236	0.81712	0.35585	19.39776	19.4834525	19.7059625	19.4374025
Q6ZWV7	60S ribosomal protein L35	Rpl35	66489	2.0956	0.15427	0.81172	0.35585	20.7927775	20.815285	20.7037825	20.9882125
Q8BGB7	Enolase-phosphatase E1 (EC 3.1.3.77) (2,3-diketo-5-methylthio-1-phosphopentane phosphatase) (MASA homolog)	Enoph1 Masa	67870	2.0993	0.15378	0.81309	0.35585	19.47522	19.291485	19.5278025	19.364375
Q80Z38	SH3 and multiple ankyrin repeat domains protein 2 (Shank2) (Cortactin-binding protein 1) (CortBP1)	Shank2 Cortbp1 Kiaa1022	210274	2.1111	0.1522	0.8176	0.35585	19.480815	19.373935	19.5078975	19.127155
Q8BLJ3	PI-PLC X domain-containing protein 3	Plcxd3	239318	2.1007	0.15359	0.81364	0.35585	19.806495	19.8003725	20.05257	19.4760825
P57776	Elongation factor 1-delta (EF-1-delta)	Eef1d	66656	2.0914	0.15484	0.81012	0.35662	21.0375475	21.059075	21.166285	21.270055
Q9CQ62	2,4-dienoyl-CoA reductase, mitochondrial (EC 1.3.1.34) (2,4-dienoyl-CoA reductase [NADPH]) (4-enoyl-CoA reductase [NADPH])	Decr1	67460	2.0896	0.1551	0.8094	0.35668	19.409345	19.80846	19.6029625	19.618875
Q6P9K9	Neurexin-3 (Neurexin III-alpha) (Neurexin-3-alpha)	Nrxn3	18191	2.0842	0.15582	0.80737	0.35732	19.92332	19.986915	19.8184775	19.517625
Q6NXK7	Inactive dipeptidyl peptidase 10 (Dipeptidyl peptidase X) (DPP X)	Dpp10	269109	2.0841	0.15584	0.80731	0.35732	21.3815575	21.7273325	21.678265	21.719675
Q60634	Flotillin-2 (Epidermal surface antigen) (ESA) (Membrane component chromosome 17 surface marker 1 homolog)	Flot2 Esa1 M17s1	14252	2.0812	0.15624	0.80622	0.3574	21.2943125	21.3750225	21.27532	21.2375075
P04370	Myelin basic protein (MBP) (Myelin A1 protein)	Mbp Shi	17196	2.077	0.15682	0.8046	0.3574	28.53891	28.64793	28.40335	28.6230975
Q60692	Proteasome subunit beta type-6 (EC 3.4.25.1) (Low molecular mass protein 19) (Macropain delta chain) (Multicatalytic endopeptidase complex delta chain) (Proteasome delta chain) (Proteasome subunit Y)	Psmb6 Lmp19	19175	2.0783	0.15664	0.80509	0.3574	21.5744525	21.3830225	21.26786	21.48401
P62858	40S ribosomal protein S28	Rps28	54127	2.0799	0.15642	0.80571	0.3574	21.51703	21.3895375	21.4783975	21.43792
Q6X893	Choline transporter-like protein 1 (Solute carrier family 44 member 1) (CD antigen CD92)	Slc44a1 Cd92 Cdw92 Ctl1	100434	2.0693	0.15788	0.80166	0.35876	19.982935	20.107035	19.883535	20.4164725
Q91WC3	Long-chain-fatty-acid--CoA ligase 6 (EC 6.2.1.3) (Arachidonate--CoA ligase) (EC 6.2.1.15) (Long-chain acyl-CoA synthetase 6) (LACS 6)	Acsl6 Facl6	216739	2.0693	0.15788	0.80167	0.35876	22.0234975	22.113025	22.16071	22.2753875
Q68FD5	Clathrin heavy chain 1	Cltc	67300	2.0625	0.15882	0.79908	0.36036	28.079745	28.16562	28.238705	28.17573
Q04447	Creatine kinase B-type (EC 2.7.3.2) (B-CK) (Creatine kinase B chain) (Creatine phosphokinase B-type) (CPK-B)	Ckb Ckbb	12709	2.0604	0.15912	0.79826	0.36051	27.8953075	27.96048	28.0257025	28.04868
Q9JKB1	Ubiquitin carboxyl-terminal hydrolase isozyme L3 (UCH-L3) (EC 3.4.19.12) (Ubiquitin thioesterase L3)	Uchl3	50933	2.0585	0.15939	0.79754	0.36057	21.3683875	21.2287	21.4622375	21.263605
E9Q401	Ryanodine receptor 2 (RYR-2) (RyR2) (Cardiac muscle ryanodine receptor) (Cardiac muscle ryanodine receptor-calcium release channel) (Type 2 ryanodine receptor)	Ryr2	20191	2.05	0.16058	0.79431	0.36235	20.1700475	19.9793225	20.0747475	19.6875975
O35682	Myeloid-associated differentiation marker (Myeloid up-regulated protein)	Myadm Mug	50918	2.0495	0.16065	0.79412	0.36235	19.57986	19.2665425	19.4359575	19.28019
P97478	5-demethoxyubiquinone hydroxylase, mitochondrial (DMQ hydroxylase) (EC 1.14.13.-) (Timing protein clk-1 homolog) (Ubiquinone biosynthesis monooxygenase COQ7)	Coq7	12850	2.0472	0.16098	0.79322	0.36257	20.3190125	20.412415	20.16596	20.2878175

Q01768	Nucleoside diphosphate kinase B (NDK B) (NDP kinase B) (EC 2.7.4.6) (Histidine protein kinase NDKB) (EC 2.7.13.3) (P18) (nm23-M2)	Nme2	18103	2.0411	0.16185	0.79089	0.36374	20.82418	21.2812875	21.0021825	21.34826
P99026	Proteasome subunit beta type-4 (EC 3.4.25.1) (Low molecular mass protein 3) (Macropain beta chain) (Multicatalytic endopeptidase complex beta chain) (Proteasome beta chain) (Proteasome chain 3)	Psmb4 Lmp3	19172	2.0402	0.16198	0.79054	0.36374	21.1233675	20.8822325	20.9756425	20.87658
Q64514	Tripeptidyl-peptidase 2 (TPP-2) (EC 3.4.14.10) (Tripeptidyl aminopeptidase) (Tripeptidyl-peptidase II) (TPP-II)	Tpp2	22019	2.0336	0.16292	0.78804	0.3653	21.11985	20.8575975	21.0408675	20.9808025
Q60668	Heterogeneous nuclear ribonucleoprotein D0 (hnRNP D0) (AU-rich element RNA-binding protein 1)	Hnrnpd Auf1 Hnrpd	11991	2.0289	0.16359	0.78624	0.36628	23.04563	23.0936875	22.997385	23.1760375
P09671	Superoxide dismutase [Mn], mitochondrial (EC 1.15.1.1)	Sod2 Sod-2	20656	2.0266	0.16393	0.78533	0.36651	23.673285	23.75485	23.636	23.7533225
Q08274	Dystrophia myotonica WD repeat-containing protein (Dystrophia myotonica-containing WD repeat motif protein) (Protein DMR-N9)	Dmwd Dm9	13401	2.0224	0.16454	0.78372	0.36734	19.7124425	19.4269825	19.2772025	19.131225
P29341	Polyadenylate-binding protein 1 (PABP-1) (Poly(A)-binding protein 1)	Pabpc1 Pabp1	18458	2.0189	0.16505	0.78239	0.36793	22.590635	22.5669625	22.6267175	22.7165075
P05064	Fructose-bisphosphate aldolase A (EC 4.1.2.13) (Aldolase 1) (Muscle-type aldolase)	Aldoa Aldo1	11674	2.0102	0.16633	0.77903	0.3684	28.18695	28.2272625	28.247895	28.1767825
P07901	Heat shock protein HSP 90-alpha (Heat shock 86 kDa) (HSP 86) (HSP86) (Tumor-specific transplantation 86 kDa antigen) (TSTA)	Hsp90aa1 Hsp86 Hsp86-1 Hspca	15519	2.0122	0.16603	0.77981	0.3684	25.8297125	25.7873175	25.9141375	25.8326825
P70202	Latexin (Endogenous carboxypeptidase inhibitor) (ECI) (Tissue carboxypeptidase inhibitor) (TCI)	Lxn	17035	1.9994	0.16792	0.77489	0.3684	21.5936175	21.552635	21.5919925	21.7888725
P28667	MARCKS-related protein (Brain protein F52) (MARCKS-like protein 1) (Macrophage myristoylated alanine-rich C kinase substrate) (Mac-MARCKS) (MacMARCKS)	Marcksl1 Mlp Mrp	17357	1.9978	0.16816	0.77428	0.3684	20.3029475	19.965855	20.1299375	19.78903
P14152	Malate dehydrogenase, cytoplasmic (EC 1.1.1.37) (Cytosolic malate dehydrogenase)	Mdh1 Mor2	17449	1.9988	0.16801	0.77466	0.3684	27.0166675	26.9760375	27.014905	26.980545
P62075	Mitochondrial import inner membrane translocase subunit Tim13	Timm13 Tim13a Timm13a	30055	2.0036	0.1673	0.77651	0.3684	21.098115	20.896255	20.9940875	20.901705
O55022	Membrane-associated progesterone receptor component 1 (mPR)	Pgrmc1 Pgrmc	53328	2.0066	0.16685	0.77767	0.3684	21.993685	22.422665	22.106815	22.199795
Q8R326	Paraspeckle component 1 (Paraspeckle protein 1) (mPSP1)	Pspc1 Psp1	66645	2.0055	0.16702	0.77722	0.3684	20.6379775	20.6739675	20.6260525	20.591945
Q7TNP2	Serine/threonine-protein phosphatase 2A 65 kDa regulatory subunit A beta isoform (PP2A subunit A isoform PR65-beta) (PP2A subunit A isoform R1-beta)	Ppp2r1b	73699	2.0124	0.16601	0.77988	0.3684	21.4929575	21.050025	21.2127325	21.091855
Q6ZQK5	Arf-GAP with coiled-coil, ANK repeat and PH domain-containing protein 2 (Centaurin-beta-2) (Cnt-b2)	Acap2 Centb2 Kiaa0041	78618	2.0114	0.16615	0.7795	0.3684	18.7664575	19.2694075	18.4885725	18.57583
Q8JZK9	Hydroxymethylglutaryl-CoA synthase, cytoplasmic (HMG-CoA synthase) (EC 2.3.3.10) (3-hydroxy-3-methylglutaryl coenzyme A synthase)	Hmgcs1	208715	2.0075	0.16672	0.77801	0.3684	18.9460025	18.3896425	19.0951825	19.10734
Q8BHB9	Chloride intracellular channel protein 6	Clic6	209195	2.0013	0.16764	0.77563	0.3684	18.80330975	17.79600525	13.7502615	17.9047905
O55125	Protein NipSnap homolog 1 (NipSnap1)	Nipsnap1	18082	1.9911	0.16915	0.77172	0.3691	22.58473	22.6880175	22.6673025	22.4012975
P08228	Superoxide dismutase [Cu-Zn] (EC 1.15.1.1)	Sod1	20655	1.9906	0.16924	0.7715	0.3691	25.0996975	25.110965	24.9150725	25.0988275
Q9WUM5	Succinate--CoA ligase [ADP/GDP-forming] subunit alpha, mitochondrial (EC 6.2.1.4) (EC 6.2.1.5) (Succinyl-CoA synthetase subunit alpha) (SCS-alpha)	Suclg1	56451	1.9876	0.16969	0.77036	0.3691	23.541885	23.6509025	23.5872925	23.7185025

Q9D1K2	V-type proton ATPase subunit F (V-ATPase subunit F) (V-ATPase 14 kDa subunit) (Vacuolar proton pump subunit F)	Atp6v1f Atp6s14 Vatf	66144	1.9901	0.16931	0.77131	0.3691	22.21941	22.11957	22.2508775	22.12736
Q810U4	Neuronal cell adhesion molecule (Nr-CAM) (Neuronal surface protein Bravo) (mBravo) (NgCAM-related cell adhesion molecule) (Ng-CAM-related)	Nrcam Kiaa0343	319504	1.9889	0.16949	0.77085	0.3691	22.632895	22.4526525	22.63077	22.57165
P62746	Rho-related GTP-binding protein RhoB	Rhob Arhb	11852	1.9846	0.17014	0.76918	0.36957	22.2511725	22.11876	22.05369	21.836475
Q99JY9	Actin-related protein 3 (Actin-like protein 3)	Actr3 Arp3	74117	1.9803	0.17078	0.76756	0.37043	24.605165	24.56274	24.5795475	24.47934
Q60865	Caprin-1 (Cytoplasmic activation- and proliferation-associated protein 1) (GPI-anchored membrane protein 1) (GPI-anchored protein p137) (GPI-p137) (p137GPI) (Membrane component chromosome 11 surface marker 1) (RNA granule protein 105)	Caprin1 Gpiap Gpiap1 Gpip137 M11s1 Rng105 G5E5	53872	1.9767	0.17133	0.76617	0.37056	19.95406	19.8963025	19.8524075	20.027505
Q9CQR4	Acyl-coenzyme A thioesterase 13 (Acyl-CoA thioesterase 13) (EC 3.1.2.-) (Thioesterase superfamily member 2)	Acot13 Them2	66834	1.9783	0.1711	0.76675	0.37056	21.992285	21.96932	21.807325	21.943025
P53026	60S ribosomal protein L10a (CSA-19) (Neural precursor cell expressed developmentally down-regulated protein 6) (NEDD-6)	Rpl10a Nedd-6 Nedd6		1.97	0.17236	0.76357	0.37174	20.961535	20.9642475	20.990515	21.0892725
Q8R2R9	AP-3 complex subunit mu-2 (Adaptor-related protein complex 3 subunit mu-2) (Clathrin assembly protein assembly protein complex 3 mu-2 medium chain) (Clathrin coat assembly protein AP47 homolog 2) (Clathrin coat-associated protein AP47 homolog 2) (Golgi adaptor AP-1 47 kDa protein homolog 2) (HA1 47 kDa subunit homolog 2) (Mu3B-adaptin) (m3B) (P47B)	Ap3m2	64933	1.9703	0.17232	0.76367	0.37174	23.23347	22.58191	22.6592975	23.03037
P26516	26S proteasome non-ATPase regulatory subunit 7 (26S proteasome regulatory subunit RPN8) (26S proteasome regulatory subunit S12) (Mov34 protein) (Proteasome subunit p40)	Psmd7 Mov-34 Mov34	17463	1.9602	0.17387	0.75978	0.37289	18.979025	18.401705	18.88962	18.8515625
Q9JL62	Glycolipid transfer protein (GLTP)	Gltp	56356	1.9615	0.17366	0.7603	0.37289	19.6450225	19.61048	19.58479	19.9325825
Q9DCZ1	GMP reductase 1 (GMPR 1) (EC 1.7.1.7) (Guanosine 5'-monophosphate oxidoreductase 1) (Guanosine monophosphate reductase 1)	Gmpr Gmpr1	66355	1.9612	0.17371	0.76017	0.37289	19.14202	18.84337	19.00103	18.673755
Q8BG32	26S proteasome non-ATPase regulatory subunit 11 (26S proteasome regulatory subunit RPN6) (26S proteasome regulatory subunit S9) (26S proteasome regulatory subunit p44.5)	Psmd11	69077	1.963	0.17343	0.76088	0.37289	21.7323425	21.58457	21.778875	21.7471975
O08997	Copper transport protein ATOX1 (Metal transport protein ATX1)	Atox1	11927	1.9555	0.17459	0.75797	0.37392	20.5109025	20.718725	20.3092375	20.9204525
Q8BW96	Calcium/calmodulin-dependent protein kinase type 1D (EC 2.7.11.17) (CaM kinase I delta) (CaM-KI delta) (CaMKI delta) (CaM kinase ID) (CaMKI-like protein kinase) (CKLiK) (mCKLiK)	Camk1d	227541	1.9514	0.17524	0.75637	0.37478	21.39442	21.27285	21.3350175	21.00776
Q9R0K7	Plasma membrane calcium-transporting ATPase 2 (PMCA2) (EC 7.2.2.10) (Plasma membrane calcium ATPase isoform 2) (Plasma membrane calcium pump isoform 2)	Atp2b2 Pmca2	11941	1.9484	0.1757	0.75522	0.37524	24.6386025	24.528585	24.5193025	24.37445
Q921M7	Protein FAM49B	Fam49b	223601	1.9429	0.17656	0.7531	0.37656	22.567335	22.5104425	22.687915	22.464145
O54901	OX-2 membrane glycoprotein (MRC OX-2 antigen) (CD antigen CD200)	Cd200 Mox2		1.9399	0.17704	0.75193	0.37669	20.94984	20.80048	20.87692	20.6563975
Q8BHE3	Caytaxin	Atcay	16467	1.9394	0.17712	0.75173	0.37669	20.5871175	20.79687	20.946655	20.4572025
Q9R0P3	S-formylglutathione hydrolase (FGH) (EC 3.1.2.12) (Esterase 10) (Esterase D) (Sid 478)	Esd Es10 Sid478	13885	1.9303	0.17856	0.74821	0.3787	22.1083	22.1163975	22.183915	22.2409275

Q9D5V5	Cullin-5 (CUL-5)	Cul5	75717	1.9317	0.17833	0.74876	0.3787	19.1732825	19.239835	19.46992	19.2284975
P48318	Glutamate decarboxylase 1 (EC 4.1.1.15) (67 kDa glutamic acid decarboxylase) (GAD-67) (Glutamate decarboxylase 67 kDa isoform)	Gad1 Gad67	14415	1.9207	0.1801	0.74449	0.38143	21.8125175	21.6859075	21.7444075	21.64902
P68181	cAMP-dependent protein kinase catalytic subunit beta (PKA C-beta) (EC 2.7.11.11)	Prkacb Pkacb	18749	1.9177	0.18058	0.74333	0.38192	22.292625	22.3777975	22.4648225	22.29237
Q8BP47	Asparagine--tRNA ligase, cytoplasmic (EC 6.1.1.22) (Asparaginyl-tRNA synthetase) (AsnRS) (Asparaginyl-tRNA synthetase 1)	NARS1 Nars	70223	1.9147	0.18106	0.74217	0.3824	22.02164	21.834545	22.0278375	21.861315
P47915	60S ribosomal protein L29	Rpl29 Rpl43	19944	1.9132	0.18131	0.74158	0.3824	19.190305	19.2781425	18.8343375	18.627415
P62748	Hippocalcin-like protein 1 (Neural visinin-like protein 3) (NVL-3) (NVP-3) (Visinin-like protein 3) (VILIP-3)	Hpcal1	53602	1.9108	0.18171	0.74063	0.38271	19.9620275	20.2695625	20.1347325	19.972275
P06801	NADP-dependent malic enzyme (NADP-ME) (EC 1.1.1.40) (Malic enzyme 1)	Me1 Mod-1 Mod1	17436	1.9085	0.18207	0.73975	0.38296	21.1115675	21.136895	21.4282275	21.2061275
P45878	Peptidyl-prolyl cis-trans isomerase FKBP2 (PPIase FKBP2) (EC 5.2.1.8) (13 kDa FK506-binding protein) (13 kDa FKBP) (FKBP-13) (FK506-binding protein 2) (FKBP-2) (Immunophilin FKBP13) (Rotamase)	Fkbp2 Fkbp13	14227	1.9002	0.18344	0.73651	0.3853	20.5626475	20.551105	20.7119125	20.64112
O35643	AP-1 complex subunit beta-1 (Adaptor protein complex AP-1 subunit beta-1) (Adaptor-related protein complex 1 subunit beta-1) (Beta-1-adaptin) (Beta-adaptin 1) (Clathrin assembly protein complex 1 beta large chain) (Golgi adaptor HA1/AP1 adaptin beta subunit)	Ap1b1 Adtb1	11764	1.8883	0.1854	0.7319	0.38887	24.1948725	24.206705	24.0873425	24.358025
P84099	60S ribosomal protein L19	Rpl19	19921	1.8825	0.18637	0.72963	0.39034	20.765465	20.7155975	20.6884125	20.92402
Q9DBG3	AP-2 complex subunit beta (AP105B) (Adaptor protein complex AP-2 subunit beta) (Adaptor-related protein complex 2 subunit beta) (Beta-2-adaptin) (Beta-adaptin) (Clathrin assembly protein complex 2 beta large chain) (Plasma membrane adaptor HA2/AP2 adaptin beta subunit)	Ap2b1 Clapb1	71770	1.8811	0.18661	0.72907	0.39034	25.9068125	25.9173	25.9086125	25.84641
Q8VE33	Ganglioside-induced differentiation-associated protein 1-like 1 (GDAP1-L1)	Gdap1l1 Gdap1l		1.8724	0.18806	0.7257	0.39104	20.99004	20.952685	21.2181675	21.08673
P47738	Aldehyde dehydrogenase, mitochondrial (EC 1.2.1.3) (AHD-M1) (ALDH class 2) (ALDH-E2) (ALDHI)	Aldh2 Ahd-1 Ahd1	11669	1.876	0.18745	0.72711	0.39104	23.4834875	23.634915	23.33621	23.5046825
Q9JM52	Misshapen-like kinase 1 (EC 2.7.11.1) (GCK family kinase MiNK) (MAPK/ERK kinase kinase kinase 6) (MEK kinase kinase 6) (MEKKK 6) (Misshapen/NIK-related kinase) (Mitogen-activated protein kinase kinase kinase kinase 6)	Mink1 Map4k6 Mink	50932	1.8729	0.18798	0.7259	0.39104	20.4886525	20.375765	20.3984275	20.1603675
Q8BH66	Atlastin-1 (EC 3.6.5.-) (Spastic paraplegia 3A homolog)	Atl1 Spg3a	73991	1.8749	0.18765	0.72665	0.39104	21.9887	21.86403	22.026305	21.9020675
Q99LR1	Lysophosphatidylserine lipase ABHD12 (EC 3.1.1.-) (2-arachidonoylglycerol hydrolase ABHD12) (Abhydrolase domain-containing protein 12) (Monoacylglycerol lipase ABHD12) (EC 3.1.1.23) (Oxidized phosphatidylserine lipase ABHD12) (EC 3.1.-.-)	Abhd12	76192	1.8715	0.18822	0.72533	0.39104	20.201665	20.35325	20.342295	20.5564025
Q5EBJ4	Ermin (Juxtanodin) (JN)	Ermn Kiaa1189	77767	1.8696	0.18854	0.72459	0.39118	20.556005	20.531615	20.06891	20.7982375
Q9CPV4	Glyoxalase domain-containing protein 4	Glod4	67201	1.8644	0.18942	0.72258	0.39246	22.2263625	22.42306	22.28275	22.392895
Q9QYB1	Chloride intracellular channel protein 4 (mc3s5/mtCLIC)	Clic4	29876	1.8572	0.19065	0.71975	0.39448	20.1279125	20.3002475	20.150415	20.3822825
P62137	Serine/threonine-protein phosphatase PP1-alpha catalytic subunit (PP-1A) (EC 3.1.3.16)	Ppp1ca Ppp1a	19045	1.8533	0.19132	0.71824	0.39532	21.3409525	21.5574625	21.481205	21.5302325

P63101	14-3-3 protein zeta/delta (Protein kinase C inhibitor protein 1) (KCIP-1) (SEZ-2)	Ywhaz	22631	1.8497	0.19195	0.71681	0.3961	27.9458575	27.93098	27.955	27.8132125
P51830	Adenylate cyclase type 9 (EC 4.6.1.1) (ATP pyrophosphate-lyase 9) (Adenylate cyclase type IX) (Adenylyl cyclase 9) (AC9) (Adenylyl cyclase type 10) (ACTP10)	Adcy9	11515	1.8454	0.1927	0.71512	0.3971	19.69338	19.6420375	19.791265	19.4903475
Q99L43	Phosphatidate cytidylyltransferase 2 (EC 2.7.7.41) (CDP-DAG synthase 2) (CDP-DG synthase 2) (CDP-diacylglycerol synthase 2) (CDS 2) (CDP-diglyceride pyrophosphorylase 2) (CDP-diglyceride synthase 2) (CTP:phosphatidate cytidylyltransferase 2)	Cds2	110911	1.8382	0.19395	0.71231	0.39914	21.0882125	21.0920375	21.0291375	20.906075
P20357	Microtubule-associated protein 2 (MAP-2)	Map2 Mtap2	17756	1.8313	0.19516	0.7096	0.40109	26.716375	26.650575	26.76082	26.58352
O08788	Dynactin subunit 1 (150 kDa dynein-associated polypeptide) (DAP-150) (DP-150) (p150-glued)	Dctn1	13191	1.8211	0.19696	0.70562	0.40316	23.3115825	23.0575075	23.1062675	23.0674925
Q9Z0S1	3'(2'),5'-bisphosphate nucleotidase 1 (EC 3.1.3.7) (Bisphosphate 3'-nucleotidase 1) (PAP-inositol 1,4-phosphatase) (PIP)	Bpnt1	23827	1.8213	0.19693	0.7057	0.40316	22.3861275	22.295875	22.258435	22.2476475
Q9Z2U0	Proteasome subunit alpha type-7 (EC 3.4.25.1) (Proteasome subunit RC6-1)	Psma7	26444	1.8212	0.19695	0.70566	0.40316	21.69097	21.6826225	21.7343925	21.8547275
Q9QYB5	Gamma-adducin (Adducin-like protein 70)	Add3 Addl	27360	1.8148	0.19809	0.70314	0.40321	24.223955	24.4577475	24.2002025	24.53628
P28656	Nucleosome assembly protein 1-like 1 (Brain protein DN38) (NAP-1-related protein)	Nap1l1 Nrp	53605	1.8194	0.19727	0.70493	0.40321	21.643505	21.725895	21.91891	21.7925425
P63325	40S ribosomal protein S10	Rps10	67097	1.8153	0.198	0.70333	0.40321	20.31554	20.4030225	20.5251275	20.523165
Q80UG2	Plexin-A4	Plxna4 Kiaa1550	243743	1.8171	0.19768	0.70404	0.40321	20.2548825	20.1580425	20.22583	20.0120825
Q9Z0H8	CAP-Gly domain-containing linker protein 2 (Cytoplasmic linker protein 115) (CLIP-115) (Cytoplasmic linker protein 2)	Clip2 Cyln2 Kiaa0291	269713	1.8136	0.1983	0.70267	0.40321	20.309265	20.241545	20.2152475	20.0937475
P46660	Alpha-internexin (Alpha-Inx) (66 kDa neurofilament protein) (NF-66) (Neurofilament-66)	Ina	226180	1.8049	0.19986	0.69927	0.40584	25.482535	25.4730275	25.341795	25.7147275
P52196	Thiosulfate sulfurtransferase (EC 2.8.1.1) (Rhodanese)	Tst	22117	1.7968	0.20134	0.69607	0.40721	21.5501775	21.5298975	21.5393675	21.75814
Q8CIW6	Solute carrier family 26 member 6 (Anion exchange transporter) (Chloride-formate exchanger) (Pendrin-L1) (Pendrin-like protein 1) (Putative anion transporter-1) (Pat-1)	Slc26a6 Cfex Pat1	171429	1.7971	0.20129	0.69617	0.40721	20.849515	20.647635	20.383345	20.6673325
Q922E4	Ethanolamine-phosphate cytidylyltransferase (EC 2.7.7.14) (CTP:phosphoethanolamine cytidylyltransferase) (Phosphorylethanolamine transferase)	Pcyt2	68671	1.7977	0.20118	0.69642	0.40721	18.555195	18.24756	18.6736375	18.19056
P55264	Adenosine kinase (AK) (EC 2.7.1.20) (Adenosine 5'-phosphotransferase)	Adk	11534	1.7864	0.20325	0.69197	0.41053	21.914265	21.89262	21.66765	21.8601875
P63323	40S ribosomal protein S12	Rps12		1.7848	0.20354	0.69134	0.41058	21.48197	21.37451	21.5128375	21.5889125
Q99J08	SEC14-like protein 2 (Alpha-tocopherol-associated protein) (TAP)	Sec14l2	67815	1.7798	0.20447	0.68938	0.4119	20.174925	20.0526725	20.030335	20.06225
Q9Z1S5	Neuronal-specific septin-3	Septin3 Sep3 Sept3	24050	1.7776	0.20489	0.68848	0.41221	23.4170025	23.3860375	23.4439225	23.3386925
Q6GSS7	Histone H2A type 2-A (H2a-614) (H2a-615) (Histone H2A.2)	Hist2h2aa1; Hist2h2aa2	15267 319192	1.7723	0.20587	0.6864	0.41364	26.5243975	26.62431	26.5865225	26.74332
Q80UG5	Septin-9 (SL3-3 integration site 1 protein)	Septin9 Kiaa0991 Sept9 Sint1	53860	1.7682	0.20663	0.6848	0.41463	21.703525	21.5005175	21.7675975	21.747085
P61027	Ras-related protein Rab-10	Rab10	19325	1.762	0.20781	0.68234	0.41484	23.648165	23.537345	23.5424875	23.4146375
P35278	Ras-related protein Rab-5C	Rab5c	19345	1.7605	0.2081	0.68173	0.41484	20.99602	20.9167875	21.03972	20.9006325

Q8BG51	Mitochondrial Rho GTPase 1 (MIRO-1) (EC 3.6.5.-) (Ras homolog gene family member T1)	Rhot1 Arht1	59040	1.7642	0.20738	0.68323	0.41484	20.2510825	20.2390725	20.06814	20.1451525
Q921H8	3-ketoacyl-CoA thiolase A, peroxisomal (EC 2.3.1.16) (Acetyl-CoA acyltransferase A) (Beta-ketothiolase A) (Peroxisomal 3-oxoacyl-CoA thiolase A)	Acaa1a Acaa1	113868	1.7605	0.2081	0.68174	0.41484	19.436975	19.2739625	19.638955	19.9118825
Q8BGZ1	Hippocalcin-like protein 4 (Neural visinin-like protein 2) (NVP-2)	Hpcal4	170638	1.7662	0.20702	0.68399	0.41484	23.4055575	23.4331075	23.672045	23.3891875
Q9QWI6	SRC kinase signaling inhibitor 1 (SNAP-25-interacting protein) (SNIP) (p130Cas-associated protein) (p140Cap)	Srcin1 Kiaa1684 P140	56013	1.7579	0.20858	0.68072	0.41526	22.9396375	22.94882	22.96294	22.805145
Q9D898	Actin-related protein 2/3 complex subunit 5-like protein (Arp2/3 complex 16 kDa subunit 2) (ARC16-2)	Arpc5l	74192	1.7563	0.20889	0.68008	0.41534	20.7091625	20.9376925	20.937645	20.6532025
Q8BKC5	Importin-5 (Imp5) (Importin subunit beta-3) (Karyopherin beta-3) (Ran-binding protein 5) (RanBP5)	Ipo5 Kpnb3 Ranbp5	70572	1.7507	0.20994	0.6779	0.41689	20.335625	20.23299	20.4349525	20.414965
P48678	Prelamin-A/C [Cleaved into: Lamin-A/C]	Lmna Lmn1	16905	1.7475	0.21057	0.6766	0.41734	22.370225	22.3273175	22.306805	22.580265
Q8BG39	Synaptic vesicle glycoprotein 2B (Synaptic vesicle protein 2B)	Sv2b Kiaa0735	64176	1.7467	0.21072	0.67629	0.41734	24.321015	24.26816	24.379985	24.1973325
P97370	Sodium/potassium-transporting ATPase subunit beta-3 (Sodium/potassium-dependent ATPase subunit beta-3) (ATPB-3) (CD antigen CD298)	Atp1b3	11933	1.7443	0.21117	0.67536	0.4177	21.003325	20.95489	20.9090375	21.0943925
Q8K1M6	Dynamin-1-like protein (EC 3.6.5.5) (Dynamin family member proline-rich carboxyl-terminal domain less) (Dymple) (Dynamin-related protein 1)	Dnm1l Drp1	74006	1.7406	0.21189	0.67388	0.41858	23.826325	23.7830675	23.9388925	23.8142
O88447	Kinesin light chain 1 (KLC 1)	Klc1 Kns2		1.7301	0.21393	0.66974	0.42042	20.791215	20.670745	20.78451	20.2779875
Q62433	Protein NDRG1 (N-myc downstream-regulated gene 1 protein) (Protein Ndr1)	Ndrg1 Ndr1 Ndrl Tdd5	17988	1.7314	0.21367	0.67025	0.42042	19.8528225	20.0626025	19.7332225	20.1966875
Q61937	Nucleophosmin (NPM) (Nucleolar phosphoprotein B23) (Nucleolar protein NO38) (Numatrin)	Npm1	18148	1.7333	0.2133	0.67101	0.42042	23.087325	22.94555	23.0952725	22.8996075
Q9D172	Glutamine amidotransferase-like class 1 domain-containing protein 3A, mitochondrial	Gatd3a D10Jhu81e	28295	1.7325	0.21347	0.67066	0.42042	23.67039	23.7490375	23.6334625	23.7537025
Q9DBS2	Tumor protein p63-regulated gene 1-like protein (Mossy fiber terminal-associated vertebrate-specific presynaptic protein) (Protein FAM79A)	Tprg1l Fam79a Mover Tprgl	67808	1.7271	0.21451	0.66854	0.42103	20.4439375	20.6672175	20.5919425	20.39719
O88643	Serine/threonine-protein kinase PAK 1 (EC 2.7.11.1) (Alpha-PAK) (CDC42/RAC effector kinase PAK-A) (p21-activated kinase 1) (PAK-1) (p65-PAK)	Pak1 Paka		1.7226	0.2154	0.66675	0.42166	21.7413025	21.64018	21.76136	21.6056025
Q922Q8	Leucine-rich repeat-containing protein 59 [Cleaved into: Leucine-rich repeat-containing protein 59, N-terminally processed]	Lrrc59	98238	1.7213	0.21566	0.66623	0.42166	20.1815175	20.2480625	20.3221525	20.4017575
Q8K1B8				1.7224	0.21543	0.66669	0.42166	19.0068975	18.809505	18.5544	18.6988325
Q9CQ75	NADH dehydrogenase [ubiquinone] 1 alpha subcomplex subunit 2 (Complex I-B8) (CI-B8) (NADH-ubiquinone oxidoreductase B8 subunit)	Ndufa2	17991	1.711	0.2177	0.66214	0.42186	20.63081	20.4590525	20.41265	20.3201325
P42208	Septin-2 (Neural precursor cell expressed developmentally down-regulated protein 5) (NEDD-5)	Septin2 Nedd-5 Nedd5 Sept2	18000	1.716	0.21671	0.66412	0.42186	20.64204	20.751585	20.62618	20.8541625
P15532	Nucleoside diphosphate kinase A (NDK A) (NDP kinase A) (EC 2.7.4.6) (Metastasis inhibition factor NM23) (NDPK-A) (Tumor metastatic process-associated protein) (nm23-M1)	Nme1 Nm23	18102	1.7193	0.21605	0.66544	0.42186	25.0971425	25.1597825	25.0710075	25.2437675
Q61206	Platelet-activating factor acetylhydrolase IB subunit beta (EC 3.1.1.47) (PAF acetylhydrolase 30 kDa subunit) (PAF-AH 30	Pafah1b2 Pafahb	18475	1.711	0.21769	0.66215	0.42186	22.4614175	22.587085	22.636415	22.48457

	kDa subunit) (PAF-AH subunit beta) (PAFAH subunit beta)										
Q9EQH3	Vacuolar protein sorting-associated protein 35 (Maternal-embryonic 3) (Vesicle protein sorting 35)	Vps35 Mem3	65114	1.7119	0.21751	0.66252	0.42186	22.584645	22.67192	22.579845	22.632905
Q640R3	Hepatocyte cell adhesion molecule (Protein hepaCAM)	Hepacam	72927	1.7122	0.21745	0.66263	0.42186	21.62344	21.793925	21.7023825	21.92022
Q80T41	Gamma-aminobutyric acid type B receptor subunit 2 (GABA-B receptor 2) (GABA-B-R2) (GABA-BR2) (GABABR2) (Gb2) (G-protein coupled receptor 51)	Gabbr2 Gm425 Gpr51	242425	1.7172	0.21646	0.66462	0.42186	19.50288	19.361815	19.403985	19.14083
Q8BKX1	Brain-specific angiogenesis inhibitor 1-associated protein 2 (BAI-associated protein 2) (BAI1-associated protein 2) (Insulin receptor substrate protein of 53 kDa) (IRSp53) (Insulin receptor substrate p53) (Insulin receptor tyrosine kinase 53 kDa substrate)	Baiap2	108100	1.7038	0.21913	0.6593	0.4241	22.81754	22.83521	22.9392	22.7610075
Q99L47	Hsc70-interacting protein (Hip) (Protein FAM10A1) (Protein ST13 homolog)	St13 Fam10a1 Hip	70356	1.702	0.21951	0.65855	0.42429	21.6516225	21.4180625	21.5001875	21.52448
Q7TNS2	MICOS complex subunit Mic10 (Mitochondrial inner membrane organizing system protein 1)	Micos10 Mic10 Minos1	433771	1.6998	0.21994	0.65769	0.42459	19.4630625	19.0158875	19.1341025	18.9082175
Q8R1Q8	Cytoplasmic dynein 1 light intermediate chain 1 (Dynein light chain A) (DLC-A) (Dynein light intermediate chain 1, cytosolic)	Dync1li1 Dncli1 Dnclic1	235661	1.6949	0.22092	0.65576	0.42595	21.499335	21.515435	21.663125	21.4737575
Q91WS0	CDGSH iron-sulfur domain-containing protein 1 (MitoNEET)	Cisd1 D10Ertd214e Zcd1	52637	1.6889	0.22216	0.65334	0.42779	23.5688075	23.4223825	23.4003225	23.4016775
P07310	Creatine kinase M-type (EC 2.7.3.2) (Creatine kinase M chain) (Creatine phosphokinase M-type) (CPK-M) (M-CK)	Ckm Ckmm	12715	1.6865	0.22264	0.6524	0.42817	17.403175	18.7427025	18.057335	19.258625
Q8BG05	Heterogeneous nuclear ribonucleoprotein A3 (hnRNP A3)	Hnrnpa3 Hnrpa3	229279	1.6743	0.22515	0.64752	0.43246	23.74997	23.691235	23.7645125	23.7704025
Q9CQJ8	NADH dehydrogenase [ubiquinone] 1 beta subcomplex subunit 9 (Complex I-B22) (CI-B22) (NADH-ubiquinone oxidoreductase B22 subunit)	Ndufb9	66218	1.6722	0.22557	0.64671	0.43273	21.2474775	20.9408225	21.18711	20.9599775
P26443	Glutamate dehydrogenase 1, mitochondrial (GDH 1) (EC 1.4.1.3)	Glud1 Glud	14661	1.6661	0.22686	0.64425	0.43464	26.1542375	26.0922775	26.0361175	26.1851875
P34022	Ran-specific GTPase-activating protein (HpaII tiny fragments locus 9a protein) (Ran-binding protein 1) (RANBP1)	Ranbp1 Htf9-a Htf9a	19385	1.6582	0.2285	0.64111	0.43724	21.657345	21.5592375	21.5177425	21.6230225
A2A690	Protein TANC2 (Tetratricopeptide repeat, ankyrin repeat and coiled-coil domain-containing protein 2)	Tanc2 Kiaa1148	77097	1.6545	0.22927	0.63965	0.43817	22.12545	22.0279275	22.1962775	22.0636775
P18872	Guanine nucleotide-binding protein G(o) subunit alpha	Gnao1 Gna0 Gnao	14681	1.6496	0.23031	0.63769	0.43892	27.21007	27.255115	27.207925	27.1475925
P13595	Neural cell adhesion molecule 1 (N-CAM-1) (NCAM-1) (CD antigen CD56)	Ncam1 Ncam	17967	1.6513	0.22996	0.63835	0.43892	26.0664925	25.9922675	25.996575	25.94034
Q62443	Neuronal pentraxin-1 (NP1) (Neuronal pentraxin I) (NP-I)	Nptx1	18164	1.6486	0.23052	0.63728	0.43892	21.5859575	21.53704	21.63118	21.4295275
P62812	Gamma-aminobutyric acid receptor subunit alpha-1 (GABA(A) receptor subunit alpha-1)	Gabra1 Gabra-1	14394	1.6466	0.23096	0.63647	0.43898	19.91916	19.686455	19.73268	19.657965
P62484	Abl interactor 2 (Abelson interactor 2) (Abi-2) (Abl-binding protein 3) (AblBP3) (Arg-binding protein 1) (ArgBP1)	Abi2	329165	1.6457	0.23114	0.63613	0.43898	20.1299175	20.3000775	20.04033	19.9680975
Q9DAK9	14 kDa phosphohistidine phosphatase (EC 3.9.1.3) (Phosphohistidine phosphatase 1) (PHPT1) (Protein histidine phosphatase) (PHP)	Phpt1 Php14	75454	1.6443	0.23144	0.63557	0.43901	19.2601925	19.1476925	19.0474725	19.2116325
Q9WTT4	V-type proton ATPase subunit G 2 (V-ATPase subunit G 2) (V-ATPase 13 kDa subunit 2) (Vacuolar proton pump subunit G 2)	Atp6v1g2 Atp6g2 Ng38	66237	1.6404	0.23228	0.63398	0.44007	22.5142025	22.1933175	22.3672175	22.2361775
P62889	60S ribosomal protein L30	Rpl30	19946	1.6375	0.23289	0.63284	0.4404	19.5112325	19.93717	20.1290275	20.1385925

P19253	60S ribosomal protein L13a (Transplantation antigen P198) (Tum-P198 antigen)	Rpl13a P198 Tstap198-7	22121	1.636	0.23322	0.63223	0.4404	20.372565	19.8906075	20.154315	20.59265
O08539	Myc box-dependent-interacting protein 1 (Amphiphysin II) (Amphiphysin-like protein) (Bridging integrator 1) (SH3 domain-containing protein 9)	Bin1 Amphl Sh3p9	30948	1.6355	0.23332	0.63204	0.4404	23.952735	23.95357	23.98444	23.92924
P16054	Protein kinase C epsilon type (EC 2.7.11.13) (nPKC-epsilon)	Prkce Pkce Pkcea	18754	1.6288	0.23478	0.62934	0.44042	22.939975	22.81682	22.829225	22.842845
O35678	Monoglyceride lipase (MGL) (EC 3.1.1.23) (Monoacylglycerol lipase) (MAGL)	Mgll	23945	1.632	0.23409	0.63062	0.44042	21.33746	21.3586275	21.5030125	21.4415025
Q9ES28	Rho guanine nucleotide exchange factor 7 (Beta-Pix) (PAK-interacting exchange factor beta) (p85SPR)	Arhgef7 Kiaa0142 Pak3bp	54126	1.6309	0.23431	0.63021	0.44042	19.8435025	20.16145	19.9897875	20.0147025
Q9D023	Mitochondrial pyruvate carrier 2 (Brain protein 44)	Mpc2 Brp44	70456	1.6299	0.23453	0.6298	0.44042	21.8428575	21.6630875	21.879935	21.6776975
Q8BH04	Phosphoenolpyruvate carboxykinase [GTP], mitochondrial (PEPCK-M) (EC 4.1.1.32)	Pck2	74551	1.6303	0.23445	0.62996	0.44042	20.5056225	20.422785	20.2320875	19.934475
O35129	Prohibitin-2 (B-cell receptor-associated protein BAP37) (Repressor of estrogen receptor activity)	Phb2 Bap Bcap37 Rea	12034	1.6219	0.23627	0.62659	0.44054	23.46487	23.3627575	23.511755	23.3344525
P23927	Alpha-crystallin B chain (Alpha(B)-crystallin) (P23)	Cryab Crya2	12955	1.6245	0.23571	0.62763	0.44054	19.43798	19.713175	19.243085	20.108425
Q8BH95	Enoyl-CoA hydratase, mitochondrial (EC 4.2.1.17) (Enoyl-CoA hydratase 1) (Short-chain enoyl-CoA hydratase) (SCEH)	Echs1	93747	1.6257	0.23545	0.6281	0.44054	21.8531175	21.7135475	21.8244675	21.733495
Q8K0T0	Reticulon-1 (Neuroendocrine-specific protein)	Rtn1 Nsp	104001	1.6266	0.23524	0.62849	0.44054	24.0506325	24.158875	24.0719875	23.9290625
P17182	Alpha-enolase (EC 4.2.1.11) (2-phospho-D-glycerate hydro-lyase) (Enolase 1) (Non-neural enolase) (NNE)	Eno1 Eno-1	13806 433182	1.6219	0.23628	0.62657	0.44054	28.2997075	28.300785	28.3913	28.257975
Q9R0Y5	Adenylate kinase isoenzyme 1 (AK 1) (EC 2.7.4.3) (EC 2.7.4.6) (ATP-AMP transphosphorylase 1) (ATP:AMP phosphotransferase) (Adenylate monophosphate kinase) (Myokinase)	Ak1	11636	1.6125	0.23832	0.62284	0.4412	23.8115	23.8810825	23.9564025	23.9220675
P62827	GTP-binding nuclear protein Ran (GTPase Ran) (Ras-like protein TC4) (Ras-related nuclear protein)	Ran Rasl2-8	19384	1.6097	0.23895	0.6217	0.4412	23.5960125	23.60716	23.4521675	23.63242
P97461	40S ribosomal protein S5 [Cleaved into: 40S ribosomal protein S5, N-terminally processed]	Rps5	20103	1.6116	0.23854	0.62244	0.4412	20.41439	20.3512975	20.3265025	20.415515
P53986	Monocarboxylate transporter 1 (MCT 1) (Solute carrier family 16 member 1)	Slc16a1 Mct1	20501	1.6146	0.23787	0.62367	0.4412	18.8897575	19.1590975	19.0004625	19.377595
Q8C8R3	Ankyrin-2 (ANK-2) (Ankyrin-B) (Brain ankyrin)	Ank2 AnkB	109676	1.6126	0.23831	0.62285	0.4412	25.199355	25.18206	25.286135	25.1912275
A6H690	IQ and AAA domain-containing protein 1-like (Protein IQCA1P1)	Iqca1l Iqca1p1	231045	1.6184	0.23703	0.6252	0.4412	21.39261	21.184655	21.1195075	21.1159325
Q5M8N0	CB1 cannabinoid receptor-interacting protein 1 (CRIP-1)	Cnrip1	380686	1.6139	0.23803	0.62336	0.4412	22.88075	22.901855	22.9471275	22.7436125
O54962	Barrier-to-autointegration factor (Breakpoint cluster region protein 1) (LAP2-binding protein 1) [Cleaved into: Barrier-to-autointegration factor, N-terminally processed]	Banf1 Baf Bcrp1 L2bp1	23825	1.6101	0.23886	0.62185	0.4412	20.7531375	19.87218	19.847125	20.0027775
Q8CAA7	Glucose 1,6-bisphosphate synthase (EC 2.7.1.106) (Phosphoglucomutase-2-like 1)	Pgm2l1	70974	1.5974	0.24167	0.61677	0.44516	22.8754075	22.853785	22.9411825	22.8159
Q810U3	Neurofascin	Nfasc	269116	1.5975	0.24165	0.61681	0.44516	23.853365	23.90695	23.925985	23.996725
Q9D7G0	Ribose-phosphate pyrophosphokinase 1 (EC 2.7.6.1) (Phosphoribosyl pyrophosphate synthase I) (PRS-I)	Prps1	19139	1.5926	0.24277	0.61481	0.44663	20.97431	21.2074525	20.9870575	21.1025075
Q8R191	Synaptogyrin-3	Syngr3	20974	1.5872	0.24397	0.61266	0.44831	23.08334	23.04276	23.16649	22.9513825

Q9WV98	Mitochondrial import inner membrane translocase subunit Tim9	Timm9 Tim9 Tim9a Timm9a	30056	1.5842	0.24465	0.61146	0.44901	21.6717725	21.5695975	21.4690275	21.55871
P47955	60S acidic ribosomal protein P1	Rplp1	56040	1.5814	0.24529	0.61032	0.44965	21.277915	21.6655125	21.743045	21.8272325
Q8JZN5	Complex I assembly factor ACAD9, mitochondrial (Acyl-CoA dehydrogenase family member 9) (ACAD-9) (EC 1.3.8.-)	Acad9	229211	1.5753	0.24669	0.60785	0.45167	20.2101975	20.4041825	20.2244	20.3824525
Q8CHG7	Rap guanine nucleotide exchange factor 2 (Cyclic nucleotide ras GEF) (CNrasGEF) (Neural RAP guanine nucleotide exchange protein) (nRap GEP) (PDZ domain-containing guanine nucleotide exchange factor 1) (PDZ-GEF1) (RA-GEF-1) (Ras/Rap1-associating GEF-1)	Rapgef2 Kiaa0313 Pdzgef1		1.5717	0.2475	0.60642	0.45262	20.12634	19.9344275	19.97509	19.6417625
Q9WV34	MAGUK p55 subfamily member 2 (Discs large homolog 2) (Protein MPP2)	Mpp2 Dlgh2	50997	1.5629	0.24953	0.60288	0.45469	20.5912825	20.74164	20.7176325	20.3885275
Q9Z2I0	Mitochondrial proton/calcium exchanger protein (Leucine zipper-EF-hand-containing transmembrane protein 1)	Letm1	56384	1.563	0.24951	0.60291	0.45469	22.6267475	22.5736625	22.718345	22.52023
Q8K4Z3	NAD(P)H-hydrate epimerase (EC 5.1.99.6) (Apolipoprotein A-I-binding protein) (AI-BP) (NAD(P)HX epimerase)	Naxe Aibp Apoa1bp	246703	1.5654	0.24897	0.60386	0.45469	20.54795	20.687055	20.8510175	20.568405
Q8VE52	Opioid growth factor receptor-like protein 1	Ogfrl1	70155	1.559	0.25044	0.6013	0.4558	19.2641475	19.056995	19.1874975	18.62585
Q9D6J5	NADH dehydrogenase [ubiquinone] 1 beta subcomplex subunit 8, mitochondrial (Complex I-ASHI) (CI-ASHI) (NADH-ubiquinone oxidoreductase ASHI subunit)	Ndufb8	67264	1.5533	0.25177	0.599	0.45768	21.8701125	21.704885	21.81174	21.66064
Q9JM63	ATP-sensitive inward rectifier potassium channel 10 (Inward rectifier K(+) channel Kir4.1) (Potassium channel, inwardly rectifying subfamily J member 10)	Kcnj10	16513	1.5519	0.25209	0.59844	0.45772	19.3711625	19.0694825	19.4929175	19.69413
Q64133	Amine oxidase [flavin-containing] A (EC 1.4.3.4) (Monoamine oxidase type A) (MAO-A)	Maoa	17161	1.5457	0.25357	0.5959	0.45986	20.0424025	20.1763525	20.065545	20.1772375
Q9WV02	RNA-binding motif protein, X chromosome (Heterogeneous nuclear ribonucleoprotein G) (hnRNP G) [Cleaved into: RNA-binding motif protein, X chromosome, N-terminally processed]	Rbmx Hnrnpg Hnrpg Rbmxp1 Rbmxrt	19655	1.5388	0.2552	0.59312	0.46172	21.7901975	21.757975	21.757835	21.6940425
P60122	RuvB-like 1 (EC 3.6.4.12) (49 kDa TATA box-binding protein-interacting protein) (49 kDa TBP-interacting protein) (DNA helicase p50) (Pontin 52) (TIP49a)	Ruvbl1 Tip49 Tip49a	56505	1.5389	0.25517	0.59316	0.46172	19.7838125	19.187455	19.42888	19.4032925
P47809	Dual specificity mitogen-activated protein kinase kinase 4 (MAP kinase kinase 4) (MAPKK 4) (EC 2.7.12.2) (C-JUN N-terminal kinase kinase 1) (JNK kinase 1) (JNKK 1) (JNK-activating kinase 1) (MAPK/ERK kinase 4) (MEK 4) (SAPK/ERK kinase 1) (SEK1)	Map2k4 Jnkk1 Mek4 Mkk4 Prkmk4 Sek1 Serk1 Skk1	26398	1.5351	0.25606	0.59166	0.46273	20.9313075	20.877625	20.91667	20.79767
P56382	ATP synthase subunit epsilon, mitochondrial (ATPase subunit epsilon) (ATP synthase F1 subunit epsilon)	Atp5f1e Atp5e	67126	1.5319	0.25685	0.59033	0.46276	22.63867	22.533695	22.3065575	22.4969475
Q6P069	Sorcin	Sri	109552	1.5313	0.25699	0.59009	0.46276	19.9793	19.849545	19.6297875	19.9236275
P83882	60S ribosomal protein L36a (60S ribosomal protein L44)	Rpl36a Rpl44	19982 66483	1.5332	0.25654	0.59085	0.46276	19.3753425	19.15625	19.142665	19.442005
O09131	Glutathione S-transferase omega-1 (GSTO-1) (EC 2.5.1.18) (Glutathione S-transferase omega 1-1) (GSTO 1-1) (Glutathione-dependent dehydroascorbate reductase) (EC 1.8.5.1) (Monomethylarsonic acid reductase) (MMA(V) reductase) (EC 1.20.4.2) (S-(Phenacyl)glutathione reductase) (SPG-R) (p28)	Gsto1 Gstx Gtsttl	14873	1.5265	0.25813	0.58816	0.46317	19.976885	20.106335	19.8689525	20.08764
P54227	Stathmin (Leukemia-associated gene protein) (Leukemia-associated phosphoprotein p18)	Stmn1 Lag Lap18 Pr22	16765	1.528	0.25778	0.58875	0.46317	23.0840575	23.1200225	23.259415	23.22774

	(Metablastin) (Oncoprotein 18) (Op18) (Phosphoprotein p19) (pp19) (Prosolin) (Protein Pr22) (pp17)										
Q9Z1P6	NADH dehydrogenase [ubiquinone] 1 alpha subcomplex subunit 7 (Complex I-B14.5a) (CI-B14.5a) (NADH-ubiquinone oxidoreductase subunit B14.5a)	Ndufa7	66416	1.5268	0.25806	0.58828	0.46317	22.34271	22.1386075	21.9814125	21.9703075
P62874	Guanine nucleotide-binding protein G(I)/G(S)/G(T) subunit beta-1 (Transducin beta chain 1)	Gnb1	14688	1.5213	0.2594	0.58603	0.46491	26.5590375	26.5756825	26.654635	26.5037025
O09111	NADH dehydrogenase [ubiquinone] 1 beta subcomplex subunit 11, mitochondrial (Complex I-ESSS) (CI-ESSS) (NADH-ubiquinone oxidoreductase ESSS subunit) (Neuronal protein 15.6) (Np15.6) (p15.6)	Ndufb11 Np15	104130	1.515	0.26093	0.58348	0.4671	21.6456425	21.5031775	21.4325075	21.453995
P06151	L-lactate dehydrogenase A chain (LDH-A) (EC 1.1.1.27) (LDH muscle subunit) (LDH-M)	Ldha Ldh-1 Ldh1	16828	1.5093	0.2623	0.5812	0.46816	25.8183225	25.7496725	25.8402	25.7972275
P48722	Heat shock 70 kDa protein 4L (Heat shock 70-related protein APG-1) (Osmotic stress protein 94)	Hspa4l Apg1 Hsp4l Osp94	18415	1.5092	0.26232	0.58117	0.46816	23.95763	23.8304275	23.9702075	23.9706
P61982	14-3-3 protein gamma [Cleaved into: 14-3-3 protein gamma, N-terminally processed]	Ywhag	22628	1.5088	0.26244	0.58098	0.46816	26.4693725	26.38972	26.5063525	26.36224
Q9WV69	Dematin (Dematin actin-binding protein) (Erythrocyte membrane protein band 4.9)	Dmtn Epb4.9 Epb49	13829	1.5056	0.26322	0.57968	0.469	22.69244	22.5713375	22.6038775	22.5224775
Q9WTP6	Adenylate kinase 2, mitochondrial (AK 2) (EC 2.7.4.3) (ATP-AMP transphosphorylase 2) (ATP:AMP phosphotransferase) (Adenylate monophosphate kinase)	Ak2	11637	1.498	0.2651	0.5766	0.4699	21.164745	21.02926	20.7939025	20.6754725
O54774	AP-3 complex subunit delta-1 (AP-3 complex subunit delta) (Adaptor-related protein complex 3 subunit delta-1) (Delta-adaptin) (mBLVR1)	Ap3d1 Ap3d	11776	1.4995	0.26471	0.57723	0.4699	22.17141	22.1904675	22.26461	22.05567
P62821	Ras-related protein Rab-1A (YPT1-related protein)	Rab1A Rab1	19324	1.4973	0.26526	0.57632	0.4699	24.49403	24.422785	24.434465	24.374545
Q9R0X4	Acyl-coenzyme A thioesterase 9, mitochondrial (Acyl-CoA thioesterase 9) (EC 3.1.2.-) (Acyl coenzyme A thioester hydrolase 2) (MTE-2) (Acyl-CoA thioester hydrolase 9) (Mitochondrial 48 kDa acyl-CoA thioester hydrolase 1) (Mt-ACT48.1) (Protein U8) (p48)	Acot9 Acate2	56360	1.5003	0.26452	0.57755	0.4699	19.661065	19.669025	19.62247	19.267925
Q6R891	Neurabin-2 (Neurabin-II) (Protein phosphatase 1 regulatory subunit 9B) (Spinophilin)	Ppp1r9b	217124	1.4994	0.26474	0.57717	0.4699	22.2610575	22.1752075	22.2037525	22.073735
P54775	26S proteasome regulatory subunit 6B (26S proteasome AAA-ATPase subunit RPT3) (CIP21) (MB67-interacting protein) (MIP224) (Proteasome 26S subunit ATPase 4) (Tat-binding protein 7) (TBP-7)	Psmc4 Tbp7	23996	1.4941	0.26605	0.57504	0.47021	18.994315	18.90955	19.3169925	19.14117
Q0VE82	Copine-7 (Copine VII)	Cpne7	102278	1.4946	0.26594	0.57522	0.47021	19.6636675	19.421285	19.077665	19.48596
O08756	3-hydroxyacyl-CoA dehydrogenase type-2 (EC 1.1.1.35) (17-beta-hydroxysteroid dehydrogenase 10) (17-beta-HSD 10) (EC 1.1.1.51) (3-hydroxy-2-methylbutyryl-CoA dehydrogenase) (EC 1.1.1.178) (3-hydroxyacyl-CoA dehydrogenase type II) (Endoplasmic reticulum-associated amyloid beta-peptide-binding protein) (Mitochondrial ribonuclease P protein 2) (Mitochondrial RNase P protein 2) (Type II HADH)	Hsd17b10 Erab Hadh2		1.4918	0.26663	0.57408	0.47046	20.04721	20.2375225	20.3376425	20.2456175
Q3UHJ0	AP2-associated protein kinase 1 (EC 2.7.11.1) (Adaptor-associated kinase 1)	Aak1 Kiaa1048	269774	1.4899	0.26712	0.5733	0.47046	23.6901525	23.621955	23.7065475	23.6164075
P04104	Keratin, type II cytoskeletal 1 (67 kDa cytokeratin) (Cytokeratin-1) (CK-1) (Keratin-1) (K1) (Type-II keratin Kb1)	Krt1 Krt2-1	16678	1.4905	0.26695	0.57357	0.47046	19.22394	17.28608	18.68088	18.360275

Q3TEA8	Heterochromatin protein 1-binding protein 3	Hp1bp3	15441	1.4852	0.26829	0.5714	0.47198	20.073755	19.7380925	19.912075	19.4584225
P63038	60 kDa heat shock protein, mitochondrial (EC 5.6.1.7) (60 kDa chaperonin) (Chaperonin 60) (CPN60) (HSP-65) (Heat shock protein 60) (HSP-60) (Hsp60) (Mitochondrial matrix protein P1)	Hspd1 Hsp60	15510	1.4759	0.27062	0.56763	0.47529	26.2380925	26.179595	26.2104425	26.15312
Q6PEB6	MOB-like protein phocein (Class II mMOB1) (Mob1 homolog 3) (Mob3) (Mps one binder kinase activator-like 3) (Preimplantation protein 3)	Mob4 Mob3 Mobkl3 Phocn Prei3	19070	1.4744	0.27101	0.56701	0.47529	20.0040725	19.8595825	19.9038475	19.845635
P67984	60S ribosomal protein L22 (Heparin-binding protein HBp15)	Rpl22	19934	1.4728	0.27141	0.56637	0.47529	20.87127	20.897055	21.013705	20.97934
Q99PF4	Cadherin-23 (Otocadherin)	Cdh23	22295	1.4739	0.27112	0.56683	0.47529	20.96333	21.0530875	21.3539325	21.1196025
Q6ZWX6	Eukaryotic translation initiation factor 2 subunit 1 (Eukaryotic translation initiation factor 2 subunit alpha) (eIF-2-alpha) (eIF-2A) (eIF-2alpha)	Eif2s1 Eif2a	13665	1.4666	0.27299	0.56386	0.47662	20.3457325	20.202445	20.2874	20.4108625
Q9Z2Q6	Septin-5 (Cell division control-related protein 1) (CDCrel-1) (Peanut-like protein 1)	Septin5 Pnutl1 Sept5	18951	1.4661	0.27311	0.56366	0.47662	24.6897225	24.7279725	24.8045075	24.6369075
Q9Z1N5	Spliceosome RNA helicase Ddx39b (EC 3.6.4.13) (56 kDa U2AF65-associated protein) (DEAD box protein UAP56) (HLA-B-associated transcript 1 protein)	Ddx39b Bat1 Bat1a Uap56	53817	1.468	0.27263	0.56443	0.47662	21.721585	21.6880775	21.8402475	21.9450275
O88485	Cytoplasmic dynein 1 intermediate chain 1 (Cytoplasmic dynein intermediate chain 1) (Dynein intermediate chain 1, cytosolic) (DH IC-1)	Dync1i1 Dnci1 Dncic1	13426	1.4605	0.27457	0.56134	0.47862	20.7383875	20.611105	20.601835	20.4553925
Q9R0Q7	Prostaglandin E synthase 3 (EC 5.3.99.3) (Cytosolic prostaglandin E2 synthase) (cPGES) (Hsp90 co-chaperone) (Progesterone receptor complex p23) (Sid 3177) (Telomerase-binding protein p23)	Ptges3 Sid3177 Tebp	56351	1.4583	0.27512	0.56048	0.47902	22.303865	22.4197875	22.44147	22.4972125
O55042	Alpha-synuclein (Non-A beta component of AD amyloid) (Non-A4 component of amyloid precursor) (NACP)	Snca Syn	20617	1.4503	0.2772	0.5572	0.48156	24.7682075	24.57919	24.382685	24.4469475
Q9D6R2	Isocitrate dehydrogenase [NAD] subunit alpha, mitochondrial (EC 1.1.1.41) (Isocitric dehydrogenase subunit alpha) (NAD(+)-specific ICDH subunit alpha)	Idh3a	67834	1.4509	0.27705	0.55744	0.48156	25.66623	25.6988825	25.69715	25.642295
Q9DCX2	ATP synthase subunit d, mitochondrial (ATPase subunit d) (ATP synthase peripheral stalk subunit d)	Atp5pd Atp5h	71679	1.447	0.27806	0.55586	0.48195	25.0912825	25.1693625	25.118905	25.1745375
Q6ZPE2	Myotubularin-related protein 5 (Inactive phosphatidylinositol 3-phosphatase 5) (SET-binding factor 1) (Sbf1)	Sbf1 Kiaa3020 Mtmr5	77980	1.4473	0.27798	0.55599	0.48195	20.0892375	20.000865	20.06084	19.91853
Q9D8W5	26S proteasome non-ATPase regulatory subunit 12 (26S proteasome regulatory subunit RPN5) (26S proteasome regulatory subunit p55)	Psmd12	66997	1.4376	0.28052	0.55204	0.48566	20.5017	20.7084175	20.7202875	20.6955625
Q78PY7	Staphylococcal nuclease domain-containing protein 1 (EC 3.1.31.1) (100 kDa coactivator) (p100 co-activator)	Snd1	56463	1.4355	0.28107	0.55119	0.48581	19.83865	20.121565	19.9817525	20.068435
O54984	ATPase Asna1 (EC 3.6.-.-) (Arsenical pump-driving ATPase) (Arsenite-stimulated ATPase)	Asna1 Arsa	56495	1.4349	0.28124	0.55092	0.48581	20.7371275	20.7210425	20.832395	20.82248
P58771	Tropomyosin alpha-1 chain (Alpha-tropomyosin) (Tropomyosin-1)	Tpm1 Tpm-1 Tpma	22003	1.4276	0.28317	0.54795	0.48859	21.0334925	21.14632	20.8828025	21.11441
Q9JIS5	Synaptic vesicle glycoprotein 2A (Synaptic vesicle protein 2) (Synaptic vesicle protein 2A) (Calcium regulator SV2A)	Sv2a Kiaa0736 Sv2	64051	1.4228	0.28447	0.54597	0.49027	24.4071925	24.3449375	24.34384	24.2525125
P62073	Mitochondrial import inner membrane translocase subunit Tim10	Timm10 Tim10	30059	1.4211	0.28492	0.54527	0.4905	19.4393725	18.65794	19.4005525	18.5894375
P63085	Mitogen-activated protein kinase 1 (MAP kinase 1) (MAPK 1) (EC 2.7.11.24) (ERT1)	Mapk1 Erk2 Mapk Prkm1	26413	1.4177	0.28582	0.5439	0.49095	24.677245	24.809595	24.737395	24.6689325

	(Extracellular signal-regulated kinase 2) (ERK-2) (MAP kinase isoform p42) (p42-MAPK) (Mitogen-activated protein kinase 2) (MAP kinase 2) (MAPK 2)										
P40336	Vacuolar protein sorting-associated protein 26A (H<beta>58 protein) (H beta 58) (Vesicle protein sorting 26A) (mVPS26)	Vps26a Vps26	30930	1.4183	0.28568	0.54412	0.49095	19.7159625	19.5343525	19.8274225	19.3499425
Q9CXZ1	NADH dehydrogenase [ubiquinone] iron-sulfur protein 4, mitochondrial (Complex I-18 kDa) (CI-18 kDa) (Complex I-AQDQ) (CI-AQDQ) (NADH-ubiquinone oxidoreductase 18 kDa subunit)	Ndufs4	17993	1.4157	0.28636	0.54308	0.49132	22.154315	22.0254875	22.06853	22.026625
O35226	26S proteasome non-ATPase regulatory subunit 4 (26S proteasome regulatory subunit RPN10) (26S proteasome regulatory subunit S5A) (Multiubiquitin chain-binding protein)	Psmd4 Mcb1	19185	1.4125	0.28723	0.54177	0.4922	19.9806525	19.738925	19.7000125	19.9721925
P62315	Small nuclear ribonucleoprotein Sm D1 (Sm-D1) (Sm-D autoantigen) (snRNP core protein D1)	Snrpd1	20641	1.4114	0.28752	0.54133	0.4922	20.21361	20.297455	20.30163	20.3515025
P56399	Ubiquitin carboxyl-terminal hydrolase 5 (EC 3.4.19.12) (Deubiquitinating enzyme 5) (Isopeptidase T) (Ubiquitin thioesterase 5) (Ubiquitin-specific-processing protease 5)	Usp5 Isot	22225	1.4066	0.28883	0.53936	0.49236	23.4984475	23.57924	23.654485	23.573685
Q91XV3	Brain acid soluble protein 1 (22 kDa neuronal tissue-enriched acidic protein) (Neuronal axonal membrane protein NAP-22)	Basp1 Nap22	70350	1.4063	0.28891	0.53924	0.49236	27.374535	27.2597925	27.16413	26.9667875
Q9DC07	LIM zinc-binding domain-containing Nebulette (Actin-binding Z-disk protein)	Nebl Lnebl	74103	1.4083	0.28838	0.54003	0.49236	21.5706175	21.3351525	21.26678	21.184005
Q8BZ98	Dynamin-3 (EC 3.6.5.5)	Dnm3 Kiaa0820	103967	1.409	0.28818	0.54034	0.49236	21.9877525	21.942455	22.11576	21.9903675
Q9CPX8	Cytochrome b-c1 complex subunit 10 (Complex III subunit 10) (Complex III subunit XI) (Ubiquinol-cytochrome c reductase complex 6.4 kDa protein)	Uqcr11 Uqcr	66594	1.4039	0.28957	0.53824	0.49295	20.11217	20.390105	20.2842675	20.21394
P62881	Guanine nucleotide-binding protein subunit beta-5 (Gbeta5) (Transducin beta chain 5)	Gnb5	14697	1.3891	0.29364	0.53219	0.49932	21.77916	21.7145175	21.7904925	21.595925
Q9DC69	NADH dehydrogenase [ubiquinone] 1 alpha subcomplex subunit 9, mitochondrial (Complex I-39kD) (CI-39kD) (NADH-ubiquinone oxidoreductase 39 kDa subunit)	Ndufa9	66108	1.3811	0.29587	0.52889	0.50224	23.53299	23.66699	23.7128625	23.6314525
Q3UIU2	NADH dehydrogenase [ubiquinone] 1 beta subcomplex subunit 6 (Complex I-B17) (CI-B17) (NADH-ubiquinone oxidoreductase B17 subunit)	Ndufb6 Gm137	230075	1.3806	0.29602	0.52868	0.50224	21.240345	21.1645075	21.3893875	21.196575
P42125	Enoyl-CoA delta isomerase 1, mitochondrial (EC 5.3.3.8) (3,2-trans-enoyl-CoA isomerase) (Delta(3),Delta(2)-enoyl-CoA isomerase) (D3,D2-enoyl-CoA isomerase) (Dodecenoyl-CoA isomerase)	Eci1 Dci	13177	1.377	0.29703	0.5272	0.50229	19.7322975	19.9157025	19.7437225	19.8499
P56387	Dynein light chain Tctex-type 3 (Protein 91/23) (T-complex-associated testis-expressed 1-like)	Dynlt3 Tcte1l	67117	1.3772	0.29697	0.52729	0.50229	22.4384325	22.5850625	22.603485	22.71936
Q6PE15	Mycophenolic acid acyl-glucuronide esterase, mitochondrial (EC 3.1.1.93) (Alpha/beta hydrolase domain-containing protein 10) (Abhydrolase domain-containing protein 10)	Abhd10	213012	1.3778	0.2968	0.52754	0.50229	19.9192	19.8011475	19.87448	19.7486525
Q99MN1	Lysine--tRNA ligase (EC 2.7.7.-) (EC 6.1.1.6) (Lysyl-tRNA synthetase) (LysRS)	Kars1 Kars	85305	1.3712	0.29864	0.52485	0.50402	19.14164	19.3539575	19.407265	19.38885
E9Q3L2	Phosphatidylinositol 4-kinase alpha (PI4-kinase alpha) (PI4K-alpha) (PtdIns-4-kinase alpha) (EC 2.7.1.67)	Pi4ka Pik4 Pik4ca	224020	1.371	0.29872	0.52474	0.50402	20.250825	20.1947975	20.262025	20.27373
O88441	Metaxin-2 (Mitochondrial outer membrane import complex protein 2)	Mtx2 MNCb-0780	53375	1.3676	0.29968	0.52334	0.50509	19.971505	19.6281925	20.0041325	19.99053
P28474	Alcohol dehydrogenase class-3 (EC 1.1.1.1) (Alcohol dehydrogenase 2) (Alcohol dehydrogenase 5) (Alcohol dehydrogenase B2) (ADH-B2) (Alcohol dehydrogenase class-III) (Glutathione-dependent formaldehyde	Adh5 Adh-2 Adh2	11532	1.3609	0.30157	0.52062	0.50771	22.19582	22.0778575	22.068145	22.1933725

	dehydrogenase) (FALDH) (FDH) (GSH-FDH) (EC 1.1.1.-) (S-(hydroxymethyl)glutathione dehydrogenase) (EC 1.1.1.284)										
O88844	Isocitrate dehydrogenase [NADP] cytoplasmic (IDH) (EC 1.1.1.42) (Cytosolic NADP-isocitrate dehydrogenase) (IDP) (NADP(+)-specific ICDH) (Oxalosuccinate decarboxylase)	Idh1	15926	1.3585	0.30226	0.51962	0.50831	22.6751425	22.6020825	22.7110625	22.6621275
P11031	Activated RNA polymerase II transcriptional coactivator p15 (Positive cofactor 4) (PC4) (SUB1 homolog) (Single-stranded DNA-binding protein p9) (p14)	Sub1 Pc4 Rpo2tc1	20024	1.3568	0.30275	0.51891	0.50859	20.910295	20.8229675	20.8678375	21.014025
Q8BMF3	NADP-dependent malic enzyme, mitochondrial (NADP-ME) (EC 1.1.1.40) (Malic enzyme 3)	Me3	109264	1.3551	0.30324	0.51821	0.50885	19.94371	19.9358675	20.22215	19.9027275
Q71LX4	Talin-2	Tln2	70549	1.3523	0.30404	0.51707	0.50963	21.781315	22.1242925	21.9443275	21.9516775
P14231	Sodium/potassium-transporting ATPase subunit beta-2 (Adhesion molecule in glia) (AMOG) (Sodium/potassium-dependent ATPase subunit beta-2)	Atp1b2	11932	1.3297	0.31061	0.50778	0.51611	24.2887725	24.1936825	24.20698	24.3402
Q62093	Serine/arginine-rich splicing factor 2 (Protein PR264) (Putative myelin regulatory factor 1) (MRF-1) (Splicing component, 35 kDa) (Splicing factor SC35) (SC-35) (Splicing factor, arginine/serine-rich 2)	Srsf2 Pr264 Sfrs10 Sfrs2	20382	1.33	0.31052	0.50791	0.51611	20.5795325	20.2568425	20.43897	20.1516775
P39447	Tight junction protein ZO-1 (Tight junction protein 1) (Zona occludens protein 1) (Zonula occludens protein 1)	Tjp1 Zo1	21872	1.3336	0.30946	0.50939	0.51611	21.6990925	21.573295	21.36038	21.3154
P60335	Poly(rC)-binding protein 1 (Alpha-CP1) (Heterogeneous nuclear ribonucleoprotein E1) (hnRNP E1)	Pcbp1	23983	1.3324	0.30984	0.50886	0.51611	22.7852	22.6774975	22.628145	22.691775
Q9JKD3	Secretory carrier-associated membrane protein 5 (Secretory carrier membrane protein 5)	Scamp5	56807	1.3356	0.30889	0.51019	0.51611	22.2314	21.97881	21.7961725	21.7968675
Q9CQR2	40S ribosomal protein S21	Rps21	66481	1.3316	0.31006	0.50856	0.51611	20.5992175	20.432515	20.51055	20.5993225
Q8C522	Endonuclease domain-containing 1 protein (EC 3.1.30.-)	Endod1 Kiaa0830	71946	1.3377	0.30826	0.51108	0.51611	20.2474975	20.2905725	20.27993	20.44432
P61957	Small ubiquitin-related modifier 2 (SUMO-2) (SMT3 homolog 2) (Ubiquitin-like protein SMT3B) (Smt3B)	Sumo2 Smt3b Smt3h2	170930	1.3305	0.31038	0.50811	0.51611	21.446755	21.39021	21.18522	21.3394325
Q9Z2W0	Aspartyl aminopeptidase (EC 3.4.11.21)	Dnpep	13437	1.3221	0.31287	0.50463	0.51862	19.8037775	19.802795	20.0525025	19.995035
Q9WUB3	Glycogen phosphorylase, muscle form (EC 2.4.1.1) (Myophosphorylase)	Pygm	19309	1.3212	0.31314	0.50426	0.51862	20.5456025	20.426185	20.16468	20.1797425
P41105	60S ribosomal protein L28	Rpl28	19943	1.3217	0.313	0.50446	0.51862	20.91924	20.6965075	20.71302	20.88496
P70670	Nascent polypeptide-associated complex subunit alpha, muscle-specific form (Alpha-NAC, muscle-specific form) (skNAC)	Naca Gm1878	17938	1.3139	0.31531	0.50126	0.52165	21.1339575	21.146115	21.1089	20.9649725
Q9CZ44	NSFL1 cofactor p47 (p97 cofactor p47)	Nsfl1c	386649	1.3118	0.31594	0.50039	0.52213	21.509335	21.4786725	21.6051575	21.6305125
Q61545	RNA-binding protein EWS	Ewsr1 Ews Ewsh	14030	1.3079	0.31713	0.49876	0.52352	19.669335	19.51236	19.6417275	19.602025
Q9JLM8	Serine/threonine-protein kinase DCLK1 (EC 2.7.11.1) (Doublecortin-like and CAM kinase-like 1) (Doublecortin-like kinase 1)	Dclk1 Dcamkl1 Dclk	13175	1.3012	0.31913	0.49603	0.5248	22.3175075	22.46861	22.5359675	22.4212975
P25444	40S ribosomal protein S2 (40S ribosomal protein S4) (Protein LLRep3)	Rps2 Llrep3 Rps4	16898	1.3035	0.31846	0.49695	0.5248	21.6559075	21.6796325	21.67757	21.86287
Q9CQ92	Mitochondrial fission 1 protein (FIS1 homolog) (Tetratricopeptide repeat protein 11) (TPR repeat protein 11)	Fis1 Ttc11	66437	1.299	0.31983	0.49509	0.5248	20.9505825	20.9994125	20.9854525	21.081955
Q9D7X3	Dual specificity protein phosphatase 3 (EC 3.1.3.16) (EC 3.1.3.48) (T-DSP11) (Vaccinia H1-related phosphatase) (VHR)	Dusp3	72349	1.2985	0.31997	0.49489	0.5248	20.99024	20.7879975	20.950045	20.8139825

Q9QYS2	Metabotropic glutamate receptor 3 (mGluR3)	Grm3 Gprc1c Mglur3	108069	1.3021	0.31886	0.4964	0.5248	21.3076425	21.21661	21.332115	21.2115025
Q8BTG7	Protein NDRG4 (N-myc downstream-regulated gene 4 protein) (Protein Ndr4)	Ndrg4 Kiaa1180 Ndr4	234593	1.2988	0.31988	0.49501	0.5248	19.94443	19.8211425	19.978805	19.917205
P61750	ADP-ribosylation factor 4	Arf4	11843	1.294	0.32133	0.49305	0.52591	19.563965	19.44972	19.4701	19.7090075
P26369	Splicing factor U2AF 65 kDa subunit (U2 auxiliary factor 65 kDa subunit) (U2 snRNP auxiliary factor large subunit)	U2af2 U2af65	22185	1.2946	0.32115	0.49329	0.52591	19.9819475	19.9226825	19.85737	19.666815
Q9CRB9	MICOS complex subunit Mic19 (Coiled-coil-helix-coiled-coil-helix domain-containing protein 3)	Chchd3 Mic19	66075	1.2834	0.3246	0.48865	0.53069	22.1170375	22.036135	21.8939125	21.9698875
Q9CPP6	NADH dehydrogenase [ubiquinone] 1 alpha subcomplex subunit 5 (Complex I subunit B13) (Complex I-13kD-B) (CI-13kD-B) (NADH-ubiquinone oxidoreductase 13 kDa-B subunit)	Ndufa5	68202	1.279	0.32596	0.48684	0.53234	22.0812325	22.2650675	22.10478	22.1775225
P35762	CD81 antigen (26 kDa cell surface protein TAPA-1) (Target of the antiproliferative antibody 1) (CD antigen CD81)	Cd81 Tapa1	12520	1.2758	0.32695	0.48552	0.53339	23.9286725	24.114115	24.1383975	24.2617725
Q8CHC4	Synaptojanin-1 (EC 3.1.3.36) (Synaptic inositol 1,4,5-trisphosphate 5-phosphatase 1)	Synj1 Kiaa0910		1.274	0.32751	0.48477	0.53355	24.866285	24.799945	24.8258775	24.8340425
Q9WV60	Glycogen synthase kinase-3 beta (GSK-3 beta) (EC 2.7.11.26) (Serine/threonine-protein kinase GSK3B) (EC 2.7.11.1)	Gsk3b	56637	1.2727	0.32793	0.48422	0.53355	20.998755	20.8245925	20.78085	20.8562825
P62835	Ras-related protein Rap-1A (Ras-related protein Krev-1)	Rap1a Krev-1	109905	1.2721	0.3281	0.484	0.53355	23.2960925	23.1721375	23.17132	23.2174025
P16460	Argininosuccinate synthase (EC 6.3.4.5) (Citrulline--aspartate ligase)	Ass1 Ass	11898	1.2659	0.33005	0.48142	0.53615	20.55302	20.6629975	20.555865	20.7744275
O88712	C-terminal-binding protein 1 (CtBP1) (EC 1.1.1.-)	Ctbp1	13016	1.2629	0.33101	0.48016	0.53715	22.83454	22.80538	23.0634975	22.7987025
P56391	Cytochrome c oxidase subunit 6B1 (Cytochrome c oxidase subunit VIb isoform 1) (COX VIb-1)	Cox6b1 Cox6b	110323	1.2578	0.33261	0.47806	0.53865	24.46817	24.53623	24.55577	24.5528875
Q8BLF1	Neutral cholesterol ester hydrolase 1 (NCEH) (EC 3.1.1.-) (Arylacetamide deacetylase-like 1) (Chlorpyrifos oxon-binding protein) (CPO-BP)	Nceh1 Aadacl1 Kiaa1363	320024	1.2577	0.33264	0.47802	0.53865	21.0674225	21.1790725	21.2753175	21.155645
Q9ERD7	Tubulin beta-3 chain	Tubb3	22152	1.2524	0.33433	0.47583	0.53909	25.775325	25.936475	26.0162425	25.9202325
P68254	14-3-3 protein theta (14-3-3 protein tau)	Ywhaq	22630	1.253	0.33413	0.47608	0.53909	25.51108	25.3842675	25.423805	25.22819
Q921G7	Electron transfer flavoprotein-ubiquinone oxidoreductase, mitochondrial (ETF-QO) (ETF-ubiquinone oxidoreductase) (EC 1.5.5.1) (Electron-transferring-flavoprotein dehydrogenase) (ETF dehydrogenase)	Etfdh	66841	1.2542	0.33376	0.47656	0.53909	20.95832	20.8013	20.8023025	20.9125125
Q99JF8	PC4 and SFRS1-interacting protein (Lens epithelium-derived growth factor) (mLEDGF)	Psip1 Ledgf	101739	1.255	0.33349	0.47692	0.53909	20.67902	20.8855125	20.7036025	20.880505
Q9D710	Thioredoxin-related transmembrane protein 2 (Thioredoxin domain-containing protein 14)	Tmx2 Txndc14	66958	1.2485	0.33558	0.4742	0.53997	19.7200675	19.6921075	19.8991575	19.7335975
Q9EPW0	Inositol polyphosphate-4-phosphatase type I A (Inositol polyphosphate 4-phosphatase type I) (Inositol polyphosphate 4-phosphatase-1) (4-Ptase-1) (Type I inositol 3,4-bisphosphate 4-phosphatase) (EC 3.1.3.66)	Inpp4a	269180	1.2489	0.33547	0.47435	0.53997	20.2769825	20.3169325	20.42094	20.21008
Q64516	Glycerol kinase (GK) (Glycerokinase) (EC 2.7.1.30) (ATP:glycerol 3-phosphotransferase)	Gk Gyk	14933	1.2456	0.33649	0.47302	0.54087	20.98446	20.7473275	20.72639	20.817785
Q05920	Pyruvate carboxylase, mitochondrial (EC 6.4.1.1) (Pyruvic carboxylase) (PCB)	Pc Pcx		1.2435	0.33719	0.47212	0.54096	23.0652525	23.1597075	23.079525	23.1633025
Q9JJI8	60S ribosomal protein L38	Rpl38	67671	1.2432	0.33727	0.47203	0.54096	18.64062	18.7909775	18.89571	19.1098425
Q8BHZ0	Protein FAM49A	Fam49a D12Ertd553e	76820	1.2421	0.33765	0.47153	0.54096	20.2658975	20.4648675	20.5922375	20.397665

Q9QUH0	Glutaredoxin-1 (Thioltransferase-1) (TTase-1)	Glrx Glrx1 Grx Grx1	93692	1.2411	0.33797	0.47113	0.54096	19.8646425	19.5378725	19.7057575	19.3952275
O54829	Regulator of G-protein signaling 7 (RGS7)	Rgs7	24012	1.2371	0.33925	0.46949	0.54244	21.920415	21.8910425	22.1291925	22.0121975
P16858	Glyceraldehyde-3-phosphate dehydrogenase (GAPDH) (EC 1.2.1.12) (Peptidyl-cysteine S-nitrosylase GAPDH) (EC 2.6.99.-)	Gapdh Gapd	14433	1.2329	0.34061	0.46774	0.54366	29.09202	29.09202	29.1372475	28.9809975
Q9WUL7	ADP-ribosylation factor-like protein 3	Arl3	56350	1.231	0.34123	0.46696	0.54366	21.510675	21.353035	21.4491825	21.335555
Q9EQ80	NIF3-like protein 1	Nif3l1	65102	1.2314	0.34112	0.4671	0.54366	19.257295	19.4658525	19.3384875	19.3542925
Q8JZQ2	AFG3-like protein 2 (EC 3.4.24.-)	Afg3l2	69597	1.2304	0.34144	0.46669	0.54366	20.9494975	21.0446925	21.001335	20.76454
Q9R111	Guanine deaminase (Guanase) (Guanine aminase) (EC 3.5.4.3) (Guanine aminohydrolase) (GAH)	Gda	14544	1.2293	0.3418	0.46623	0.54367	24.17143	24.3054775	24.17576	24.0987475
P28738	Kinesin heavy chain isoform 5C (Kinesin heavy chain neuron-specific 2)	Kif5c Nkhc2	16574	1.226	0.34287	0.46487	0.54481	22.14231	22.1014025	22.2530775	22.2817075
P70349	Histidine triad nucleotide-binding protein 1 (EC 3.-.-.-) (Adenosine 5'-monophosphoramidase) (Protein kinase C inhibitor 1) (Protein kinase C-interacting protein 1) (PKCI-1)	Hint1 Hint Pkci Pkci1 Prkcnh1	15254	1.2243	0.34344	0.46414	0.54516	23.1953175	23.33044	23.1484075	23.34692
Q8QZS1	3-hydroxyisobutyryl-CoA hydrolase, mitochondrial (EC 3.1.2.4) (3-hydroxyisobutyryl-coenzyme A hydrolase) (HIB-CoA hydrolase) (HIBYL-CoA-H)	Hibch	227095	1.2206	0.34465	0.46263	0.54649	20.62926	20.730665	20.796795	20.80386
Q8JZQ9	Eukaryotic translation initiation factor 3 subunit B (eIF3b) (Eukaryotic translation initiation factor 3 subunit 9) (eIF-3-eta) (eIF3 p116)	Eif3b Eif3s9	27979	1.2188	0.34524	0.46187	0.54688	20.3575875	20.453655	20.2773675	20.367075
P97807	Fumarate hydratase, mitochondrial (Fumarase) (EC 4.2.1.2) (EF-3)	Fh Fh1	14194	1.2161	0.34612	0.46077	0.5477	23.825485	23.9657775	23.99611	23.9564825
Q9D394	Protein RUFY3 (Rap2-interacting protein x) (RIPx) (Single axon-regulated protein 1) (Singar1)	Rufy3 D5Bwg0860e Ripx	52822	1.209	0.34849	0.45781	0.55088	22.2524125	22.15583	22.1744275	22.0950975
Q9D4H8	Cullin-2 (CUL-2)	Cul2	71745	1.2053	0.34974	0.45626	0.55228	20.1224225	19.632575	19.969395	19.8899475
Q8VEH3	ADP-ribosylation factor-like protein 8A (ADP-ribosylation factor-like protein 10B) (Novel small G protein indispensable for equal chromosome segregation 2)	Arl8a Arl10b Gie2	68724	1.2038	0.35023	0.45565	0.55248	21.9067825	21.7886775	21.7729825	21.8985775
Q9R0P9	Ubiquitin carboxyl-terminal hydrolase isozyme L1 (UCH-L1) (EC 3.4.19.12) (Neuron cytoplasmic protein 9.5) (PGP 9.5) (PGP9.5) (Ubiquitin thioesterase L1)	Uchl1	22223	1.2025	0.35066	0.45511	0.55259	25.79481	25.814245	25.86065	25.95524
P12367	cAMP-dependent protein kinase type II-alpha regulatory subunit	Prkar2a		1.1984	0.35205	0.4534	0.55386	21.18895	21.129145	21.126585	21.0083525
P63168	Dynein light chain 1, cytoplasmic (8 kDa dynein light chain) (DLC8) (Dynein light chain LC8-type 1) (Protein inhibitor of neuronal nitric oxide synthase) (PIN) (mPIN)	Dynll1 Dlc1 Dncl1 Dnclc1	56455	1.198	0.35219	0.45322	0.55386	22.60013	22.4473825	22.7231775	22.504645
P62900	60S ribosomal protein L31	Rpl31	114641	1.1964	0.35274	0.45254	0.5539	20.248995	20.3190825	20.452465	20.4902725
Q9EPE9	Manganese-transporting ATPase 13A1 (CATP) (EC 7.2.2.-)	Atp13a1 Atp13a	170759	1.1958	0.35295	0.45229	0.5539	19.896645	19.969535	19.737	19.8779925
P62196	26S proteasome regulatory subunit 8 (26S proteasome AAA-ATPase subunit RPT6) (Proteasome 26S subunit ATPase 5) (Proteasome subunit p45) (p45/SUG) (mSUG1)	Psmc5 Sug1	19184	1.1946	0.35332	0.45183	0.55392	20.3831875	20.5526975	20.5467725	20.274835
P03888	NADH-ubiquinone oxidoreductase chain 1 (EC 7.1.1.2) (NADH dehydrogenase subunit 1)	Mtnd1 mt-Nd1 Nd1	17716 3338902	1.1909	0.3546	0.45026	0.55536	20.6335875	20.74776	20.90061	20.8115825
P05063	Fructose-bisphosphate aldolase C (EC 4.1.2.13) (Aldolase 3) (Brain-type aldolase) (Scrapie-responsive protein 2) (Zebrin II)	Aldoc Aldo3 Scrg2	11676	1.1883	0.35549	0.44918	0.55568	26.1612225	26.1097175	26.0103575	26.165375

Q148V7	RAB11-binding protein RELCH (LisH domain and HEAT repeat-containing protein KIAA1468) (RAB11-binding protein containing LisH, coiled-coil, and HEAT repeats)	Relch Kiaa1468	227446	1.1881	0.35553	0.44912	0.55568	19.517475	19.66283	19.784845	19.35526
O08749	Dihydrolipoyl dehydrogenase, mitochondrial (EC 1.8.1.4) (Dihydrolipoamide dehydrogenase)	Dld	13382	1.1831	0.35725	0.44703	0.55779	24.7349525	24.8021075	24.6834975	24.77343
P47754	F-actin-capping protein subunit alpha-2 (CapZ alpha-2)	Capza2 Cappa2	12343	1.179	0.35865	0.44533	0.55909	24.4751025	24.4679875	24.5015575	24.35974
O70325	Phospholipid hydroperoxide glutathione peroxidase (PHGPx) (EC 1.11.1.12) (Glutathione peroxidase 4) (GPx-4) (GSHPx-4)	Gpx4	625249	1.1786	0.35882	0.44513	0.55909	21.3823025	21.3248075	21.2259975	21.3801975
Q64727	Vinculin (Metavinculin)	Vcl	22330	1.1744	0.36025	0.4434	0.56075	20.134965	20.29566	20.3314925	20.4522325
Q91X97	Neurocalcin-delta	Ncald D15Ertd412e	52589	1.1703	0.36168	0.44168	0.56241	21.350305	21.29466	21.34312	21.259965
Q3U1J4	DNA damage-binding protein 1 (DDB p127 subunit) (Damage-specific DNA-binding protein 1) (UV-damaged DNA-binding factor)	Ddb1	13194	1.1656	0.36331	0.43972	0.5638	22.1554375	22.30917	22.0518475	22.2480925
Q9DBC7	cAMP-dependent protein kinase type I-alpha regulatory subunit [Cleaved into: cAMP-dependent protein kinase type I-alpha regulatory subunit, N-terminally processed]	Prkar1a	19084	1.1666	0.36295	0.44015	0.5638	21.18834	20.9944575	21.0392025	21.1371375
P63073	Eukaryotic translation initiation factor 4E (eIF-4E) (eIF4E) (mRNA cap-binding protein) (eIF-4F 25 kDa subunit)	Eif4e	13684	1.1637	0.36397	0.43893	0.56425	19.23991	19.1636275	19.0599825	19.2280725
Q99LD4	COP9 signalosome complex subunit 1 (SGN1) (Signalosome subunit 1) (G protein pathway suppressor 1) (GPS-1) (JAB1-containing signalosome subunit 1)	Gps1 Cops1 Csn1		1.1592	0.36554	0.43707	0.56572	21.1969425	21.069065	21.113105	21.119865
O70435	Proteasome subunit alpha type-3 (EC 3.4.25.1) (Macropain subunit C8) (Multicatalytic endopeptidase complex subunit C8) (Proteasome component C8) (Proteasome subunit K)	Psma3	19167	1.1567	0.36643	0.43601	0.56572	20.700075	20.5133025	20.6720275	20.5645925
O88685	26S proteasome regulatory subunit 6A (26S proteasome AAA-ATPase subunit RPT5) (Proteasome 26S subunit ATPase 3) (Tat-binding protein 1) (TBP-1)	Psmc3 Tbp1	19182	1.1557	0.36677	0.4356	0.56572	21.6351625	21.457915	21.602625	21.449415
Q9CYH2	Peroxiredoxin-like 2A (Peroxiredoxin-like 2 activated in M-CSF stimulated monocytes) (Protein PAMM) (Redox-regulatory protein FAM213A)	Prxl2a Fam213a Pamm	70564	1.1568	0.36638	0.43607	0.56572	21.102345	21.120625	20.919305	20.89711
Q3UUG6	TBC1 domain family member 24	Tbc1d24 Kiaa1171	224617	1.1562	0.36659	0.43582	0.56572	20.16928	20.5269375	20.54172	20.4897625
Q6P5E6	ADP-ribosylation factor-binding protein GGA2 (Gamma-adaptin-related protein 2) (Golgi-localized, gamma ear-containing, ARF-binding protein 2)	Gga2 Kiaa1080	74105	1.1536	0.36751	0.43473	0.56628	20.05782	19.7591825	20.180435	19.914245
Q8VIJ6	Splicing factor, proline- and glutamine-rich (DNA-binding p52/p100 complex, 100 kDa subunit) (Polypyrimidine tract-binding protein-associated-splicing factor) (PSF) (PTB-associated-splicing factor)	Sfpq Psf	71514	1.1506	0.36856	0.43349	0.56727	22.3018425	22.2074225	22.2700725	22.342425
Q8BH59	Calcium-binding mitochondrial carrier protein Aralar1 (Mitochondrial aspartate glutamate carrier 1) (Solute carrier family 25 member 12)	Slc25a12 Aralar1	78830	1.1497	0.36889	0.4331	0.56727	25.500925	25.5701375	25.5223825	25.57805
O08795	Glucosidase 2 subunit beta (80K-H protein) (Glucosidase II subunit beta) (Protein kinase C substrate 60.1 kDa protein heavy chain) (PKCSH)	Prkcsh	19089	1.1484	0.36937	0.43254	0.56744	20.0462725	19.901865	19.8363575	20.02782
P08003	Protein disulfide-isomerase A4 (EC 5.3.4.1) (Endoplasmic reticulum resident protein 72) (ER protein 72) (ERp-72) (ERp72)	Pdia4 Cai Erp72	12304	1.142	0.37164	0.42988	0.56872	20.0021025	19.9180675	19.83524	19.9670775

Q60875	Rho guanine nucleotide exchange factor 2 (Guanine nucleotide exchange factor H1) (GEF-H1) (LBC'S first cousin) (Lymphoid blast crisis-like 1) (Oncogene LFC) (Rhobin)	Arhgef2 Kiaa0651 Lbcl1 Lfc	16800	1.1418	0.3717	0.42981	0.56872	20.7525875	20.8256725	20.8785775	20.718825
Q62048	Astrocytic phosphoprotein PEA-15 (15 kDa phosphoprotein enriched in astrocytes)	Pea15 Pea15a	18611	1.1404	0.37221	0.42921	0.56872	23.0103025	23.2914275	23.1720675	23.2399475
Q60829	Protein phosphatase 1 regulatory subunit 1B (DARPP-32) (Dopamine- and cAMP-regulated neuronal phosphoprotein)	Ppp1r1b	19049	1.1421	0.37161	0.42991	0.56872	19.1447925	19.2639675	19.4556225	19.3524925
Q99L13	3-hydroxyisobutyrate dehydrogenase, mitochondrial (HIBADH) (EC 1.1.1.31)	Hibadh	58875	1.1398	0.37244	0.42894	0.56872	20.96778	20.8879225	21.0834475	20.99841
Q7TMF3	NADH dehydrogenase [ubiquinone] 1 alpha subcomplex subunit 12 (Complex I-B17.2) (CI-B17.2) (CIB17.2) (NADH-ubiquinone oxidoreductase subunit B17.2)	Ndufa12	66414	1.1414	0.37187	0.42961	0.56872	22.42363	22.3020375	22.4087925	22.3642475
O88737	Protein bassoon	Bsn Kiaa0434	12217	1.1353	0.37403	0.4271	0.56886	24.0141775	24.0784275	23.9184325	23.9579025
P01837	Immunoglobulin kappa constant (Ig kappa chain C region MOPC 21)	Igkc	0	1.1354	0.37401	0.42712	0.56886	18.15712	16.151009	10.56355675	13.78873625
Q61704	Inter-alpha-trypsin inhibitor heavy chain H3 (ITI heavy chain H3) (ITI-HC3) (Inter-alpha-inhibitor heavy chain 3)	Itih3	16426	1.1367	0.37355	0.42765	0.56886	14.83238075	19.3819875	17.68084525	17.8953655
Q4KML4	Costars family protein ABRACL (ABRA C-terminal-like protein)	Abracl	73112	1.1363	0.37368	0.4275	0.56886	18.49374925	14.7437995	18.5827745	18.096093
Q9CPQ1	Cytochrome c oxidase subunit 6C (Cytochrome c oxidase polypeptide VIc)	Cox6c	12864	1.1332	0.37479	0.42621	0.56915	23.9462625	23.8199	23.746265	23.92377
Q9JKY5	Huntingtin-interacting protein 1-related protein (HIP1-related protein)	Hip1r	29816	1.1327	0.37496	0.42601	0.56915	19.8720875	19.71305	19.7175175	19.8173875
Q8BIJ6	Isoleucine--tRNA ligase, mitochondrial (EC 6.1.1.5) (Isoleucyl-tRNA synthetase) (IleRS)	Iars2	381314	1.1297	0.37606	0.42475	0.57025	20.3788375	20.4102925	20.2462525	20.40329
P14824	Annexin A6 (67 kDa calelectrin) (Annexin VI) (Annexin-6) (Calphobindin-II) (CPB-II) (Chromobindin-20) (Lipocortin VI) (Protein III) (p68) (p70)	Anxa6 Anx6	11749	1.1218	0.37895	0.42142	0.57354	23.32136	23.3269475	23.44159	23.423845
Q60931	Voltage-dependent anion-selective channel protein 3 (VDAC-3) (mVDAC3) (Outer mitochondrial membrane protein porin 3)	Vdac3	22335	1.1186	0.38009	0.42011	0.57354	24.7724775	24.8775225	24.7944125	24.8576425
Q76MZ3	Serine/threonine-protein phosphatase 2A 65 kDa regulatory subunit A alpha isoform (PP2A subunit A isoform PR65-alpha) (PP2A subunit A isoform R1-alpha)	Ppp2r1a	51792	1.1193	0.37983	0.42042	0.57354	25.1236925	25.04896	25.1007525	25.0914375
Q99LC3	NADH dehydrogenase [ubiquinone] 1 alpha subcomplex subunit 10, mitochondrial (Complex I-42kD) (CI-42kD) (NADH-ubiquinone oxidoreductase 42 kDa subunit)	Ndufa10	67273	1.1206	0.37937	0.42093	0.57354	23.9872575	24.1072075	24.1416475	24.0093075
Q8CCT4	Transcription elongation factor A protein-like 5 (TCEA-like protein 5) (Transcription elongation factor S-II protein-like 5)	Tceal5	331532	1.1186	0.38011	0.4201	0.57354	22.103205	21.889405	21.8658725	21.694385
Q99J99	3-mercaptopyruvate sulfurtransferase (MST) (EC 2.8.1.2)	Mpst	246221	1.1153	0.38131	0.41873	0.57478	20.1476275	20.422845	20.26572	20.4125325
Q8R081	Heterogeneous nuclear ribonucleoprotein L (hnRNP L)	Hnrnpl Hnrpl	15388	1.1087	0.38375	0.41595	0.57595	23.3047	23.2750425	23.3463875	23.3213775
P27546	Microtubule-associated protein 4 (MAP-4)	Map4 Mtap4	17758	1.1071	0.38434	0.41528	0.57595	22.48569	22.3722375	22.5267325	22.477995
Q9CXS4	Centromere protein V (CENP-V) (Proline-rich protein 6)	Cenpv Prr6	73139	1.112	0.38252	0.41735	0.57595	20.533675	20.4495775	20.493155	20.33128
Q9EQ20	Methylmalonate-semialdehyde dehydrogenase [acylating], mitochondrial (MMSDH) (Malonate-semialdehyde dehydrogenase [acylating]) (EC 1.2.1.18) (EC 1.2.1.27) (Aldehyde dehydrogenase family 6 member A1)	Aldh6a1	104776	1.1076	0.38414	0.41551	0.57595	21.483815	21.53408	21.393575	21.54127

Q80TB8	Synaptic vesicle membrane protein VAT-1 homolog-like (EC 1.-.-.-)	Vat1l Kiaa1576	270097	1.1075	0.38418	0.41546	0.57595	19.78163	20.23059	20.13591	19.861815
P62830	60S ribosomal protein L23	Rpl23	100044627 100862455 65019	1.109	0.38362	0.41609	0.57595	21.230155	21.2564	21.28652	21.4108325
Q9D358	Low molecular weight phosphotyrosine protein phosphatase (LMW-PTP) (LMW-PTPase) (EC 3.1.3.48) (Low molecular weight cytosolic acid phosphatase) (EC 3.1.3.2)	Acp1	11431	1.0991	0.38729	0.41196	0.5798	21.213215	20.9739475	21.1050625	20.8718725
Q922D8	C-1-tetrahydrofolate synthase, cytoplasmic (C1-THF synthase) [Cleaved into: C-1-tetrahydrofolate synthase, cytoplasmic, N-terminally processed] [Includes: Methylenetetrahydrofolate dehydrogenase (EC 1.5.1.5); Methenyltetrahydrofolate cyclohydrolase (EC 3.5.4.9); Formyltetrahydrofolate synthetase (EC 6.3.4.3)]	Mthfd1	108156	1.0945	0.38902	0.41002	0.58182	19.377225	19.1994425	19.2486425	19.51917
P70296	Phosphatidylethanolamine-binding protein 1 (PEBP-1) (HCNPpp) [Cleaved into: Hippocampal cholinergic neurostimulating peptide (HCNP)]	Pebp1 Pbp Pebp	23980	1.0933	0.38947	0.40953	0.58191	26.0013675	25.976615	25.96912	25.8925525
Q8VDN2	Sodium/potassium-transporting ATPase subunit alpha-1 (Na(+)/K(+) ATPase alpha-1 subunit) (EC 7.2.2.13) (Sodium pump subunit alpha-1)	Atp1a1	11928	1.0873	0.39174	0.407	0.58267	25.594275	25.6114725	25.6019175	25.70485
Q61735	Leukocyte surface antigen CD47 (Integrin-associated protein) (IAP) (CD antigen CD47)	Cd47	16423	1.0858	0.39231	0.40637	0.58267	22.536205	22.4942475	22.6161925	22.4513875
Q9CPQ8	ATP synthase subunit g, mitochondrial (ATPase subunit g) (ATP synthase membrane subunit g)	Atp5mg Atp5l	27425	1.0843	0.39287	0.40575	0.58267	23.242895	23.3701875	23.237175	23.385705
Q9Z1G3	V-type proton ATPase subunit C 1 (V-ATPase subunit C 1) (Vacuolar proton pump subunit C 1)	Atp6v1c1 Atp6c Atp6c1 Vatc	66335	1.0866	0.39202	0.40669	0.58267	23.901225	23.8236875	23.8393	23.7832625
Q8BK30	NADH dehydrogenase [ubiquinone] flavoprotein 3, mitochondrial (Complex I-9kD) (CI-9kD) (NADH-ubiquinone oxidoreductase 9 kDa subunit)	Ndufv3	78330	1.0901	0.3907	0.40816	0.58267	19.45018	19.2267325	19.230475	19.27855
Q3UHB1	5'-nucleotidase domain-containing protein 3 (EC 3.1.3.-) (GRP94-neighboring nucleotidase)	Nt5dc3 Gnn	103466	1.0884	0.39134	0.40745	0.58267	22.41032	22.2951725	22.46324	22.3393775
Q923D2	Flavin reductase (NADPH) (FR) (EC 1.5.1.30) (Biliverdin reductase B) (BVR-B) (EC 1.3.1.24) (Biliverdin-IX beta-reductase) (NADPH-dependent diaphorase) (NADPH-flavin reductase) (FLR)	Blvrb	233016	1.0845	0.39278	0.40585	0.58267	18.905234	14.8538165	19.1414085	14.46362125
Q6A065	Centrosomal protein of 170 kDa (Cep170)	Cep170 Kiaa0470	545389	1.0839	0.39303	0.40558	0.58267	21.1453575	21.310225	21.06074	21.244435
P61255	60S ribosomal protein L26 (Silica-induced gene 20 protein) (SIG-20)	Rpl26	19941	1.0825	0.39357	0.40498	0.5829	21.3779925	21.331895	21.2100425	21.3650325
Q9Z2I9	Succinate--CoA ligase [ADP-forming] subunit beta, mitochondrial (EC 6.2.1.5) (ATP-specific succinyl-CoA synthetase subunit beta) (A-SCS) (Succinyl-CoA synthetase beta-A chain) (SCS-betaA)	Sucla2	20916	1.0785	0.39507	0.40333	0.58383	24.7843125	24.74374	24.64538	24.79295
P60840	Alpha-endosulfine (ARPP-19e)	Ensa	56205	1.0782	0.39519	0.4032	0.58383	21.2011625	21.06263	21.08001	21.06939
Q922J6	Tetraspanin-2 (Tspan-2)	Tspan2	70747	1.0778	0.39534	0.40303	0.58383	21.4299025	21.34596	20.9927175	21.16662
Q99020	Heterogeneous nuclear ribonucleoprotein A/B (hnRNP A/B) (CArG-binding factor-A) (CBF-A)	Hnrnpab Cbf-a Cgbfa Hnrpab	15384	1.0727	0.39731	0.40087	0.58393	21.298715	21.38199	21.38354	21.38734
Q61885	Myelin-oligodendrocyte glycoprotein	Mog	17441	1.0754	0.39626	0.40202	0.58393	24.334765	24.2538675	24.069825	24.49384
Q62425	Cytochrome c oxidase subunit NDUFA4	Ndufa4	17992	1.0719	0.39759	0.40056	0.58393	25.1378525	25.0726575	25.058705	25.11277
Q8BP67	60S ribosomal protein L24	Rpl24	68193	1.0732	0.39709	0.40111	0.58393	21.9756975	21.83409	21.9751625	21.96255

O55131	Septin-7 (CDC10 protein homolog)	Septin7 Cdc10 Sept7	235072	1.0729	0.39722	0.40097	0.58393	25.123785	25.2283725	25.17881	25.150545
P84228	Histone H3.2	Hist1h3b H3-53 H3.2 H3b; Hist1h3c H3-143; Hist1h3d H3-B; Hist1h3e H3-F; Hist1h3f H3.2-221 H3f; Hist2h3b H3.2-616; Hist2h3c1 H3.2-615 Hist2h3ca1; Hist2h3c2 H3.2-614 Hist2h3ca2	15077 260423 319148 319149 319150 319151 319154 97114	1.0716	0.3977	0.40044	0.58393	15.46430825	19.01826525	19.59145725	19.11194175
Q9QXZ0	Microtubule-actin cross-linking factor 1 (Actin cross-linking family 7)	Macf1 Acf7 Aclp7 Macf		1.0676	0.39926	0.39875	0.58565	19.52196	19.696965	19.7196475	19.55479
Q9D3D9	ATP synthase subunit delta, mitochondrial (ATP synthase F1 subunit delta) (F-ATPase delta subunit)	Atp5f1d Atp5d	66043	1.066	0.39986	0.39809	0.58598	23.6876525	23.6478725	23.4826975	23.7206175
Q9WUM4	Coronin-1C (Coronin-3)	Coro1c	23790	1.0649	0.40028	0.39763	0.58604	22.862925	22.90886	22.9677325	22.9120125
P61514	60S ribosomal protein L37a	Rpl37a	19981	1.0635	0.40085	0.39702	0.5863	19.46279	19.2526925	19.357835	19.4474125
Q9WVE8	Protein kinase C and casein kinase substrate in neurons protein 2 (Syndapin-2) (Syndapin-II) (SdpII)	Pacsin2	23970	1.0603	0.40207	0.3957	0.58695	19.7858575	19.514355	19.612515	19.59522
P62754	40S ribosomal protein S6 (Phosphoprotein NP33)	Rps6	105244208 20104	1.0613	0.4017	0.39609	0.58695	22.624425	22.521785	22.52244	22.6285125
Q99PU5	Long-chain-fatty-acid--CoA ligase ACSBG1 (EC 6.2.1.3) (Acyl-CoA synthetase bubblegum family member 1) (mBG1) (Gonadotropin-regulated long chain acyl CoA synthetase) (GR-LACS) (Lipidosin)	Acsbg1 Kiaa0631 Lpd	94180	1.0584	0.4028	0.39491	0.58747	21.047495	21.106585	20.9787025	21.270315
Q9DCZ4	MICOS complex subunit Mic26 (Apolipoprotein O) (MICOS complex subunit Mic23) (Protein FAM121B)	Apoo Fam121b Mic23 Mic26	68316	1.0565	0.40355	0.39411	0.58799	20.8876125	20.7970875	20.6927225	20.7978375
P57780	Alpha-actinin-4 (Non-muscle alpha-actinin 4)	Actn4	60595	1.0543	0.40442	0.39317	0.5887	22.746765	22.762345	22.86892	22.784025
G5E8K5	Ankyrin-3 (ANK-3) (Ankyrin-G)	Ank3	11735	1.0485	0.40668	0.39075	0.59087	21.0584	20.924725	20.9371125	20.9190775
Q99LP6	GrpE protein homolog 1, mitochondrial (Mt-GrpE#1)	Grpel1	17713	1.0487	0.4066	0.39083	0.59087	21.2146675	21.207535	21.25857	20.8603625
P12787	Cytochrome c oxidase subunit 5A, mitochondrial (Cytochrome c oxidase polypeptide Va)	Cox5a	12858	1.0428	0.40895	0.38833	0.59306	24.84574	24.766575	24.8315625	24.878745
Q9JHI5	Isovaleryl-CoA dehydrogenase, mitochondrial (IVD) (EC 1.3.8.4)	Ivd	56357	1.0427	0.40899	0.38829	0.59306	21.4823775	21.5682225	21.3952625	21.426325
Q8K1J6	CCA tRNA nucleotidyltransferase 1, mitochondrial (EC 2.7.7.72) (mitochondrial tRNA nucleotidyl transferase, CCA-adding) (mt CCA-adding enzyme) (mt tRNA CCA-diphosphorylase) (mt tRNA CCA-pyrophosphorylase) (mt tRNA adenylyltransferase)	Trnt1	70047	1.0408	0.40974	0.38749	0.59306	21.29853	20.9289025	20.6992825	20.7441675
Q8BH44	Coronin-2B	Coro2b	235431	1.0408	0.40973	0.3875	0.59306	21.620195	21.685505	21.7585125	21.6181775
P63001	Ras-related C3 botulinum toxin substrate 1 (EC 3.6.5.2) (p21-Rac1)	Rac1	19353	1.0379	0.41088	0.38629	0.59358	24.3004275	24.241425	24.143735	24.3339
Q9EPJ9	ADP-ribosylation factor GTPase-activating protein 1 (ARF GAP 1) (ADP-ribosylation factor 1 GTPase-activating protein) (ARF1 GAP) (ARF1-directed GTPase-activating protein)	Arfgap1 Arf1gap	228998	1.0381	0.41078	0.38639	0.59358	20.824865	20.8019125	20.906985	20.749675
P50114	Protein S100-B (S-100 protein beta chain) (S-100 protein subunit beta) (S100 calcium-binding protein B)	S100b	20203	1.0353	0.41192	0.38518	0.59452	20.5465725	21.4978375	21.3106625	21.5442825

O55142	60S ribosomal protein L35a	Rpl35a	57808	1.0339	0.41246	0.38462	0.59473	19.72437	19.473775	19.5580425	19.5972475
P08249	Malate dehydrogenase, mitochondrial (EC 1.1.1.37)	Mdh2 Mor1	17448	1.0299	0.41406	0.38294	0.59549	28.0733975	28.106245	28.0823325	28.0410225
P23492	Purine nucleoside phosphorylase (PNP) (EC 2.4.2.1) (Inosine phosphorylase) (Inosine-guanosine phosphorylase)	Pnp Np Pnp1	18950	1.0309	0.41367	0.38334	0.59549	20.0350175	20.1745325	20.1042325	20.407325
P46097	Synaptotagmin-2 (Inositol polyphosphate-binding protein) (IP4-binding protein) (IP4BP) (Synaptotagmin II) (SytII)	Syt2	20980	1.0287	0.41454	0.38243	0.59549	19.41043	19.28937	19.12674	19.5788225
Q9JJK2	LanC-like protein 2 (Testis-specific adriamycin sensitivity protein)	Lancl2 Tasp	71835	1.0287	0.41454	0.38243	0.59549	22.3725525	22.4454225	22.564285	22.3422925
Q62059	Versican core protein (Chondroitin sulfate proteoglycan core protein 2) (Chondroitin sulfate proteoglycan 2) (Large fibroblast proteoglycan) (PG-M)	Vcan Cspg2		1.0266	0.41539	0.38154	0.59597	20.399755	20.3781425	20.3593175	20.6580825
P10637	Microtubule-associated protein tau (Neurofibrillary tangle protein) (Paired helical filament-tau) (PHF-tau)	Mapt Mtapt Tau	17762	1.0245	0.41625	0.38064	0.59597	24.7581525	24.80313	24.81288	24.634965
P61759	Prefoldin subunit 3 (von Hippel-Lindau-binding protein 1) (VBP-1) (VHL-binding protein 1)	Vbp1 Pfdn3	22327	1.0253	0.41592	0.38099	0.59597	19.97303	19.86167	19.9731075	19.85377
Q8CGY8	UDP-N-acetylglucosamine--peptide N-acetylglucosaminyltransferase 110 kDa subunit (EC 2.4.1.255) (O-GlcNAc transferase subunit p110) (O-linked N-acetylglucosamine transferase 110 kDa subunit) (OGT)	Ogt	108155	1.024	0.41644	0.38045	0.59597	20.8419575	20.84559	20.8300675	20.6908525
P14211	Calreticulin (CRP55) (Calregulin) (Endoplasmic reticulum resident protein 60) (ERp60) (HACBP)	Calr	12317	1.0212	0.41758	0.37926	0.59649	23.5828125	23.78119	23.78236	23.88635
O70251	Elongation factor 1-beta (EF-1-beta)	Eef1b Eef1b2	55949	1.0217	0.41738	0.37947	0.59649	22.2119425	22.261465	22.3450175	22.3000425
P24288	Branched-chain-amino-acid aminotransferase, cytosolic (BCAT(c)) (EC 2.6.1.42) (Protein ECA39)	Bcat1 Eca39	12035	1.0193	0.41833	0.37848	0.597	20.9451225	20.354895	20.3730225	20.3950625
Q61646	Haptoglobin [Cleaved into: Haptoglobin alpha chain; Haptoglobin beta chain]	Hp	15439	1.0165	0.41947	0.3773	0.59807	14.36464025	19.7228825	13.47394675	14.0737965
O35526	Syntaxin-1A (Neuron-specific antigen HPC-1)	Stx1a	20907	1.0135	0.42069	0.37603	0.59821	23.37482	23.38054	23.598255	23.2790775
Q8CGC6	RNA-binding protein 28 (RNA-binding motif protein 28)	Rbm28	68272	1.0134	0.42075	0.37598	0.59821	21.2431175	21.1799225	21.1758225	20.3807125
Q920E5	Farnesyl pyrophosphate synthase (FPP synthase) (FPS) (EC 2.5.1.10) ((2E,6E)-farnesyl diphosphate synthase) (Cholesterol-regulated 39 kDa protein) (CR 39) (Dimethylallyltranstransferase) (EC 2.5.1.1) (Farnesyl diphosphate synthase) (Geranyltranstransferase)	Fdps	110196	1.0138	0.42058	0.37615	0.59821	20.097365	19.78059	19.9724125	19.7693325
Q99MR8	Methylcrotonoyl-CoA carboxylase subunit alpha, mitochondrial (MCCase subunit alpha) (EC 6.4.1.4) (3-methylcrotonyl-CoA carboxylase 1) (3-methylcrotonyl-CoA carboxylase biotin-containing subunit) (3-methylcrotonyl-CoA:carbon dioxide ligase subunit alpha)	Mccc1 Mcca	72039	1.0123	0.42119	0.37552	0.59829	18.9041825	19.0356375	18.6141475	18.814405
P43006	Excitatory amino acid transporter 2 (GLT-1) (Sodium-dependent glutamate/aspartate transporter 2) (Solute carrier family 1 member 2)	Slc1a2 Eaat2 Glt1	20511	1.0101	0.42206	0.37463	0.59841	27.0285775	27.002015	26.9270725	26.892395
Q7M750	Opalin (Oligodendrocytic myelin paranodal and inner loop protein) (Transmembrane protein 10)	Opalin Tmem10	226115	1.0105	0.4219	0.37479	0.59841	19.6502325	19.6298575	19.344	19.628445
P55088	Aquaporin-4 (AQP-4) (Mercurial-insensitive water channel) (MIWC) (WCH4)	Aqp4	11829	1.0008	0.42588	0.37071	0.59931	20.91935	21.4939075	21.23748	21.60852
O55100	Synaptogyrin-1	Syngr1	20972	1.0021	0.42534	0.37126	0.59931	23.505215	23.3461975	23.4499825	23.3511175

Q9JMG7	Hepatoma-derived growth factor-related protein 3 (HRP-3)	Hdgfl3 Hdgfrp3	29877	1.0043	0.42447	0.37215	0.59931	21.432805	21.387525	21.3504425	21.51461
O70493	Sorting nexin-12 (SDP8 protein)	Snx12	55988	1.0009	0.42583	0.37076	0.59931	20.82029	20.8443725	20.9227675	20.83675
Q91VN4	MICOS complex subunit Mic25 (Coiled-coil-helix-coiled-coil-helix domain-containing protein 6)	Chchd6 Mic25	66098	0.99908	0.4266	0.36998	0.59931	20.85183	20.707225	20.6900125	20.64766
Q99JY8	Phospholipid phosphatase 3 (EC 3.1.3.-) (EC 3.1.3.4) (Lipid phosphate phosphohydrolase 3) (PAP2-beta) (Phosphatidate phosphohydrolase type 2b) (Phosphatidic acid phosphatase 2b) (PAP-2b) (PAP2b)	Plpp3 Lpp3 Ppap2b	67916	0.99904	0.42662	0.36996	0.59931	21.5177575	21.66535	21.6172	21.626445
Q8BVI4	Dihydropteridine reductase (EC 1.5.1.34) (HDHPR) (Quinoid dihydropteridine reductase)	Qdpr Dhpr	110391	1.0028	0.42508	0.37153	0.59931	23.2019025	22.91631	23.071575	23.064395
P58389	Serine/threonine-protein phosphatase 2A activator (EC 5.2.1.8) (PP2A, subunit B', PR53 isoform) (Phosphotyrosyl phosphatase activator) (PTPA) (Serine/threonine-protein phosphatase 2A regulatory subunit 4) (Serine/threonine-protein phosphatase 2A regulatory subunit B')	Ptpa Ppp2r4	110854	1.0059	0.4238	0.37284	0.59931	22.03367	22.162825	22.010925	22.0435
Q6PIC6	Sodium/potassium-transporting ATPase subunit alpha-3 (Na(+)/K(+) ATPase alpha-3 subunit) (EC 7.2.2.13) (Na(+)/K(+) ATPase alpha(III) subunit) (Sodium pump subunit alpha-3)	Atp1a3	232975	1	0.42622	0.37036	0.59931	29.27293	29.27293	29.2277025	29.27293
P47963	60S ribosomal protein L13 (A52)	Rpl13	270106	1.0002	0.42612	0.37047	0.59931	22.4802775	22.4159425	22.4764725	22.54808
Q91VR2	ATP synthase subunit gamma, mitochondrial (ATP synthase F1 subunit gamma) (F-ATPase gamma subunit)	Atp5f1c Atp5c1	11949	0.98974	0.43047	0.36605	0.60225	24.9687	24.9834875	24.916005	25.029095
P62482	Voltage-gated potassium channel subunit beta-2 (EC 1.1.1.-) (K(+) channel subunit beta-2) (Kv-beta-2) (Neuroimmune protein F5)	Kcnab2 Ckbeta2 I2rf5 Kcnb3	16498	0.99142	0.42978	0.36676	0.60225	22.2789425	22.2564575	22.3373025	22.079795
P07759	Serine protease inhibitor A3K (Serpin A3K) (Contrapsin) (SPI-2)	Serpina3k Mcm2 Spi2	20714	0.9914	0.42978	0.36675	0.60225	21.242445	22.3017725	20.940195	21.5865425
O88653	Ragulator complex protein LAMTOR3 (Late endosomal/lysosomal adaptor and MAPK and MTOR activator 3) (MEK-binding partner 1) (Mp1) (Mitogen-activated protein kinase kinase 1-interacting protein 1) (Mitogen-activated protein kinase scaffold protein 1)	Lamtor3 Map2k1ip1 Mapbp Mapksp1	56692	0.98995	0.43039	0.36614	0.60225	19.786865	19.8010625	20.0004925	19.96084
P17742	Peptidyl-prolyl cis-trans isomerase A (PPIase A) (EC 5.2.1.8) (Cyclophilin A) (Cyclosporin A-binding protein) (Rotamase A) (SP18) [Cleaved into: Peptidyl-prolyl cis-trans isomerase A, N-terminally processed]	Ppia	268373	0.98924	0.43069	0.36584	0.60225	27.4757775	27.41489	27.4757775	27.3742075
Q9DC70	NADH dehydrogenase [ubiquinone] iron-sulfur protein 7, mitochondrial (EC 1.6.99.3) (EC 7.1.1.2) (Complex I-20kD) (CI-20kD) (NADH-ubiquinone oxidoreductase 20 kDa subunit)	Ndufs7	75406	0.98545	0.43227	0.36425	0.60391	21.573325	21.8990375	21.752285	21.677655
P06745	Glucose-6-phosphate isomerase (GPI) (EC 5.3.1.9) (Autocrine motility factor) (AMF) (Neuroleukin) (NLK) (Phosphoglucose isomerase) (PGI) (Phosphohexose isomerase) (PHI)	Gpi Gpi1	14751	0.98204	0.4337	0.36281	0.60535	26.278475	26.32156	26.266815	26.2258625
P0DN34	NADH dehydrogenase [ubiquinone] 1 beta subcomplex subunit 1 (Complex I-MNLL) (CI-MNLL) (NADH-ubiquinone oxidoreductase MNLL subunit)	Ndufb1		0.97763	0.43555	0.36096	0.60739	20.66009	20.64009	20.4827625	20.63676
Q9QUM9	Proteasome subunit alpha type-6 (EC 3.4.25.1) (Macropain iota chain) (Multicatalytic endopeptidase complex iota chain) (Proteasome iota chain)	Psma6	26443	0.97629	0.43612	0.36039	0.60763	22.2852775	22.2115575	22.322775	22.465245

Q9EQU5	Protein SET (Phosphatase 2A inhibitor I2PP2A) (I-2PP2A) (Template-activating factor I) (TAF-I)	Set	56086	0.97305	0.43749	0.35903	0.60842	21.5034725	21.4839525	21.4291525	21.539205
P56959	RNA-binding protein FUS (Protein pigpen)	Fus	233908	0.97326	0.4374	0.35912	0.60842	21.2621925	21.176345	21.2353325	21.20557
Q99PJ0	Neurotrimin	Ntm Hnt Nt		0.9674	0.43989	0.35665	0.61094	22.6565975	22.550195	22.6709675	22.4349975
Q99J85	Neuronal pentraxin receptor	Nptxr Npr		0.96597	0.4405	0.35605	0.61094	21.063	21.4679325	21.400945	21.3274875
Q91Z31	Polypyrimidine tract-binding protein 2 (Brain-enriched polypyrimidine tract-binding protein) (Brain-enriched PTB) (Neural polypyrimidine tract-binding protein) (RRM-type RNA-binding protein brPTB)	Ptbp2 Brptb Nptb	56195	0.96659	0.44024	0.35631	0.61094	20.223255	20.20443	20.1896825	20.0858125
Q91WJ8	Far upstream element-binding protein 1 (FBP) (FUSE-binding protein 1)	Fubp1 D3Ertd330e		0.96335	0.44162	0.35495	0.61146	20.53657	20.5026925	20.323015	20.5577975
Q9Z0P4	Paralemmin-1 (Paralemmin)	Palm	18483	0.96322	0.44168	0.35489	0.61146	23.466505	23.4105725	23.3908225	23.3527025
P70188	Kinesin-associated protein 3 (KAP-3) (KAP3)	Kifap3	16579	0.96091	0.44267	0.35392	0.61227	18.3587875	18.638615	18.414455	18.2817725
P03930	ATP synthase protein 8 (A6L) (F-ATPase subunit 8)	Mtatp8 Atp8 mt-Atp8	17706	0.95773	0.44403	0.35259	0.61305	23.2402975	23.1502175	23.0723925	23.226995
P03899	NADH-ubiquinone oxidoreductase chain 3 (EC 7.1.1.2) (NADH dehydrogenase subunit 3)	Mtnd3 mt-Nd3 Nd3	17718 3338896	0.95824	0.44381	0.3528	0.61305	19.7903175	19.74534	19.62596	19.7254075
Q91V14	Solute carrier family 12 member 5 (Electroneutral potassium-chloride cotransporter 2) (K-Cl cotransporter 2) (mKCC2) (Neuronal K-Cl cotransporter)	Slc12a5 Kcc2 Kiaa1176	57138	0.95653	0.44455	0.35208	0.61322	24.32627	24.3289525	24.4051875	24.3096275
Q61831	Mitogen-activated protein kinase 10 (MAP kinase 10) (MAPK 10) (EC 2.7.11.24) (MAP kinase p49 3F12) (Stress-activated protein kinase JNK3) (c-Jun N-terminal kinase 3)	Mapk10 Jnk3 Prkm10 Serk2	26414	0.94957	0.44756	0.34915	0.61639	21.2243675	21.20586	21.2285375	21.0518775
Q0VBF8	Protein stum homolog	Stum	381310	0.94934	0.44766	0.34905	0.61639	22.7311975	22.830615	22.8051575	22.87683
Q9D517	1-acyl-sn-glycerol-3-phosphate acyltransferase gamma (EC 2.3.1.51) (1-acylglycerol-3-phosphate O-acyltransferase 3) (1-AGP acyltransferase 3) (1-AGPAT 3) (Lysophosphatidic acid acyltransferase gamma) (LPAAT-gamma)	Agpat3 Lpaat3	28169	0.94673	0.44879	0.34795	0.61739	20.14589	19.6765475	20.6569275	20.3942375
P28184	Metallothionein-3 (MT-3) (Growth inhibitory factor) (GIF) (Metallothionein-III) (MT-III)	Mt3	17751	0.94457	0.44973	0.34704	0.61757	22.08439	21.7504125	21.7440725	21.328805
Q91VR5	ATP-dependent RNA helicase DDX1 (EC 3.6.4.13) (DEAD box protein 1)	Ddx1	104721	0.94482	0.44963	0.34715	0.61757	20.75758	20.857045	21.03138	20.90433
Q9JKC6	Cell cycle exit and neuronal differentiation protein 1 (BM88 antigen)	Cend1 Bm88	57754	0.94357	0.45017	0.34662	0.61762	24.160435	24.02303	23.8873525	23.73319
Q60737	Casein kinase II subunit alpha (CK II alpha) (EC 2.7.11.1)	Csnk2a1 Ckiia	12995	0.94006	0.4517	0.34515	0.61861	20.78489	20.8988675	21.0592425	20.90891
Q9CPU0	Lactoylglutathione lyase (EC 4.4.1.5) (Aldoketomutase) (Glyoxalase I) (Glx I) (Ketone-aldehyde mutase) (Methylglyoxalase) (S-D-lactoylglutathione methylglyoxal lyase)	Glo1	109801	0.94037	0.45157	0.34528	0.61861	22.4267475	22.56477	22.5134025	22.5654325
Q9R1P3	Proteasome subunit beta type-2 (EC 3.4.25.1) (Macropain subunit C7-I) (Multicatalytic endopeptidase complex subunit C7-I) (Proteasome component C7-I)	Psmb2	26445	0.93911	0.45212	0.34475	0.61863	19.95601	19.78776	19.8633175	19.86746
P63158	High mobility group protein B1 (High mobility group protein 1) (HMG-1)	Hmgb1 Hmg-1 Hmg1	15289	0.93812	0.45255	0.34433	0.61866	18.717095	19.8710325	19.2070725	19.66011
O35685	Nuclear migration protein nudC (Nuclear distribution protein C homolog) (Silica-induced gene 92 protein) (SIG-92)	Nudc	18221	0.93591	0.45352	0.3434	0.61944	21.2713575	21.1326825	21.1690525	21.2735975

Q8BFZ3	Beta-actin-like protein 2 (Kappa-actin)	Actbl2	238880	0.93471	0.45405	0.34289	0.61961	20.17236	19.9627825	20.017515	19.9391525
Q9DB20	ATP synthase subunit O, mitochondrial (ATP synthase peripheral stalk subunit OSCP) (Oligomycin sensitivity conferral protein) (OSCP)	Atp5po Atp5o D12Wsu28e	28080	0.93321	0.45471	0.34226	0.61995	25.4023225	25.4447925	25.4216225	25.4629025
Q9CT10	Ran-binding protein 3 (RanBP3)	Ranbp3	71810	0.92979	0.45622	0.34082	0.62146	18.6862725	18.4947425	18.7410025	18.67572
Q8K4B0	Metastasis-associated protein MTA1	Mta1		0.92497	0.45836	0.33879	0.6216	20.90508	20.8948375	20.742445	20.3534325
Q06138	Calcium-binding protein 39 (MO25alpha) (Protein Mo25)	Cab39 Mo25	12283	0.92842	0.45683	0.34025	0.6216	20.40677	20.299835	20.45314	20.4538825
P13707	Glycerol-3-phosphate dehydrogenase [NAD(+)], cytoplasmic (GPD-C) (GPDH-C) (EC 1.1.1.8)	Gpd1 Gdc-1 Gdc1 Kiaa4010	14555	0.92726	0.45734	0.33976	0.6216	21.6080125	21.76244	21.68151	21.900575
P43276	Histone H1.5 (H1 VAR.5) (H1b)	H1-5 H1f5 Hist1h1b	56702	0.92561	0.45808	0.33906	0.6216	19.501745	19.283525	19.2126775	19.37554
P60521	Gamma-aminobutyric acid receptor-associated protein-like 2 (GABA(A) receptor-associated protein-like 2) (Golgi-associated ATPase enhancer of 16 kDa) (GATE-16)	Gabarapl2	93739	0.92539	0.45817	0.33897	0.6216	19.775435	19.6998925	19.8166125	19.706975
Q9JLZ3	Methylglutaconyl-CoA hydratase, mitochondrial (EC 4.2.1.18) (AU-specific RNA-binding enoyl-CoA hydratase) (AU-binding enoyl-CoA hydratase) (muAUH) (Itaconyl-CoA hydratase) (EC 4.2.1.56)	Auh	11992	0.921	0.46013	0.33712	0.62279	23.97337	23.95704	23.8308925	23.967065
P03921	NADH-ubiquinone oxidoreductase chain 5 (EC 7.1.1.2) (NADH dehydrogenase subunit 5)	Mtnd5 mt-Nd5 Nd5	17721	0.92025	0.46046	0.3368	0.62279	21.4375075	21.4352725	21.6790525	21.5659775
Q8CBY8	Dynactin subunit 4 (Dynactin subunit p62)	Dctn4	67665	0.92152	0.45989	0.33734	0.62279	19.65457	19.75725	19.816985	19.72955
Q61016	Guanine nucleotide-binding protein G(I)/G(S)/G(O) subunit gamma-7	Gng7 Gngt7		0.91642	0.46217	0.33519	0.62381	21.3109	21.263095	21.22739	21.1316825
Q9DBJ1	Phosphoglycerate mutase 1 (EC 5.4.2.11) (EC 5.4.2.4) (BPG-dependent PGAM 1) (Phosphoglycerate mutase isozyme B) (PGAM-B)	Pgam1	18648	0.91582	0.46244	0.33494	0.62381	26.14799	26.2272975	26.2714675	26.240385
P68510	14-3-3 protein eta	Ywhah	22629	0.91746	0.46171	0.33563	0.62381	26.054655	25.9389675	25.8825875	25.9652375
Q8C1A5	Thimet oligopeptidase (EC 3.4.24.15)	Thop1	50492	0.913	0.46371	0.33375	0.62442	21.09506	21.002865	21.0243325	21.17924
Q8CIE6	Coatomer subunit alpha (Alpha-coat protein) (Alpha-COP) [Cleaved into: Xenin (Xenopsin-related peptide); Proxenin]	Copa	12847	0.91368	0.4634	0.33404	0.62442	19.720535	19.4276175	19.40112	19.604475
P22892	AP-1 complex subunit gamma-1 (Adaptor protein complex AP-1 subunit gamma-1) (Adaptor-related protein complex 1 subunit gamma-1) (Clathrin assembly protein complex 1 gamma-1 large chain) (Gamma-adaptin) (Gamma1-adaptin) (Golgi adaptor HA1/AP1 adaptin subunit gamma-1)	Ap1g1 Adtg Clapg1	11765	0.91111	0.46456	0.33296	0.62501	20.61588	20.5712175	20.65317	20.49977
P16388	Potassium voltage-gated channel subfamily A member 1 (MBK1) (MKI) (Voltage-gated potassium channel subunit Kv1.1)	Kcna1	16485	0.90647	0.46666	0.331	0.6264	20.033975	19.962605	20.2265575	20.33787
Q8BYI9	Tenascin-R (TN-R) (Janusin) (Neural recognition molecule J1-160/180) (Restrictin)	Tnr	21960	0.90759	0.46615	0.33147	0.6264	25.1392	25.1112925	25.185985	25.14801
Q68FF6	ARF GTPase-activating protein GIT1 (ARF GAP GIT1) (G protein-coupled receptor kinase-interactor 1) (GRK-interacting protein 1)	Git1	216963	0.9061	0.46682	0.33085	0.6264	19.8444625	19.7212025	19.827125	19.5750325
Q8K595	Mucolipin-2 (Transient receptor potential channel mucolipin 2) (TRPML2)	Mcoln2	68279	0.90464	0.46749	0.33023	0.62674	20.69927	20.84381	20.54415	20.7010075
O70503	Very-long-chain 3-oxoacyl-CoA reductase (EC 1.1.1.330) (17-beta-hydroxysteroid dehydrogenase 12) (17-beta-HSD 12) (3-	Hsd17b12 Kik1	56348	0.90015	0.46953	0.32834	0.62892	20.1697525	20.133565	20.2475575	20.1270825

	ketoacyl-CoA reductase) (KAR) (Estradiol 17-beta-dehydrogenase 12) (EC 1.1.1.62) (KIK-I)											
Q8VBV7	COP9 signalosome complex subunit 8 (SGN8) (Signalosome subunit 8) (COP9 homolog) (JAB1-containing signalosome subunit 8)	Cops8 Csn8	108679	0.89871	0.47018	0.32773	0.62925	20.0707925	20.0906275	20.1935075	20.0702475
Q3UUI3	Acyl-coenzyme A thioesterase THEM4 (Acyl-CoA thioesterase THEM4) (EC 3.1.2.2) (Carboxyl-terminal modulator protein) (Thioesterase superfamily member 4)	Them4 Ctmp	75778	0.89665	0.47112	0.32687	0.62995	19.287905	19.278205	19.4423475	19.0794875
P62331	ADP-ribosylation factor 6	Arf6	11845	0.89516	0.4718	0.32624	0.63031	20.7431375	20.715045	20.80137	20.6599375
P30275	Creatine kinase U-type, mitochondrial (EC 2.7.3.2) (Acidic-type mitochondrial creatine kinase) (Mia-CK) (Ubiquitous mitochondrial creatine kinase) (U-MtCK)	Ckmt1	12716	0.89423	0.47223	0.32585	0.63033	25.2808175	25.3857475	25.3690225	25.3876
P62897	Cytochrome c, somatic	Cycs	13063	0.89224	0.47314	0.32501	0.63099	24.617115	24.6607475	24.75307	24.6779925
O35658	Complement component 1 Q subcomponent-binding protein, mitochondrial (GC1q-R protein) (Glycoprotein gC1qBP) (C1qBP)	C1qbp Gc1qbp		0.88913	0.47457	0.3237	0.63124	21.46639	21.6173575	21.60711	21.6309625
Q60597	2-oxoglutarate dehydrogenase, mitochondrial (EC 1.2.4.2) (2-oxoglutarate dehydrogenase complex component E1) (OGDC-E1) (Alpha-ketoglutarate dehydrogenase)	Ogdh Kiaa4192	18293	0.88919	0.47454	0.32372	0.63124	24.415805	24.3625975	24.4296625	24.4561175
Q8CHP8	Glycerol-3-phosphate phosphatase (G3PP) (EC 3.1.3.21) (Aspartate-based ubiquitous Mg(2+)-dependent phosphatase) (AUM) (EC 3.1.3.48) (Phosphoglycolate phosphatase) (PGP)	Pgp	67078	0.8905	0.47394	0.32427	0.63124	20.78084	20.62599	20.590885	20.7013575
F8VPU2	FERM, ARHGEF and pleckstrin domain-containing protein 1 (FERM, RhoGEF and pleckstrin domain-containing protein 1)	Farp1	223254	0.87918	0.47917	0.31951	0.63681	20.409545	20.67571	20.7605325	20.70523
Q80TL0	Protein phosphatase 1E (EC 3.1.3.16) (Ca(2+)/calmodulin-dependent protein kinase phosphatase N) (CaMKP-N) (CaMKP-nucleus) (CaMKN) (Partner of PIX 1) (Partner of PIX-alpha) (Partner of PIXA)	Ppm1e Camkn Kiaa1072	320472	0.87225	0.48241	0.31659	0.64055	22.29107	22.090715	22.1593175	22.1524075
Q920P5	Adenylate kinase isoenzyme 5 (AK 5) (EC 2.7.4.3) (EC 2.7.4.6) (ATP-AMP transphosphorylase 5)	Ak5	229949	0.86569	0.48548	0.31383	0.64408	20.9218875	21.00501	20.972655	20.7489775
Q7TQD2	Tubulin polymerization-promoting protein (TPPP) (EC 3.6.5.-) (25 kDa brain-specific protein) (TPPP/p25) (p25-alpha)	Tppp	72948	0.86251	0.48698	0.31249	0.6455	24.54301	24.561755	24.5942225	24.5098425
Q9ERS2	NADH dehydrogenase [ubiquinone] 1 alpha subcomplex subunit 13 (Cell death regulatory protein GRIM-19) (Complex I-B16.6) (CI-B16.6) (Gene associated with retinoic and interferon-induced mortality 19 protein) (GRIM-19) (Gene associated with retinoic and IFN-induced mortality 19 protein) (NADH-ubiquinone oxidoreductase B16.6 subunit)	Ndufa13 Grim19	67184	0.86123	0.48759	0.31195	0.64575	25.29036	25.23792	25.0251	25.2055125
O35593	26S proteasome non-ATPase regulatory subunit 14 (EC 3.4.19.-) (26S proteasome regulatory subunit RPN11) (MAD1)	Psmd14 Pad1	59029	0.86014	0.48811	0.31149	0.64587	20.12922	20.01873	20.205175	20.0413325
Q9QYR9	Acyl-coenzyme A thioesterase 2, mitochondrial (Acyl-CoA thioesterase 2) (EC 3.1.2.2) (Acyl coenzyme A thioester hydrolase) (MTE-I) (Very-long-chain acyl-CoA thioesterase)	Acot2 Mte1	171210	0.85844	0.48891	0.31077	0.64637	19.98116	20.07738	19.94396	20.1782475
Q8BHN3	Neutral alpha-glucosidase AB (EC 3.2.1.207) (Alpha-glucosidase 2) (Glucosidase II subunit alpha)	Ganab G2an Kiaa0088	14376	0.85651	0.48982	0.30996	0.64643	20.71484	20.7997975	20.5480025	20.60956
Q9CS84	Neurexin-1 (Neurexin I-alpha) (Neurexin-1-alpha)	Nrxn1 Kiaa0578	18189	0.85568	0.49022	0.30961	0.64643	20.470125	20.5262175	20.45249	20.449475
Q9QZ88	Vacuolar protein sorting-associated protein 29 (Vesicle protein sorting 29)	Vps29	56433	0.85698	0.4896	0.31016	0.64643	20.93515	20.9400175	20.7898075	20.97199

P16330	2',3'-cyclic-nucleotide 3'-phosphodiesterase (CNP) (CNPase) (EC 3.1.4.37)	Cnp Cnp1	12799	0.85451	0.49078	0.30912	0.64661	27.6698675	27.6984825	27.47191	27.7269675
Q9D4C9	Clavesin-1 (Retinaldehyde-binding protein 1-like 1)	Clvs1 Rlbp1l1	74438	0.85244	0.49176	0.30824	0.64735	21.535435	21.430875	21.4023875	21.4501225
P70404	Isocitrate dehydrogenase [NAD] subunit gamma 1, mitochondrial (Isocitric dehydrogenase subunit gamma) (NAD(+)-specific ICDH subunit gamma)	Idh3g	15929	0.84932	0.49325	0.30693	0.64847	23.76381	23.7704775	23.9008825	23.8071775
Q9Z1L5	Voltage-dependent calcium channel subunit alpha-2/delta-3 (Voltage-gated calcium channel subunit alpha-2/delta-3) [Cleaved into: Voltage-dependent calcium channel subunit alpha-2-3; Voltage-dependent calcium channel subunit delta-3]	Cacna2d3	12294	0.84888	0.49346	0.30674	0.64847	18.99839	19.118715	18.8642375	18.6454175
Q99JX3	Golgi reassembly-stacking protein 2 (GRS2) (Golgi reassembly-stacking protein of 55 kDa) (GRASP55)	Gorasp2	70231	0.84755	0.4941	0.30619	0.64874	18.96728	19.0597825	19.2268975	19.3143925
Q9WUC3	Lymphocyte antigen 6H (Ly-6H)	Ly6h	23934	0.84562	0.49502	0.30537	0.64923	23.0901275	22.9992875	22.8126	22.79941
Q9CQV8	14-3-3 protein beta/alpha (Protein kinase C inhibitor protein 1) (KCIP-1) [Cleaved into: 14-3-3 protein beta/alpha, N-terminally processed]	Ywhab	54401	0.84412	0.49575	0.30474	0.64923	25.53887	25.551235	25.4583275	25.467115
Q9JII6	Aldo-keto reductase family 1 member A1 (EC 1.1.1.2) (EC 1.1.1.33) (EC 1.1.1.372) (EC 1.1.1.54) (Alcohol dehydrogenase [NADP(+)]) (Aldehyde reductase) (Glucuronate reductase) (EC 1.1.1.19) (Glucuronolactone reductase) (EC 1.1.1.20)	Akr1a1 Akr1a4	58810	0.84446	0.49558	0.30489	0.64923	23.070595	23.0705825	23.0286575	23.0207025
Q60870	Receptor expression-enhancing protein 5 (GP106) (Polyposis locus protein 1 homolog) (Protein TB2 homolog)	Reep5 Dp1		0.83906	0.49818	0.30261	0.6518	21.2712275	21.2310825	21.2457225	21.0503475
Q9QXV0	ProSAAS (IA-4) (Proprotein convertase subtilisin/kexin type 1 inhibitor) (Proprotein convertase 1 inhibitor) (pro-SAAS) [Cleaved into: KEP; Big SAAS (b-SAAS); Little SAAS (l-SAAS); Big PEN-LEN (b-PEN-LEN) (SAAS CT(1-49)); PEN; PEN-20; PEN-19; Little LEN (l-LEN); Big LEN (b-LEN) (SAAS CT(25-40))]	Pcsk1n	30052	0.83829	0.49856	0.30228	0.6518	21.25952	21.42205	21.1779025	21.2781725
P84091	AP-2 complex subunit mu (AP-2 mu chain) (Adaptor protein complex AP-2 subunit mu) (Adaptor-related protein complex 2 subunit mu) (Clathrin assembly protein complex 2 mu medium chain) (Clathrin coat assembly protein AP50) (Clathrin coat-associated protein AP50) (Mu2-adaptin) (Plasma membrane adaptor AP-2 50 kDa protein)	Ap2m1 Clapm1	11773	0.83599	0.49967	0.30132	0.65269	25.07013	25.049045	25.10687	25.0302975
Q8CC35	Synaptopodin	Synpo Kiaa1029		0.83126	0.50196	0.29933	0.65345	22.076565	22.1173675	22.14237	21.93406
P61202	COP9 signalosome complex subunit 2 (SGN2) (Signalosome subunit 2) (Alien homolog) (JAB1-containing signalosome subunit 2) (Thyroid receptor-interacting protein 15) (TR-interacting protein 15) (TRIP-15)	Cops2 Csn2 Trip15	12848	0.83373	0.50077	0.30037	0.65345	20.291085	20.23045	20.3392775	20.228275
P12815	Programmed cell death protein 6 (ALG-257) (Apoptosis-linked gene 2 protein) (ALG-2) (PMP41)	Pdcd6 Alg2	18570	0.83174	0.50173	0.29953	0.65345	20.178425	20.516975	20.1498575	20.3884225
P21107	Tropomyosin alpha-3 chain (Gamma-tropomyosin) (Tropomyosin-3)	Tpm3 Tpm-5 Tpm5	59069	0.83195	0.50163	0.29962	0.65345	24.576385	24.54646	24.45661	24.51452
Q8BGH2	Sorting and assembly machinery component 50 homolog	Samm50	68653	0.83017	0.5025	0.29887	0.65359	21.70678	21.6515525	21.7422575	21.68109
Q9CZ13	Cytochrome b-c1 complex subunit 1, mitochondrial (Complex III subunit 1) (Core protein I) (Ubiquinol-cytochrome-c reductase complex core protein 1)	Uqcrc1	22273	0.8268	0.50414	0.29745	0.65517	25.43596	25.4699375	25.4447425	25.490875

P52503	NADH dehydrogenase [ubiquinone] iron-sulfur protein 6, mitochondrial (Complex I-13kD-A) (CI-13kD-A) (NADH-ubiquinone oxidoreductase 13 kDa-A subunit)	Ndufs6 Ip13	407785	0.82385	0.50558	0.29621	0.65648	22.59902	22.6147475	22.4711475	22.331805
P01027	Complement C3 (HSE-MSF) [Cleaved into: Complement C3 beta chain; C3-beta-c (C3bc); Complement C3 alpha chain; C3a anaphylatoxin; Acylation stimulating protein (ASP) (C3adesArg); Complement C3b alpha' chain; Complement C3c alpha' chain fragment 1; Complement C3dg fragment; Complement C3g fragment; Complement C3d fragment; Complement C3f fragment; Complement C3c alpha' chain fragment 2]	C3	12266	0.82141	0.50678	0.29518	0.65734	20.7128125	21.9178325	21.431605	21.451325
Q8VDP4	Cell cycle and apoptosis regulator protein 2 (Cell division cycle and apoptosis regulator protein 2)	Ccar2	219158	0.82076	0.5071	0.29491	0.65734	19.0300975	19.4104675	19.1050875	19.091255
P17156	Heat shock-related 70 kDa protein 2 (Heat shock protein 70.2)	Hspa2 Hcp70.2 Hsp70-2	15512	0.81554	0.50967	0.29271	0.65955	20.7021975	20.856935	20.6873725	20.8163775
O70194	Eukaryotic translation initiation factor 3 subunit D (eIF3d) (Eukaryotic translation initiation factor 3 subunit 7) (eIF-3-zeta) (eIF3 p66)	Eif3d Eif3s7	55944	0.81636	0.50927	0.29305	0.65955	19.925235	19.7947125	19.98742	19.9119
Q3UMR5	Calcium uniporter protein, mitochondrial	Mcu	215999	0.81419	0.51034	0.29214	0.65985	20.960995	20.9795025	21.011465	20.889075
P19536	Cytochrome c oxidase subunit 5B, mitochondrial (Cytochrome c oxidase polypeptide Vb)	Cox5b		0.81111	0.51187	0.29084	0.66057	23.61334	23.559585	23.5451675	23.4957375
P10922	Histone H1.0 (Histone H1') (Histone H1(0)) (MyD196) [Cleaved into: Histone H1.0, N-terminally processed]	H1-0 H1f0 H1fv	14958	0.80965	0.51259	0.29023	0.66057	23.2886	23.2673825	23.078135	23.1452675
Q9WUK2	Eukaryotic translation initiation factor 4H (eIF-4H) (Williams-Beuren syndrome chromosomal region 1 protein homolog)	Eif4h Wbscr1	22384	0.80959	0.51262	0.2902	0.66057	21.5700825	21.4538425	21.376825	21.266345
Q8VE47	Ubiquitin-like modifier-activating enzyme 5 (Ubiquitin-activating enzyme 5) (UFM1-activating enzyme) (Ubiquitin-activating enzyme E1 domain-containing protein 1)	Uba5 Ube1dc1	66663	0.81087	0.51198	0.29075	0.66057	19.7134775	19.8598825	20.0322475	19.61003
P21619	Lamin-B2	Lmnb2	16907	0.80663	0.51409	0.28896	0.6619	19.5919425	19.6874275	19.5478025	19.7533975
Q7TMK9	Heterogeneous nuclear ribonucleoprotein Q (hnRNP Q) (Glycine- and tyrosine-rich RNA-binding protein) (GRY-RBP) (NS1-associated protein 1) (Synaptotagmin-binding, cytoplasmic RNA-interacting protein) (pp68)	Syncrip Hnrpq Nsap1 Nsap1l	56403	0.80571	0.51455	0.28857	0.66193	21.765525	21.7556525	21.8475225	21.845035
O35136	Neural cell adhesion molecule 2 (N-CAM-2) (NCAM-2) (Neural cell adhesion molecule RB-8) (R4B12)	Ncam2 Ocam Rncam	17968	0.80482	0.515	0.2882	0.66195	21.26985	21.39821	21.4888225	21.3324275
Q9CPR4	60S ribosomal protein L17	Rpl17		0.79876	0.51803	0.28565	0.66529	18.2162175	18.531745	18.83735	19.4844425
Q61425	Hydroxyacyl-coenzyme A dehydrogenase, mitochondrial (HCDH) (EC 1.1.1.35) (Medium and short-chain L-3-hydroxyacyl-coenzyme A dehydrogenase) (Short-chain 3-hydroxyacyl-CoA dehydrogenase)	Hadh Hadhsc Mschad Schad	15107	0.79282	0.52101	0.28315	0.66856	20.023185	20.2352525	20.0902975	20.2764375
P36916	Guanine nucleotide-binding protein-like 1 (GTP-binding protein MMR1)	Gnl1 Gna-rs1 Mmr1	14670	0.78748	0.52371	0.28091	0.67146	19.6087975	19.5139575	19.5823325	19.5776775
Q91ZX7	Prolow-density lipoprotein receptor-related protein 1 (LRP-1) (Alpha-2-macroglobulin receptor) (A2MR) (CD antigen CD91) [Cleaved into: Low-density lipoprotein receptor-related protein 1 85 kDa subunit (LRP-85); Low-density lipoprotein receptor-related protein 1 515 kDa subunit (LRP-515); Low-density lipoprotein receptor-related protein 1 intracellular domain (LRPICD)]	Lrp1 A2mr	16971	0.77504	0.53006	0.27568	0.67732	21.0185575	21.008395	21.02482	21.159785

P60202	Myelin proteolipid protein (PLP) (Lipophilin)	Plp1 Plp	18823	0.7754	0.52988	0.27583	0.67732	28.4524675	28.4309975	28.3175475	28.601095
Q60932	Voltage-dependent anion-selective channel protein 1 (VDAC-1) (mVDAC1) (Outer mitochondrial membrane protein porin 1) (Plasmalemmal porin) (Voltage-dependent anion-selective channel protein 5) (VDAC-5) (mVDAC5)	Vdac1 Vdac5	22333	0.77643	0.52935	0.27626	0.67732	26.85251	26.9146	26.8753425	26.848635
Q91V24	ATP-binding cassette sub-family A member 7	Abca7	27403	0.77656	0.52928	0.27631	0.67732	19.5712125	19.319035	19.1812275	19.1353525
Q9CZ42	ATP-dependent (S)-NAD(P)H-hydrate dehydratase (EC 4.2.1.93) (ATP-dependent NAD(P)HX dehydratase) (Carbohydrate kinase domain-containing protein) (NAD(P)HX dehydratase)	Naxd Carkd	69225	0.77248	0.53138	0.2746	0.67844	20.9011225	20.92118	21.03276	20.9381575
P14873	Microtubule-associated protein 1B (MAP-1B) (MAP1(X)) (MAP1.2) [Cleaved into: MAP1B heavy chain; MAP1 light chain LC1]	Map1b Mtap1b Mtap5	17755	0.76987	0.53272	0.2735	0.67921	25.3634625	25.3597675	25.38659	25.4035325
Q9DBE8	Alpha-1,3/1,6-mannosyltransferase ALG2 (EC 2.4.1.132) (EC 2.4.1.257) (Asparagine-linked glycosylation protein 2 homolog) (GDP-Man:Man(1)GlcNAc(2)-PP-Dol alpha-1,3-mannosyltransferase) (GDP-Man:Man(1)GlcNAc(2)-PP-dolichol mannosyltransferase) (GDP-Man:Man(2)GlcNAc(2)-PP-Dol alpha-1,6-mannosyltransferase)	Alg2 MNCb-5081	56737	0.76957	0.53287	0.27338	0.67921	19.6448075	19.8226275	19.6869725	19.7290275
Q9CR61	NADH dehydrogenase [ubiquinone] 1 beta subcomplex subunit 7 (Complex I-B18) (CI-B18) (NADH-ubiquinone oxidoreductase B18 subunit)	Ndufb7	66916	0.76753	0.53392	0.27252	0.67999	21.318685	21.18548	21.2393075	21.2215875
Q91VR8	Protein BRICK1 (BRK1)	Brk1	101314	0.76661	0.5344	0.27213	0.68002	20.5394925	20.75746	20.550185	20.7659375
O08585	Clathrin light chain A (Lca)	Clta		0.76453	0.53548	0.27126	0.68083	23.831085	23.71901	23.616785	23.6695625
Q9D2G2	Dihydrolipoyllysine-residue succinyltransferase component of 2-oxoglutarate dehydrogenase complex, mitochondrial (EC 2.3.1.61) (2-oxoglutarate dehydrogenase complex component E2) (OGDC-E2) (Dihydrolipoamide succinyltransferase component of 2-oxoglutarate dehydrogenase complex) (E2K)	Dlst	78920	0.76344	0.53604	0.2708	0.68098	23.4030325	23.3159725	23.407065	23.3253125
P42669	Transcriptional activator protein Pur-alpha (Purine-rich single-stranded DNA-binding protein alpha)	Pura	19290	0.76047	0.53758	0.26955	0.68185	24.322375	24.3121125	24.3707725	24.3070475
O88342	WD repeat-containing protein 1 (Actin-interacting protein 1) (AIP1)	Wdr1	22388	0.76041	0.53762	0.26953	0.68185	24.2806775	24.3365675	24.26482	24.3164325
Q99KJ8	Dynactin subunit 2 (50 kDa dynein-associated polypeptide) (Dynactin complex 50 kDa subunit) (DCTN-50) (Growth cone membrane protein 23-48K) (GMP23-48K) (p50 dynamitin)	Dctn2	69654	0.75954	0.53807	0.26916	0.68185	22.3052975	22.3511375	22.394365	22.3538525
P20917	Myelin-associated glycoprotein (Siglec-4a)	Mag	17136	0.7577	0.53903	0.26839	0.68221	23.2576925	23.3152375	23.1980925	23.469125
P52480	Pyruvate kinase PKM (EC 2.7.1.40) (Pyruvate kinase muscle isozyme)	Pkm Pk3 Pkm2 Pykm	18746	0.75425	0.54083	0.26694	0.68221	27.85154	27.870845	27.8714575	27.8021225
P49722	Proteasome subunit alpha type-2 (EC 3.4.25.1) (Macropain subunit C3) (Multicatalytic endopeptidase complex subunit C3) (Proteasome component C3)	Psma2 Lmpc3	19166	0.75386	0.54103	0.26678	0.68221	21.20356	21.3443325	21.3506825	21.4137375
Q8C1B7	Septin-11	Septin11 D5Ertd606e Sept11	52398	0.7556	0.54012	0.26751	0.68221	24.24903	24.2989475	24.3679575	24.3269825
Q9CR21	Acyl carrier protein, mitochondrial (ACP) (CI-SDAP) (NADH-ubiquinone oxidoreductase 9.6 kDa subunit)	Ndufab1	70316	0.75476	0.54056	0.26716	0.68221	23.305945	23.2338775	23.3230175	23.2536075

O35841	Apoptosis inhibitor 5 (API-5) (AAC-11)	Api5	11800	0.75432	0.54079	0.26697	0.68221	18.6360325	17.98584	18.742875	18.17836
P10852	4F2 cell-surface antigen heavy chain (4F2hc) (Solute carrier family 3 member 2) (CD antigen CD98)	Slc3a2 Mdu1	17254	0.74478	0.5458	0.26297	0.68552	24.167005	24.12052	24.18389	24.1919075
Q9D1E6	Tubulin-folding cofactor B (Cytoskeleton-associated protein 1) (Cytoskeleton-associated protein CKAPI) (Tubulin-specific chaperone B)	Tbcb Ckap1	66411	0.74604	0.54514	0.26349	0.68552	23.25023	23.0851525	22.98152	23.1278825
B9EJ80	PDZ domain-containing protein 8	Pdzd8	107368	0.74632	0.54499	0.26361	0.68552	20.6627175	20.6226925	20.4314025	20.5546975
Q6P1B1	Xaa-Pro aminopeptidase 1 (EC 3.4.11.9) (Aminoacylproline aminopeptidase) (Cytosolic aminopeptidase P) (Soluble aminopeptidase P) (sAmp) (X-Pro aminopeptidase 1) (X-prolyl aminopeptidase 1, soluble)	Xpnpep1	170750	0.74459	0.5459	0.26288	0.68552	20.416635	20.4036625	20.217575	20.385305
Q9CZD3	Glycine--tRNA ligase (EC 6.1.1.14) (Diadenosine tetraphosphate synthetase) (Ap4A synthetase) (EC 2.7.7.-) (Glycyl-tRNA synthetase 1) (GlyRS)	Gars1 Gars	353172	0.74702	0.54462	0.26391	0.68552	20.4634425	20.28068	20.305855	20.382945
Q66JS6	Eukaryotic translation initiation factor 3 subunit J-B (eIF3j-B) (Eukaryotic translation initiation factor 3 subunit 1-B) (eIF-3-alpha-B) (eIF3 p35)	Eif3j2 Eif3s1-2 Gm9781	100042807	0.74235	0.54708	0.26195	0.68644	19.266985	19.16143	19.1034725	19.27629
Q3THK7	GMP synthase [glutamine-hydrolyzing] (EC 6.3.5.2) (GMP synthetase) (Glutamine amidotransferase)	Gmps	229363	0.74108	0.54776	0.26141	0.68672	21.4628025	21.4778075	21.555705	21.4401825
Q8BGD9	Eukaryotic translation initiation factor 4B (eIF-4B)	Eif4b	75705	0.73754	0.54963	0.25993	0.6885	20.2409225	20.2355975	20.153575	20.0358475
Q7TSJ2	Microtubule-associated protein 6 (MAP-6) (Stable tubule-only polypeptide) (STOP)	Map6 Mtap6	17760	0.73632	0.55028	0.25942	0.68875	24.8553125	24.7756425	24.7492225	24.8040775
P12382	ATP-dependent 6-phosphofructokinase, liver type (ATP-PFK) (PFK-L) (EC 2.7.1.11) (6-phosphofructokinase type B) (Phosphofructo-1-kinase isozyme B) (PFK-B) (Phosphohexokinase)	Pfkl Pfk-l Pfkb	18641	0.73057	0.55335	0.257	0.69202	23.1284875	23.13863	23.23885	23.1373225
P49442	Inositol polyphosphate 1-phosphatase (IPP) (IPPase) (EC 3.1.3.57)	Inpp1	16329	0.72144	0.55824	0.25318	0.69719	20.4097275	20.374955	20.44498	20.2701175
Q80Y14	Glutaredoxin-related protein 5, mitochondrial (Monothiol glutaredoxin-5)	Glrx5	73046	0.72116	0.55839	0.25306	0.69719	18.86536	18.5620025	18.9368025	18.3818025
Q9WTX5	S-phase kinase-associated protein 1 (Cyclin-A/CDK2-associated protein p19) (S-phase kinase-associated protein 1A) (p19A) (p19skp1)	Skp1 Skp1a	21402	0.72004	0.559	0.25259	0.69735	21.938655	21.7906525	21.9111075	21.7670025
Q9CXV1	Succinate dehydrogenase [ubiquinone] cytochrome b small subunit, mitochondrial (CybS) (CII-4) (QPs3) (Succinate dehydrogenase complex subunit D) (Succinate-ubiquinone oxidoreductase cytochrome b small subunit) (Succinate-ubiquinone reductase membrane anchor subunit)	Sdhd	66925	0.71917	0.55946	0.25223	0.69735	20.642305	20.50047	20.526955	20.6061425
Q8BFZ9	Erlin-2 (Endoplasmic reticulum lipid raft-associated protein 2) (Stomatin-prohibitin-flotillin-HflC/K domain-containing protein 2) (SPFH domain-containing protein 2)	Erlin2 Spfh2	244373	0.71838	0.55989	0.2519	0.69735	19.642225	19.63269	19.449725	19.6341875
Q91XL9	Oxysterol-binding protein-related protein 1 (ORP-1) (OSBP-related protein 1)	Osbpl1a Orp1 Orp1a Orp1l	64291	0.71395	0.56229	0.25004	0.69977	19.13755	19.2840525	19.5013175	19.3288675
Q9JLB0	MAGUK p55 subfamily member 6 (Dlgh4 protein) (P55T protein) (Protein associated with Lin-7 2)	Mpp6 Dlgh4 Pals2	56524	0.71241	0.56312	0.2494	0.70024	21.1785575	21.2320575	21.29496	21.2199275
Q9DCT2	NADH dehydrogenase [ubiquinone] iron-sulfur protein 3, mitochondrial (EC 1.6.99.3) (EC 7.1.1.2) (Complex I-30kD) (CI-30kD) (NADH-ubiquinone oxidoreductase 30 kDa subunit)	Ndufs3	68349	0.70878	0.5651	0.24788	0.70212	24.2268525	24.3384075	24.2935425	24.2808725
P18572	Basigin (Basic immunoglobulin superfamily) (HT7 antigen) (Membrane glycoprotein gp42) (CD antigen CD147)	Bsg	12215	0.70737	0.56587	0.24729	0.7025	22.3592375	22.4148375	22.371375	22.4966575

Q9D880	Mitochondrial import inner membrane translocase subunit TIM50	Timm50 Tim50	66525	0.7012	0.56924	0.2447	0.70555	20.66196	20.63077	20.5369075	20.5610075
Q9CR62	Mitochondrial 2-oxoglutarate/malate carrier protein (OGCP) (Solute carrier family 25 member 11)	Slc25a11	67863	0.70146	0.5691	0.24481	0.70555	23.8291925	23.8500675	23.844225	23.76464
Q9CQ60	6-phosphogluconolactonase (6PGL) (EC 3.1.1.31)	Pgls	66171	0.69761	0.57121	0.2432	0.70742	20.1747775	20.2192575	20.3019525	20.2441725
Q9CZU6	Citrate synthase, mitochondrial (EC 2.3.3.1) (Citrate (Si)-synthase)	Cs	12974	0.69557	0.57234	0.24235	0.70766	25.5527325	25.5760325	25.4953825	25.5740575
Q80YN3	Breast carcinoma-amplified sequence 1 homolog (Novel amplified in breast cancer 1 homolog)	Bcas1 Nabc1	76960	0.696	0.5721	0.24253	0.70766	22.191195	21.96203	21.9554475	22.1293375
Q9QY76	Vesicle-associated membrane protein-associated protein B (VAMP-B) (VAMP-associated protein B) (VAP-B) (VAMP-associated protein 33b)	Vapb		0.69277	0.57389	0.24117	0.70873	21.277605	21.158195	21.1582025	21.191315
Q8BGQ7	Alanine--tRNA ligase, cytoplasmic (EC 6.1.1.7) (Alanyl-tRNA synthetase) (AlaRS) (Protein sticky) (Sti)	Aars1 Aars	234734	0.69232	0.57413	0.24099	0.70873	22.11305	22.082515	22.0922575	22.144085
P32848	Parvalbumin alpha	Pvalb Pva	19293	0.69145	0.57462	0.24062	0.70875	21.323275	21.1044825	21.1812625	21.119595
Q71M36	Chondroitin sulfate proteoglycan 5 (Acidic leucine-rich EGF-like domain-containing brain protein) (Neuroglycan C)	Cspg5 Caleb Ngc	29873	0.69061	0.57508	0.24027	0.70875	20.8824725	20.943275	20.8941825	21.0053175
P05132	cAMP-dependent protein kinase catalytic subunit alpha (PKA C-alpha) (EC 2.7.11.11)	Prkaca Pkaca	18747	0.68834	0.57634	0.23932	0.70924	20.075565	20.233725	20.12783	20.104495
Q8BVQ5	Protein phosphatase methylesterase 1 (PME-1) (EC 3.1.1.89)	Ppme1 Pme1	72590	0.68822	0.5764	0.23928	0.70924	21.3399025	21.2321325	21.26733	21.307135
Q9JLV5	Cullin-3 (CUL-3)	Cul3	26554	0.68679	0.5772	0.23867	0.70965	19.9961075	20.0327825	20.0899775	19.959425
Q61035	Histidine--tRNA ligase, cytoplasmic (EC 6.1.1.21) (Histidyl-tRNA synthetase) (HisRS)	Hars1 Hars	15115	0.68578	0.57776	0.23826	0.70976	21.4743625	21.3302625	21.431795	21.43075
Q8CGP0	Histone H2B type 3-B	Hist3h2bb Hist3h2bb-ps		0.68075	0.58056	0.23615	0.71041	26.48722	26.49495	26.4250725	26.4000525
Q61990	Poly(rC)-binding protein 2 (Alpha-CP2) (CTBP) (CBP) (Putative heterogeneous nuclear ribonucleoprotein X) (hnRNP X)	Pcbp2 Cbp Hnrnpx Hnrpx	18521	0.67955	0.58123	0.23565	0.71041	21.495175	21.5535975	21.4205325	21.5348925
P28661	Septin-4 (Brain protein H5) (Peanut-like protein 2)	Septin4 Pnutl2 Sept4	18952	0.68159	0.58009	0.2365	0.71041	20.4121475	20.28471	20.2532625	20.5052925
Q99K48	Non-POU domain-containing octamer-binding protein (NonO protein)	Nono	53610	0.68174	0.58001	0.23657	0.71041	20.8048375	20.82713	20.9695775	20.898745
Q80TL4	PHD finger protein 24	Phf24 Kiaa1045 N28178	230085	0.67979	0.58109	0.23575	0.71041	22.6817675	22.8243325	22.7329625	22.7083175
Q8R0A7	Uncharacterized protein KIAA0513	Kiaa0513	234797	0.679	0.58154	0.23542	0.71041	21.5991825	21.6208475	21.56736	21.6962625
Q6ZWU9	40S ribosomal protein S27	Rps27	100043813 57294	0.67977	0.58111	0.23575	0.71041	20.42515	20.239925	20.403305	20.4191225
Q6ZWY8	Thymosin beta-10	Tmsb10 Ptmb10	19240	0.6772	0.58255	0.23467	0.71107	23.728995	23.5687375	23.3559775	23.0224425
P26350	Prothymosin alpha [Cleaved into: Prothymosin alpha, N-terminally processed; Thymosin alpha]	Ptma	19231	0.67301	0.58489	0.23292	0.71337	21.0789725	21.01063	20.7509575	20.7015025
P60710	Actin, cytoplasmic 1 (Beta-actin) [Cleaved into: Actin, cytoplasmic 1, N-terminally processed]	Actb	11461	0.66667	0.58847	0.23027	0.71374	30.03173	30.0090725	30.0090725	30.03173
Q9R1V6	Disintegrin and metalloproteinase domain-containing protein 22 (ADAM 22)	Adam22	11496	0.67005	0.58656	0.23168	0.71374	21.8797325	21.8598325	21.938785	21.8562825
Q7TMM9	Tubulin beta-2A chain	Tubb2a Tubb2	22151	0.66667	0.58847	0.23027	0.71374	29.9411	29.9637575	29.9637575	29.9411
P99029	Peroxiredoxin-5, mitochondrial (EC 1.11.1.15) (Antioxidant enzyme B166) (AOEB166) (Liver tissue 2D-page spot 2D-0014IV) (PLP)	Prdx5 Prdx6	54683	0.67019	0.58648	0.23174	0.71374	25.61107	25.6362975	25.552865	25.575305

	(Peroxiredoxin V) (Prx-V) (Peroxisomal antioxidant enzyme) (Thioredoxin peroxidase PMP20)										
P62267	40S ribosomal protein S23	Rps23	66475	0.66817	0.58762	0.2309	0.71374	20.5751225	20.47477	20.6002275	20.6104425
Q9DCJ5	NADH dehydrogenase [ubiquinone] 1 alpha subcomplex subunit 8 (Complex I-19kD) (CI-19kD) (Complex I-PGIV) (CI-PGIV) (NADH-ubiquinone oxidoreductase 19 kDa subunit)	Ndufa8	68375	0.6674	0.58806	0.23058	0.71374	23.2056425	23.3632925	23.28728	23.31848
P47941	Crk-like protein	Crkl Crkol	12929	0.66918	0.58705	0.23132	0.71374	19.0736125	18.9858775	19.2458	19.058085
P33173	Kinesin-like protein KIF1A (Axonal transporter of synaptic vesicles)	Kif1a Atsv Kif1		0.66401	0.58998	0.22917	0.71386	19.3280675	19.310215	19.4477225	19.30712
Q8K2C9	Very-long-chain (3R)-3-hydroxyacyl-CoA dehydratase 3 (EC 4.2.1.134) (3-hydroxyacyl-CoA dehydratase 3) (HACD3) (Butyrate-induced protein 1) (B-ind1) (Protein-tyrosine phosphatase-like A domain-containing protein 1)	Hacd3 Ptplad1	57874	0.66476	0.58955	0.22948	0.71386	20.8048975	20.9330575	20.8566775	21.2158425
P19246	Neurofilament heavy polypeptide (NF-H) (200 kDa neurofilament protein) (Neurofilament triplet H protein)	Nefh Kiaa0845 Nfh	380684	0.66422	0.58986	0.22925	0.71386	22.4989025	22.52425	22.41436	22.70613
Q0VF58	Collagen alpha-1(XIX) chain (Collagen alpha-1(Y) chain)	Col19a1	12823	0.66169	0.59129	0.2282	0.71488	21.046835	20.9760725	21.1355375	20.85078
Q6ZQF0	DNA topoisomerase 2-binding protein 1 (DNA topoisomerase II-beta-binding protein 1) (TopBP1) (DNA topoisomerase II-binding protein 1)	Topbp1 Kiaa0259	235559	0.65685	0.59404	0.22618	0.71764	20.4057	20.3096225	20.1767	20.16099
Q91V41	Ras-related protein Rab-14	Rab14	68365	0.65592	0.59457	0.2258	0.71772	25.4289625	25.3726725	25.3452325	25.3993875
Q60930	Voltage-dependent anion-selective channel protein 2 (VDAC-2) (mVDAC2) (Outer mitochondrial membrane protein porin 2) (Voltage-dependent anion-selective channel protein 6) (VDAC-6) (mVDAC6)	Vdac2 Vdac6	22334	0.65287	0.59632	0.22452	0.71869	26.0212825	26.08176	25.9919375	26.04852
Q9D6J6	NADH dehydrogenase [ubiquinone] flavoprotein 2, mitochondrial (EC 1.6.99.3) (EC 7.1.1.2) (NADH-ubiquinone oxidoreductase 24 kDa subunit)	Ndufv2	72900	0.65363	0.59588	0.22484	0.71869	23.3062825	23.34294	23.4153225	23.318145
O54991	Contactin-associated protein 1 (Caspr) (Caspr1) (MHDNIV) (NCP1) (Neurexin IV) (Neurexin-4) (Paranodin)	Cntnap1 Nrxn4	53321	0.64433	0.60121	0.22097	0.72402	21.4543975	21.3338825	21.367095	21.3295575
P47753	F-actin-capping protein subunit alpha-1 (CapZ alpha-1)	Capza1 Cappa1	0	0.64288	0.60205	0.22037	0.72446	18.77487	18.6916125	18.9875775	19.05171
P14094	Sodium/potassium-transporting ATPase subunit beta-1 (Sodium/potassium-dependent ATPase subunit beta-1)	Atp1b1 Atp4b	11931	0.64099	0.60314	0.21958	0.7252	27.3741825	27.3218375	27.3109975	27.2961125
Q6PDI5	Proteasome adapter and scaffold protein ECM29 (Proteasome-associated protein ECM29 homolog)	Ecpas Ecm29 Kiaa0368	230249	0.63873	0.60445	0.21864	0.7262	14.125367	14.86012725	17.57586625	18.07891
P84104	Serine/arginine-rich splicing factor 3 (Pre-mRNA-splicing factor SRP20) (Protein X16) (Splicing factor, arginine/serine-rich 3)	Srsf3 Sfrs3 Srp20 X16	20383	0.63731	0.60527	0.21805	0.72661	21.09957	21.009715	21.0467625	21.04249
Q62393	Tumor protein D52 (mD52)	Tpd52	21985	0.63598	0.60604	0.2175	0.72697	20.5481775	20.56424	20.5155975	20.3673825
B9EKR1	Receptor-type tyrosine-protein phosphatase zeta (R-PTP-zeta) (EC 3.1.3.48)	Ptprz1	19283	0.63373	0.60735	0.21656	0.72796	23.7436725	23.6264925	23.6456425	23.7143175
Q9ES97	Reticulon-3	Rtn3	20168	0.632	0.60835	0.21584	0.72859	24.1338625	24.003185	24.036475	23.98825
Q9CXW3	Calcyclin-binding protein (CacyBP) (Siah-interacting protein)	Cacybp Sip	12301	0.62876	0.61024	0.2145	0.73028	20.7429025	20.74125	20.91106	20.8586125
Q78IK2	ATP synthase membrane subunit DAPIT, mitochondrial (Diabetes-associated protein in	Atp5md Dapit Usmg5	66477	0.62612	0.61178	0.2134	0.73064	22.7441025	22.69472	22.6118375	22.712395

	insulin-sensitive tissues) (Up-regulated during skeletal muscle growth protein 5)										
Q91ZP9	N-terminal EF-hand calcium-binding protein 2 (EF-hand calcium-binding protein 2) (Neuronal calcium-binding protein 2)	Necab2 Efcbp2	117148	0.6258	0.61197	0.21327	0.73064	19.5272475	19.46906	19.3895075	19.2525275
Q8VCT3	Aminopeptidase B (AP-B) (EC 3.4.11.6) (Arginine aminopeptidase) (Arginyl aminopeptidase) (Cytosol aminopeptidase IV)	Rnpep	215615	0.62643	0.6116	0.21353	0.73064	21.0568925	21.05538	21.166675	21.1577725
Q8BW75	Amine oxidase [flavin-containing] B (EC 1.4.3.4) (Monoamine oxidase type B) (MAO-B)	Maob	109731	0.62472	0.6126	0.21282	0.73082	21.5448975	21.61033	21.410745	21.55496
Q9WTR5	Cadherin-13 (Heart cadherin) (H-cadherin) (Truncated cadherin) (T-cad) (T-cadherin)	Cdh13	12554	0.62087	0.61486	0.21122	0.73294	19.84763	20.06908	20.15276	19.93661
P47791	Glutathione reductase, mitochondrial (GR) (GRase) (EC 1.8.1.7)	Gsr Gr1	14782	0.61962	0.61559	0.21071	0.73324	19.1911275	19.168095	19.370085	19.35664
Q9D0J8	Parathymosin	Ptms	69202	0.61634	0.61753	0.20934	0.73497	22.4131775	22.1905875	22.1542475	22.187495
Q3TDK6	Protein rogdi homolog (Leucine-zipper-containing LZF)	Rogdi Lzf	66049	0.61509	0.61826	0.20883	0.73527	21.24567	21.31084	21.16839	21.0557475
A2ALS5	Rap1 GTPase-activating protein 1 (Rap1GAP) (Rap1GAP1) (ARPP-90)	Rap1gap Kiaa0474 Rap1ga1	110351	0.61226	0.61994	0.20765	0.73611	19.795645	19.6227025	19.7817675	19.7014225
Q6ZPJ3	(E3-independent) E2 ubiquitin-conjugating enzyme UBE2O (EC 2.3.2.24) (E2/E3 hybrid ubiquitin-protein ligase UBE2O) (Ubiquitin carrier protein O) (Ubiquitin-conjugating enzyme E2 O) (Ubiquitin-conjugating enzyme E2 of 230 kDa) (Ubiquitin-conjugating enzyme E2-230K) (Ubiquitin-protein ligase O)	Ube2o Kiaa1734	217342	0.61245	0.61982	0.20773	0.73611	20.95143	20.94393	21.09994	20.969355
P61264	Syntaxin-1B	Stx1b Stx1b1 Stx1b2	56216	0.60979	0.6214	0.20663	0.73727	25.75874	25.6951225	25.6815825	25.66157
Q78ZA7	Nucleosome assembly protein 1-like 4	Nap1l4	17955	0.60733	0.62285	0.20561	0.73772	20.9538125	20.93848	20.967315	21.0036625
O35326	Serine/arginine-rich splicing factor 5 (Delayed-early protein HRS) (Pre-mRNA-splicing factor SRP40) (Splicing factor, arginine/serine-rich 5)	Srsf5 Hrs Sfrs5	20384	0.60671	0.62322	0.20536	0.73772	19.66358	19.584515	19.455565	19.567825
Q80Z24	Neuronal growth regulator 1 (Kindred of IgLON) (Kilon) (Neurotractin)	Negr1 Kiaa3001 Ntra	320840	0.60824	0.62232	0.20599	0.73772	20.82278	20.8449025	20.795825	20.6695175
Q9DCS9	NADH dehydrogenase [ubiquinone] 1 beta subcomplex subunit 10 (Complex I-PDSW) (CI-PDSW) (NADH-ubiquinone oxidoreductase PDSW subunit)	Ndufb10	68342	0.60246	0.62575	0.2036	0.74014	23.298245	23.2715125	23.35829	23.283415
Q9Z2D6	Methyl-CpG-binding protein 2 (MeCp-2 protein) (MeCp2)	Mecp2	17257	0.59945	0.62755	0.20235	0.7417	19.424525	19.3040225	19.3480225	19.29774
Q9JIA1	Leucine-rich glioma-inactivated protein 1	Lgi1	56839	0.59704	0.62899	0.20136	0.74282	22.9902475	22.96359	22.9911175	22.920735
O55029	Coatomer subunit beta' (Beta'-coat protein) (Beta'-COP) (p102)	Copb2	50797	0.59511	0.63014	0.20056	0.74361	20.3268875	20.1848775	20.29418	20.322895
Q14C59	Transmembrane protease serine 11B-like protein (EC 3.4.21.-) (Airway trypsin-like protease 5) (Transmembrane protease serine 11B)	Tmprss11b Hatl5 Tmprss11bnl	319875	0.59241	0.63177	0.19944	0.74495	19.632375	19.4589725	19.5310225	19.6473825
Q3U0V1	Far upstream element-binding protein 2 (FUSE-binding protein 2) (KH type-splicing regulatory protein) (KSRP)	Khsrp Fubp2	16549	0.58531	0.63604	0.19651	0.74883	20.58842	20.4831675	20.6078725	20.5980725
Q9D0E1	Heterogeneous nuclear ribonucleoprotein M (hnRNP M)	Hnrnpm Hnrpm	76936	0.58582	0.63574	0.19672	0.74883	21.387855	21.290205	21.2379675	21.0607225
Q8VEM8	Phosphate carrier protein, mitochondrial (Phosphate transport protein) (PTP) (Solute carrier family 25 member 3)	Slc25a3	18674	0.58009	0.63921	0.19436	0.75169	25.6786475	25.748555	25.74728	25.7342325
Q9DCU2	Plasmolipin (Plasma membrane proteolipid)	Pllp Plapi Pmlp Tm4sf11	67801	0.57969	0.63945	0.19419	0.75169	21.5525225	21.4903825	21.56044	21.8321

Q9QUI0	Transforming protein RhoA (EC 3.6.5.2)	Rhoa Arha Arha2	11848	0.57679	0.64121	0.193	0.75318	23.399875	23.3848475	23.40583	23.4488825
O35295	Transcriptional activator protein Pur-beta (Purine-rich element-binding protein B) (Vascular actin single-stranded DNA-binding factor 2 p44 component)	Purb	19291	0.57556	0.64196	0.19249	0.75331	21.7552175	21.667515	21.734545	21.60397
Q99N28	Cell adhesion molecule 3 (Immunoglobulin superfamily member 4B) (IgSF4B) (Nectin-like protein 1) (NECL-1) (Synaptic cell adhesion molecule 3) (TSLC1-like protein 1)	Cadm3 Igsf4b Necl1 Syncam3 Tsll1	94332	0.57498	0.64231	0.19225	0.75331	23.2385975	23.19288	23.2276575	23.18953
P08553	Neurofilament medium polypeptide (NF-M) (160 kDa neurofilament protein) (Neurofilament 3) (Neurofilament triplet M protein)	Nefm Nef3 Nfm	18040	0.57265	0.64373	0.19129	0.7544	25.9022	25.8294025	25.755125	25.9873675
A2AJI0	MAP7 domain-containing protein 1	Map7d1 Kiaa1187 Mtap7d1	245877	0.57055	0.64502	0.19043	0.75532	19.3138875	19.3245425	19.33096	19.464045
Q8BIG7	Catechol O-methyltransferase domain-containing protein 1 (EC 2.1.1.-)	Comtd1	69156	0.56936	0.64574	0.18994	0.75559	19.5965525	19.484995	19.519685	19.4572025
Q00PI9	Heterogeneous nuclear ribonucleoprotein U-like protein 2 (MLF1-associated nuclear protein)	Hnrnpul2 Hnrpul2 Manp	68693	0.56227	0.65009	0.18703	0.7601	20.9989225	20.9136375	20.927105	21.0049775
O35927	Catenin delta-2 (Neural plakophilin-related ARM-repeat protein) (NPRAP) (Neurojungin)	Ctnnd2 Catnd2 Nprap	18163	0.55565	0.65418	0.18431	0.76429	21.7472575	21.73285	21.62164	21.6731575
Q924M7	Mannose-6-phosphate isomerase (EC 5.3.1.8) (Phosphohexomutase) (Phosphomannose isomerase) (PMI)	Mpi Mpi1 Pmi	110119	0.55369	0.65539	0.1835	0.76512	20.2633775	20.2553775	20.3557275	20.2579375
Q8CAQ8	MICOS complex subunit Mic60 (Mitochondrial inner membrane protein) (Mitofilin)	Immt Mic60	76614	0.55283	0.65592	0.18315	0.76516	24.5860375	24.5634725	24.5206025	24.522345
P16125	L-lactate dehydrogenase B chain (LDH-B) (EC 1.1.1.27) (LDH heart subunit) (LDH-H)	Ldhb Ldh-2 Ldh2	16832	0.55075	0.65721	0.1823	0.76572	26.93866	26.8553675	26.84615	26.89334
Q9WUA2	Phenylalanine--tRNA ligase beta subunit (EC 6.1.1.20) (Phenylalanyl-tRNA synthetase beta subunit) (PheRS)	Farsb Farsl Farslb Frsb	23874	0.55031	0.65748	0.18212	0.76572	20.5475225	20.5961575	20.49257	20.5185825
P56812	Programmed cell death protein 5 (TF-1 cell apoptosis-related protein 19) (Protein TFAR19)	Pdcd5 Tfar19	56330	0.54962	0.65791	0.18184	0.76572	19.43585	19.273745	19.37104	19.366785
Q6P1F6	Serine/threonine-protein phosphatase 2A 55 kDa regulatory subunit B alpha isoform (PP2A subunit B isoform B55-alpha) (PP2A subunit B isoform PR55-alpha) (PP2A subunit B isoform R2-alpha) (PP2A subunit B isoform alpha)	Ppp2r2a	71978	0.54773	0.65909	0.18106	0.76651	22.0780925	22.0471975	22.0862	22.0930275
P48771	Cytochrome c oxidase subunit 7A2, mitochondrial (Cytochrome c oxidase subunit VIIa-liver/heart) (Cytochrome c oxidase subunit VIIa-L)	Cox7a2 Cox7a3 Cox7al	12866	0.54635	0.65994	0.18049	0.76692	23.897665	23.802065	23.883725	23.83333
O09167	60S ribosomal protein L21	Rpl21		0.54325	0.66187	0.17922	0.768	20.407645	20.2586225	20.332275	20.420655
P80317	T-complex protein 1 subunit zeta (TCP-1-zeta) (CCT-zeta-1)	Cct6a Cct6 Cctz Cctz1	12466	0.54404	0.66138	0.17955	0.768	22.73703	22.7023725	22.7774425	22.8010575
Q6PDL0	Cytoplasmic dynein 1 light intermediate chain 2 (Dynein light intermediate chain 2, cytosolic)	Dync1li2 Dncli2 Dnclic2	234663	0.54036	0.66368	0.17804	0.76897	19.829175	19.91552	19.9675225	19.9471475
Q6P560	Zinc finger protein 182 (Zinc finger protein 21)	Znf182 Zfp182 Znf21	319535	0.5403	0.66372	0.17802	0.76897	20.72543	22.2727925	21.559485	22.3706325
D3Z7P3	Glutaminase kidney isoform, mitochondrial (GLS) (EC 3.5.1.2) [Cleaved into: Glutaminase kidney isoform, mitochondrial 68 kDa chain; Glutaminase kidney isoform, mitochondrial 65 kDa chain]	Gls Gls1 Kiaa0838	14660	0.53579	0.66654	0.17617	0.77165	23.9671725	23.990335	24.0529875	23.9943825
Q99KK2	N-acylneuraminate cytidylyltransferase (EC 2.7.7.43) (CMP-N-acetylneuraminic acid synthase) (CMP-NeuNAc synthase)	Cmas	12764	0.53452	0.66734	0.17565	0.77199	19.4260175	19.361645	19.479915	19.51582
P06837	Neuromodulin (Axonal membrane protein GAP-43) (Calmodulin-binding protein P-57) (Growth-associated protein 43)	Gap43 Basp2	14432	0.53246	0.66863	0.17481	0.77245	24.8946425	24.8012025	24.8742975	24.6458975

P57759	Endoplasmic reticulum resident protein 29 (ERp29)	Erp29	67397	0.53227	0.66875	0.17474	0.77245	20.978605	20.9293725	21.051675	21.0075475
Q9CY27	Very-long-chain enoyl-CoA reductase (EC 1.3.1.93) (Synaptic glycoprotein SC2) (Trans-2,3-enoyl-CoA reductase) (TER)	Tecr Gpsn2	106529	0.52973	0.67035	0.1737	0.77371	20.017495	20.0842575	19.90189	20.0622475
Q9D8B3	Charged multivesicular body protein 4b (Chromatin-modifying protein 4b) (CHMP4b)	Chmp4b	75608	0.52873	0.67098	0.17329	0.77386	20.4020225	20.407805	20.29748	20.4432325
P50247	Adenosylhomocysteinase (AdoHcyase) (EC 3.3.1.1) (CUBP) (Liver copper-binding protein) (S-adenosyl-L-homocysteine hydrolase)	Ahcy	11615 269378	0.52721	0.67194	0.17267	0.77438	21.609715	21.60003	21.5382075	21.645095
P62962	Profilin-1 (Profilin I)	Pfn1	18643	0.52594	0.67274	0.17215	0.77471	24.29241	24.2544475	24.3824475	24.261385
Q8VDQ8	NAD-dependent protein deacetylase sirtuin-2 (EC 2.3.1.286) (Regulatory protein SIR2 homolog 2) (SIR2-like protein 2) (mSIR2L2)	Sirt2 Sir2l2	64383	0.52469	0.67353	0.17164	0.77504	24.2321	24.1453825	24.0793375	24.30011
P10639	Thioredoxin (Trx) (ATL-derived factor) (ADF)	Txn Txn1	22166	0.52117	0.67575	0.17021	0.77643	22.98461	22.869955	22.9011725	22.90694
Q9Z1D1	Eukaryotic translation initiation factor 3 subunit G (eIF3g) (Eukaryotic translation initiation factor 3 RNA-binding subunit) (eIF-3 RNA-binding subunit) (Eukaryotic translation initiation factor 3 subunit 4) (eIF-3-delta) (eIF3 p42) (eIF3 p44)	Eif3g Eif3p42 Eif3s4	53356	0.52172	0.67541	0.17043	0.77643	18.857965	18.28279	18.6431175	18.41304
O35143	ATPase inhibitor, mitochondrial (ATP synthase F1 subunit epsilon) (Inhibitor of F(1)F(o)-ATPase) (IF(1)) (IF1)	ATP5IF1 Atpi Atpif1 If1	11983	0.51639	0.67879	0.16826	0.777	21.838505	21.80439	21.643775	21.80346
Q9JM14	5'(3')-deoxyribonucleotidase, cytosolic type (EC 3.1.3.-) (Cytosolic 5',3'-pyrimidine nucleotidase) (Deoxy-5'-nucleotidase 1) (dNT-1)	Nt5c Dnt1	50773	0.51783	0.67787	0.16885	0.777	20.8265825	20.75999	20.891325	20.7513775
Q99JI4	26S proteasome non-ATPase regulatory subunit 6 (26S proteasome regulatory subunit RPN7) (26S proteasome regulatory subunit S10) (p42A)	Psmd6	66413	0.5175	0.67808	0.16872	0.777	20.60375	20.604515	20.5774975	20.7586775
Q9CYN9	Renin receptor (ATPase H(+)-transporting lysosomal accessory protein 2) (ATPase H(+)-transporting lysosomal-interacting protein 2) (Renin/prorenin receptor) [Cleaved into: Renin receptor extracellular fragment; Renin receptor cytoplasmic fragment]	Atp6ap2 Atp6ip2	70495	0.51782	0.67788	0.16884	0.777	20.1294475	20.06509	20.020295	19.931495
E9Q414	Apolipoprotein B-100 (Apo B-100) [Cleaved into: Apolipoprotein B-48 (Apo B-48)]	Apob	238055	0.51649	0.67873	0.1683	0.777	19.9735675	19.7823475	19.8078125	19.8812625
Q8R4F1	Netrin-G2 (Laminet-2)	Ntng2 Lmnt2	171171	0.51201	0.68158	0.16648	0.77948	20.263435	20.220705	20.351655	20.20423
P61922	4-aminobutyrate aminotransferase, mitochondrial (EC 2.6.1.19) ((S)-3-amino-2-methylpropionate transaminase) (EC 2.6.1.22) (GABA aminotransferase) (GABA-AT) (Gamma-amino-N-butyrate transaminase) (GABA transaminase) (GABA-T) (L-AIBAT)	Abat Gabat	268860	0.51059	0.68249	0.16591	0.77948	24.5049325	24.57042	24.7206425	24.5279675
P21614	Vitamin D-binding protein (DBP) (VDB) (Gc-globulin) (Group-specific component)	Gc	14473	0.51125	0.68206	0.16617	0.77948	16.490914	18.0932775	16.53169325	14.981235
P63030	Mitochondrial pyruvate carrier 1 (Brain protein 44-like protein)	Mpc1 Brp44l	55951	0.50877	0.68365	0.16517	0.77994	23.588895	23.373545	23.342345	23.4332275
Q99K51	Plastin-3 (T-plastin)	Pls3	102866	0.50835	0.68391	0.165	0.77994	21.1673625	21.15171	21.166295	21.255445
P49443	Protein phosphatase 1A (EC 3.1.3.16) (Protein phosphatase 2C isoform alpha) (PP2C-alpha) (Protein phosphatase IA)	Ppm1a Pppm1a	19042	0.50639	0.68517	0.1642	0.78079	20.62629	20.5325025	20.5410225	20.5347975
P36536	GTP-binding protein SAR1a	Sar1a Sara Sara1		0.50471	0.68625	0.16352	0.78143	18.7111	18.7986525	18.927365	18.8038425
Q9CQA3	Succinate dehydrogenase [ubiquinone] iron-sulfur subunit, mitochondrial (EC 1.3.5.1) (Iron-sulfur subunit of complex II) (Ip)	Sdhb	67680	0.50174	0.68815	0.16232	0.78301	23.0351325	22.9942875	23.001335	23.065625

Q9QZB7	Actin-related protein 10 (Actin-related protein 11)	Actr10 Act11 Actr11 Arp11	56444	0.50064	0.68886	0.16187	0.78324	19.15061	19.258725	19.2658025	19.2742425
Q02819	Nucleobindin-1 (CALNUC)	Nucb1 Nuc Nucb	18220	0.49083	0.69518	0.1579	0.78984	18.2786125	18.4763925	18.5883725	18.688305
P84096	Rho-related GTP-binding protein RhoG (Sid 10750)	Rhog Arhg Sid10750	56212	0.48883	0.69648	0.15709	0.79072	21.514095	21.3169025	21.2875525	21.43976
Q6IRU5	Clathrin light chain B (Lcb)	Cltb	74325	0.48692	0.69771	0.15632	0.79141	23.6800575	23.583945	23.5842575	23.6234775
Q8CGC7	Bifunctional glutamate/proline--tRNA ligase (Bifunctional aminoacyl-tRNA synthetase) [Includes: Glutamate--tRNA ligase (EC 6.1.1.17) (Glutamyl-tRNA synthetase) (GluRS); Proline--tRNA ligase (EC 6.1.1.15) (Prolyl-tRNA synthetase) (ProRS)]	Eprs1 Eprs Qprs	107508	0.4863	0.69812	0.15607	0.79141	21.059425	21.0891075	21.1118275	21.042925
Q11011	Puromycin-sensitive aminopeptidase (PSA) (EC 3.4.11.14) (Cytosol alanyl aminopeptidase) (AAP-S)	Npepps Psa	19155	0.48482	0.69908	0.15547	0.79191	24.6584375	24.636915	24.6495125	24.58896
Q9D6F9	Tubulin beta-4A chain (Tubulin beta-4 chain)	Tubb4a Tubb4	22153	0.48259	0.70053	0.15457	0.79296	24.9497225	24.9981525	25.020655	25.08683
Q2PFD7	PH and SEC7 domain-containing protein 3 (Exchange factor for ADP-ribosylation factor guanine nucleotide factor 6 D) (Exchange factor for ARF6 D) (Pleckstrin homology and SEC7 domain-containing protein 3)	Psd3 Efa6d Kiaa0942	234353	0.47962	0.70246	0.15338	0.79456	23.24336	23.22721	23.18288	23.193235
Q9CQ69	Cytochrome b-c1 complex subunit 8 (Complex III subunit 8) (Complex III subunit VIII) (Ubiquinol-cytochrome c reductase complex 9.5 kDa protein) (Ubiquinol-cytochrome c reductase complex ubiquinone-binding protein QP-C)	Uqcrq	22272	0.47536	0.70524	0.15166	0.79711	21.8155825	21.77893	21.7158625	21.645475
Q99LF4	RNA-splicing ligase RtcB homolog (EC 6.5.1.8) (3'-phosphate/5'-hydroxy nucleic acid ligase) (Focal adhesion-associated protein) (FAAP)	Rtcb D10Wsu52e	28088	0.47439	0.70587	0.15127	0.79724	19.86073	19.64486	19.7414875	19.8995375
P11276	Fibronectin (FN) [Cleaved into: Anastellin]	Fn1	14268	0.46695	0.71074	0.14829	0.80156	20.8395075	20.69517	20.712665	20.72692
Q8VEK0	Cell cycle control protein 50A (P4-ATPase flippase complex beta subunit TMEM30A) (Transmembrane protein 30A)	Tmem30a Cdc50a D9Wsu20e	69981	0.46738	0.71046	0.14846	0.80156	19.4911275	19.466395	19.4319525	19.3736325
Q791V5	Mitochondrial carrier homolog 2	Mtch2	56428	0.45423	0.71913	0.14319	0.81042	23.2122	23.15347	23.1860925	23.16899
Q9R1P4	Proteasome subunit alpha type-1 (EC 3.4.25.1) (Macropain subunit C2) (Multicatalytic endopeptidase complex subunit C2) (Proteasome component C2) (Proteasome nu chain)	Psma1	26440	0.45172	0.72079	0.14219	0.81109	21.5418775	21.46869	21.483735	21.53461
O88544	COP9 signalosome complex subunit 4 (SGN4) (Signalosome subunit 4) (JAB1-containing signalosome subunit 4)	Cops4 Csn4	26891	0.45222	0.72046	0.14239	0.81109	21.2085	21.1679875	21.2658075	21.1689375
P62743	AP-2 complex subunit sigma (Adaptor protein complex AP-2 subunit sigma) (Adaptor-related protein complex 2 subunit sigma) (Clathrin assembly protein 2 sigma small chain) (Clathrin coat assembly protein AP17) (Clathrin coat-associated protein AP17) (Plasma membrane adaptor AP-2 17 kDa protein) (Sigma-adaptin 3b) (Sigma2-adaptin)	Ap2s1 Ap17 Claps2	232910	0.44936	0.72236	0.14125	0.81225	22.2143175	22.22836	22.263355	22.172595
Q3TC72	Fumarylacetoacetate hydrolase domain-containing protein 2A (EC 3.-.-.-)	Fahd2 Fahd2a	68126	0.4479	0.72333	0.14066	0.81275	20.2597075	20.2336925	20.3297825	20.2390775
P62192	26S proteasome regulatory subunit 4 (P26s4) (26S proteasome AAA-ATPase subunit RPT2) (Proteasome 26S subunit ATPase 1)	Psmc1	19179	0.44305	0.72655	0.13873	0.81338	20.973175	21.030195	21.0830025	20.9268125
P51150	Ras-related protein Rab-7a	Rab7a Rab7	19349	0.44317	0.72647	0.13878	0.81338	23.3971225	23.407135	23.445655	23.45867

Q9DCB4	cAMP-regulated phosphoprotein 21 (ARPP-21) (Regulator of calmodulin signaling) (Thymocyte cAMP-regulated phosphoprotein)	Arpp21 Rcs Tarpp	74100	0.4445	0.72559	0.13931	0.81338	18.7458025	18.434755	18.69274	18.6248925
P58281	Dynamin-like 120 kDa protein, mitochondrial (EC 3.6.5.5) (Large GTP-binding protein) (LargeG) (Optic atrophy protein 1 homolog) [Cleaved into: Dynamin-like 120 kDa protein, form S1]	Opa1	74143	0.44441	0.72565	0.13928	0.81338	23.6545425	23.66265	23.6779	23.6121825
P59325	Eukaryotic translation initiation factor 5 (eIF-5)	Eif5	217869	0.44438	0.72567	0.13926	0.81338	19.605895	19.6369875	19.6544325	19.699115
Q6P5F9	Exportin-1 (Exp1) (Chromosome region maintenance 1 protein homolog)	Xpo1 Crm1	103573	0.43936	0.72901	0.13726	0.81553	20.9028725	20.9784975	21.11056	20.9793425
P22315	Ferrochelatase, mitochondrial (EC 4.99.1.1) (Heme synthase) (Protoheme ferro-lyase)	Fech		0.43347	0.73295	0.13493	0.81813	19.7398125	19.7463025	19.6808975	19.6965375
Q62446	Peptidyl-prolyl cis-trans isomerase FKBP3 (PPIase FKBP3) (EC 5.2.1.8) (25 kDa FK506-binding protein) (25 kDa FKBP) (FKBP-25) (FK506-binding protein 3) (FKBP-3) (Immunophilin FKBP25) (Rapamycin-selective 25 kDa immunophilin) (Rotamase)	Fkbp3 Fkbp25	30795	0.43355	0.7329	0.13496	0.81813	19.457665	19.43048	19.325905	19.4675525
Q8C2Q3	RNA-binding protein 14 (RNA-binding motif protein 14)	Rbm14	56275	0.43417	0.73248	0.1352	0.81813	18.7404275	18.535705	18.6030125	18.7359125
Q60676	Serine/threonine-protein phosphatase 5 (PP5) (EC 3.1.3.16) (Protein phosphatase T) (PPT)	Ppp5c	19060	0.43246	0.73362	0.13453	0.81829	20.749335	20.6873375	20.75519	20.602485
Q9JHW4	Selenocysteine-specific elongation factor (Elongation factor sec) (Eukaryotic elongation factor, selenocysteine-tRNA-specific) (mSelB)	Eefsec Selb	65967	0.43082	0.73472	0.13388	0.81892	20.07974	20.0929025	20.0511825	20.201615
O09061	Proteasome subunit beta type-1 (EC 3.4.25.1) (Macropain subunit C5) (Multicatalytic endopeptidase complex subunit C5) (Proteasome component C5) (Proteasome gamma chain)	Psmb1	19170	0.42839	0.73635	0.13291	0.82014	22.104535	22.0138275	22.0406725	22.020055
P46664	Adenylosuccinate synthetase isozyme 2 (AMPSase 2) (AdSS 2) (EC 6.3.4.4) (Adenylosuccinate synthetase, acidic isozyme) (Adenylosuccinate synthetase, liver isozyme) (L-type adenylosuccinate synthetase) (IMP--aspartate ligase 2)	Adss2 Adss	11566	0.42614	0.73787	0.13202	0.82078	19.9639775	19.915825	20.066035	19.9796375
Q60749	KH domain-containing, RNA-binding, signal transduction-associated protein 1 (GAP-associated tyrosine phosphoprotein p62) (Src-associated in mitosis 68 kDa protein) (Sam68) (p21 Ras GTPase-activating protein-associated p62) (p68)	Khdrbs1	20218	0.42593	0.738	0.13194	0.82078	19.93361	19.949695	20.15389	20.0333475
Q9JHK4	Geranylgeranyl transferase type-2 subunit alpha (EC 2.5.1.60) (Geranylgeranyl transferase type II subunit alpha) (Rab geranyl-geranyltransferase subunit alpha) (Rab GG transferase alpha) (Rab GGTase alpha) (Rab geranylgeranyltransferase subunit alpha)	Rabggta	56187	0.42421	0.73916	0.13126	0.82147	19.5199925	19.3230175	19.4616825	19.345395
Q91WD7	Kinesin-like protein KIF18A	Kif18a	228421	0.42258	0.74026	0.13062	0.82209	20.2367525	20.10238	20.0223175	20.017995
Q6NZL0	Protein SOGA3	Soga3	67412	0.42165	0.74089	0.13025	0.82219	20.1458225	20.06014	20.0636575	20.01251
Q99PL6	UBX domain-containing protein 6 (UBX domain-containing protein 1)	Ubxn6 Ubxd1 Ubxdc2	66530	0.41601	0.74469	0.12802	0.82537	19.217285	19.7559525	19.61843	19.61881
Q3THS6	S-adenosylmethionine synthase isoform type-2 (AdoMet synthase 2) (EC 2.5.1.6) (Methionine adenosyltransferase 2) (MAT 2)	Mat2a	232087	0.41579	0.74484	0.12794	0.82537	20.8388075	20.9117725	20.881725	20.94072
Q9Z1Z2	Serine-threonine kinase receptor-associated protein (UNR-interacting protein)	Strap Unrip	20901	0.41433	0.74583	0.12736	0.82587	20.8182325	20.8073675	20.83894	20.7150775
Q8R574	Phosphoribosyl pyrophosphate synthase-associated protein 2 (PRPP synthase-associated protein 2) (41 kDa	Prpsap2	212627	0.40852	0.74976	0.12508	0.82962	22.17065	22.0738025	21.965985	22.0479875

	phosphoribosypyrophosphate synthetase-associated protein) (PAP41)										
P61971	Nuclear transport factor 2 (NTF-2)	Nutf2 Ntf2	68051	0.40597	0.75149	0.12408	0.83074	20.650785	20.773735	20.9249475	20.7216875
A2AGT5	Cytoskeleton-associated protein 5	Ckap5	75786	0.40542	0.75186	0.12386	0.83074	19.671905	19.559035	19.62689	19.7106625
Q9WV92	Band 4.1-like protein 3 (4.1B) (Differentially expressed in adenocarcinoma of the lung protein 1) (DAL-1) (DAL1P) (mDAL-1) [Cleaved into: Band 4.1-like protein 3, N-terminally processed]	Epb41l3 Dal1 Epb4.1l3 Kiaa0987	13823	0.40006	0.75551	0.12176	0.83357	23.9580825	23.9859125	23.9218375	23.9998475
Q61481	Calcium/calmodulin-dependent 3',5'-cyclic nucleotide phosphodiesterase 1A (Cam-PDE 1A) (EC 3.1.4.17) (61 kDa Cam-PDE)	Pde1a	18573	0.40044	0.75525	0.12191	0.83357	20.0583225	19.972805	20.0697775	20.1210525
P35235	Tyrosine-protein phosphatase non-receptor type 11 (EC 3.1.3.48) (Protein-tyrosine phosphatase SYP) (SH-PTP2) (SHP-2) (Shp2)	Ptpn11	19247	0.39844	0.7566	0.12113	0.83418	19.527975	19.481395	19.605525	19.39986
Q9CXY6	Interleukin enhancer-binding factor 2 (Nuclear factor of activated T-cells 45 kDa)	Ilf2 Nf45	67781	0.39753	0.75722	0.12078	0.83426	19.3863025	19.27132	19.2890675	19.2817
Q61598	Rab GDP dissociation inhibitor beta (Rab GDI beta) (GDI-3) (Guanosine diphosphate dissociation inhibitor 2) (GDI-2)	Gdi2 Gdi3	14569	0.39388	0.75972	0.11935	0.83519	25.80312	25.82238	25.83073	25.83776
Q9CQ54	NADH dehydrogenase [ubiquinone] 1 subunit C2 (Complex I-B14.5b) (CI-B14.5b) (NADH-ubiquinone oxidoreductase subunit B14.5b)	Ndufc2	68197	0.39509	0.75889	0.11982	0.83519	21.2656775	21.2752825	21.365185	21.281705
Q8BGN3	Glycerophosphocholine cholinephosphodiesterase ENPP6 (GPC-Cpde) (EC 3.1.4.-) (EC 3.1.4.38) (Choline-specific glycerophosphodiester phosphodiesterase) (Ectonucleotide pyrophosphatase/phosphodiesterase family member 6) (E-NPP 6) (NPP-6)	Enpp6	320981	0.39466	0.75918	0.11965	0.83519	19.5051525	19.397385	19.59536	19.648385
P38647	Stress-70 protein, mitochondrial (75 kDa glucose-regulated protein) (GRP-75) (Heat shock 70 kDa protein 9) (Mortalin) (Peptide-binding protein 74) (PBP74) (p66 MOT)	Hspa9 Grp75 Hsp74 Hspa9a	15526	0.39076	0.76185	0.11813	0.83573	24.6048075	24.5738225	24.5895625	24.6357375
Q9CR68	Cytochrome b-c1 complex subunit Rieske, mitochondrial (EC 7.1.1.8) (Complex III subunit 5) (Cytochrome b-c1 complex subunit 5) (Rieske iron-sulfur protein) (RISP) (Rieske protein UQCRFS1) (Ubiquinol-cytochrome c reductase iron-sulfur subunit) [Cleaved into: Cytochrome b-c1 complex subunit 9 (Su9) (Subunit 9) (8 kDa subunit 9) (Complex III subunit IX) (Cytochrome b-c1 complex subunit 11) (UQCRFS1 mitochondrial targeting sequence) (UQCRFS1 MTS) (Ubiquinol-cytochrome c reductase 8 kDa protein)]	Uqcrfs1	66694	0.3914	0.76141	0.11838	0.83573	23.716735	23.76325	23.6645825	23.69577
Q8BMF4	Dihydrolipoyllysine-residue acetyltransferase component of pyruvate dehydrogenase complex, mitochondrial (EC 2.3.1.12) (Dihydrolipoamide acetyltransferase component of pyruvate dehydrogenase complex) (Pyruvate dehydrogenase complex component E2) (PDC-E2) (PDCE2)	Dlat	235339	0.39136	0.76143	0.11837	0.83573	24.8742025	24.875385	24.951185	24.8684275
Q9EPN1	Neurobeachin (Lysosomal-trafficking regulator 2)	Nbea Lyst2	26422	0.38628	0.76491	0.11639	0.8383	21.286075	21.26198	21.2531	21.20773
Q9QZ06	Toll-interacting protein	Tollip	54473	0.38573	0.76529	0.11618	0.8383	20.176985	20.250715	20.11929	20.05846
Q9ESW4	Acylglycerol kinase, mitochondrial (EC 2.7.1.107) (EC 2.7.1.138) (EC 2.7.1.94) (Multiple substrate lipid kinase) (MuLK) (Multi-substrate lipid kinase)	Agk Mulk	69923	0.38472	0.76598	0.11578	0.83846	20.89466	20.8561425	20.91755	20.8200425

Q91ZZ3	Beta-synuclein	Sncb	104069	0.3823	0.76764	0.11484	0.83907	24.064055	24.1898725	23.9646075	24.059715
Q8C163	Nuclease EXOG, mitochondrial (EC 3.1.30.-) (Endonuclease G-like 1) (Endo G-like 1)	Exog Endogl1	208194	0.38281	0.76729	0.11504	0.83907	19.2674825	19.480965	19.52043	19.266945
Q91VD9	NADH-ubiquinone oxidoreductase 75 kDa subunit, mitochondrial (EC 1.6.99.3) (EC 7.1.1.2) (Complex I-75kD) (CI-75kD)	Ndufs1	227197	0.38108	0.76848	0.11437	0.83939	25.098745	25.126235	25.10344	25.0924825
Q61553	Fascin (Singed-like protein)	Fscn1 Fan1 Snl	14086	0.37825	0.77042	0.11327	0.84079	24.2745275	24.3306175	24.350455	24.3706
O88545	COP9 signalosome complex subunit 6 (SGN6) (Signalosome subunit 6) (JAB1-containing signalosome subunit 6)	Cops6 Csn6	26893	0.37761	0.77086	0.11303	0.84079	19.9330625	20.0436525	19.95117	20.021525
Q9Z204	Heterogeneous nuclear ribonucleoproteins C1/C2 (hnRNP C1/C2)	Hnrnpc Hnrpc	15381	0.37633	0.77174	0.11253	0.84115	21.6754275	21.6878325	21.736215	21.662215
Q91VZ6	Stromal membrane-associated protein 1	Smap1	98366	0.3755	0.77231	0.11221	0.84117	20.2430525	20.2777725	20.189675	20.2452325
Q00493	Carboxypeptidase E (CPE) (EC 3.4.17.10) (Carboxypeptidase H) (CPH) (Enkephalin convertase) (Prohormone-processing carboxypeptidase)	Cpe	12876	0.37454	0.77297	0.11184	0.84129	21.6665425	21.765545	21.7879275	21.89679
Q91YT0	NADH dehydrogenase [ubiquinone] flavoprotein 1, mitochondrial (EC 1.6.99.3) (EC 7.1.1.2) (Complex I-51kD) (CI-51kD) (NADH-ubiquinone oxidoreductase 51 kDa subunit)	Ndufv1	17995	0.37279	0.77418	0.11116	0.8414	24.0835175	24.119575	24.14266	24.1387825
P32067	Lupus La protein homolog (La autoantigen homolog) (La ribonucleoprotein)	Ssb Ss-b	20823	0.37358	0.77363	0.11147	0.8414	20.4100925	20.46162	20.4722575	20.4736425
P60766	Cell division control protein 42 homolog (EC 3.6.5.2) (G25K GTP-binding protein)	Cdc42	12540	0.37052	0.77574	0.11029	0.8425	22.9089975	22.9695625	22.92013	22.96993
P70372	ELAV-like protein 1 (Elav-like generic protein) (Hu-antigen R) (HuR) (MelG)	Elavl1 Elra Hua	15568	0.36524	0.77938	0.10825	0.84371	20.4509625	20.342415	20.402315	20.3684725
P97300	Neuroplastin (Stromal cell-derived receptor 1) (SDR-1)	Nptn Sdfr1 Sdr1	20320	0.36588	0.77893	0.1085	0.84371	24.3706425	24.362005	24.302915	24.3058875
Q8R5J9	PRA1 family protein 3 (ADP-ribosylation factor-like protein 6-interacting protein 5) (ARL-6-interacting protein 5) (Aip-5) (Addicsin) (GTRAP3-18) (Glutamate transporter EAAC1-interacting protein) (Prenylated Rab acceptor protein 2) (Protein JWa)	Arl6ip5 Aip5 Jwa Pra2 Praf3	65106	0.3649	0.77961	0.10812	0.84371	20.5166	20.535155	20.5359	20.6083975
P56383	ATP synthase F(0) complex subunit C2, mitochondrial (ATP synthase lipid-binding protein) (ATP synthase membrane subunit c locus 2) (ATP synthase proteolipid P2) (ATPase protein 9) (ATPase subunit c)	Atp5mc2 Atp5g2	67942	0.36574	0.77903	0.10845	0.84371	19.9981775	19.9910375	20.37317	19.779095
Q91WD5	NADH dehydrogenase [ubiquinone] iron-sulfur protein 2, mitochondrial (EC 1.6.99.3) (EC 7.1.1.2) (Complex I-49kD) (CI-49kD) (NADH-ubiquinone oxidoreductase 49 kDa subunit)	Ndufs2	226646	0.36683	0.77828	0.10887	0.84371	23.36892	23.3880075	23.4095525	23.4245575
Q811D0	Disks large homolog 1 (Embryo-dlg/synapse-associated protein 97) (E-dlg/SAP97) (Synapse-associated protein 97) (SAP-97) (SAP97)	Dlg1 Dlgh1	13383	0.36355	0.78054	0.1076	0.84411	20.8595475	20.926785	20.940135	20.9951125
P45591	Cofilin-2 (Cofilin, muscle isoform)	Cfl2	12632	0.35978	0.78315	0.10616	0.84633	21.0382775	21.08739	21.1742475	21.1000725
Q8BI08	Protein MAL2	Mal2	105853	0.35711	0.785	0.10513	0.84773	21.2568925	21.1720725	21.1971775	21.1444
Q8VE70	Programmed cell death protein 10 (TF-1 cell apoptosis-related protein 15)	Pdcd10 Tfar15	56426	0.35528	0.78626	0.10443	0.8479	26.10321	26.040915	26.17983	26.0960675
Q8R4V1	NFAT activation molecule 1 (Calcineurin/NFAT-activating ITAM-containing protein) (NFAT-activating protein with ITAM motif 1)	Nfam1 Cnaip	74039	0.35585	0.78587	0.10465	0.8479	19.36604	19.246115	19.247975	19.20915
Q8BJU0	Small glutamine-rich tetratricopeptide repeat-containing protein alpha (Alpha-SGT)	Sgta Sgt	52551	0.35069	0.78944	0.10268	0.85073	21.32711	21.2196975	21.28239	21.2514725

P24369	Peptidyl-prolyl cis-trans isomerase B (PPIase B) (EC 5.2.1.8) (CYP-S1) (Cyclophilin B) (Rotamase B) (S-cyclophilin) (SCYLP)	Ppib	19035	0.34776	0.79148	0.10156	0.85232	22.9052375	22.98391	22.902515	23.047785
Q9CZ04	COP9 signalosome complex subunit 7a (SGN7a) (Signalosome subunit 7a) (JAB1-containing signalosome subunit 7a)	Cops7a Csn7a	26894	0.3467	0.79221	0.10116	0.85251	19.58784	19.6980225	19.637775	19.89468
Q8BNY6	Neuronal calcium sensor 1 (NCS-1) (Frequenin homolog)	Ncs1 Freq	14299	0.34304	0.79476	0.099766	0.8532	21.5975825	21.53693	21.5161075	21.47781
P31938	Dual specificity mitogen-activated protein kinase kinase 1 (MAP kinase kinase 1) (MAPKK 1) (EC 2.7.12.2) (ERK activator kinase 1) (MAPK/ERK kinase 1) (MEK 1)	Map2k1 Mek1 Prkmk1	26395	0.34338	0.79452	0.099897	0.8532	22.9384675	23.015085	22.9575525	22.9757975
Q9CQV6	Microtubule-associated proteins 1A/1B light chain 3B (Autophagy-related protein LC3 B) (Autophagy-related ubiquitin-like modifier LC3 B) (MAP1 light chain 3-like protein 2) (MAP1A/MAP1B light chain 3 B) (MAP1A/MAP1B LC3 B) (Microtubule-associated protein 1 light chain 3 beta)	Map1lc3b Map1alc3 Map1lc3	67443	0.34403	0.79407	0.10014	0.8532	21.1193525	21.120565	21.196585	21.085765
P62983	Ubiquitin-40S ribosomal protein S27a (Ubiquitin carboxyl extension protein 80) [Cleaved into: Ubiquitin; 40S ribosomal protein S27a]	Rps27a Uba80 Ubcep1	78294	0.34256	0.79509	0.099584	0.8532	27.190275	27.2029575	27.1855475	27.252485
Q8R5C5	Beta-centractin (Actin-related protein 1B) (ARP1B)	Actr1b	226977	0.33757	0.79856	0.097693	0.85632	21.2930825	21.3509275	21.36147	21.3841275
P09405	Nucleolin (Protein C23)	Ncl Nuc	17975	0.33548	0.80002	0.096902	0.85728	21.8692175	21.900985	21.9180025	21.9554425
Q3THE2	Myosin regulatory light chain 12B (Myosin regulatory light chain 2-B, smooth muscle isoform) (Myosin regulatory light chain 20 kDa) (MLC20) (Myosin regulatory light chain MRLC2)	Myl12b Mrlc2 Mylc2b	67938	0.33282	0.80187	0.095898	0.85866	22.1660975	22.248965	22.271075	22.305495
P11087	Collagen alpha-1(I) chain (Alpha-1 type I collagen)	Col1a1 Cola1	12842	0.32904	0.8045	0.094474	0.86088	17.6527625	17.7820475	17.946375	17.43151
P97797	Tyrosine-protein phosphatase non-receptor type substrate 1 (SHP substrate 1) (SHPS-1) (Brain Ig-like molecule with tyrosine-based activation motifs) (Bit) (CD172 antigen-like family member A) (Inhibitory receptor SHPS-1) (MyD-1 antigen) (Signal-regulatory protein alpha-1) (Sirp-alpha-1) (mSIRP-alpha1) (p84) (CD antigen CD172a)	Sirpa Bit Myd1 Ptpns1 Shps1 Sirp	19261	0.32804	0.8052	0.094096	0.86102	23.5484025	23.6746675	23.671795	23.6628575
P68033	Actin, alpha cardiac muscle 1 (Alpha-cardiac actin) [Cleaved into: Actin, alpha cardiac muscle 1, intermediate form]	Actc1 Actc	11464	0.32103	0.81009	0.091467	0.86444	23.24343	23.15575	23.0865125	23.06605
Q8CHH9	Septin-8	Septin8 Kiaa0202 Sept8	20362	0.32129	0.80991	0.091564	0.86444	22.406195	22.5592475	22.52275	22.5325975
Q9D0T1	NHP2-like protein 1 (Fertilization antigen 1) (FA-1) (High mobility group-like nuclear protein 2 homolog 1) (Sperm-specific antigen 1) (U4/U6.U5 small nuclear ribonucleoprotein SNU13) (U4/U6.U5 tri-snRNP 15.5 kDa protein) [Cleaved into: NHP2-like protein 1, N-terminally processed]	Snu13 Nhp2l1 Ssfa1	100862468 20826	0.32104	0.81009	0.091469	0.86444	21.0049975	21.02832	21.02662	20.9313475
P63242	Eukaryotic translation initiation factor 5A-1 (eIF-5A-1) (eIF-5A1) (Eukaryotic initiation factor 5A isoform 1) (eIF-5A) (eIF-4D)	Eif5a	276770	0.31836	0.81196	0.090468	0.86582	22.6781125	22.631975	22.628085	22.6681925
Q6WVG3	BTB/POZ domain-containing protein KCTD12 (Pfetin) (Predominantly fetal expressed T1 domain)	Kctd12 Pfet1	239217	0.31586	0.8137	0.089535	0.86708	21.4156725	21.48727	21.412785	21.36917
P26645	Myristoylated alanine-rich C-kinase substrate (MARCKS)	Marcks Macs	17118	0.31338	0.81544	0.08861	0.86771	23.26914	23.32491	23.3032375	23.1801525
P14576	Signal recognition particle 54 kDa protein (SRP54)	Srp54	24067 665155	0.31421	0.81486	0.088916	0.86771	18.347555	18.765525	18.6782275	18.7577025
P08551	Neurofilament light polypeptide (NF-L) (68 kDa neurofilament protein) (Neurofilament triplet L protein)	Nefl Nf68 Nfl	18039	0.30851	0.81884	0.086798	0.87034	25.483485	25.35305	25.34114	25.4803525

P61082	NEDD8-conjugating enzyme Ubc12 (EC 2.3.2.-) (NEDD8 carrier protein) (Ubiquitin-conjugating enzyme E2 M)	Ube2m Ubc-rs2 Ubc12	22192	0.30823	0.81904	0.086693	0.87034	21.44652	21.392455	21.4486575	21.39947
P00416	Cytochrome c oxidase subunit 3 (EC 1.9.3.1) (Cytochrome c oxidase polypeptide III)	mt-Co3 COIII Mtco3	17710	0.30559	0.82089	0.085717	0.87064	19.06617	19.6977675	19.2784075	19.1093125
Q01405	Protein transport protein Sec23A (SEC23-related protein A)	Sec23a Sec23 Sec23r	20334	0.30457	0.8216	0.085338	0.87064	20.106885	20.2721825	20.31946	20.2100075
P17809	Solute carrier family 2, facilitated glucose transporter member 1 (Glucose transporter type 1, erythrocyte/brain) (GLUT-1) (GT1)	Slc2a1 Glut1	20525	0.30556	0.82091	0.085706	0.87064	21.2051625	21.268695	21.2624975	21.2122925
P70414	Sodium/calcium exchanger 1 (Na(+)/Ca(2+)-exchange protein 1) (Solute carrier family 8 member 1)	Slc8a1 Ncx	20541	0.30489	0.82138	0.085455	0.87064	21.1355525	21.14228	21.17033	21.0774625
P17426	AP-2 complex subunit alpha-1 (100 kDa coated vesicle protein A) (Adaptor protein complex AP-2 subunit alpha-1) (Adaptor-related protein complex 2 subunit alpha-1) (Alpha-adaptin A) (Alpha1-adaptin) (Clathrin assembly protein complex 2 alpha-A large chain) (Plasma membrane adaptor HA2/AP2 adaptin alpha A subunit)	Ap2a1 Adtaa Clapa1	11771	0.3024	0.82313	0.084534	0.87165	24.935815	24.924885	24.9688925	24.9248975
Q8R366	Immunoglobulin superfamily member 8 (IgSF8) (CD81 partner 3) (Glu-Trp-Ile EWI motif-containing protein 2) (EWI-2) (Keratinocyte-associated transmembrane protein 4) (KCT-4) (Prostaglandin regulatory-like protein) (PGRL) (CD antigen CD316)	Igsf8 Ewi2 Kct4 Pgrl	140559	0.2945	0.82866	0.081626	0.87689	23.02631	22.9482525	22.9640675	22.94761
P60761	Neurogranin (Ng) (RC3) [Cleaved into: NEUG(55-78)]	Nrgn	64011	0.28772	0.83341	0.079142	0.88077	22.678355	22.7197975	22.4658625	22.14665
Q8VD37	SH3-containing GRB2-like protein 3-interacting protein 1 (Endophilin-3-interacting protein)	Sgip1	73094	0.28762	0.83348	0.079107	0.88077	21.2626025	21.34778	21.303525	21.2895775
Q62167	ATP-dependent RNA helicase DDX3X (EC 3.6.4.13) (D1Pas1-related sequence 2) (DEAD box RNA helicase DEAD3) (mDEAD3) (DEAD box protein 3, X-chromosomal) (Embryonic RNA helicase)	Ddx3x D1Pas1-rs2 Ddx3 Dead3 Erh	13205	0.2824	0.83713	0.077205	0.88309	21.3843175	21.3936425	21.4051575	21.415315
Q99MN9	Propionyl-CoA carboxylase beta chain, mitochondrial (PCCase subunit beta) (EC 6.4.1.3) (Propanoyl-CoA:carbon dioxide ligase subunit beta)	Pccb	66904	0.28283	0.83684	0.07736	0.88309	21.3740525	21.3782525	21.30188	21.29359
Q9DCD0	6-phosphogluconate dehydrogenase, decarboxylating (EC 1.1.1.44)	Pgd	110208	0.28202	0.8374	0.077068	0.88309	21.393755	21.41152	21.412645	21.48918
P00493	Hypoxanthine-guanine phosphoribosyltransferase (HGPRT) (HGPRTase) (EC 2.4.2.8) (HPRT B)	Hprt1 Hprt	15452	0.27991	0.83888	0.0763	0.88348	22.44554	22.481055	22.52067	22.4899525
Q9Z1W9	STE20/SPS1-related proline-alanine-rich protein kinase (Ste-20-related kinase) (EC 2.7.11.1) (Serine/threonine-protein kinase 39)	Stk39 Spak	53416	0.27984	0.83893	0.076276	0.88348	19.28176	19.226455	19.2022775	19.2056575
P40240	CD9 antigen (CD antigen CD9)	Cd9	12527	0.27413	0.84293	0.07421	0.88708	20.8642075	20.742035	20.839485	20.705845
Q9CQQ7	ATP synthase F(0) complex subunit B1, mitochondrial (ATP synthase peripheral stalk-membrane subunit b) (ATP synthase subunit b) (ATPase subunit b)	Atp5pb Atp5f1	11950	0.26868	0.84675	0.072247	0.89048	24.675705	24.6822525	24.6225125	24.62884
Q9D164	FXYD domain-containing ion transport regulator 6 (PLM-like protein) (Phosphohippolin)	Fxyd6 Plp	59095	0.26374	0.8502	0.070477	0.8935	20.13289	19.7484175	19.978865	20.092495
P24549	Retinal dehydrogenase 1 (RALDH 1) (RalDH1) (EC 1.2.1.-) (EC 1.2.1.36) (ALDH-E1) (ALHDII) (Aldehyde dehydrogenase family 1 member A1) (Aldehyde dehydrogenase, cytosolic)	Aldh1a1 Ahd-2 Ahd2 Aldh1	11668	0.26043	0.85252	0.069296	0.8938	19.7179025	19.5328775	19.5892125	19.6205775
O08553	Dihydropyrimidinase-related protein 2 (DRP-2) (Unc-33-like phosphoprotein 2) (ULIP-2)	Dpysl2 Crmp2 Ulip2	12934	0.26216	0.85131	0.069912	0.8938	28.486345	28.5143575	28.53919	28.4953725
Q61171	Peroxiredoxin-2 (EC 1.11.1.15) (Thiol-specific antioxidant protein) (TSA) (Thioredoxin	Prdx2 Tdpx1 Tpx	21672	0.26023	0.85266	0.069226	0.8938	24.823985	24.8506025	24.86521	24.8586225

	peroxidase 1) (Thioredoxin-dependent peroxide reductase 1)										
Q9R0P5	Destrin (Actin-depolymerizing factor) (ADF) (Sid 23)	Dstn Dsn Sid23	56431	0.25999	0.85282	0.069141	0.8938	23.5498925	23.583215	23.52564	23.56926
Q791T5	Mitochondrial carrier homolog 1 (Mitochondrial carrier-like protein 1)	Mtch1	56462	0.25819	0.85408	0.0685	0.89389	21.0648075	21.003135	21.0332725	20.9764875
Q9DCH4				0.25846	0.8539	0.068593	0.89389	18.124325	18.4569275	17.8767275	17.961275
Q8R1I1	Cytochrome b-c1 complex subunit 9 (Complex III subunit 9) (Complex III subunit X) (Cytochrome c1 non-heme 7 kDa protein) (Ubiquinol-cytochrome c reductase complex 7.2 kDa protein)	Uqcr10	66152	0.25619	0.85548	0.067789	0.89474	22.6349	22.547375	22.5816875	22.62805
P70398	Probable ubiquitin carboxyl-terminal hydrolase FAF-X (EC 3.4.19.12) (Deubiquitinating enzyme FAF-X) (Fat facets homolog) (Fat facets protein-related, X-linked) (Ubiquitin carboxyl-terminal hydrolase FAM) (Ubiquitin thioesterase FAF-X) (Ubiquitin-specific protease 9, X chromosome) (Ubiquitin-specific-processing protease FAF-X)	Usp9x Fafl Fam	22284	0.25104	0.85908	0.065967	0.89789	20.5844975	20.3794875	20.480615	20.5049225
P12023	Amyloid-beta A4 protein (ABPP) (APP) (Alzheimer disease amyloid A4 protein homolog) (Amyloid precursor protein) (Amyloid-beta precursor protein) (Amyloidogenic glycoprotein) (AG) [Cleaved into: N-APP; Soluble APP-alpha (S-APP-alpha); Soluble APP-beta (S-APP-beta); C99 (APP-C99) (Beta-secretase C-terminal fragment) (Beta-CTF); Amyloid-beta protein 42 (Abeta42) (Beta-APP42); Amyloid-beta protein 40 (Abeta40) (Beta-APP40); C83 (Alpha-secretase C-terminal fragment) (Alpha-CTF); P3(42); P3(40); C80; Gamma-secretase C-terminal fragment 59 (APP-C59) (Amyloid intracellular domain 59) (AID(59)) (Gamma-CTF(59)); Gamma-secretase C-terminal fragment 57 (APP-C57) (Amyloid intracellular domain 57) (AID(57)) (Gamma-CTF(57)); Gamma-secretase C-terminal fragment 50 (Amyloid intracellular domain 50) (AID(50)) (Gamma-CTF(50)); C31]	App	11820	0.2482	0.86106	0.064966	0.89934	20.214185	20.1832125	20.196755	20.2543125
P46638	Ras-related protein Rab-11B	Rab11b	19326	0.24394	0.86403	0.063471	0.9015	22.6108425	22.6321175	22.6246625	22.66551
P99024	Tubulin beta-5 chain	Tubb5	22154	0.23977	0.86694	0.062013	0.9015	24.9286175	24.9257225	24.938825	24.9688475
O88343	Electrogenic sodium bicarbonate cotransporter 1 (Sodium bicarbonate cotransporter) (Na(+)/HCO3(-) cotransporter) (Solute carrier family 4 member 4)	Slc4a4 Nbc1 Nbce1	54403	0.2393	0.86726	0.061849	0.9015	22.9413675	22.95245	22.887925	22.9039325
Q8R317	Ubiquilin-1 (Protein linking IAP with cytoskeleton 1) (PLIC-1)	Ubqln1 Plic1	56085	0.24121	0.86593	0.062516	0.9015	19.9843475	19.9084625	19.984315	19.8741
Q9D1D4	Transmembrane emp24 domain-containing protein 10 (21 kDa transmembrane-trafficking protein) (Transmembrane protein Tmp21) (p24 family protein delta-1) (p24delta1)	Tmed10 Tmp21	68581	0.23983	0.86689	0.062035	0.9015	19.987285	19.8654725	19.88185	19.8783925
Q8BU30	Isoleucine--tRNA ligase, cytoplasmic (EC 6.1.1.5) (Isoleucyl-tRNA synthetase) (IRS) (IleRS)	Iars1 Iars	105148	0.24231	0.86517	0.062898	0.9015	18.99611	19.114965	19.1270875	19.0639625
Q03734	Serine protease inhibitor A3M (Serpin A3M)	Serpina3m	20717	0.24007	0.86672	0.062119	0.9015	17.6897075	18.2327025	17.554705	17.9670525
B2RSH2	Guanine nucleotide-binding protein G(i) subunit alpha-1 (Adenylate cyclase-inhibiting G alpha protein)	Gnai1 Gnai-1	14677	0.23743	0.86857	0.061197	0.90224	22.9591025	23.023925	22.9580875	22.9614575

Q64433	10 kDa heat shock protein, mitochondrial (Hsp10) (10 kDa chaperonin) (Chaperonin 10) (CPN10)	Hspe1	15528	0.22984	0.87384	0.05857	0.9071	24.2313775	24.19514	24.1984625	24.2029375
P14733	Lamin-B1	Lmnb1	16906	0.22651	0.87614	0.057425	0.9086	21.094985	21.0732875	21.1398	21.0357175
P63330	Serine/threonine-protein phosphatase 2A catalytic subunit alpha isoform (PP2A-alpha) (EC 3.1.3.16)	Ppp2ca	19052	0.22604	0.87647	0.057264	0.9086	20.267925	20.19728	20.3408175	20.24262
P53994	Ras-related protein Rab-2A	Rab2a Rab2	59021	0.22117	0.87984	0.055596	0.91148	23.7087625	23.7337925	23.77081	23.7561825
P51881	ADP/ATP translocase 2 (ADP,ATP carrier protein 2) (Adenine nucleotide translocator 2) (ANT 2) (Solute carrier family 25 member 5) [Cleaved into: ADP/ATP translocase 2, N-terminally processed]	Slc25a5 Ant2	11740	0.22003	0.88063	0.055208	0.91167	25.718035	25.7725625	25.7252725	25.752675
Q9CY58	Plasminogen activator inhibitor 1 RNA-binding protein (PAI1 RNA-binding protein 1) (PAI-RBP1) (SERPINE1 mRNA-binding protein 1)	Serbp1 Pairbp1	66870	0.21723	0.88256	0.054257	0.91305	20.526285	20.4842925	20.57399	20.62193
P24527	Leukotriene A-4 hydrolase (LTA-4 hydrolase) (EC 3.3.2.6) (Leukotriene A(4) hydrolase)	Lta4h	16993	0.21268	0.88569	0.052719	0.91535	21.2056875	21.23765	21.26198	21.284605
Q3TXS7	26S proteasome non-ATPase regulatory subunit 1 (26S proteasome regulatory subunit RPN2) (26S proteasome regulatory subunit S1)	Psmd1	70247	0.21227	0.88597	0.052579	0.91535	19.9254725	20.0493775	20.0878175	19.9831725
P70704	Phospholipid-transporting ATPase IA (EC 7.6.2.1) (ATPase class I type 8A member 1) (Chromaffin granule ATPase II) (P4-ATPase flippase complex alpha subunit ATP8A1)	Atp8a1 Atpc1	11980	0.21018	0.88741	0.051876	0.91621	21.9488325	22.00128	22.0590625	22.0799
Q9D8B4	NADH dehydrogenase [ubiquinone] 1 alpha subcomplex subunit 11 (Complex I-B14.7) (CI-B14.7) (NADH-ubiquinone oxidoreductase subunit B14.7)	Ndufa11		0.20789	0.88898	0.051107	0.91667	20.904905	20.9029175	21.0044875	20.9062425
P05201	Aspartate aminotransferase, cytoplasmic (cAspAT) (EC 2.6.1.1) (EC 2.6.1.3) (Cysteine aminotransferase, cytoplasmic) (Cysteine transaminase, cytoplasmic) (cCAT) (Glutamate oxaloacetate transaminase 1) (Transaminase A)	Got1	14718	0.2069	0.88966	0.050776	0.91667	26.528695	26.54573	26.56491	26.540885
P62869	Elongin-B (EloB) (Elongin 18 kDa subunit) (RNA polymerase II transcription factor SIII subunit B) (SIII p18) (Transcription elongation factor B polypeptide 2)	Elob Tceb2	67673	0.20708	0.88953	0.050838	0.91667	20.746945	20.7555325	20.707865	20.72223
Q9WTP7	GTP:AMP phosphotransferase AK3, mitochondrial (EC 2.7.4.10) (Adenylate kinase 3) (AK 3) (Adenylate kinase 3 alpha-like 1)	Ak3 Ak3l Ak3l1 Akl3l	56248	0.20305	0.8923	0.049491	0.91877	21.182105	21.19128	21.1911425	21.071525
Q99JR1	Sideroflexin-1	Sfxn1	14057	0.20184	0.89312	0.049089	0.919	21.522165	21.5482375	21.5048175	21.5040325
Q9D855	Cytochrome b-c1 complex subunit 7 (Complex III subunit 7) (Complex III subunit VII) (Ubiquinol-cytochrome c reductase complex 14 kDa protein)	Uqcrb		0.19803	0.89572	0.047827	0.92047	23.3720125	23.355225	23.366405	23.4006025
P02802	Metallothionein-1 (MT-1) (Metallothionein-I) (MT-I)	Mt1	17748	0.19798	0.89575	0.047813	0.92047	19.6392575	19.72689	19.484085	19.4041875
Q9CQC7	NADH dehydrogenase [ubiquinone] 1 beta subcomplex subunit 4 (Complex I-B15) (CI-B15) (NADH-ubiquinone oxidoreductase B15 subunit)	Ndufb4	100042503 68194	0.19222	0.89967	0.045917	0.92387	22.16079	22.13226	22.1584475	22.1117125
P19157	Glutathione S-transferase P 1 (Gst P1) (EC 2.5.1.18) (GST YF-YF) (GST class-pi) (GST-piB) (Preadipocyte growth factor)	Gstp1 Gstpib	14870	0.18754	0.90283	0.044392	0.9265	24.2376125	24.2540975	24.2272075	24.30209
P97333	Neuropilin-1 (A5 protein) (CD antigen CD304)	Nrp1 Nrp	18186	0.18577	0.90403	0.043815	0.92711	18.53438	18.514625	18.709045	18.4992175
Q91V61	Sideroflexin-3	Sfxn3	94280	0.18228	0.90638	0.042688	0.92834	23.981885	23.9627	24.0037125	23.9718375
Q8CIB5	Fermitin family homolog 2 (Kindlin-2) (Pleckstrin homology domain-containing family C member 1)	Fermt2 Plekhc1	218952	0.18217	0.90646	0.042653	0.92834	19.228705	19.2421	19.22375	19.289425

Q9ESN6	Tripartite motif-containing protein 2 (EC 2.3.2.27) (E3 ubiquitin-protein ligase TRIM2) (Neural activity-related RING finger protein) (RING-type E3 ubiquitin transferase TRIM2)	Trim2 Kiaa0517 Narf	80890	0.17884	0.90869	0.041585	0.93	20.9954525	21.0309175	21.0610525	21.0287875
P21278	Guanine nucleotide-binding protein subunit alpha-11 (G alpha-11) (G-protein subunit alpha-11)	Gna11 Gna-11	14672	0.15874	0.92201	0.035264	0.94287	20.432995	20.43077	20.44015	20.3831425
Q922H2	[Pyruvate dehydrogenase (acetyl-transferring)] kinase isozyme 3, mitochondrial (EC 2.7.11.2) (Pyruvate dehydrogenase kinase isoform 3)	Pdk3	236900	0.158	0.92249	0.035038	0.94287	19.83789	19.83052	19.7878	19.77622
Q9CZC8	Secernin-1	Scrn1 Kiaa0193	69938	0.15387	0.92518	0.033772	0.94499	22.67568	22.66774	22.6563725	22.681365
Q8C437	PEX5-related protein (PEX2-related protein) (PEX5-like protein) (Peroxin-5-related protein) (Tetratricopeptide repeat-containing Rab8b-interacting protein) (Pex5Rp) (TRIP8b)	Pex5l Pex2 Pex5r Pxr2	58869	0.15008	0.92764	0.032619	0.94687	18.702495	18.6178525	18.4632675	18.3733725
B2RXA1	PI-PLC X domain-containing protein 2	Plcxd2	433022	0.14418	0.93144	0.030845	0.95011	20.878975	20.7952475	20.796095	20.8143325
P48758	Carbonyl reductase [NADPH] 1 (EC 1.1.1.184) (15-hydroxyprostaglandin dehydrogenase [NADP(+)]) (EC 1.1.1.197) (NADPH-dependent carbonyl reductase 1) (Prostaglandin 9-ketoreductase) (Prostaglandin-E(2) 9-reductase) (EC 1.1.1.189)	Cbr1 Cbr	12408	0.14032	0.9339	0.029698	0.95191	22.914235	22.91664	22.93821	22.9072875
Q9D892	Inosine triphosphate pyrophosphatase (ITPase) (Inosine triphosphatase) (EC 3.6.1.9) (Non-canonical purine NTP pyrophosphatase) (Non-standard purine NTP pyrophosphatase) (Nucleoside-triphosphate diphosphatase) (Nucleoside-triphosphate pyrophosphatase) (NTPase)	Itpa	16434	0.13944	0.93446	0.029439	0.95191	20.5300775	20.5962425	20.594655	20.6273175
P40237	CD82 antigen (C33 antigen) (IA4) (Inducible membrane protein R2) (Metastasis suppressor Kangai-1 homolog) (CD antigen CD82)	Cd82 Kai1	12521	0.1378	0.9355	0.028956	0.95212	21.0146675	20.9848925	20.889295	20.91934
Q3UGR5	Haloacid dehalogenase-like hydrolase domain-containing protein 2	Hdhd2	76987	0.13716	0.93591	0.028767	0.95212	21.1284875	21.0712575	21.1228725	21.06722
Q8JZU2	Tricarboxylate transport protein, mitochondrial (Citrate transport protein) (CTP) (Solute carrier family 25 member 1) (Tricarboxylate carrier protein)	Slc25a1	13358	0.12626	0.94273	0.025612	0.95842	19.31757	19.25683	19.3021625	19.3279625
P62880	Guanine nucleotide-binding protein G(I)/G(S)/G(T) subunit beta-2 (G protein subunit beta-2) (Transducin beta chain 2)	Gnb2	14693	0.12453	0.9438	0.025122	0.95887	24.5085825	24.533215	24.54736	24.5335225
Q9CX34	Protein SGT1 homolog (Suppressor of G2 allele of SKP1 homolog)	Sugt1	67955	0.12077	0.94611	0.024058	0.96058	20.5076425	20.491855	20.532935	20.496095
P68368	Tubulin alpha-4A chain (Alpha-tubulin 4) (Alpha-tubulin isotype M-alpha-4) (Tubulin alpha-4 chain)	Tuba4a Tuba4	22145	0.11792	0.94784	0.023263	0.96106	26.8300925	26.8898425	26.8874775	26.8499275
Q9QZQ8	Core histone macro-H2A.1 (Histone macroH2A1) (mH2A1) (H2A.y) (H2A/y)	Macroh2a1 H2afy	26914	0.11848	0.9475	0.02342	0.96106	21.60403	21.64233	21.5942375	21.6592625
Q8K183	Pyridoxal kinase (EC 2.7.1.35) (Pyridoxine kinase)	Pdxk Pkh	216134	0.1082	0.95368	0.020599	0.96633	23.08564	23.066175	23.0487925	23.066035
Q91VM9	Inorganic pyrophosphatase 2, mitochondrial (EC 3.6.1.1) (Pyrophosphate phospho-hydrolase 2) (PPase 2)	Ppa2	74776	0.087699	0.96544	0.015276	0.97695	19.830515	19.8677325	19.8196325	19.78746
Q8BL97	Serine/arginine-rich splicing factor 7 (Splicing factor, arginine/serine-rich 7)	Srsf7 Sfrs7	225027	0.088046	0.96525	0.015362	0.97695	21.0721625	21.056	21.1015125	21.0234225
Q9CQH3	NADH dehydrogenase [ubiquinone] 1 beta subcomplex subunit 5, mitochondrial (Complex I-SGDH) (CI-SGDH) (NADH-ubiquinone oxidoreductase SGDH subunit)	Ndufb5	66046	0.076806	0.97134	0.012631	0.98114	22.67337	22.6352425	22.6407325	22.6641
P62320	Small nuclear ribonucleoprotein Sm D3 (Sm-D3) (snRNP core protein D3)	Snrpd3	67332	0.076495	0.9715	0.012558	0.98114	20.6909675	20.6455	20.6847325	20.6720175

P70663	SPARC-like protein 1 (Extracellular matrix protein 2) (Matrix glycoprotein Sc1)	Sparcl1 Ecm2 Sc1	13602	0.077714	0.97085	0.012846	0.98114	18.770045	18.83408	18.916805	18.7463825
P05202	Aspartate aminotransferase, mitochondrial (mAspAT) (EC 2.6.1.1) (EC 2.6.1.7) (Fatty acid-binding protein) (FABP-1) (Glutamate oxaloacetate transaminase 2) (Kynurenine aminotransferase 4) (Kynurenine aminotransferase IV) (Kynurenine--oxoglutarate transaminase 4) (Kynurenine--oxoglutarate transaminase IV) (Plasma membrane-associated fatty acid-binding protein) (FABPpm) (Transaminase A)	Got2 Got-2	14719	0.074231	0.97269	0.012026	0.98149	26.7814425	26.7709	26.7823225	26.76111
Q8VED9	Galectin-related protein (Galectin-related protein A) (Lectin galactoside-binding-like protein A)	Lgalsl Grpa Lgalsla	216551	0.07338	0.97313	0.011829	0.98149	21.0993575	21.0661025	21.0965525	21.06263
Q03265	ATP synthase subunit alpha, mitochondrial (ATP synthase F1 subunit alpha)	Atp5f1a Atp5a1	11946	0.069931	0.97491	0.011036	0.98198	28.2688325	28.27907	28.269915	28.27969
O08919	Numb-like protein	Numbl Nbl	18223	0.070051	0.97485	0.011063	0.98198	20.8565975	21.003495	21.067175	20.989295
Q91YR1	Twinfilin-1 (Protein A6)	Twf1 Ptk9	19230	0.063069	0.97835	0.0095069	0.9848	20.47486	20.494575	20.43859	20.5085875
Q9WV55	Vesicle-associated membrane protein-associated protein A (VAMP-A) (VAMP-associated protein A) (VAP-A) (33 kDa VAMP-associated protein) (VAP-33)	Vapa Vap33	30960	0.057288	0.98114	0.008271	0.98695	22.5404975	22.5309475	22.5690925	22.545615
Q8C0L0	Thioredoxin-related transmembrane protein 4 (Thioredoxin domain-containing protein 13)	Tmx4 D2Bwg1356e Kiaa1162 Txndc13	52837	0.055801	0.98184	0.0079613	0.98701	19.7978925	19.8453425	19.779025	19.77908
P00158	Cytochrome b (Complex III subunit 3) (Complex III subunit III) (Cytochrome b-c1 complex subunit 3) (Ubiquinol-cytochrome-c reductase complex cytochrome b subunit)	Mt-Cyb Cob Cytb mt-Cytb Mtcyb	17711	0.048637	0.9851	0.006519	0.98964	20.3582625	20.3774025	20.39368	20.415625
Q9ER00	Syntaxin-12	Stx12	100226	0.040465	0.98859	0.0049832	0.99249	21.0170775	21.024435	21.0066925	21.0273525
Q9R1P1	Proteasome subunit beta type-3 (EC 3.4.25.1) (Proteasome chain 13) (Proteasome component C10-II) (Proteasome theta chain)	Psmb3	26446	0.036152	0.99032	0.0042246	0.99357	20.58314	20.6655425	20.6249075	20.6267875
O89053	Coronin-1A (Coronin-like protein A) (Clipin-A) (Coronin-like protein p57) (Tryptophan aspartate-containing coat protein) (TACO)	Coro1a Coro1	12721	0.033892	0.99119	0.0038426	0.99379	24.1433825	24.1585175	24.1281675	24.1509425
Q9CR51	V-type proton ATPase subunit G 1 (V-ATPase subunit G 1) (V-ATPase 13 kDa subunit 1) (Vacuolar proton pump subunit G 1)	Atp6v1g1 Atp6g1	66290	0.028573	0.99314	0.0029892	0.99428	20.145765	20.1475175	20.1260825	20.1667225
P83940	Elongin-C (EloC) (Elongin 15 kDa subunit) (RNA polymerase II transcription factor SIII subunit C) (SIII p15) (Stromal membrane-associated protein SMAP1B homolog) (Transcription elongation factor B polypeptide 1)	Eloc Tceb1	67923	0.029243	0.9929	0.003093	0.99428	20.8311775	20.792655	20.80854	20.8202875
Q99LY9	NADH dehydrogenase [ubiquinone] iron-sulfur protein 5 (Complex I-15 kDa) (CI-15 kDa) (NADH-ubiquinone oxidoreductase 15 kDa subunit)	Ndufs5	595136	0.027162	0.99363	0.0027742	0.99428	22.140165	22.12744	22.150495	22.144735
Q9CQD1	Ras-related protein Rab-5A (EC 3.6.5.2)	Rab5a nnyRab5a	271457	0.0040225	0.99963	0.00016176	0.99963	22.782405	22.77544	22.775515	22.777865
