# Supplementary material for: The Impacts of Surgery and Intracerebral Electrodes in C57BL/6J Mouse Kainate Model of Epileptogenesis: Seizure Threshold, Proteomics, and Cytokine Profiles
Source: Front Neurol. 2021 Jul 12;12:625017. doi: 10.3389/fneur.2021.625017 (PMC8312573; doi:10.3389/fneur.2021.625017)
Supplement: Supplementary Table 2 — The impact of intracerebral electrode implants on the expression of proteins in the hippocampus. The groups compared were between surgery vs. no surgery treated with vehicle (distilled water), and all the proteins that were significantly altered by p > 0.01 are listed and the proteins with >2-fold change are highlighted. [file Table_2.docx]

| **Uniprot ID** | **Gene**  **Symbol** | **Protein**  **names** | **Gene**  **names** | **KEGG-ID (mmu)** | **Fold change** | **log2(FC)** | **p value** | **neg log10(p)** |
| --- | --- | --- | --- | --- | --- | --- | --- | --- |
| P98086 | C1QA_MOUSE | Complement C1q subcomponent subunit A | C1qa | 12259 | 4.7473 | 2.2471 | 0.000337 | 3.4723 |
| Q02105 | C1QC_MOUSE | Complement C1q subcomponent subunit C | C1qc C1qg | 12262 | 4.2034 | 2.0715 | 0.000383 | 3.4169 |
| P14106 | C1QB_MOUSE | Complement C1q subcomponent subunit B | C1qb | 12260 | 3.4434 | 1.7838 | 0.000235 | 3.6293 |
| P20152 | VIME_MOUSE | Vimentin | Vim | 22352 | 2.8094 | 1.4903 | 0.00067 | 3.174 |
| P03995 | GFAP_MOUSE | Glial fibrillary acidic protein (GFAP) | Gfap | 14580 | 2.7743 | 1.4721 | 3.33E-06 | 5.4779 |
| Q8CIG9 | FBXL8_MOUSE | F-box and leucine-rich repeat protein 8 | Fbxl8 Fbl8 | 50788 | 2.3142 | 1.2105 | 0.000804 | 3.095 |
| P55012 | S12A2_MOUSE | Solute carrier family 12 member 2 (Basolateral Na-K-Cl symporter) | Slc12a2 Nkcc1 | 20496 | 2.1188 | 1.0833 | 6.57E-05 | 4.1824 |
| Q99L04 | DHRS1_MOUSE | Dehydrogenase/reductase SDR family member 1 | Dhrs1 D14ertd484e | 52585 | 2.0032 | 1.0023 | 0.000742 | 3.1294 |
| Q3URK3 | TET1_MOUSE | Methylcytosine dioxygenase TET1 (CXXC-6) | Tet1 Cxxc6 Kiaa1676 |  | 1.7901 | 0.84006 | 0.001172 | 2.931 |
| Q6P2B1 | TNPO3_MOUSE | Transportin-3 | Tnpo3 | 320938 | 1.7778 | 0.83013 | 0.0012 | 2.9208 |
| P16045 | LEG1_MOUSE | Galectin-1 (Gal-1) (14 kDa lectin/Galaptin) | Lgals1 Gbp | 16852 | 1.6891 | 0.75622 | 0.000323 | 3.4909 |
| Q8VHL1 | SETD7_MOUSE | Histone-lysine N-methyltransferase SETD7 | Setd7 Kiaa1717 Set7 Set9 | 73251 | 1.6064 | 0.68381 | 5.11E-05 | 4.2916 |
| Q5SSL4 | ABR_MOUSE | Active breakpoint cluster region-related protein | Abr | 109934 | 1.557 | 0.6388 | 0.001222 | 2.9129 |
| P35505 | FAAA_MOUSE | Fumarylacetoacetase (FAA) (Beta-diketonase) | Fah | 14085 | 1.5044 | 0.58915 | 0.008928 | 2.0493 |
| Q8R001 | MARE2_MOUSE | Microtubule-associated protein RP/EB family member 2 | Mapre2 | 212307 | 1.4923 | 0.57753 | 0.004346 | 2.3619 |
| P02468 | LAMC1_MOUSE | Laminin subunit gamma-1 (Laminin B2 chain) | Lamc1 Lamb-2 Lamc-1 |  | 1.4743 | 0.56004 | 0.008929 | 2.0492 |
| Q6PH08 | ERC2_MOUSE | ERC protein 2 (CAZ-associated structural protein 1) (CAST1) | Erc2 Cast1 D14Ertd171e Kiaa0378 | 238988 | 1.4199 | 0.5058 | 0.0061 | 2.2147 |
| Q9DB73 | NB5R1_MOUSE | NADH-cytochrome b5 reductase 1 (b5R.1) | Cyb5r1 Nqo3a2 | 72017 | 1.4114 | 0.49715 | 0.006531 | 2.185 |
| P54071 | IDHP_MOUSE | Isocitrate dehydrogenase [NADP], mitochondrial (IDH) | Idh2 | 269951 | 1.3875 | 0.47252 | 0.003895 | 2.4095 |
| Q9CQJ6 | DENR_MOUSE | Density-regulated protein (DRP) | Denr | 68184 | 1.3757 | 0.46018 | 0.000492 | 3.3076 |
| O08709 | PRDX6_MOUSE | Peroxiredoxin-6 (Antioxidant protein 2) (Non-selenium glutathione peroxidase) (NSGPx) | Prdx6 Aop2 Ltw4 Prdx5 | 11758 | 1.372 | 0.45626 | 0.000832 | 3.0799 |
| Q6PB66 | LPPRC_MOUSE | Leucine-rich PPR motif-containing protein, mitochondrial | Lrpprc Lrp130 | 72416 | 1.358 | 0.44145 | 0.000638 | 3.1952 |
| P56565 | S10A1_MOUSE | Protein S100-A1 (S100 calcium-binding protein A1) | S100a1 | 20193 | 1.3459 | 0.42857 | 0.003379 | 2.4712 |
| Q6NZJ6 | IF4G1_MOUSE | Eukaryotic translation initiation factor 4 gamma 1 | Eif4g1 | 208643 | 1.3393 | 0.42147 | 0.000411 | 3.386 |
| P26041 | MOES_MOUSE | Moesin (Membrane-organizing extension spike protein) | Msn | 17698 | 1.334 | 0.41579 | 0.004246 | 2.372 |
| Q8BGT8 | PHIPL_MOUSE | Phytanoyl-CoA hydroxylase-interacting protein-like | Phyhipl | 70911 | 1.3276 | 0.40883 | 0.004495 | 2.3473 |
| Q9CQ62 | DECR_MOUSE | 2,4-dienoyl-CoA reductase [NADPH]) (4-enoyl-CoA reductase [NADPH]) | Decr1 | 67460 | 1.3251 | 0.40614 | 0.000835 | 3.0785 |
| Q8BP92 | RCN2_MOUSE | Reticulocalbin-2 (TCBP-49) | Rcn2 | 26611 | 1.3184 | 0.39874 | 0.00321 | 2.4936 |
| Q9Z1Z0 | USO1_MOUSE | General vesicular transport factor p115 (Protein USO1 homolog) (Vesicle-docking protein) | Uso1 Vdp | 56041 | 1.3013 | 0.3799 | 0.008387 | 2.0764 |
| Q9CPY7 | AMPL_MOUSE | Cytosol aminopeptidase (EC 3.4.11.1) (Leucine aminopeptidase 3) (LAP-3) | Lap3 Lapep | 66988 | 1.2943 | 0.37219 | 0.004971 | 2.3035 |
| Q9WVA3 | BUB3_MOUSE | Mitotic checkpoint protein BUB3 | Bub3 | 12237 | 1.261 | 0.33457 | 0.000573 | 3.2421 |
| O70318 | E41L2_MOUSE | Band 4.1-like protein 2 (Generally expressed protein 4.1) (4.1G) | Epb41l2 Epb4.1l2 | 13822 | 1.2548 | 0.32741 | 0.006488 | 2.1879 |
| Q9QYG0 | NDRG2_MOUSE | Protein NDRG2 | Ndrg2 Kiaa1248 Ndr2 | 29811 | 1.2418 | 0.31244 | 0.000394 | 3.4045 |
| P10605 | CATB_MOUSE | Cathepsin B | Ctsb | 13030 | 1.2221 | 0.28942 | 0.002661 | 2.575 |
| Q5SRX1 | TM1L2_MOUSE | TOM1-like protein 2 (Target of Myb-like protein 2) | Tom1l2 | 216810 | 1.2206 | 0.28764 | 0.008536 | 2.0687 |
| P21460 | CYTC_MOUSE | Cystatin-C (Cystatin-3) | Cst3 | 13010 | 1.2088 | 0.27356 | 0.006292 | 2.2012 |
| P80314 | TCPB_MOUSE | T-complex protein 1 subunit beta (TCP-1-beta) (CCT-beta) | Cct2 Cctb | 12461 | 1.1925 | 0.25396 | 0.005599 | 2.2519 |
| P31786 | ACBP_MOUSE | Acyl-CoA-binding protein (ACBP) (Diazepam-binding inhibitor) (DBI) | Dbi | 13167 | 1.1513 | 0.20326 | 0.007813 | 2.1072 |
| Q7M6Y3 | PICAL_MOUSE | Phosphatidylinositol-binding clathrin assembly protein (CALM) | Picalm Calm Fit1 | 233489 | 1.1503 | 0.20204 | 0.009291 | 2.0319 |
| Q8CI94 | PYGB_MOUSE | Glycogen phosphorylase, brain form | Pygb | 110078 | 1.116 | 0.15838 | 0.001782 | 2.7492 |
| P62301 | RS13_MOUSE | 40S ribosomal protein S13 | Rps13 | 68052 | 1.1073 | 0.14704 | 0.006763 | 2.1699 |
| Q64133 | AOFA_MOUSE | Amine oxidase [flavin-containing] A (EC 1.4.3.4) (Monoamine oxidase type A) (MAO-A) | Maoa | 17161 | 1.1059 | 0.14516 | 0.006301 | 2.2006 |
| P14206 | RSSA_MOUSE | 40S ribosomal protein SA (37 kDa laminin receptor precursor) (37LRP) | Rpsa Lamr1 P40-8 | 16785 | 1.093 | 0.12834 | 0.005205 | 2.2836 |
| P14869 | RLA0_MOUSE | 60S acidic ribosomal protein P0 (60S ribosomal protein L10E) | Rplp0 Arbp | 11837 | 1.077 | 0.10703 | 0.004379 | 2.3587 |
| Q8VIJ6 | SFPQ_MOUSE | Splicing factor, proline- and glutamine-rich (DNA-binding p52/p100 complex) | Sfpq Psf | 71514 | 0.93289 | -0.10022 | 0.009792 | 2.0091 |
| P50396 | GDIA_MOUSE | Rab GDP dissociation inhibitor alpha (Rab GDI alpha) | Gdi1 Rabgdia | 14567 | 0.91944 | -0.12117 | 0.002626 | 2.5807 |
| Q60864 | STIP1_MOUSE | Stress-induced-phosphoprotein 1 (STI1) (Hsc70/Hsp90-organizing protein) | Stip1 | 20867 | 0.91932 | -0.12136 | 0.004949 | 2.3055 |
| P62858 | RS28_MOUSE | 40S ribosomal protein S28 | Rps28 | 54127 | 0.91919 | -0.12157 | 0.005545 | 2.2561 |
| Q9QYB8 | ADDB_MOUSE | Beta-adducin (Add97) (Erythrocyte adducin subunit beta) | Add2 | 11519 | 0.91619 | -0.12628 | 0.000305 | 3.5164 |
| P47757 | CAPZB_MOUSE | F-actin-capping protein subunit beta (CapZ beta) | Capzb Cappb1 | 12345 | 0.91494 | -0.12825 | 0.007829 | 2.1063 |
| Q9CQZ5 | NDUA6_MOUSE | NADH dehydrogenase 1 alpha subcomplex subunit 6 (Complex I-B14) | Ndufa6 | 67130 | 0.91473 | -0.12858 | 0.007995 | 2.0972 |
| P32037 | GTR3_MOUSE | Glucose transporter type 3, brain (GLUT-3) | Slc2a3 Glut3 | 20527 | 0.91012 | -0.13587 | 0.001334 | 2.8749 |
| G5E829 | AT2B1_MOUSE | Plasma membrane calcium-transporting ATPase 1 | Atp2b1 | 67972 | 0.90982 | -0.13635 | 0.008957 | 2.0478 |
| Q9CZY3 | UB2V1_MOUSE | Ubiquitin-conjugating enzyme E2 variant 1 (UEV-1) (CROC-1) | Ube2v1 Croc1 | 66589 | 0.90276 | -0.14759 | 0.005311 | 2.2748 |
| P62715 | PP2AB_MOUSE | Serine/threonine-protein phosphatase 2A (PP2A-beta) | Ppp2cb | 19053 | 0.89797 | -0.15526 | 0.009378 | 2.0279 |
| P62702 | RS4X_MOUSE | 40S ribosomal protein S4, X isoform | Rps4x Rps4 | 20102 | 0.8857 | -0.17511 | 0.006742 | 2.1712 |
| Q810U4 | NRCAM_MOUSE | Neuronal cell adhesion molecule (Nr-CAM/ (Ng-CAM-related) | Nrcam Kiaa0343 | 319504 | 0.88113 | -0.18258 | 0.006673 | 2.1757 |
| Q8BUV3 | GEPH_MOUSE | Gephyrin | Gphn | 268566 | 0.88096 | -0.18285 | 0.009165 | 2.0379 |
| Q8CGK3 | LONM_MOUSE | Mitochondrial ATP-dependent protease Lon) (Serine protease 15) | Lonp1 Prss15 | 74142 | 0.88092 | -0.18292 | 0.008196 | 2.0864 |
| O35465 | FKBP8_MOUSE | Peptidyl-prolyl cis-trans isomerase FKBP8 (PPIase FKBP8) | Fkbp8 Fkbp38 Sam11 | 14232 | 0.88006 | -0.18432 | 0.000143 | 3.8451 |
| P19783 | COX41_MOUSE | Cytochrome c oxidase subunit 4 isoform 1, mitochondrial | Cox4i1 Cox4 Cox4a | 12857 | 0.87942 | -0.18537 | 0.008333 | 2.0792 |
| P61161 | ARP2_MOUSE | Actin-related protein 2 (Actin-like protein 2) | Actr2 Arp2 | 66713 | 0.87771 | -0.18819 | 0.009737 | 2.0116 |
| P84084 | ARF5_MOUSE | ADP-ribosylation factor 5 | Arf5 | 11844 | 0.87539 | -0.192 | 0.006693 | 2.1744 |
| Q9JKR6 | HYOU1_MOUSE | Hypoxia up-regulated protein 1 (GRP-170) (140 kDa Ca(2+)-binding protein) (CBP-140) | Hyou1 Grp170 | 12282 | 0.87416 | -0.19403 | 0.009546 | 2.0202 |
| P62245 | RS15A_MOUSE | 40S ribosomal protein S15a | Rps15a | 267019 | 0.87323 | -0.19557 | 0.009769 | 2.0102 |
| P06880 | SOMA_MOUSE | Somatotropin (Growth hormone) | Gh1 Gh | 14599 | 0.86222 | -0.21388 | 0.006032 | 2.2195 |
| P63321 | RALA_MOUSE | Ras-related protein Ral-A | Rala Ral Ral-a | 56044 | 0.85917 | -0.21898 | 0.002371 | 2.6251 |
| P26883 | FKB1A_MOUSE | Peptidyl-prolyl cis-trans isomerase FKBP1A (PPIase FKBP1A) | Fkbp1a Fkbp1 | 14225 | 0.85709 | -0.22248 | 0.009919 | 2.0035 |
| P48774 | GSTM5_MOUSE | Glutathione S-transferase Mu 5 | Gstm5 Fsc2 Gstm3 | 14866 | 0.85562 | -0.22497 | 0.009345 | 2.0294 |
| P35803 | GPM6B_MOUSE | Neuronal membrane glycoprotein M6-b (M6b) | Gpm6b M6b | 14758 | 0.855 | -0.22601 | 0.006105 | 2.2143 |
| Q80XN0 | BDH_MOUSE | D-beta-hydroxybutyrate dehydrogenase, mitochondrial, (3-hydroxybutyrate dehydrogenase) (BDH) | Bdh1 Bdh | 71911 | 0.8533 | -0.22887 | 0.008734 | 2.0588 |
| Q8K3J1 | NDUS8_MOUSE | NADH dehydrogenase iron-sulfur protein 8, mitochondrial | Ndufs8 | 225887 | 0.84695 | -0.23965 | 0.000658 | 3.1817 |
| Q60854 | SPB6_MOUSE | Serpin B6 (Placental thrombin inhibitor) (Proteinase inhibitor 6) (PI-6) | Serpinb6 Serpinb6a Spi3 | 20719 | 0.84473 | -0.24344 | 0.007476 | 2.1264 |
| Q9JKD3 | SCAM5_MOUSE | Secretory carrier-associated membrane protein 5 | Scamp5 | 56807 | 0.841 | -0.24982 | 0.009545 | 2.0202 |
| P35279 | RAB6A_MOUSE | Ras-related protein Rab-6A (Rab-6) | Rab6a Rab6 MNCb-1660 | 19346 | 0.83945 | -0.25249 | 0.001006 | 2.9975 |
| O08788 | DCTN1_MOUSE | Dynactin subunit 1 (DAP-150) | Dctn1 | 13191 | 0.83734 | -0.25611 | 0.00038 | 3.4202 |
| P68404 | KPCB_MOUSE | Protein kinase C beta type (PKC-B) | Prkcb Pkcb Prkcb1 | 18751 | 0.83695 | -0.25679 | 0.002678 | 2.5722 |
| Q9JI46 | NUDT3_MOUSE | Diphosphoinositol polyphosphate phosphohydrolase 1 (DIPP-1) | Nudt3 Dipp Dipp1 | 56409 | 0.83296 | -0.26368 | 0.004246 | 2.372 |
| Q99KB8 | GLO2_MOUSE | Hydroxyacylglutathione hydrolase, mitochondrial, (Glyoxalase II) (Glx II) | Hagh Glo2 | 14651 | 0.83202 | -0.26531 | 0.002899 | 2.5378 |
| P54823 | DDX6_MOUSE | Probable ATP-dependent RNA helicase DDX6 | Ddx6 Hlr2 Rck | 13209 | 0.81921 | -0.2877 | 0.003637 | 2.4393 |
| Q64105 | SPRE_MOUSE | Sepiapterin reductase (SPR) (EC 1.1.1.153) | Spr |  | 0.81506 | -0.29503 | 0.002494 | 2.603 |
| Q8C0E2 | VP26B_MOUSE | Vacuolar protein sorting-associated protein 26B | Vps26b | 69091 | 0.81332 | -0.29811 | 0.000534 | 3.2728 |
| Q8R0S2 | IQEC1_MOUSE | IQ motif and SEC7 domain-containing protein 1 | Iqsec1 Kiaa0763 | 232227 | 0.80803 | -0.30752 | 0.00277 | 2.5575 |
| P63054 | PCP4_MOUSE | Calmodulin regulator protein PCP4 (Brain-specific antigen PCP-4) | Pcp4 Pep19 | 18546 | 0.80477 | -0.31336 | 0.001173 | 2.9308 |
| Q9JM76 | ARPC3_MOUSE | Actin-related protein 2/3 complex subunit 3 (Arp2/3 complex 21 kDa subunit) | Arpc3 | 56378 | 0.78231 | -0.35419 | 0.002433 | 2.6139 |
| P63040 | CPLX1_MOUSE | Complexin-1 (921-S) (Complexin I) (CPX I) (Synaphin-2) | Cplx1 | 12889 | 0.77992 | -0.3586 | 0.008389 | 2.0763 |
| Q62188 | DPYL3_MOUSE | Dihydropyrimidinase-related protein 3 (DRP-3/ ULIP-1) | Dpysl3 Drp3 Ulip | 22240 | 0.76198 | -0.39218 | 0.000431 | 3.3657 |
| Q7TNP2 | 2AAB_MOUSE | Serine/threonine-protein phosphatase 2A (PP2A subunit A isoform PR65-beta) | Ppp2r1b | 73699 | 0.74324 | -0.42809 | 0.007634 | 2.1173 |
| Q9Z0H4 | CELF2_MOUSE | CUGBP Elav-like family member 2 (CELF-2) (Neuroblastoma apoptosis-related RNA-binding protein) | Celf2 Cugbp2 Napor | 14007 | 0.73153 | -0.45101 | 0.000751 | 3.1243 |
| Q62283 | TSN7_MOUSE | Tetraspanin-7 (Tspan-7) (Cell surface glycoprotein A15) | Tspan7 Mxs1 Tm4sf2 | 21912 | 0.73152 | -0.45104 | 0.004656 | 2.332 |
| P62071 | RRAS2_MOUSE | Ras-related protein R-Ras2 | Rras2 | 66922 | 0.72895 | -0.45612 | 0.00109 | 2.9627 |
| P47962 | RL5_MOUSE | 60S ribosomal protein L5 | Rpl5 | 1.01E+08 | 0.72716 | -0.45966 | 0.003159 | 2.5004 |
| P30416 | FKBP4_MOUSE | Peptidyl-prolyl cis-trans isomerase FKBP4 (PPIase FKBP4) | Fkbp4 Fkpb52 | 14228 | 0.71958 | -0.47477 | 0.000363 | 3.4399 |
| Q9WTL7 | LYPA2_MOUSE | Acyl-protein thioesterase 2 (APT-2) | Lypla2 | 26394 | 0.71293 | -0.48817 | 0.005573 | 2.2539 |
| Q8CBW3 | ABI1_MOUSE | Abl interactor 1 (Abelson interactor 1) (Abi-1) | Abi1 Ssh3bp1 | 11308 | 0.69449 | -0.52597 | 0.000833 | 3.0792 |
| B1AWN6 | SCN2A_MOUSE | Sodium channel protein type 2 subunit alpha ( Nav1.2) | Scn2a Scn2a1 | 110876 | 0.69171 | -0.53176 | 0.005758 | 2.2397 |
| Q9ESJ4 | SPN90_MOUSE | NCK-interacting protein with SH3 domain | Nckipsd Spin90 Wasbp | 80987 | 0.68625 | -0.54319 | 0.001364 | 2.8651 |
| Q8R2R9 | AP3M2_MOUSE | AP-3 complex subunit mu-2(Clathrin coat-associated protein AP47 homolog 2) | Ap3m2 | 64933 | 0.62897 | -0.66893 | 0.001388 | 2.8577 |
| Q9JJZ2 | TBA8_MOUSE | Tubulin alpha-8 chain (Alpha-tubulin 8) | Tuba8 | 53857 | 0.61052 | -0.7119 | 0.007258 | 2.1392 |
| P02088 | HBB1_MOUSE | Hemoglobin subunit beta-1 (Beta-1-globin) (Hemoglobin beta-1 chain) | Hbb-b1 | 100503605 101488143 15129 | 0.60319 | -0.72932 | 0.007105 | 2.1484 |
| O88741 | GDAP1_MOUSE | Ganglioside-induced differentiation-associated protein 1 (GDAP1) | Gdap1 | 14545 | 0.52789 | -0.92169 | 0.006218 | 2.2064 |
| P08414 | KCC4_MOUSE | Calcium/calmodulin-dependent protein kinase type IV (CaMK IV) | Camk4 |  | 0.5191 | -0.94592 | 0.003148 | 2.5019 |
| Q9D0M5 | DYL2_MOUSE | Dynein light chain 2, cytoplasmic | Dynll2 Dlc2 | 68097 | 0.47171 | -1.084 | 6.97E-05 | 4.1571 |
