# Supplementary material for: The Impacts of Surgery and Intracerebral Electrodes in C57BL/6J Mouse Kainate Model of Epileptogenesis: Seizure Threshold, Proteomics, and Cytokine Profiles
Source: Front Neurol. 2021 Jul 12;12:625017. doi: 10.3389/fneur.2021.625017 (PMC8312573; doi:10.3389/fneur.2021.625017)
Supplement: Supplementary Table 3 — The impact of KA-induced SE in intracerebral electrode implanted animals on the expression of proteins in the hippocampus. The groups compared were between surgery vs. no surgery treated with KA, and all the proteins that were significantly altered by p > 0.01 are listed and the proteins with >2-fold change are highlighted. [file Table_3.docx]

| **Uniprot ID** | **Gene**  **Symbol** | **Protein**  **names** | **Gene names** | **KEGG-ID (mmu)** | **Fold change** | **log2(FC)** | **P value** | **neg log10(p)** |
| --- | --- | --- | --- | --- | --- | --- | --- | --- |
| E9PV24 | FIBA_MOUSE | Fibrinogen alpha chain | Fga | 14161 | 5.9018 | 2.5612 | 0.005928 | 2.2271 |
| Q61838 | PZP_MOUSE | Pregnancy zone protein (Alpha-2-macroglobulin) | Pzp A2m |  | 3.7671 | 1.9135 | 0.009382 | 2.0277 |
| P07724 | ALBU_MOUSE | Serum albumin | Alb Alb-1 Alb1 | 11657 | 3.6877 | 1.8827 | 0.001262 | 2.899 |
| Q921I1 | TRFE_MOUSE | Serotransferrin (Transferrin) (Beta-1 metal-binding globulin) | Tf Trf | 22041 | 3.3186 | 1.7306 | 0.000404 | 3.3938 |
| Q00897 | A1AT4_MOUSE | Alpha-1-antitrypsin 1-4 (Alpha-1 protease inhibitor 4) | Serpina1d Dom4 Spi1-4 | 20703 | 2.5277 | 1.3378 | 0.008136 | 2.0896 |
| Q8CIG9 | FBXL8_MOUSE | F-box/LRR-repeat protein 8 | Fbxl8 Fbl8 | 50788 | 2.521 | 1.334 | 0.000421 | 3.3757 |
| P14106 | C1QB_MOUSE | Complement C1q subcomponent subunit B | C1qb | 12260 | 2.3267 | 1.2183 | 0.007832 | 2.1061 |
| P55012 | S12A2_MOUSE | Solute carrier family 12 member 2 (Na-K-Cl symporter) | Slc12a2 Nkcc1 | 20496 | 2.2689 | 1.182 | 0.001829 | 2.7379 |
| Q6A026 | PDS5A_MOUSE | Sister chromatid cohesion protein PDS5 homolog A | Pds5a Kiaa0648 |  | 2.0361 | 1.0258 | 0.000415 | 3.3817 |
| Q8BTM8 | FLNA_MOUSE | Filamin-A (FLN-A) (Actin-binding protein 280) | Flna Fln Fln1 | 192176 | 2.0359 | 1.0257 | 0.006744 | 2.1711 |
| Q9DBY0 | FOXP4_MOUSE | Forkhead box protein P4 (Fork head-related protein-like A) | Foxp4 | 74123 | 1.9203 | 0.94132 | 0.002336 | 2.6316 |
| Q3URK3 | TET1_MOUSE | Methylcytosine dioxygenase TET1 (CXXC-6) | Tet1 Cxxc6 Kiaa1676 |  | 1.8305 | 0.87223 | 0.000391 | 3.4077 |
| P13020 | GELS_MOUSE | Gelsolin (Actin-depolymerizing factor) (ADF) (Brevin) | Gsn Gsb | 227753 | 1.8185 | 0.86274 | 0.001637 | 2.786 |
| Q8VHL1 | SETD7_MOUSE | Histone-lysine N-methyltransferase SETD7 | Setd7 Kiaa1717 Set7 Set9 | 73251 | 1.7726 | 0.82586 | 6.43E-05 | 4.1915 |
| P52760 | RIDA_MOUSE | Heat-responsive protein 12 | Rida Hrp12 | 15473 | 1.7555 | 0.81184 | 0.005336 | 2.2728 |
| P02468 | LAMC1_MOUSE | Laminin subunit gamma-1 | Lamc1 Lamb-2 Lamc-1 |  | 1.7517 | 0.80876 | 0.000599 | 3.2224 |
| P35505 | FAAA_MOUSE | Fumarylacetoacetase (FAA) | Fah | 14085 | 1.6933 | 0.75982 | 6.80E-06 | 5.1676 |
| Q9DB73 | NB5R1_MOUSE | NADH-cytochrome b5 reductase 1 | Cyb5r1 Nqo3a2 | 72017 | 1.6409 | 0.71447 | 0.00487 | 2.3125 |
| Q9Z1Z0 | USO1_MOUSE | General vesicular transport factor p115 (Vesicle-docking protein) | Uso1 Vdp | 56041 | 1.619 | 0.69511 | 0.004089 | 2.3884 |
| P10605 | CATB_MOUSE | Cathepsin B | Ctsb | 13030 | 1.4724 | 0.55821 | 0.004661 | 2.3315 |
| Q5SSL4 | ABR_MOUSE | Active breakpoint cluster region-related protein | Abr | 109934 | 1.4584 | 0.54439 | 0.000488 | 3.3113 |
| Q9CQJ6 | DENR_MOUSE | Density-regulated protein (DRP) | Denr | 68184 | 1.4251 | 0.51102 | 0.003575 | 2.4467 |
| Q6PB66 | LPPRC_MOUSE | Leucine-rich PPR motif-containing protein, mitochondrial | Lrpprc Lrp130 | 72416 | 1.4005 | 0.48591 | 0.000576 | 3.2394 |
| Q8R001 | MARE2_MOUSE | Microtubule-associated protein RP (APC-binding protein EB2) | Mapre2 | 212307 | 1.3985 | 0.48391 | 0.00707 | 2.1506 |
| Q8BGT8 | PHIPL_MOUSE | Phytanoyl-CoA hydroxylase-interacting protein-like | Phyhipl | 70911 | 1.398 | 0.48336 | 0.005824 | 2.2348 |
| Q6NZJ6 | IF4G1_MOUSE | Eukaryotic translation initiation factor 4 gamma 1 | Eif4g1 | 208643 | 1.3752 | 0.45964 | 0.008902 | 2.0505 |
| O08547 | SC22B_MOUSE | Vesicle-trafficking protein SEC22b (ER-Golgi SNARE of 24 kDa) | Sec22b Sec22l1 | 20333 | 1.2833 | 0.35982 | 0.00082 | 3.086 |
| Q9D1A2 | CNDP2_MOUSE | Glutamate carboxypeptidase-like protein 1 | Cndp2 Cn2 | 66054 | 1.1882 | 0.24874 | 0.002309 | 2.6366 |
| Q9D8N0 | EF1G_MOUSE | Elongation factor 1-gamma (EF-1-gamma) (eEF-1B gamma) | Eef1g | 67160 | 1.1733 | 0.23059 | 0.003603 | 2.4434 |
| P40142 | TKT_MOUSE | Transketolase (P68) | Tkt | 21881 | 1.1526 | 0.20489 | 0.004398 | 2.3568 |
| P80315 | TCPD_MOUSE | T-complex protein 1 subunit delta (TCP-1-delta) (A45) (CCT-delta) | Cct4 Cctd | 12464 | 1.1523 | 0.20451 | 0.002837 | 2.5472 |
| P35564 | CALX_MOUSE | Calnexin | Canx | 12330 | 1.1212 | 0.16502 | 0.009241 | 2.0343 |
| Q9CZX8 | RS19_MOUSE | 40S ribosomal protein S19 | Rps19 | 20085 | 1.1179 | 0.16085 | 0.007796 | 2.1081 |
| P67778 | PHB_MOUSE | Prohibitin (B-cell receptor-associated protein 32) (BAP 32) | Phb | 18673 | 1.0935 | 0.12897 | 0.007679 | 2.1147 |
| P62301 | RS13_MOUSE | 40S ribosomal protein S13 | Rps13 | 68052 | 1.0885 | 0.12237 | 0.00751 | 2.1244 |
| P28663 | SNAB_MOUSE | Beta-soluble NSF attachment protein (SNAP-beta) (Brain protein I47) | Napb Snapb | 17957 | 0.93704 | -0.09381 | 0.006164 | 2.2101 |
| Q60864 | STIP1_MOUSE | Stress-induced-phosphoprotein 1 (STI1) (mSTI1) (Hsc70/Hsp90) | Stip1 | 20867 | 0.91156 | -0.1336 | 0.009315 | 2.0308 |
| Q8R3V5 | SHLB2_MOUSE | Endophilin-B2 (SH3 domain-containing GRB2-like protein B2) | Sh3glb2 Kiaa1848 | 227700 | 0.89605 | -0.15835 | 0.002191 | 2.6594 |
| P62259 | 1433E_MOUSE | 14-3-3 protein epsilon (14-3-3E) | Ywhae | 22627 | 0.88604 | -0.17456 | 0.005584 | 2.253 |
| P46460 | NSF_MOUSE | Vesicle-fusing ATPase | Nsf Skd2 | 18195 | 0.88597 | -0.17467 | 0.006715 | 2.1729 |
| Q9QYB8 | ADDB_MOUSE | Beta-adducin (Add97) (Erythrocyte adducin subunit beta) | Add2 | 11519 | 0.87864 | -0.18666 | 0.00804 | 2.0947 |
| Q80XI4 | PI42B_MOUSE | Phosphatidylinositol 5-phosphate 4-kinase type-2 beta | Pip4k2b Pip5k2b | 108083 | 0.87677 | -0.18973 | 0.002466 | 2.608 |
| Q8BJI1 | S6A17_MOUSE | Sodium-dependent neutral amino acid transporter SLC6A17 | Slc6a17 Ntt4 | 229706 | 0.87073 | -0.1997 | 0.009229 | 2.0348 |
| Q9EPW0 | INP4A_MOUSE | Inositol polyphosphate-4-phosphatase type I A | Inpp4a | 269180 | 0.86561 | -0.2082 | 0.005889 | 2.23 |
| Q61411 | RASH_MOUSE | GTPase HRas (H-Ras-1) (Transforming protein p21) | Hras Hras1 | 15461 | 0.86552 | -0.20835 | 0.007567 | 2.1211 |
| Q8K021 | SCAM1_MOUSE | Secretory carrier-associated membrane protein 1 | Scamp1 | 107767 | 0.86149 | -0.2151 | 0.005513 | 2.2586 |
| P0DP26 | CALM1_MOUSE | Calmodulin-1 | Calm1 Calm Cam Cam1 | 1.23E+14 | 0.85188 | -0.23128 | 0.003264 | 2.4862 |
| Q9DBP5 | KCY_MOUSE | UMP-CMP kinase | Cmpk1 Cmk Cmpk Uck Umk Umpk | 66588 | 0.84907 | -0.23604 | 0.004069 | 2.3905 |
| Q8C0E2 | VP26B_MOUSE | Vacuolar protein sorting-associated protein 26B | Vps26b | 69091 | 0.84583 | -0.24156 | 0.000803 | 3.0953 |
| P46096 | SYT1_MOUSE | Synaptotagmin-1 (Synaptotagmin I) (SytI) (p65) | Syt1 | 20979 | 0.84558 | -0.24199 | 0.005802 | 2.2364 |
| Q9Z2H5 | E41L1_MOUSE | Band 4.1-like protein 1 (Neuronal protein 4.1) (4.1N) | Epb41l1 Epb4 Epb4.1l1 Kiaa0338 | 13821 | 0.83269 | -0.26414 | 0.001825 | 2.7389 |
| Q80VP1 | EPN1_MOUSE | Epsin-1 (EPS-15-interacting protein 1) | Epn1 | 13854 | 0.83032 | -0.26826 | 0.005008 | 2.3004 |
| P61161 | ARP2_MOUSE | Actin-related protein 2 (Actin-like protein 2) | Actr2 Arp2 | 66713 | 0.82806 | -0.2722 | 0.002874 | 2.5416 |
| P39053 | DYN1_MOUSE | Dynamin-1 (EC 3.6.5.5) | Dnm1 Dnm Kiaa4093 | 13429 | 0.81844 | -0.28905 | 0.001452 | 2.8379 |
| Q8BK64 | AHSA1_MOUSE | Activator of 90 kDa heat shock protein ATPase homolog 1 (AHA1) | Ahsa1 | 217737 | 0.81202 | -0.30041 | 0.000718 | 3.1437 |
| O08532 | CA2D1_MOUSE | Voltage-dependent calcium channel subunit alpha-2/delta-1 | Cacna2d1 Cacna2 | 12293 | 0.81131 | -0.30167 | 0.001721 | 2.7643 |
| O70161 | PI51C_MOUSE | Phosphatidylinositol 4-phosphate 5-kinase type-1 gamma | Pip5k1c Kiaa0589 | 18717 | 0.80765 | -0.30821 | 0.001577 | 2.8022 |
| Q9D1G1 | RAB1B_MOUSE | Ras-related protein Rab-1B | Rab1b | 76308 | 0.79625 | -0.3287 | 0.005767 | 2.2391 |
| P28660 | NCKP1_MOUSE | Nck-associated protein 1 (NAP 1) (Brain protein H19) | Nckap1 Hem2 Kiaa0587 Nap1 | 50884 | 0.79547 | -0.33012 | 0.000689 | 3.1621 |
| Q8CGK3 | LONM_MOUSE | Mitochondrial ATP-dependent protease Lon) (Serine protease 15) | Lonp1 Prss15 | 74142 | 0.79445 | -0.33198 | 0.00923 | 2.0348 |
| P62823 | RAB3C_MOUSE | Ras-related protein Rab-3C | Rab3c | 67295 | 0.79155 | -0.33724 | 0.001443 | 2.8409 |
| Q9CPQ3 | TOM22_MOUSE | Mitochondrial import receptor subunit TOM22 | Tomm22 Tom22 | 223696 | 0.78587 | -0.34763 | 0.006501 | 2.1871 |
| P11798 | KCC2A_MOUSE | Calcium/calmodulin-dependent protein kinase type II subunit alpha | Camk2a | 12322 | 0.77971 | -0.35899 | 0.003565 | 2.4479 |
| Q6ZWV3 | RL10_MOUSE | 60S ribosomal protein L10 | Rpl10 Qm | 110954 | 0.77909 | -0.36015 | 0.005698 | 2.2443 |
| P84086 | CPLX2_MOUSE | Complexin-2 (921-L) (Complexin II) (CPX II) (Synaphin-1) | Cplx2 | 12890 | 0.77847 | -0.36129 | 0.008533 | 2.0689 |
| P62071 | RRAS2_MOUSE | Ras-related protein R-Ras2 | Rras2 | 66922 | 0.77517 | -0.36741 | 0.006548 | 2.1839 |
| Q9Z2W9 | GRIA3_MOUSE | Glutamate receptor 3 (GluR-3/AMPA 3) | Gria3 Glur3 Kiaa4184 | 53623 | 0.76787 | -0.38107 | 0.004743 | 2.3239 |
| Q9ESJ4 | SPN90_MOUSE | NCK-interacting protein with SH3 domain | Nckipsd Spin90 Wasbp | 80987 | 0.7668 | -0.38307 | 0.001694 | 2.771 |
| P32037 | GTR3_MOUSE | Glucose transporter type 3, brain) (GLUT-3) | Slc2a3 Glut3 | 20527 | 0.76495 | -0.38657 | 0.000791 | 3.1018 |
| Q62283 | TSN7_MOUSE | Tetraspanin-7 (Tspan-7) (Cell surface glycoprotein A15) | Tspan7 Mxs1 Tm4sf2 | 21912 | 0.76046 | -0.39506 | 0.001355 | 2.8681 |
| P60879 | SNP25_MOUSE | Synaptosomal-associated protein 25 (SNAP-25) | Snap25 Snap | 20614 | 0.75293 | -0.40941 | 0.00413 | 2.3841 |
| Q91VR7 | MLP3A_MOUSE | Microtubule-associated proteins 1A/1B light chain 3A | Map1lc3a | 66734 | 0.72419 | -0.46556 | 0.006216 | 2.2065 |
| Q8CBW3 | ABI1_MOUSE | Abl interactor 1 (Abelson interactor 1) (Abi-1) (Ablphilin-1) | Abi1 Ssh3bp1 | 11308 | 0.72193 | -0.47006 | 0.004061 | 2.3914 |
| Q641P0 | ARP3B_MOUSE | Actin-related protein 3B (ARP3-beta) (Actin-like protein 3B) | Actr3b | 242894 | 0.72024 | -0.47346 | 0.004502 | 2.3466 |
| Q9R1Q9 | VAS1_MOUSE | V-type proton ATPase subunit S1 (V-ATPase subunit S1) | Atp6ap1 Atp6ip1 Atp6s1 | 54411 | 0.69498 | -0.52496 | 0.007797 | 2.1081 |
| Q80U28 | MADD_MOUSE | MAP kinase-activating death domain protein | Madd Kiaa0358 | 228355 | 0.67852 | -0.55954 | 0.002866 | 2.5427 |
| Q62188 | DPYL3_MOUSE | Dihydropyrimidinase-related protein 3 (DRP-3) | Dpysl3 Drp3 Ulip | 22240 | 0.66806 | -0.58196 | 0.004702 | 2.3278 |
| Q8JZS0 | LIN7A_MOUSE | Protein lin-7 homolog A (Lin-7A) | Lin7a Mals1 Veli1 | 108030 | 0.64069 | -0.6423 | 0.002113 | 2.6751 |
| B1AWN6 | SCN2A_MOUSE | Sodium channel protein type 2 subunit alpha (Nav1.2) | Scn2a Scn2a1 | 110876 | 0.63528 | -0.65453 | 0.003452 | 2.4619 |
| Q9D0M5 | DYL2_MOUSE | Dynein light chain 2, cytoplasmic | Dynll2 Dlc2 | 68097 | 0.43805 | -1.1908 | 0.000108 | 3.9667 |
