# Supplementary material for: The Impacts of Surgery and Intracerebral Electrodes in C57BL/6J Mouse Kainate Model of Epileptogenesis: Seizure Threshold, Proteomics, and Cytokine Profiles
Source: Front Neurol. 2021 Jul 12;12:625017. doi: 10.3389/fneur.2021.625017 (PMC8312573; doi:10.3389/fneur.2021.625017)
Supplement: Supplementary Table 4 — The list of KEGG pathways and the key proteins of the pathways affected by intracerebral electrode implants. The groups compared were between surgery vs. no surgery and treated with the vehicle (distilled water). [file Table_4.docx]

| **Term** | **#** | **%** | **PVal** | **Genes** | **Total** | **Hits** | **Total** | **Fold Enrich** | **Bonfer** | **Benjam** | **FDR** |
| --- | --- | --- | --- | --- | --- | --- | --- | --- | --- | --- | --- |
| mmu05020:Prion diseases | 5 | 4.86 | 0.0001 | Q60864 (Stip1), P02468 (lamc1), P14106 (C1qb), Q02105 (C1qc), P98086 (C1qa) | 62 | 33 | 7691 | 18.79521 | 0.016886 | 0.016886 | 0.145138 |
| mmu03010:Ribosome | 7 | 6.80 | 0.0001 | P47962 (Rpl5), P62858 (Rps28), P14869 (Rplp0), P62301 (Rps13), P62245 (Rps15a), P62702 (Rps4x), P14206 (Rpsa Lamr1) | 62 | 145 | 7691 | 5.988543 | 0.123881 | 0.063988 | 1.121572 |
| mmu05142:Chagas disease | 5 | 4.85 | 0.0088 | P62715 (Ppp2cb),Q7TNP2 (Ppp2r1b), P14106 (C1qa), Q02105 (C1qb), P98086 (C1qc) | 62 | 103 | 7691 | 6.021766 | 0.705844 | 0.334942 | 9.909686 |
| mmu04970:Salivary secretion | 4 | 3.88 | 0.0229 | P55012 (Nkcc1), P68404 (Prkcb1), P21460 (Cst3), G5E829 (Atp2b1) | 62 | 77 | 7691 | 6.444072 | 0.959271 | 0.550764 | 23.88919 |
| mmu00480:Glutathione metabolism | 3 | 2.91 | 0.0503 | P48774 (Gstm5), P54071 (Idh2), Q9CPY7 (lap3) | 62 | 55 | 7691 | 6.766276 | 0.999957 | 0.812996 | 57.59651 |
| mmu04730:Long-term depression | 3 | 2.91 | 0.0840 | P62715 (Ppp2cb), P68404 (Prkcb1), Q7TNP2 (Pp2r1b) | 62 | 61 | 7691 | 6.10074 | 0.999995 | 0.823019 | 64.43525 |
| mmu04728:Dopaminergic synapse | 4 | 3.88 | 0.0898 | Q64133 (Maoa), P62715 (Ppp2cb), P68404 (Prkcb1), Q7TNP2 (Pp2r1b) | 62 | 134 | 7691 | 3.702937 | 0.999998 | 0.80289 | 66.97842 |
| mmu05031:Amphetamine addiction | 3 | 2.91 | 0.0986 | Q64133 (Maoa), P68404 (Prkcb1), P08414 (Camk4) | 62 | 67 | 7691 | 5.554405 | 0.999999 | 0.796521 | 70.53896 |
