# Supplementary material for: The Impacts of Surgery and Intracerebral Electrodes in C57BL/6J Mouse Kainate Model of Epileptogenesis: Seizure Threshold, Proteomics, and Cytokine Profiles
Source: Front Neurol. 2021 Jul 12;12:625017. doi: 10.3389/fneur.2021.625017 (PMC8312573; doi:10.3389/fneur.2021.625017)
Supplement: Supplementary Table 5 — The list of KEGG pathways and the key proteins of the pathways affected by intracerebral electrode implants and KA-induced SE. The groups compared were between surgery vs. no surgery and treated with KA. [file Table_5.docx]

| **KEGG Pathways** | **#** | **%** | **p val.** | **Genes/Proteins** | **List Total** | **# of Hits** | **Pop Total** | **Fold Enrich** | **Bonfe.** | **Benja** | **FDR** |
| --- | --- | --- | --- | --- | --- | --- | --- | --- | --- | --- | --- |
| mmu04721:Synaptic vesicle cycle | 5 | 6.25 | 0.0004. | P46096, P60879, P39053, P84086, P46460 | 46 | 62 | 7691 | 13.48 | 0.056279 | 0.056279 | 0.517549609 |
| mmu04810:Regulation of actin cytoskeleton | 6 | 7.5 | 0.0077 | P62071, P28660, Q61411, Q80XI4, P13020, O70161 | 46 | 213 | 7691 | 4.71 | 0.634667 | 0.395572 | 8.625421506 |
| mmu05020:Prion diseases | 3 | 3.75 | 0.0157 | Q60864, P02468, P14106 | 46 | 33 | 7691 | 15.20 | 0.873093 | 0.497470 | 16.88303856 |
| mmu04144:Endocytosis | 6 | 7.5 | 0.0174 | Q8R3V5, Q8C0E2, Q61411, P39053, Q80VP1, O70161 | 46 | 261 | 7691 | 3.84 | 0.899005 | 0.436265 | 18.56625634 |
| mmu04070:Phosphatidylinositol signaling system | 4 | 5 | 0.0188 | Q80XI4, P0DP26, O70161, Q9EPW0 | 46 | 97 | 7691 | 6.89 | 0.915486 | 0.389921 | 19.85553843 |
| mmu04722:Neurotrophin signaling pathway | 4 | 5 | 0.0340 | P11798, P62259, Q61411, P0DP26 | 46 | 122 | 7691 | 5.48 | 0.988995 | 0.528372 | 33.23257454 |
| mmu05134:Legionellosis | 3 | 3.75 | 0.0436 | Q9D1G1, O08547, Q9D8N0 | 46 | 57 | 7691 | 8.80 | 0.996958 | 0.563031 | 40.4970019 |
| mmu05214:Glioma | 3 | 3.75 | 0.0551 | P11798, Q61411, P0DP26 | 46 | 65 | 7691 | 7.72 | 0.999376 | 0.602465 | 48.37099387 |
| mmu04720:Long-term potentiation | 3 | 3.75 | 0.0567 | P11798, Q61411, P0DP26 | 46 | 66 | 7691 | 7.60 | 0.999494 | 0.569667 | 49.32850715 |
| mmu04921:Oxytocin signaling pathway | 4 | 5 | 0.0569 | P11798, Q61411, O08532, P0DP26 | 46 | 150 | 7691 | 4.46 | 0.999509 | 0.533185 | 49.46205562 |
| mmu05031:Amphetamine addiction | 3 | 3.75 | 0.0582 | P11798, Q9Z2W9, P0DP26 | 46 | 67 | 7691 | 7.49 | 0.99959 | 0.507913 | 50.27877397 |
| mmu00562:Inositol phosphate metabolism | 3 | 3.75 | 0.0629 | Q80XI4, O70161, Q9EPW0 | 46 | 70 | 7691 | 7.17 | 0.999786 | 0.50540 | 53.08274188 |
| mmu04610:Complement and coagulation cascades | 3 | 3.75 | 0.0726 | Q00897, E9PV24, P14106 | 46 | 76 | 7691 | 6.60 | 0.999945 | 0.529638 | 58.45383064 |
| mmu04912:GnRH signaling pathway | 3 | 3.75 | 0.0934 | P11798, Q61411, P0DP26 | 46 | 88 | 7691 | 5.70 | 0.999997 | 0.597821 | 68.09272507 |
